# The mediating role of general academic emotions in burnout and procrastination among Chinese medical undergraduates during the COVID-19 pandemic: A cross-sectional study

**DOI:** 10.3389/fpubh.2022.1011801

**Published:** 2022-12-05

**Authors:** Ruoyi Qu, Ning Ding, Honghe Li, Xinzhi Song, Zhangzhao Cong, Ruoxin Cai, Yaxin Zhu, Deliang Wen

**Affiliations:** ^1^Institute for International Health Professions Education and Research, China Medical University, Shenyang, China; ^2^Department of Teaching Affairs, China Medical University, Shenyang, China; ^3^The First Clinical Department, China Medical University, Shenyang, China

**Keywords:** COVID-19, general academic emotions, burnout, procrastination, medical undergraduates

## Abstract

**Background:**

Academic procrastination has become more prevalent during the COVID-19 pandemic due to teaching/learning changes. This phenomenon induces academic burnout, which is already serious among medical students. However, the academic emotion, which is the factor most vulnerable to changes in the academic environment, is still unknown. Therefore, the current study aimed to investigate the mediating role of general academic emotions in procrastination and burnout among Chinese medical students during the COVID-19 pandemic.

**Methods:**

This cross-sectional study enrolled 995 medical students from China Medical University. We applied the Chinese version of the Maslach Burnout Inventory Student Survey (MBI-SS), the Aitken Procrastination Inventory (API) and the General Academic Emotion Questionnaire for College Students (GAEQ) to evaluate the variables of interest. We examined the mediation effects of GAEs by hierarchical linear regression analysis.

**Results:**

Correlation analyses showed a significant positive correlation between procrastination and burnout. Procrastination and burnout positively and negatively correlated with negative academic emotions, respectively. Hierarchical linear regression analyses showed that procrastination had positive associations with negative academic emotions, while it had negative associations with positive academic emotions. The contributions (as mediators) of GAEs to burnout and procrastination were 21.16% (NAEs), 29.75% (PAEs), 54.25% (NDEs) and 23.69% (PDEs).

**Conclusions:**

The results indicate that academic emotions had mediating effects on procrastination and burnout. Medical students' worries about the uncertainty of the learning environment may have exacerbated academic burnout. Targeted improvements in the teaching environment to communicate encouragement and reduce anxiety and helplessness among medical undergraduates for implementing medical education while preventing and controlling the infection.

## Introduction

Due to travel limitations and closures of medical schools and universities, online learning (“zero contact”) has rapidly been accepted as the “new normal” (1–3) and has played a positive role in formal medical teaching/learning (4–6) worldwide during the coronavirus disease 2019 (COVID-19) pandemic. However, the virtual learning environment involves no actual interpersonal interactions, which has worsened existing challenges and created new barriers between students and teachers, especially psychological ones, such as variance in academic motivation, undetectable procrastination and reduced opportunity for psychological interventions (7–9). Therefore, identifying psychological risk factors is important to improve satisfaction with online teaching/learning (10).

Burnout is generally conceptualized as a prolonged response to chronic emotional and interpersonal workplace stress (11) and has three core dimensions: emotional exhaustion, cynicism (also referred to as depersonalization) and increasing feelings of inefficacy (12). The demand-control theory of Robert Karasek (13) explains the balance between psychological demands and available resources. Excessive labor or tense relationships may lead to low engagement and well-being, as well as suicidal tendencies (14), which explains the vulnerability to burnout among health professionals and medical undergraduates (15–17). According to a global meta-analysis of 17,431 medical students in 24 studies, the total burnout prevalence was estimated to be 44.2%, which is even higher than among residents (18). Emotional exhaustion was the most prevalent symptom (40.8%); depression, anxiety, suicidality and other emotional disturbances were also associated with burnout (19–21). Especially in the context of the COVID-19 pandemic, lack of family support caused by isolation, the extension of time spent on degree and suppressed enthusiasm for offline learning are all contributors to burnout among medical students (22, 23). A quantitative study with 741 training medical students from six U.S. medical schools suggested that, 74.7% of the participants agreed that COVID-19 had a great impact on their medical education, and 61.3% of the respondents were even willing to take the risk of illness to offset the burnout caused by the change of clinical activities (24). Although studies have focused on the impact of COVID-19 on the mental health of medical students (25), but there is still insufficient evidence to analyze influential factors and giving a better policy to relieve the anxiety emotions.

Extending previous studies (26), Steel (27) defined procrastination as “*the voluntary delay of an intended and necessary and/or [personally] important activity, despite expecting potential negative consequences that outweigh the positive consequences of the delay.”* Previous studies found that procrastinating prevalence among university students was double or triple that of the general population (28–30). Regarding medical professionals and undergraduates, similar to the burnout phenomenon mentioned above, procrastination, i.e., the needless delay of things that one intends to do, is also a major risk factor for low well-being (31). Evidence suggests that procrastination is positively correlated with academic anxiety (32), distress (33) and low motivation in students (34), resulting in more agitation before a test or poor academic performance across the entire semester. Besides the psychological aspects, a correlation between procrastination and the academic environment has also been demonstrated among medical students (35–37). Heavy dependency on the internet and lax time management may significantly contribute to the Internet or smartphone addiction (38, 39) during COVID-19 quarantine. This may exacerbate low well-being and increase the possibility of emotional disorders caused by procrastination (40). Although burnout and procrastination among medical students are positively correlated with adverse emotional factors and poor emotional management, few studies have discussed the relationship between burnout and procrastination.

General academic emotions (GAEs) have also been suggested to play a role in satisfaction with the learning environment and academic performance in medical campus (41–43). Pekrun's control-value theory (44) explained that subjective control over activities and their outcomes, as well as subjective appraisals of these activities and outcomes, are relevant to academic emotions. Desire and a clear expectation of success promote positive academic emotions and facilitate the self-discipline required to achieve good outcomes. Likewise, unavoidable failure or a lack of internal control result in negative academic emotions. Against the backdrop of the COVID-19 pandemic, online learning, lockdown of hospitals, inability to perform actual operations and many other major changes were unprecedented challenges to the provision of medical education (45). In the process of adapting to these changes involving peer interactions and learning evaluations, medical undergraduates are facing much uncertainty, which may disrupt academic emotions and achievement (46, 47).

In that case, we hypothesize that: (1) Procrastination is positively correlated with burnout among medical undergraduates based on their relationships with emotional factors and (2) GAEs play mediating roles in the relationship between burnout and procrastination among Chinese medical undergraduates. We assessed the association between burnout and procrastination among Chinese medical undergraduates studying at home during the COVID-19 pandemic, and the mediating effects of GAEs in the association of burnout with procrastination (after adjusting for the demographic variables and online learning preferences). Looking forward to addressing the concerns mentioned above and discovering the intervention targeted to improve mental health of medical undergraduates during the COVID-19 pandemic.

## Methods

### Study design and procedure

The Human Research Ethics Committee of China Medical University approved our study. All participants were familiarized with the study protocol before signing the consent form, and ethical principles were adhered to during the whole survey process. All information collected from participants was confidential and anonymous. We conducted this cross-sectional study of China Medical University (CMU) from August to September 2020. The questionnaire and consent forms were distributed online by scanning a QR code. In total, 1,045 medical undergraduates who had studied exclusively online in the spring semester during the COVID-19 pandemic voluntarily took part in our survey. Ultimately, 995 undergraduates completed the online questionnaire satisfactorily.

### Demographic variables

The medical undergraduates participating in the study were in their first to the fourth year, and were majoring in clinical medicine, preventive medicine, nursing, and medical technology. We collected demographic information including age, gender, and household registration. Online learning duration and preference data were also gathered. We applied the following measuring tools to assess burnout, procrastination and academic emotions.

### Measurement of burnout

Burnout among medical undergraduates was assessed using the Chinese version of the Student Burnout Inventory, adapted from the Maslach Burnout Inventory Student Survey (MBI-SS). This self-report scale contains 16 items, scored from one point (*strongly disagree*) to five points (*totally agree*) and classified into three dimensions including exhaustion, cynicism and professional efficacy. The exhaustion dimension consists of four items (items 2, 5, 8, and 12) and reflects fatigue resulting from the study. The cynicism dimension is composed of five items (items 3, 6, 9, 10, 13) and indicates a negative attitude toward studying. The professional efficacy dimension includes seven items (items 1, 4, 7, 11, 14, 15, 16) and is concerned with the sense of personal achievement during learning. The inventory has adequate reliability and validity for measuring Chinese samples (48). The Cronbach's alpha coefficient of our study was 0.876.

### Measurement of procrastination

We measured procrastination among medical undergraduates using the Chinese version of the Aitken Procrastination Inventory (API), which is a single- dimension scale including 19 items; item scores range from one point (*strongly disagree*) to five points (*totally agree*). This self-report scale evaluates undergraduates' long-term procrastination. The Chinese version of the API has proven reliability and validity (49). The Cronbach's alpha coefficient in our study was 0.905.

### Measurement of GAEs

The General Academic Emotion Questionnaire for College Students (GAEQ) was applied to evaluate academic emotions. The GAEQ is adapted from the Academic Emotion Questionnaire (AEQ) (50) and contains 88 items scored from one point (*strongly disagree*) to five points (*totally agree*). This self-report instrument measures 10 academic emotions including anxiety (15 items), boredom (13 items), relief (10 items), hopelessness (10 items), pride (9 items), shame (7 items), enjoyment (7 items), hope (7 items), anger (5 items) and interest (5 items). Based on the theory of Pekrun (51) and results of exploratory factor analysis, negative activating emotions (NAEs: shame, anxiety, and anger), positive activating emotions (PAEs: interest, enjoyment, and hope), negative deactivating emotions (NDEs: hopelessness and boredom) and positive deactivating emotions (PDEs: pride and relief) are distinguished. The acceptable reliability and validity of the GAEQ have been proven in Chinese college students (52) and the Cronbach's coefficient in the present study was 0.926.

### Statistical analysis

We report continuous variables as means with standard deviation (SD) and categorical variables as frequencies and percentages, based on descriptive analyses. We applied the *t*-test or one-way ANOVA to analyze burnout, procrastination and GAEs according to demographic factors. Pearson correlation analysis was used to identify correlations among burnout, procrastination and GAEs.

Binary logistic regression analysis was applied to assess the impact of GAEs and procrastination on burnout. Participants were divided into high- and low-burnout groups using the mean as the cut-off value. The quartile spacing method was used to categorize participants into degree groups (low, relatively low, relatively high and high), based on their GAEs and procrastination scores, to estimate relationships between burnout and specific components (exhaustion, cynicism and professional efficacy); odds ratios (ORs) and 95% confidence intervals (CIs) were generated.

**Figure 1:**
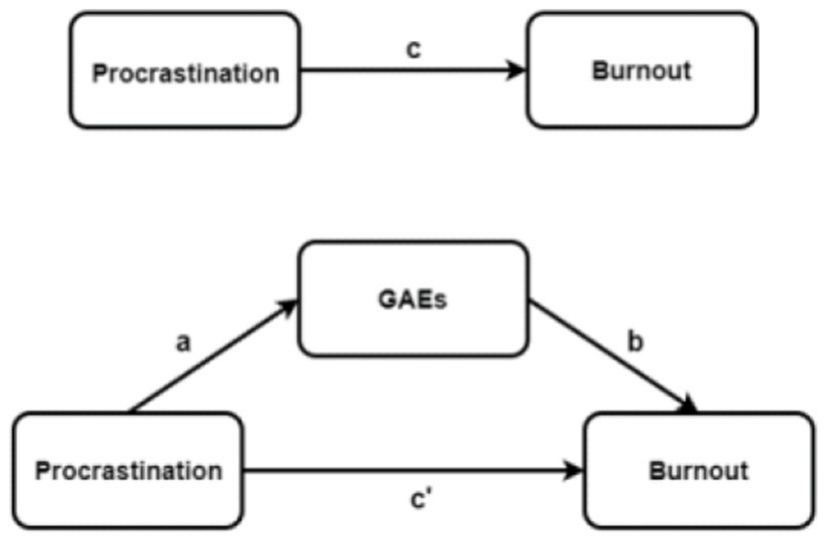
Procrastination, Burnout and GAEs in hierarchical linear regression analysis process among Chinese medical undergraduates. a, Association of procrastination with GAEs; b, association of GAEs with burnout; c, direct association between procrastination and burnout; c', association between procrastination and burnout with GAEs as mediators.

We examined the mediation effect of GAEs on the relationship between burnout and procrastination by hierarchical linear regression analysis. Procrastination was modeled as an independent variable, while burnout was the dependent variable. The enter and resampling methods were used to assess the mediating role of GAEs. Covariates included demographic variables and online learning preferences. Figure 1 presents the hierarchical linear regression analysis process. We performed bootstrap analysis (53) based on the process of Hayes (version 3.4.1). Five-thousand samples were bias-corrected and 95% CIs were generated for each GAE, to identify significant mediation effects.

All statistical analyses were conducted with SPSS 20.0 for Windows software (SPSS, Inc., Chicago, IL, USA). All tests were two-sided (α = 0.05). *P*-values < 0.05 and 95% CIs excluding zero were considered to indicate statistical significance.

### Rigor

We implemented several strategies to ensure the credibility of the results. Suggestions from experts majoring in medical education, social medicine and health management were taken into consideration at the design stage. Knowledgeable colleagues explained the study procedure to the participants before they filled out the questionnaire. All questionnaire items were mandatory, and maximum and minimum completion times were set to guarantee data quality. Participants who responded “totally agree” or “totally disagree” to all items were excluded. Double entry was applied in the data collation stage. A small-scale pre-experiment was performed before the formal investigation; any problems were recorded in detail.

## Results

### Participant characteristics

In total, 995 medical undergraduates from CMU were included in our survey. The average age of the participants was 19.83 ± 1.15 years and the majority were female (65.63%) and residing in an urban area (64.62%). The undergraduates mostly used notebook computers (65.33%) for online learning and normally spent 30–40 h (58.29%) engaged in distance learning per week. Table 1 shows the detailed demographic characteristics of all participants.

**Table 1:** Demographic characteristic of subjects.

**Variables**		**Numbers**	**Percentage (%)**
Gender	male	342	34.37
	female	653	65.63
Household registration	rural	352	35.38
	urban	643	64.62
Academic year	Year 1	461	46.33
	Year 2	365	36.68
	Year 3	115	11.56
	Year 4	54	5.43
Major	clinical medicine	615	61.81
	preventive medicine	251	25.23
	nursing	38	3.82
	medical technology	91	9.14
Equipment	Desktop computer	45	4.52
	Notebook computer	650	65.33
	Tablet computer	99	9.95
	Mobile phone	201	20.20
Online learning	<30 h	142	14.27
time weekly	30–35 h	305	30.65
	36–40 h	275	27.64
	41–45 h	152	15.28
	>46 h	121	12.16

### GAEs

There was significant sex difference in terms of the deactivation of academic emotions. Rural undergraduates' scored lower for PDEs than urban undergraduates, who scored higher for NDEs. Junior medical undergraduates scored highly for PAEs. Participants using desktop computers for online learning had the highest NAE scores. Undergraduates with higher PAE scores spent more time distance learning. All of the GAE results are presented in Table 2.

**Table 2:** Results of GAEQ among CMU medical undergraduates under the background of COVID-19.

**Variable**		**NAEs**		**PAEs**		**NDEs**		**PDEs**	
	**Means ±SD**	***P***	**Means ±SD**	***P***	**Means ±SD**	***P***	**Means ±SD**	***P***
Gender	Male	78.01 ± 21.18	0.059	76.93 ± 13.61	0.986	52.53 ± 20.89	0.006^**^	70.32 ± 13.50	0.006^**^
	Female	75.56 ± 18.49		75.56 ± 18.49		48.97 ± 15.76		67.98 ± 11.04	
Location	Rural	77.80 ± 18.73	0.095	75.91 ± 12.25	0.060	51.71 ± 17.02	0.046^**^	67.63 ± 11.69	0.025^**^
	Urban	75.64 ± 19.85		77.47 ± 12.59		49.36 ± 18.11		69.41 ± 12.12	
Grade	Year 1	76.82 ± 20.37	0.734	77.35 ± 12.83	0.025^**^	50.33 ± 18.45	0.338	69.55 ± 12.63	0.090
	Year 2	76.32 ± 18.94		77.61 ± 12.08		49.19 ± 17.19		68.72 ± 11.30	
	Year 3	74.62 ± 18.82		74.46 ± 12.57		51.34 ± 17.05		66.62 ± 12.02	
	Year 4	77.24 ± 16.77		73.87 ± 11.23		53.35 ± 16.94		67.28 ± 10.31	
Major	Clinical medicine	76.55 ± 19.53	0.403	77.45 ± 12.36	0.152	49.68 ± 17.77	0.169	69.14 ± 11.94	0.616
	Preventive medicine	76.58 ± 18.92		75.37 ± 12.71		51.67 ± 17.60		68.02 ± 11.76	
	Nursing	79.58 ± 19.63		76.74 ± 12.48		53.76 ± 17.24		69.32 ± 11.77	
	Medical technology	73.62 ± 20.62		77.66 ± 12.60		48.10 ± 18.09		68.26 ± 13.05	
Equipment	Desktop computer	82.53 ± 22.07	0.036^**^	77.98 ± 11.65	0.905	54.91 ± 19.16	0.085	69.96 ± 11.08	0.856
	Notebook computer	75.66 ± 18.94		76.92 ± 12.56		49.41 ± 17.03		68.79 ± 11.93	
	Tablet computer	74.44 ± 21.13		77.20 ± 13.11		49.54 ± 19.42		69.09 ± 12.26	
	Mobile phone	78.41 ± 19.47		76.54 ± 12.17		51.99 ± 18.72		68.34 ± 12.32	
Online learning	<30 h	79.53 ± 19.16	0.055	74.39 ± 12.93	0.006^**^	54.37 ± 19.00	0.007^**^	66.86 ± 13.29	0.007^**^
time weekly	30–35 h	77.28 ± 18.89		76.79 ± 12.28		51.09 ± 17.58		68.35 ± 11.57	
	36–40 h	74.60 ± 18.93		77.20 ± 12.56		47.92 ± 16.69		69.27 ± 11.84	
	41–45 h	74.07 ± 17.88		76.45 ± 12.16		48.76 ± 15.62		67.98 ± 10.82	
	>46 h	77.59 ± 23.65		80.18 ± 12.19		50.01 ± 20.64		72.02 ± 11.99	

* *P* < 0.05,

** *P* < 0.01; Results were all controlled by the covariates; SD, standard deviations; NAE, negative activating emotions; PAE, positive activating emotions; NDE, negative deactivating emotions; PDE, positive deactivating emotions.

“Bold” mean that the values are statistically significant.

### Burnout and procrastination levels

Female undergraduates reported higher total burnout levels than male undergraduates, as well as within the dimensions of exhaustion and cynicism. Undergraduates from rural areas showed higher total burnout levels than urban undergraduates, as well as higher burnout in the exhaustion and professional efficacy domains. Year 1 medical undergraduates experienced less burnout in the professional efficacy domain than undergraduates in other years. Medical undergraduates who spent less time engaged in online learning experienced more burnout.

Rural undergraduates showed higher levels of procrastination. Medical undergraduates who used desktop computers procrastinated the most during the COVID-19 pandemic. The level of procrastination of participants studying for <30 h per week was significantly different from that of the other participants. Supplementary Tables S1, S2 show the results in detail.

### Relationships among burnout, procrastination and GAEs

Correlations among burnout, procrastination and GAEs are shown in Table 3. There was a significant positive correlation between procrastination and burnout among the CMU after adjusting for age, major, online learning equipment and all other covariates. Procrastination and burnout positively and negatively correlated with negative academic emotions, respectively.

**Table 3:** Means, SD and correlations of continuous variables.

**Variables**	**Means**	**SD**	**1**	**2**	**3**	**4**	**5**	**6**
1. Procrastination	42.27	12.19	1					
2. Burnout	34.63	9.52	0.708***	1				
3. NAEs	76.41	19.48	0.519***	0.573***	1			
4. PAEs	76.92	12.49	-0.566***	-0.637***	-0.249***	1		
5. NDEs	50.19	17.76	0.690***	0.778***	0.775***	-0.556***	1	
6. PDEs	68.78	11.99	-0.531***	-0.598***	-0.372***	0.836***	-0.476***	1

***P < 0.001; SD, standard deviations; Results were all controlled by the covariates; NAE, negative activating emotions; PAE, positive activating emotions; NDE, negative deactivating emotions; PDE, positive deactivating emotions.

“Bold” mean that the values are statistically significant.

The associations of burnout and its components with procrastination and GAEs are presented in Table 4. Binary logistic regression showed that burnout and its components significantly decreased with an increase of positive academic emotions and procrastination, and increased with higher levels of negative academic emotions.

**Table 4:** The relations of burnout, delay and GAEs by using binary logistic regressions.

**Variables**		**Burnout**	**Exhaustion**	**Cynicism**	**Professional efficacy**
		**OR (95%CI)**	**OR (95%CI)**	**OR (95%CI)**	**OR (95%CI)**
NAEs	Low (ref.)	1.00	1.00	1.00	1.00
	Relatively low	5.04*** (3.125, 8.131)	3.48*** (2.151, 5.643)	4.86*** (3.017, 7.838)	3.71*** (2.508, 5.476)
	Relatively high	18.86*** (11.517, 30.893)	15.66*** (9.271, 25.463)	11.03*** (6.828, 17.811)	7.13*** (4.729, 10.762)
	High	19.01*** (11.679, 30.938)	22.28*** (13.640, 36.397)	11.48*** (7.141, 11.456)	4.49*** (3.041, 6.639)
PAEs	Low (ref.)	1.00	1.00	1.00	1.00
	Relatively low	0.19*** (0.112, 0.325)	0.34*** (0.222, 0.514)	0.29*** (0.192, 0.449)	0.42*** (0.247, 0.708)
	Relatively high	0.50*** (0.030, 0.083)	0.18*** (0.122, 0.270)	0.11*** (0.073, 0.166)	0.12*** (0.075, 0.192)
	High	0.12*** (0.007, 0.022)	0.06*** (0.039, 0.096)	0.05*** (0.028, 0.073)	0.01*** (0.006, 0.020)
NDEs	Low (ref.)	1.00	1.00	1.00	1.00
	Relatively low	8.94*** (4.423, 18.055)	3.66*** (2.108, 6.353)	6.77*** (3.504, 13.095)	6.28*** (4.058, 9.707)
	Relatively high	58.95*** (29.251, 118.810)	20.50*** (11.991,35.037)	25.23*** (13.262,48.015)	13.04*** (8.338, 20.380)
	High	218.35*** (101.984, 467.506)	83.33*** (45.752, 151.787)	104.62*** (53.028, 206.415)	15.82*** (10.025, 24.965)
PDEs	Low (ref.)	1.00	1.00	1.00	1.00
	Relatively low	0.23*** (0.143, 0.384)	0.50*** (0.336, 0.750)	0.35*** (0.236, 0.521)	0.41*** (0.239, 0.703)
	Relatively high	0.06*** (0.037, 0.100)	0.21*** (0.137, 0.311)	0.17*** (0.112, 0.255)	0.08*** (0.050, 0.141)
	High	0.02*** (0.010, 0.031)	0.02*** (0.046, 0.114)	0.08*** (0.050, 0.124)	0.01*** (0.004, 0.016)
Procrastination	Low (ref.)	1.00	1.00	1.00	1.00
	Relatively low	5.22*** (2.998, 9.103)	3.47*** (2.120, 5.658)	4.09*** (2.297, 7.264)	5.23*** (3.383, 8.075)
	Relatively high	21.47*** (12.468, 36.964)	9.86*** (6.148, 15.809)	14.53*** (8.392, 25.159)	12.58*** (8.097, 19.558)
	High	85.11*** (46.283,156.523)	44.56*** (26.112, 76.038)	60.87*** (33.660, 110.093)	15.18*** (9.639, 23.911)

***P < 0.001; Results were all controlled by the covariates; SD, standard deviations; GAEs, general academic emotions; NAEs, negative activating emotions; PAEs, positive activating emotions; NDEs, negative deactivating emotions; PDEs, positive deactivating emotions.

### Mediating roles of GAEs

Table 5 shows the mediating effects of GAEs. Procrastination had positive associations with NAEs and NDEs, and negative associations with PAEs and PDEs (path **a**). NAEs and NDEs positively correlated with burnout, while PAEs and PDEs showed the opposite correlation (path **b**). When procrastination and GAEs were simultaneously entered into the regression model, NAEs (95% CI: 0.092–0.142), PAEs (95% CI: 0.121–0.195), NDEs (95% CI: 0.261–0.342) and PDEs (95% CI: 0.095–0.166) mediated the direct effects (0.553–0.436, 0.394, 0.300 and 0.131 respectively, after adjusting for all covariates (path **c'**). The contributions (as mediators) of GAEs to burnout and procrastination (path **c**) were 21.16% (NAEs), 29.75% (PAEs), 54.25% (NDEs) and 23.69% (PDEs).

**Table 5:** The mediating role of GAEs on the associations between procrastination and burnout.

**Mediators**	**c**	**a**	**b**	**c'**	Mediation (a*b)	**95%CI**
NAEs	0.553***	0.848***	0.138***	0.436***	0.117*	0.092–0.142
PAEs	0.553***	−0.594***	−0.268***	0.394***	0.159*	0.121–0.195
NDEs	0.553***	1.012***	0.297***	0.253***	0.300*	0.261–0.342
PDEs	0.553***	−0.247***	−0.532***	0.422***	0.131*	0.095–0.166

*P < 0.05, ***P < 0.001; Results were all controlled by the covariates; SD, standard deviations; GAEs, general academic emotions; NAEs, negative activating emotions; PAEs, positive activating emotions; NDEs, negative deactivating emotions; PDEs, positive deactivating emotions. a, associations of procrastination with GAEs; b, associations of GAEs with burnout; c', association between procrastination and burnout after adding GAEs as mediators; 95%CI were calculated by bootstrap method.

## Discussion

In today's unprecedented COVID-19 pandemic era, quarantine measures, which have been used effectively for centuries to slow the transmission of infection, have been implemented worldwide. Due to the high concentration of students and frequent social activities on campus, universities around the world have closed campuses and implemented online curricula and digital learning (54). Lockdown, isolation and social distancing effectively controlled the epidemic, but have had a detrimental impact on students' mental health (especially on medical undergraduates whose major emphasizes practice) (55). In the present study, we first discussed the positive correlation between procrastination and burnout and demonstrated mediating effects of GAEs. The mediating effect of NDEs was the most significant among all GAEs; due to the significant change in the learning environment, medical undergraduates' procrastination led to more serious burnout in association with major uncertainties and anxiety. In turn, this may undermine academic performance and psychological health. We also found that gender, location, online learning duration and equipment preferences, and academic year were associated with mental health and GAEs.

The prevalence of burnout differs by gender among medical professions (56). Female medical professionals suggested suffer more from burnout, due to discriminative behavior from patients, occupational biases or gendered macro-aggressions (57–59). However, an investigation assessing the frequency of psychological distress among physician residents showed that, whereas female residents were more likely to suffer from anxiety and depression, male residents were more vulnerable to burnout (60). It seems that, during training and earlier career stages, male medical undergraduates may suffer more from burnout, which were similar to the result of present study. The question is, why female medical professionals suffer more from burnout later in their careers? Whether burnout among female medical professionals should be labeled as a “workplace” or “occupational” characteristic also merits further study (61, 62). Regarding the influence of switching to the online learning environment, we found that rural medical undergraduates reported higher burnout and procrastination levels. This might be related to online learning equipment proficiency and quality, barriers to accessing learning resources, and interference with learning by anxiety regarding peer competition. Furthermore, medical undergraduates who spent <30 h studying online per week had higher levels of burnout and procrastination. Medical undergraduates who devoted less time to learning online were more likely to have psychological problems related to a lack of self-control, uncertainty regarding learning goals and anxiety about quarantine. In addition, desktop learning appeared to cause the highest level of procrastination, such that medical undergraduates preferred using mobile devices to study during the pandemic.

Lockdown, quarantine measures and social distancing have had detrimental effects on the mental health of medical undergraduates, leading to dramatically increased levels of depression, anxiety and stress (63, 64). We also found positive correlations of burnout with procrastination and negative learning-related emotions. Moreover, NDEs showed the highest correlations with burnout and procrastination among all academic emotions, indicating that the medical undergraduates felt confusion and helplessness when trying to learn during the pandemic. In terms of deactivating emotions, female undergraduates and those from rural areas felt more helpless and scared than urban undergraduates. Pekrun pointed out that academic emotions encompass all emotional experiences that a person may experience during the life course (44). Medical undergraduates are already under high academic and social pressure, which can cause procrastination (32). Major events, such as the COVID-19 pandemic, might trigger learning anxiety, boredom in association with homework, loss of interest in learning or no expectation of success in examinations, or even professional identity and professionalism among medical undergraduates.

We studied the mediating role of academic emotions and confirmed our hypothesis, i.e., that academic emotions suggested mediating effects with respect to procrastination and anxiety. PAEs and PDEs reduced the correlation between procrastination and burnout by 28.75 and 23.69% respectively. Thus, the mediating role of PAEs was greater than that of PDEs. NDEs explained 54.25% of the mediating effect, which was not only higher than that explained by NAEs (21.16%), but also higher than all other GAEs. This indicated that medical undergraduates' worries about uncertainties of the learning environment, including pessimism about their academic prospects and low interest in learning, were most prominent when engaged in distance learning in the context of the COVID-19 pandemic. According to Pekrun (44), NDEs reflect undergraduates' uncertainty about outcomes, loss of control, and feelings of powerlessness regarding the learning process associated with increased telecommunication, perceptual barriers, and a lack of self-regulation or external regulation of learning (46, 50). In addition to further verifying Pekrun's theory, we also found that, as negative emotions heightened, the overall risk of burnout increased. Thus, without timely intervention, negative academic emotions might exacerbate academic burnout.

There were several limitations to the present study. The participants were all from CMU, which might reduce the representativeness and generalizability of our results. Also, the results may have been affected by recall bias and survey-driven self-selection bias. Other potential factors, such as high homework loads, challenging exams, lack of role models may also contribute to medical undergraduates' stress and procrastination, that were worthy of further discussion. Analysis of pre-epidemic data would have enhanced the usefulness of our study, along with follow-up. Balancing pandemic prevention measures with the protection of medical undergraduates' physical and mental health is an urgent issue. The present study provides empirical evidence regarding how to identify targets for, and formulate, intervention strategies for Chinese medical undergraduates while simultaneously preventing the spread of COVID-19.

## Conclusions

In summary, the current results highlighted the correlation between burnout and procrastination with the mediating role of general academic emotions among medical undergraduates. In the context of COVID-19, this study profoundly identified the emotional maladjustment and confusion of medical undergraduates in response to changes in their learning environment. Our findings provide a practical basis for further accurate optimization of online teaching environment, improvement of teaching evaluation methods, promotion of medical undergraduates' anxiety, stress and depression management, in terms of improving mental health of medical undergraduates.

## Data availability statement

The datasets presented in this study can be found in online repositories. The names of the repository/repositories and accession number (s) can be found in the article/Supplementary material.

## Ethics statement

The studies involving human participants were reviewed and approved by the Human Research Ethics Committee of China Medical University. The patients/participants provided their written informed consent to participate in this study.

## Author contributions

ND and DW substantially contributed to the conception and design of the research. ZC contributed to recruit volunteers and RC helped with the data acquisition. RQ analyzed the data, interpreted the results, and prepared the initial draft of the manuscript. The double check with the dataset was carried out by XS. ND, HL, and YZ critically reviewed the manuscript and gave advice for modifications. HL, ND, and DW worked for the final approval of the version of the manuscript to be published. All authors contributed to the article and approved this submitted version.

## Funding

This study was supported by the First batch of the 14th Five-Year Medical Education Scientific Research Project of China Medical University (YDJK20211051) and China Postdoctoral Science Foundation (2021MD703900).

## Conflict of interest

The authors declare that the research was conducted in the absence of any commercial or financial relationships that could be construed as a potential conflict of interest.

## Publisher's note

All claims expressed in this article are solely those of the authors and do not necessarily represent those of their affiliated organizations, or those of the publisher, the editors and the reviewers. Any product that may be evaluated in this article, or claim that may be made by its manufacturer, is not guaranteed or endorsed by the publisher.

## Abbreviations

CI: confidence interval
CMU: China Medical University
GAEs: general academic emotions
NAEs: negative activating emotions
NDEs: negative deactivating emotions
OR: odds ratio
PAEs: positive activating emotions
PDEs: positive deactivating emotions
SD: standard deviation.
