# Supplementary material for: The mediating role of general academic emotions in burnout and procrastination among Chinese medical undergraduates during the COVID-19 pandemic: A cross-sectional study
Source: Front Public Health. 2022 Dec 5;10:1011801. doi: 10.3389/fpubh.2022.1011801 (PMC9760956; doi:10.3389/fpubh.2022.1011801)
Supplement: Supplementary file 1 [file Table_1.DOCX]

| SP Table1.Burnout of CMU medical undergraduates under the background of COVID-19 | | | | | | | | | |
| --- | --- | --- | --- | --- | --- | --- | --- | --- | --- |
| **Variable** | | **Total burnout** | | **Exhaustion** | | **Cynicism** | | **Professional efficacy** | |
|  |  | **Means ± SD** | **P** | **Means ± SD** | **P** | **Means ± SD** | **P** | **Means ± SD** | **P** |
| Gender | Male | 36.11 ± 10.54 | **0.001^**^** | 9.97 ± 4.10 | **< 0.001^***^** | 9.69 ± 5.02 | **< 0.001^**^** | 16.46 ± 4.12 | 0.188 |
|  | Female | 33.85 ± 8.85 |  | 8.94 ± 3.48 |  | 8.11 ± 3.66 |  | 16.80 ± 3.57 |  |
|  |  |  |  |  |  |  |  |  |  |
| Location | Rural | 35.84 ± 9.54 | **0.003^**^** | 9.93 ± 3.77 | **< 0.001^***^** | 8.88 ± 4.31 | 0.211 | 17.03 ± 3.77 | **0.034^*^** |
|  | Urban | 33.96 ± 9.45 |  | 8.94 ± 3.67 |  | 8.53 ± 4.21 |  | 16.50 ± 3.76 |  |
|  |  |  |  |  |  |  |  |  |  |
| Grade | Year1 | 34.33 ± 9.60 | 0.055 | 9.18 ± 3.78 | 0.216 | 8.75 ± 4.38 | 0.363 | 16.41 ± 3.77 | **0.005^**^** |
|  | Year2 | 34.16 ± 9.39 |  | 9.19 ± 3.71 |  | 8.36 ± 4.03 |  | 16.61 ± 3.78 |  |
|  | Year3 | 36.24 ± 9.46 |  | 9.75 ± 3.67 |  | 9.01 ± 4.31 |  | 17.49 ± 3.69 |  |
|  | Year4 | 36.85 ± 9.45 |  | 10.02 ± 3.63 |  | 9.02 ± 4.36 |  | 17.81 ± 3.53 |  |
|  |  |  |  |  |  |  |  |  |  |
| Major | Clinical medicine | 34.33 ± 9.60 | 0.312 | 9.25 ± 3.74 | 0.219 | 8.57 ± 4.19 | 0.606 | 16.51 ± 3.77 | 0.312 |
|  | Preventive medicine | 34.16 ± 9.39 |  | 9.59 ± 3.67 |  | 8.81 ± 4.12 |  | 17.03 ± 3.77 |  |
|  | Nursing | 36.24 ± 9.46 |  | 9.58 ± 4.00 |  | 9.37 ± 4.92 |  | 16.95 ± 3.72 |  |
|  | Medical technology | 36.85 ± 9.45 |  | 8.67 ± 3.74 |  | 8.46 ± 4.67 |  | 16.77 ± 3.80 |  |
|  |  |  |  |  |  |  |  |  |  |
| Equipment | Desktop computer | 36.09 ± 10.54 | 0.641 | 9.87 ± 4.23 | 0.606 | 9.67 ± 5.36 | 0.322 | 16.56 ± 4.14 | 0.944 |
|  | Notebook computer | 34.45 ± 9.42 |  | 9.20 ± 3.67 |  | 8.53 ± 3.96 |  | 16.72 ± 3.73 |  |
|  | Tablet computer | 24.35 ± 9.59 |  | 9.27 ± 3.93 |  | 8.60 ± 4.45 |  | 16.48 ± 3.78 |  |
|  | Mobile phone | 35.00 ± 9.60 |  | 9.46 ± 3.75 |  | 8.84 ± 4.72 |  | 16.71 ± 3.84 |  |
|  |  |  |  |  |  |  |  |  |  |
| Online learning time weekly | <30h | 37.25 ± 10.90 | **0.004^**^** | 10.06 ± 4.06 | **0.003^**^** | 9.73 ± 5.18 | **0.007^**^** | 17.45 ± 4.24 | **0.006^**^** |
|  | 30-35h | 34.69 ± 9.19 |  | 9.24 ± 3.58 |  | 8.77 ± 4.18 |  | 16.67 ± 3.63 |  |
|  | 36-40h | 33.45 ± 9.24 |  | 8.68 ± 3.53 |  | 8.15 ± 3.74 |  | 16.62 ± 3.71 |  |
|  | 41-45h | 34.65 ± 8.70 |  | 9.37 ± 3.46 |  | 8.40 ± 3.86 |  | 16.88 ± 3.54 |  |
|  | >46h | 34.04 ± 9.77 |  | 9.82 ± 4.28 |  | 8.52 ± 4.54 |  | 15.70 ± 3.82 |  |

| SP Table2. Delay of CMU medical undergraduates under the background of COVID-19 | | | |
| --- | --- | --- | --- |
| **Variable** | | **Procrastination** | |
|  |  | **Means ± SD** | **P** |
| Gender | Male | 43.11 ± 12.79 | 0.127 |
|  | Female | 41.83 ± 11.85 |  |
|  |  |  |  |
| Location | Rural | 43.53 ± 12.34 | **0.016^*^** |
|  | Urban | 41.58 ± 12.05 |  |
|  |  |  |  |
| Grade | Year1 | 42.25 ± 12.42 | 0.351 |
|  | Year2 | 42.02 ± 12.38 |  |
|  | Year3 | 41.83 ± 10.52 |  |
|  | Year4 | 45.13 ± 12.16 |  |
|  |  |  |  |
| Major | Clinical medicine | 42.17 ± 12.41 | 0.538 |
|  | Preventive medicine | 43.03 ± 11.54 |  |
|  | Nursing | 40.34 ± 11.05 |  |
|  | Medical technology | 41.68 ± 12.91 |  |
|  |  |  |  |
| Equipment | Desktop computer | 46.84 ± 10.77 | **0.011^*^** |
|  | Notebook computer | 41.76 ± 12.27 |  |
|  | Tablet computer | 40.90 ± 12.32 |  |
|  | Mobile phone | 43.59 ± 11.89 |  |
|  |  |  |  |
| Online learning time weekly | <30h | 46.99 ± 13.56 | **<0.001^***^** |
|  | 30-35h | 42.25 ± 11.56 |  |
|  | 36-40h | 40.78 ± 11.89 |  |
|  | 41-45h | 41.84 ± 11.79 |  |
|  | >46h | 40.74 ± 12.00 |  |
