# Supplementary material for: The mediating role of general academic emotions in burnout and procrastination among Chinese medical undergraduates during the COVID-19 pandemic: A cross-sectional study
Source: Front Public Health. 2022 Dec 5;10:1011801. doi: 10.3389/fpubh.2022.1011801 (PMC9760956; doi:10.3389/fpubh.2022.1011801)
Supplement: Supplementary file 2 [file Data_Sheet_1.pdf]

序号(No.)	学号(CODE)	性别(sex)	年龄(age)	户口 (location)	年级 (grades)	专业 (major)	设备 (learning equipment)
1	2016340305	1	23	1	4	1	2
2	2016352414	2	22	1	4	1	4
3	2016352702	2	23	1	4	2	4
4	2016352703	1	22	1	4	2	3
5	2016352704	2	22	2	4	2	3
6	2016352705	1	22	1	4	2	2
7	2016352706	2	23	1	4	2	2
8	2016352707	1	22	2	4	2	4
9	2016352708	2	22	2	4	2	2
10	2016352709	1	22	1	4	2	2
11	2016352710	2	22	1	4	2	4
12	2016352711	1	23	2	4	2	2
13	2016352712	2	22	2	4	2	1
14	2016352713	1	22	1	4	2	4
15	2016352714	2	22	2	4	2	2
16	2016352716	2	21	1	4	2	3
17	2016352717	1	22	2	4	2	3
18	2016352719	1	23	1	4	2	4
19	2016352720	2	22	1	4	2	3
20	2016352721	1	22	1	4	2	2
21	2016352722	2	22	2	4	2	2
22	2016352723	2	22	2	4	2	2
23	2016352724	2	22	1	4	2	4
24	2016352725	2	21	2	4	2	4
25	2016352726	2	21	2	4	2	2
26	2016352727	2	20	1	4	2	2
27	2016352728	2	22	2	4	2	2
28	2016352729	2	22	1	4	2	4
29	2016352730	2	22	1	4	2	3
30	2016352802	2	22	1	4	2	4
31	2016352805	1	22	1	4	2	2
32	2016352808	2	23	2	4	2	2
33	2016352809	1	21	2	4	2	2
34	2016352814	2	23	2	4	2	4
35	2016352815	1	22	2	4	2	4
36	2016352816	2	22	2	4	2	4
37	2016352817	1	21	1	4	2	3
38	2016352825	2	22	1	4	2	3
39	2016352830	2	22	2	4	2	2
40	2016352906	2	23	1	4	2	2
41	2016352911	1	22	1	4	2	2
42	2016352913	1	22	2	4	2	2
43	2016352917	1	21	2	4	2	3
44	2016352919	1	22	1	4	2	3
45	2016352920	2	22	2	4	2	4
46	2016352921	2	22	2	4	2	3
47	2016352922	2	24	2	4	2	3
48	2016352923	2	22	2	4	2	4
49	2016352925	2	22	2	4	2	2
50	2016352928	2	23	1	4	2	4

51	2016352929	2	23	2	4	2	2
52	2016362715	1	22	2	4	2	4
53	2016364625	1	22	2	4	1	2
54	2017328514	2	20	2	3	1	2
55	2017328520	2	21	1	3	1	2
56	2017328604	2	21	1	3	1	4
57	2017328622	2	22	2	3	1	1
58	2017328810	2	21	1	3	1	3
59	2017340529	1	21	2	3	1	4
60	2017340716	2	21	2	3	1	2
61	2017340932	2	21	2	3	1	2
62	2017341119	1	21	2	3	1	2
63	2017352017	1	21	2	3	1	2
64	2017352028	2	21	2	3	1	2
65	2017352119	1	21	2	3	1	2
66	2017352304	2	21	1	3	1	2
67	2017352306	2	20	2	3	1	2
68	2017352307	1	21	1	3	1	2
69	2017352309	1	22	2	3	1	2
70	2017352310	2	21	2	3	1	2
71	2017352311	1	20	2	3	1	3
72	2017352312	2	20	2	3	1	2
73	2017352324	2	20	1	3	1	2
74	2017352326	2	21	2	3	1	2
75	2017352327	2	21	2	3	1	3
76	2017352328	2	22	1	3	1	3
77	2017352404	2	21	2	3	1	2
78	2017352406	2	21	2	3	1	2
79	2017352412	2	20	1	3	1	4
80	2017352413	1	20	2	3	1	2
81	2017352420	2	21	1	3	1	2
82	2017352421	2	20	1	3	1	4
83	2017352422	2	21	2	3	1	4
84	2017352424	2	21	1	3	1	4
85	2017352510	2	21	2	3	1	3
86	2017352513	1	22	2	3	1	3
87	2017352520	2	20	2	3	1	2
88	2017352524	2	21	1	3	1	3
89	2017352803	1	22	1	3	2	2
90	2017352804	2	22	1	3	2	2
91	2017352808	2	22	2	3	2	2
92	2017352809	1	23	1	3	2	2
93	2017352811	1	22	1	3	2	2
94	2017352812	2	20	2	3	2	3
95	2017352814	2	21	2	3	2	4
96	2017352816	2	20	2	3	2	4
97	2017352817	1	20	1	3	2	2
98	2017352818	2	21	2	3	2	3
99	2017352820	2	22	1	3	2	3
100	2017352821	1	21	1	3	2	2
101	2017352822	2	23	2	3	2	2
102	2017352823	1	21	2	3	2	2
103	2017352824	2	21	2	3	2	3
104	2017352825	1	22	2	3	2	2
105	2017352826	2	20	2	3	2	4
106	2017352830	2	22	2	3	2	2

107	2017352901	1	21	1	3	2	4
108	2017352902	2	20	2	3	2	2
109	2017352902	1	21	2	3	2	2
110	2017352903	1	22	2	3	2	2
111	2017352904	2	21	2	3	2	2
112	2017352906	2	20	2	3	2	2
113	2017352907	1	21	2	3	2	2
114	2017352908	2	20	2	3	2	2
115	2017352909	1	22	1	3	2	2
116	2017352910	2	22	2	3	2	2
117	2017352911	1	23	1	3	2	3
118	2017352912	2	22	2	3	2	2
119	2017352913	1	21	1	3	2	2
120	2017352914	2	21	2	3	2	2
121	2017352915	1	21	2	3	2	3
122	2017352916	2	21	2	3	2	2
123	2017352917	1	21	2	3	2	3
124	2017352918	2	22	1	3	2	2
125	2017352920	2	21	2	3	2	2
126	2017352921	1	21	2	3	2	2
127	2017352922	2	22	1	3	2	3
128	2017352923	1	21	2	3	2	1
129	2017352924	2	22	1	3	2	4
130	2017352926	2	22	1	3	2	2
131	2017352927	1	21	2	3	2	1
132	2017352928	2	22	1	3	2	2
133	2017352929	2	21	2	3	2	3
134	2017353002	2	22	1	3	2	2
135	2017353003	1	21	1	3	2	3
136	2017353004	2	22	1	3	2	4
137	2017353005	1	22	1	3	2	3
138	2017353007	1	22	1	3	2	4
139	2017353008	2	21	1	3	2	4
140	2017353010	2	22	2	3	2	2
141	2017353011	1	21	1	3	2	2
142	2017353012	2	20	1	3	2	2
143	2017353014	2	21	2	3	2	2
144	2017353015	1	21	2	3	2	2
145	2017353016	2	21	1	3	2	4
146	2017353017	1	22	1	3	2	4
147	2017353018	2	21	2	3	2	3
148	2017353019	1	24	1	3	2	2
149	2017353020	2	21	1	3	2	4
150	2017353021	1	19	2	3	2	2
151	2017353022	2	21	2	3	2	2
152	2017353023	1	22	2	3	2	2
153	2017353024	2	21	2	3	2	2
154	2017353025	1	21	2	3	2	2
155	2017353026	2	21	2	3	2	2
156	2017353027	1	23	1	3	2	4
157	2017353028	2	21	2	3	2	2
158	2017353029	2	20	2	3	2	4
159	2017353030	2	20	2	3	2	3
160	2017353930	2	21	2	3	2	2
161	2017364113	1	21	1	3	4	4
162	2017364217	2	20	2	3	3	2

163	2017364512	2	21	2	3	3	4
164	2017364523	2	21	2	3	3	2
165	2017364607	2	21	2	3	3	3
166	2017364625	2	21	1	3	3	4
167	2017364721	2	20	1	3	3	2
168	2018327816	2	20	2	2	1	2
169	2018328502	2	19	2	2	1	2
170	2018328504	2	20	2	2	1	2
171	2018328510	2	20	1	2	1	2
172	2018328513	1	20	1	2	1	2
173	2018328516	2	19	2	2	1	2
174	2018328520	2	20	2	2	1	4
175	2018328523	1	20	2	2	1	2
176	2018328609	1	20	1	2	1	4
177	2018328625	1	21	2	2	1	2
178	2018328706	2	22	1	2	1	4
179	2018328707	1	20	2	2	1	4
180	2018328708	2	20	2	2	1	2
181	2018328710	2	20	2	2	1	3
182	2018328714	2	21	2	2	1	2
183	2018328717	1	20	2	2	1	2
184	2018328720	2	21	2	2	1	1
185	2018328722	2	20	1	2	1	2
186	2018328724	2	20	2	2	1	2
187	2018328725	1	20	2	2	1	2
188	2018328808	2	20	1	2	1	2
189	2018328811	1	20	2	2	1	2
190	2018328816	2	20	1	2	1	2
191	2018328822	2	20	2	2	1	4
192	2018328823	1	20	2	2	1	2
193	2018328825	1	21	2	2	1	2
194	2018340119	1	20	2	2	1	2
195	2018340203	1	20	1	2	1	2
196	2018340205	1	20	2	2	1	2
197	2018340206	2	21	1	2	1	2
198	2018340209	1	20	2	2	1	2
199	2018340210	2	20	2	2	1	2
200	2018340212	2	19	2	2	1	2
201	2018340213	1	20	2	2	1	2
202	2018340214	2	20	2	2	1	4
203	2018340215	1	21	2	2	1	2
204	2018340216	2	20	2	2	1	2
205	2018340219	1	20	2	2	1	2
206	2018340220	2	20	1	2	1	2
207	2018340221	1	20	2	2	1	2
208	2018340222	2	20	2	2	1	2
209	2018340223	1	19	2	2	1	2
210	2018340224	2	21	1	2	1	4
211	2018340226	2	19	2	2	1	2
212	2018340302	2	20	2	2	1	3
213	2018340304	2	20	2	2	1	2
214	2018340305	1	18	1	2	1	2
215	2018340316	2	21	2	2	1	2
216	2018340322	2	20	2	2	1	2
217	2018340404	2	21	2	2	1	2
218	2018340408	2	19	2	2	1	4

219	2018340416	2	20	2	2	1	4
220	2018340420	2	19	1	2	1	2
221	2018340425	2	21	2	2	1	3
222	2018340426	2	19	2	2	1	2
223	2018340504	2	21	2	2	1	4
224	2018340505	1	20	2	2	1	2
225	2018340506	2	20	1	2	1	3
226	2018340514	2	20	2	2	1	4
227	2018340516	2	20	2	2	1	4
228	2018340523	1	20	1	2	1	4
229	2018340524	2	20	1	2	1	2
230	2018340602	2	21	1	2	1	2
231	2018340607	1	20	1	2	1	2
232	2018340614	2	19	2	2	1	2
233	2018340616	2	20	2	2	1	2
234	2018340618	2	20	2	2	1	4
235	2018340619	1	19	1	2	1	2
236	2018340620	2	20	2	2	1	2
237	2018340623	1	21	1	2	1	2
238	2018340624	2	20	2	2	1	3
239	2018340704	2	20	2	2	1	3
240	2018340712	2	20	2	2	1	3
241	2018340714	2	20	2	2	1	1
242	2018340718	2	20	2	2	1	2
243	2018340720	2	20	2	2	1	2
244	2018340722	2	18	2	2	1	3
245	2018340724	2	19	2	2	1	2
246	2018340725	1	19	2	2	1	2
247	2018340801	1	20	2	2	1	2
248	2018340802	2	20	2	2	1	2
249	2018340804	2	20	2	2	1	2
250	2018340806	2	20	2	2	1	2
251	2018340807	1	20	2	2	1	4
252	2018340808	2	20	2	2	1	2
253	2018340810	2	20	2	2	1	3
254	2018340811	1	21	1	2	1	2
255	2018340812	2	20	2	2	1	3
256	2018340818	2	19	2	2	1	3
257	2018340825	2	21	2	2	1	2
258	2018340826	2	20	2	2	1	4
259	2018340916	2	20	2	2	1	3
260	2018340919	1	19	2	2	1	1
261	2018340920	2	21	1	2	1	2
262	2018340923	1	20	2	2	1	2
263	2018341501	1	20	2	2	1	2
264	2018341502	2	19	2	2	1	2
265	2018341504	2	20	1	2	1	4
266	2018341506	2	20	2	2	1	2
267	2018341508	2	20	2	2	1	2
268	2018341509	1	20	1	2	1	2
269	2018341510	2	19	1	2	1	2
270	2018341511	1	20	1	2	1	2
271	2018341514	2	20	1	2	1	2
272	2018341516	2	19	1	2	1	2
273	2018341519	1	20	2	2	1	2
274	2018341522	2	20	1	2	1	2

275	2018341523	1	20	2	2	1	4
276	2018341524	2	20	2	2	1	2
277	2018341525	2	21	1	2	1	2
278	2018341526	2	20	2	2	1	2
279	2018341606	2	21	1	2	1	3
280	2018341701	1	20	2	2	1	2
281	2018341702	2	19	1	2	1	2
282	2018341703	1	20	1	2	1	3
283	2018341704	2	21	2	2	1	2
284	2018341705	1	20	2	2	1	2
285	2018341707	1	20	1	2	1	2
286	2018341708	2	20	2	2	1	2
287	2018341709	1	21	1	2	1	4
288	2018341710	2	20	2	2	1	1
289	2018341711	1	20	2	2	1	1
290	2018341713	1	21	2	2	1	4
291	2018341714	2	21	1	2	1	2
292	2018341715	1	20	2	2	1	1
293	2018341716	2	20	2	2	1	2
294	2018341717	1	22	2	2	1	2
295	2018341718	2	20	1	2	1	2
296	2018341719	1	20	2	2	1	3
297	2018341720	2	19	2	2	1	2
298	2018341721	1	20	2	2	1	2
299	2018341722	2	20	1	2	1	2
300	2018341724	2	20	1	2	1	2
301	2018341725	2	20	2	2	1	2
302	2018341726	2	19	2	2	1	3
303	2018341801	1	21	2	2	1	2
304	2018341804	2	20	2	2	1	2
305	2018341805	1	21	1	2	1	2
306	2018341811	1	20	2	2	1	2
307	2018341812	2	20	2	2	1	2
308	2018341813	1	20	2	2	1	2
309	2018341825	2	22	1	2	1	2
310	2018341901	1	20	2	2	1	2
311	2018341903	1	19	2	2	1	2
312	2018341904	2	20	1	2	1	4
313	2018341906	2	21	2	2	1	2
314	2018341906	2	21	2	2	1	2
315	2018341907	1	20	2	2	1	2
316	2018341911	1	20	2	2	1	2
317	2018341912	2	20	2	2	1	2
318	2018341913	1	20	2	2	1	2
319	2018341914	2	22	1	2	1	2
320	2018341916	2	20	2	2	1	1
321	2018341919	1	20	2	2	1	2
322	2018341921	1	20	2	2	1	2
323	2018341922	2	22	1	2	1	3
324	2018341923	1	20	2	2	1	4
325	2018342004	2	22	1	2	1	4
326	2018342005	1	20	2	2	1	2
327	2018342006	2	20	2	2	1	4
328	2018342007	1	20	2	2	1	1
329	2018342009	1	19	1	2	1	2
330	2018342010	2	21	2	2	1	2

331	2018342013	1	19	1	2	1	2
332	2018342014	2	20	1	2	1	4
333	2018342018	2	19	2	2	1	2
334	2018342019	1	20	2	2	1	2
335	2018342020	2	20	2	2	1	2
336	2018342022	2	21	2	2	1	2
337	2018342024	2	20	2	2	1	2
338	2018342025	2	20	2	2	1	4
339	2018342026	2	20	1	2	1	2
340	2018342101	1	20	2	2	1	2
341	2018342102	2	20	2	2	1	2
342	2018342103	1	20	2	2	1	2
343	2018342104	2	20	2	2	1	1
344	2018342105	1	21	1	2	1	2
345	2018342107	1	20	2	2	1	4
346	2018342108	2	20	2	2	1	2
347	2018342109	1	20	2	2	1	1
348	2018342110	2	20	1	2	1	2
349	2018342112	2	20	2	2	1	4
350	2018342115	1	21	2	2	1	2
351	2018342116	2	20	2	2	1	2
352	2018342119	1	21	1	2	1	2
353	2018342122	2	21	2	2	1	4
354	2018342123	2	20	2	2	1	2
355	2018342124	2	20	1	2	1	4
356	2018352204	2	20	1	2	1	4
357	2018352210	2	20	1	2	1	4
358	2018352219	1	20	2	2	1	2
359	2018352316	2	20	2	2	1	3
360	2018352326	2	20	1	2	1	2
361	2018352415	1	20	1	2	1	3
362	2018352417	1	19	1	2	1	2
363	2018352419	2	20	2	2	1	3
364	2018352422	2	18	1	2	1	2
365	2018352426	2	20	2	2	1	3
366	2018352428	2	20	2	2	1	2
367	2018352501	1	20	2	2	1	2
368	2018352502	2	20	2	2	1	4
369	2018352504	2	20	1	2	1	2
370	2018352507	1	21	1	2	1	4
371	2018352512	2	20	2	2	1	2
372	2018352521	2	20	1	2	1	2
373	2018352523	2	20	2	2	1	2
374	2018352527	2	20	2	2	1	2
375	2018352528	2	21	2	2	1	4
376	2018352607	1	20	2	2	1	2
377	2018352611	1	20	2	2	1	1
378	2018352704	2	20	1	2	1	4
379	2018352705	1	20	1	2	1	3
380	2018352706	2	21	1	2	1	2
381	2018352708	2	19	2	2	1	4
382	2018352710	2	19	2	2	1	2
383	2018352711	1	20	2	2	1	2
384	2018352712	2	20	1	2	1	2
385	2018352727	1	22	2	2	1	2
386	2018352728	1	22	2	2	1	4

387	2018352729	1	20	2	2	1	2
388	2018352806	2	22	1	2	1	2
389	2018352807	1	19	2	2	1	4
390	2018352810	2	19	1	2	1	3
391	2018352908	2	21	1	2	1	4
392	2018352914	2	20	2	2	1	2
393	2018352916	2	20	2	2	1	2
394	2018352927	2	20	2	2	1	2
395	2018353201	1	20	2	2	2	3
396	2018353202	2	20	2	2	2	2
397	2018353203	1	20	2	2	2	4
398	2018353204	2	20	2	2	2	2
399	2018353205	1	20	2	2	2	2
400	2018353206	2	20	2	2	2	3
401	2018353207	1	20	1	2	2	2
402	2018353208	2	20	2	2	2	2
403	2018353209	1	23	1	2	2	4
404	2018353210	2	20	2	2	2	3
405	2018353211	1	20	2	2	2	4
406	2018353213	1	20	2	2	2	4
407	2018353214	2	20	2	2	1	2
408	2018353216	2	19	1	2	2	2
409	2018353217	1	20	1	2	2	3
410	2018353218	2	20	2	2	2	4
411	2018353219	2	20	1	2	2	4
412	2018353220	2	20	2	2	2	4
413	2018353222	2	21	2	2	2	2
414	2018353223	2	20	2	2	2	2
415	2018353224	2	20	2	2	2	2
416	2018353225	2	19	1	2	2	2
417	2018353226	2	21	2	2	2	4
418	2018353227	2	20	2	2	2	2
419	2018353228	2	19	1	2	2	2
420	2018353229	2	20	2	2	2	2
421	2018353302	2	21	1	2	2	4
422	2018353303	1	20	1	2	2	4
423	2018353304	2	22	1	2	2	2
424	2018353305	1	19	2	2	2	4
425	2018353306	2	20	2	2	2	2
426	2018353307	1	21	2	2	2	1
427	2018353308	2	20	2	2	2	1
428	2018353309	1	19	1	2	2	2
429	2018353309	1	19	1	2	2	2
430	2018353310	2	20	2	2	2	4
431	2018353311	1	21	2	2	2	4
432	2018353312	2	20	1	2	2	2
433	2018353313	1	20	2	2	2	2
434	2018353316	2	20	1	2	2	3
435	2018353317	1	20	1	2	2	2
436	2018353318	2	19	2	2	2	3
437	2018353320	2	19	1	2	2	2
438	2018353322	2	20	1	2	2	2
439	2018353323	2	21	1	2	2	3
440	2018353324	2	20	1	2	2	2
441	2018353325	2	20	2	2	2	2
442	2018353327	2	20	2	2	2	3

443	2018353328	2	19	2	2	2	2
444	2018353330	2	20	2	2	2	2
445	2018353401	1	20	1	2	2	2
446	2018353405	1	21	2	2	2	3
447	2018353407	1	21	1	2	2	2
448	2018353408	2	20	2	2	2	2
449	2018353409	1	21	2	2	2	2
450	2018353411	1	21	1	2	2	2
451	2018353413	1	20	2	2	2	2
452	2018353414	2	20	2	2	2	3
453	2018353415	1	20	2	2	2	2
454	2018353418	2	20	2	2	1	4
455	2018353420	2	20	2	2	2	2
456	2018353421	2	20	1	2	2	2
457	2018353423	2	20	2	2	2	2
458	2018353424	2	20	2	2	2	1
459	2018353426	2	20	2	2	2	2
460	2018353427	2	20	2	2	2	2
461	2018353428	2	19	2	2	2	2
462	2018353429	2	20	1	2	1	1
463	2018353430	2	20	1	2	1	2
464	2018353526	2	19	2	2	1	2
465	2018353606	2	20	2	2	1	2
466	2018353806	2	20	2	2	1	2
467	2018353909	1	20	2	2	1	2
468	2018354026	2	20	1	2	1	4
469	2018364202	2	21	2	2	4	2
470	2018364203	1	20	2	2	4	2
471	2018364204	2	20	1	2	4	4
472	2018364206	2	19	1	2	4	2
473	2018364207	1	19	1	2	4	4
474	2018364208	2	20	1	2	4	4
475	2018364209	1	20	2	2	4	4
476	2018364210	2	20	2	2	4	4
477	2018364213	1	20	2	2	4	2
478	2018364214	2	19	2	2	4	2
479	2018364216	2	20	1	2	4	4
480	2018364217	1	19	2	2	4	2
481	2018364218	2	20	1	2	4	2
482	2018364219	1	20	2	2	4	2
483	2018364222	2	20	2	2	4	3
484	2018364224	2	20	1	2	4	2
485	2018364225	2	19	2	2	4	2
486	2018364226	2	20	2	2	4	2
487	2018364227	2	19	2	2	4	2
488	2018364228	2	20	1	2	4	2
489	2018364229	2	20	2	2	4	4
490	2018364232	2	20	2	2	4	2
491	2018364233	2	20	2	2	4	2
492	2018364234	2	19	2	2	4	2
493	2018364235	2	19	1	2	4	4
494	2018364302	2	21	1	2	4	2
495	2018364306	2	20	1	2	1	4
496	2018364318	2	20	1	2	4	2
497	2018364321	2	21	1	2	4	2
498	2018364328	2	20	2	2	4	1

499	2018364403	1	19	2	2	4	2
500	2018364408	2	20	1	2	4	2
501	2018364422	2	21	1	2	4	4
502	2018364423	2	20	2	2	4	2
503	2018364425	2	19	1	2	4	2
504	2018364429	2	20	2	2	4	2
505	2018364503	1	19	2	2	4	1
506	2018364504	2	22	1	2	1	4
507	2018364512	2	20	1	2	1	4
508	2018364516	2	19	2	2	4	2
509	2018364518	2	20	1	2	4	2
510	2018364521	1	20	2	2	4	2
511	2018364525	2	20	1	2	4	2
512	2018364526	2	19	2	2	4	2
513	2018364723	2	22	1	2	3	2
514	2018364728	2	19	1	2	3	4
515	2018364801	1	20	1	2	3	2
516	2018364831	2	19	1	2	3	2
517	2018364916	2	20	2	2	3	2
518	2018364929	2	22	1	2	3	4
519	2018365028	2	20	2	2	3	2
520	2018365030	2	21	1	2	3	2
521	2018365206	2	20	1	2	1	2
522	2018365629	2	20	2	2	2	3
523	2018365710	2	19	1	2	1	2
524	2018365802	2	20	2	2	4	4
525	2018365806	2	20	1	2	2	3
526	2018365828	2	20	2	2	2	3
527	2018372120	2	19	2	2	1	3
528	2019328301	2	19	1	1	1	2
529	2019328302	1	19	2	1	1	2
530	2019328303	2	18	2	1	1	2
531	2019328304	1	19	2	1	1	3
532	2019328305	2	19	2	1	1	4
533	2019328306	1	19	2	1	1	2
534	2019328307	2	19	2	1	1	3
535	2019328308	1	18	2	1	1	4
536	2019328309	2	19	2	1	1	2
537	2019328310	1	19	2	1	1	2
538	2019328311	2	19	2	1	1	2
539	2019328313	2	19	1	1	1	2
540	2019328314	1	19	2	1	1	2
541	2019328315	2	19	2	1	1	2
542	2019328316	1	19	2	1	1	2
543	2019328317	2	19	2	1	1	2
544	2019328318	1	19	2	1	1	2
545	2019328319	2	18	2	1	1	2
546	2019328320	1	19	2	1	1	2
547	2019328321	2	19	2	1	1	1
548	2019328322	1	19	2	1	1	2
549	2019328323	1	21	1	1	1	2
550	2019328324	1	18	2	1	1	2
551	2019328325	1	20	1	1	1	4
552	2019328401	2	19	1	1	1	2
553	2019328402	1	18	2	1	1	2
554	2019328403	2	19	2	1	1	2

555	2019328409	2	19	2	1	1	2
556	2019328413	2	19	1	1	1	2
557	2019328415	2	19	2	1	1	2
558	2019328418	1	19	2	1	1	4
559	2019328419	2	20	2	1	1	1
560	2019328501	2	18	2	1	1	2
561	2019328504	1	19	2	1	1	1
562	2019328505	2	19	1	1	1	4
563	2019328508	1	19	1	1	1	4
564	2019328509	2	20	1	1	1	3
565	2019328512	1	19	2	1	1	2
566	2019328513	2	19	2	1	1	4
567	2019328515	2	17	2	1	1	3
568	2019328516	1	19	2	1	1	2
569	2019328517	2	19	2	1	1	2
570	2019328519	2	19	2	1	1	4
571	2019328522	1	19	2	1	1	2
572	2019328603	2	19	1	1	1	2
573	2019328615	2	19	2	1	1	2
574	2019328630	1	18	2	1	1	2
575	2019328706	1	18	2	1	1	4
576	2019328707	2	19	2	1	1	4
577	2019328711	2	19	2	1	1	2
578	2019328721	2	19	2	1	1	2
579	2019328804	2	20	1	1	1	2
580	2019328805	1	18	1	1	1	2
581	2019328806	2	20	1	1	1	2
582	2019328810	2	19	1	1	1	2
583	2019328814	2	18	2	1	1	4
584	2019328815	1	19	2	1	1	2
585	2019328816	2	19	1	1	1	2
586	2019328818	2	19	2	1	1	3
587	2019328819	1	19	2	1	1	4
588	2019328820	2	19	1	1	1	4
589	2019328822	2	19	2	1	1	2
590	2019328824	2	19	1	1	1	4
591	2019328827	2	19	2	1	1	2
592	2019340804	2	19	2	1	1	2
593	2019340806	2	18	1	1	1	2
594	2019340808	2	19	1	1	1	2
595	2019340810	2	19	2	1	1	2
596	2019340815	1	18	2	1	1	2
597	2019340818	2	20	2	1	1	2
598	2019340824	2	20	2	1	1	4
599	2019340912	2	19	2	1	1	2
600	2019340916	2	20	1	1	1	2
601	2019340926	1	19	1	1	1	4
602	2019341002	2	19	2	1	1	2
603	2019341006	2	19	1	1	1	4
604	2019341017	1	19	2	1	1	4
605	2019341601	1	18	1	1	1	4
606	2019341602	2	19	2	1	1	3
607	2019341603	1	19	2	1	1	4
608	2019341604	2	19	2	1	1	2
609	2019341605	1	19	1	1	1	1
610	2019341606	2	20	2	1	1	4

611	2019341607	1	19	2	1	1	2
612	2019341608	2	19	2	1	1	2
613	2019341610	2	20	2	1	1	1
614	2019341611	1	19	2	1	1	4
615	2019341612	2	18	2	1	1	2
616	2019341614	2	18	2	1	1	3
617	2019341616	2	19	1	1	1	2
618	2019341617	1	19	1	1	1	1
619	2019341618	2	20	1	1	1	2
620	2019341619	1	19	2	1	1	4
621	2019341620	2	19	2	1	1	2
622	2019341622	2	19	1	1	1	2
623	2019341623	2	20	1	1	1	1
624	2019341625	1	19	2	1	1	4
625	2019341626	1	18	2	1	1	2
626	2019341701	1	18	1	1	1	2
627	2019341702	2	20	2	1	1	2
628	2019341704	2	19	1	1	1	2
629	2019341705	1	20	2	1	1	3
630	2019341707	1	19	2	1	1	2
631	2019341708	2	19	2	1	1	4
632	2019341709	1	18	2	1	1	1
633	2019341710	2	19	2	1	1	1
634	2019341711	1	19	2	1	1	2
635	2019341712	2	19	2	1	1	2
636	2019341714	2	19	2	1	1	2
637	2019341716	2	19	1	1	1	2
638	2019341717	1	20	1	1	1	2
639	2019341718	2	20	2	1	1	4
640	2019341719	1	19	2	1	1	3
641	2019341720	2	18	2	1	1	4
642	2019341721	1	22	1	1	1	4
643	2019341722	2	19	2	1	1	2
644	2019341723	2	19	2	1	1	2
645	2019341724	2	20	2	1	1	4
646	2019341725	1	19	1	1	1	3
647	2019341726	1	19	1	1	1	2
648	2019341802	2	20	2	1	1	2
649	2019341804	2	20	2	1	1	2
650	2019341805	1	20	2	1	1	2
651	2019341806	2	19	2	1	1	2
652	2019341808	2	18	2	1	1	2
653	2019341809	1	18	2	1	1	4
654	2019341810	2	17	2	1	1	2
655	2019341811	1	20	2	1	1	2
656	2019341812	2	20	2	1	1	2
657	2019341813	1	19	1	1	1	1
658	2019341814	2	19	2	1	1	2
659	2019341815	1	19	2	1	1	2
660	2019341816	2	19	2	1	1	2
661	2019341817	1	19	1	1	1	2
662	2019341818	2	19	1	1	1	4
663	2019341819	1	19	2	1	1	2
664	2019341820	2	19	1	1	1	2
665	2019341822	2	18	1	1	1	4
666	2019341823	2	19	1	1	1	4

667	2019341824	2	18	1	1	1	2
668	2019341824	1	20	1	1	1	2
669	2019341825	2	18	2	1	1	2
670	2019341826	1	20	2	1	1	2
671	2019341901	1	19	2	1	1	2
672	2019341902	2	19	2	1	1	2
673	2019341903	1	19	1	1	1	2
674	2019341904	2	18	2	1	1	2
675	2019341905	1	20	1	1	1	2
676	2019341907	1	19	2	1	1	2
677	2019341908	2	19	2	1	1	2
678	2019341909	1	19	2	1	1	2
679	2019341910	2	19	2	1	1	1
680	2019341911	1	20	2	1	1	2
681	2019341912	2	19	2	1	1	2
682	2019341914	2	19	2	1	1	2
683	2019341915	1	20	2	1	1	2
684	2019341916	2	19	2	1	1	2
685	2019341917	1	20	2	1	1	2
686	2019341918	2	19	2	1	1	2
687	2019341919	1	19	2	1	1	2
688	2019341920	2	19	1	1	1	2
689	2019341921	1	20	1	1	1	4
690	2019341922	2	18	2	1	1	2
691	2019341923	2	18	2	1	1	2
692	2019341924	2	19	2	1	1	2
693	2019341925	2	20	2	1	1	4
694	2019341926	1	19	2	1	1	3
695	2019342001	1	20	1	1	1	4
696	2019342002	2	19	1	1	1	4
697	2019342003	1	19	1	1	1	2
698	2019342004	2	19	2	1	1	2
699	2019342005	1	19	2	1	1	2
700	2019342006	2	18	2	1	1	2
701	2019342008	2	19	2	1	1	3
702	2019342009	1	19	2	1	1	2
703	2019342010	2	19	2	1	1	4
704	2019342011	1	19	2	1	1	2
705	2019342012	2	19	2	1	1	2
706	2019342014	2	19	2	1	1	2
707	2019342015	1	19	2	1	1	2
708	2019342016	2	19	2	1	1	2
709	2019342017	1	18	2	1	1	2
710	2019342017	1	18	2	1	1	2
711	2019342017	1	19	1	1	1	2
712	2019342018	2	18	1	1	1	2
713	2019342019	1	21	2	1	1	2
714	2019342020	2	18	2	1	1	2
715	2019342021	1	20	2	1	1	2
716	2019342023	2	19	1	1	1	4
717	2019342024	2	20	2	1	1	2
718	2019342025	2	19	2	1	1	2
719	2019342101	1	20	1	1	1	4
720	2019342102	2	20	2	1	1	2
721	2019342103	1	25	2	1	1	2
722	2019342104	2	19	2	1	1	2

723	2019342105	1	19	2	1	1	2
724	2019342106	2	19	1	1	1	2
725	2019342107	1	19	2	1	1	2
726	2019342108	2	19	2	1	1	2
727	2019342109	1	20	1	1	1	2
728	2019342110	2	19	1	1	1	2
729	2019342111	1	21	2	1	1	1
730	2019342112	2	19	2	1	1	2
731	2019342113	1	19	2	1	1	1
732	2019342114	2	19	2	1	1	2
733	2019342115	1	19	2	1	1	2
734	2019342116	2	19	1	1	1	3
735	2019342117	1	19	1	1	1	2
736	2019342118	2	19	2	1	1	4
737	2019342119	1	19	2	1	1	2
738	2019342120	2	19	2	1	1	2
739	2019342122	2	19	1	1	1	2
740	2019342123	2	19	2	1	1	2
741	2019342124	2	20	1	1	1	3
742	2019342125	2	19	1	1	1	2
743	2019342201	1	21	2	1	1	2
744	2019342202	2	20	1	1	1	4
745	2019342203	1	18	1	1	1	2
746	2019342204	2	19	2	1	1	2
747	2019342205	1	19	1	1	1	2
748	2019342206	2	20	2	1	1	2
749	2019342207	1	19	2	1	1	4
750	2019342208	2	19	2	1	1	2
751	2019342210	2	19	2	1	1	2
752	2019342211	1	18	2	1	1	2
753	2019342212	2	19	1	1	1	2
754	2019342213	1	19	2	1	1	2
755	2019342214	2	18	2	1	1	2
756	2019342216	2	19	2	1	1	3
757	2019342217	1	19	2	1	1	3
758	2019342218	2	19	2	1	1	2
759	2019342219	1	18	1	1	1	2
760	2019342220	2	19	2	1	1	2
761	2019342221	1	18	2	1	1	2
762	2019342222	2	19	2	1	1	4
763	2019342223	2	20	2	1	1	2
764	2019342224	2	19	2	1	1	2
765	2019342225	2	18	2	1	1	4
766	2019352501	1	20	2	1	1	2
767	2019352502	2	19	1	1	1	4
768	2019352503	1	20	2	1	1	1
769	2019352504	2	20	2	1	1	2
770	2019352505	1	19	2	1	1	2
771	2019352506	2	18	2	1	1	2
772	2019352507	1	19	1	1	1	2
773	2019352508	2	20	2	1	1	2
774	2019352509	1	20	1	1	1	2
775	2019352512	2	19	2	1	1	2
776	2019352513	1	20	1	1	1	2
777	2019352514	2	18	1	1	1	2
778	2019352515	1	20	2	1	1	1

779	2019352516	2	18	2	1	1	4
780	2019352517	2	20	2	1	1	4
781	2019352518	2	19	2	1	1	4
782	2019352520	2	19	1	1	1	2
783	2019352521	2	18	2	1	1	2
784	2019352522	2	18	2	1	1	1
785	2019352523	2	19	2	1	1	2
786	2019352524	2	20	2	1	1	4
787	2019352525	2	18	2	1	1	2
788	2019352601	1	18	2	1	1	2
789	2019352602	2	19	1	1	1	1
790	2019352604	2	19	2	1	1	2
791	2019352605	1	18	2	1	1	2
792	2019352606	2	19	1	1	1	2
793	2019352607	1	19	2	1	1	2
794	2019352608	2	18	2	1	1	2
795	2019352609	1	19	2	1	1	4
796	2019352610	2	19	1	1	1	2
797	2019352611	1	18	2	1	1	2
798	2019352612	2	19	2	1	1	2
799	2019352613	1	21	2	1	1	3
800	2019352614	2	19	2	1	1	2
801	2019352615	1	20	2	1	1	4
802	2019352616	2	19	1	1	1	2
803	2019352617	2	18	1	1	1	4
804	2019352618	2	19	2	1	1	2
805	2019352619	2	19	2	1	1	2
806	2019352620	2	19	2	1	1	2
807	2019352621	2	20	2	1	1	2
808	2019352622	2	20	2	1	1	2
809	2019352623	2	18	2	1	1	2
810	2019352624	2	19	2	1	1	2
811	2019352625	2	19	2	1	1	4
812	2019352702	2	19	1	1	1	2
813	2019352704	2	18	2	1	1	4
814	2019352705	1	18	1	1	1	4
815	2019352706	2	19	2	1	1	2
816	2019352708	2	19	2	1	1	2
817	2019352709	2	19	2	1	1	4
818	2019352711	2	19	1	1	1	2
819	2019352712	2	19	2	1	1	2
820	2019352713	2	18	2	1	1	4
821	2019352714	2	20	2	1	1	2
822	2019352715	2	20	2	1	1	2
823	2019352716	2	19	2	1	1	4
824	2019352717	2	20	2	1	1	4
825	2019352718	2	19	2	1	1	4
826	2019352719	2	20	1	1	1	2
827	2019352720	2	18	2	1	1	2
828	2019352721	2	20	2	1	1	2
829	2019352722	2	19	1	1	1	4
830	2019352723	2	19	1	1	1	2
831	2019352724	2	20	2	1	1	1
832	2019352725	2	18	2	1	1	2
833	2019352726	2	20	1	1	1	2
834	2019352727	2	20	1	1	1	4

835	2019352728	2	20	1	1	1	2
836	2019352730	2	19	2	1	1	2
837	2019352906	2	19	2	1	1	2
838	2019352913	2	19	2	1	1	3
839	2019352919	2	18	2	1	1	2
840	2019352924	2	19	2	1	1	2
841	2019352926	2	18	2	1	1	2
842	2019352927	2	18	1	1	1	4
843	2019353015	2	18	2	1	1	4
844	2019353301	1	19	1	1	2	4
845	2019353302	2	19	1	1	2	2
846	2019353303	1	19	1	1	2	2
847	2019353305	1	21	1	1	2	4
848	2019353306	2	18	2	1	2	2
849	2019353307	1	18	2	1	2	2
850	2019353309	1	20	1	1	2	4
851	2019353310	2	20	1	1	2	2
852	2019353311	1	20	1	1	2	2
853	2019353312	2	19	1	1	2	4
854	2019353313	1	19	2	1	2	2
855	2019353314	2	18	2	1	2	2
856	2019353315	1	19	2	1	2	2
857	2019353316	2	18	2	1	2	2
858	2019353317	1	21	1	1	2	4
859	2019353319	2	20	2	1	2	2
860	2019353320	2	20	1	1	2	2
861	2019353321	2	20	2	1	2	4
862	2019353322	2	18	2	1	2	2
863	2019353323	2	19	2	1	2	3
864	2019353324	2	20	1	1	2	4
865	2019353325	2	19	2	1	2	2
866	2019353402	2	20	1	1	2	1
867	2019353403	1	19	1	1	2	2
868	2019353404	2	18	2	1	2	2
869	2019353412	2	19	1	1	2	2
870	2019353414	2	19	1	1	2	2
871	2019353418	2	19	2	1	2	2
872	2019353419	2	20	2	1	2	2
873	2019353420	2	18	2	1	2	2
874	2019353421	2	19	2	1	2	2
875	2019353422	2	19	2	1	2	2
876	2019353423	2	19	2	1	2	2
877	2019353501	1	19	2	1	2	4
878	2019353502	2	19	1	1	2	2
879	2019353503	1	19	2	1	2	2
880	2019353504	2	19	2	1	2	2
881	2019353506	2	19	1	1	2	2
882	2019353507	1	19	2	1	2	2
883	2019353508	2	20	1	1	2	2
884	2019353509	1	20	1	1	2	2
885	2019353510	2	19	1	1	2	2
886	2019353511	1	19	2	1	2	2
887	2019353512	2	19	1	1	2	2
888	2019353514	2	19	2	1	2	4
889	2019353515	1	20	2	1	2	2
890	2019353516	2	19	2	1	2	2

891	2019353517	2	19	2	1	2	2
892	2019353518	2	18	2	1	2	2
893	2019353519	2	19	2	1	2	2
894	2019353520	2	18	1	1	2	2
895	2019353521	2	19	1	1	2	2
896	2019353522	2	20	2	1	2	2
897	2019353523	2	20	2	1	2	2
898	2019353525	1	20	1	1	2	4
899	2019364401	1	19	1	1	4	2
900	2019364402	2	18	1	1	4	4
901	2019364403	1	20	1	1	1	4
902	2019364404	2	19	1	1	4	2
903	2019364405	1	21	1	1	4	2
904	2019364406	2	19	2	1	1	4
905	2019364407	1	19	2	1	1	2
906	2019364408	2	19	2	1	4	2
907	2019364409	1	20	1	1	4	2
908	2019364410	2	19	2	1	4	2
909	2019364411	1	20	1	1	4	1
910	2019364412	2	19	2	1	4	2
911	2019364413	1	18	2	1	4	2
912	2019364414	2	20	1	1	4	2
913	2019364415	1	19	2	1	1	2
914	2019364416	2	18	1	1	4	2
915	2019364417	1	20	2	1	1	2
916	2019364418	2	19	2	1	4	2
917	2019364420	2	18	2	1	4	2
918	2019364421	1	19	2	1	4	2
919	2019364422	2	19	1	1	4	2
920	2019364423	1	20	1	1	4	2
921	2019364424	2	18	1	1	4	2
922	2019364424	2	18	1	1	4	2
923	2019364425	2	19	1	1	1	2
924	2019364425	2	19	1	1	1	2
925	2019364501	1	19	1	1	4	2
926	2019364502	2	18	1	1	4	2
927	2019364503	1	18	1	1	1	2
928	2019364504	2	19	1	1	4	2
929	2019364505	1	18	2	1	1	2
930	2019364506	2	19	1	1	4	2
931	2019364508	2	19	2	1	4	2
932	2019364509	1	19	1	1	1	2
933	2019364510	2	19	2	1	4	2
934	2019364512	2	19	1	1	4	4
935	2019364513	1	19	1	1	4	2
936	2019364514	2	20	1	1	4	4
937	2019364516	2	19	2	1	4	2
938	2019364517	1	20	1	1	4	1
939	2019364518	2	19	2	1	4	3
940	2019364519	1	20	1	1	1	2
941	2019364520	2	20	1	1	4	4
942	2019364521	1	20	1	1	1	2
943	2019364522	2	20	1	1	4	2
944	2019364522	2	20	1	1	4	2
945	2019364523	2	19	1	1	4	2
946	2019364524	2	19	2	1	4	4

947	2019364602	2	20	1	1	4	2
948	2019364616	2	19	2	1	4	2
949	2019364625	2	20	1	1	4	4
950	2019364701	1	18	1	1	4	2
951	2019364705	1	20	1	1	4	4
952	2019364709	1	19	2	1	4	2
953	2019364711	1	20	2	1	1	3
954	2019364713	1	19	2	1	4	1
955	2019364714	2	18	2	1	4	2
956	2019364718	2	19	1	1	4	3
957	2019364720	2	18	2	1	4	2
958	2019364727	2	19	2	1	4	3
959	2019364729	2	19	2	1	4	1
960	2019364814	2	18	2	1	3	2
961	2019364816	2	19	2	1	3	2
962	2019364905	1	19	2	1	3	2
963	2019364907	1	19	2	1	3	4
964	2019364914	2	19	2	1	3	2
965	2019364916	2	19	1	1	3	2
966	2019364923	2	19	2	1	3	2
967	2019364926	2	19	1	1	3	2
968	2019364927	2	18	2	1	3	2
969	2019365004	2	19	1	1	3	2
970	2019365010	2	20	2	1	3	3
971	2019365016	2	19	2	1	3	4
972	2019365118	2	20	2	1	3	3
973	2019365205	1	19	2	1	3	2
974	2019365206	2	19	1	1	3	2
975	2019365211	2	19	2	1	3	3
976	2019365220	2	19	2	1	3	4
977	2019365228	2	20	2	1	3	2
978	2019365414	2	19	1	1	3	2
979	2019365417	2	19	2	1	3	4
980	2019365420	2	19	2	1	3	4
981	2019365509	2	19	2	1	3	4
982	2019365511	2	19	2	1	3	2
983	2019366415	1	18	2	1	2	3
984	2019366416	2	18	1	1	2	2
985	2019366425	1	19	2	1	2	2
986	2019432022	2	20	1	1	1	2
987	2018352427	2	20	1	2	1	3
988	2018352505	1	20	2	2	1	2
989	2018353314	2	18	1	2	2	2
990	2019341906	2	20	1	1	1	2
991		1	20	2	1	1	4
992		1	20	2	3	3	2
993		1	24	2	4	2	1
994		1	20	2	2	1	2
995		1	19	2	2	2	4

学习时长 (learning duration)	totalenvironmental	de1	de2	de3	de4	de5	e1	e2	e3	e4	e5
3	212	52	39	40	52	29	5	5	5	1	5
3	175	43	33	32	42	25	4	4	4	2	4
3	212	53	39	35	56	29	5	5	5	1	5
2	148	37	26	28	35	22	3	3	3	2	3
1	179	44	36	32	43	24	2	5	3	3	4
2	191	52	38	31	45	25	5	5	3	1	3
2	234	56	47	40	56	35	5	5	5	5	5
3	175	43	34	32	44	22	4	4	4	2	4
4	147	36	32	22	38	19	2	3	2	3	3
5	223	55	41	39	54	34	5	5	5	5	5
1	201	50	38	33	52	28	5	5	5	1	4
1	166	42	36	25	42	21	3	5	3	3	3
2	208	55	37	33	52	31	5	5	5	2	2
2	175	47	36	28	39	25	4	4	4	3	3
6	214	52	39	39	52	32	5	5	5	2	5
4	191	46	40	31	47	27	5	5	4	2	4
1	185	46	37	31	42	29	5	5	5	2	4
2	192	47	34	34	48	29	4	4	4	2	4
3	187	47	37	29	47	27	4	5	4	1	3
3	157	43	33	22	36	23	5	5	4	3	3
3	217	54	39	38	55	31	5	5	5	2	4
6	246	60	51	40	60	35	5	5	5	5	5
4	158	40	37	26	36	19	5	5	5	3	5
2	211	53	38	37	53	30	5	5	5	1	4
3	161	43	32	24	39	23	5	5	4	2	2
2	206	52	38	35	50	31	5	5	5	2	3
1	180	46	38	31	40	25	5	5	3	2	4
6	183	44	38	30	44	27	5	5	5	1	3
1	174	44	33	31	43	23	5	5	3	1	4
1	174	48	37	24	40	25	5	5	4	2	2
6	175	43	37	25	43	27	3	4	4	2	2
3	216	53	39	40	52	32	5	5	5	2	5
2	175	43	36	29	42	25	3	4	3	4	4
1	174	44	35	28	41	26	4	4	4	3	4
5	203	42	41	36	52	32	5	5	4	4	3
4	209	55	39	38	49	28	5	5	5	2	5
4	183	43	37	33	44	26	4	5	4	2	4
1	148	36	31	24	36	21	3	3	3	3	3
1	189	50	35	33	45	26	5	5	5	2	4
3	164	38	35	25	39	27	4	5	4	3	4
2	164	40	33	26	41	24	4	4	4	3	3
3	154	37	35	24	36	22	4	4	4	3	3
5	167	43	29	27	40	28	4	3	3	4	4
3	200	48	35	38	54	25	1	1	1	1	5
2	172	43	33	29	43	24	4	5	4	2	3
4	202	51	39	33	51	28	5	5	5	2	3
4	213	52	39	39	52	31	5	5	5	2	4
2	250	60	55	40	60	35	5	5	5	5	5
5	167	43	32	28	39	25	3	4	4	2	3
2	212	51	39	38	55	29	5	5	5	2	5

6	212	51	39	36	53	33	4	5	5	5	5
1	156	36	36	26	35	23	3	4	3	2	4
2	204	51	39	34	50	30	5	5	5	2	3
2	192	46	37	34	46	29	4	5	5	2	4
3	183	46	34	33	47	23	4	5	4	1	3
2	175	43	34	30	44	24	5	5	3	2	2
3	190	50	36	32	47	25	5	5	4	2	4
6	175	44	38	30	42	21	4	4	3	3	4
2	177	44	35	29	44	25	3	5	5	1	3
2	183	43	37	29	45	29	5	5	4	4	3
2	172	42	34	26	43	27	4	4	4	3	2
3	197	51	38	33	48	27	4	5	3	1	4
2	197	48	38	36	47	28	5	5	4	1	4
6	210	60	39	40	42	29	5	5	5	3	5
3	169	42	33	32	41	21	4	4	4	3	4
3	187	45	37	32	45	28	4	4	4	4	4
1	201	52	35	32	51	31	5	5	5	2	3
2	174	43	33	31	43	24	4	4	4	2	3
2	151	36	29	23	40	23	3	4	3	3	2
4	159	40	26	27	39	27	3	4	4	3	2
2	177	43	35	31	41	27	4	4	4	3	3
4	169	44	29	32	38	26	3	3	4	3	5
3	220	56	43	40	52	29	5	5	5	1	5
3	218	56	39	40	52	31	5	5	5	1	5
3	151	41	29	25	32	24	5	5	5	2	4
2	143	35	30	19	36	23	3	5	3	3	3
2	197	51	38	32	48	28	4	5	5	1	4
3	179	43	37	28	44	27	4	5	4	1	4
3	198	53	37	31	50	27	5	5	2	2	3
1	178	46	36	28	43	25	4	5	4	2	3
3	181	43	36	30	45	27	4	5	4	3	4
2	200	50	38	35	50	27	5	5	4	2	4
2	176	50	33	27	41	25	5	5	4	2	3
3	203	53	38	36	49	27	5	5	5	2	3
5	207	50	37	38	51	31	5	5	5	1	5
6	150	36	33	24	36	21	3	3	3	3	3
1	213	52	39	38	56	28	5	5	2	1	5
2	181	44	37	32	43	25	4	5	3	2	4
3	190	46	40	32	44	28	4	4	4	4	4
5	236	58	47	40	56	35	5	5	5	5	5
2	169	43	33	25	42	26	3	5	4	3	2
4	205	49	39	36	52	29	5	5	5	1	3
3	189	47	36	33	47	26	5	5	4	2	4
2	227	57	43	40	52	35	5	5	5	5	5
1	230	56	47	40	52	35	5	5	5	5	5
3	198	50	36	33	48	31	4	4	4	3	3
1	155	35	27	27	42	24	3	3	3	3	4
1	176	45	33	31	42	25	5	4	4	3	3
2	205	51	39	34	52	29	5	5	5	2	3
1	143	34	30	24	34	21	3	3	3	3	3
4	169	41	31	31	41	25	4	4	4	1	4
2	209	49	37	39	53	31	4	4	4	2	4
3	184	46	36	32	44	26	5	5	4	2	4
2	218	55	39	38	54	32	4	5	5	2	3
3	180	44	34	30	47	25	5	5	4	1	3
2	209	50	39	40	50	30	5	5	4	1	5

4	218	48	47	36	56	31	1	5	5	1	1
4	146	36	30	21	36	23	3	4	3	3	2
3	163	41	33	23	43	23	3	4	3	3	1
2	222	56	39	40	56	31	5	5	5	1	5
3	175	42	35	30	42	26	4	5	4	2	3
3	218	52	39	40	56	31	5	5	5	1	5
1	212	52	39	40	50	31	5	5	5	1	5
4	201	52	37	35	50	27	5	5	5	1	3
6	178	49	37	26	40	26	4	4	4	2	4
3	187	43	36	34	47	27	3	5	3	1	4
2	213	52	39	38	52	32	5	5	5	2	5
2	175	42	32	29	45	27	4	4	4	3	4
3	177	44	36	29	41	27	4	4	4	3	3
2	204	51	39	37	51	26	5	5	5	3	4
2	154	39	28	26	36	25	4	4	4	4	3
5	181	44	33	31	47	26	3	5	3	2	3
5	250	60	55	40	60	35	5	5	5	5	5
2	159	35	35	26	39	24	2	5	4	1	2
6	250	60	55	40	60	35	5	5	5	5	5
3	208	51	37	37	53	30	5	5	5	3	3
4	164	43	28	30	41	22	4	4	4	2	4
1	155	37	35	25	36	22	3	3	3	3	3
3	171	46	33	28	39	25	4	5	3	2	4
3	172	43	33	32	42	22	4	5	4	2	4
4	190	48	37	32	46	27	5	5	5	2	3
3	174	42	33	32	43	24	3	4	3	2	4
2	209	50	38	37	53	31	5	5	5	1	4
1	163	40	32	28	39	24	3	4	3	4	4
6	202	48	39	32	54	29	5	5	5	3	1
4	151	42	32	16	38	23	5	5	3	3	2
1	144	37	29	23	34	21	3	3	3	3	3
3	160	39	31	31	38	21	3	4	3	3	2
3	157	39	32	27	36	23	4	4	4	2	4
3	130	31	28	17	33	21	3	4	3	3	2
4	213	54	39	35	53	32	5	5	5	3	3
4	207	54	39	34	51	29	5	5	5	3	4
3	167	41	32	25	40	29	3	5	5	3	3
5	195	49	39	33	47	27	5	5	5	3	5
3	222	57	42	37	54	32	5	5	5	2	2
1	116	26	33	16	24	17	1	5	1	5	5
2	148	36	32	25	35	20	4	4	4	2	4
3	179	45	38	25	48	23	5	5	3	3	2
4	180	42	36	28	46	28	3	5	3	3	2
2	185	47	37	30	46	25	5	5	4	2	2
2	150	36	33	24	36	21	3	3	3	3	3
3	176	43	32	30	46	25	4	4	4	1	3
4	172	39	38	25	40	30	5	5	5	3	3
4	158	40	34	25	36	23	3	5	3	2	3
1	214	52	39	40	52	31	5	5	5	1	5
6	250	60	55	40	60	35	5	5	5	5	5
2	221	56	39	40	56	30	5	5	5	1	5
2	206	51	38	35	51	31	4	5	5	2	5
2	183	44	37	31	44	27	4	4	4	3	4
2	214	51	40	37	54	32	5	5	5	2	4
2	197	47	46	32	47	25	4	5	4	2	4
4	190	46	40	30	48	26	3	3	3	3	3

2	206	52	37	36	51	30	3	5	4	2	4
1	210	53	39	39	50	29	5	5	5	2	4
2	188	47	38	33	48	22	5	5	5	1	4
2	197	48	43	30	49	27	4	4	3	4	3
1	218	54	41	38	53	32	5	5	4	3	5
4	188	49	39	29	46	25	5	5	3	4	3
2	204	52	37	37	51	27	5	5	5	2	4
2	200	48	44	32	48	28	4	4	4	4	4
1	160	39	33	26	36	26	5	5	4	4	3
6	219	52	39	40	56	32	5	5	5	2	5
5	213	52	39	39	52	31	5	5	5	1	4
2	176	44	34	29	43	26	4	5	3	2	4
3	148	34	37	18	34	25	4	4	4	4	1
6	196	50	35	31	49	31	5	5	5	4	4
4	201	51	37	35	51	27	5	5	5	1	3
1	228	55	45	40	53	35	5	5	5	5	5
3	218	52	39	40	52	35	5	5	5	5	5
2	150	36	33	24	36	21	3	3	3	3	3
3	193	48	36	33	48	28	5	5	5	1	4
6	199	53	40	33	48	25	5	5	3	4	3
2	211	55	40	38	49	29	4	4	3	4	4
2	187	46	39	25	49	28	5	5	4	5	3
3	181	45	33	30	48	25	5	5	4	2	2
5	150	36	33	24	36	21	3	3	3	3	3
2	177	46	34	27	45	25	4	4	4	4	4
5	186	48	39	27	46	26	4	4	3	3	3
3	213	52	39	39	51	32	5	5	5	2	5
5	195	50	39	32	47	27	4	5	3	2	4
1	197	51	41	28	44	33	5	5	5	3	3
1	199	52	40	27	53	27	5	5	4	5	3
3	184	45	33	33	44	29	4	5	3	3	3
3	212	50	39	39	52	32	3	5	5	2	4
3	218	52	43	40	52	31	5	5	5	1	5
2	213	52	39	40	51	31	5	5	5	1	5
3	188	51	36	28	46	27	5	5	5	2	1
3	180	45	36	30	45	24	3	5	3	2	3
6	141	35	28	23	38	17	3	3	3	1	3
2	199	52	36	31	49	31	5	5	5	3	4
5	168	41	32	28	41	26	4	4	4	3	4
3	182	43	36	32	44	27	4	5	5	2	4
4	152	36	33	24	38	21	3	3	3	3	3
4	214	52	39	40	52	31	5	5	5	1	5
2	203	54	40	33	50	26	5	5	5	2	4
5	183	46	33	29	48	27	3	4	3	3	4
2	172	44	35	29	41	23	4	5	3	3	4
2	182	46	32	31	46	27	4	4	4	3	3
3	180	44	35	30	46	25	4	4	4	2	4
3	180	46	36	30	43	25	5	5	4	2	4
1	174	46	32	28	43	25	4	4	3	2	2
4	178	45	32	32	43	26	4	4	4	2	4
2	206	52	39	34	51	30	5	5	5	2	3
2	214	52	39	40	52	31	5	5	5	1	5
3	217	54	40	39	54	30	5	5	5	2	5
1	162	42	30	26	42	22	3	3	2	3	3
2	179	46	32	32	43	26	3	4	4	2	4
2	200	49	39	33	50	29	5	5	5	1	5

1	177	44	33	33	41	26	4	4	4	2	4
1	175	44	37	27	43	24	5	5	4	2	4
5	232	56	45	40	60	31	5	5	5	1	5
1	162	41	35	28	38	20	4	5	2	3	4
6	221	54	41	40	54	32	5	5	5	2	5
1	209	52	39	39	52	27	5	5	5	1	5
3	175	43	38	26	43	25	4	5	3	2	3
6	207	53	37	34	52	31	5	5	5	2	4
3	194	48	37	35	47	27	4	5	4	2	4
4	179	43	39	30	45	22	4	4	3	1	3
2	200	49	37	35	51	28	5	5	5	2	3
2	179	43	35	32	45	24	4	4	3	1	4
5	182	44	36	31	44	27	4	5	4	2	3
3	189	48	37	30	47	27	4	4	4	2	4
1	207	53	39	34	51	30	4	5	5	2	4
5	216	52	43	36	54	31	5	5	5	1	4
5	228	56	47	38	56	31	5	5	5	1	3
4	194	50	39	32	44	29	3	5	5	3	4
2	176	43	38	27	40	28	4	4	4	4	3
3	203	52	39	32	50	30	5	5	5	2	4
3	196	51	39	36	45	25	5	5	4	1	5
3	161	39	33	29	43	17	3	5	1	1	5
2	209	53	39	35	51	31	5	5	4	3	4
3	208	53	39	34	51	31	5	5	5	1	2
2	214	54	41	37	51	31	5	5	5	2	3
3	177	44	32	31	44	26	4	4	4	2	3
2	209	51	39	38	50	31	5	5	5	1	4
3	170	42	38	27	41	22	4	4	3	3	4
1	199	51	37	36	48	27	5	5	5	1	4
2	185	47	35	32	45	26	4	5	4	2	3
5	193	49	36	32	47	29	5	4	3	2	4
2	218	52	43	38	52	33	5	5	5	3	4
3	206	49	39	37	52	29	5	5	5	1	2
3	183	49	37	31	40	26	4	5	4	2	3
6	205	51	39	36	49	30	5	5	5	1	5
3	227	57	48	38	53	31	5	5	5	2	5
2	192	46	38	34	47	27	5	5	5	1	5
4	214	53	39	38	53	31	5	5	5	1	4
3	198	48	40	33	50	27	3	5	5	1	3
2	203	51	39	36	49	28	5	5	4	1	3
3	204	51	38	34	54	27	5	5	5	2	4
2	208	48	39	38	52	31	1	5	5	1	5
3	210	52	39	36	52	31	5	5	5	1	5
2	182	46	36	32	43	25	4	5	4	2	3
4	175	46	34	29	40	26	4	4	4	4	4
2	222	57	43	38	54	30	5	5	5	5	5
3	193	49	38	34	49	23	5	5	5	1	4
1	164	42	36	24	39	23	4	4	4	3	2
2	155	39	32	23	38	23	3	4	3	3	2
4	165	42	32	25	38	28	4	5	4	4	4
4	198	50	39	32	50	27	4	5	4	4	4
3	181	42	38	31	45	25	3	4	3	2	3
1	203	50	39	36	50	28	3	5	3	1	3
2	195	50	38	32	49	26	3	5	4	1	3
2	186	46	36	32	45	27	5	5	5	2	4
6	141	35	28	25	33	20	3	3	3	2	3

4	218	53	40	40	52	33	5	5	5	3	5
4	199	51	37	35	45	31	5	5	5	2	4
4	211	49	39	40	52	31	5	5	5	1	5
6	208	53	38	35	52	30	5	5	5	1	2
3	213	52	39	39	52	31	5	5	5	1	5
3	186	44	36	32	46	28	4	4	4	4	4
2	232	55	51	40	54	32	5	5	5	5	5
4	168	44	36	25	38	25	5	5	3	3	3
3	152	39	32	23	37	21	5	4	3	4	3
2	212	52	39	38	52	31	5	5	5	1	3
4	183	45	35	30	44	29	5	5	5	4	5
3	190	46	37	33	48	26	5	5	5	1	4
1	152	35	35	26	41	15	2	2	2	2	2
2	183	51	32	30	47	23	5	4	3	1	3
4	213	50	47	35	50	31	5	5	5	5	4
1	250	60	55	40	60	35	5	5	5	5	5
5	206	51	42	34	51	28	5	5	3	2	1
1	151	37	34	24	34	22	4	4	4	3	3
1	163	42	35	27	38	21	4	4	3	3	4
2	196	50	37	35	47	27	5	5	5	1	4
5	175	43	33	31	43	25	3	4	4	2	4
2	215	53	45	34	53	30	5	5	5	2	5
3	186	46	39	30	46	25	4	5	4	2	3
3	206	50	37	37	54	28	5	5	5	2	3
3	182	47	34	32	45	24	4	4	4	2	4
1	208	49	39	39	50	31	5	5	5	1	5
3	189	48	38	31	45	27	5	5	5	3	3
3	195	51	39	30	49	26	4	5	3	3	2
4	188	45	38	32	45	28	4	4	4	4	4
3	208	52	38	38	49	31	5	5	5	1	4
3	189	47	34	33	46	29	4	4	4	2	4
3	178	44	33	29	45	27	4	4	4	3	3
3	201	52	37	36	45	31	5	5	5	1	5
3	148	34	33	25	37	19	3	3	3	3	4
3	212	52	39	38	52	31	5	5	5	1	5
1	212	52	39	38	52	31	5	5	5	1	5
5	213	52	38	39	53	31	5	5	5	1	4
3	196	48	39	34	49	26	5	5	5	2	3
4	188	45	39	30	49	25	4	5	4	1	3
4	188	45	39	30	49	25	4	5	4	1	3
3	179	42	36	29	47	25	4	5	3	2	4
2	214	52	39	40	52	31	5	5	5	1	5
5	170	43	33	28	42	24	4	4	4	1	3
3	182	45	35	32	44	26	5	5	3	3	3
3	213	55	43	39	49	27	4	5	5	2	5
1	210	52	39	37	52	30	5	5	5	1	4
2	236	56	51	38	56	35	5	5	5	5	5
2	209	54	37	36	53	29	5	5	5	1	3
3	235	56	51	40	53	35	5	5	5	5	5
4	189	50	36	32	42	29	5	5	5	3	4
3	178	43	36	30	43	26	3	5	3	2	3
3	132	35	27	26	25	19	5	4	4	1	4
3	161	43	31	27	39	21	4	4	3	2	3
6	173	40	33	28	46	26	3	5	4	2	2
2	150	36	33	24	36	21	3	3	3	3	3
2	213	53	39	36	54	31	5	5	5	1	4

2	223	60	40	40	52	31	5	5	5	1	5
1	176	43	35	29	44	25	4	5	4	1	3
2	209	50	39	35	53	32	5	5	5	5	3
3	176	42	35	32	42	25	4	4	4	1	4
3	197	49	38	35	49	26	4	5	4	1	4
2	177	44	34	30	44	25	4	4	4	2	3
2	235	55	48	40	57	35	5	5	5	5	5
2	142	33	27	22	34	26	3	3	3	3	2
2	174	43	32	31	44	24	4	4	4	2	4
5	156	38	35	24	36	23	5	5	5	3	3
4	164	42	31	27	40	24	4	4	3	2	3
4	220	56	45	38	50	31	5	5	5	1	5
2	250	60	55	40	60	35	5	5	5	5	5
2	200	48	44	32	48	28	4	4	4	4	4
3	205	47	40	37	48	33	5	5	5	4	4
2	200	51	39	32	49	29	5	5	5	1	3
3	214	52	39	40	52	31	5	5	5	1	5
2	206	50	39	36	51	30	5	5	5	1	4
3	199	51	42	29	48	29	5	5	5	2	4
2	223	56	39	40	58	30	5	5	5	2	5
2	206	49	41	34	52	30	3	5	5	2	3
2	176	45	34	30	43	24	4	4	4	2	3
3	181	44	37	32	43	25	4	4	4	2	4
2	197	51	35	34	48	29	5	5	4	4	4
3	222	56	39	40	52	35	5	5	5	5	5
2	179	45	35	29	46	24	4	5	3	2	4
2	175	44	36	26	45	24	3	5	3	2	2
4	216	54	39	38	54	31	5	5	5	5	5
1	220	52	39	38	56	35	5	5	5	5	5
4	173	43	34	26	45	25	4	4	4	2	4
3	187	48	38	34	43	24	4	5	3	2	4
3	144	38	30	22	32	22	4	4	3	3	3
4	212	55	39	36	52	30	5	5	4	1	5
4	177	46	32	32	45	22	4	4	4	2	4
4	138	34	28	23	34	19	3	4	2	2	2
3	191	42	41	36	46	26	3	3	4	2	4
5	201	51	40	34	47	29	4	5	5	2	4
4	183	47	34	31	46	25	4	4	4	2	3
3	176	45	36	27	42	26	4	5	4	2	2
6	212	51	39	39	53	30	5	5	5	2	5
4	209	54	39	34	51	31	5	5	5	3	4
3	175	45	33	29	44	24	4	4	3	2	3
4	223	56	43	39	53	32	5	5	5	2	4
2	188	45	37	32	48	26	4	5	4	2	4
3	218	52	39	40	52	35	5	5	5	5	5
2	209	53	38	39	50	29	5	5	5	1	5
3	174	42	32	33	41	26	4	5	3	1	3
6	143	37	28	24	35	19	4	4	4	3	4
1	210	52	37	37	58	26	1	5	5	1	3
2	180	44	36	30	44	26	4	4	4	3	4
3	188	44	36	36	47	25	5	4	3	1	4
2	191	46	37	31	49	28	3	5	4	3	4
3	250	60	55	40	60	35	5	5	5	5	5
3	176	43	36	27	47	23	4	5	4	2	4
3	212	52	39	38	54	29	5	5	5	1	5
1	155	38	36	24	36	21	3	5	3	3	3

3	136	34	26	24	33	19	3	3	3	3	3
2	209	52	39	38	52	28	4	5	4	1	5
3	218	52	39	40	52	35	5	5	5	5	5
2	193	49	38	36	45	25	5	5	3	2	5
4	188	47	38	32	45	26	3	4	4	2	4
2	188	48	33	31	47	29	5	5	4	2	4
2	192	47	40	28	49	28	5	5	5	2	4
3	196	50	37	32	50	27	4	5	4	1	4
3	211	49	39	39	52	32	5	5	5	2	5
4	199	48	36	36	47	32	4	5	5	5	4
3	189	49	36	29	47	28	5	5	5	2	2
4	174	43	32	30	44	25	4	4	4	1	4
3	148	40	28	23	37	20	4	4	2	3	2
2	215	52	39	40	52	32	5	5	5	2	5
3	187	43	44	29	42	29	4	5	5	4	4
3	152	40	31	25	32	24	4	4	4	2	4
1	171	42	39	28	40	22	3	5	3	3	3
2	215	52	39	40	53	31	5	5	5	1	5
1	222	52	42	38	59	31	5	5	5	2	5
6	180	44	36	30	44	26	4	4	4	2	4
5	216	59	39	39	54	25	5	5	5	1	5
3	157	38	34	23	37	25	4	4	4	3	2
2	213	55	39	36	50	33	5	5	5	3	5
3	222	56	43	40	52	31	5	5	5	1	5
1	161	43	34	25	37	22	4	4	3	3	2
4	168	42	33	29	41	23	4	4	3	2	4
3	217	54	43	32	55	33	5	5	5	3	2
3	171	43	34	26	44	24	5	5	5	2	2
2	201	53	45	29	47	27	5	4	4	4	4
2	155	36	32	26	40	21	5	4	3	3	3
2	145	34	32	23	36	20	2	3	2	3	2
2	194	50	36	33	47	28	5	5	5	1	5
5	205	52	39	33	50	31	5	5	5	1	3
5	188	44	36	35	45	28	4	4	4	2	3
1	148	40	31	23	33	21	2	4	2	3	2
3	201	46	40	35	48	32	3	5	5	2	3
2	177	46	39	28	45	19	3	5	3	2	5
2	229	57	46	40	53	33	5	5	5	3	5
1	186	46	35	31	46	28	4	5	5	2	4
5	222	53	47	39	49	34	5	5	5	4	4
5	168	42	34	28	43	21	4	4	3	4	4
1	222	52	39	40	56	35	5	5	5	5	5
1	222	56	43	40	52	31	5	5	5	1	5
2	186	49	35	31	44	27	5	5	5	3	4
1	193	46	41	32	48	26	2	4	4	2	4
4	164	42	32	27	39	24	3	4	3	2	4
2	208	52	39	34	52	31	5	5	5	1	5
6	147	35	29	24	37	22	4	4	4	3	4
6	250	60	55	40	60	35	5	5	5	5	5
2	216	52	39	39	52	34	5	5	5	5	4
3	186	47	40	29	45	25	4	5	4	2	3
1	214	56	40	35	51	32	5	5	5	2	3
2	218	52	39	40	56	31	5	5	5	1	5
4	208	54	39	33	51	31	5	5	5	2	5
3	213	51	39	40	52	31	5	5	5	1	5
3	212	54	40	35	54	29	5	5	5	1	4

2	167	43	35	27	41	21	3	5	2	3	3
4	215	52	39	40	53	31	5	5	5	1	5
3	196	49	37	35	48	27	5	5	3	1	5
4	171	43	32	29	39	28	5	5	5	1	3
3	184	45	38	31	44	26	4	4	4	2	3
6	185	47	38	29	44	27	4	5	4	2	2
1	191	48	36	32	45	30	5	5	5	3	3
1	207	52	43	33	48	31	4	5	4	3	2
2	178	46	39	28	39	26	5	5	5	2	3
1	204	50	38	35	56	25	5	5	3	1	5
3	203	53	41	34	46	29	5	5	5	1	2
6	194	48	38	32	49	27	5	5	5	1	4
4	214	52	39	40	52	31	5	5	5	1	5
2	153	36	34	24	36	23	4	4	4	4	3
3	214	52	39	40	52	31	5	5	5	1	5
2	205	55	40	33	47	30	5	5	5	1	2
2	208	52	39	38	48	31	5	5	5	1	5
1	178	45	32	32	43	26	4	4	4	2	4
6	161	42	33	24	38	24	5	5	5	5	5
2	238	60	47	36	60	35	5	5	5	5	5
4	189	50	34	30	47	28	5	5	5	1	4
3	157	41	31	22	41	22	4	4	4	2	2
5	194	51	36	35	47	25	5	5	3	2	4
1	176	45	32	31	43	25	4	4	4	2	4
2	154	38	34	25	34	23	3	3	3	2	3
1	140	34	26	23	35	22	3	4	3	3	4
2	176	47	37	29	42	21	4	5	4	2	3
6	150	36	33	24	36	21	3	3	3	3	3
4	199	52	41	33	48	25	5	5	4	1	4
1	171	42	34	29	42	24	4	4	3	3	2
3	204	51	38	35	50	30	5	5	5	3	4
2	153	38	30	25	40	20	3	4	3	2	3
5	220	58	39	39	54	30	5	5	5	1	4
1	191	48	37	32	46	28	5	5	5	2	3
6	162	43	38	25	40	16	4	4	1	3	1
2	165	44	35	23	41	22	4	5	4	2	3
2	202	53	40	32	49	28	5	5	3	1	3
2	188	46	39	31	44	28	5	5	5	3	3
2	173	46	32	31	40	24	4	4	4	4	4
2	197	49	37	34	48	29	4	5	4	2	4
4	213	52	41	38	48	34	5	5	5	5	5
1	205	50	39	34	55	27	5	5	5	1	3
3	215	56	38	38	52	31	5	5	5	1	5
5	208	55	39	32	52	30	5	5	5	2	3
6	184	46	36	33	43	26	5	5	5	2	4
3	147	35	28	24	36	24	3	3	2	3	2
3	215	53	39	39	53	31	5	5	5	2	4
2	214	52	39	40	52	31	5	5	5	1	5
3	144	37	29	24	36	18	4	4	2	1	3
2	218	52	43	40	52	31	5	5	5	1	5
3	189	47	36	31	47	28	4	4	4	4	4
4	219	54	40	39	54	32	5	5	5	2	4
2	198	48	35	35	47	33	4	5	5	5	4
2	185	45	35	32	45	28	4	4	4	4	4
2	197	49	35	36	48	29	5	5	5	1	5
3	149	36	32	24	36	21	5	5	3	3	1

3	175	45	32	30	44	24	4	4	4	3	2
2	201	51	38	31	51	30	3	5	4	3	2
4	250	60	55	40	60	35	5	5	5	5	5
2	149	37	29	23	37	23	4	4	4	2	2
4	205	52	37	34	51	31	5	5	5	1	4
3	181	44	34	32	44	27	5	5	5	1	4
2	150	36	33	24	36	21	3	3	3	3	3
4	185	47	38	31	43	26	5	5	5	1	3
2	168	42	34	26	38	28	4	5	4	4	4
3	200	49	38	34	48	31	5	5	5	1	5
2	182	44	35	29	47	27	4	4	4	1	3
3	198	52	36	32	50	28	5	5	5	1	3
2	227	56	44	40	54	33	5	5	5	3	5
3	215	52	41	39	52	31	5	5	5	1	5
5	214	53	41	36	52	32	5	5	4	4	4
1	193	48	39	32	49	25	5	5	4	2	4
1	158	39	34	27	38	20	3	4	2	4	3
2	176	44	35	30	44	23	4	4	4	2	4
2	211	52	42	37	50	30	5	5	5	2	5
3	250	60	55	40	60	35	5	5	5	5	5
1	151	38	30	23	40	20	2	4	3	4	2
1	158	37	36	28	34	23	4	3	3	3	4
6	220	57	39	37	57	30	5	5	5	1	4
2	180	45	36	32	44	23	5	5	4	2	4
6	212	56	39	36	52	29	5	5	3	1	2
6	187	47	35	33	47	25	4	5	4	1	4
6	204	52	38	36	52	26	5	5	5	1	5
1	178	41	40	28	45	24	3	3	3	3	3
4	219	52	43	38	54	32	5	5	5	2	4
1	199	49	38	33	50	29	5	5	5	2	4
2	185	47	38	29	45	26	5	5	4	3	3
3	142	36	27	24	34	21	3	3	3	3	3
5	162	44	30	25	39	24	4	4	3	2	3
3	197	50	39	33	47	28	5	5	4	2	2
3	214	52	39	40	52	31	5	5	5	1	5
1	173	44	33	30	39	27	4	5	5	2	4
2	181	44	36	31	44	26	4	4	4	2	4
3	212	52	39	40	52	29	5	5	5	1	5
1	191	47	39	29	49	27	5	5	5	2	2
1	195	50	36	33	46	30	5	5	5	1	3
4	181	45	34	31	44	27	4	4	4	2	4
3	212	52	40	37	55	28	5	5	3	2	4
2	209	52	39	36	52	30	5	5	5	1	3
4	174	41	35	27	45	26	3	4	3	3	4
3	176	45	32	30	44	25	5	4	4	1	4
3	175	43	33	30	45	24	4	4	3	2	4
4	214	52	39	38	54	31	5	5	5	1	3
2	213	52	38	39	53	31	4	4	4	2	4
2	187	49	33	30	47	28	5	5	4	2	4
3	213	54	39	37	52	31	5	5	5	1	5
2	214	52	39	40	52	31	5	5	5	1	5
6	218	54	39	40	52	33	3	5	5	3	5
3	218	55	41	39	51	32	5	5	5	2	4
3	204	52	38	34	51	29	5	5	5	1	5
3	178	44	36	30	42	26	5	5	4	1	4
2	212	53	39	38	52	30	5	5	5	2	4

1	140	34	26	21	37	22	3	3	2	4	3
2	220	54	42	36	57	31	5	5	5	1	5
3	222	50	39	40	60	33	5	5	5	3	5
3	214	52	39	40	52	31	5	5	5	1	5
3	177	42	40	26	44	25	5	5	5	3	3
2	146	34	32	23	37	20	2	4	2	3	2
2	221	53	46	37	54	31	5	4	5	3	3
1	205	51	39	38	49	28	5	5	5	2	5
4	170	42	37	28	38	25	5	5	5	1	5
2	187	47	39	31	46	24	4	5	4	2	3
1	184	50	32	34	45	23	5	5	5	2	5
4	180	46	40	24	49	21	5	5	2	3	1
2	182	44	36	32	44	26	4	4	4	2	4
4	183	44	36	32	45	26	4	4	4	2	4
3	210	53	41	34	51	31	5	5	5	3	3
4	169	43	31	28	43	24	4	4	4	2	3
2	183	45	34	32	45	27	4	5	4	2	4
1	200	50	39	30	51	30	3	5	3	3	3
3	238	57	48	40	58	35	5	5	5	5	5
3	174	45	35	28	43	23	5	5	5	3	3
3	177	40	33	33	45	26	4	5	3	1	4
2	203	52	41	30	51	29	5	5	5	1	5
3	174	43	32	32	42	25	4	4	4	2	4
5	212	51	38	38	51	34	5	5	5	5	4
4	208	50	39	37	51	31	5	5	5	1	3
1	179	46	35	30	43	25	5	5	5	2	4
1	148	36	31	24	36	21	3	3	3	3	3
4	181	47	33	31	41	29	4	5	4	3	4
4	180	44	35	30	44	27	5	5	5	2	3
5	209	50	39	37	52	31	5	5	5	1	3
1	164	42	35	24	39	24	4	4	3	2	3
1	213	52	39	40	52	30	5	5	4	1	5
3	204	54	41	34	49	26	3	5	3	2	3
3	200	48	44	32	48	28	4	4	4	4	4
2	216	52	39	38	56	31	5	5	5	1	3
2	177	44	32	32	43	26	4	4	4	2	4
3	218	56	39	40	52	31	5	5	5	1	5
1	166	41	34	27	41	23	4	4	3	4	3
5	193	51	36	31	50	25	4	5	3	2	4
2	167	44	32	24	43	24	4	4	4	2	3
2	179	45	34	29	44	27	5	5	5	2	4
4	213	52	39	39	52	31	5	5	5	1	4
5	196	47	36	34	50	29	5	5	5	2	2
2	207	50	40	38	49	30	5	5	5	1	5
3	214	52	39	38	53	32	5	5	5	2	4
4	173	43	32	30	44	24	4	4	4	2	4
4	183	41	42	30	43	27	2	4	4	4	4
4	198	49	39	32	51	27	5	5	5	2	3
4	174	41	33	31	44	25	3	4	4	2	3
6	212	50	39	40	52	31	5	5	5	1	5
3	202	51	39	37	48	27	5	5	5	2	5
4	177	46	34	29	44	24	5	5	3	1	4
2	212	52	43	36	52	29	5	5	5	1	3
4	191	52	37	31	47	24	5	5	5	3	1
5	224	56	41	40	56	31	5	5	5	1	5
5	182	42	35	32	45	28	4	4	4	4	4

1	207	46	43	36	51	31	1	5	3	3	2
2	199	49	37	33	50	30	5	5	5	2	4
3	169	41	32	27	42	27	4	4	4	3	3
2	207	51	36	38	52	30	5	3	3	2	3
2	213	54	40	37	51	31	5	5	5	1	3
4	214	52	39	38	54	31	5	5	5	1	5
2	130	34	24	23	32	17	3	3	2	4	3
2	159	36	37	25	38	23	4	4	4	3	4
2	200	48	37	35	48	32	5	5	5	5	5
1	218	52	43	40	52	31	5	5	5	1	5
3	205	54	39	37	52	23	5	5	2	1	5
4	191	48	41	32	46	24	3	5	4	3	4
4	211	53	39	36	52	31	5	5	5	1	4
2	180	45	39	30	43	23	4	4	4	2	4
6	224	56	39	38	58	33	5	5	5	5	5
2	175	43	36	28	44	24	4	4	4	1	3
2	204	51	39	34	52	28	4	5	4	2	2
3	149	41	33	22	34	19	5	4	3	3	2
6	250	60	55	40	60	35	5	5	5	5	5
5	204	47	41	37	49	30	4	4	4	1	4
2	227	53	49	38	53	34	5	5	5	5	5
2	224	56	43	38	56	31	5	5	5	1	3
2	152	37	29	23	38	25	3	4	2	3	3
2	151	37	32	24	36	22	4	4	4	3	3
2	176	42	34	31	43	26	1	5	5	1	4
5	212	54	41	39	49	29	5	5	3	1	5
3	200	49	38	36	48	29	4	5	4	2	4
3	130	30	30	19	32	19	2	4	2	4	3
2	178	44	35	30	43	26	4	4	4	2	3
3	164	40	36	30	39	19	4	4	3	3	3
2	206	52	39	35	50	30	5	5	5	1	4
3	171	39	41	29	43	19	1	4	1	3	3
2	216	52	39	40	52	33	5	5	5	3	5
2	156	37	35	24	39	21	3	5	4	2	2
2	171	42	31	31	44	23	4	4	4	3	3
3	212	52	45	36	46	33	5	5	5	5	5
2	168	42	36	27	39	24	4	4	4	2	3
3	161	42	34	25	38	22	5	5	5	4	4
5	211	52	39	38	52	30	5	5	5	1	4
6	246	60	55	40	60	31	5	5	5	1	5
3	170	40	33	32	41	24	4	4	3	2	4
1	169	45	36	24	42	22	5	5	2	2	3
3	152	39	31	24	36	22	3	3	3	3	3
6	213	57	41	40	52	23	5	5	5	1	5
5	179	44	38	28	41	28	5	5	5	3	3
2	179	44	36	30	42	27	4	4	4	3	4
3	188	45	39	32	44	28	4	4	4	3	4
2	175	43	32	30	43	27	4	4	4	4	4
3	187	48	35	30	46	28	5	5	5	3	3
2	218	52	39	40	52	35	5	5	5	5	5
2	197	53	36	31	49	28	5	5	4	3	2
1	184	47	34	31	44	28	5	5	4	4	4
2	209	52	38	35	52	32	5	5	5	2	4
3	215	52	41	38	53	31	5	5	5	1	3
2	210	53	39	36	51	31	5	5	5	1	5
6	214	52	39	40	52	31	5	5	5	1	5

2	191	46	35	34	47	29	4	5	3	2	4
2	211	52	38	38	52	31	5	5	5	1	5
2	209	50	39	36	52	32	5	5	5	2	5
6	194	53	37	30	43	31	5	5	5	3	3
4	205	51	38	38	53	25	5	5	3	1	5
4	169	41	36	27	41	24	4	5	3	1	3
3	203	51	39	34	52	27	4	5	5	1	3
1	159	44	33	20	33	29	5	5	5	5	1
2	211	52	42	36	50	31	5	5	5	1	3
6	156	36	32	23	38	27	3	3	1	3	2
1	205	52	39	31	52	31	5	5	5	2	2
4	217	54	39	38	54	32	5	5	5	2	5
4	206	52	37	33	53	31	5	5	5	2	3
4	209	52	39	39	52	27	5	5	5	1	4
5	213	51	39	39	53	31	5	5	5	1	5
2	200	53	40	34	44	29	5	5	5	2	4
3	234	56	47	40	56	35	5	5	5	5	5
1	187	53	35	29	47	23	5	5	5	2	4
1	250	60	55	40	60	35	5	5	5	5	5
5	177	43	33	31	42	28	5	5	5	3	4
6	191	46	39	30	52	24	3	5	4	2	2
2	184	44	37	32	45	26	3	4	4	2	4
4	218	56	43	36	52	31	5	5	5	1	5
3	195	49	39	34	44	29	5	5	5	1	4
3	180	45	35	32	43	25	4	4	4	2	4
3	216	53	39	40	53	31	5	5	5	1	5
6	250	60	55	40	60	35	5	5	5	5	5
3	214	52	39	38	52	33	5	5	5	3	3
3	213	56	40	37	52	28	5	5	5	2	2
2	177	44	34	30	44	25	4	5	5	3	3
6	181	46	32	32	43	28	4	4	4	4	4
1	189	50	33	31	49	26	5	5	5	2	3
4	178	44	35	24	48	27	4	5	4	3	3
3	151	39	30	24	36	22	4	3	3	3	2
2	216	52	41	40	52	31	5	5	5	1	5
4	226	54	43	40	55	34	4	4	4	5	5
4	183	45	38	29	43	28	5	5	3	4	4
3	218	52	39	40	52	35	5	5	5	5	5
2	210	58	35	36	51	30	5	5	5	1	5
2	179	44	34	32	44	25	4	4	4	1	4
3	205	50	39	36	53	27	5	5	3	1	3
4	149	39	30	23	37	20	3	4	2	3	3
4	185	46	39	32	45	23	5	4	3	2	4
4	197	49	39	34	50	25	4	4	4	3	3
3	217	54	42	37	53	31	5	5	5	2	5
5	198	49	42	30	51	26	5	5	4	3	3
4	197	48	38	33	46	32	5	5	5	5	5
4	198	48	39	32	49	30	3	5	5	1	5
1	250	60	55	40	60	35	5	5	5	5	5
1	164	40	32	25	41	26	5	5	5	3	3
2	171	42	34	27	41	27	4	4	3	2	4
6	173	42	35	30	43	23	4	4	4	2	2
3	154	37	34	26	36	21	5	5	3	3	3
2	177	43	35	30	45	24	4	4	4	2	4
3	209	54	38	35	52	30	5	5	5	3	4
1	161	41	32	26	37	25	5	5	4	4	4

2	165	40	36	26	39	24	3	3	3	3	3
4	155	39	28	26	38	24	4	4	4	2	3
2	170	43	32	28	41	26	3	4	4	2	3
4	163	42	29	26	37	29	5	5	5	5	5
1	191	50	40	31	43	27	5	5	5	2	3
3	188	47	32	30	51	28	5	3	2	2	3
3	211	53	39	39	50	30	5	5	5	2	5
4	216	56	41	38	50	31	5	5	5	1	3
3	209	52	39	35	53	30	5	5	5	2	5
2	209	51	41	36	51	30	5	5	5	1	4
2	207	53	36	36	52	30	5	5	5	3	5
2	215	52	39	37	56	31	5	5	5	1	2
1	196	47	44	32	46	27	3	4	4	4	4
3	208	53	39	31	54	31	5	5	5	3	3
3	214	52	39	40	52	31	5	5	5	1	5
1	183	47	37	29	44	26	5	5	5	2	4
1	190	44	39	30	47	30	4	5	5	5	3
3	164	41	32	25	40	26	4	4	3	2	4
2	199	51	37	34	49	28	4	5	4	4	4
3	178	44	37	30	41	26	4	4	4	2	4
6	222	54	43	39	54	32	4	4	5	4	5
2	157	42	31	22	38	24	4	4	3	3	2
3	208	49	39	38	51	31	5	5	5	1	5
1	199	52	38	32	49	28	5	5	5	2	3
2	202	49	44	32	48	29	4	4	4	4	4
1	149	39	31	22	33	24	4	4	4	3	3
1	250	60	55	40	60	35	5	5	5	5	5
3	205	53	36	37	49	30	5	5	5	2	4
4	203	53	40	34	48	28	5	5	5	3	3
3	171	43	36	30	40	22	5	5	5	1	4
2	191	48	36	32	47	28	4	4	4	4	4
2	175	45	33	30	42	25	4	4	4	2	4
4	211	54	39	36	51	31	5	5	5	2	3
1	170	42	32	31	40	25	4	4	4	2	4
6	217	52	39	40	55	31	5	5	5	1	5
2	184	46	34	31	44	29	5	5	5	2	5
6	214	52	39	40	54	29	5	5	5	1	5
3	175	44	34	29	45	23	4	5	3	3	3
1	213	52	39	39	52	31	5	5	5	1	4
2	171	44	34	29	40	24	4	5	4	3	3
4	207	54	37	36	50	30	5	5	5	2	5
3	202	50	38	36	48	30	4	4	4	2	4
6	224	56	39	40	56	33	5	5	5	3	5
2	183	45	35	28	47	28	4	4	4	4	4
3	196	50	37	34	47	28	5	5	4	2	4
3	188	47	36	33	49	23	4	5	2	1	4
3	173	43	32	30	44	24	4	4	4	2	3
2	150	38	34	22	34	22	4	4	3	4	3
2	217	54	44	35	55	29	5	5	5	2	4
5	213	51	49	34	49	30	5	5	5	5	5
1	142	36	30	23	32	21	3	3	3	3	3
3	166	41	33	27	42	23	4	4	4	2	3
3	202	54	37	30	50	31	5	5	5	1	5
3	166	37	34	23	47	25	4	4	3	4	2
2	206	50	41	32	52	31	5	5	5	1	1
4	188	52	38	29	46	23	5	5	5	2	4

5	215	53	39	36	56	31	5	5	5	1	3
2	178	44	34	30	43	27	4	4	4	2	4
2	155	39	33	24	36	23	3	3	4	4	3
4	221	57	39	39	55	31	4	5	4	2	4
4	175	39	37	31	42	26	4	5	5	1	4
2	219	57	41	38	55	28	5	5	5	2	3
3	193	48	37	31	52	25	5	5	5	2	3
3	193	50	36	29	51	27	5	5	5	2	1
2	207	51	39	35	52	30	5	5	5	2	4
6	208	50	37	36	51	34	5	5	5	5	5
2	182	43	36	32	44	27	3	4	4	1	3
4	207	51	36	36	53	31	5	5	5	1	4
1	174	44	35	26	43	26	4	4	3	3	3
3	218	56	39	38	52	33	5	5	5	3	5
2	219	53	43	38	57	28	2	3	3	2	3
4	207	50	38	37	51	31	4	5	5	2	4
3	171	41	33	31	41	25	3	5	4	2	4
5	182	45	36	29	45	27	4	5	4	2	3
3	196	50	37	33	50	26	5	5	3	4	3
2	179	43	35	31	44	26	3	3	3	3	3
2	250	60	55	40	60	35	5	5	5	5	5
2	219	52	43	40	52	32	5	5	5	2	5
2	250	60	55	40	60	35	5	5	5	5	5
1	207	52	39	35	51	30	5	5	5	1	3
2	213	56	39	35	53	30	5	5	5	1	4
3	212	52	37	40	52	31	5	5	5	1	5
5	210	52	39	36	52	31	5	5	5	1	2
4	170	43	33	27	41	26	3	4	4	2	3
3	212	52	39	38	52	31	5	5	5	1	3
5	186	46	40	32	43	25	5	5	5	2	4
6	213	51	42	37	52	31	5	5	5	1	4
1	154	39	34	20	38	23	5	5	5	3	3
4	170	43	33	27	43	24	4	4	4	3	3
4	152	40	31	26	36	19	4	4	4	3	3
1	159	44	29	22	40	24	4	4	4	3	3
3	170	45	34	29	38	24	4	5	4	2	5
2	147	38	31	22	35	21	3	3	3	3	2
4	210	52	39	40	52	27	5	5	5	1	5
1	175	42	34	29	45	25	4	4	4	2	3
3	200	48	44	32	48	28	4	4	4	4	4
2	214	52	39	40	52	31	5	5	5	1	5
2	183	44	39	32	42	26	4	4	4	2	4
2	192	52	37	29	47	27	5	5	5	2	3
4	178	42	37	32	42	25	4	5	4	1	4
4	141	35	30	18	37	21	4	4	2	4	3
3	174	44	34	29	42	25	4	4	4	3	4
4	216	53	39	39	54	31	5	5	5	1	5
4	163	36	31	27	41	28	3	4	3	3	3
1	226	54	43	40	56	33	5	5	5	3	5
3	250	60	55	40	60	35	5	5	5	5	5
1	181	44	35	31	45	26	4	4	4	2	3
4	168	45	33	28	39	23	4	5	3	2	4
1	149	36	31	26	36	20	3	3	3	3	3
2	175	43	32	30	45	25	4	4	4	1	4
3	155	38	35	21	39	22	4	4	3	4	3
3	145	36	29	22	37	21	3	3	3	3	3

4	250	60	55	40	60	35	5	5	5	5	5
3	212	52	39	38	52	31	5	5	5	1	3
3	205	51	39	34	52	29	5	5	3	1	4
4	226	57	43	39	57	30	5	5	5	2	5
3	193	47	38	33	45	30	5	5	5	5	5
4	188	48	36	31	47	26	5	5	3	3	3
1	165	41	35	26	40	23	4	4	4	2	2
1	162	41	34	28	38	21	3	4	4	3	4
2	150	36	33	24	36	21	3	3	3	3	3
2	192	52	38	32	45	25	4	5	3	2	4
2	205	51	39	35	50	30	5	5	5	1	3
1	185	45	40	28	45	27	4	4	4	4	4
4	179	46	40	27	40	26	4	5	3	3	3
2	166	41	34	25	42	24	3	4	3	3	2
2	214	56	38	36	54	30	5	5	5	1	5
4	155	33	32	28	39	23	3	3	3	4	2
3	180	47	33	29	43	28	5	4	5	3	3
2	234	57	52	39	54	32	5	5	5	2	4
3	180	45	33	30	44	28	4	4	4	2	3
4	144	34	29	24	34	23	5	5	5	3	3
3	182	43	37	31	45	26	4	4	4	2	4
2	192	50	39	28	46	29	5	5	5	1	3
5	216	53	39	40	53	31	5	5	5	1	5
2	214	52	39	40	52	31	5	5	5	1	5
6	191	48	36	32	47	28	4	4	4	4	4
2	155	35	34	24	39	23	3	4	4	2	4
4	149	40	28	24	37	20	4	4	3	3	4
4	186	50	37	31	43	25	5	5	4	2	4
2	209	52	39	37	50	31	5	5	5	1	5
1	177	43	35	30	44	25	3	5	3	2	3
2	189	48	37	31	45	28	5	5	5	1	4
3	201	53	39	31	49	29	5	5	5	1	1
2	174	45	33	27	41	28	5	5	5	3	4
1	170	40	34	29	41	26	3	4	4	2	3
1	210	48	39	40	52	31	1	5	5	1	5
2	183	46	37	30	44	26	5	5	5	2	4
3	164	40	35	26	41	22	4	5	3	2	3
3	163	41	33	26	39	24	4	5	4	1	4
3	214	52	39	40	52	31	5	5	5	1	5
3	208	51	39	33	52	33	5	5	5	3	4
2	202	51	41	32	48	30	5	5	5	2	4
2	166	39	33	28	40	26	4	5	4	2	3
1	201	50	38	33	51	29	4	5	5	2	4
3	185	47	39	31	46	22	5	5	4	2	3
2	216	52	39	40	52	33	5	5	5	3	5
5	174	44	34	32	40	24	4	4	3	2	3
4	173	42	32	29	44	26	4	4	4	3	4
2	182	43	35	31	47	26	4	4	4	2	4
2	217	55	43	36	50	33	5	5	5	5	5
4	205	48	39	35	52	31	3	5	3	4	2
1	235	60	48	40	52	35	5	5	5	5	5
2	234	60	43	40	60	31	5	5	5	1	5
2	182	47	36	29	46	24	5	5	4	3	3
1	218	52	39	40	56	31	5	5	5	1	5
3	183	46	37	30	44	26	4	4	4	2	4
4	189	48	40	31	43	27	5	5	5	1	2

1	235	58	48	38	60	31	3	5	5	1	3
1	230	60	47	36	56	31	5	5	5	1	1
2	214	55	41	36	52	30	5	5	5	1	4
2	206	53	39	35	50	29	5	5	5	1	4
3	158	37	30	27	41	23	3	3	3	3	3
3	184	45	39	30	44	26	5	5	5	2	3
4	173	43	38	25	43	24	4	5	3	3	2
2	186	45	38	31	45	27	3	5	3	2	3
3	141	35	27	24	33	22	3	3	3	2	3
2	209	54	39	35	51	30	5	5	5	2	4
1	212	52	39	36	54	31	5	5	5	2	5
6	171	41	37	28	41	24	5	5	4	2	3
2	214	52	39	40	52	31	5	5	5	1	5
2	213	52	39	39	52	31	5	5	5	1	5
1	179	45	36	30	43	25	4	4	3	2	4
2	191	49	36	33	44	29	5	5	5	2	5
1	204	51	42	32	47	32	5	5	5	3	5
3	184	45	38	31	42	28	3	5	3	2	3
3	206	51	45	32	50	28	4	4	4	1	3
3	213	57	39	35	54	28	5	5	5	1	3
6	222	52	43	40	52	35	5	5	5	5	5
4	152	35	34	21	41	21	4	5	4	1	3
1	163	40	35	25	39	24	4	4	4	4	3
1	176	42	34	32	42	26	4	4	4	2	4
3	184	44	39	31	45	25	4	5	4	2	4
1	150	36	33	24	36	21	3	3	3	3	3
6	216	52	39	40	53	32	5	5	5	2	5
4	213	51	39	40	52	31	4	5	5	1	5
4	220	54	44	40	51	31	5	5	5	1	5
1	127	31	34	14	32	16	3	5	5	3	2
4	210	51	39	37	52	31	5	5	5	1	4
4	214	52	39	38	54	31	5	5	5	1	3
2	197	48	39	35	48	27	5	5	4	1	5
2	199	51	38	35	50	25	4	5	4	1	4
4	208	53	41	30	56	28	5	5	5	2	3
4	176	40	35	29	45	27	2	5	4	2	4
3	156	38	34	25	36	23	4	4	4	4	4
2	182	46	35	29	47	25	4	5	5	3	3
3	168	42	37	27	37	25	4	5	4	2	4
3	202	46	46	32	49	29	4	4	5	4	3
2	182	46	37	29	42	28	5	5	5	1	3
3	213	55	38	36	52	32	5	5	5	4	3
5	217	52	43	40	51	31	5	5	5	1	5
6	169	45	34	25	40	25	5	5	5	1	3
2	218	52	43	40	52	31	5	5	5	1	5
3	148	37	33	23	34	21	5	5	5	2	3
3	181	44	34	32	44	27	3	5	5	1	4
1	250	60	55	40	60	35	5	5	5	5	5
4	233	60	47	36	58	32	5	5	5	2	3
2	183	44	38	27	47	27	4	4	4	3	4
1	152	39	32	22	34	25	3	4	2	3	2
3	212	51	39	38	52	32	4	5	5	2	4
2	180	44	36	31	43	26	4	4	4	2	4
2	182	45	36	30	43	28	4	4	4	4	4
2	158	40	30	25	41	22	4	4	4	2	3
3	179	43	35	31	44	26	4	4	4	2	3

2	195	49	38	33	45	30	5	5	5	1	3
2	186	45	38	29	48	26	5	5	4	2	3
3	184	46	37	32	45	24	3	4	4	3	4
1	250	60	55	40	60	35	5	5	5	5	5
3	160	43	33	24	37	23	5	4	4	2	3
3	217	52	38	40	52	35	5	5	5	5	5
2	180	43	38	29	43	27	4	5	5	3	3
5	177	45	35	31	41	25	4	5	4	2	4
4	214	56	39	36	48	35	5	5	5	5	5
1	210	53	41	33	51	32	5	5	5	2	2
4	211	51	39	37	52	32	4	5	5	2	4
3	199	51	38	35	48	27	5	5	5	1	4
2	166	41	32	24	43	26	3	4	4	2	2
2	216	54	39	38	52	33	5	5	5	3	3
4	212	52	39	38	53	30	5	5	4	2	5
2	174	45	31	31	43	24	4	4	2	4	2
2	167	44	35	24	36	28	5	5	5	3	1
2	175	44	35	29	44	23	4	4	4	2	3
5	188	53	34	31	43	27	4	4	4	2	4
2	194	49	38	32	47	28	4	5	4	2	3
3	169	43	33	27	41	25	4	4	4	3	3
3	205	48	43	36	49	29	5	5	4	1	3
3	210	54	38	37	52	29	5	5	5	1	3
4	162	41	33	24	39	25	3	4	4	2	2
2	172	42	34	30	43	23	4	4	4	2	4
1	218	52	43	40	52	31	5	5	5	1	5
3	221	55	51	34	52	29	5	4	5	4	5
4	205	50	38	34	54	29	5	5	5	2	4
2	210	51	41	35	54	29	4	5	5	2	2
1	211	52	39	36	52	32	5	5	5	3	3
2	202	49	43	32	49	29	4	5	4	2	4
2	186	46	38	30	44	28	5	5	4	1	3
2	214	52	39	40	52	31	5	5	5	1	5
3	196	53	38	30	49	26	5	5	3	2	3
1	174	44	37	28	42	23	4	4	3	3	3
2	204	54	39	33	53	25	5	5	4	2	3
2	213	54	45	33	52	29	5	5	3	1	1
2	171	46	34	26	40	25	4	4	4	2	4
3	228	52	49	40	52	35	5	5	5	5	5
3	206	52	39	36	52	27	5	5	5	1	3
4	173	44	32	30	43	24	4	4	4	2	4
3	220	55	38	39	55	33	5	5	5	4	4
5	169	41	31	31	41	25	3	3	3	2	4
2	202	51	39	34	50	28	5	5	5	3	5
3	218	52	43	40	52	31	5	5	5	1	5
2	173	44	33	29	42	25	5	5	4	2	3
4	178	45	35	27	45	26	4	5	4	4	3
2	173	42	35	28	43	25	4	4	3	3	3
3	186	47	33	33	45	28	5	5	5	2	5

e6	e7	e8	e9	e10	e11	e12	e13	e14	e15	e16	e17	e18	e19
5	5	1	1	5	5	5	5	5	5	5	1	5	5
4	4	1	2	4	4	5	4	5	3	4	1	4	3
5	5	1	1	5	5	5	5	5	5	5	5	5	3
4	3	1	1	5	3	3	3	3	4	4	4	3	3
5	4	1	1	4	4	4	5	3	4	4	2	4	3
5	5	1	1	5	3	4	5	5	5	5	3	5	3
5	5	5	5	5	5	5	5	5	5	5	5	5	5
4	3	1	1	4	4	4	4	4	4	4	3	4	2
3	4	2	3	3	3	3	3	3	3	3	5	3	3
5	5	1	3	5	5	5	5	5	5	5	5	5	5
5	4	1	1	4	4	5	5	5	5	5	1	5	4
5	3	1	1	4	5	5	5	3	3	3	2	5	3
5	5	1	1	5	5	5	5	5	5	5	1	5	5
4	4	2	2	4	2	4	4	4	4	4	3	4	3
5	5	1	1	4	5	5	5	5	5	5	1	5	5
5	4	4	2	5	2	5	5	4	5	5	5	5	4
5	3	1	1	5	5	5	5	4	5	5	2	5	5
4	4	1	1	5	5	5	5	5	5	5	1	5	5
5	3	1	1	5	5	5	5	5	5	5	1	5	3
4	3	1	1	3	3	5	5	3	4	4	2	4	3
5	4	1	1	4	5	5	5	5	5	5	5	5	4
5	5	5	5	5	5	5	5	5	5	5	5	5	5
5	5	3	3	3	3	3	3	3	1	3	3	3	1
5	4	1	1	5	5	4	5	5	5	5	3	5	4
4	4	1	1	4	4	5	4	3	4	3	2	4	3
5	4	1	1	4	4	4	5	5	5	5	2	5	5
4	3	2	4	5	4	4	4	3	5	4	3	4	4
5	3	1	1	3	4	5	4	4	5	4	2	4	5
4	4	1	1	4	4	4	5	4	4	4	3	4	3
5	3	1	1	4	5	5	5	5	5	5	2	5	3
4	4	1	1	4	3	3	4	4	4	4	3	4	4
5	5	1	1	5	5	5	5	5	5	5	1	5	5
4	3	2	2	3	2	4	4	3	4	4	5	4	4
4	2	1	2	4	4	4	4	4	4	4	2	3	4
5	1	1	5	5	5	5	5	4	5	4	2	4	5
5	5	1	1	5	5	4	5	5	5	5	1	5	3
4	3	2	2	5	3	3	4	4	4	4	4	4	4
3	3	2	2	3	3	3	3	3	3	3	3	3	3
5	3	1	1	3	4	5	5	4	4	5	3	4	4
5	3	1	1	4	5	5	4	4	5	4	3	4	5
4	3	1	1	4	3	4	4	4	4	4	3	4	3
4	3	3	3	3	3	3	3	3	3	3	3	3	3
4	3	1	1	3	2	4	4	4	5	4	3	4	3
5	5	1	1	5	5	5	5	5	5	5	5	5	5
4	3	1	1	4	3	4	5	4	4	4	3	4	3
5	4	1	1	4	4	5	5	4	5	4	2	5	3
5	5	1	1	5	5	5	5	5	5	5	1	5	4
5	5	5	5	5	5	5	5	5	5	5	5	5	5
4	4	2	2	3	3	3	4	4	4	4	1	3	4
5	5	1	1	5	5	5	5	5	5	5	5	5	4

5	5	1	1	5	5	5	5	5	5	5	1	5	5
3	2	2	4	3	4	2	3	2	4	4	2	3	4
5	5	1	1	4	5	5	5	5	5	5	1	5	3
5	4	1	1	5	5	5	5	5	5	5	2	4	3
4	3	1	1	4	5	5	4	4	4	4	1	4	2
4	3	1	1	4	4	4	4	4	4	4	3	4	4
4	4	1	2	4	5	5	5	4	5	5	4	5	2
5	3	2	3	4	4	4	5	3	3	4	3	4	3
5	4	1	1	3	4	5	5	4	4	4	1	4	4
4	3	1	1	3	4	4	4	4	5	4	4	4	3
5	3	1	1	4	4	5	4	3	5	4	2	4	3
5	4	1	1	5	5	5	5	4	5	5	2	5	5
5	3	1	1	5	5	5	5	5	5	4	1	4	4
5	5	4	1	5	5	5	5	5	5	5	1	5	5
4	3	1	2	5	4	4	4	4	2	4	3	4	1
4	4	1	4	4	4	4	4	4	4	4	3	4	4
4	4	1	1	4	5	5	5	5	5	5	2	4	5
4	4	1	2	4	4	4	4	4	4	4	2	4	4
4	2	1	2	3	4	5	3	4	5	4	3	3	2
3	3	1	1	4	3	3	3	3	5	4	2	3	3
4	3	2	2	5	4	4	4	4	4	4	2	4	4
4	3	1	1	5	4	4	4	4	5	4	1	4	3
5	5	1	5	5	5	5	5	5	5	5	1	5	5
5	5	1	1	5	5	5	5	5	5	5	1	5	5
4	4	1	1	3	3	3	4	4	4	4	1	4	3
3	3	1	1	1	4	3	4	3	4	3	1	3	3
5	4	1	1	3	5	5	5	5	4	5	3	4	4
5	4	1	3	4	4	4	4	4	5	4	4	5	4
5	3	1	1	4	4	5	5	5	5	5	1	5	4
4	3	2	3	3	4	4	4	4	4	4	2	4	4
5	3	1	1	4	5	5	4	4	4	4	3	4	4
5	5	1	1	4	5	5	5	5	5	5	2	5	3
4	4	1	2	4	4	4	5	3	5	5	3	4	4
5	5	1	1	3	5	5	3	4	5	5	2	5	3
5	3	1	1	5	5	5	5	5	5	5	1	5	5
3	3	3	3	3	3	3	3	3	3	3	3	3	3
5	5	1	1	5	5	5	5	5	5	5	5	5	5
5	3	1	3	4	5	5	4	4	5	5	2	4	3
4	4	4	4	4	4	4	4	4	4	4	4	4	4
5	5	5	5	5	5	5	5	5	5	5	5	5	5
4	3	1	1	3	4	4	4	3	5	4	3	4	3
5	3	1	1	5	5	5	5	5	5	5	1	5	3
4	4	1	3	4	4	4	4	4	4	4	3	4	4
5	5	3	3	5	5	5	5	5	5	5	1	5	5
5	5	5	5	5	5	5	5	5	5	5	1	5	5
5	3	1	1	5	5	5	5	5	5	5	1	5	5
3	3	1	1	4	5	4	4	4	5	3	2	3	2
3	3	1	3	4	4	4	4	4	4	4	1	4	4
5	3	1	1	5	5	5	5	4	5	5	5	5	4
3	3	2	2	3	3	3	3	3	3	3	2	3	3
4	4	1	1	4	4	4	3	4	4	4	1	4	4
4	4	1	1	5	4	4	4	5	5	5	1	5	5
5	3	1	1	4	4	4	4	4	4	4	2	5	4
5	5	1	1	5	5	5	5	5	5	5	3	5	5
5	3	1	1	5	5	5	4	4	5	4	3	4	4
5	4	1	1	5	5	5	5	5	5	5	1	5	5

5	5	1	5	5	5	5	5	5	5	5	1	5	5
3	2	1	1	2	4	4	4	3	3	3	2	3	3
4	2	1	1	3	3	5	4	2	4	4	2	5	3
5	5	1	1	5	5	5	5	5	5	5	1	5	5
4	3	1	2	4	4	4	4	3	4	4	2	4	4
5	5	1	1	5	5	5	5	5	5	5	5	5	5
5	5	1	1	5	5	5	5	5	5	5	1	5	5
5	5	1	1	5	5	5	5	4	5	5	2	5	5
3	5	1	2	5	4	5	5	5	5	5	1	5	5
5	3	1	1	5	5	5	4	5	5	5	3	5	4
5	5	1	1	5	5	5	5	5	5	5	1	5	5
4	2	1	1	3	4	4	4	4	4	4	4	4	4
4	4	2	4	4	4	4	4	4	4	4	1	3	4
5	4	1	1	5	5	4	5	1	5	5	3	5	5
3	3	1	3	3	3	3	3	4	4	4	2	3	3
5	2	1	1	5	5	5	4	4	5	4	3	4	5
5	5	5	5	5	5	5	5	5	5	5	5	5	5
5	4	1	1	5	3	4	4	4	4	4	2	4	3
5	5	5	5	5	5	5	5	5	5	5	5	5	5
5	2	1	1	5	5	5	5	5	5	5	2	5	2
4	3	1	1	4	4	3	4	4	4	3	2	4	3
4	2	2	3	3	2	3	3	4	3	4	4	3	3
4	3	1	1	4	4	5	4	4	5	4	2	4	4
4	3	1	1	4	4	4	4	3	3	4	2	4	3
5	4	1	1	4	5	5	5	4	5	5	1	5	3
4	4	1	2	4	4	4	4	4	4	4	1	4	4
5	3	1	1	5	5	5	5	5	5	5	2	5	5
4	3	1	1	4	4	4	4	4	4	4	1	4	3
5	5	1	1	5	5	5	1	5	5	5	3	5	5
4	2	1	1	1	4	5	5	4	5	5	2	4	1
3	3	2	2	3	3	3	3	3	3	4	2	3	3
4	2	1	1	5	5	5	4	3	4	4	1	4	3
4	2	1	1	3	2	3	4	3	3	3	3	3	4
3	3	1	1	1	3	4	3	3	3	3	1	3	2
5	5	1	1	4	5	5	5	5	5	5	2	5	5
5	5	1	3	4	5	5	5	4	4	4	3	5	4
4	3	1	1	3	3	5	4	5	5	5	4	3	3
5	3	1	1	4	5	5	5	5	5	4	1	5	3
5	5	1	1	5	5	5	5	5	5	5	5	5	5
5	1	1	1	1	1	1	1	1	5	5	1	5	1
4	3	1	3	3	3	3	3	3	3	3	3	3	3
4	2	1	4	3	5	5	4	3	5	5	4	5	4
5	2	1	2	4	5	5	4	4	5	4	2	5	5
5	3	1	1	4	5	4	4	4	5	4	2	5	3
3	3	3	3	3	3	3	3	3	3	3	3	3	3
4	3	1	1	3	4	4	4	4	4	4	5	4	4
5	1	1	1	3	5	5	4	3	5	3	4	5	5
5	3	1	1	3	3	4	4	4	4	4	2	4	3
5	5	1	1	5	5	5	5	5	5	5	1	5	5
5	5	5	5	5	5	5	5	5	5	5	5	5	5
5	3	1	1	5	5	5	5	5	5	5	5	5	4
5	4	1	1	5	5	4	5	5	5	5	2	5	5
4	4	1	4	4	4	4	4	4	4	4	1	4	4
5	2	1	1	5	5	5	5	5	5	5	2	5	5
5	2	1	5	3	4	3	5	3	5	4	1	5	4
3	3	3	3	3	4	4	4	4	4	4	4	4	4

5	5	1	1	5	5	5	5	5	5	5	1	5	5
5	5	1	1	5	5	5	5	5	5	5	1	5	4
5	4	1	1	4	4	4	5	4	5	4	1	5	3
4	3	4	4	5	4	4	4	4	3	4	3	4	5
3	3	1	1	5	5	5	5	5	5	5	2	5	5
5	3	1	1	4	5	5	5	5	5	5	3	5	5
4	5	1	1	5	5	5	5	4	5	5	1	4	4
4	4	4	4	4	4	4	4	4	4	4	4	4	4
4	2	1	1	4	4	4	4	3	4	3	2	4	3
5	5	1	1	5	5	5	5	5	5	5	5	5	5
5	5	1	1	5	5	5	5	5	5	5	1	5	5
4	3	1	1	3	5	4	4	5	5	5	3	4	3
4	1	1	4	1	3	5	4	1	5	3	3	4	2
4	3	1	1	3	5	5	5	5	5	5	1	4	4
4	4	1	1	5	5	5	5	5	5	5	2	5	3
5	5	5	5	5	5	5	5	5	5	5	5	5	5
5	5	1	1	5	5	5	5	5	5	5	1	5	5
3	3	3	3	3	3	3	3	3	3	3	3	3	3
5	3	1	1	4	5	4	5	5	5	5	1	5	4
5	4	1	1	3	4	5	5	4	4	5	1	5	4
3	4	3	4	5	4	4	4	4	5	5	4	5	4
5	2	1	2	1	5	5	5	2	5	5	1	5	3
4	3	1	1	4	4	5	4	4	4	5	3	4	2
3	3	3	3	3	3	3	3	3	3	3	3	3	3
4	4	2	2	4	4	4	4	4	4	4	4	4	3
4	4	2	2	3	4	4	4	4	4	4	3	4	4
5	5	1	1	5	5	5	5	5	5	5	1	5	5
4	5	2	3	4	4	5	4	4	5	4	2	4	5
5	5	1	1	4	5	5	5	5	5	5	1	5	5
5	4	1	1	1	5	5	5	4	5	5	5	5	3
4	3	1	1	5	4	5	4	4	5	4	2	4	5
5	5	1	1	5	5	5	5	5	5	5	1	5	5
5	5	3	3	5	5	5	5	5	5	5	1	5	5
5	5	1	1	5	5	5	5	5	5	5	1	5	5
4	4	1	1	3	4	5	5	5	4	4	1	4	4
4	4	1	3	4	4	5	4	4	4	4	2	4	4
3	3	1	1	3	3	3	3	3	3	3	5	3	3
5	5	1	1	3	5	4	5	5	5	5	3	5	5
4	3	1	1	3	4	4	4	4	4	4	3	5	4
5	4	1	2	4	4	4	3	4	4	4	3	4	4
3	3	3	3	3	3	3	3	3	3	3	3	3	3
5	5	1	1	5	5	5	5	5	5	5	1	5	5
5	4	2	2	5	5	5	5	4	4	5	5	5	5
5	5	1	2	4	4	5	5	4	4	4	3	4	5
4	4	2	2	4	4	4	4	3	4	4	2	4	3
4	4	1	1	4	4	4	4	4	4	4	1	4	4
4	4	1	4	4	4	4	4	4	4	4	4	4	4
5	4	1	2	4	4	4	4	4	4	4	3	3	3
4	4	1	1	4	4	4	4	4	5	4	2	3	3
4	4	1	1	4	4	4	5	4	4	4	2	4	4
5	5	1	1	4	4	5	5	5	5	5	1	5	5
5	5	1	1	5	5	5	5	5	5	5	1	5	5
5	5	1	2	5	5	5	5	5	5	5	3	5	4
3	3	1	2	3	4	4	4	4	4	4	4	4	3
4	4	1	1	4	4	4	4	4	4	4	1	4	4
5	5	1	1	4	5	5	5	5	5	5	2	5	3

4	4	1	1	4	4	4	4	4	4	4	1	4	4
5	3	1	3	3	5	5	4	3	5	4	3	4	3
5	5	1	3	5	5	5	5	5	5	5	5	5	5
5	4	1	1	3	4	4	4	3	3	4	3	4	2
5	5	2	2	5	5	5	5	5	5	5	3	5	5
5	5	1	1	5	5	5	5	1	5	5	1	5	5
5	3	1	1	3	5	4	4	4	5	4	2	5	4
5	5	1	1	5	5	5	4	5	5	5	3	5	5
5	4	1	1	5	5	4	5	5	5	5	2	5	3
3	3	3	2	3	3	3	3	3	3	3	3	3	4
5	5	1	2	5	5	5	4	4	4	4	2	5	4
4	4	1	3	4	4	4	4	4	4	4	1	4	4
4	3	1	3	4	4	4	4	4	5	5	2	4	4
5	3	1	1	3	4	4	5	4	5	4	3	4	4
5	4	1	1	3	5	5	5	5	5	5	1	5	3
5	5	1	1	5	5	5	5	5	5	5	1	5	5
5	5	1	5	5	5	5	5	5	5	5	1	5	5
5	5	1	1	5	5	5	5	5	5	5	3	5	5
5	4	1	4	4	4	4	3	4	5	4	1	5	4
5	5	1	1	4	4	5	5	5	5	5	1	5	5
5	5	1	1	5	5	5	5	5	3	4	2	5	3
4	4	1	1	4	4	5	4	4	3	3	3	4	2
5	5	1	1	5	5	5	5	5	5	5	1	5	5
5	3	1	3	5	5	5	5	5	5	5	1	5	5
5	5	1	1	4	5	5	5	5	5	5	2	5	5
4	3	1	1	4	4	4	4	4	4	4	1	4	4
5	4	1	1	4	5	5	5	5	5	5	1	5	5
4	3	4	3	3	4	4	3	3	4	4	3	3	2
5	4	1	1	5	5	5	5	5	5	5	3	4	3
4	3	1	2	4	4	4	4	3	5	5	1	4	4
4	3	1	1	5	5	4	5	5	5	4	2	4	5
5	5	1	1	4	5	5	5	5	5	5	1	5	5
5	2	1	1	5	5	5	5	5	5	5	1	5	5
5	4	1	2	4	4	4	4	4	5	5	1	4	3
5	4	1	1	5	5	5	5	5	5	5	1	5	4
5	5	1	5	5	5	5	5	5	5	5	5	5	5
5	3	1	1	5	5	4	4	4	5	5	3	4	4
5	5	1	1	5	5	5	5	5	5	5	2	5	5
5	3	1	5	3	4	5	5	4	5	5	3	5	4
5	5	1	1	4	5	5	5	5	5	5	2	5	4
5	4	1	1	4	5	5	5	5	5	5	4	5	2
5	5	1	1	5	5	5	5	5	5	5	1	5	5
5	5	1	1	5	5	5	5	5	5	5	1	5	5
5	3	1	1	4	4	4	4	4	4	4	3	5	4
4	4	1	1	4	4	4	4	4	4	4	1	4	4
5	5	1	1	5	5	5	5	5	5	5	2	5	3
5	5	1	1	5	5	5	5	5	4	4	1	5	1
4	2	1	2	4	4	4	4	3	4	4	3	4	2
4	3	1	2	3	3	5	3	4	4	3	2	4	3
5	4	2	1	5	5	4	4	3	5	5	2	3	5
5	4	2	3	4	5	5	5	4	5	5	3	4	3
4	2	2	2	4	4	4	4	4	4	4	2	4	4
5	3	1	1	5	5	5	5	5	5	5	1	5	4
5	3	1	1	5	5	5	5	4	5	5	2	5	4
5	5	1	1	4	4	4	4	4	4	4	3	4	4
3	2	2	3	3	3	3	3	3	3	3	2	3	3

5	5	1	2	5	5	5	5	5	5	5	1	5	5
4	4	1	4	5	5	5	5	5	5	5	2	5	5
5	4	1	1	5	5	5	3	5	5	5	1	5	5
5	5	1	1	4	4	4	5	5	5	5	2	5	5
5	5	1	1	5	5	5	5	5	5	5	1	5	5
4	4	2	2	4	4	4	4	4	4	4	2	4	4
5	5	5	5	5	5	5	5	4	5	5	5	5	5
5	3	1	1	2	4	5	4	3	5	4	4	5	5
4	3	2	2	3	4	4	4	3	3	3	2	3	3
5	5	1	1	5	5	5	5	5	5	5	1	5	5
4	3	1	2	3	4	4	4	4	4	4	2	3	4
4	4	1	1	4	4	4	4	4	4	4	2	4	4
2	2	2	2	2	2	2	4	1	4	2	5	5	2
4	5	1	1	5	5	5	4	4	5	5	3	4	3
4	4	4	4	4	4	4	4	4	5	4	4	5	4
5	5	5	5	5	5	5	5	5	5	5	5	5	5
5	4	1	5	5	5	5	5	5	5	5	3	5	5
3	3	3	3	3	3	3	3	3	3	3	1	3	3
4	3	2	2	3	3	4	4	3	3	3	3	4	2
4	4	1	1	4	4	4	5	5	4	4	1	5	4
4	4	1	1	4	4	4	4	4	4	4	4	4	3
5	3	1	3	3	3	3	3	5	5	5	5	5	5
5	3	1	1	4	4	4	5	4	4	4	2	5	4
5	1	1	1	5	5	5	5	5	4	5	5	5	5
4	5	1	1	4	4	5	4	2	4	5	2	5	4
5	3	1	1	5	5	5	5	5	5	5	1	5	5
5	4	1	1	4	4	4	5	2	5	4	2	4	4
5	4	1	1	5	5	5	5	4	4	4	2	5	5
4	4	1	1	4	4	4	4	4	4	4	1	4	4
5	5	1	1	5	5	5	5	5	5	5	1	5	5
5	4	1	1	4	4	5	5	5	5	5	2	5	5
4	4	1	1	4	4	5	4	4	4	4	3	4	4
5	3	1	1	5	5	5	5	5	5	5	1	5	5
4	2	2	2	3	4	4	3	3	3	3	2	3	2
5	5	1	1	5	5	5	5	5	5	5	1	5	5
5	5	1	1	5	5	5	5	5	5	5	1	5	5
4	5	1	1	5	5	5	5	5	5	5	2	5	5
5	3	1	2	4	5	5	5	4	5	4	1	5	3
5	3	1	3	4	5	5	5	4	4	4	2	5	4
5	3	1	3	4	5	5	5	4	4	4	2	5	4
5	3	1	2	4	4	5	4	4	5	4	1	4	3
5	5	1	1	5	5	5	5	5	5	5	1	5	5
5	4	1	1	3	4	4	4	4	4	4	1	4	3
3	3	1	2	4	4	4	4	3	5	5	1	4	4
5	5	1	5	5	5	5	5	4	5	5	3	5	5
5	5	1	1	5	5	5	5	5	5	5	1	5	5
5	5	1	5	5	5	5	5	5	5	5	1	5	5
5	5	1	1	5	5	5	5	5	5	5	2	5	4
5	5	5	5	5	5	5	5	5	5	5	2	5	5
5	4	1	1	4	4	5	4	4	5	5	1	5	3
4	2	1	1	3	5	5	3	5	4	5	3	5	3
3	5	4	4	4	4	4	1	1	4	1	3	1	3
4	3	1	1	3	4	4	4	4	4	4	2	3	2
4	2	1	1	5	4	4	4	4	4	4	3	4	4
3	3	3	3	3	3	3	3	3	3	3	3	3	3
5	5	1	1	5	5	5	5	5	5	5	3	5	5

5	5	2	1	5	5	5	5	5	5	5	1	5	5
5	4	1	1	4	4	5	4	4	4	4	2	4	4
5	4	1	1	4	5	5	5	4	5	5	2	5	5
4	4	1	4	4	4	4	4	4	4	4	1	4	4
5	5	1	1	4	4	5	5	4	5	5	1	5	3
4	4	1	2	4	4	4	4	4	4	4	2	4	4
5	5	5	5	5	5	5	5	5	5	5	5	5	5
3	2	2	2	3	3	3	3	4	5	3	3	3	3
4	3	1	1	4	4	4	4	4	4	4	2	4	4
3	3	3	3	3	3	3	3	3	3	3	3	3	3
4	4	1	1	4	4	4	4	4	4	4	2	4	4
5	5	3	4	5	4	4	3	5	5	5	1	5	5
5	5	5	5	5	5	5	5	5	5	5	5	5	5
4	4	4	4	4	4	4	4	4	4	4	4	4	4
4	1	1	3	3	3	3	4	4	5	5	1	5	5
5	4	1	1	4	4	5	5	5	5	5	1	5	5
5	5	1	1	5	5	5	5	5	5	5	1	5	5
5	4	1	1	4	5	5	5	5	5	5	1	5	4
5	4	1	5	4	5	5	5	5	5	5	2	5	3
5	5	1	2	5	5	5	5	5	5	5	4	5	5
5	3	1	5	5	5	5	5	5	5	5	3	5	4
4	4	2	2	4	4	4	4	4	4	4	2	4	4
4	4	1	4	4	4	4	4	4	4	4	1	4	3
5	4	1	1	5	5	5	5	4	5	4	2	5	4
5	5	1	1	5	5	5	5	5	5	5	1	5	5
5	4	1	1	2	5	5	4	4	4	4	4	5	4
5	3	1	1	2	4	4	4	4	4	4	2	4	3
5	5	1	1	5	5	5	5	5	5	5	3	5	3
5	5	1	1	3	5	5	5	5	5	5	1	5	5
4	4	2	2	3	4	4	4	4	4	4	2	4	4
5	4	1	2	5	5	5	4	4	4	5	2	4	4
3	2	1	3	2	2	2	4	4	4	4	3	3	3
5	5	1	1	5	5	5	5	5	5	5	2	5	5
4	4	1	1	4	4	4	4	1	5	5	4	4	4
4	2	1	1	3	3	3	3	3	3	3	2	4	3
2	5	4	5	4	3	3	4	3	4	4	1	5	4
5	4	2	2	5	5	5	5	3	5	5	2	4	5
4	4	1	2	4	4	5	4	4	4	4	3	4	4
5	4	1	1	3	4	4	3	4	4	5	2	4	4
5	5	1	1	4	4	5	5	5	5	5	3	5	5
5	5	1	1	5	5	5	4	4	5	5	3	4	5
3	4	2	1	3	4	4	4	4	4	4	4	3	5
5	4	1	5	5	5	5	5	5	5	5	3	5	5
5	4	2	1	4	4	5	5	4	4	4	3	4	4
5	5	1	1	5	5	5	5	5	5	5	1	5	5
5	5	1	1	5	5	5	5	5	5	5	1	5	4
4	4	1	1	5	5	5	3	4	5	4	1	4	4
4	3	1	1	3	3	3	3	3	3	3	2	4	2
5	5	1	1	5	5	5	5	1	5	5	3	5	5
4	4	2	2	3	4	4	4	4	4	4	2	4	3
4	3	1	3	5	5	4	4	5	4	4	3	4	3
5	3	1	1	4	4	5	5	3	5	5	4	4	3
5	5	5	5	5	5	5	5	5	5	5	5	5	5
4	2	1	1	3	4	5	4	4	4	4	5	4	2
5	5	1	1	5	5	5	5	5	5	5	3	5	5
3	3	3	3	3	3	5	5	3	4	3	1	3	3

3	3	1	2	3	3	3	3	3	2	3	1	3	2
5	4	1	1	4	5	5	5	5	5	5	1	5	5
5	5	1	1	5	5	5	5	5	5	5	1	5	5
5	3	1	1	4	4	5	5	3	5	5	1	4	5
4	4	2	4	4	4	4	4	4	4	4	4	4	4
5	5	1	1	4	4	4	5	5	5	5	5	4	4
5	3	1	1	5	5	5	3	3	5	5	2	5	5
5	4	1	1	4	5	5	5	5	5	5	3	5	4
5	2	1	1	5	5	5	5	5	5	5	1	5	5
5	5	1	1	5	5	5	5	5	5	5	3	5	4
4	4	1	1	5	5	4	5	4	5	4	2	4	4
4	4	1	1	4	4	4	4	4	4	4	2	4	4
4	3	1	1	3	3	3	3	2	3	3	4	3	3
5	5	1	1	5	5	5	5	5	5	5	1	5	5
4	4	4	4	4	4	4	4	4	4	3	4	5	4
4	4	2	2	4	4	3	4	4	4	4	1	3	3
4	3	1	4	3	4	4	4	3	3	4	1	4	4
5	5	1	1	5	5	5	5	5	5	5	2	5	5
5	3	1	5	5	5	5	5	5	5	5	4	5	5
4	4	2	2	4	4	4	4	4	4	4	2	4	4
5	5	1	1	5	3	5	5	3	5	5	5	5	3
4	3	1	2	3	3	3	4	4	4	4	2	4	3
5	5	1	1	1	3	5	5	5	5	5	1	5	5
5	5	1	1	5	5	5	5	5	5	5	1	5	5
4	3	1	3	3	3	3	4	3	4	3	3	3	3
4	4	1	1	4	4	4	4	4	4	3	3	4	3
5	5	1	3	5	5	5	5	5	5	5	3	5	5
4	4	1	1	4	4	4	4	4	4	4	5	4	4
4	4	4	4	4	4	4	4	2	4	4	4	4	5
4	2	2	2	4	4	4	4	3	4	3	5	3	3
3	2	3	2	3	3	3	3	3	3	3	3	3	3
5	4	1	1	4	5	5	5	5	5	5	3	5	3
5	5	1	1	5	5	5	5	5	5	5	2	5	5
4	2	1	1	4	4	4	4	4	5	5	3	4	4
4	3	1	2	3	2	4	4	3	3	4	4	4	2
4	3	1	3	5	3	3	5	5	5	5	3	5	5
5	3	1	1	3	5	5	5	4	2	3	3	5	2
5	5	2	2	5	5	5	5	5	5	5	1	5	5
5	2	1	1	4	5	5	5	4	5	5	2	4	4
5	5	5	5	5	5	5	5	5	5	5	1	5	5
4	4	1	2	5	4	5	4	3	2	4	3	3	2
5	5	1	1	5	5	5	5	5	5	5	1	5	5
5	5	1	5	5	5	5	5	5	5	5	1	5	5
5	5	1	1	4	4	4	4	4	4	4	3	4	3
4	4	1	4	4	4	4	4	4	4	4	4	4	4
4	3	1	1	4	4	4	4	4	4	4	2	4	3
5	5	1	1	5	5	5	5	5	5	5	1	5	5
4	2	1	1	2	3	3	3	3	3	3	5	3	3
5	5	5	5	5	5	5	5	5	5	5	5	5	5
5	4	1	1	5	5	5	5	4	5	5	1	5	5
5	3	1	5	4	5	5	5	4	4	5	2	4	4
5	5	1	2	3	4	5	5	5	5	5	1	5	5
5	5	1	1	5	5	5	5	5	5	5	1	5	5
5	5	1	1	5	5	5	5	5	5	5	1	5	4
5	3	1	1	5	5	5	5	5	5	5	1	5	5
5	5	1	2	5	5	5	5	5	5	5	4	5	5

5	4	1	1	2	3	3	3	2	4	4	4	4	4
5	5	1	1	5	5	5	5	5	5	5	2	5	5
5	3	1	1	3	3	4	5	5	5	5	5	5	5
3	3	1	2	3	3	3	4	4	5	5	1	4	4
4	4	2	4	4	4	4	4	4	4	4	2	4	4
5	4	1	1	4	4	4	5	5	4	4	1	4	4
5	3	1	1	5	4	4	5	5	5	5	2	5	4
5	4	1	5	4	3	5	5	5	5	5	2	5	5
3	3	3	3	3	3	3	4	4	4	4	2	4	4
5	3	1	1	5	5	5	5	5	5	5	5	3	3
5	5	2	2	5	5	5	5	5	5	5	1	5	3
5	4	1	1	5	5	5	5	5	5	4	4	5	3
5	5	1	1	5	5	5	5	5	5	5	1	5	5
4	2	2	3	3	3	3	3	3	3	3	3	3	3
5	5	1	1	5	5	5	5	5	5	5	1	5	5
5	5	1	4	4	5	4	5	5	5	5	1	5	5
5	5	1	1	5	5	5	5	5	5	5	1	5	5
4	3	1	1	4	4	4	4	4	4	4	3	4	4
5	5	1	1	1	3	5	5	3	2	3	3	3	3
5	5	1	1	1	5	5	5	5	5	5	5	5	5
4	4	1	2	4	4	4	4	5	5	5	5	4	4
4	2	1	1	3	4	4	4	4	4	4	2	4	2
5	4	1	2	5	5	5	5	4	4	4	1	4	4
4	4	1	1	5	4	4	4	4	4	4	3	4	4
3	4	3	3	3	3	3	3	3	4	3	2	3	4
3	2	1	1	3	4	4	3	3	3	3	2	3	3
5	3	1	1	4	5	5	4	4	2	4	2	4	2
3	3	3	3	3	3	3	3	3	3	3	3	3	3
5	5	1	5	4	5	5	5	4	5	5	1	5	3
5	3	1	1	4	4	4	4	4	4	4	2	4	4
5	5	1	2	5	5	5	5	5	5	5	1	5	3
4	4	1	1	4	4	4	4	4	4	3	2	4	3
5	5	1	1	5	5	5	5	5	5	5	3	5	5
5	2	1	1	3	5	5	5	4	5	5	2	5	5
4	1	3	3	1	4	4	4	2	2	4	5	3	1
5	3	1	1	3	5	5	5	4	4	4	2	4	2
5	3	1	2	3	5	5	5	4	5	5	1	5	5
4	4	3	3	4	4	4	4	4	4	4	2	4	4
4	4	1	1	3	4	4	4	3	4	4	1	4	3
5	4	1	2	5	4	5	5	5	5	5	3	5	5
5	5	1	1	5	5	5	5	5	5	5	1	5	5
5	5	1	1	3	5	5	3	2	5	5	5	5	5
5	5	1	1	5	5	5	5	5	5	5	1	5	5
5	5	1	1	4	5	5	5	5	5	5	3	5	5
5	4	1	1	4	4	5	5	4	3	4	1	5	4
3	2	1	2	2	2	2	2	3	4	3	5	3	4
5	4	1	1	5	5	5	5	4	5	5	2	5	5
5	5	1	1	5	5	5	5	5	5	5	1	5	5
4	3	1	1	4	4	4	4	4	4	4	2	4	2
5	5	1	1	5	5	5	5	5	5	5	1	5	5
4	4	1	4	4	4	4	4	4	4	4	3	3	4
5	4	1	1	5	5	5	5	5	5	5	2	5	5
5	4	1	1	5	5	5	4	5	5	5	1	5	3
4	4	1	4	4	4	4	4	4	4	4	4	4	4
5	5	1	1	5	5	5	5	5	5	5	1	5	5
3	1	1	2	3	3	3	3	3	3	3	1	5	5

4	2	1	1	4	4	4	5	4	4	4	2	4	3
5	3	1	1	4	5	5	4	4	5	5	3	5	4
5	5	5	5	5	5	5	5	5	5	5	5	5	5
4	3	1	1	3	4	4	4	3	4	3	3	4	3
5	5	1	1	5	5	5	5	5	5	5	2	5	5
5	4	1	1	4	5	5	5	5	4	4	1	4	4
3	3	3	3	3	3	3	3	3	3	3	3	3	3
5	2	1	1	4	4	4	5	5	5	5	1	4	5
4	4	1	1	3	4	4	4	4	4	4	2	4	3
5	5	1	1	5	5	3	5	5	5	5	1	4	5
4	3	1	2	4	4	5	4	4	4	4	3	4	4
5	5	1	1	5	5	5	5	5	5	5	4	5	4
5	5	2	2	5	5	5	4	5	5	5	1	5	5
5	5	1	3	5	5	5	5	5	5	5	1	5	5
5	4	1	4	5	5	5	5	5	5	5	1	5	5
5	4	1	1	3	5	5	5	4	4	4	5	5	3
4	2	1	3	4	3	3	4	2	4	5	3	4	3
4	4	1	4	4	4	4	4	4	4	4	5	4	2
5	5	1	5	4	5	5	5	5	5	5	1	5	4
5	5	5	5	5	5	5	5	5	5	5	5	5	5
4	1	1	1	1	4	4	4	2	3	3	2	4	2
3	2	4	3	3	4	3	3	3	3	4	3	3	4
5	4	1	1	4	5	5	5	5	5	5	3	5	5
5	3	1	2	4	4	5	4	4	4	4	3	3	3
5	5	1	1	5	5	5	5	5	5	5	1	5	5
5	4	1	1	5	5	5	5	4	5	5	1	4	3
5	3	1	1	5	5	5	5	5	5	5	3	5	3
3	3	3	3	3	3	3	3	3	3	4	4	4	4
5	5	1	3	5	5	5	5	5	5	5	2	5	5
5	4	1	1	4	5	5	5	4	5	5	2	5	4
4	3	1	4	4	4	4	5	5	5	5	2	4	3
3	3	1	1	3	3	3	3	3	3	3	1	3	3
4	3	1	1	4	4	3	5	4	4	4	2	4	3
5	4	1	2	5	5	4	5	4	5	4	1	5	4
5	5	1	1	5	5	5	5	5	5	5	1	5	5
4	3	1	1	4	3	3	5	4	4	4	1	4	4
4	4	2	2	4	4	4	4	4	4	4	3	4	4
5	5	1	1	5	5	5	5	5	5	5	1	5	3
5	1	1	1	5	5	5	5	3	5	5	1	5	5
5	4	1	1	5	4	5	5	5	5	4	1	4	5
4	4	1	2	4	4	4	4	4	5	5	2	4	4
5	3	1	1	5	5	5	5	4	5	5	3	5	5
5	5	1	1	5	5	5	5	5	5	5	1	5	4
5	2	1	2	3	5	4	4	4	5	4	2	4	3
4	4	1	1	4	4	4	4	4	4	4	2	4	4
4	3	1	1	4	4	4	4	4	4	4	4	4	4
5	5	1	1	5	5	5	5	5	5	5	3	5	5
5	5	1	1	5	5	5	5	5	5	5	1	5	5
4	5	1	1	4	4	4	4	5	5	5	3	4	5
5	5	1	1	5	5	5	5	5	5	5	1	5	5
5	5	1	1	5	5	5	5	5	5	5	1	5	5
5	5	1	1	5	5	5	5	5	5	5	1	5	5
5	4	1	3	5	5	5	5	5	5	5	1	5	5
5	5	1	1	3	5	5	5	5	5	5	1	5	3
5	4	1	2	4	4	4	4	4	4	3	2	4	4
5	5	1	1	5	5	5	5	5	5	5	2	5	5

4	4	2	2	4	3	4	4	4	4	3	2	3	3
5	5	1	3	5	5	5	5	5	5	5	4	5	5
5	3	1	1	5	5	5	5	5	5	5	5	5	5
5	5	1	1	5	5	5	5	5	5	5	1	5	5
4	3	4	3	3	4	4	4	3	4	3	4	3	3
3	2	2	2	3	3	3	3	3	3	3	4	3	3
5	3	1	1	4	4	4	4	4	4	5	1	5	5
5	5	1	1	5	5	5	5	5	5	5	3	5	2
5	3	1	5	5	5	5	5	5	5	5	1	3	3
4	4	1	3	4	4	4	5	4	4	4	2	4	2
4	4	1	1	4	3	3	5	4	3	5	1	4	2
5	3	1	3	1	5	4	4	2	5	5	2	5	2
4	4	2	2	4	4	4	4	4	4	4	2	4	4
4	4	1	4	4	4	4	4	4	4	4	1	4	4
5	4	1	2	3	5	5	5	4	5	5	2	5	5
4	3	1	1	4	4	4	4	4	4	3	3	3	3
4	2	1	1	4	4	5	5	4	5	5	3	4	4
5	3	1	1	3	5	5	5	5	5	5	1	5	5
5	5	5	5	5	5	5	5	5	5	5	5	5	5
4	4	1	4	4	4	4	4	4	1	3	2	4	2
4	3	1	1	5	4	3	3	5	5	4	3	4	4
5	5	1	1	3	3	5	5	5	5	5	4	5	4
4	4	1	1	4	4	4	4	4	4	4	1	4	4
5	4	1	1	5	5	5	5	5	5	5	1	5	5
5	3	1	1	5	5	5	5	5	5	5	1	5	5
4	4	1	1	4	4	4	5	4	4	4	2	4	3
3	3	2	3	3	3	3	3	3	3	3	3	3	3
4	4	1	1	4	4	4	5	5	4	4	2	4	4
5	3	1	1	3	5	5	4	3	5	4	1	5	5
5	3	1	1	5	5	5	5	5	5	5	1	5	5
4	3	2	2	3	4	4	4	4	4	4	2	4	4
5	5	1	1	5	5	5	5	5	5	5	1	5	5
3	5	1	1	5	5	5	5	5	5	5	1	5	5
4	4	4	4	4	4	4	4	4	4	4	4	4	4
5	5	1	1	5	5	5	5	5	5	5	1	5	5
4	4	1	1	4	4	4	4	4	4	4	1	4	4
5	5	1	1	5	5	5	5	5	5	5	1	5	5
4	4	1	2	4	4	4	4	3	4	3	4	4	3
5	4	1	1	4	5	5	5	4	5	5	3	4	4
4	3	1	1	2	4	5	4	4	4	4	3	5	4
4	4	1	1	3	4	4	4	4	4	4	3	4	4
5	5	1	1	5	5	5	5	5	5	5	1	5	5
5	2	1	1	5	5	5	4	4	5	5	1	5	5
5	3	1	5	5	5	5	5	5	5	5	2	5	5
5	5	1	1	5	5	5	5	5	5	5	2	5	5
4	4	1	1	3	4	4	4	4	4	4	4	4	3
4	4	4	4	4	4	4	4	4	4	4	2	4	4
5	5	1	1	3	5	5	5	5	5	5	2	5	3
4	2	1	1	4	4	4	4	4	4	4	2	4	4
5	3	1	1	5	5	5	5	5	5	5	1	5	5
5	1	1	1	4	5	5	5	3	5	5	1	5	5
5	3	1	1	3	5	5	4	5	4	5	1	4	3
5	5	1	5	5	5	5	5	5	5	5	1	5	5
5	3	1	1	5	4	5	5	3	5	5	4	4	2
5	5	1	3	5	5	5	5	5	5	5	1	5	5
4	4	1	1	4	4	4	4	4	4	4	1	4	4

5	3	1	5	4	5	4	5	5	5	5	1	5	5
5	4	1	1	4	4	5	4	4	5	5	5	5	5
4	2	1	1	3	4	5	4	4	4	4	2	4	4
4	4	1	1	5	5	5	5	5	5	5	1	5	5
5	4	1	2	5	5	5	5	5	5	5	2	5	5
5	5	1	1	5	5	5	5	5	5	5	5	5	5
3	3	1	1	3	3	3	3	3	3	3	5	3	2
4	4	2	4	3	4	3	3	3	3	3	5	3	3
5	5	1	1	5	5	5	5	5	5	5	1	5	4
5	5	1	5	5	5	5	5	5	5	5	1	5	5
5	5	1	1	5	5	5	5	5	2	4	3	5	3
5	5	1	5	5	5	5	5	4	3	4	1	5	2
5	5	1	1	5	5	5	5	5	5	5	1	5	5
4	2	2	3	4	4	4	4	3	4	4	1	4	3
5	5	1	1	4	5	5	5	5	5	5	5	5	4
5	3	1	1	4	4	4	4	4	4	4	3	5	3
5	4	1	1	4	5	5	5	5	5	5	1	5	4
4	4	1	1	5	5	5	5	4	2	3	1	3	2
5	5	5	5	5	5	5	5	5	5	5	5	5	5
5	5	1	5	5	5	5	5	5	5	4	1	5	5
5	5	5	5	5	5	5	5	5	5	5	1	5	5
5	5	1	1	5	5	5	5	5	5	5	1	5	5
3	2	1	1	3	4	4	4	3	5	4	5	4	4
3	3	1	3	3	3	3	3	3	3	3	3	3	3
5	5	1	1	4	4	4	4	4	5	4	1	4	4
5	3	1	3	4	4	5	5	5	5	5	1	5	5
5	4	1	1	5	5	5	4	5	4	4	3	4	4
4	2	2	2	2	3	4	4	2	3	2	3	2	3
4	4	1	2	3	4	4	4	4	4	4	1	4	4
4	2	1	3	4	4	4	4	3	2	4	3	4	2
5	5	1	1	5	5	5	5	5	5	5	1	5	5
4	4	1	4	2	4	4	4	4	1	4	1	4	3
5	5	1	1	5	5	5	5	5	5	5	1	5	5
4	2	1	2	3	4	5	3	3	4	4	4	4	2
3	1	1	1	4	4	4	4	2	5	5	2	3	3
5	5	5	5	5	5	5	5	5	5	5	2	5	5
4	3	2	2	4	4	4	3	3	4	4	2	4	4
4	2	1	1	2	4	5	4	4	2	4	1	4	1
5	5	1	1	5	5	5	5	5	5	5	1	5	5
5	5	5	5	5	5	5	5	5	5	5	5	5	5
4	2	1	2	4	4	4	4	4	4	4	1	4	3
4	4	2	3	3	4	4	4	5	4	4	3	3	3
5	3	3	3	3	3	3	5	3	4	3	2	3	3
5	5	1	3	5	5	5	5	5	5	5	1	5	1
5	3	1	1	3	5	5	5	3	5	5	2	4	4
4	4	2	2	3	4	4	4	4	4	4	2	4	4
5	3	2	3	5	4	3	4	5	5	4	2	4	4
4	4	1	1	4	4	4	4	4	4	4	1	4	4
4	4	1	2	3	4	5	5	5	5	4	2	4	3
5	5	1	1	5	5	5	5	5	5	5	1	5	5
5	3	1	1	4	5	5	4	5	5	5	3	5	3
4	4	1	1	4	4	4	4	4	4	4	3	4	4
4	4	1	1	5	5	5	5	5	5	5	1	5	5
5	5	1	3	5	5	5	5	5	5	5	2	5	5
5	3	1	1	4	5	5	5	5	5	5	2	5	5
5	5	1	1	5	5	5	5	5	5	5	1	5	5

5	4	1	1	3	4	4	4	4	5	5	1	5	5
5	5	1	1	5	5	5	5	5	5	5	1	5	5
5	3	1	1	4	5	5	5	5	5	5	1	5	5
5	5	1	5	5	5	5	5	5	5	5	1	5	5
5	4	1	1	5	5	5	5	5	5	5	4	5	4
5	4	1	1	3	4	4	4	4	4	4	3	4	3
5	5	1	1	5	5	5	5	5	5	5	2	5	3
4	1	1	1	1	3	5	5	3	5	5	1	4	3
5	2	1	3	5	5	5	5	5	5	5	1	5	5
5	3	3	2	3	3	3	4	4	5	2	3	4	4
5	5	1	1	5	5	5	5	5	5	5	1	5	5
5	5	1	3	5	5	5	5	5	5	5	1	5	5
5	5	1	1	4	5	5	5	4	5	5	3	5	5
5	5	1	1	5	5	5	5	5	5	5	1	5	5
5	5	1	1	5	5	5	5	5	5	5	4	5	5
5	4	1	2	4	4	5	5	5	5	5	1	5	4
5	5	5	5	5	5	5	5	5	5	5	5	5	5
5	5	1	1	3	5	5	5	5	5	5	2	5	1
5	5	5	5	5	5	5	5	5	5	5	5	5	5
4	4	1	1	4	4	4	4	4	4	4	1	4	4
5	1	1	1	5	5	4	5	2	4	4	5	5	5
4	3	1	3	4	4	4	4	4	4	4	1	4	4
5	5	1	5	5	5	5	5	5	5	5	1	5	5
5	4	1	1	4	5	4	5	5	5	5	1	5	4
4	4	1	1	4	4	4	4	4	4	4	2	4	3
5	5	1	1	5	5	5	5	5	5	5	2	5	5
5	5	5	5	5	5	5	5	5	5	5	5	5	5
5	5	1	1	5	5	5	5	5	5	5	1	5	5
5	5	1	3	5	5	5	5	5	5	5	2	5	5
5	4	1	1	4	4	4	4	3	4	4	2	4	3
4	4	1	1	4	4	4	4	4	4	4	2	4	4
3	3	1	1	3	5	5	5	4	4	5	2	5	5
5	3	1	1	2	5	5	4	4	5	4	2	4	4
3	3	2	3	3	3	3	4	3	3	4	5	4	3
5	5	1	3	5	5	5	5	5	5	5	1	5	5
5	5	2	2	5	5	5	5	5	5	5	2	5	5
3	3	2	4	3	3	5	3	4	5	5	2	3	4
5	5	1	1	5	5	5	5	5	5	5	1	5	5
5	5	1	1	5	5	5	5	5	5	5	3	5	5
4	4	1	1	4	4	4	4	4	4	4	2	4	4
5	1	1	1	5	5	5	5	5	5	5	3	5	3
4	3	1	1	2	3	3	4	3	4	4	2	3	3
4	3	2	3	4	4	4	4	4	4	4	2	4	4
4	4	2	3	4	4	5	4	3	5	5	2	4	5
5	5	2	1	3	5	5	5	5	5	5	1	5	4
5	2	1	5	5	5	5	5	5	4	5	2	5	2
5	5	2	2	5	5	5	5	5	5	5	2	4	4
5	3	1	1	3	5	5	5	5	5	5	1	5	5
5	5	5	5	5	5	5	5	5	5	5	5	5	5
3	3	1	2	2	3	3	3	3	3	3	2	3	3
5	3	1	3	4	4	4	5	4	5	4	2	4	3
4	3	1	4	4	4	4	4	4	4	4	2	4	4
4	2	1	3	5	3	3	3	3	3	3	3	3	3
4	3	1	2	3	4	4	4	3	4	4	2	4	4
5	5	1	1	5	5	5	5	5	5	5	1	5	3
4	4	1	3	3	3	3	4	4	4	4	2	3	3

3	3	4	4	4	4	4	4	4	4	4	3	4	4
3	2	1	1	3	3	4	3	3	4	3	3	3	3
3	3	1	2	4	4	4	4	4	4	4	2	3	3
5	5	1	1	3	3	3	5	5	5	3	1	3	3
4	3	2	2	4	4	4	4	4	5	5	2	4	4
4	1	1	1	3	4	5	2	5	5	3	4	3	5
5	5	1	1	5	5	5	5	5	5	5	2	5	5
5	5	1	3	5	5	5	5	5	5	5	1	5	5
5	4	1	1	4	5	5	5	4	5	5	2	5	4
5	4	1	1	5	5	5	5	5	5	5	1	5	5
4	3	1	1	5	5	5	5	5	5	5	3	4	5
5	5	1	1	5	5	5	5	5	5	5	5	5	5
4	4	4	4	4	4	4	4	4	4	4	2	4	3
5	4	1	1	3	5	5	5	5	5	5	3	5	3
5	5	1	1	5	5	5	5	5	5	5	1	5	5
5	4	1	1	3	4	5	5	4	4	4	2	4	3
5	2	1	1	3	5	5	5	4	5	5	2	5	4
4	4	1	2	4	4	4	2	4	4	4	2	4	4
4	4	1	1	4	4	4	4	2	4	5	1	5	5
4	4	1	4	4	4	4	4	4	4	4	1	4	4
5	5	5	5	5	5	4	5	4	5	5	4	5	4
4	3	1	1	3	3	4	4	3	3	4	2	4	2
5	2	1	1	5	5	5	5	5	5	5	1	5	5
5	4	1	1	4	5	5	5	5	4	5	1	5	4
4	4	4	4	4	4	4	5	5	4	4	4	4	4
3	3	2	2	2	3	2	3	3	4	4	4	4	4
5	5	5	5	5	5	5	5	5	5	5	5	5	5
5	5	1	1	5	5	5	5	5	5	5	1	5	4
5	5	1	1	4	3	2	4	4	5	5	1	5	5
4	2	2	2	4	4	4	4	4	4	4	2	4	2
4	4	2	2	4	4	4	4	4	4	4	4	4	4
4	4	1	2	4	4	4	4	4	4	4	2	3	4
5	3	1	1	5	5	5	5	5	5	5	2	5	5
4	3	1	1	4	4	4	3	4	4	4	1	4	3
5	5	1	1	5	5	5	5	5	5	5	5	5	5
5	3	1	1	4	4	4	5	5	5	5	3	5	4
5	5	1	1	5	5	5	5	5	5	5	1	5	5
4	4	1	2	4	5	5	4	4	4	4	1	3	3
5	5	1	1	5	5	5	5	5	5	5	1	5	5
5	4	1	3	5	5	5	5	3	4	4	2	4	3
5	5	1	1	5	5	5	5	5	5	5	1	5	3
5	4	1	2	4	4	5	4	4	5	5	1	4	5
5	5	1	1	5	5	5	5	5	5	5	5	5	5
4	4	1	1	4	1	4	4	4	4	4	5	4	4
5	4	1	1	4	5	5	5	5	5	5	1	4	4
5	5	1	1	5	5	5	4	4	5	5	2	5	5
4	3	1	1	5	4	5	4	4	4	4	3	4	3
4	3	2	3	2	3	4	4	3	3	3	2	4	3
5	5	2	5	3	5	5	5	5	4	5	3	5	5
5	5	5	5	5	5	4	4	4	4	5	4	5	4
5	3	1	1	3	3	3	4	3	3	3	3	3	3
3	3	2	2	3	3	4	3	3	3	3	2	3	4
5	5	1	1	3	5	5	5	5	5	5	1	5	5
3	2	2	2	2	3	5	4	4	4	3	5	4	3
5	5	1	3	1	5	5	3	5	5	5	1	5	5
5	4	1	1	4	4	5	5	4	3	4	1	5	2

5	5	1	3	5	5	5	5	5	5	5	5	5	5
4	4	1	2	4	4	4	4	4	5	4	2	4	3
5	4	1	3	3	4	4	5	3	3	3	1	3	3
5	5	1	1	5	5	5	5	5	5	5	3	5	5
5	1	1	1	4	4	5	4	4	4	4	1	4	4
5	5	1	1	5	5	5	5	5	5	5	3	5	3
5	5	1	1	5	5	5	5	4	4	4	4	4	3
5	3	1	1	5	5	5	5	3	5	3	1	4	3
5	3	1	1	3	5	5	5	5	5	5	3	5	5
5	5	1	1	5	5	5	5	5	5	5	3	4	4
5	4	1	1	4	5	4	4	4	4	4	1	4	5
4	4	1	1	3	5	5	5	5	5	5	2	5	5
3	3	1	3	3	4	4	4	4	4	4	3	4	4
5	5	1	1	5	5	5	5	5	5	5	1	5	5
3	3	1	1	5	2	5	4	4	4	4	5	5	5
5	5	1	1	5	5	5	5	5	5	5	2	5	4
4	4	1	1	4	4	5	4	4	4	4	1	4	3
5	4	1	1	4	4	4	5	4	5	5	3	5	4
5	4	1	2	5	5	5	5	4	5	5	3	3	3
4	4	1	3	4	4	4	4	4	4	4	2	4	4
5	5	5	5	5	5	5	5	5	5	5	5	5	5
5	5	1	5	5	5	5	5	5	5	5	1	5	5
5	5	5	5	5	5	5	5	5	5	5	5	5	5
5	5	1	1	5	5	5	5	5	5	5	1	5	5
5	5	1	1	4	5	5	5	5	5	5	5	5	5
5	5	1	1	5	5	5	5	5	5	5	1	5	5
5	5	1	1	5	5	5	5	5	5	5	1	5	5
4	3	1	2	3	3	4	4	4	4	4	2	4	4
5	5	1	1	5	5	5	5	5	5	5	1	5	5
5	4	1	3	5	5	5	4	2	4	4	1	5	4
5	4	1	3	5	5	5	5	5	5	5	1	5	5
3	2	1	1	3	5	5	3	2	3	3	2	5	4
4	3	1	1	3	4	4	4	4	4	4	2	4	2
4	3	1	1	3	4	4	4	4	2	4	1	3	1
4	3	1	1	3	4	4	4	3	4	4	1	4	3
5	2	1	1	3	3	4	5	3	5	5	2	4	4
4	2	1	2	3	3	3	3	3	4	4	2	4	3
5	5	1	1	5	5	5	5	5	5	5	1	5	1
4	3	1	3	4	4	4	4	4	4	4	4	4	3
4	4	4	4	4	4	4	4	4	4	4	4	4	4
5	5	1	1	5	5	5	5	5	5	5	1	5	5
4	4	2	4	4	4	4	4	4	4	4	1	4	4
5	5	1	2	3	4	5	5	5	5	5	2	4	3
5	4	1	4	4	4	4	4	4	4	4	1	4	4
4	2	1	1	1	5	4	3	3	3	2	3	3	1
4	3	2	1	3	4	4	4	4	4	4	2	4	4
5	5	1	1	5	5	5	5	5	5	5	3	5	5
3	2	1	3	3	3	3	3	3	5	3	2	3	5
5	5	1	5	5	5	5	5	5	5	5	5	5	5
5	5	5	5	5	5	5	5	5	5	5	5	5	5
4	4	1	4	4	4	4	4	4	4	4	5	4	4
4	3	1	1	3	4	4	4	3	5	5	1	4	3
3	3	2	2	5	3	3	3	3	3	3	3	3	3
4	4	1	1	4	4	4	4	4	4	4	1	4	4
4	3	1	3	3	4	4	4	3	4	3	3	3	3
4	3	1	1	1	4	5	3	3	3	3	3	3	3

5	5	5	5	5	5	5	5	5	5	5	5	5	5
5	5	1	1	5	5	5	5	5	5	5	1	5	5
5	4	1	1	4	5	5	5	5	5	5	4	5	5
5	5	1	5	5	5	5	5	5	5	5	2	5	3
5	2	2	2	4	4	4	4	4	4	4	3	4	4
5	3	1	1	3	5	5	5	5	5	5	1	4	3
4	3	1	2	3	4	4	3	2	4	4	3	4	4
4	3	1	2	4	4	4	3	3	4	4	1	4	2
3	3	3	3	3	3	3	3	3	3	3	3	3	3
5	3	2	2	4	1	5	5	5	3	4	5	4	4
5	4	1	1	4	4	5	5	5	5	4	1	5	5
4	4	1	3	3	4	4	4	3	4	4	1	4	4
5	4	1	2	4	3	5	5	4	5	5	2	5	4
4	2	2	2	3	4	5	3	3	4	4	3	4	4
5	5	1	1	4	5	5	5	5	5	5	3	5	4
4	2	4	2	4	4	2	4	2	4	2	4	2	4
4	4	1	1	4	4	4	4	4	4	4	2	4	4
5	5	5	2	5	5	5	5	5	5	5	2	5	5
5	3	1	1	3	4	4	4	4	5	4	1	4	4
3	3	1	1	3	3	3	3	3	3	3	1	3	3
4	3	2	3	4	4	4	4	4	4	4	3	4	4
5	3	1	1	3	5	5	5	5	5	5	1	5	5
5	5	1	1	5	5	5	5	5	5	5	2	5	5
5	5	1	5	5	5	5	5	5	5	5	1	5	5
4	4	2	2	4	4	4	4	4	4	4	5	4	4
4	3	2	4	3	3	3	3	4	4	3	5	3	3
4	2	1	1	2	3	4	3	3	3	3	2	3	2
5	4	1	1	4	4	4	5	4	4	5	2	5	3
5	5	1	1	5	5	5	5	5	5	5	1	5	5
4	3	1	2	4	4	4	4	4	4	4	4	4	4
5	3	1	1	4	5	5	5	5	5	5	1	5	4
5	5	1	1	5	5	5	5	5	5	5	1	5	5
4	2	1	2	5	4	4	5	5	5	5	5	5	5
4	3	2	2	3	3	4	4	4	4	4	1	4	4
5	5	1	1	5	5	5	5	5	5	5	1	5	5
5	4	1	2	3	5	5	4	4	4	4	2	4	3
5	3	1	1	4	4	5	4	4	3	4	2	4	3
5	4	1	1	4	3	3	4	4	4	4	1	4	4
5	5	1	1	5	5	5	5	5	5	5	1	5	5
5	3	1	1	4	5	5	5	5	5	5	2	5	5
5	3	1	2	3	5	5	5	4	5	5	1	5	5
4	3	1	1	4	4	4	4	4	5	4	4	4	4
5	4	1	1	4	4	5	5	5	5	5	3	5	2
5	5	1	5	4	5	4	4	4	3	4	5	4	1
5	5	1	1	5	5	5	5	5	5	5	1	5	5
4	5	1	1	5	4	4	4	4	4	4	3	4	4
4	4	1	1	3	4	4	4	4	4	4	3	4	4
4	4	2	2	4	4	4	4	4	4	4	5	4	4
4	5	1	5	5	5	5	5	5	5	5	3	3	3
5	2	1	1	3	5	5	5	4	5	4	1	5	5
5	5	5	5	5	5	5	5	5	5	5	5	5	5
5	5	1	1	5	5	5	5	5	5	5	5	5	5
5	3	1	1	4	5	5	5	3	4	4	3	4	2
5	5	1	1	5	5	5	5	5	5	5	1	5	5
4	4	2	3	4	4	4	4	4	4	4	2	4	4
5	1	1	1	3	5	5	5	4	5	5	1	5	5

5	5	1	2	5	5	5	5	5	5	5	5	5	5
5	5	1	5	5	5	5	5	5	5	5	5	5	5
5	4	1	3	4	5	5	5	5	5	5	1	5	5
5	5	1	1	5	5	5	5	5	5	5	2	5	5
3	3	1	1	3	3	3	3	3	3	3	3	3	3
5	4	1	3	4	4	4	4	4	5	4	2	4	3
5	2	2	2	3	3	4	4	4	4	4	3	4	3
5	4	1	3	4	4	5	5	5	5	5	2	4	4
3	3	1	1	3	3	3	3	4	4	4	1	3	3
5	5	1	1	3	5	5	5	5	5	5	2	5	5
5	5	1	1	4	5	5	5	5	5	5	3	5	4
4	3	2	2	4	3	4	4	4	4	4	3	4	3
5	5	1	1	5	5	5	5	5	5	5	1	5	5
5	5	1	1	5	5	5	5	5	5	5	1	5	5
4	2	2	4	3	4	4	4	4	4	4	2	4	4
5	5	1	1	5	5	5	5	5	5	5	2	5	4
5	5	1	5	1	5	5	5	5	5	5	1	5	5
5	3	2	1	4	3	4	5	5	5	5	2	5	4
5	4	1	4	5	5	5	5	5	5	5	1	5	4
5	5	1	1	5	5	5	5	5	5	5	5	5	3
5	5	1	5	5	5	5	5	5	5	5	1	5	5
5	2	1	1	5	4	5	5	4	4	4	2	4	1
3	3	3	3	3	3	3	3	3	3	3	3	3	3
4	4	2	2	4	4	4	4	4	4	4	1	4	4
5	3	1	4	4	4	5	5	4	4	4	4	4	3
3	3	3	3	3	3	3	3	3	3	3	3	3	3
5	5	1	1	5	5	5	5	5	5	5	2	5	5
5	5	1	1	5	5	5	5	5	5	5	1	5	5
3	3	3	4	5	3	4	5	5	5	5	3	5	5
4	2	2	2	2	2	3	1	1	1	1	5	1	1
5	4	1	1	4	5	5	5	5	5	5	2	5	5
5	5	1	1	5	5	5	5	5	5	5	3	5	5
5	4	1	3	4	4	5	5	5	5	5	1	5	4
5	5	1	3	4	5	5	5	4	4	4	1	4	4
5	4	1	3	5	5	5	5	4	5	5	5	5	4
5	3	1	1	3	4	4	4	4	5	4	4	4	4
4	4	1	4	3	3	3	4	3	4	3	3	4	2
4	3	1	1	3	4	5	5	3	5	5	1	4	3
4	4	4	2	4	3	3	5	4	5	5	1	5	4
5	4	5	4	4	4	5	5	4	4	4	3	4	4
5	4	1	1	4	5	4	5	5	5	5	1	4	4
5	3	1	1	5	5	5	5	5	5	5	1	5	4
5	5	1	5	5	5	5	5	5	5	5	1	5	5
5	4	1	1	3	3	4	4	4	3	4	3	4	3
5	5	1	5	5	5	5	5	5	5	5	1	5	5
4	1	1	1	3	3	5	4	2	4	3	1	3	2
5	4	1	1	4	4	5	4	4	4	4	1	4	4
5	5	5	5	5	5	5	5	5	5	5	5	5	5
5	5	1	1	3	5	5	5	5	5	5	3	5	5
3	2	3	4	4	4	4	4	4	4	4	5	3	4
3	2	1	4	2	5	5	5	4	5	4	1	3	3
5	5	1	1	5	5	5	5	5	5	5	1	5	5
4	4	2	2	4	4	4	4	4	4	4	2	4	4
4	4	2	2	3	4	4	4	4	4	4	2	4	4
3	3	1	1	3	3	3	4	3	3	3	4	4	3
4	4	1	4	4	4	4	4	4	4	4	2	4	4

4	4	1	2	2	3	2	4	4	5	3	1	4	5
5	4	1	1	4	5	5	5	4	5	4	3	5	4
3	5	1	4	4	4	4	4	4	4	4	4	4	1
5	5	5	5	5	5	5	5	5	5	5	5	5	5
4	3	1	2	3	4	4	4	3	4	4	1	4	3
5	5	1	1	5	5	5	5	5	5	5	1	5	5
3	3	2	4	4	4	4	4	4	4	4	3	4	4
4	3	1	2	4	4	4	4	4	4	4	2	4	3
5	5	1	1	5	5	5	5	5	5	5	1	5	5
5	4	1	3	4	5	5	5	5	5	5	1	5	5
5	5	1	1	5	5	5	5	5	5	5	2	5	5
5	4	1	1	4	5	5	5	5	5	5	1	5	3
4	4	1	1	3	3	3	3	3	4	3	1	4	4
3	3	1	3	5	5	5	5	5	5	5	1	5	5
5	5	1	1	5	5	4	5	4	5	5	2	5	5
4	3	1	2	5	3	3	4	3	4	4	4	3	4
5	3	2	3	3	3	4	5	5	4	4	1	4	4
4	4	2	2	4	4	4	4	4	4	4	3	4	3
4	4	1	2	4	4	4	5	5	5	5	1	5	5
5	4	1	3	4	4	4	4	4	5	5	5	5	5
4	3	1	1	3	4	4	4	4	4	4	1	4	3
5	4	1	5	5	5	5	5	5	5	5	2	5	4
5	4	1	1	5	5	5	5	5	5	5	1	5	4
4	4	1	4	4	4	4	4	4	4	4	1	4	4
4	3	2	2	3	3	3	4	4	3	4	2	4	3
5	5	1	5	5	5	5	5	5	5	5	1	5	5
4	5	4	5	4	4	5	4	4	5	5	5	5	3
5	3	1	3	4	5	5	5	5	5	5	5	4	3
5	4	1	3	4	5	5	5	5	5	5	4	5	4
5	5	1	1	4	5	5	5	5	5	5	1	5	5
5	4	1	2	3	4	4	4	4	5	5	3	4	5
5	3	1	1	3	3	5	5	4	5	5	1	5	5
5	5	1	1	5	5	5	5	5	5	5	1	5	5
5	5	1	1	3	5	5	5	5	5	5	1	4	3
4	3	2	2	3	4	3	4	3	4	4	3	4	3
5	5	1	1	3	5	5	5	3	3	5	1	5	5
5	3	1	5	5	5	5	5	5	5	5	1	5	5
4	4	1	3	3	3	3	5	4	4	4	1	4	4
5	5	5	5	5	5	5	5	5	5	4	2	5	5
5	5	1	1	5	5	5	5	5	5	5	1	5	3
4	4	1	1	4	4	4	4	4	4	4	4	4	4
4	3	1	1	5	5	5	5	4	5	5	3	4	5
4	4	1	1	4	4	4	4	4	4	4	2	4	4
5	3	1	1	3	5	5	5	3	4	5	1	5	3
5	5	1	5	5	5	5	5	5	5	5	1	5	5
4	4	1	1	4	4	4	5	3	4	4	1	4	4
4	3	1	2	3	4	4	4	3	4	4	3	4	3
4	3	2	2	4	4	4	4	3	4	4	3	4	4
5	5	1	1	5	5	5	5	5	5	5	3	4	3

e20	e21	e22	e23	e24	e25	e26	e27	e28	e29	e30	e31	e32	e33
5	5	5	5	5	1	5	5	3	5	5	5	5	5
4	4	4	4	4	2	3	4	3	4	3	4	4	4
5	3	5	5	5	2	4	3	5	5	5	5	5	5
3	5	4	3	3	2	3	3	4	3	3	3	3	3
4	4	3	4	4	3	4	4	4	5	3	4	4	4
3	3	4	4	4	5	3	3	3	5	5	5	5	4
5	5	5	5	5	5	5	5	5	5	5	5	5	5
4	4	4	4	4	2	4	4	2	4	4	4	4	4
3	2	2	3	3	4	3	2	2	3	3	3	3	3
5	4	5	5	5	5	5	5	4	5	5	5	5	5
5	4	5	5	5	2	4	4	4	4	4	4	5	5
5	3	3	5	5	3	3	3	3	5	3	3	2	3
5	4	5	5	5	4	4	4	4	4	5	4	4	5
4	3	4	4	4	3	3	3	3	4	3	4	4	4
5	5	5	5	5	1	5	5	5	5	5	5	5	5
4	4	4	4	4	2	4	4	4	4	4	4	4	4
4	4	4	4	4	2	4	4	3	5	4	3	4	3
5	5	5	4	4	1	4	4	4	4	4	4	5	4
5	3	4	4	5	2	2	3	4	4	4	4	4	5
4	2	3	3	3	4	3	1	3	4	3	3	3	3
4	5	5	5	5	5	5	5	5	5	5	5	5	5
5	5	5	5	5	5	5	5	5	5	5	5	5	5
3	3	3	3	3	3	3	3	3	3	3	3	3	3
5	4	5	5	5	2	5	4	5	5	5	5	5	5
4	3	3	4	4	2	3	2	3	4	3	3	3	4
5	5	5	5	5	2	4	4	4	5	5	5	4	5
4	3	3	3	4	3	4	4	3	4	3	3	3	4
3	3	3	4	4	4	4	4	4	5	4	4	4	5
4	3	4	4	4	1	4	4	4	4	3	4	4	4
5	3	4	4	4	2	3	2	3	5	3	5	4	4
4	4	4	4	4	3	3	3	4	4	4	4	4	4
5	5	5	5	5	2	5	5	5	5	5	5	5	5
4	4	4	4	4	2	3	3	3	4	4	4	4	4
4	4	4	4	4	5	1	4	4	4	4	3	3	5
3	5	5	5	3	1	5	3	5	5	5	5	5	4
5	4	5	5	5	3	5	4	3	5	5	5	5	5
4	4	4	4	4	2	4	4	4	4	4	4	4	4
3	3	3	3	3	3	3	3	3	3	3	3	3	3
5	4	5	5	5	2	5	3	3	5	3	4	3	4
3	3	3	3	3	2	3	2	3	5	3	3	3	3
4	3	3	4	4	2	4	3	3	4	4	3	4	4
3	3	3	3	3	3	3	3	3	3	3	3	3	3
4	3	3	4	4	2	3	2	4	4	3	4	3	5
5	5	5	5	5	1	5	3	3	5	5	5	5	5
4	4	4	4	4	2	3	3	3	4	3	4	4	4
5	4	4	5	5	2	5	4	4	5	5	5	5	5
5	5	5	5	5	1	5	5	5	5	5	5	5	5
5	5	5	5	5	5	5	5	5	5	5	5	5	5
4	4	4	4	4	2	3	3	3	4	4	4	3	3
5	4	5	5	5	1	5	4	4	5	5	5	5	5

5	3	5	5	5	2	4	4	5	5	5	5	5	5
2	4	3	3	3	2	2	3	4	4	4	4	3	4
5	4	5	5	5	1	4	4	5	5	5	5	5	5
4	4	4	4	4	2	4	3	4	5	3	5	5	5
4	4	4	4	4	3	4	4	3	4	4	4	4	4
4	4	4	4	4	2	4	4	4	4	4	4	4	4
5	4	5	5	4	3	4	4	3	4	4	4	4	4
4	3	3	4	4	3	4	4	3	4	4	4	4	3
4	4	4	4	5	2	3	4	4	4	4	4	4	4
4	4	4	4	4	2	4	3	5	5	5	4	4	5
5	4	4	4	3	2	3	2	4	4	4	3	4	5
4	5	5	5	4	4	4	4	4	5	5	5	4	4
5	4	4	4	4	2	4	4	4	5	4	5	5	5
5	5	5	5	5	5	5	5	1	5	1	5	1	5
3	3	3	3	4	3	4	4	3	4	3	4	4	3
4	4	4	4	4	3	4	4	4	4	4	4	4	4
5	4	4	5	5	2	3	4	4	4	4	5	4	5
4	4	4	4	4	1	4	4	4	4	4	4	4	4
3	4	3	3	3	2	2	2	2	3	3	3	3	4
3	3	4	3	3	3	3	2	4	3	4	4	3	4
4	4	4	3	4	3	4	4	4	4	3	4	3	4
4	3	3	3	4	3	4	4	2	3	2	3	3	3
5	5	5	5	5	1	5	5	5	5	5	5	5	5
5	5	5	5	5	5	5	5	5	5	5	5	5	5
3	3	3	3	3	3	3	3	3	3	3	3	3	3
3	2	3	3	3	2	2	2	4	4	4	3	3	4
5	4	4	5	5	3	4	4	4	5	3	4	5	4
5	4	4	5	4	1	3	3	4	4	4	3	3	4
5	4	4	5	5	3	3	4	5	4	4	4	4	5
4	4	4	4	4	3	3	3	3	4	4	4	4	4
4	3	3	4	4	3	4	3	4	4	4	4	4	4
4	5	4	5	4	2	4	5	4	4	4	4	5	5
4	3	4	3	4	3	3	2	3	4	4	4	3	4
5	5	5	5	5	3	5	5	3	4	5	5	5	3
5	5	5	5	5	1	4	4	5	5	5	5	3	5
3	3	3	3	3	3	3	3	3	3	3	3	3	3
5	5	5	5	5	1	5	3	5	5	5	5	5	5
4	4	4	4	4	3	4	3	4	4	3	4	4	4
4	4	4	4	4	4	4	4	4	4	4	4	4	4
5	5	5	5	5	5	5	5	5	5	5	5	5	5
4	3	4	4	4	2	3	2	4	4	4	4	4	4
5	5	5	5	5	1	5	3	5	5	5	5	5	5
4	4	4	4	4	2	4	3	4	4	4	4	4	4
5	5	5	5	5	2	5	5	5	5	5	5	5	5
5	5	5	5	5	1	5	5	5	5	5	5	5	5
5	5	5	5	5	2	4	3	4	4	4	4	5	5
3	2	3	4	4	3	3	3	3	3	3	3	2	4
4	4	4	4	4	2	4	3	2	3	3	4	4	4
5	4	4	4	5	3	4	4	4	5	4	4	5	5
3	3	3	3	3	2	3	3	3	3	3	3	3	3
4	3	4	4	4	1	4	4	4	4	4	4	3	4
5	5	5	5	5	1	5	5	5	5	5	5	5	4
4	4	4	4	4	3	4	4	4	4	4	4	4	4
5	5	5	5	5	5	5	5	5	5	5	5	5	5
4	3	4	4	4	2	4	3	3	4	4	4	3	5
5	5	5	5	5	1	5	5	5	5	5	5	5	5

5	5	5	5	5	1	5	5	5	5	5	5	5	5
3	3	3	3	4	2	3	2	4	4	3	3	3	3
3	1	4	4	3	2	4	2	4	3	3	3	4	4
5	5	5	5	5	1	5	5	5	5	5	5	5	5
4	3	4	4	4	2	4	3	4	5	3	5	3	3
5	5	5	5	5	1	5	5	5	5	5	5	5	5
5	5	5	5	5	1	5	5	5	5	5	5	5	5
5	5	5	5	5	1	5	4	2	5	4	4	4	5
5	1	4	4	5	1	3	3	1	4	2	4	2	4
4	4	4	4	4	2	5	4	5	5	4	4	4	4
5	5	5	5	5	1	4	4	5	5	5	5	5	5
4	4	4	4	4	2	3	3	4	4	4	4	4	4
4	3	3	4	4	3	4	3	4	4	4	4	4	4
5	5	5	5	5	1	5	3	2	5	5	5	5	5
3	3	3	3	3	3	4	3	2	3	3	4	3	3
5	3	5	4	4	2	4	4	3	3	4	4	4	4
5	5	5	5	5	5	5	5	5	5	5	5	5	5
3	3	3	3	3	1	2	2	4	4	4	4	4	5
5	5	5	5	5	5	5	5	5	5	5	5	5	5
5	5	5	5	5	3	5	4	5	5	3	5	3	5
4	3	4	4	4	2	3	3	3	4	3	4	3	4
4	3	3	3	3	3	3	2	3	3	4	4	3	3
4	3	4	3	4	3	3	2	3	4	4	4	4	3
4	4	4	4	4	2	4	4	4	4	4	4	3	4
5	4	4	5	4	1	4	4	4	4	3	4	4	5
4	4	4	4	4	2	4	4	3	4	4	4	4	4
5	5	5	5	5	1	5	3	5	5	5	5	4	5
4	4	3	3	4	2	4	3	3	4	4	4	4	4
5	5	5	5	5	1	5	1	1	5	5	5	5	5
2	1	4	4	4	3	2	1	3	4	3	2	3	3
3	3	3	3	3	3	2	3	3	3	3	3	3	3
3	4	3	4	4	3	4	4	3	3	3	4	4	3
3	3	3	3	3	3	3	3	3	3	3	3	3	3
3	2	1	3	3	3	2	2	3	4	2	3	4	4
5	5	5	5	5	2	5	3	4	5	5	5	5	5
5	5	5	5	5	3	3	4	4	4	4	5	4	4
4	1	2	4	4	4	4	2	4	4	2	4	4	4
5	3	4	5	5	3	4	4	2	5	4	5	5	5
5	5	5	5	5	2	5	5	5	5	5	5	5	5
5	1	1	1	1	1	1	1	1	3	3	1	3	1
3	3	3	3	3	3	3	3	3	3	3	3	3	2
5	4	4	4	3	2	3	3	2	4	4	4	4	4
5	5	5	5	5	3	2	2	5	5	5	5	5	5
4	4	4	5	5	2	4	3	3	4	3	4	4	5
3	3	3	3	3	3	3	3	3	3	3	3	3	3
4	4	4	4	4	3	4	4	4	4	4	4	4	4
5	4	2	3	4	4	2	1	4	5	3	5	5	3
4	3	3	3	4	3	3	3	3	4	4	3	4	4
5	5	5	5	5	1	5	5	5	5	5	5	5	5
5	5	5	5	5	5	5	5	5	5	5	5	5	5
5	5	5	5	5	3	5	5	5	5	5	5	5	5
4	4	5	5	5	3	4	3	5	5	5	5	4	5
4	4	4	4	4	2	4	3	4	4	4	4	4	4
5	5	5	5	5	2	4	4	5	5	5	5	5	5
4	4	4	4	4	4	4	3	3	5	4	5	5	5
4	4	4	4	4	4	4	4	4	4	4	4	4	4

5	4	5	5	5	2	5	4	4	5	5	5	4	5
5	5	5	5	5	1	5	5	4	5	5	5	5	5
4	5	4	5	5	1	4	2	2	5	4	4	4	5
4	3	5	4	4	4	4	3	4	4	4	3	4	4
5	5	5	5	5	1	5	3	5	5	5	5	5	5
5	5	5	5	5	1	3	4	1	5	1	2	5	5
5	5	5	5	5	1	4	4	2	5	5	5	5	5
4	4	4	4	4	4	4	4	4	4	4	4	4	4
3	3	3	4	4	2	3	2	4	4	2	3	3	3
5	5	5	5	5	1	5	5	5	5	5	5	5	5
5	5	5	5	5	1	5	5	5	5	5	5	5	5
5	3	3	4	4	2	4	4	4	4	3	4	4	4
3	4	3	3	4	3	3	1	5	5	1	1	2	4
3	3	5	5	5	5	4	4	4	5	5	5	5	5
5	4	5	5	5	1	4	4	3	5	4	5	4	5
5	5	5	5	5	5	5	5	5	5	5	5	5	5
5	5	5	5	5	1	5	5	5	5	5	5	5	5
3	3	3	3	3	3	3	3	3	3	3	3	3	3
5	4	4	4	4	1	4	4	4	4	4	4	4	5
5	5	3	3	5	4	4	3	1	5	5	5	5	5
5	5	5	4	5	4	5	5	5	4	5	5	5	5
4	2	2	5	5	3	3	2	4	4	5	5	5	5
4	4	4	4	5	1	4	3	4	4	5	4	4	5
3	3	3	3	3	3	3	3	3	3	3	3	3	3
4	3	3	4	4	3	3	2	2	3	2	3	3	4
4	3	4	4	4	4	3	3	4	4	4	4	4	4
5	5	5	5	5	1	5	4	5	5	4	5	5	5
4	4	4	5	4	3	4	4	5	4	4	4	3	4
5	5	5	5	5	2	2	3	5	5	5	5	5	5
5	5	5	5	4	5	2	2	2	5	3	5	5	5
4	5	5	5	4	2	3	4	4	4	4	4	4	4
5	5	5	5	5	1	5	5	5	5	5	5	5	5
5	5	5	5	5	1	5	5	5	5	5	5	5	5
5	5	5	5	5	1	5	5	5	5	5	5	5	5
4	3	4	4	5	5	3	3	3	4	5	5	5	4
4	4	4	4	4	2	4	3	3	4	4	4	3	4
3	3	3	3	3	3	2	3	1	3	3	3	3	3
5	5	5	5	5	2	3	3	4	4	4	4	4	5
4	4	3	4	4	3	3	3	3	4	4	4	3	4
4	4	4	4	4	2	4	4	4	4	4	4	4	4
3	3	3	3	3	3	3	3	3	3	3	3	3	4
5	5	5	5	5	1	5	5	5	5	5	5	5	5
5	4	5	5	5	4	5	3	2	4	4	4	4	4
4	4	4	5	4	3	2	1	4	4	4	5	3	5
4	4	4	4	4	2	4	2	3	4	4	4	3	4
4	4	4	4	4	2	4	4	4	4	4	4	4	5
4	4	4	4	4	2	4	2	3	4	4	4	4	4
4	4	4	3	4	3	3	3	4	4	3	4	4	4
4	4	4	4	5	2	3	3	4	4	4	4	4	4
4	4	4	4	4	3	4	4	4	4	4	4	4	4
5	5	5	5	5	1	3	4	3	5	5	5	5	5
5	5	5	5	5	1	5	5	5	5	5	5	5	5
5	5	5	5	5	2	5	4	4	5	5	5	5	5
4	3	3	4	4	3	4	3	3	4	3	3	3	4
4	4	4	4	4	3	4	4	4	4	4	4	4	4
5	4	5	5	5	1	3	4	5	5	5	5	5	5

4	5	5	4	4	1	4	4	4	4	4	4	4	4
5	3	4	4	4	2	4	3	3	4	3	3	4	4
5	5	5	5	5	5	5	5	5	5	5	5	5	5
5	3	3	3	3	2	4	2	2	4	2	4	4	3
5	5	5	5	5	3	5	5	5	5	5	5	5	5
5	5	5	5	5	1	5	4	5	5	5	5	5	5
5	3	3	4	4	2	4	4	3	5	3	4	4	4
5	4	5	5	5	3	5	3	4	5	5	5	3	5
5	4	5	4	5	1	4	4	5	5	4	5	4	5
4	4	4	4	4	2	4	4	4	4	4	4	4	4
5	4	4	4	4	2	4	4	4	4	5	5	4	5
4	4	4	4	4	1	4	4	4	4	4	4	4	5
4	4	4	4	4	2	4	4	4	4	4	4	4	4
4	4	4	4	4	4	4	3	4	4	4	4	4	4
5	5	5	5	5	3	3	4	5	5	5	5	5	5
5	5	5	5	5	1	3	4	5	5	5	5	5	5
5	5	5	5	5	5	5	5	5	5	5	5	5	5
5	5	5	5	5	3	3	3	3	5	3	5	5	5
4	4	3	4	4	2	2	3	4	4	3	4	4	4
5	5	5	5	5	1	2	2	3	5	5	5	5	5
5	5	5	5	5	2	4	4	4	5	3	5	5	3
4	3	3	3	4	2	3	3	1	4	3	3	3	3
5	4	5	5	5	2	4	4	4	5	5	5	5	5
5	5	5	5	5	2	3	4	5	5	5	5	4	5
5	5	5	5	5	2	5	5	4	5	5	5	5	5
4	4	4	4	4	4	4	4	4	4	4	4	4	4
5	5	5	5	5	1	5	5	5	5	5	5	5	5
4	4	3	4	3	3	3	2	3	4	3	3	3	3
5	5	5	5	5	1	5	4	4	5	3	5	5	5
4	4	4	4	5	3	4	3	3	4	4	5	4	5
4	3	4	4	5	3	4	4	4	5	4	4	4	5
5	5	5	5	5	1	5	5	5	5	5	5	5	5
5	5	5	5	5	1	5	5	3	5	5	5	5	5
4	4	5	3	4	2	4	4	4	5	4	4	4	4
5	4	5	5	5	2	4	3	5	5	5	5	5	5
5	5	5	5	5	5	5	5	5	5	5	5	5	5
5	3	3	4	4	1	4	3	3	5	5	5	5	5
5	5	5	5	5	2	5	4	5	5	5	5	5	5
5	4	5	4	5	1	5	3	3	4	4	5	3	5
5	5	5	5	5	2	5	4	4	5	4	5	5	5
5	3	4	5	5	2	4	4	3	4	4	5	5	5
5	5	5	5	5	1	5	4	5	5	5	4	5	5
5	5	5	5	5	1	5	5	5	5	5	5	5	5
5	4	4	4	4	3	4	4	3	4	4	4	4	4
4	4	4	4	4	3	4	3	3	3	3	2	4	4
5	5	5	5	5	2	5	3	2	5	5	5	5	5
5	5	5	5	5	1	3	2	2	5	4	5	4	5
4	3	4	4	4	3	3	2	3	4	2	3	4	4
4	4	3	3	3	3	3	2	2	4	3	3	3	4
3	3	3	3	3	4	3	1	3	3	3	3	3	3
4	4	5	5	5	2	4	2	2	4	3	4	4	4
4	4	4	4	4	3	4	4	4	4	4	4	4	4
5	5	5	5	5	2	5	4	5	5	5	5	5	5
5	4	4	4	5	3	3	4	3	5	4	4	4	5
4	4	4	4	4	2	4	4	4	4	4	4	4	4
3	4	3	3	3	3	3	3	3	2	3	3	3	3

5	5	5	5	5	2	5	5	5	5	5	5	5	5
5	5	5	5	5	2	4	4	4	4	4	4	4	4
5	5	5	5	5	1	5	5	5	5	5	5	5	5
5	5	5	5	5	2	5	4	4	4	5	5	5	5
5	5	5	5	5	1	5	4	5	5	5	5	5	5
4	4	4	4	4	2	4	4	4	4	4	4	4	4
5	5	5	5	5	5	5	5	5	5	5	5	5	5
4	4	4	5	5	2	3	2	2	4	2	4	4	2
3	2	2	4	4	3	3	3	2	3	3	3	3	3
5	5	5	5	5	1	5	5	5	5	5	5	5	5
4	4	4	4	3	2	3	3	4	4	4	4	4	4
4	4	4	5	5	2	4	4	4	5	5	5	5	5
5	5	2	5	4	2	5	2	2	5	5	2	5	2
5	3	3	5	5	2	3	3	2	4	4	4	3	4
4	4	4	4	4	4	5	4	4	4	4	5	4	4
5	5	5	5	5	5	5	5	5	5	5	5	5	5
5	5	5	5	5	1	5	3	3	5	5	5	5	5
3	3	3	3	3	3	3	3	3	3	3	3	3	3
4	3	3	4	4	3	4	3	3	4	2	3	3	3
5	4	5	1	5	1	5	4	3	4	5	4	5	5
4	4	4	4	4	2	4	3	4	4	4	4	3	3
5	5	5	5	5	5	4	4	4	5	5	4	5	5
5	3	4	5	5	2	4	4	3	5	5	4	5	4
5	5	5	5	5	2	5	5	2	4	4	4	4	5
4	4	4	4	4	2	4	4	4	4	4	4	4	4
5	5	5	5	5	1	5	5	5	5	5	5	5	4
4	4	4	4	4	4	4	4	4	4	4	4	4	5
5	5	5	5	5	3	2	2	2	5	5	4	5	4
4	4	4	4	4	1	4	4	4	4	4	4	4	4
5	4	5	5	5	1	5	5	5	5	4	5	4	5
5	5	5	4	4	2	4	4	4	4	4	4	4	4
4	4	4	4	4	2	3	3	4	4	4	4	4	4
5	5	5	5	5	3	3	5	5	5	1	3	3	5
3	2	2	4	4	3	3	3	2	3	3	3	4	3
5	5	5	5	5	1	5	3	5	5	5	5	5	5
5	5	5	5	5	1	5	3	5	5	5	5	5	5
5	5	5	5	5	1	5	5	5	5	5	5	5	5
4	4	4	4	4	3	4	4	4	5	5	5	4	5
4	4	4	4	4	2	3	4	4	5	5	4	4	5
4	4	4	4	4	2	3	4	4	5	5	4	4	5
4	3	4	5	4	2	4	3	4	5	5	5	4	5
5	5	5	5	5	1	5	5	5	5	5	5	5	5
4	4	4	4	4	2	4	2	4	4	4	4	4	4
4	4	4	4	4	2	5	3	5	5	5	5	5	5
5	5	5	5	5	3	5	4	3	5	5	5	5	5
5	5	5	5	5	1	5	3	4	5	5	5	5	5
5	5	5	5	5	1	5	3	5	5	5	5	5	5
5	5	5	5	5	2	4	4	5	5	5	5	3	5
5	5	5	5	5	5	5	5	5	5	5	5	5	5
4	4	5	4	5	3	4	3	4	4	3	4	4	5
5	3	3	3	5	3	5	3	4	4	4	4	4	3
1	3	1	1	4	4	4	4	1	1	1	3	1	1
4	3	4	4	4	3	3	3	3	4	4	4	3	4
4	4	4	4	4	1	3	2	4	4	4	4	4	4
3	3	3	3	3	3	3	3	3	3	3	3	3	3
5	5	5	5	5	2	4	3	5	5	5	5	5	5

5	5	5	5	5	5	5	5	5	5	5	5	5	5
4	3	4	4	4	2	4	3	4	4	4	4	4	4
5	5	5	5	5	1	5	4	3	5	5	4	5	5
4	4	4	4	4	1	4	4	4	4	4	4	4	4
5	5	5	5	5	1	4	4	4	5	5	4	5	5
4	4	4	4	4	2	4	3	3	4	4	4	4	4
5	5	5	5	5	3	5	5	5	5	5	5	5	5
3	3	3	2	3	2	2	3	5	3	3	3	3	3
4	4	4	4	4	2	4	3	2	4	4	4	4	4
3	3	3	3	3	3	3	3	3	3	3	3	3	3
4	3	3	4	4	2	3	3	3	4	3	3	3	4
5	5	5	5	5	3	4	4	5	5	5	5	5	5
5	5	5	5	5	5	5	5	5	5	5	5	5	5
4	4	4	4	4	4	4	4	4	4	4	4	4	4
5	5	5	5	5	1	5	5	5	5	5	5	5	5
5	5	5	5	5	1	3	3	5	5	5	4	5	5
5	5	5	5	5	1	5	5	5	5	5	5	5	5
5	5	5	5	5	1	4	4	5	5	5	5	5	5
5	3	5	5	5	2	3	2	4	5	3	4	4	5
5	5	5	5	5	3	5	5	3	5	5	5	5	5
4	4	5	5	5	3	5	4	4	5	5	4	4	5
4	4	4	4	4	3	4	3	2	4	4	4	4	4
4	4	4	4	4	2	4	4	4	4	4	4	4	4
5	5	5	5	5	4	4	3	4	5	5	5	3	4
5	5	5	5	5	1	5	5	5	5	5	5	5	5
5	4	4	4	4	2	3	4	3	4	4	4	4	4
5	4	4	4	4	2	4	2	3	4	4	4	4	4
5	5	5	5	5	3	5	3	3	5	5	5	5	5
5	5	5	5	5	1	5	5	5	5	5	5	5	5
4	4	4	4	4	2	3	2	4	4	4	4	2	4
4	4	5	4	4	3	4	4	3	4	2	4	4	2
3	3	3	3	3	2	3	2	2	2	2	2	2	3
5	4	5	5	5	2	4	4	5	5	4	4	5	5
4	4	4	4	4	1	4	4	4	4	4	4	4	4
3	3	3	3	3	2	3	3	3	3	3	3	3	3
4	5	4	4	3	2	5	4	5	5	4	5	4	4
5	5	5	5	5	2	3	4	4	5	5	5	5	5
4	4	4	4	4	3	4	4	3	4	4	4	4	4
4	4	4	4	4	3	3	4	4	4	3	3	3	4
4	5	5	5	5	1	5	5	5	5	5	5	5	5
5	4	5	4	5	3	4	3	5	5	5	5	4	5
5	4	4	4	4	2	4	4	2	4	3	3	3	5
5	5	5	5	5	5	5	5	5	5	5	5	5	4
4	4	4	4	4	2	4	4	4	4	4	4	4	4
5	5	5	5	5	1	5	5	5	5	5	5	5	5
5	5	5	5	5	2	5	4	4	5	5	5	4	5
5	4	4	4	4	2	4	4	5	4	4	4	2	4
3	3	3	3	3	4	3	2	2	3	3	3	3	3
5	5	5	5	5	5	5	5	5	5	5	5	3	5
4	4	4	4	4	2	4	3	4	4	4	4	4	4
4	4	4	5	4	1	5	4	4	5	4	5	4	4
4	4	4	4	4	2	4	4	5	5	4	4	5	5
5	5	5	5	5	5	5	5	5	5	5	5	5	5
4	4	4	4	4	2	2	2	3	4	4	4	4	4
5	5	5	5	5	1	5	3	3	5	5	5	5	5
3	3	3	3	3	3	3	3	2	3	3	3	3	3

3	3	3	3	3	2	3	3	1	3	3	3	3	3
5	5	5	5	5	2	5	4	3	5	5	5	5	5
5	5	5	5	5	1	5	5	5	5	5	5	5	5
4	5	5	5	5	1	4	3	2	5	5	5	5	5
4	4	4	4	4	4	4	4	4	4	4	4	4	4
3	3	4	4	4	2	4	2	4	4	4	4	2	4
5	3	5	5	5	4	2	2	3	5	5	4	5	5
5	5	5	5	5	3	3	4	4	5	4	4	4	5
5	5	5	5	5	1	5	4	5	5	5	5	5	5
5	5	5	5	5	1	5	5	4	5	4	4	4	4
5	3	4	4	5	2	4	3	4	5	4	4	5	4
4	3	4	4	4	2	4	3	4	4	4	4	4	4
4	3	3	4	4	2	3	2	3	3	3	4	3	3
5	5	5	5	5	1	5	5	5	5	5	5	5	5
4	4	4	4	4	2	4	4	4	4	3	3	3	4
3	3	3	3	3	3	2	3	3	3	3	3	3	3
3	4	4	3	4	3	4	3	3	4	4	3	4	4
5	5	5	5	5	1	5	5	5	5	5	5	5	5
5	5	5	5	5	1	5	3	4	5	5	5	4	5
4	4	4	4	4	2	2	4	4	4	4	4	4	4
5	5	5	5	5	4	5	4	3	5	5	5	5	5
4	3	3	3	3	2	2	3	4	4	4	4	4	4
5	5	5	5	5	2	5	5	5	5	5	5	5	5
5	5	5	5	5	1	5	5	5	5	5	5	5	5
4	3	4	4	4	3	3	3	3	3	3	4	4	4
4	3	4	4	4	2	3	3	3	4	4	4	4	4
5	5	5	5	5	2	2	3	5	5	5	5	5	5
4	4	4	4	4	2	2	2	2	4	4	4	4	4
4	5	5	5	5	5	1	3	3	4	4	4	4	3
3	3	3	4	2	4	2	2	2	4	3	4	2	2
3	3	3	3	3	3	3	3	3	3	3	3	3	3
5	4	4	4	4	1	4	4	5	4	4	4	4	4
4	4	4	5	5	4	4	4	5	5	5	5	5	5
4	4	4	4	4	2	5	4	4	5	5	5	5	4
4	3	3	4	4	4	3	3	4	4	3	2	3	3
5	5	5	5	5	1	4	3	5	5	5	5	5	5
5	3	4	5	5	2	3	3	2	5	3	3	3	3
5	5	5	5	5	2	5	5	5	5	5	5	5	5
4	3	4	5	5	3	5	3	4	4	4	4	4	4
5	5	5	5	5	1	5	5	5	5	5	5	5	5
3	3	2	4	4	3	2	3	3	4	3	4	3	3
5	5	5	5	5	1	5	5	5	5	5	5	5	5
5	5	5	5	5	1	5	5	5	5	5	5	5	5
4	4	4	4	4	3	4	3	3	4	4	4	4	4
4	4	4	4	4	4	4	4	4	4	4	4	4	4
4	3	4	4	4	3	3	2	4	4	3	3	4	3
5	3	5	5	5	1	3	3	5	5	5	5	5	5
3	3	3	3	3	3	3	3	3	3	3	3	3	3
5	5	5	5	5	5	5	5	5	5	5	5	5	5
5	5	5	5	5	2	5	5	5	5	5	5	5	5
5	4	4	4	5	3	4	3	3	5	4	3	4	4
5	4	5	5	5	1	5	5	5	5	5	5	5	5
5	5	5	5	5	1	5	5	5	5	5	5	5	5
5	5	5	5	5	2	3	2	5	5	5	4	5	5
5	5	5	5	5	2	5	5	5	5	5	5	5	5
5	5	5	5	5	2	5	4	4	5	5	5	5	5

5	4	5	4	4	1	3	4	2	3	4	4	4	4
5	5	5	5	5	1	5	5	5	5	5	5	5	5
5	5	5	5	5	1	4	4	4	4	5	4	5	4
4	4	4	4	4	2	4	4	4	4	4	4	4	4
4	4	4	4	4	3	4	4	4	4	4	4	4	4
5	3	4	5	5	2	4	3	3	5	4	4	5	5
5	4	4	5	5	2	4	4	4	4	4	4	4	4
5	5	5	5	5	2	5	4	4	4	4	4	4	5
4	4	4	4	4	4	4	4	4	4	4	4	4	4
5	5	5	5	5	1	3	2	3	5	5	5	5	5
5	5	5	5	5	2	2	5	5	5	5	5	5	5
4	4	4	4	4	2	4	3	3	5	4	4	4	4
5	5	5	5	5	1	5	5	5	5	5	5	5	5
3	3	3	3	3	3	3	3	3	3	3	3	3	3
5	5	5	5	5	1	5	5	5	5	5	5	5	5
5	5	5	4	5	2	4	4	4	4	4	4	5	5
5	5	5	5	5	1	5	4	5	5	5	4	5	5
4	4	4	4	4	3	4	4	4	4	4	4	4	4
3	3	3	3	3	3	3	3	3	3	3	3	3	3
5	5	5	5	5	5	5	5	5	5	5	5	5	5
4	4	4	4	4	3	3	3	4	4	4	4	3	4
4	3	3	4	4	2	3	2	2	4	2	2	3	4
4	5	5	5	5	3	4	4	3	4	5	4	4	5
4	4	4	4	4	2	3	3	3	4	3	4	3	4
3	3	3	3	4	3	3	3	3	2	3	3	4	3
3	3	3	3	3	3	2	2	4	3	3	3	3	3
5	3	4	4	4	2	3	3	3	5	2	4	4	4
3	3	3	3	3	3	3	3	3	3	3	3	3	3
5	4	5	4	4	2	4	3	3	5	4	4	3	5
4	4	4	4	4	2	4	3	2	4	4	4	4	4
5	4	4	5	5	2	4	4	4	4	4	4	4	5
3	2	3	4	4	3	4	3	2	4	4	3	3	4
5	5	5	5	5	3	5	5	5	5	5	5	5	5
5	5	5	5	5	2	5	3	3	5	4	4	4	4
4	4	4	4	4	3	3	3	2	4	1	4	3	1
4	3	3	5	5	3	2	2	2	4	2	3	3	3
5	4	5	5	5	3	3	4	5	5	5	5	5	5
4	4	4	4	4	2	4	4	4	4	4	4	4	4
4	4	4	4	4	4	4	4	3	4	4	4	4	4
5	5	5	5	5	2	5	3	4	4	4	4	4	5
5	5	5	5	5	1	5	5	5	5	5	5	5	5
5	5	5	5	5	1	4	4	4	5	5	5	5	5
5	5	5	5	5	5	3	5	5	5	5	5	5	5
5	4	5	5	5	4	4	3	3	5	4	5	5	5
4	4	4	4	4	3	4	4	4	5	5	4	4	4
2	3	3	2	2	4	3	3	4	3	3	4	4	4
5	5	5	5	5	3	5	5	5	5	5	5	5	5
5	5	5	5	5	1	5	5	5	5	5	5	5	5
3	3	3	3	3	1	3	2	2	3	3	3	3	2
5	5	5	5	5	1	5	5	5	5	5	5	5	5
4	4	4	4	4	4	4	3	4	4	4	4	4	4
5	5	5	5	5	3	5	5	5	5	5	5	5	5
5	4	4	4	4	3	4	4	5	4	4	4	3	5
4	4	4	4	4	1	4	4	4	4	4	4	4	4
5	5	5	5	5	1	5	4	4	4	4	4	4	4
5	5	5	5	3	1	3	3	1	3	3	3	3	3

4	4	4	4	4	4	4	4	2	4	4	4	4	4
5	4	4	4	5	2	4	2	5	4	5	5	5	5
5	5	5	5	5	5	5	5	5	5	5	5	5	5
4	3	3	4	3	2	3	3	3	3	3	3	3	3
5	3	5	5	5	1	4	3	5	5	5	5	3	5
4	4	4	4	4	1	4	4	4	4	4	4	4	4
3	3	3	3	3	3	3	3	3	3	3	3	3	3
5	5	5	5	5	3	5	3	2	5	5	5	5	5
4	3	4	2	4	1	3	3	5	4	3	3	4	3
5	4	4	4	4	1	3	2	5	5	5	5	5	5
4	4	4	5	4	2	4	3	5	4	4	4	4	5
5	4	4	4	4	2	4	4	4	4	4	4	4	5
5	5	5	5	5	2	5	5	5	5	5	5	5	5
5	5	5	5	5	1	5	4	5	5	5	5	5	5
5	4	5	4	5	2	5	4	4	4	5	5	5	5
4	5	5	4	5	1	3	3	3	5	4	5	5	4
4	4	4	4	3	3	4	2	1	4	3	4	3	4
4	4	4	4	4	2	3	3	4	4	4	4	4	4
5	4	5	5	5	1	5	4	4	5	5	5	5	5
5	5	5	5	5	5	5	5	5	5	5	5	5	5
4	3	4	4	4	4	4	3	2	3	4	2	3	4
3	4	3	3	4	4	3	4	3	4	3	4	3	3
5	5	5	5	5	5	5	4	4	5	5	5	5	5
4	4	4	4	4	3	4	4	2	5	4	4	3	4
5	5	5	5	5	1	5	4	5	5	5	5	5	5
4	4	5	5	5	2	4	4	4	4	5	4	4	4
5	4	4	4	4	3	3	4	4	5	5	5	5	4
4	3	3	3	4	1	4	3	4	4	4	4	4	4
5	5	5	5	5	1	5	4	5	5	5	5	5	5
4	5	4	4	4	3	3	4	4	5	4	5	5	5
4	4	4	4	4	1	4	3	3	5	4	4	4	4
3	3	3	3	3	3	3	3	3	3	3	3	3	3
4	4	4	4	4	2	2	3	4	4	3	3	3	4
5	4	5	5	5	1	4	4	4	5	4	4	4	4
5	5	5	5	5	1	5	5	5	5	5	5	5	5
4	4	4	4	4	2	3	3	4	4	4	4	3	4
4	4	4	4	4	2	4	3	4	4	4	4	4	4
5	5	5	5	5	1	5	5	5	5	5	5	5	5
5	5	5	5	5	2	3	4	3	5	5	5	5	5
4	4	5	5	5	1	4	4	5	5	4	5	3	5
4	4	4	4	4	2	4	3	4	4	4	4	4	4
5	5	5	5	5	2	5	4	4	5	5	5	5	5
5	5	5	5	5	1	5	5	5	5	5	5	5	5
4	4	4	4	4	2	3	3	4	4	4	3	4	4
4	4	4	4	4	2	2	4	4	3	4	4	4	4
4	4	4	4	4	2	3	3	3	4	4	4	4	4
5	5	5	5	5	1	5	5	5	5	5	5	5	5
5	5	5	5	5	2	5	5	5	5	5	5	5	5
4	4	5	5	4	2	4	3	2	4	4	4	3	4
5	5	5	5	5	3	5	4	5	5	5	3	5	5
5	5	5	5	5	1	5	5	5	5	5	5	5	5
5	5	5	5	5	1	5	5	5	5	5	5	5	5
5	5	5	5	5	5	5	5	5	5	5	5	5	5
5	5	5	5	5	1	4	3	5	5	5	5	4	5
4	4	4	4	4	2	3	3	5	5	4	4	4	4
5	5	5	5	5	2	5	4	3	5	5	5	5	5

3	3	2	4	3	3	2	3	2	2	2	2	2	4
5	5	5	5	5	2	3	3	5	5	5	5	5	5
5	5	5	5	5	1	5	5	5	5	5	5	5	5
5	5	5	5	5	1	5	5	5	5	5	5	5	5
4	3	4	4	3	3	3	3	4	3	3	4	3	4
3	3	3	3	3	3	3	3	3	3	3	3	3	3
5	5	5	5	5	1	5	5	5	5	5	5	5	5
5	5	5	5	5	2	3	5	4	5	5	5	5	4
3	3	3	3	3	3	3	3	3	3	3	3	3	3
4	4	4	4	4	2	4	4	4	4	4	4	4	4
4	5	5	5	5	4	5	3	2	4	4	3	3	4
5	4	4	5	4	3	4	3	3	5	5	4	4	5
4	4	4	4	4	2	4	4	4	4	4	4	4	4
4	4	4	4	4	2	4	4	4	4	4	4	4	4
5	5	5	5	5	2	4	4	4	5	5	5	5	5
4	4	4	4	4	3	3	3	3	4	3	3	3	3
4	4	4	4	4	2	4	4	3	4	3	4	5	5
5	5	5	5	5	3	3	2	4	5	5	5	5	5
5	5	5	5	5	5	5	5	5	5	5	5	5	5
4	3	4	5	5	2	3	3	4	4	5	4	4	3
4	4	4	4	4	1	4	4	4	4	4	4	4	4
5	2	4	4	5	1	4	4	4	4	4	4	4	5
4	4	4	4	4	1	4	4	3	4	4	4	4	4
5	5	5	5	5	1	5	4	4	5	5	5	4	5
5	5	5	5	5	1	4	5	5	5	5	5	5	5
4	4	4	4	4	2	4	3	3	4	4	3	4	4
3	3	3	3	3	3	3	3	3	3	3	3	3	3
4	3	4	5	4	3	4	4	5	4	4	3	4	4
4	4	4	4	4	2	4	4	3	4	3	4	4	4
5	5	5	5	5	1	5	4	5	5	5	5	5	5
4	3	4	4	4	2	3	3	3	4	3	3	4	4
5	5	5	5	5	1	5	5	5	5	5	5	5	5
5	5	5	5	5	1	1	5	5	5	5	5	5	5
4	4	4	4	4	4	4	4	4	4	4	4	4	4
5	5	5	5	5	1	5	5	5	5	5	5	5	5
4	4	4	4	4	2	4	4	4	4	4	4	4	4
5	5	5	5	5	1	5	5	5	5	5	5	5	5
4	3	3	4	4	3	3	4	2	4	2	4	4	3
5	4	4	5	5	2	3	4	3	5	3	4	4	4
4	4	4	4	4	4	4	3	2	3	2	3	3	3
4	4	4	4	4	2	3	3	4	4	3	4	4	4
5	5	5	5	5	1	5	5	5	5	5	5	5	5
5	5	5	5	5	1	5	3	3	4	4	4	4	4
5	5	5	4	4	2	4	4	4	4	5	5	4	5
5	5	5	5	5	1	5	4	5	5	5	5	5	5
4	4	4	4	4	1	4	3	3	4	4	4	4	4
4	4	4	4	3	3	4	4	3	4	3	4	4	4
5	3	4	5	5	1	5	3	2	5	5	5	5	5
4	4	4	4	4	2	4	4	3	4	4	4	4	4
5	5	5	5	5	1	5	5	5	5	5	5	5	5
5	5	5	5	5	3	5	5	2	5	5	5	5	2
4	4	4	4	4	2	3	4	4	4	4	4	3	4
5	5	5	5	5	1	5	3	3	5	5	5	5	5
5	5	5	5	5	3	4	2	1	5	5	5	5	5
5	5	5	5	5	1	5	5	5	5	5	5	5	5
4	4	4	4	4	1	4	4	4	4	4	4	4	4

5	5	5	5	5	1	5	5	5	5	5	5	5	5
5	5	5	5	5	2	4	3	4	5	4	5	5	5
4	3	4	3	3	2	3	3	4	4	4	4	4	4
5	5	5	5	5	1	5	5	5	5	5	5	5	5
5	5	5	5	5	2	5	5	5	5	5	5	5	5
5	5	5	5	5	1	5	3	5	5	5	5	5	5
2	3	3	3	3	3	3	3	2	2	2	2	2	2
3	3	3	3	3	3	3	3	3	3	3	3	4	2
5	3	3	5	5	1	5	2	3	3	5	5	5	5
5	5	5	5	5	1	5	5	5	5	5	5	5	5
5	5	5	5	5	2	3	4	5	5	4	5	5	4
5	4	4	4	4	2	3	3	3	4	4	4	4	4
5	4	5	5	5	2	4	4	5	5	5	5	5	5
4	4	4	4	4	3	4	2	3	4	4	4	4	4
5	4	5	5	5	1	5	5	4	5	5	5	5	5
5	4	4	4	4	2	4	2	4	5	4	4	4	4
5	5	5	5	5	2	5	4	3	5	5	4	5	5
4	2	3	3	3	2	2	2	2	4	2	3	4	3
5	5	5	5	5	5	5	5	5	5	5	5	5	5
5	5	4	4	4	1	4	4	5	4	4	5	5	4
5	5	5	5	5	1	5	5	5	5	5	5	5	5
5	5	5	5	5	1	5	5	5	5	5	5	5	5
3	3	3	3	3	2	2	2	4	4	3	4	3	3
3	3	3	3	3	3	3	3	3	3	3	3	3	3
4	4	4	4	4	2	4	3	3	4	4	4	4	4
5	5	5	5	5	5	5	5	5	5	5	5	5	5
4	4	5	4	5	2	4	4	5	5	5	5	5	4
3	2	2	3	3	3	2	2	1	4	2	2	2	2
4	4	4	4	4	2	4	4	4	4	4	4	4	4
4	4	4	4	4	2	4	4	2	4	2	4	4	2
5	5	5	5	5	1	5	3	4	5	3	3	5	5
4	4	4	4	4	1	4	4	3	4	4	4	4	4
5	5	5	5	5	1	5	5	5	5	5	5	5	5
3	3	3	3	3	3	2	3	2	4	3	4	4	3
3	4	4	4	4	2	4	4	2	4	4	4	4	4
5	5	5	5	5	2	4	4	4	5	5	5	5	3
4	3	3	4	4	3	3	3	3	4	3	4	4	3
4	2	4	4	4	4	4	1	2	4	2	4	4	3
5	5	5	5	5	1	4	5	4	5	5	5	5	5
5	5	5	5	5	5	5	5	5	5	5	5	5	5
4	4	3	4	4	2	4	4	4	4	3	4	4	4
4	3	4	4	4	3	2	3	3	4	3	3	3	4
4	3	3	3	3	4	3	3	3	3	3	3	3	4
5	5	5	5	5	2	5	5	1	5	5	5	5	5
5	4	3	3	5	1	3	4	5	5	5	5	5	3
4	4	4	4	4	2	4	3	4	4	4	4	4	4
4	4	4	4	4	4	4	4	3	4	4	4	5	4
4	4	4	4	4	2	4	3	3	4	4	4	4	4
4	4	4	4	4	2	4	4	3	3	4	4	4	4
5	5	5	5	5	1	5	5	5	5	5	5	5	5
5	4	4	4	5	4	4	3	4	5	5	4	3	4
4	4	4	4	4	4	4	4	4	4	4	4	4	4
5	5	5	5	5	2	3	3	5	5	5	5	5	5
5	5	5	5	5	1	5	5	5	5	5	5	5	5
5	5	5	5	5	2	5	3	5	5	5	5	5	5
5	5	5	5	5	1	5	5	5	5	5	5	5	5

4	5	4	4	4	1	4	3	5	5	5	5	3	5
5	5	5	5	5	1	3	5	5	5	5	5	4	5
5	3	5	5	5	1	5	4	5	5	5	5	5	5
5	5	5	5	5	3	3	3	3	3	3	3	3	3
5	5	5	5	5	1	4	4	2	4	4	5	5	5
4	3	3	4	3	1	3	3	5	5	5	5	5	4
5	5	5	5	5	1	3	3	4	5	5	5	5	5
5	3	3	3	3	3	3	3	5	5	3	3	5	3
5	5	5	5	5	3	4	4	5	5	5	5	5	5
4	5	3	3	4	3	1	2	5	5	3	3	3	5
5	5	5	5	5	1	5	3	4	5	5	5	5	5
5	5	5	5	5	2	5	4	5	5	5	5	5	5
5	4	5	5	5	1	3	4	5	5	5	5	3	5
5	5	5	5	5	1	5	5	1	5	5	5	5	5
5	5	5	4	5	1	5	4	5	5	5	5	5	5
5	4	4	4	4	3	3	4	3	5	3	5	5	4
5	5	5	5	5	5	5	5	5	5	5	5	5	5
5	5	5	5	5	2	2	3	3	4	4	4	4	4
5	5	5	5	5	5	5	5	5	5	5	5	5	5
4	4	4	4	4	1	4	4	4	4	4	4	4	4
5	4	4	4	5	2	2	3	2	5	4	4	5	4
4	4	4	4	4	4	4	4	4	4	4	4	4	4
5	5	5	5	5	1	1	5	5	5	5	5	5	5
5	4	5	4	4	1	4	4	5	5	4	5	5	5
4	4	4	4	4	4	4	4	4	4	4	4	4	4
5	5	5	5	5	2	5	5	5	5	5	5	5	5
5	5	5	5	5	5	5	5	5	5	5	5	5	5
5	5	5	5	5	1	5	5	5	5	5	5	5	5
5	5	5	5	5	3	5	5	2	5	5	5	5	5
4	4	4	4	4	3	3	4	3	4	4	4	4	4
4	4	4	4	4	4	4	4	4	4	4	4	4	4
5	5	5	5	5	3	3	3	4	4	5	5	5	5
4	4	4	4	4	3	3	3	3	5	5	2	4	5
3	3	2	3	3	3	3	3	3	3	3	3	3	3
5	5	5	5	5	1	5	5	5	5	5	5	5	5
5	5	5	5	5	2	5	5	5	5	5	5	5	5
4	4	4	4	4	3	3	3	4	4	3	4	4	4
5	5	5	5	5	1	5	5	5	5	5	5	5	5
5	5	5	5	5	3	4	4	4	4	4	4	4	4
4	4	4	4	4	2	4	4	4	4	4	4	4	4
5	5	5	5	5	5	4	4	5	5	3	5	5	5
4	3	4	4	3	2	2	2	2	4	2	4	3	4
4	4	4	5	5	2	3	3	3	4	4	5	4	4
5	5	5	5	4	2	3	4	3	4	5	5	4	4
5	5	5	5	5	2	4	5	5	5	5	5	5	5
5	4	3	5	5	2	4	3	4	5	5	3	4	5
4	4	4	4	4	2	4	3	4	4	4	4	4	4
5	5	5	5	5	2	2	3	5	5	5	5	5	5
5	5	5	5	5	5	5	5	5	5	5	5	5	5
3	3	3	3	3	1	4	1	5	4	4	4	4	4
4	3	3	3	4	2	3	3	5	4	4	3	3	5
4	4	4	4	4	1	4	4	1	4	4	4	4	4
3	3	3	3	3	3	3	3	3	3	3	3	3	3
4	4	4	4	4	2	4	3	3	4	4	4	4	4
5	4	5	5	5	3	4	4	4	5	5	4	4	5
3	3	3	3	4	1	3	2	3	3	3	3	3	3

4	4	4	4	4	2	3	3	3	3	3	3	3	3
3	3	4	3	3	3	3	3	5	5	4	3	3	3
4	3	3	4	4	2	3	3	4	4	3	3	3	3
3	3	3	3	3	3	3	3	3	3	3	3	3	5
4	4	5	3	5	3	4	4	3	4	4	4	4	4
5	5	5	5	5	4	3	3	5	5	5	5	5	5
5	5	5	5	5	2	5	4	4	5	5	5	5	5
5	5	5	5	5	3	5	5	5	5	5	5	5	5
5	4	5	5	5	2	3	4	5	5	5	5	5	5
5	5	5	5	5	1	4	4	4	5	5	5	5	5
5	5	5	5	5	3	3	3	2	4	5	5	5	4
5	5	5	5	5	1	5	5	5	5	5	5	5	5
4	4	4	4	4	4	4	4	4	4	4	4	4	4
5	3	5	5	5	3	5	3	5	5	5	4	5	5
5	5	5	5	5	1	5	5	5	5	5	5	5	5
4	3	4	4	4	2	3	3	4	5	4	4	5	4
5	4	4	4	4	1	4	3	3	5	5	5	5	4
4	2	4	4	4	2	3	3	4	4	3	2	3	4
5	5	5	5	5	2	3	4	5	5	5	4	2	4
4	4	4	4	4	2	3	3	4	4	4	4	4	4
5	4	5	5	4	5	5	5	5	5	5	5	5	5
4	3	3	3	3	2	2	2	5	4	3	2	3	4
5	5	5	5	5	1	5	3	5	5	5	5	5	5
5	4	4	4	5	2	3	4	4	4	4	4	5	5
4	4	4	4	4	4	4	4	4	4	4	4	4	4
4	3	3	3	3	3	3	2	3	3	3	3	3	3
5	5	5	5	5	5	5	5	5	5	5	5	5	5
5	5	5	5	5	2	5	4	4	4	4	4	4	5
5	5	5	5	5	1	2	5	1	5	5	5	5	5
4	4	4	4	4	2	4	2	2	4	2	4	3	4
4	4	4	4	4	4	4	4	4	4	4	4	4	4
4	4	4	4	4	3	3	3	3	4	4	4	4	3
5	5	5	5	5	1	5	4	4	5	5	5	5	5
4	4	4	4	4	2	3	4	4	4	4	4	4	4
5	5	5	5	5	1	5	5	5	5	5	5	5	5
4	4	5	5	5	1	3	4	4	4	2	3	3	4
5	5	5	5	5	1	5	5	3	5	5	5	5	5
4	3	3	4	3	2	4	3	3	4	4	5	4	5
5	5	5	5	5	1	5	5	5	5	5	5	5	5
4	4	4	4	4	2	3	4	3	4	3	4	2	4
5	5	5	5	5	3	3	3	5	5	5	5	3	5
5	5	5	5	5	2	4	4	5	5	5	5	5	4
5	5	5	5	5	5	5	5	5	5	5	5	5	5
4	1	4	5	3	2	3	4	4	4	4	4	4	4
5	5	5	4	5	1	4	4	4	4	4	4	5	5
5	5	3	5	4	2	4	3	1	5	4	5	4	5
4	4	3	4	4	3	3	3	3	4	4	4	4	3
3	3	3	3	3	3	2	3	3	4	3	3	3	3
5	5	5	5	5	2	5	3	3	4	5	5	5	5
4	4	4	4	4	4	4	4	4	4	4	4	4	4
3	3	3	3	3	3	3	3	3	3	3	3	3	3
3	4	4	4	3	3	3	3	3	3	3	3	3	4
5	3	5	5	5	1	3	3	5	5	5	5	3	5
3	3	3	4	3	2	3	3	3	4	4	4	4	4
5	5	5	5	5	1	5	5	5	5	5	5	5	5
5	3	4	4	5	3	4	2	2	4	5	4	5	5

5	5	5	5	5	2	3	5	5	5	5	5	3	5
4	4	4	4	4	2	4	3	5	4	4	4	4	4
3	3	3	3	3	3	3	3	3	3	3	3	3	3
5	5	5	5	5	3	5	5	5	5	5	5	5	5
4	4	4	4	4	1	3	4	4	4	4	4	4	4
5	5	5	5	5	2	5	5	3	5	5	5	5	5
4	4	4	5	5	1	4	2	2	5	5	5	5	5
5	5	5	5	5	3	3	2	4	5	5	5	3	5
5	5	5	5	5	1	5	3	3	5	5	5	5	5
4	4	4	5	5	1	4	4	5	5	5	5	5	5
4	4	4	4	4	1	4	4	5	5	5	4	5	4
5	5	5	5	5	1	5	4	5	5	5	5	3	5
4	4	4	4	4	3	3	2	4	4	4	4	4	4
5	5	5	5	5	1	5	3	5	5	5	5	5	5
5	5	5	5	5	5	5	5	5	5	5	5	5	5
5	5	5	5	5	1	4	4	5	5	5	5	5	5
4	5	4	4	4	1	3	3	4	4	3	4	4	4
4	4	4	4	4	1	3	3	4	4	4	4	4	4
4	4	4	4	5	2	4	3	3	5	4	4	5	5
4	4	4	4	4	2	4	4	4	4	4	4	4	4
5	5	5	5	5	5	5	5	5	5	5	5	5	5
5	5	5	5	5	1	5	5	5	5	5	5	5	5
5	5	5	5	5	5	5	5	5	5	5	5	5	5
5	5	5	5	5	1	5	3	4	5	5	4	5	5
5	5	5	5	5	3	4	4	4	4	3	4	4	3
5	5	5	5	5	1	5	5	5	5	5	5	3	5
5	5	5	5	5	1	5	4	5	5	5	5	5	5
4	4	4	4	4	3	3	3	4	4	4	4	4	4
5	5	5	5	5	1	5	5	5	5	5	5	5	5
5	4	4	4	5	1	4	3	4	5	4	4	4	4
5	5	5	5	5	1	5	3	5	5	5	5	5	5
4	2	4	4	4	2	2	2	2	5	2	2	4	3
4	3	4	4	4	2	3	3	3	4	4	4	4	4
4	3	2	4	4	3	3	3	2	4	2	3	3	3
4	3	4	4	4	3	3	1	3	3	3	3	3	4
4	4	4	3	5	2	3	2	2	5	4	3	3	5
3	4	4	4	4	3	2	2	2	3	3	3	3	3
5	5	5	5	5	1	5	5	5	5	5	5	5	5
4	4	4	4	4	2	3	3	4	4	4	4	4	4
4	4	4	4	4	4	4	4	4	4	4	4	4	4
5	5	5	5	5	1	5	5	5	5	5	5	5	5
4	4	4	4	4	2	4	4	4	4	4	4	4	4
4	3	4	4	4	4	4	4	3	4	4	4	4	4
4	4	4	4	4	1	4	4	4	4	4	4	4	4
4	3	1	4	4	3	3	1	4	3	2	3	3	3
4	4	4	4	4	3	4	2	2	4	3	4	4	4
5	5	5	5	5	2	5	4	5	5	5	5	5	5
3	3	3	3	3	3	3	3	5	5	5	5	3	5
5	5	5	5	5	3	5	5	5	5	5	5	5	5
5	5	5	5	5	5	5	5	5	5	5	5	5	5
4	4	4	4	4	2	4	4	4	4	4	4	4	4
4	5	4	3	4	2	3	2	3	4	3	4	4	4
3	3	3	3	3	3	3	3	2	3	3	3	3	3
4	3	4	4	4	1	4	3	4	4	4	4	4	4
4	2	2	3	2	3	2	3	2	4	2	3	4	3
3	3	3	3	3	3	3	3	3	3	3	3	3	3

5	5	5	5	5	5	5	5	5	5	5	5	5	5
5	5	5	5	5	1	5	5	5	5	5	5	5	5
5	5	5	5	5	1	3	3	5	5	3	5	5	5
5	5	5	5	5	2	5	4	5	5	5	5	5	5
4	4	4	4	4	4	4	4	4	4	4	4	4	4
4	4	4	4	4	4	4	4	3	5	4	4	4	4
4	2	4	3	3	2	4	3	3	4	4	4	4	4
4	4	4	4	4	3	3	3	2	4	3	3	3	4
3	3	3	3	3	3	3	3	3	3	3	3	3	3
4	5	5	5	5	4	4	2	4	4	4	4	4	3
5	3	5	5	5	1	5	5	5	5	5	5	5	5
4	3	4	4	4	1	4	2	4	4	4	4	4	4
5	4	4	4	4	1	3	3	3	5	5	3	5	4
5	3	3	4	4	3	4	3	3	4	4	3	3	3
5	4	5	5	5	1	5	4	5	5	5	5	4	5
2	4	2	4	2	3	4	2	3	4	2	4	2	4
4	4	4	5	5	2	3	3	4	4	5	4	5	4
5	5	5	5	5	2	5	5	5	5	5	5	5	5
4	4	4	5	5	3	3	4	5	4	4	4	3	5
3	3	3	3	3	1	3	3	3	3	3	3	3	3
4	4	4	4	4	2	3	4	4	4	4	4	4	4
5	3	5	5	5	3	3	3	3	5	5	5	5	5
5	5	5	5	5	2	5	5	5	5	5	5	5	5
5	5	5	5	5	1	5	5	5	5	5	5	1	5
4	4	4	4	4	4	4	4	4	4	4	4	4	4
3	3	3	3	3	2	3	2	3	3	3	3	3	3
4	2	3	4	4	4	3	3	3	4	3	3	2	3
5	3	4	4	5	2	3	4	4	4	4	4	4	4
5	4	5	5	5	1	4	4	5	5	5	5	5	5
4	4	4	4	4	3	3	4	4	4	4	4	3	4
5	4	4	4	5	2	3	3	4	5	4	4	4	5
5	4	5	5	5	2	3	3	3	5	5	5	5	5
4	3	4	4	4	3	4	2	2	2	2	2	2	4
4	4	4	4	4	1	3	4	4	4	4	4	4	4
5	5	5	5	5	1	5	5	5	5	5	5	5	5
4	4	4	4	4	2	3	3	4	4	5	5	5	3
4	3	3	4	4	2	3	4	3	4	4	3	4	3
4	3	3	4	4	2	3	2	4	4	4	3	3	4
5	5	5	5	5	1	5	5	5	5	5	5	5	5
5	4	5	5	5	2	5	3	5	5	5	3	5	5
5	4	5	5	5	2	4	3	4	5	5	5	5	5
4	3	4	4	4	2	3	3	3	4	3	4	3	4
5	3	4	5	4	3	4	4	5	5	4	4	4	5
5	4	4	4	4	1	4	4	3	4	3	4	4	4
5	5	5	5	5	1	5	5	5	5	5	5	5	5
4	4	4	4	4	2	4	4	3	4	2	4	4	4
4	4	4	4	4	1	3	3	3	4	4	4	4	4
4	4	4	4	4	2	4	3	4	4	4	4	4	4
4	5	5	4	4	4	4	4	5	5	4	4	4	4
5	5	5	5	5	2	5	5	5	5	5	5	5	5
5	5	5	5	5	5	5	5	5	5	5	5	5	5
5	5	5	5	5	5	5	5	5	5	5	5	5	5
4	4	4	5	5	3	3	3	3	5	4	4	3	4
5	5	5	5	5	1	5	5	5	5	5	5	5	5
4	4	4	4	4	2	3	3	4	4	4	4	4	4
5	5	5	5	5	1	1	5	2	5	1	5	5	3

5	5	5	5	5	5	5	5	5	5	5	5	5	5
5	5	5	5	5	5	5	5	5	5	5	5	5	5
5	5	5	5	5	1	5	4	4	5	5	4	5	5
5	5	5	5	5	1	3	3	3	5	5	5	5	5
3	3	3	3	3	2	3	3	4	4	4	4	4	4
4	4	4	4	4	2	4	3	3	4	4	4	4	4
3	3	3	3	4	4	3	3	3	4	4	4	4	4
4	4	4	4	4	2	4	4	4	4	4	4	4	4
3	3	3	3	3	2	3	3	3	3	3	3	3	3
5	5	5	5	5	2	4	4	4	5	5	5	5	5
5	5	5	5	5	1	4	4	5	5	5	4	5	5
4	4	4	4	4	2	3	3	3	4	4	4	4	3
5	5	5	5	5	1	5	5	5	5	5	5	5	5
5	5	5	5	5	1	5	4	5	5	5	5	5	5
4	4	4	4	4	4	3	4	4	4	4	4	4	4
5	4	4	4	4	2	4	4	4	4	4	4	4	4
5	5	5	5	5	2	5	3	5	5	5	5	5	5
4	4	4	4	4	2	4	4	4	4	4	4	4	4
4	5	5	4	4	1	4	5	4	5	5	5	5	5
5	3	5	5	5	5	5	4	4	5	5	5	5	5
5	5	5	5	5	1	5	5	5	5	5	5	5	5
4	2	3	3	1	1	2	1	3	4	4	3	4	4
3	3	3	3	3	3	3	3	3	3	3	3	3	3
4	4	4	4	4	1	4	4	4	4	4	4	4	4
4	4	4	4	4	1	4	3	4	4	4	4	4	4
3	3	3	3	3	3	3	3	3	3	3	3	3	3
5	5	5	5	5	1	5	5	5	5	5	5	5	5
5	5	5	5	5	1	5	5	5	5	5	5	5	5
5	5	5	5	5	3	5	5	5	5	5	5	5	5
5	1	1	1	1	5	1	1	1	1	1	1	5	4
5	5	5	5	5	1	5	4	5	5	5	5	5	5
5	5	5	5	5	1	5	5	5	5	5	5	5	5
4	4	4	4	4	1	4	4	4	4	4	4	4	5
5	5	5	5	5	2	4	4	4	4	4	4	4	5
5	1	5	5	5	2	5	4	4	5	5	2	5	5
4	3	4	4	4	2	4	3	4	4	4	4	4	4
3	3	3	3	3	3	3	3	3	3	3	3	3	3
4	3	3	4	4	3	4	3	3	4	4	4	4	5
5	3	3	3	3	2	3	3	3	3	3	3	3	5
4	3	4	4	4	3	5	4	5	4	4	4	4	4
4	3	4	4	5	1	4	3	4	5	4	4	4	4
5	5	5	5	5	2	5	4	4	4	4	4	5	5
5	5	5	5	5	1	5	5	5	5	5	5	5	5
4	3	4	4	4	2	3	4	4	4	4	3	3	4
5	5	5	5	5	1	5	5	5	5	5	5	5	5
4	3	3	3	4	2	3	1	3	3	2	4	3	3
5	4	4	5	5	1	4	4	4	4	4	4	3	4
5	5	5	5	5	5	5	5	5	5	5	5	5	5
5	5	5	5	5	5	5	5	5	5	5	5	5	5
4	3	3	4	4	4	4	3	4	4	4	4	4	4
4	2	3	3	3	2	2	2	3	4	3	4	3	3
5	5	5	5	5	1	5	4	5	5	5	5	5	5
4	3	4	4	4	2	4	4	4	4	3	4	4	4
4	3	4	4	4	3	4	4	4	4	3	4	4	4
4	3	3	4	4	2	3	2	3	4	4	4	3	3
4	4	4	4	4	2	4	4	4	4	4	4	4	4

5	5	5	5	5	2	5	3	5	5	5	5	5	5
4	3	4	5	4	1	4	3	2	5	4	4	5	5
4	4	4	4	4	2	4	4	4	4	4	4	4	4
5	5	5	5	5	5	5	5	5	5	5	5	5	5
4	3	3	4	4	2	3	3	3	4	3	3	4	3
5	5	5	5	5	1	5	5	5	5	5	5	4	5
4	4	4	4	4	4	4	4	4	4	4	4	4	4
4	4	4	4	4	3	4	3	4	4	4	4	4	4
5	5	5	5	5	5	5	5	5	5	5	5	5	5
5	5	5	5	5	2	4	3	5	5	5	5	5	5
5	5	5	5	5	1	4	4	5	5	5	5	5	5
5	4	4	5	5	2	4	4	4	5	4	5	4	5
4	4	4	4	4	2	3	3	5	4	4	4	4	4
5	5	5	5	5	5	5	5	5	5	5	5	5	5
5	5	5	5	5	1	4	4	5	5	5	5	5	5
4	4	4	3	4	4	4	4	3	4	4	4	4	4
4	4	3	3	4	2	3	3	3	3	3	3	3	4
4	4	4	4	4	3	3	3	3	3	4	4	4	3
5	3	5	5	5	4	5	3	1	4	4	4	4	4
5	5	5	5	5	4	4	4	4	4	4	4	4	4
4	4	4	4	4	2	4	3	3	4	4	4	4	4
5	5	5	5	5	1	4	4	5	5	5	5	5	5
5	5	5	5	5	1	5	5	5	5	5	5	4	5
4	4	4	4	4	2	2	2	4	4	4	3	3	4
3	4	4	4	4	3	4	3	3	4	4	4	4	4
5	5	5	5	5	1	5	5	5	5	5	5	5	5
5	3	5	4	5	4	5	4	4	5	5	4	5	5
5	5	4	5	5	3	3	4	5	4	4	5	4	5
5	5	5	5	5	3	5	4	4	5	5	5	5	5
5	5	5	5	5	1	5	4	4	5	5	5	5	5
5	5	5	5	5	2	4	3	5	5	5	5	5	5
5	4	4	5	5	1	5	3	5	5	5	4	4	4
5	5	5	5	5	1	5	5	5	5	5	5	5	5
5	4	5	5	5	3	3	4	3	5	4	3	5	5
4	4	4	4	4	2	3	3	3	4	4	4	4	3
5	2	5	5	5	1	5	5	3	5	5	5	5	5
5	5	5	5	5	1	2	5	5	5	5	5	3	5
4	4	4	4	4	2	2	2	3	3	3	3	3	4
5	5	5	4	5	2	5	5	5	5	5	5	5	5
5	5	5	5	5	1	5	3	3	5	5	5	5	5
4	4	4	3	4	2	4	3	2	4	3	4	4	4
5	5	5	5	5	3	5	5	5	5	5	5	5	5
4	4	4	4	4	2	4	3	4	4	4	4	4	4
5	5	5	5	5	1	3	3	5	5	5	5	5	5
5	5	5	5	5	1	5	5	5	5	5	5	5	5
4	3	4	4	4	1	4	3	4	4	4	4	4	4
4	4	4	4	5	3	4	3	4	4	4	3	3	4
4	4	4	4	3	3	3	2	4	4	3	4	3	4
4	3	3	4	4	4	4	4	4	4	4	4	4	4

e34	e35	e36	e37	e38	e39	e40	e41	e42	e43	e44	e45	e46	e47
5	1	5	5	5	1	5	5	5	5	5	5	5	5
4	2	3	4	4	1	4	4	4	4	3	5	5	5
5	1	5	5	5	1	5	5	5	5	5	5	5	5
3	2	2	2	3	1	4	3	3	3	3	3	3	3
4	2	4	4	4	1	5	4	4	3	4	4	4	5
5	3	2	5	3	1	4	5	4	5	5	4	5	4
5	1	5	5	5	1	5	5	5	5	5	5	5	5
4	2	4	4	4	2	4	4	3	4	4	4	4	4
3	3	3	3	3	3	3	3	3	3	3	3	3	3
1	3	5	5	4	1	5	5	5	5	5	5	5	5
5	5	5	5	5	1	5	5	5	4	4	4	4	4
3	3	3	5	3	2	4	3	3	3	3	3	3	4
5	1	5	5	5	1	5	5	5	5	5	5	5	5
4	2	3	4	4	2	4	4	3	4	4	4	4	4
5	1	5	5	5	1	5	5	5	5	5	5	5	5
4	4	4	4	4	1	5	3	3	3	3	3	4	4
3	3	3	4	4	1	5	3	3	3	4	4	5	4
4	5	4	4	4	1	4	4	4	4	4	4	4	4
5	2	4	5	4	1	5	5	4	4	4	4	5	5
3	2	3	4	3	1	5	3	2	4	3	4	3	5
5	1	4	5	5	1	5	5	5	5	5	5	5	5
5	5	5	5	5	5	5	5	5	5	5	5	5	5
3	3	3	3	3	3	3	3	3	3	3	3	3	3
5	1	5	5	5	1	4	5	5	5	5	5	5	5
4	1	3	4	4	1	4	3	3	3	4	4	4	4
5	1	5	5	5	1	5	5	5	5	5	5	5	5
3	2	5	4	4	2	5	4	3	3	3	4	5	5
5	2	4	5	4	2	5	4	2	3	3	5	3	4
4	1	4	4	4	1	4	4	4	4	4	4	4	4
5	1	3	4	4	1	5	3	3	2	5	2	3	4
4	2	4	5	4	5	4	4	4	4	4	1	5	3
5	1	5	5	5	1	5	5	5	5	5	5	5	5
4	3	2	4	4	2	4	4	3	3	4	4	4	4
5	2	4	4	4	4	4	4	3	2	3	4	3	3
5	3	4	5	4	1	4	5	5	4	5	5	5	5
5	1	5	5	5	1	5	5	5	5	5	5	5	5
4	2	4	4	4	2	4	4	4	4	4	4	4	4
3	3	3	3	3	3	3	3	3	3	3	3	3	3
5	2	4	4	4	2	4	5	3	2	4	5	4	4
3	2	3	3	3	1	4	3	3	3	3	3	3	3
4	3	3	4	4	1	5	3	3	3	3	3	3	3
3	3	3	3	3	3	3	3	3	3	3	3	3	3
4	3	3	3	5	1	4	3	3	2	3	5	5	4
5	1	5	5	5	1	5	5	5	5	5	5	5	5
4	2	4	4	4	1	4	4	4	4	4	4	4	4
4	2	4	5	5	1	5	4	5	5	5	4	5	5
5	1	5	5	5	1	5	5	5	5	5	5	5	5
5	5	5	5	5	5	5	5	5	5	5	5	5	5
3	2	4	4	4	1	4	4	4	4	4	4	4	4
5	1	4	5	4	1	5	5	5	5	5	5	4	5

5	2	5	5	4	1	5	5	5	5	5	5	3	5
2	3	2	3	3	4	3	3	2	3	4	3	4	3
5	1	4	5	4	1	5	5	5	5	5	5	5	5
5	2	4	4	4	1	5	4	4	4	4	5	5	5
4	1	5	4	5	1	5	5	5	5	5	5	5	5
4	1	4	4	4	1	4	4	4	4	4	4	3	4
4	1	4	4	4	2	4	4	3	4	4	4	5	4
3	3	4	4	4	2	4	3	4	4	4	4	3	4
4	1	4	4	4	1	5	4	5	4	4	4	3	4
5	1	4	4	4	4	4	4	3	3	3	4	4	4
4	3	3	4	3	1	5	3	3	3	3	5	5	5
5	1	4	5	4	1	5	4	4	4	5	2	5	5
5	1	4	5	5	1	5	5	5	5	5	5	5	5
1	5	3	5	5	3	4	5	1	5	5	5	5	5
4	2	4	4	4	1	4	4	4	4	4	4	4	4
4	2	4	4	4	2	4	4	4	4	4	4	4	4
5	1	5	5	5	1	5	5	5	5	5	4	5	5
4	2	4	4	4	1	4	4	4	4	4	4	2	4
3	2	3	4	4	1	3	3	3	3	3	4	4	4
3	2	3	3	3	1	3	4	4	4	4	5	5	4
3	3	4	4	4	2	4	3	4	3	3	4	4	3
3	3	3	4	4	1	4	3	4	5	5	5	5	5
5	1	5	5	5	1	5	5	5	5	5	5	3	5
5	1	5	5	5	1	5	5	5	5	5	5	5	5
3	1	3	3	3	1	3	3	3	3	3	3	3	3
4	2	3	4	3	1	4	3	3	3	3	3	3	3
5	1	5	5	5	1	5	4	4	4	5	5	5	4
4	3	3	5	4	1	4	4	3	3	3	3	5	4
5	2	5	5	5	1	5	5	5	5	5	4	4	5
4	2	4	4	4	1	4	4	4	4	4	4	4	4
4	2	3	4	4	1	5	4	3	4	4	4	4	4
5	2	4	4	4	2	5	5	5	4	4	4	4	4
4	1	3	4	4	1	4	4	3	4	4	4	4	5
3	2	4	5	5	1	5	5	5	5	5	5	5	5
5	2	5	5	5	1	5	5	5	5	5	5	5	5
3	3	3	3	3	3	3	3	3	3	3	3	3	3
5	1	5	5	5	1	5	5	5	5	5	5	5	5
4	2	4	4	4	1	5	4	4	3	3	5	4	4
4	2	3	4	4	2	4	4	4	4	4	4	4	4
5	1	5	5	5	1	5	5	5	5	5	5	5	5
4	2	3	4	3	1	4	4	3	4	4	4	4	5
5	1	5	5	3	1	5	5	5	5	5	5	5	5
4	2	4	4	4	1	5	5	5	4	5	5	4	5
5	1	5	5	5	1	5	5	5	5	5	5	5	5
5	1	5	5	5	1	5	5	5	5	5	5	5	5
5	1	5	5	5	1	4	4	4	4	5	5	5	5
4	2	3	4	3	1	4	4	4	3	3	4	4	2
4	2	4	4	4	1	4	4	4	4	4	5	4	5
5	1	5	5	5	1	5	5	4	4	5	5	5	5
3	2	3	3	3	3	3	3	3	3	3	3	3	3
4	1	4	4	4	1	4	4	4	4	4	4	4	4
5	5	5	5	5	1	5	5	5	5	5	5	5	5
4	2	4	4	4	1	5	4	4	4	4	4	4	4
5	1	5	5	5	1	5	5	5	5	5	5	5	5
4	2	4	4	4	1	5	4	4	3	4	4	4	4
5	1	5	5	5	1	5	5	5	4	5	5	5	4

5	5	5	5	5	5	5	5	5	5	5	5	5	5
3	2	3	3	3	2	4	3	3	3	3	3	4	4
3	3	4	4	4	1	5	4	4	4	5	5	4	5
5	5	5	5	5	1	5	5	5	5	5	5	5	5
4	3	4	4	4	1	5	4	3	4	3	4	5	4
5	1	5	5	5	1	5	5	5	5	5	5	5	5
5	1	4	5	5	1	5	5	5	5	5	5	5	5
5	1	5	5	5	1	4	4	5	4	5	5	5	5
2	4	2	4	3	4	4	3	4	4	4	3	4	4
4	2	4	4	4	1	4	4	4	4	4	4	4	4
5	1	5	5	5	1	5	5	5	5	5	5	5	5
4	2	4	4	4	1	4	4	4	3	4	4	4	4
4	2	3	4	3	2	4	4	4	4	4	4	4	4
5	1	4	5	5	1	5	5	5	5	5	5	5	5
3	3	4	3	3	1	3	3	3	3	3	3	4	4
4	4	4	4	4	1	4	4	3	4	4	4	4	4
5	5	5	5	5	5	5	5	5	5	5	5	5	5
4	3	3	4	3	1	4	4	3	3	3	4	4	4
5	5	5	5	5	5	5	5	5	5	5	5	5	5
5	3	5	5	5	1	5	5	5	5	5	5	5	5
4	2	3	4	4	1	1	4	4	4	4	5	2	5
4	3	3	4	3	3	4	3	3	2	3	4	3	3
3	2	3	4	4	1	4	4	4	3	4	4	4	5
4	2	4	4	4	1	4	4	3	4	4	4	3	4
5	1	4	5	4	1	5	4	4	4	4	5	4	5
4	2	4	4	4	1	4	4	4	4	4	4	4	3
5	1	5	5	5	1	5	5	5	5	5	5	5	5
4	2	3	4	4	1	4	4	4	3	4	1	3	4
5	1	5	5	5	1	5	5	5	5	5	5	5	5
3	3	2	3	4	1	5	3	2	2	2	4	4	3
3	2	3	3	3	2	3	3	3	3	3	3	3	3
4	3	2	4	4	1	4	4	3	3	3	4	2	4
3	3	3	3	3	3	4	4	3	4	4	4	4	4
4	2	3	3	1	1	3	2	2	2	2	3	4	4
5	2	5	5	5	1	5	5	5	5	5	5	5	5
4	2	4	5	5	1	5	5	5	5	5	4	5	5
4	3	2	4	4	1	4	4	4	2	2	4	4	4
5	1	4	5	4	1	5	4	3	4	4	4	4	5
5	2	2	5	5	1	4	5	5	5	5	5	5	5
1	3	1	3	3	1	5	1	1	5	1	5	3	5
3	2	4	4	3	1	4	3	3	3	3	3	2	3
4	4	3	4	4	1	5	5	3	4	4	1	3	5
5	3	2	3	3	1	3	5	3	3	3	3	3	3
5	2	4	5	5	1	5	4	4	3	4	5	4	5
3	3	3	3	3	3	3	3	3	3	3	3	3	3
4	1	4	4	4	1	4	4	4	4	4	4	4	4
3	3	3	3	3	1	5	5	2	1	2	2	5	4
3	1	3	3	3	1	5	3	3	3	3	4	4	5
5	1	5	5	5	1	5	5	5	5	5	5	5	5
5	5	5	5	5	5	5	5	5	5	5	5	5	5
5	1	5	5	5	1	5	5	5	5	5	5	5	5
5	1	4	5	5	1	5	5	5	5	5	4	4	5
4	3	4	4	4	2	4	4	4	4	4	4	4	4
5	2	5	5	5	1	5	5	5	5	5	5	5	5
5	4	4	5	3	1	5	5	3	5	4	4	4	4
4	4	4	4	4	4	4	4	4	4	4	4	4	4

5	1	4	4	5	1	5	5	5	5	5	4	5	5
4	1	5	5	5	1	5	5	4	5	5	5	4	5
5	1	4	5	5	1	5	5	5	5	5	5	2	4
4	4	5	4	4	5	4	5	4	5	5	4	4	5
5	1	5	5	5	5	5	5	5	5	5	5	5	5
5	2	3	5	5	1	5	5	5	2	4	3	2	5
5	1	5	5	5	1	5	5	5	5	5	5	5	5
4	4	4	4	4	4	4	4	4	4	4	4	4	4
3	2	2	4	4	2	4	4	3	3	3	4	4	4
5	1	5	5	5	1	5	5	5	5	5	5	5	5
5	1	5	5	5	1	5	5	5	5	5	5	5	5
4	2	3	4	4	1	5	3	4	4	4	4	4	4
2	5	1	4	1	3	4	3	2	1	1	4	4	4
5	4	4	4	4	1	4	4	3	3	4	4	4	5
5	1	5	5	5	1	5	5	4	5	5	5	5	5
5	2	3	3	4	1	5	5	3	5	5	5	5	5
5	1	5	5	5	1	5	5	5	5	5	5	5	5
3	3	3	3	3	3	3	3	3	3	3	3	3	3
5	2	4	4	4	1	5	4	5	5	5	5	4	5
5	4	4	5	5	1	5	5	3	4	5	5	5	5
4	2	4	4	5	2	4	4	5	4	4	5	4	5
5	3	2	5	5	1	5	4	4	4	3	5	5	5
5	1	4	4	4	1	4	4	4	4	5	5	5	4
3	3	3	3	3	3	3	3	3	3	3	3	3	3
4	3	4	4	4	2	4	4	4	4	4	4	4	4
4	3	4	4	4	3	4	4	4	4	4	4	4	4
5	1	5	5	5	1	5	5	5	5	5	5	5	5
5	2	4	5	5	3	4	4	4	5	5	4	3	4
5	1	4	5	5	3	5	3	3	3	3	3	5	5
4	4	3	4	4	4	4	5	5	4	4	4	4	5
4	2	4	4	5	1	4	4	3	3	3	5	5	5
5	1	5	5	5	1	5	5	5	5	5	5	5	5
5	1	5	5	5	1	5	5	5	5	5	5	5	5
5	1	5	5	5	1	5	5	5	5	5	5	5	5
5	2	5	5	5	1	5	5	4	5	5	5	4	4
4	2	4	4	4	2	4	4	4	4	4	4	4	5
3	3	3	3	3	2	3	3	3	3	3	3	3	3
5	1	4	4	4	1	5	5	5	5	5	4	4	3
4	2	4	4	4	1	4	4	3	3	3	3	4	3
4	2	4	4	4	1	5	4	4	4	4	4	4	4
4	3	3	3	3	3	3	3	3	3	3	3	3	3
5	1	5	5	5	1	5	5	5	5	5	5	5	5
4	2	4	4	4	3	4	4	4	4	4	4	4	4
4	2	3	4	4	1	4	5	4	5	4	4	4	5
4	2	4	3	4	2	4	3	3	3	4	4	4	4
4	4	4	4	4	1	4	4	4	4	4	4	4	4
4	2	4	4	4	1	4	4	4	4	4	4	4	4
4	1	4	4	4	2	4	4	4	4	4	4	4	4
4	2	4	4	5	1	4	4	4	4	4	4	4	4
4	1	4	4	4	1	4	4	4	4	4	4	4	4
5	1	5	5	5	1	5	5	5	5	5	5	5	5
5	1	5	5	5	1	5	5	5	5	5	5	5	5
5	2	5	5	5	1	5	5	5	5	5	5	5	5
4	2	4	4	4	1	4	3	3	3	3	4	3	4
4	2	4	4	4	1	4	4	4	4	4	4	4	4
5	1	4	5	4	1	5	4	4	4	4	4	5	4

4	1	3	4	4	1	4	4	4	4	4	4	4	4
4	2	3	4	3	2	4	3	3	4	4	4	4	4
5	5	5	5	5	5	5	5	5	5	5	5	5	5
4	3	2	4	2	1	5	3	3	3	3	5	5	5
5	1	5	5	5	1	5	5	5	5	5	5	5	5
5	1	5	5	5	1	5	5	5	5	5	5	5	5
3	2	4	5	5	1	5	3	4	4	3	2	4	5
5	2	5	5	5	1	5	5	5	4	5	3	5	5
5	1	4	5	4	1	4	4	5	4	4	5	3	5
4	4	4	4	4	4	4	4	4	4	4	4	4	4
5	1	4	4	4	1	5	5	5	5	5	5	5	5
5	1	4	4	4	1	5	4	4	4	4	4	4	4
4	2	4	4	4	2	4	4	4	4	4	4	4	4
4	4	4	4	4	4	4	4	4	4	4	4	4	4
5	1	5	5	5	1	5	5	5	5	5	5	5	5
5	5	5	5	5	5	5	5	5	5	5	5	5	5
5	5	5	5	5	5	5	5	5	5	5	5	5	5
5	1	3	5	4	1	5	3	3	3	4	4	3	5
4	2	4	4	4	2	4	4	3	4	4	3	3	4
5	1	5	5	5	1	5	5	5	5	5	5	5	5
5	1	4	5	5	1	4	4	4	4	4	4	5	5
4	2	3	4	3	1	5	3	5	3	4	5	5	4
5	1	4	5	5	1	5	5	5	5	5	4	5	5
5	1	5	5	5	1	4	5	5	5	5	5	5	5
5	1	3	5	5	2	5	5	5	5	5	5	5	5
4	4	4	4	4	1	4	4	4	4	4	4	4	4
5	1	5	5	5	1	5	5	5	5	5	5	5	5
4	3	3	4	4	3	4	4	3	4	4	4	4	4
5	1	4	4	4	1	4	4	4	4	4	4	4	4
4	3	3	4	4	2	4	4	4	5	4	5	5	4
5	1	5	4	5	2	4	4	4	4	4	4	5	5
5	1	5	5	5	5	5	5	5	5	5	5	5	5
5	1	5	5	5	1	5	5	5	5	5	5	5	5
3	2	4	4	4	1	5	4	4	4	5	4	4	5
5	1	4	5	4	1	5	5	4	5	5	5	5	5
5	1	5	5	5	4	4	4	4	4	4	4	4	4
5	1	4	5	5	1	5	4	3	3	4	5	5	5
5	1	5	5	5	1	5	5	5	5	5	5	5	5
5	2	4	5	5	1	5	5	4	5	5	5	5	5
5	1	4	5	4	1	5	5	4	5	4	5	5	5
5	1	5	5	5	1	5	5	5	5	5	5	5	5
5	1	5	5	5	1	5	5	5	5	5	5	5	5
5	1	5	5	5	1	5	5	5	5	5	1	5	5
4	2	4	4	4	1	4	4	3	3	4	5	4	5
4	2	4	4	4	4	4	4	4	4	4	4	3	4
5	2	5	5	5	1	5	5	5	5	5	5	5	5
5	2	5	5	5	1	5	5	4	3	3	5	5	5
4	2	3	4	3	3	4	3	3	2	4	4	4	4
3	3	3	4	4	2	3	3	3	3	3	3	4	4
3	3	3	3	3	2	3	3	3	3	3	3	4	3
4	2	4	5	5	1	5	5	5	5	5	5	5	5
4	3	4	4	4	3	4	4	4	4	4	4	4	4
5	1	4	5	4	1	5	4	5	5	5	5	5	5
5	1	5	5	5	1	5	4	4	5	5	5	5	5
4	2	4	4	4	2	4	4	4	4	4	4	4	4
3	2	3	3	3	2	3	3	3	3	3	3	3	3

5	1	5	5	5	1	5	5	5	5	5	5	5	5
4	1	4	4	4	1	4	4	4	4	4	5	5	5
5	1	5	5	5	1	5	5	5	5	5	5	5	5
5	2	5	5	5	1	5	5	5	5	5	5	5	5
5	1	5	5	5	1	5	5	5	5	5	5	5	5
4	4	4	4	4	2	4	4	4	4	4	4	4	4
5	5	5	5	5	5	5	5	3	5	5	5	3	3
3	3	2	4	3	2	4	3	3	2	3	4	4	5
3	3	2	3	3	2	4	3	2	3	3	3	3	4
5	1	5	5	5	1	5	5	5	5	5	5	5	5
4	4	4	4	4	2	4	4	3	4	4	4	4	4
5	1	5	5	5	1	5	4	4	4	4	4	4	4
3	5	5	5	2	1	5	3	1	1	2	5	2	5
5	1	3	5	5	1	4	5	5	2	5	4	5	5
4	4	5	4	4	4	5	4	5	4	4	5	4	4
5	5	5	5	5	5	5	5	5	5	5	5	5	5
5	1	4	4	5	1	5	5	4	5	5	5	5	5
3	3	3	3	3	3	3	3	3	3	3	3	3	3
3	3	3	4	4	2	4	3	3	3	3	4	4	4
5	2	5	5	5	1	5	5	5	5	5	5	5	5
3	2	4	4	4	2	4	4	4	4	4	4	4	4
5	5	5	5	5	1	5	5	4	4	4	4	4	4
4	2	3	5	4	1	5	4	4	4	4	4	4	4
5	3	4	5	5	1	5	5	5	5	5	5	5	5
4	2	4	4	4	1	5	4	4	4	4	4	4	5
4	2	4	5	4	1	5	4	5	5	5	5	5	5
5	2	4	4	4	4	4	4	4	4	4	4	4	4
4	2	3	5	4	1	5	5	5	5	5	5	5	5
4	4	4	4	4	4	4	4	4	4	4	4	4	4
5	1	5	5	5	1	5	5	5	5	5	5	5	5
4	1	4	4	4	1	4	4	5	5	4	4	4	4
4	2	3	4	4	2	4	4	4	4	4	4	4	4
5	1	5	5	5	1	5	5	5	5	5	5	5	5
3	3	2	4	2	3	2	3	3	3	3	4	3	3
5	1	5	5	5	1	5	5	5	5	5	5	5	5
5	1	5	5	5	1	5	5	5	5	5	5	5	5
5	1	5	5	5	1	5	5	5	5	5	5	5	5
4	2	5	5	4	1	5	5	4	5	5	5	3	5
5	2	4	4	4	1	5	4	5	4	4	4	4	5
5	2	4	4	4	1	5	4	5	4	4	4	4	5
5	1	3	4	4	1	4	3	5	4	4	3	4	4
5	1	5	5	5	1	5	5	5	5	5	5	5	5
4	1	4	4	4	1	4	4	4	4	4	4	4	4
5	3	2	3	4	2	4	4	4	4	4	4	3	4
5	2	4	5	4	1	5	5	3	3	4	5	3	5
5	1	5	5	5	1	5	5	5	5	5	5	5	5
5	5	5	5	5	5	5	5	5	5	5	5	5	5
5	1	5	5	5	1	5	5	5	5	5	5	4	5
5	1	5	5	5	5	5	5	5	5	5	5	5	5
4	2	4	4	5	1	5	4	4	4	4	5	5	5
3	3	3	5	3	1	5	4	3	4	4	5	5	5
1	1	4	3	4	4	1	1	3	1	3	3	5	1
4	2	3	4	4	1	4	4	4	2	3	4	3	4
4	3	4	4	4	1	4	4	4	4	4	4	4	4
3	3	3	3	3	3	3	3	3	3	3	3	3	3
5	1	5	5	5	1	5	5	5	5	5	5	5	5

5	1	5	5	5	1	5	5	5	5	5	5	5	5
4	1	4	4	4	1	5	4	4	4	4	4	4	4
5	1	5	5	5	1	5	5	5	5	5	5	5	4
4	1	4	4	4	1	4	4	4	4	4	4	4	4
4	1	5	4	4	1	5	5	4	5	4	5	5	5
4	2	4	4	4	1	4	4	4	4	4	4	4	4
5	2	5	5	5	2	5	5	5	5	5	5	5	5
3	2	3	3	3	2	2	3	3	3	3	3	3	3
4	2	4	4	4	1	4	4	4	4	4	4	4	4
3	3	3	3	3	3	3	3	3	3	3	3	3	3
4	1	4	4	4	1	4	4	4	3	4	4	4	4
5	1	5	5	5	3	4	5	5	5	5	5	5	5
5	5	5	5	5	5	5	5	5	5	5	5	5	5
4	4	4	4	4	4	4	4	4	4	4	4	4	4
5	1	5	5	5	1	5	5	5	5	5	5	5	5
5	1	4	5	5	1	5	5	5	5	5	5	3	5
5	1	5	5	5	1	5	5	5	5	5	5	5	5
5	1	5	5	5	1	5	5	5	5	4	5	5	5
5	1	4	5	4	1	5	5	4	5	4	4	5	5
5	5	5	5	5	1	4	5	5	5	5	5	5	5
5	1	4	4	4	1	4	4	5	5	5	5	5	5
4	2	4	4	4	1	4	4	4	4	4	4	4	4
4	2	4	4	4	2	4	4	4	4	4	4	4	4
4	2	4	4	4	1	4	4	4	4	4	4	4	3
5	1	5	5	5	1	5	5	5	5	5	5	5	5
4	1	4	4	4	1	4	4	4	4	4	4	4	5
4	2	4	4	4	2	5	4	4	4	4	4	5	5
5	1	5	5	5	1	5	5	5	5	5	5	5	5
5	5	5	5	5	1	5	5	5	5	5	5	5	5
4	3	4	4	4	2	4	3	4	4	4	3	3	3
4	4	4	4	4	4	4	5	4	4	4	4	4	5
2	3	3	4	4	2	4	3	3	3	3	4	3	3
5	1	5	5	5	1	5	5	5	5	5	5	5	5
4	1	4	4	4	1	4	4	4	4	4	4	2	4
3	2	3	3	3	1	3	3	3	3	3	3	3	3
5	4	5	4	3	4	4	4	5	4	4	5	4	5
5	1	5	5	5	1	5	5	3	1	4	3	5	5
4	2	4	4	4	1	4	4	4	4	4	4	4	4
4	2	4	5	4	2	4	4	4	4	4	4	4	4
5	1	5	5	5	1	5	5	5	5	5	5	3	5
5	2	5	5	5	2	5	5	4	5	4	4	4	5
5	1	3	4	4	4	4	4	4	3	4	4	4	4
5	1	5	5	5	1	5	5	5	5	5	5	5	5
4	4	4	4	4	2	4	4	4	4	4	4	4	4
5	1	5	5	5	1	5	5	5	5	5	5	5	5
5	1	5	5	5	1	5	5	5	5	5	5	5	5
4	1	4	4	3	1	5	5	4	3	4	4	4	4
3	3	4	3	3	1	3	3	3	3	3	3	2	3
5	5	5	5	5	1	5	5	5	5	5	4	4	5
4	2	4	4	4	2	4	4	4	4	4	4	4	4
4	1	5	5	4	1	4	4	4	4	5	5	5	5
4	2	4	5	5	1	4	3	4	4	4	4	5	5
5	5	5	5	5	5	5	5	5	5	5	5	5	5
4	2	4	4	4	2	5	4	4	3	4	4	4	5
5	1	5	5	5	1	5	5	5	5	5	5	5	5
3	3	3	3	3	2	5	3	3	3	3	3	3	3

3	3	3	3	3	1	3	3	3	3	3	3	5	3
5	1	5	5	5	1	5	5	5	5	5	5	5	5
5	1	5	5	5	1	5	5	5	5	5	5	5	5
5	1	4	5	5	1	5	5	3	4	5	5	5	5
4	2	4	4	4	2	4	4	4	4	4	4	4	4
4	2	4	4	4	1	5	5	5	5	5	5	5	5
5	2	3	5	3	2	5	5	2	5	3	3	5	3
5	1	4	4	4	1	5	4	4	4	4	4	4	5
5	1	5	5	5	1	5	5	5	5	5	5	5	5
4	1	4	4	4	1	4	4	4	4	4	4	4	4
5	1	4	4	5	2	4	4	5	5	4	4	4	4
4	2	4	4	4	1	4	4	4	4	4	4	4	4
3	2	2	4	4	1	3	3	3	3	3	3	4	4
5	1	5	5	5	1	5	5	5	5	5	5	5	5
3	3	3	4	4	3	4	3	3	4	3	3	4	3
3	1	3	3	3	3	3	3	3	3	3	3	4	3
4	2	4	4	4	2	4	4	3	4	4	4	3	4
5	1	5	5	5	1	5	5	5	5	5	5	5	5
5	5	5	5	5	1	5	5	5	5	5	5	5	5
4	2	4	4	4	2	4	4	4	4	4	4	4	4
5	1	5	5	5	1	5	5	5	5	5	5	5	5
4	2	3	4	3	1	4	3	3	3	3	3	3	3
5	1	5	5	5	1	5	5	5	5	5	5	5	5
5	1	5	5	5	1	5	5	5	5	5	5	5	5
3	2	3	4	4	2	4	3	3	2	3	4	3	4
4	1	4	4	4	1	5	4	3	3	4	4	4	3
5	2	5	5	5	2	5	5	5	5	5	5	5	5
4	2	4	4	4	2	4	4	3	3	3	4	3	3
3	4	3	5	5	5	5	5	5	5	5	3	5	4
4	4	4	4	2	2	3	4	2	2	3	4	3	2
3	3	3	3	3	3	3	3	3	3	3	3	3	3
4	1	5	5	5	1	4	4	4	4	4	4	4	4
5	1	5	5	5	1	5	4	4	4	4	4	5	5
4	1	4	5	4	1	5	5	5	5	5	5	5	5
2	3	2	3	4	1	4	3	3	2	3	4	4	3
5	1	5	5	5	1	5	5	5	5	5	5	5	3
5	2	3	5	5	1	5	3	4	3	4	5	4	5
5	2	5	5	5	2	5	5	5	5	5	5	5	5
4	2	4	4	4	1	5	4	4	4	4	4	4	4
5	1	4	5	5	1	5	5	5	5	5	5	5	5
3	3	4	4	4	3	3	3	4	3	3	4	4	4
5	5	5	5	5	1	5	5	5	5	5	5	5	5
5	1	5	5	5	1	5	5	5	5	5	5	5	5
4	2	4	4	4	2	4	4	4	4	5	4	5	4
4	4	4	4	4	4	4	4	4	4	4	4	4	4
4	1	4	4	3	1	4	4	4	4	4	4	4	4
5	1	5	5	5	1	5	5	5	5	5	5	5	5
3	3	3	3	3	2	3	3	3	3	3	3	3	3
5	5	5	5	5	5	5	5	5	5	5	5	5	5
5	1	5	5	5	1	5	5	5	5	5	5	5	5
4	2	4	4	4	1	5	4	4	4	4	4	4	4
5	1	5	5	5	1	5	5	5	5	5	5	5	5
5	5	5	5	5	1	5	5	5	5	5	5	5	5
5	1	4	5	5	1	5	5	5	5	5	4	5	5
5	1	5	5	5	1	5	5	5	5	5	5	5	5
5	2	5	5	5	2	4	4	5	5	5	3	4	5

4	1	3	5	4	1	5	3	3	4	5	4	4	4
5	1	5	5	5	1	5	5	5	5	5	5	5	5
4	1	4	4	5	1	4	5	5	4	4	5	4	4
4	2	4	4	4	1	3	4	3	3	3	3	5	3
4	2	4	4	4	2	4	4	4	4	4	4	4	4
4	1	4	5	4	1	5	4	4	4	4	5	5	5
4	2	4	4	4	1	5	4	4	4	4	4	4	4
5	2	5	5	5	2	5	5	5	4	5	4	5	5
4	2	4	4	4	3	3	3	3	3	4	3	3	3
5	1	5	5	5	2	5	5	5	5	5	5	5	5
5	1	5	5	5	1	5	5	1	5	5	5	5	5
4	1	4	5	4	1	5	4	5	4	4	4	5	5
5	1	5	5	5	1	5	5	5	5	5	5	5	5
3	3	3	3	3	3	3	3	3	3	3	3	3	3
5	1	5	5	5	1	5	5	5	5	5	5	5	5
5	2	4	4	4	1	5	5	4	5	5	5	5	5
5	1	5	5	5	1	5	5	5	5	5	5	5	5
4	1	3	4	4	1	4	4	4	4	4	4	4	4
3	3	3	3	3	3	3	3	3	3	3	3	3	3
5	5	5	5	5	5	5	5	5	5	5	5	5	5
4	1	4	5	5	1	4	4	4	5	5	4	4	4
4	2	4	4	4	1	4	4	4	4	4	3	4	4
5	2	4	4	5	2	4	4	3	3	5	5	5	5
4	2	3	4	4	2	4	4	4	4	4	4	4	4
3	3	2	3	3	4	3	4	3	4	3	3	4	3
3	2	3	3	3	1	3	3	3	3	3	3	3	3
4	1	4	5	5	1	5	4	3	3	4	5	4	5
3	3	3	3	3	3	3	3	3	3	3	3	3	3
5	1	4	5	5	1	5	5	5	5	5	5	5	5
4	2	4	4	4	1	5	4	3	3	3	4	4	4
5	1	4	5	4	1	5	5	5	5	5	5	5	5
4	2	3	3	3	1	3	3	3	3	3	3	2	3
5	1	5	5	5	1	5	5	5	5	5	5	4	5
4	2	3	4	4	1	5	5	4	4	4	4	4	4
3	5	3	4	4	3	4	4	3	3	5	5	5	3
4	2	3	5	3	1	5	3	3	2	3	4	4	4
5	1	4	5	5	1	5	5	3	5	5	5	5	4
4	2	4	4	4	2	4	4	4	4	4	4	4	4
4	2	3	4	4	1	4	4	2	4	4	4	3	4
5	2	3	4	4	2	4	4	4	4	4	4	4	4
5	1	5	5	5	2	5	5	5	5	5	3	4	5
5	1	5	5	5	1	5	5	5	5	5	5	5	5
5	1	5	5	5	1	4	5	5	5	5	5	5	5
5	1	4	5	5	1	5	4	5	5	5	5	5	5
4	1	4	4	4	1	4	4	3	4	4	5	4	4
3	2	4	4	4	1	3	3	3	3	3	4	4	4
5	2	4	5	5	1	5	5	5	5	5	5	5	5
5	1	5	5	5	1	5	5	5	5	5	5	5	5
3	2	4	4	3	1	3	3	3	3	3	3	3	3
5	1	5	5	5	1	5	5	5	5	5	5	5	5
4	4	4	4	4	2	4	4	4	4	4	4	4	4
5	2	5	5	5	2	5	5	5	5	5	5	5	5
4	2	3	4	4	1	5	5	5	5	5	5	5	5
4	1	4	4	4	1	4	4	4	4	4	4	4	4
4	4	4	4	4	1	4	4	4	4	4	4	4	4
3	3	3	3	3	3	3	3	3	3	3	3	3	3

4	2	4	4	4	1	4	4	4	4	4	4	4	4
5	1	5	5	5	1	5	5	5	5	5	5	5	5
5	5	5	5	5	5	5	5	5	5	5	5	5	5
3	1	3	3	3	2	3	3	3	3	3	3	4	3
5	1	5	5	5	1	5	5	5	5	5	5	5	5
4	1	4	4	4	1	4	4	4	4	4	4	4	4
3	3	3	3	3	3	3	3	3	3	3	3	3	3
5	2	3	3	3	3	3	3	3	3	3	3	3	3
4	1	3	4	3	1	5	3	4	4	3	4	4	4
5	1	5	5	5	1	5	5	5	4	4	5	5	5
5	1	4	4	4	2	4	4	3	4	4	3	5	5
5	1	5	5	5	1	4	4	4	4	4	4	4	4
5	3	5	5	5	3	5	5	5	5	5	5	5	5
5	1	5	5	5	1	5	5	5	5	5	5	5	5
5	4	4	5	5	1	5	5	5	4	5	4	5	5
4	1	4	5	5	1	5	4	4	4	4	5	5	5
4	4	2	4	3	2	3	2	3	2	2	4	4	4
4	2	4	4	4	1	4	4	3	4	4	4	3	4
5	1	5	5	5	2	3	5	5	5	5	5	5	5
5	5	5	5	5	5	5	5	5	5	5	5	5	5
4	2	3	4	3	1	4	3	3	3	4	5	4	4
2	3	2	3	2	3	4	4	2	4	2	2	4	3
5	5	5	5	5	1	5	5	4	5	5	5	5	5
4	2	4	4	4	2	4	4	3	3	4	4	4	3
5	1	5	5	5	1	5	5	5	5	5	5	5	5
5	1	4	4	4	1	5	4	4	4	4	4	4	4
5	1	5	5	5	1	4	5	5	5	5	5	3	5
4	4	4	4	4	4	4	4	4	4	4	4	4	4
5	2	5	5	5	2	5	5	5	5	5	5	5	5
5	2	4	4	4	1	5	4	4	5	5	4	5	5
5	2	4	4	4	1	5	3	4	4	4	4	3	5
3	3	3	3	3	3	3	3	3	3	3	3	3	3
4	2	3	4	4	1	3	4	4	2	4	2	4	4
5	1	4	5	5	1	5	5	4	5	5	5	5	5
5	1	5	5	5	1	5	5	5	5	5	5	5	5
4	1	4	4	4	1	5	4	4	4	4	4	4	4
4	2	4	4	4	2	4	4	3	4	4	4	4	4
5	1	5	5	5	1	5	5	5	5	5	5	5	5
5	2	4	5	5	1	5	3	3	5	3	2	4	4
4	2	4	4	5	2	5	5	5	5	4	3	4	5
4	2	4	4	4	2	4	4	4	4	4	4	4	4
5	2	5	5	5	2	4	4	5	5	5	5	5	5
5	1	5	5	5	1	5	5	5	5	5	3	5	5
4	2	4	4	4	1	4	4	4	4	4	3	4	4
4	2	4	4	4	2	4	4	4	4	4	4	4	4
4	2	3	4	4	2	4	4	4	4	4	4	4	4
5	1	5	5	5	1	5	5	5	5	5	5	5	5
5	2	5	5	5	1	5	5	5	5	5	5	5	5
4	1	4	4	4	1	4	4	5	5	5	3	5	5
5	1	5	5	5	1	5	5	5	5	5	5	5	5
5	1	5	5	5	1	5	5	5	5	5	5	5	5
5	1	5	5	5	1	5	5	5	5	5	5	5	5
5	1	4	5	5	1	5	5	5	5	5	5	5	5
5	1	4	5	5	1	5	4	5	5	5	5	5	5
4	1	4	4	4	1	4	4	4	4	4	4	4	4
5	2	4	5	5	1	5	5	5	5	5	5	5	5

4	3	3	3	3	1	3	2	3	2	2	2	3	2
5	3	5	5	5	1	5	5	5	5	5	5	5	5
5	5	5	5	5	1	5	5	5	5	5	5	5	5
5	1	5	5	5	1	5	5	5	5	5	5	5	5
3	4	3	4	3	4	3	3	4	3	3	4	3	4
3	3	3	3	3	3	3	3	3	3	3	3	3	3
5	5	5	5	5	5	5	5	5	5	5	5	5	5
4	1	5	5	3	1	5	5	5	5	5	5	5	5
3	3	3	3	3	3	3	3	3	3	3	3	3	3
4	4	4	4	4	2	4	4	4	4	4	4	4	4
4	5	3	3	3	1	5	4	5	3	3	5	5	5
5	3	3	5	4	1	5	3	4	3	4	4	4	4
4	2	4	4	4	2	4	4	4	4	4	4	4	4
4	4	4	4	4	2	4	4	4	4	4	4	4	4
5	2	4	5	5	2	5	5	4	5	5	5	5	5
4	2	4	4	4	2	4	4	4	4	4	4	4	4
4	1	3	4	4	1	4	4	5	4	4	4	5	5
5	1	5	5	5	1	5	5	5	5	5	4	5	5
5	3	5	5	5	1	5	5	5	5	5	5	5	5
3	3	3	3	4	1	4	4	4	4	4	4	4	4
4	4	4	4	4	1	4	4	4	4	4	4	4	4
5	1	5	5	5	5	5	5	5	5	5	3	5	5
4	1	4	4	4	1	4	4	4	4	4	4	4	4
5	1	5	5	5	1	5	5	5	5	5	5	5	5
5	1	5	5	5	1	5	5	5	5	5	5	5	5
4	2	4	4	4	2	4	4	4	4	4	4	4	4
3	3	3	3	3	3	3	3	3	3	3	3	3	3
4	1	4	4	4	3	2	5	4	4	3	4	4	4
4	3	3	4	4	1	4	4	4	3	4	4	4	4
5	1	5	5	5	1	5	5	5	5	5	5	5	5
4	2	3	4	4	1	4	3	3	3	3	3	4	4
5	1	5	5	5	1	5	5	5	5	5	5	5	5
5	2	5	5	5	1	5	5	5	5	5	5	1	5
4	4	4	4	4	4	4	4	4	4	4	4	4	4
5	5	5	5	5	1	5	5	5	5	5	5	5	5
4	2	4	4	4	1	4	4	4	4	4	4	4	4
5	1	5	5	5	1	5	5	5	5	5	5	5	5
4	2	4	4	3	1	5	3	3	3	3	3	4	4
5	2	4	5	5	1	4	4	5	4	5	4	4	5
3	4	3	4	3	1	5	4	4	4	4	1	4	5
4	2	4	4	4	1	5	4	4	4	4	4	4	4
5	1	5	5	5	1	5	5	5	5	5	5	5	5
4	4	4	4	4	1	5	5	5	5	5	5	5	5
4	2	4	4	5	1	5	5	5	5	5	5	5	5
5	1	5	5	5	1	5	5	5	5	5	5	5	5
4	1	4	4	4	1	4	4	3	4	4	4	4	4
4	4	4	4	3	3	4	3	3	3	3	3	4	4
5	1	3	5	3	1	5	5	5	5	5	5	5	5
4	2	4	4	4	2	4	4	4	4	4	4	4	4
5	1	5	5	5	1	5	5	5	5	5	5	5	5
5	1	4	5	5	1	5	5	5	5	5	3	5	5
4	2	4	4	4	1	5	4	4	4	5	3	4	4
5	1	5	5	5	1	5	5	5	5	5	5	5	5
5	3	2	4	5	3	3	5	3	3	3	4	5	5
5	5	5	5	5	1	5	5	5	5	5	5	5	5
4	4	4	4	4	4	4	4	4	4	4	4	4	4

5	1	5	5	5	1	5	5	5	5	5	5	5	5
5	1	4	4	4	1	4	4	4	5	4	4	5	5
4	2	4	4	4	1	4	4	3	4	4	4	4	4
5	1	5	5	5	1	5	5	5	5	5	5	5	5
5	1	5	5	5	1	5	5	5	5	5	4	5	5
5	1	5	5	5	1	5	5	5	5	5	5	5	5
2	3	3	3	3	2	3	3	2	2	3	3	1	2
5	2	2	4	3	3	2	3	2	4	2	3	4	3
5	1	3	5	3	1	5	5	5	5	5	5	5	5
5	1	5	5	5	1	5	5	5	5	5	5	5	5
5	1	5	5	5	1	5	5	5	5	5	5	5	5
4	1	4	4	4	2	5	4	5	5	5	5	5	5
5	1	5	5	5	1	5	5	5	5	5	5	5	5
4	3	4	4	4	3	4	4	4	4	4	4	4	4
5	5	5	5	5	1	5	5	5	5	5	5	5	5
4	2	3	4	4	1	5	3	4	4	4	4	4	4
5	1	5	5	5	1	5	5	5	5	5	5	5	5
3	1	4	5	3	1	5	3	2	2	3	3	3	4
5	5	5	5	5	5	5	5	5	5	5	5	5	5
5	1	5	5	5	1	5	5	5	5	5	5	5	4
5	5	5	5	5	4	4	4	4	4	4	4	4	4
5	5	5	5	5	5	5	5	5	5	5	5	5	5
3	2	3	3	3	2	3	3	3	3	3	3	4	4
3	3	3	3	3	3	3	3	3	3	3	3	3	3
4	2	4	4	4	1	4	4	4	4	4	4	4	4
5	1	5	5	5	1	5	5	5	5	5	5	5	5
4	1	5	5	5	1	5	5	4	4	5	5	5	5
3	5	1	3	3	1	4	2	2	2	2	4	4	2
4	2	4	4	4	2	4	4	4	4	4	4	4	4
4	3	3	4	3	2	4	3	3	3	3	4	4	4
5	1	5	5	5	1	5	5	5	5	5	5	5	5
4	2	4	4	4	4	4	4	4	4	4	4	4	4
5	1	5	5	5	1	5	5	5	5	5	5	5	5
3	2	3	3	3	2	5	4	2	3	3	3	4	4
4	2	4	4	4	2	4	4	4	4	4	4	4	4
3	3	3	4	4	1	4	4	4	4	4	4	4	5
4	3	3	3	4	3	4	3	3	3	3	4	4	4
3	4	2	4	2	2	4	4	4	4	4	4	4	4
5	1	5	5	5	1	5	5	5	5	5	5	5	5
5	5	5	5	5	5	5	5	5	5	5	5	5	5
4	2	4	4	4	1	4	4	4	4	4	4	4	4
3	2	3	4	3	2	4	3	4	4	3	4	3	4
3	2	4	3	3	1	3	3	3	3	3	3	3	3
5	1	5	5	5	1	5	5	5	5	5	5	5	5
3	3	3	3	3	3	3	3	3	3	3	3	3	3
4	2	3	4	4	2	4	4	4	4	4	4	4	4
4	2	4	4	4	2	4	3	4	5	4	4	4	4
4	2	4	4	4	1	4	4	4	4	4	3	4	4
4	3	3	4	4	2	4	4	4	4	4	4	4	4
5	1	5	5	5	1	5	5	5	5	5	5	5	5
4	3	3	4	5	1	5	5	5	4	5	5	4	5
4	2	4	4	4	1	4	4	4	4	4	3	4	4
5	1	5	5	5	1	5	5	5	5	5	5	5	5
5	1	5	5	5	1	5	5	5	5	5	5	5	5
5	1	5	5	5	1	5	5	3	5	5	4	5	5
5	1	5	5	5	1	5	5	5	5	5	5	5	5

5	1	4	3	5	1	5	5	5	5	4	5	5	4
5	1	5	5	5	1	5	5	5	5	5	5	5	5
5	1	5	5	5	1	5	5	5	5	5	5	5	5
3	3	3	3	3	1	5	4	4	3	4	4	5	5
5	1	5	5	5	1	5	5	5	5	5	5	5	5
4	1	3	4	4	1	4	3	3	3	3	4	4	5
5	1	5	5	5	1	5	5	5	5	5	5	4	4
3	2	2	3	3	1	3	3	2	2	2	3	3	5
5	2	5	5	5	1	5	5	2	5	5	5	5	5
5	1	4	1	4	1	4	2	3	1	2	5	5	3
5	1	5	5	5	1	5	5	5	5	5	1	5	5
5	5	5	4	5	1	4	4	4	5	4	5	5	5
5	1	4	5	5	1	5	5	5	5	5	5	5	5
5	1	5	5	5	1	5	5	5	5	5	5	5	5
5	1	5	5	5	1	5	5	5	5	5	5	5	4
4	1	4	5	5	1	5	5	5	5	5	5	5	5
5	1	5	5	5	1	5	5	5	5	5	5	5	5
4	2	4	4	4	1	4	4	4	4	4	4	2	4
5	5	5	5	5	5	5	5	5	5	5	5	5	5
4	1	4	4	4	1	4	4	4	4	4	3	4	4
5	4	2	5	5	1	5	5	5	5	5	5	5	5
4	4	4	4	4	4	4	4	4	4	4	4	4	4
5	1	5	5	5	1	5	5	5	5	5	5	5	5
4	1	4	5	4	1	5	4	4	4	5	5	4	5
4	1	4	4	4	1	4	4	4	4	4	4	4	4
5	1	5	5	5	1	5	5	5	5	5	5	5	5
5	5	5	5	5	5	5	5	5	5	5	5	5	5
5	1	5	5	5	1	5	5	5	5	5	5	5	5
5	1	4	4	5	1	5	5	5	5	5	5	4	5
4	2	4	4	4	1	4	4	4	4	4	4	4	4
4	2	4	4	4	1	4	4	4	4	4	4	4	4
5	1	3	3	3	1	4	4	5	5	5	5	2	5
5	2	4	4	4	1	4	4	5	3	3	3	4	4
3	3	2	3	3	1	3	3	3	3	3	4	4	4
5	1	5	5	5	1	5	5	5	5	5	5	5	5
5	3	5	5	5	3	5	5	5	5	5	5	5	5
4	3	3	4	4	3	4	4	4	4	4	4	4	4
5	1	5	5	5	1	5	5	5	5	5	5	5	5
4	2	4	4	5	1	4	4	5	5	5	5	5	5
4	2	4	4	4	2	4	4	4	4	4	4	4	4
5	5	5	5	5	1	5	5	4	3	3	5	5	5
4	2	3	3	3	2	4	3	4	3	3	4	3	4
4	2	4	5	4	2	4	5	4	4	4	4	3	4
5	2	5	5	5	2	4	5	4	5	4	5	2	4
5	2	5	5	5	2	5	5	5	5	5	5	5	5
5	2	4	5	5	1	4	4	4	5	5	4	4	5
4	2	4	4	4	2	4	4	4	4	4	4	4	4
5	1	4	5	5	1	5	5	5	4	4	4	4	4
5	5	5	5	5	5	5	5	5	5	5	5	5	5
4	2	4	4	4	1	4	4	4	4	4	4	4	4
3	2	3	4	4	1	4	3	3	4	3	4	5	4
4	2	3	4	4	1	4	4	4	4	4	4	4	4
3	3	3	3	3	3	3	3	3	3	3	3	3	3
4	4	4	4	4	2	4	4	3	4	4	4	4	4
5	1	5	5	5	1	5	5	5	5	5	5	5	5
4	3	3	3	3	1	5	4	4	3	4	4	3	4

3	3	3	3	3	3	3	3	3	3	3	3	3	3
3	2	3	3	3	1	3	4	3	4	4	4	3	3
4	2	4	4	4	2	4	4	3	4	4	5	5	5
5	3	2	3	3	1	3	3	3	3	3	3	3	3
4	3	3	4	4	4	4	4	4	4	4	4	4	4
5	2	3	3	4	1	5	3	3	5	5	5	4	5
5	1	4	5	5	1	5	5	5	5	5	5	4	5
5	3	5	5	5	1	5	5	3	5	5	5	5	5
5	1	5	5	5	1	5	5	5	5	5	5	5	5
5	1	5	5	5	1	5	5	5	5	5	4	5	5
4	2	4	4	4	1	5	5	5	5	5	5	5	5
5	1	5	5	5	1	5	5	5	5	5	5	5	5
4	4	4	4	4	4	4	4	4	4	4	4	4	4
5	3	3	5	3	1	5	5	5	5	5	5	5	5
5	1	5	5	5	1	5	5	5	5	5	5	5	5
4	2	4	4	4	1	5	5	4	4	4	4	4	5
4	1	4	5	5	1	5	4	4	4	4	4	4	4
4	3	3	4	4	1	4	3	3	3	3	4	5	4
4	4	4	4	4	1	5	5	5	5	5	5	4	4
4	2	3	4	4	2	4	4	4	4	4	4	4	4
5	1	5	2	5	1	5	5	5	5	5	5	5	5
4	2	3	4	4	1	4	4	4	4	4	4	5	5
5	1	4	5	5	1	5	5	5	5	5	5	5	5
5	1	5	5	5	1	5	5	5	5	5	5	4	5
4	4	4	4	4	4	4	4	4	4	4	4	4	4
3	2	3	3	3	2	3	3	2	3	3	3	3	3
5	5	5	5	5	5	5	5	5	5	5	5	5	5
5	2	4	4	4	1	5	5	5	5	5	5	5	5
5	3	4	5	5	2	5	5	5	5	5	5	5	5
4	2	4	4	4	2	4	4	4	2	4	4	4	4
4	4	4	4	4	2	4	4	3	4	4	4	4	4
4	3	3	4	3	2	4	4	3	4	4	4	4	5
5	1	4	5	5	1	5	5	4	5	5	4	5	5
4	1	3	4	4	1	4	4	3	4	4	4	4	4
5	1	5	5	5	1	5	5	5	5	5	5	5	5
4	2	4	4	4	1	4	4	4	4	4	4	4	4
5	5	5	5	5	1	5	5	5	5	5	5	5	5
4	2	4	4	4	1	4	3	4	4	4	4	3	5
5	1	5	5	5	1	5	5	5	5	5	5	5	5
4	1	3	4	3	1	4	3	4	4	4	3	4	4
5	1	3	5	5	1	5	5	5	5	5	5	5	5
5	1	5	5	5	1	5	5	5	5	5	5	5	5
5	1	5	5	5	1	5	5	5	5	5	5	5	5
4	4	4	4	4	4	4	4	4	4	4	4	4	4
5	1	5	5	5	1	5	5	4	5	5	4	4	4
5	2	3	4	4	1	4	4	5	4	5	3	5	4
4	2	3	4	4	1	4	4	4	4	4	4	4	4
3	2	2	3	3	2	3	3	3	3	3	3	3	3
5	2	5	5	5	1	5	5	5	5	5	5	5	5
4	4	4	4	4	4	4	4	4	4	4	4	4	4
3	1	3	3	3	1	4	3	2	2	2	2	3	3
4	3	4	4	4	3	4	4	4	4	4	4	4	4
5	1	5	5	5	1	5	5	5	5	5	3	5	5
4	5	3	4	4	1	4	3	3	3	3	3	4	4
5	1	5	5	5	1	5	5	5	5	5	5	5	5
4	1	5	5	5	1	5	4	4	4	5	4	5	5

5	1	5	5	5	1	5	5	5	5	5	5	5	5
4	2	4	4	4	2	4	3	4	4	4	4	4	4
3	3	3	3	3	3	3	3	3	3	3	3	3	3
5	2	5	5	5	1	5	5	5	5	5	5	5	5
4	1	4	5	4	1	5	4	4	4	4	4	4	3
5	2	5	5	5	2	5	5	5	5	5	5	5	5
5	1	5	4	5	1	5	5	4	4	4	3	5	5
5	5	5	5	5	1	5	3	3	4	5	5	5	5
5	1	5	5	5	1	5	5	5	5	5	5	5	5
5	1	5	5	5	1	4	4	4	5	5	5	5	5
4	1	4	4	5	1	5	5	5	5	5	4	4	4
5	1	5	5	5	1	5	5	5	5	5	5	5	5
4	1	4	4	4	2	4	4	3	4	4	3	4	4
5	1	5	5	5	1	5	5	5	5	5	5	5	5
5	5	5	5	5	5	5	5	5	5	5	5	5	5
5	1	3	4	4	1	5	5	5	5	5	5	5	5
4	1	4	4	4	1	4	4	4	3	4	4	4	4
4	2	4	4	4	2	4	4	4	4	4	4	4	4
5	2	4	4	4	1	4	5	4	4	4	5	4	5
4	2	4	4	4	2	4	4	4	4	4	4	4	4
5	5	5	5	5	5	5	5	5	5	5	5	5	5
5	1	5	5	5	1	5	5	5	5	5	5	5	5
5	5	5	5	5	5	5	5	5	5	5	5	5	5
5	1	4	5	4	1	5	5	5	5	5	5	5	5
3	5	4	5	5	3	5	5	5	5	5	5	5	5
5	1	5	5	5	1	5	5	5	5	5	5	5	5
5	1	5	5	5	1	5	5	5	5	5	5	5	5
4	2	4	4	4	1	4	4	3	3	4	3	4	4
5	1	5	5	5	1	5	5	5	5	5	5	5	5
5	1	4	5	4	1	5	4	3	4	4	4	4	4
5	1	5	5	5	1	5	5	5	5	5	5	5	5
3	3	3	3	3	1	5	3	2	2	2	3	4	4
4	1	4	4	4	2	4	4	4	4	4	4	4	4
3	3	3	4	3	1	5	4	3	3	3	4	3	4
4	3	4	4	4	1	3	3	3	4	4	3	4	4
4	1	3	4	4	1	4	5	3	2	3	4	4	5
3	3	2	3	3	2	4	3	3	3	3	3	3	3
5	1	5	5	5	1	5	5	5	5	5	5	5	5
4	2	4	4	4	1	4	4	4	4	4	4	4	4
4	4	4	4	4	4	4	4	4	4	4	4	4	4
5	1	5	5	5	1	5	5	5	5	5	5	5	5
4	1	4	4	4	1	4	4	4	4	4	4	4	4
5	2	5	5	4	2	4	4	4	4	4	4	4	4
4	1	4	4	4	1	4	4	4	4	4	4	4	4
4	2	2	4	1	2	4	2	2	2	4	2	4	4
4	2	3	4	4	2	4	4	4	4	4	4	4	4
5	1	5	5	5	1	5	5	5	5	5	5	5	5
5	3	3	3	3	2	3	3	3	3	3	4	4	5
5	1	5	5	5	1	5	5	5	5	5	5	5	5
5	5	5	5	5	5	5	5	5	5	5	5	5	5
4	2	2	4	4	1	4	4	4	4	4	4	4	4
4	2	3	4	4	1	4	3	4	4	4	4	4	5
3	3	3	3	3	3	3	3	3	3	3	3	3	3
4	4	4	4	4	1	4	4	4	4	4	4	4	4
4	3	3	4	3	2	4	3	3	3	3	2	3	4
3	2	3	3	3	2	4	3	2	3	3	3	3	3

5	5	5	5	5	5	5	5	5	5	5	5	5	5
5	1	5	5	5	1	5	5	5	5	5	5	5	5
5	1	4	5	5	1	5	5	5	5	5	5	5	5
5	5	5	5	5	1	5	5	5	5	5	5	5	5
4	2	4	4	4	2	4	4	4	4	4	4	4	4
4	4	4	4	4	1	5	5	4	4	4	4	4	4
4	3	3	4	4	2	4	4	4	2	4	4	4	4
4	2	3	4	4	2	3	4	3	3	3	3	3	4
3	3	3	3	3	3	3	3	3	3	3	3	3	3
4	2	4	4	4	2	5	5	4	4	4	4	4	5
5	1	5	5	5	1	5	5	4	5	5	5	4	5
4	4	4	4	4	4	4	4	4	4	4	4	4	4
4	1	4	5	4	1	5	4	3	3	4	3	4	5
4	2	3	3	4	2	4	3	3	4	4	4	4	4
5	1	5	5	5	1	5	5	5	5	5	4	5	5
2	4	4	2	4	3	3	4	3	2	3	4	3	3
4	2	3	4	4	1	4	4	4	3	4	4	4	5
5	2	5	5	5	5	5	5	5	5	5	5	5	5
4	1	4	4	4	1	5	4	4	4	4	5	4	5
3	3	3	3	3	3	3	3	3	3	3	3	3	3
4	2	4	4	4	2	4	4	4	4	4	4	4	4
5	3	3	5	3	1	5	5	3	3	5	3	5	3
5	1	5	5	5	1	5	5	5	5	5	5	5	5
5	1	5	5	5	1	5	5	5	5	5	5	5	5
4	2	4	4	4	2	4	4	4	4	4	4	4	4
3	2	4	4	3	2	4	3	3	3	3	3	3	4
4	3	3	3	4	1	4	3	3	2	4	4	3	4
4	1	4	5	4	1	5	4	4	4	4	5	4	5
5	1	5	5	5	1	5	5	5	5	5	5	5	5
4	2	3	4	3	2	4	4	4	4	4	4	4	4
5	1	4	4	4	1	5	5	4	4	4	4	4	5
5	1	5	5	5	1	5	5	4	5	5	5	5	5
4	2	4	4	4	2	4	5	3	3	3	2	3	2
4	1	4	4	4	1	4	4	4	4	4	4	4	4
5	1	5	5	5	1	5	5	5	5	5	5	5	5
4	1	4	4	4	1	4	4	4	4	4	4	4	5
4	2	3	4	3	1	5	3	3	3	3	3	4	4
4	1	4	4	3	1	4	3	4	4	4	4	3	3
5	1	5	5	5	1	5	5	5	5	5	5	5	5
5	2	4	5	5	1	5	5	4	5	4	5	5	5
5	2	4	5	4	2	5	5	4	4	4	4	5	5
3	1	3	4	3	1	5	4	3	3	3	4	4	3
5	2	4	5	5	1	5	5	5	5	5	5	5	5
4	2	4	4	4	2	4	4	3	4	4	4	5	5
5	1	5	5	5	1	5	5	5	5	5	5	5	5
3	2	2	2	3	4	4	4	4	4	4	4	4	4
4	2	3	4	4	1	4	4	4	4	4	4	4	4
4	2	4	4	4	2	4	4	4	4	4	4	4	3
4	4	4	4	4	4	4	4	4	5	5	5	5	4
5	2	4	5	5	1	5	5	5	5	5	5	5	5
5	4	5	5	5	2	5	5	1	2	5	5	5	5
5	5	5	5	5	1	5	5	5	5	5	5	5	5
4	2	2	5	4	1	5	5	4	4	3	3	5	4
5	5	5	5	5	1	5	5	5	5	5	5	5	5
4	2	4	4	4	2	4	4	4	4	4	4	4	4
5	1	2	5	5	2	5	5	5	5	5	5	5	5

5	5	5	5	5	5	5	5	5	5	5	5	5	5
5	1	5	5	5	1	5	5	5	5	5	5	5	5
5	1	5	5	5	1	5	5	5	5	5	5	5	5
5	1	5	5	5	1	5	5	5	5	5	5	5	5
4	2	3	4	4	1	4	4	4	4	4	4	4	4
4	2	4	4	4	2	5	4	4	4	4	4	4	4
4	3	4	4	4	2	4	3	3	4	4	4	4	3
4	2	4	4	4	2	5	4	4	4	4	4	4	4
3	2	3	3	3	2	3	3	3	3	3	3	3	3
5	1	5	5	5	1	5	5	4	5	5	5	4	5
5	1	5	5	5	1	5	5	5	5	5	5	5	5
3	2	4	4	3	2	4	4	3	4	3	3	4	3
5	1	5	5	5	1	5	5	5	5	5	5	5	5
5	1	5	5	5	1	5	5	5	5	5	5	5	5
4	3	4	4	4	1	4	4	3	3	4	4	4	4
4	1	4	4	4	1	4	4	4	4	4	3	4	4
5	2	3	4	4	2	4	4	4	4	4	4	4	4
4	1	4	4	4	2	5	4	4	4	4	4	5	4
5	1	5	5	5	1	5	1	5	4	4	4	5	5
5	1	4	5	5	1	5	5	5	5	5	5	5	5
5	1	5	5	5	1	5	5	5	5	5	5	5	5
4	3	2	4	3	1	4	3	3	2	3	2	4	3
3	3	3	3	3	2	4	4	4	4	4	3	4	4
4	1	4	4	4	1	4	4	4	4	4	4	4	4
4	2	4	4	4	2	4	4	4	3	4	4	4	4
3	3	3	3	3	3	3	3	3	3	3	3	3	3
5	1	5	5	5	1	5	5	5	5	5	5	5	5
5	1	5	5	5	1	5	5	5	5	5	5	5	5
5	1	5	5	5	2	4	5	5	5	5	5	5	5
2	4	2	4	2	4	2	2	2	2	2	4	4	4
5	1	5	5	5	1	5	5	5	5	5	5	5	5
5	1	5	5	5	1	5	5	5	5	5	5	5	5
5	1	5	5	5	1	5	5	5	5	5	5	4	5
5	1	5	5	5	1	5	5	5	5	5	5	4	4
5	2	5	5	5	1	5	5	5	4	4	5	4	5
4	1	4	5	4	1	4	4	4	4	4	4	4	4
3	3	3	3	3	2	3	3	3	3	3	3	3	3
5	2	3	5	5	1	5	5	5	4	4	4	3	4
4	3	3	3	3	3	3	4	3	3	3	3	3	3
5	3	4	4	3	4	4	5	4	5	4	4	3	4
4	1	4	5	4	1	5	4	4	4	4	4	4	4
5	2	5	5	5	1	5	5	5	5	5	5	5	5
5	1	5	5	5	1	5	5	5	5	5	5	5	5
4	1	4	4	4	1	5	3	3	3	4	3	5	4
5	1	5	5	5	1	5	5	5	5	5	5	5	5
3	3	2	4	3	2	4	3	4	2	3	3	3	3
4	1	4	4	4	1	5	4	4	4	4	4	5	4
5	5	5	5	5	5	5	5	5	5	5	5	5	5
5	5	5	5	5	5	5	5	5	5	5	5	5	5
4	3	4	4	4	4	4	4	4	4	4	1	4	2
3	2	2	3	3	1	5	4	2	3	3	4	5	5
5	1	5	5	5	1	5	5	5	5	5	5	5	5
4	2	4	4	4	2	4	4	4	4	4	4	4	4
4	2	4	4	4	2	4	4	4	4	4	4	4	4
3	3	3	3	3	2	4	3	4	4	4	4	4	4
4	2	4	4	4	1	4	4	4	4	4	4	4	4

5	1	5	5	5	1	5	5	5	5	5	5	5	5
4	1	4	4	4	1	5	4	4	4	4	4	5	4
4	1	4	4	4	1	4	4	4	4	4	4	4	4
5	5	5	5	5	5	5	5	5	5	5	5	5	5
3	2	4	4	4	1	4	4	3	3	4	2	4	4
5	1	5	5	5	1	5	5	5	5	5	5	5	5
4	3	4	3	3	3	3	3	3	3	3	3	3	3
4	2	4	4	4	2	4	4	3	4	4	4	4	4
5	1	5	5	5	1	5	5	1	5	5	1	5	5
5	1	5	5	5	1	5	5	5	5	5	5	5	5
5	1	5	5	5	1	5	5	5	5	5	5	5	5
5	1	5	5	5	1	5	5	4	4	5	5	4	5
4	4	4	4	4	1	4	4	4	4	5	1	4	4
5	1	5	5	5	1	5	5	5	5	5	5	5	5
5	2	5	5	5	1	5	5	5	5	5	5	5	4
4	4	4	3	4	1	3	4	4	2	4	4	4	4
4	1	3	3	4	1	5	4	3	4	3	3	4	3
4	2	4	4	4	2	4	4	4	4	4	4	3	3
4	1	4	4	4	1	4	4	4	4	4	4	5	4
4	1	4	4	4	1	4	4	4	4	4	4	4	4
4	2	2	4	4	1	4	4	4	4	4	2	4	4
4	1	5	5	5	1	5	5	4	4	4	5	5	3
5	1	5	5	5	1	5	5	5	5	5	4	4	5
4	2	4	4	3	1	3	3	3	3	4	4	3	3
4	3	4	4	4	1	4	4	4	4	4	4	4	4
5	1	5	5	5	1	5	5	5	5	5	5	5	5
5	5	3	5	3	5	5	5	3	3	5	4	4	5
5	2	4	5	5	1	5	4	5	5	5	5	4	4
5	2	3	5	5	1	5	5	5	5	4	5	4	5
5	1	5	5	5	1	5	5	5	5	5	5	5	5
5	1	5	5	5	5	5	4	4	4	4	4	4	4
4	1	4	5	4	1	5	4	4	4	4	4	4	4
5	1	5	5	5	1	5	5	5	5	5	5	5	5
5	1	4	5	4	1	5	5	4	5	5	5	5	5
4	2	4	4	4	3	4	4	4	4	4	4	4	4
5	2	5	5	5	1	5	5	5	5	5	5	5	5
5	1	5	5	5	5	5	5	5	5	5	5	5	5
4	2	4	4	4	2	4	4	4	4	4	4	4	4
5	1	5	5	5	1	5	5	5	5	5	5	5	5
5	1	5	5	5	1	5	5	5	5	5	5	5	5
4	2	4	4	4	1	4	3	4	4	4	4	4	4
5	3	5	5	5	3	4	5	4	5	5	5	5	5
4	1	4	4	4	1	4	4	3	3	3	4	4	3
5	3	3	5	5	1	5	5	4	5	5	5	5	5
5	1	5	5	5	1	5	5	5	5	5	5	5	5
4	1	4	4	4	1	4	4	4	4	4	4	4	4
4	2	4	4	4	2	4	4	4	4	4	3	4	4
4	2	4	4	4	3	3	4	4	3	3	4	4	4
4	2	4	3	3	1	4	4	4	4	4	4	4	4

e48	e49	e50	totalburnout	db1	db2	db3	b1	b2	b3
1	5	1	44	4	5	35	5	1	1
1	4	1	38	6	7	25	4	1	3
1	5	1	46	7	5	34	5	1	1
3	3	1	42	17	6	19	3	3	1
2	5	1	39	7	9	23	3	3	2
4	3	1	48	11	10	27	5	3	3
1	5	1	40	11	5	24	3	2	1
2	4	2	36	7	8	21	4	2	2
3	3	3	50	14	15	21	3	5	3
1	5	1	63	17	15	31	5	5	3
1	4	1	36	5	5	26	5	1	1
2	4	1	49	12	15	22	3	3	3
1	5	1	45	8	10	27	5	1	2
4	3	2	46	12	13	21	4	3	2
1	5	1	41	6	5	30	5	1	1
2	5	1	40	9	6	25	4	2	1
2	4	1	43	11	8	24	4	3	2
2	4	1	50	12	7	31	4	4	3
1	4	1	44	8	7	29	5	3	3
1	3	1	46	16	10	20	3	3	3
1	5	1	44	10	6	28	4	1	1
5	5	1	44	4	5	35	5	1	1
3	3	3	48	12	15	21	3	3	3
2	5	1	40	4	5	31	5	1	1
2	3	1	45	12	11	22	4	2	2
1	4	1	48	9	8	31	5	2	2
4	3	1	43	9	6	28	5	4	1
3	4	1	45	13	11	21	4	4	3
1	4	1	42	10	5	27	4	2	1
2	3	1	43	12	5	26	5	3	1
2	4	1	46	11	7	28	4	2	1
1	5	1	47	7	5	35	5	1	1
3	4	2	53	16	17	20	3	4	4
3	2	2	39	10	10	19	3	2	3
1	5	1	44	8	5	31	4	1	1
2	3	1	39	7	6	26	5	2	2
2	4	2	44	9	8	27	4	2	2
3	3	3	48	12	15	21	3	3	3
3	5	1	45	9	10	26	4	3	2
3	3	3	49	14	15	20	3	5	3
2	3	1	49	14	14	21	3	3	3
3	3	3	48	12	15	21	3	3	3
3	4	1	43	13	11	19	4	2	3
1	3	1	42	4	5	33	5	1	1
1	4	1	43	10	8	25	4	2	2
2	5	1	41	7	5	29	4	1	1
1	5	1	38	7	6	25	5	1	1
5	5	5	80	20	25	35	5	5	5
2	4	1	45	9	10	26	4	2	2
1	5	1	43	9	5	29	4	2	1

1	5	1	45	7	5	33	5	1	1
4	4	3	47	11	15	21	4	2	3
1	4	1	34	6	5	23	4	2	1
1	3	1	45	10	6	29	4	1	1
1	4	1	42	8	5	29	5	3	1
1	4	2	46	8	10	28	4	2	2
2	4	1	42	11	8	23	4	4	2
2	2	2	51	15	11	25	4	4	2
1	4	1	37	8	9	20	4	2	1
2	3	1	45	9	9	27	3	3	2
2	3	1	46	11	13	22	3	2	2
2	4	1	44	7	5	32	4	2	1
1	3	1	38	7	5	26	4	3	1
5	5	1	54	18	11	25	3	5	3
2	3	1	45	12	9	24	4	2	2
2	4	2	49	11	10	28	4	4	2
2	4	1	45	10	10	25	5	3	2
2	3	1	36	6	5	25	4	1	1
2	4	1	38	10	8	20	4	3	2
3	4	1	39	10	6	23	4	4	1
3	4	2	48	10	12	26	4	3	2
2	3	1	49	15	5	29	4	5	1
5	5	1	42	4	5	33	5	1	1
1	5	1	39	5	5	29	5	1	1
3	3	1	32	5	5	22	3	1	1
2	2	1	46	12	14	20	3	3	3
2	4	1	35	4	5	26	5	1	1
2	3	1	47	9	12	26	4	2	2
3	4	1	40	7	5	28	5	2	1
4	3	1	41	10	7	24	4	3	2
2	4	2	50	12	11	27	4	3	2
4	4	1	44	10	7	27	5	3	1
3	4	1	47	12	13	22	4	4	3
2	5	1	44	13	9	22	4	3	2
1	3	1	38	4	5	29	5	1	1
3	3	3	48	12	15	21	3	3	3
1	5	1	46	6	5	35	5	3	1
2	3	1	38	8	5	25	4	2	1
2	3	2	48	13	13	22	4	4	2
3	5	1	44	4	5	35	5	1	1
3	3	1	44	14	10	20	3	4	2
2	5	1	37	8	6	23	5	3	1
2	5	1	47	10	10	27	4	3	2
5	5	1	48	9	6	33	5	1	1
5	5	1	44	4	5	35	5	1	1
1	4	1	46	7	5	34	5	1	1
1	4	2	38	9	8	21	3	2	1
2	4	2	44	12	11	21	4	3	2
1	5	1	49	13	9	27	4	3	2
2	3	2	40	8	11	21	3	2	2
1	3	1	31	4	5	22	4	1	1
1	5	1	47	8	9	30	5	2	2
3	4	1	47	9	10	28	4	2	2
1	5	1	47	8	6	33	5	1	1
2	4	1	41	8	5	28	4	2	1
1	4	1	44	5	5	34	5	1	1

1	5	1	45	5	5	35	5	1	1
2	3	2	45	13	12	20	3	3	2
2	4	1	44	13	7	24	5	3	1
5	5	1	41	4	5	32	5	1	1
2	4	1	41	9	7	25	4	2	2
1	5	1	39	4	5	30	5	1	1
1	4	1	42	6	5	31	5	1	1
1	4	1	43	8	6	29	5	2	1
4	4	4	48	11	14	23	4	2	4
2	4	1	44	8	8	28	4	2	1
1	5	1	46	7	5	34	5	2	1
2	4	1	39	8	8	23	3	2	2
3	3	1	47	10	10	27	4	3	2
1	4	1	47	10	6	31	5	5	1
3	3	1	45	12	12	21	3	3	3
3	3	1	41	10	10	21	3	3	3
5	5	5	80	20	25	35	5	5	5
1	2	2	43	13	7	23	4	4	1
5	5	5	44	4	5	35	5	1	1
1	5	1	44	10	7	27	5	3	1
2	4	1	44	10	7	27	4	3	2
3	2	3	50	13	16	21	3	4	3
3	3	1	34	6	5	23	3	1	1
2	3	2	46	10	10	26	4	3	2
2	4	1	44	10	12	22	4	3	3
2	4	1	41	7	7	27	4	3	1
1	5	1	46	8	5	33	5	1	1
1	3	1	42	9	7	26	4	3	1
1	5	1	59	20	14	25	5	5	1
3	5	1	44	16	6	22	3	5	2
3	3	2	42	10	11	21	3	2	2
2	2	1	44	12	11	21	3	2	2
3	3	3	44	10	10	24	4	3	2
2	3	1	66	18	23	25	2	5	5
2	4	1	46	8	10	28	5	2	2
2	5	1	45	11	7	27	4	2	1
2	3	1	45	11	10	24	3	4	2
2	5	1	52	13	9	30	5	4	2
5	5	5	54	15	9	30	5	5	5
1	5	1	62	20	23	19	1	5	5
2	3	2	47	13	13	21	3	3	3
2	4	1	38	11	6	21	4	1	1
2	3	1	47	12	14	21	3	3	2
2	4	1	44	9	9	26	4	3	2
3	3	3	46	11	13	22	3	3	3
1	4	1	37	5	5	27	4	1	1
2	5	2	47	8	8	31	5	2	1
1	3	1	39	9	9	21	3	2	2
1	5	1	44	4	5	35	5	1	1
5	5	5	80	20	25	35	5	5	5
5	5	1	46	7	5	34	5	1	1
1	5	1	39	7	5	27	5	1	1
2	4	2	44	11	11	22	4	2	2
2	5	2	49	11	9	29	5	2	2
5	5	4	59	13	19	27	4	3	3
4	4	4	64	16	20	28	4	4	4

2	5	1	46	7	10	29	5	2	2
2	5	1	47	8	7	32	5	2	1
1	5	1	44	10	12	22	4	3	3
2	4	2	42	9	9	24	4	3	2
5	5	1	46	8	9	29	5	1	1
1	5	1	36	12	5	19	2	3	1
1	4	1	39	5	5	29	5	2	1
4	4	4	64	16	20	28	4	4	4
2	4	1	47	15	10	22	3	4	2
1	5	1	44	8	5	31	5	5	1
1	5	1	41	4	5	32	5	1	1
2	3	1	40	10	7	23	4	2	2
3	4	2	38	18	11	9	2	4	1
1	4	1	44	12	5	27	5	2	1
1	5	1	45	10	7	28	5	3	2
1	5	1	44	8	7	29	5	2	1
1	5	1	42	4	5	33	5	1	1
3	3	3	48	12	15	21	3	3	3
2	4	1	48	8	9	31	5	1	1
2	5	2	49	14	10	25	4	1	3
5	4	2	56	16	14	26	4	4	3
2	5	1	47	12	12	23	3	5	3
1	4	1	47	9	10	28	4	3	3
3	3	3	48	12	15	21	3	3	3
4	4	2	64	16	20	28	4	4	4
4	4	4	57	16	16	25	4	4	4
1	5	1	43	4	5	34	5	1	1
4	3	2	57	16	12	29	4	3	3
1	2	1	47	8	5	34	5	5	1
1	5	1	53	17	15	21	4	4	2
2	4	1	42	10	6	26	4	3	1
1	5	1	48	8	5	35	5	1	1
1	5	1	42	6	5	31	5	1	1
1	4	1	43	4	5	34	5	1	1
1	2	1	41	7	5	29	5	1	1
3	4	2	49	12	11	26	5	3	2
2	3	3	46	12	7	27	3	3	1
3	3	1	38	6	5	27	5	1	1
2	2	1	44	13	10	21	4	4	2
2	3	1	46	9	10	27	4	3	2
3	3	3	48	12	15	21	3	3	3
1	5	1	44	4	5	35	5	1	1
4	4	2	56	17	14	25	4	4	5
1	4	1	41	8	7	26	4	1	1
2	3	2	44	12	12	20	3	4	2
4	4	1	43	10	6	27	3	4	2
2	4	1	45	11	10	24	4	3	2
2	5	2	41	11	9	21	3	4	2
2	3	2	39	8	6	25	4	3	2
1	4	1	46	8	10	28	4	2	2
1	5	1	52	10	12	30	5	5	5
1	5	1	43	4	5	34	5	1	1
2	4	1	47	8	10	29	5	2	2
3	3	1	49	14	11	24	3	3	3
4	4	1	42	8	8	26	4	1	1
1	5	1	36	4	5	27	4	1	1

2	4	2	42	8	9	25	5	1	2
2	3	1	50	14	14	22	3	4	3
1	5	1	44	4	5	35	5	1	1
2	4	1	45	14	14	17	3	5	4
1	5	1	46	8	5	33	5	1	1
1	5	1	44	4	5	35	5	1	1
1	4	1	41	11	5	25	3	4	1
1	3	1	49	10	6	33	5	3	1
1	4	1	43	8	6	29	5	2	1
4	4	4	44	8	10	26	4	2	2
2	5	1	39	6	6	27	4	2	2
2	5	1	39	6	5	28	4	2	1
2	4	1	37	7	5	25	4	1	1
4	4	2	39	6	6	27	4	2	2
2	4	1	34	6	6	22	5	2	1
1	3	1	37	5	5	27	5	1	1
1	5	1	44	8	5	31	5	1	1
1	3	1	39	12	5	22	3	3	1
3	3	1	47	11	10	26	4	3	3
1	4	1	41	7	6	28	5	2	2
1	4	2	43	6	5	32	5	2	1
1	5	1	41	11	5	25	4	3	1
1	5	1	45	7	6	32	5	2	1
3	4	1	44	7	9	28	5	1	1
2	5	2	46	11	6	29	5	5	2
1	3	1	36	4	5	27	4	1	1
1	3	1	42	5	5	32	5	1	1
3	3	2	44	11	10	23	4	4	2
4	4	2	46	9	9	28	5	3	1
3	4	1	42	9	6	27	4	2	1
2	4	3	41	7	5	29	4	2	1
1	5	1	44	10	10	24	5	2	2
1	5	1	41	6	5	30	5	3	1
3	3	1	45	9	10	26	4	3	3
1	4	1	41	8	5	28	5	4	1
4	4	4	41	7	5	29	5	4	1
2	5	1	35	8	6	21	4	1	2
1	5	1	43	4	5	34	5	1	1
1	5	1	41	8	8	25	4	1	1
1	4	1	40	7	5	28	4	2	1
1	5	1	45	10	8	27	5	4	2
1	5	1	39	8	5	26	5	3	1
1	5	1	44	4	5	35	5	1	1
2	4	2	45	11	10	24	4	2	2
3	2	1	48	15	9	24	4	3	3
5	5	5	39	7	7	25	5	2	1
1	5	1	45	10	8	27	4	3	2
2	4	2	46	14	11	21	3	4	3
3	3	1	42	13	11	18	3	4	2
3	3	2	39	10	9	20	2	2	2
1	5	1	50	15	8	27	4	5	2
2	4	3	49	11	10	28	4	3	2
3	4	1	32	4	5	23	5	1	1
2	4	1	43	8	5	30	5	2	1
2	4	2	46	9	10	27	4	2	2
3	2	1	36	9	6	21	3	3	1

1	5	1	54	9	10	35	5	1	1
2	3	1	41	9	5	27	5	2	1
1	5	1	48	9	5	34	5	1	1
1	5	1	43	7	5	31	5	1	1
1	5	1	44	4	5	35	5	1	1
2	4	2	46	8	10	28	4	2	2
2	1	1	58	16	18	24	2	4	4
2	3	1	49	17	13	19	4	4	3
2	4	2	42	14	12	16	2	4	4
1	5	1	44	4	5	35	5	1	1
4	3	2	43	11	8	24	3	2	1
1	4	1	37	4	5	28	4	1	1
3	5	1	42	17	11	14	1	3	3
2	5	1	34	6	5	23	3	3	1
5	4	4	68	18	22	28	4	4	4
5	5	5	80	20	25	35	5	5	5
1	4	1	53	15	7	31	5	5	1
3	3	3	48	12	15	21	3	3	3
3	4	2	48	12	15	21	3	3	3
1	5	1	36	5	6	25	5	1	2
2	3	2	44	11	6	27	4	3	1
4	4	5	48	9	14	25	4	2	1
2	5	1	39	7	7	25	4	2	1
2	3	1	45	9	5	31	5	1	1
2	4	1	41	7	7	27	4	2	2
1	5	1	44	4	5	35	5	1	1
2	3	2	48	10	12	26	4	2	2
2	4	1	51	11	14	26	4	3	3
4	4	4	45	12	5	28	4	4	1
1	3	1	44	10	5	29	5	5	1
1	4	1	39	5	6	28	4	1	1
2	4	1	47	10	10	27	4	3	2
1	2	1	42	4	5	33	5	1	1
3	3	3	46	12	14	20	2	3	3
1	5	1	39	4	5	30	5	1	1
1	5	1	37	4	7	26	5	1	1
1	5	1	44	4	5	35	5	1	1
2	4	1	37	7	5	25	4	1	1
2	3	1	41	5	5	31	5	1	1
2	3	1	41	5	5	31	5	1	1
1	4	1	33	5	5	23	3	1	1
1	5	1	44	4	5	35	5	1	1
1	4	1	38	7	8	23	4	1	2
2	3	1	45	8	9	28	4	1	2
5	4	1	49	13	9	27	4	3	1
1	5	1	41	6	5	30	5	1	1
5	5	5	68	16	17	35	5	5	5
2	5	1	44	5	5	34	5	1	1
1	5	1	51	12	13	26	5	2	4
1	2	1	47	11	6	30	5	4	1
2	4	1	45	13	8	24	4	3	2
5	1	1	44	9	11	24	3	1	1
2	2	2	42	11	8	23	3	2	2
2	4	1	40	6	7	27	4	2	2
3	3	3	48	12	15	21	3	3	3
1	5	1	41	6	5	30	5	1	1

5	5	1	49	9	5	35	5	5	1
1	4	1	38	6	5	27	4	2	1
1	5	1	45	10	6	29	5	4	1
1	4	1	37	4	5	28	4	1	1
1	5	1	40	4	8	28	5	1	1
2	4	2	41	8	10	23	4	2	3
2	5	1	48	8	10	30	5	2	2
2	3	1	50	14	15	21	3	5	3
2	4	1	42	10	7	25	4	3	2
3	3	3	48	12	15	21	3	3	3
1	3	1	44	11	9	24	4	3	2
5	5	1	48	8	5	35	5	3	1
5	5	5	80	20	25	35	5	5	5
4	4	4	64	16	20	28	4	4	4
1	5	1	43	4	5	34	5	1	1
1	4	1	44	8	7	29	5	3	2
1	5	1	43	4	5	34	5	1	1
1	4	1	41	4	5	32	5	1	1
2	4	1	39	10	6	23	4	2	1
3	4	1	46	6	5	35	5	1	1
2	4	2	50	13	7	30	5	4	1
2	3	1	47	10	10	27	4	2	2
2	4	2	45	9	9	27	4	2	2
3	4	1	44	11	8	25	4	1	1
5	5	1	44	4	5	35	5	1	1
1	3	1	47	13	5	29	4	4	1
2	5	1	38	6	5	27	4	2	1
1	5	1	49	9	10	30	5	1	2
1	5	1	38	4	5	29	5	1	1
2	4	2	42	8	10	24	4	2	2
2	3	1	48	12	9	27	4	2	1
3	3	2	40	13	12	15	2	4	4
3	5	1	42	8	5	29	5	2	1
4	4	1	37	4	5	28	4	1	1
3	3	1	39	9	9	21	3	2	2
1	4	1	50	12	10	28	1	4	1
2	5	1	49	11	11	27	5	2	1
4	4	2	47	10	10	27	4	3	2
2	3	2	42	10	10	22	4	3	2
1	5	1	43	10	7	26	5	1	1
3	3	2	50	13	10	27	4	3	2
2	4	1	45	10	6	29	5	3	1
2	5	1	47	8	6	33	5	1	1
2	4	2	43	10	8	25	4	3	2
1	5	1	48	8	5	35	5	5	1
1	3	1	40	4	5	31	5	1	1
1	2	1	36	4	5	27	4	1	1
2	2	1	34	11	7	16	3	4	2
1	5	1	43	6	5	32	5	1	1
2	4	2	48	10	11	27	4	3	2
1	4	1	37	5	5	27	5	1	1
2	5	1	38	6	6	26	4	2	1
5	5	5	80	20	25	35	5	5	5
2	4	2	50	15	13	22	4	4	4
1	5	1	43	4	5	34	5	1	1
3	3	3	48	12	15	21	3	3	3

2	2	1	41	12	8	21	3	3	2
2	5	1	42	5	5	32	5	1	1
1	5	1	44	4	5	35	5	1	1
1	3	1	45	11	5	29	5	3	1
4	3	2	48	11	13	24	4	3	2
1	2	1	38	7	5	26	5	3	1
3	5	1	49	11	15	23	4	3	3
1	5	1	40	5	7	28	4	1	1
1	5	1	42	4	5	33	5	1	1
1	4	1	38	5	5	28	4	2	1
2	4	1	43	7	6	30	4	2	1
1	4	1	39	6	6	27	4	2	2
3	4	1	36	10	11	15	3	2	3
1	5	1	46	12	7	27	5	2	1
4	3	4	46	12	12	22	4	3	2
3	2	1	39	7	11	21	3	1	1
2	3	3	48	13	10	25	4	3	3
1	5	1	44	4	5	35	5	1	1
3	5	1	49	14	6	29	5	3	1
2	4	2	48	10	10	28	4	4	2
5	5	1	51	16	5	30	5	1	1
2	3	2	44	13	9	22	3	3	2
3	5	1	47	15	5	27	5	3	1
5	5	5	44	4	5	35	5	1	1
3	4	2	43	11	10	22	3	3	3
2	3	1	38	7	6	25	4	1	1
2	5	2	51	10	12	29	5	2	3
2	3	1	48	10	13	25	4	3	2
3	3	2	48	14	15	19	2	5	3
3	2	2	45	16	11	18	2	5	3
3	3	3	48	12	15	21	3	3	3
4	4	1	51	14	10	27	5	4	1
1	4	1	47	7	6	34	5	1	1
1	2	1	40	8	6	26	4	2	1
2	1	1	41	13	7	21	3	4	1
1	3	1	42	5	5	32	5	1	1
2	4	3	39	11	8	20	4	3	2
5	5	5	60	11	14	35	5	2	2
2	3	1	43	9	6	28	4	3	1
2	3	1	57	16	7	34	5	2	1
3	4	3	45	9	10	26	4	3	2
1	5	1	44	4	5	35	5	1	1
5	5	1	44	4	5	35	5	1	1
3	3	1	39	7	5	27	5	1	1
4	4	4	64	16	20	28	4	4	4
2	2	1	44	12	8	24	4	4	2
1	5	1	40	6	5	29	5	1	1
2	2	2	37	9	9	19	3	2	2
5	5	5	69	17	20	32	5	5	5
1	5	1	45	6	5	34	5	2	1
1	3	1	43	10	8	25	4	3	2
5	5	1	44	7	6	31	5	1	1
1	5	1	44	4	5	35	5	1	1
2	5	1	39	5	6	28	4	1	1
1	5	1	43	4	5	34	5	1	1
2	3	1	51	12	7	32	5	3	1

1	4	1	42	9	7	26	5	3	1
1	5	1	39	4	5	30	5	1	1
2	4	2	39	4	5	30	5	1	1
2	4	1	41	8	10	23	4	2	2
2	4	2	45	9	10	26	4	2	2
1	4	1	43	12	8	23	4	2	1
2	4	1	45	8	10	27	4	2	2
2	3	2	42	9	8	25	5	2	2
3	3	3	51	13	16	22	3	3	4
1	5	1	42	6	5	31	5	1	1
1	3	1	41	5	5	31	5	1	1
3	5	1	39	7	5	27	4	2	1
1	5	1	42	5	5	32	5	1	1
3	3	3	48	12	15	21	3	3	3
1	5	1	42	4	5	33	5	1	1
4	4	1	50	11	7	32	4	2	1
1	1	1	41	7	5	29	5	1	1
3	4	1	41	7	6	28	4	1	1
3	3	3	48	12	15	21	3	3	3
5	5	5	44	8	5	31	5	1	1
3	4	1	50	11	8	31	5	4	2
2	3	1	44	10	11	23	4	4	2
1	4	1	48	12	5	31	5	3	1
3	4	1	48	13	10	25	4	4	2
3	2	3	48	13	16	19	3	3	3
2	2	1	41	10	10	21	3	3	2
3	5	1	37	9	7	21	4	2	2
3	3	3	48	12	15	21	3	3	3
1	4	1	39	7	7	25	4	2	1
2	4	1	46	11	10	25	4	4	2
1	5	1	46	8	9	29	5	2	2
2	3	2	38	11	8	19	3	3	2
5	5	1	48	10	5	33	5	2	1
2	4	1	45	9	7	29	4	2	2
3	4	3	45	9	10	26	4	2	2
3	5	1	43	11	11	21	3	3	4
3	5	1	37	4	5	28	5	1	1
3	4	2	46	9	10	27	4	3	2
2	4	1	50	14	14	22	4	4	3
2	4	1	41	6	6	29	5	2	1
1	1	2	49	13	9	27	5	5	3
1	4	1	38	6	5	27	5	2	1
1	5	1	43	4	5	34	5	1	1
1	5	1	44	10	6	28	5	3	1
1	4	1	39	8	6	25	4	3	1
3	3	1	40	12	5	23	4	2	1
1	5	1	46	7	5	34	5	2	1
1	5	1	44	4	5	35	5	1	1
3	3	1	31	5	6	20	3	1	1
1	5	5	49	9	5	35	5	1	1
3	4	2	39	10	6	23	4	1	1
2	5	1	35	6	5	24	5	1	1
1	4	1	42	6	9	27	5	1	1
4	4	1	33	4	5	24	4	1	1
1	4	1	40	7	5	28	4	1	1
1	3	1	49	15	14	20	3	3	3

2	4	1	50	12	11	27	4	3	2
5	3	1	45	12	7	26	5	3	1
5	5	5	48	11	15	22	4	2	3
2	3	1	40	10	10	20	3	3	2
1	3	1	39	5	5	29	5	1	1
1	4	1	35	4	5	26	5	1	1
3	3	3	36	5	7	24	3	1	1
3	3	3	48	12	15	21	3	3	3
3	4	1	39	5	12	22	3	1	3
1	5	1	38	4	5	29	5	1	1
2	4	2	38	6	5	27	4	2	1
4	4	1	42	9	5	28	5	1	1
5	5	2	53	8	10	35	5	1	2
1	5	1	40	5	6	29	5	1	1
2	5	1	53	15	9	29	5	4	1
1	5	1	39	10	7	22	4	5	1
2	3	2	42	12	10	20	2	5	2
2	2	1	45	13	9	23	4	4	1
1	3	1	42	8	5	29	5	1	1
5	5	5	80	20	25	35	5	5	5
1	3	1	51	17	16	18	3	5	4
3	2	3	46	10	15	21	3	2	3
3	5	1	52	12	5	35	5	5	1
3	4	2	41	9	7	25	4	2	2
5	5	1	44	4	5	35	5	1	1
1	4	1	43	9	7	27	4	2	1
3	5	1	41	7	5	29	5	3	1
4	4	4	64	16	20	28	4	4	4
1	5	2	49	8	10	31	5	2	2
1	5	1	47	11	7	29	4	3	1
3	4	1	46	13	7	26	4	4	2
3	3	1	48	12	15	21	3	3	3
2	4	1	41	8	9	24	4	2	2
1	5	1	39	5	5	29	5	1	1
1	5	1	44	4	5	35	5	1	1
2	3	1	37	7	6	24	4	2	1
2	4	2	42	8	7	27	4	2	2
1	5	1	44	4	5	35	5	1	1
2	4	1	40	11	6	23	4	3	1
3	2	1	44	9	6	29	5	3	1
2	4	1	49	10	11	28	4	3	2
2	5	2	51	10	6	35	5	3	1
1	5	1	46	4	9	33	5	1	5
2	4	2	42	11	10	21	3	2	2
2	4	1	42	6	9	27	4	2	2
2	4	1	41	11	10	20	4	2	2
1	5	1	44	4	5	35	5	1	1
1	5	1	47	8	5	34	5	2	1
1	4	2	43	10	7	26	4	2	1
1	5	1	46	4	9	33	5	1	1
1	5	1	43	5	5	33	5	1	1
5	5	1	43	5	5	33	5	1	1
1	5	1	41	6	5	30	5	1	1
1	5	1	36	7	5	24	5	4	1
2	3	1	38	10	5	23	4	2	1
1	4	1	48	7	7	34	5	2	1

2	3	1	40	9	12	19	2	3	2
2	5	2	46	6	5	35	5	2	1
1	5	1	44	4	5	35	5	1	1
1	5	1	43	5	5	33	5	1	1
3	4	4	59	14	19	26	4	3	4
3	3	3	48	12	15	21	3	3	3
5	5	5	62	17	16	29	4	4	4
1	2	1	44	4	5	35	5	1	1
3	3	3	48	12	15	21	3	3	3
4	4	4	50	14	11	25	4	4	2
2	5	1	42	12	6	24	5	5	1
1	5	1	36	6	5	25	5	1	1
2	4	2	51	10	15	26	4	2	2
2	4	1	46	8	10	28	4	2	2
2	4	1	40	8	6	26	5	1	1
2	4	1	45	10	7	28	4	3	2
2	4	1	40	6	6	28	5	1	1
1	4	1	50	12	11	27	5	3	1
2	5	2	48	10	13	25	5	2	2
2	3	1	49	12	13	24	5	3	3
1	3	1	38	6	6	26	4	2	2
2	5	1	34	5	6	23	5	2	2
2	4	1	40	5	8	27	4	1	1
1	4	1	44	7	5	32	5	3	1
1	4	1	41	4	5	32	5	1	1
2	3	2	50	11	13	26	4	2	3
3	3	2	47	12	14	21	3	3	3
4	1	1	46	12	10	24	4	3	2
2	5	1	49	12	10	27	4	2	2
1	5	1	47	7	5	35	5	1	1
2	3	2	43	13	8	22	3	4	2
1	5	1	45	6	5	34	5	1	1
5	1	5	44	4	5	35	5	1	1
4	4	4	49	12	15	22	4	3	3
1	5	1	44	4	5	35	5	1	1
2	4	1	48	9	11	28	4	2	3
5	5	1	44	4	5	35	5	1	1
2	4	1	40	13	7	20	3	5	1
2	5	1	48	14	6	28	4	4	1
1	4	1	46	11	11	24	5	3	3
2	4	1	42	8	10	24	4	2	2
1	5	1	44	4	5	35	5	1	1
1	4	1	40	9	5	26	5	2	1
1	3	1	41	7	5	29	5	2	1
1	5	1	43	4	5	34	5	1	1
2	4	1	39	8	8	23	4	3	3
3	4	3	50	12	15	23	4	3	3
1	5	1	51	16	9	26	5	4	3
2	4	1	40	9	5	26	4	3	1
1	5	1	42	4	5	33	5	1	1
2	5	1	50	13	6	31	5	4	1
2	3	1	42	8	10	24	4	2	2
1	5	1	41	6	5	30	5	1	1
3	3	1	48	12	15	21	3	3	3
5	5	1	44	9	5	30	5	1	1
1	4	1	41	8	7	26	4	1	1

1	5	1	44	4	5	35	5	1	1
1	3	1	40	5	6	29	5	2	2
2	3	1	43	13	8	22	3	3	1
1	5	1	44	4	5	35	5	1	1
3	3	1	43	5	5	33	5	1	1
1	3	1	38	4	5	29	5	1	1
3	2	1	46	12	13	21	3	4	3
2	3	4	48	14	15	19	2	3	4
1	3	1	49	10	7	32	5	1	1
1	5	1	56	8	13	35	5	1	1
3	5	1	42	5	5	32	5	1	1
2	4	1	51	14	10	27	4	4	2
1	5	1	41	5	5	31	5	1	1
4	3	3	52	12	13	27	4	3	3
5	3	1	50	12	15	23	5	3	3
1	4	1	36	7	6	23	4	3	2
1	5	1	45	8	6	31	5	2	1
2	3	1	43	12	13	18	3	4	3
5	5	5	46	4	7	35	5	1	1
1	5	1	43	5	5	33	5	1	1
4	4	1	57	12	10	35	5	1	1
5	5	1	52	8	9	35	5	1	1
3	2	1	40	10	8	22	3	3	1
3	3	3	40	11	8	21	4	3	2
2	4	1	44	11	9	24	4	3	2
1	3	1	41	6	5	30	5	1	1
2	4	1	40	5	5	30	4	1	1
2	2	2	41	15	12	14	1	5	3
2	4	2	38	6	6	26	4	2	1
2	4	2	45	12	9	24	3	4	2
1	5	1	48	7	6	35	5	3	2
1	4	4	56	16	14	26	4	4	2
1	5	1	40	4	5	31	5	1	1
3	4	1	45	13	9	23	4	4	2
3	4	1	41	9	7	25	4	2	2
2	4	1	57	15	18	24	5	5	5
3	3	2	41	12	9	20	3	3	2
1	2	1	45	10	18	17	3	4	4
1	5	1	39	4	5	30	5	1	1
5	5	5	68	16	17	35	5	1	1
1	3	1	42	9	7	26	4	2	1
3	4	2	41	11	6	24	4	3	1
2	3	1	48	12	15	21	3	3	3
5	5	1	56	15	12	29	5	4	2
3	3	3	48	12	15	21	3	3	3
2	3	2	43	10	10	23	4	2	2
2	4	2	51	12	12	27	4	3	2
1	4	1	47	12	13	22	4	3	1
4	5	2	50	15	11	24	4	3	3
1	5	1	40	4	5	31	5	1	1
3	4	1	41	12	8	21	4	3	2
2	3	2	41	8	7	26	4	2	1
1	5	1	44	6	5	33	5	1	1
1	5	1	43	4	5	34	5	1	1
3	5	1	45	9	5	31	5	3	1
1	5	1	44	4	5	35	5	1	1

3	4	1	47	9	8	30	5	3	2
1	5	1	46	6	7	33	5	1	1
1	5	1	52	9	9	34	5	3	3
3	5	1	48	14	8	26	4	4	2
1	4	1	38	5	7	26	5	2	1
2	3	1	34	7	5	22	4	1	1
2	4	1	38	6	5	27	5	2	1
4	4	1	39	15	10	14	3	5	1
2	5	2	44	10	5	29	4	4	1
1	4	1	46	12	14	20	4	3	3
1	5	1	41	8	10	23	5	2	2
3	4	1	49	7	7	35	5	2	1
1	5	1	39	5	7	27	4	1	3
1	5	1	49	11	6	32	5	5	1
1	4	1	38	4	5	29	5	1	1
3	4	1	44	8	5	31	5	2	1
1	5	1	51	9	7	35	5	1	1
4	4	1	38	8	10	20	4	2	2
5	5	5	80	20	25	35	5	5	5
1	4	1	38	5	5	28	4	1	1
2	5	1	50	13	14	23	5	5	2
2	4	1	46	8	10	28	4	2	2
5	5	1	49	9	5	35	5	5	1
1	4	1	39	6	6	27	5	1	2
1	4	4	36	5	5	26	4	1	1
1	5	1	51	11	5	35	5	1	1
5	5	5	80	20	25	35	5	5	5
1	5	1	51	8	8	35	5	1	1
3	5	1	53	14	11	28	5	3	2
1	4	1	45	11	7	27	4	3	1
2	3	1	46	11	9	26	4	3	2
1	3	1	44	7	8	29	5	2	2
3	3	1	47	11	9	27	4	3	2
3	2	2	45	12	10	23	4	3	3
1	5	1	41	4	5	32	5	1	1
3	5	2	48	11	13	24	5	3	2
2	4	2	40	9	6	25	4	3	1
1	5	1	45	5	5	35	5	1	1
5	5	1	49	16	7	26	5	5	1
2	4	2	44	8	9	27	4	2	1
1	5	1	48	9	6	33	5	1	1
2	3	1	45	12	12	21	4	3	2
3	4	3	48	12	10	26	4	3	2
3	4	3	51	12	13	26	4	3	3
2	5	2	34	4	5	25	5	1	1
2	4	2	48	18	6	24	4	5	2
2	4	2	45	11	10	24	4	2	2
2	4	1	38	8	6	24	5	2	1
5	5	5	80	20	25	35	5	5	5
4	4	1	46	12	9	25	4	1	3
2	4	1	38	8	9	21	3	1	2
2	4	1	41	8	6	27	4	2	1
3	3	3	48	12	15	21	3	3	3
2	4	2	47	13	12	22	4	3	2
1	5	1	46	10	6	30	5	1	1
2	3	1	44	10	10	24	3	3	2

3	3	3	48	12	15	21	3	3	3
4	3	1	44	11	10	23	3	3	2
3	4	2	43	11	7	25	4	2	2
3	3	1	44	8	5	31	3	3	1
4	4	3	49	12	8	29	4	3	1
3	5	1	40	8	5	27	5	2	1
1	3	1	43	7	5	31	5	1	1
3	3	1	48	11	10	27	5	4	3
1	5	1	43	9	6	28	4	2	1
1	4	3	39	4	5	30	5	1	1
3	5	2	44	9	7	28	5	5	2
1	5	1	44	4	5	35	5	1	1
4	4	4	64	16	20	28	4	4	4
3	5	1	36	6	6	24	5	3	1
1	5	1	44	4	5	35	5	1	1
2	3	1	43	13	8	22	3	4	2
1	5	1	49	13	8	28	4	2	1
2	3	1	40	7	5	28	4	2	1
4	4	4	62	16	18	28	4	4	4
2	3	2	47	10	11	26	4	2	2
1	5	1	56	9	12	35	5	1	1
2	2	1	42	9	8	25	3	2	3
1	5	1	48	9	5	34	5	1	1
2	4	1	41	8	5	28	5	2	1
4	4	4	40	8	6	26	4	2	1
3	2	2	35	10	10	15	2	3	2
5	5	5	80	20	25	35	5	5	5
2	3	1	40	10	6	24	4	4	1
3	5	1	53	12	8	33	5	3	2
2	4	2	53	15	14	24	4	4	2
4	4	2	53	12	15	26	4	3	3
2	4	1	45	10	12	23	4	2	2
5	5	1	42	8	5	29	5	4	1
2	4	1	39	8	5	26	4	2	1
1	4	1	44	4	5	35	5	1	1
1	4	1	35	5	5	25	4	1	1
1	3	1	50	13	5	32	5	1	1
3	3	2	42	10	10	22	3	2	3
1	5	1	42	4	5	33	5	1	1
2	1	1	45	12	7	26	4	3	1
1	5	1	51	12	5	34	5	3	1
1	3	1	42	6	7	29	4	2	2
1	5	1	60	12	13	35	5	1	1
4	4	1	64	16	20	28	4	4	4
1	3	1	43	9	5	29	5	2	1
2	4	1	43	9	7	27	4	2	1
2	4	1	41	9	5	27	4	2	1
3	3	2	47	15	15	17	3	5	3
2	5	2	48	9	9	30	5	2	2
4	4	4	64	16	20	28	4	4	4
3	3	3	44	11	13	20	3	3	1
3	3	2	51	12	15	24	3	3	3
3	3	1	34	4	5	25	5	1	1
2	4	2	42	11	10	21	3	4	2
1	5	1	44	4	5	35	5	1	1
2	4	1	40	7	8	25	5	2	1

1	5	1	40	5	5	30	5	1	1
2	3	1	43	11	8	24	3	3	3
3	3	3	32	9	7	16	3	3	2
5	5	1	58	11	12	35	5	4	2
2	3	2	39	7	5	27	4	2	1
5	5	2	48	9	10	29	5	3	2
1	4	1	41	8	6	27	4	2	1
1	3	1	48	13	9	26	5	3	3
2	3	1	47	6	7	34	5	3	1
1	3	1	36	5	7	24	5	1	1
1	2	1	35	4	5	26	4	1	1
1	5	1	47	7	11	29	5	1	1
2	4	2	40	8	8	24	4	2	2
5	5	1	44	4	7	33	5	1	1
5	5	5	61	16	16	29	4	5	5
1	5	1	43	9	6	28	5	3	2
1	4	1	34	5	5	24	3	1	1
2	4	1	42	8	7	27	4	2	2
3	5	2	48	12	10	26	4	3	3
2	4	2	46	8	10	28	4	2	2
5	5	5	76	16	25	35	5	1	5
1	5	1	44	4	5	35	5	1	1
5	5	5	80	20	25	35	5	5	5
2	5	1	49	10	6	33	5	5	1
3	5	1	50	12	15	23	5	3	3
1	5	1	44	4	5	35	5	1	1
1	5	1	33	4	5	24	5	1	1
2	4	1	41	10	9	22	3	3	2
1	5	1	44	4	5	35	5	1	1
2	3	1	41	10	8	23	4	1	1
1	5	2	40	4	5	31	5	1	1
3	4	1	45	13	13	19	2	4	3
2	4	1	41	7	7	27	4	1	1
2	3	1	47	13	13	21	3	4	3
2	2	1	40	16	10	14	2	4	2
2	4	1	38	8	8	22	4	2	2
3	3	2	46	12	14	20	3	3	3
1	5	1	44	4	5	35	5	1	1
1	3	1	41	9	6	26	4	2	1
4	4	4	64	16	20	28	4	4	4
1	5	1	47	8	5	34	5	1	1
2	4	4	48	8	13	27	4	2	2
4	4	1	45	8	7	30	5	3	1
1	4	1	37	4	5	28	4	1	1
3	4	1	46	16	17	13	2	4	4
2	4	1	46	11	10	25	4	3	2
1	5	1	42	4	5	33	5	1	1
2	3	1	43	15	6	22	4	5	1
1	5	1	44	4	5	35	5	1	1
5	5	5	36	4	5	27	5	1	1
2	4	1	41	11	8	22	4	3	2
2	3	1	38	9	7	22	3	2	2
3	3	3	46	12	13	21	3	3	1
2	4	1	41	8	7	26	4	2	2
3	4	2	49	16	11	22	3	4	2
3	3	2	42	10	11	21	3	3	3

5	5	5	80	20	25	35	5	5	5
1	5	1	44	4	5	35	5	1	1
1	5	1	45	12	5	28	4	5	1
5	5	1	51	13	5	33	5	5	1
4	4	2	41	8	10	23	4	2	2
2	4	1	44	10	8	26	4	2	1
2	2	2	45	13	7	25	4	3	2
2	3	3	49	13	14	22	3	4	3
3	3	3	48	12	15	21	3	3	3
5	4	1	46	16	5	25	4	5	1
2	5	1	43	5	6	32	5	1	1
4	4	4	42	9	9	24	3	2	2
1	2	1	40	10	7	23	4	2	1
2	3	2	38	9	7	22	3	3	1
5	5	1	39	4	6	29	5	1	1
3	4	3	50	12	16	22	4	3	4
2	3	1	40	10	9	21	4	2	2
5	5	5	58	12	11	35	5	5	2
1	4	1	46	10	8	28	4	1	1
1	3	1	36	10	5	21	3	3	1
2	4	2	46	8	10	28	4	2	2
3	3	1	37	9	8	20	3	3	1
1	5	1	43	4	5	34	5	1	1
1	5	1	44	4	5	35	5	1	1
4	4	2	56	16	12	28	4	4	2
2	4	1	45	11	11	23	4	2	2
1	3	1	47	11	15	21	3	3	3
2	4	1	36	7	5	24	4	1	1
1	3	1	43	4	5	34	5	1	1
3	3	2	43	9	10	24	3	3	2
1	3	1	42	6	8	28	5	2	1
1	3	1	40	7	6	27	5	2	1
4	2	2	51	15	12	24	4	5	3
1	4	1	38	7	5	26	4	1	1
1	5	1	44	4	5	35	5	1	1
2	3	2	41	8	8	25	4	2	2
2	4	1	44	11	11	22	4	3	2
2	3	1	38	8	8	22	4	2	1
1	5	1	44	4	5	35	5	1	1
2	5	1	46	11	10	25	4	3	2
3	3	1	48	12	14	22	4	3	3
1	4	1	40	12	6	22	4	3	1
1	4	1	43	9	5	29	5	3	1
2	4	1	43	11	5	27	4	3	1
1	5	1	44	4	5	35	5	1	1
2	4	2	49	15	12	22	4	5	3
1	4	1	42	9	6	27	4	3	2
2	4	1	48	10	12	26	4	2	2
5	4	4	66	16	20	30	4	4	4
2	5	1	51	12	8	31	5	4	4
5	5	1	39	8	5	26	5	1	1
5	5	5	60	12	13	35	5	5	5
3	4	1	41	10	9	22	3	3	1
1	5	1	39	4	7	28	5	1	1
4	4	2	47	10	10	27	4	4	2
1	5	1	50	13	11	26	5	5	1

5	5	5	51	8	9	34	5	1	1
5	5	5	44	4	5	35	5	1	1
5	5	1	41	5	6	30	5	2	1
2	2	1	34	5	5	24	5	1	1
2	4	2	42	10	10	22	3	2	2
2	4	2	46	9	10	27	4	3	2
4	4	2	51	13	15	23	4	4	3
2	4	1	44	9	9	26	4	2	2
2	3	2	38	8	10	20	3	2	2
2	4	1	43	9	5	29	5	1	1
1	5	1	40	5	5	30	5	1	1
2	4	2	41	9	10	22	4	2	2
1	5	1	44	4	5	35	5	1	1
1	5	1	42	4	5	33	5	1	1
3	4	1	44	10	9	25	4	3	1
2	3	2	46	10	10	26	4	2	2
2	3	1	52	12	15	25	5	3	3
3	4	1	38	7	5	26	4	1	1
5	5	5	43	6	5	32	5	1	1
2	4	1	39	4	6	29	5	1	1
1	5	1	48	8	5	35	5	1	1
2	5	1	42	11	10	21	3	3	4
4	4	4	48	12	15	21	3	3	3
1	4	1	49	12	15	22	4	3	3
3	3	2	47	9	10	28	4	2	2
3	3	3	39	8	10	21	3	2	2
1	5	1	52	10	10	32	5	2	4
1	5	1	40	4	5	31	5	1	1
3	5	3	43	4	5	34	5	1	1
4	4	4	58	16	25	17	2	5	5
1	4	1	44	7	5	32	5	1	1
1	5	1	42	4	5	33	5	1	1
1	4	1	42	4	5	33	5	1	1
2	4	1	40	5	6	29	5	1	1
3	5	1	48	10	5	33	5	1	1
1	4	1	35	6	6	23	4	2	1
2	3	3	48	12	15	21	3	3	3
2	5	1	50	17	14	19	4	5	4
2	3	2	45	10	13	22	3	2	3
3	4	4	54	14	15	25	3	4	2
1	3	1	33	5	5	23	4	1	1
5	5	1	43	9	5	29	5	2	1
1	4	1	43	4	5	34	5	1	1
2	3	1	39	6	8	25	4	1	1
1	5	1	44	4	5	35	5	1	1
2	3	3	43	11	10	22	4	3	2
2	4	1	41	9	5	27	4	3	1
5	5	5	80	20	25	35	5	5	5
5	5	5	64	18	19	27	5	5	5
5	3	1	55	15	15	25	4	4	2
2	2	1	44	14	15	15	3	3	4
1	5	1	39	4	5	30	5	1	1
2	4	2	46	11	10	25	4	3	2
2	4	2	47	11	10	26	4	2	2
2	3	1	40	8	11	21	3	2	2
1	4	1	46	8	10	28	4	2	2

1	3	1	41	9	5	27	5	4	1
2	4	1	38	6	5	27	4	2	1
4	4	4	45	11	8	26	4	2	1
5	5	5	80	20	25	35	5	5	5
2	3	1	45	10	10	25	4	2	2
1	5	1	44	4	5	35	5	1	1
3	3	3	57	14	15	28	5	3	3
3	2	1	39	9	9	21	4	2	2
1	5	1	44	4	5	35	5	1	1
2	4	1	37	7	5	25	5	1	1
1	4	1	38	6	5	27	4	2	1
1	4	1	43	6	6	31	5	2	2
1	4	1	35	5	5	25	4	1	1
1	5	1	46	8	9	29	5	1	1
2	5	1	46	7	5	34	5	1	1
2	4	2	51	13	13	25	4	2	2
4	3	1	35	8	8	19	3	2	2
2	4	2	44	9	10	25	4	3	2
4	4	1	43	6	5	32	5	1	1
1	4	2	44	8	6	30	5	2	1
2	4	2	50	13	11	26	4	3	3
1	4	1	38	4	5	29	5	1	1
4	5	1	41	4	5	32	5	1	1
2	2	1	37	8	10	19	3	2	2
1	4	1	41	11	9	21	4	3	2
1	5	1	44	4	5	35	5	1	1
4	5	4	70	18	23	29	5	4	5
1	4	1	44	6	7	31	4	2	1
1	5	1	46	11	5	30	4	3	1
1	5	1	43	6	5	32	5	2	1
2	4	1	39	6	6	27	5	2	1
1	4	1	37	6	5	26	5	2	1
1	5	1	44	4	5	35	5	1	1
1	5	1	44	10	6	28	5	4	2
3	3	2	43	10	10	23	4	3	2
3	5	1	54	13	12	29	5	5	3
5	5	1	41	5	7	29	5	2	1
3	4	2	48	12	11	25	4	3	2
1	5	3	49	9	8	32	5	3	1
1	5	1	43	8	5	30	5	3	1
2	3	1	44	11	9	24	4	3	2
4	5	1	50	12	14	24	5	4	2
2	4	1	44	12	9	23	4	2	2
2	4	1	42	12	9	21	3	3	3
1	5	1	44	4	5	35	5	1	1
1	4	1	39	8	8	23	4	2	1
2	4	2	48	14	12	22	4	4	3
2	4	2	43	9	10	24	4	2	2
1	2	1	45	11	7	27	4	2	1

b4	b5	b6	b7	b8	b9	b10	b11	b12	b13	b14	b15	b16	totaldelay
5	1	1	5	1	1	1	5	1	1	5	5	5	55
4	2	1	4	1	1	1	4	2	1	3	3	3	62
5	3	1	4	2	1	1	5	1	1	5	5	5	66
3	4	1	2	5	2	1	3	5	1	3	2	3	54
3	2	1	4	1	2	2	3	1	2	4	3	3	66
3	3	1	3	3	1	3	4	2	2	4	4	4	69
5	3	1	3	3	1	1	3	3	1	3	3	4	57
4	2	1	2	1	2	2	3	2	1	2	2	4	61
3	3	3	3	3	3	3	3	3	3	3	3	3	57
5	4	5	5	3	2	3	4	5	2	4	4	4	72
4	2	1	2	1	1	1	4	1	1	4	4	3	53
3	3	3	3	3	3	3	4	3	3	3	3	3	56
4	3	2	3	2	2	2	4	2	2	4	4	3	69
3	3	3	3	3	3	2	3	3	3	3	3	2	64
5	1	1	4	3	1	1	4	1	1	4	4	4	53
3	3	1	4	2	1	2	5	2	1	3	3	3	63
3	3	1	2	2	2	2	4	3	1	3	4	4	65
3	4	1	4	1	1	1	5	3	1	5	5	5	67
3	2	1	4	1	1	1	5	2	1	5	4	3	58
3	4	1	3	4	4	1	3	5	1	3	3	2	61
4	4	1	4	3	2	1	4	2	1	4	4	4	62
5	1	1	5	1	1	1	5	1	1	5	5	5	87
3	3	3	3	3	3	3	3	3	3	3	3	3	70
4	1	1	5	1	1	1	5	1	1	5	4	3	57
3	3	2	3	4	3	2	3	3	2	3	3	3	58
5	3	2	5	2	2	1	4	2	1	4	4	4	63
4	2	1	3	2	2	1	4	1	1	4	4	4	60
3	3	2	2	3	3	1	3	3	2	3	3	3	72
4	3	1	4	2	1	1	4	3	1	4	3	4	60
3	3	1	5	3	1	1	4	3	1	3	3	3	62
4	3	2	4	3	2	1	4	3	1	4	4	4	58
5	2	1	5	2	1	1	5	2	1	5	5	5	59
3	4	3	3	4	4	3	3	4	3	3	3	2	64
3	4	2	4	2	3	1	4	2	1	2	1	2	47
5	2	1	5	2	1	1	4	3	1	5	4	4	63
3	3	1	1	1	1	1	4	1	1	5	4	4	75
4	3	2	3	2	2	1	4	2	1	4	4	4	59
3	3	3	3	3	3	3	3	3	3	3	3	3	57
4	2	2	3	2	3	1	4	2	2	4	4	3	61
2	3	3	3	3	3	3	3	3	3	3	3	3	57
3	4	3	3	4	3	3	3	3	2	3	3	3	57
3	3	3	3	3	3	3	3	3	3	3	3	3	57
3	3	1	2	4	4	1	2	4	2	4	2	2	57
5	1	1	3	1	1	1	5	1	1	5	5	5	50
4	3	2	3	3	2	1	4	2	1	4	3	3	59
4	3	1	4	1	1	1	5	2	1	4	4	4	66
3	2	1	4	2	2	1	4	2	1	3	3	3	61
5	5	5	5	5	5	5	5	5	5	5	5	5	95
4	3	2	3	2	2	2	4	2	2	4	4	3	57
4	3	1	4	2	1	1	5	2	1	4	4	4	59

5	2	1	4	1	1	1	5	3	1	4	5	5	71
3	3	4	4	4	3	2	3	2	3	2	2	3	61
4	2	1	1	1	1	1	3	1	1	4	4	3	53
4	4	1	4	2	2	1	5	3	1	4	4	4	58
4	2	1	4	1	1	1	4	2	1	4	4	4	58
4	2	2	4	2	2	2	4	2	2	4	4	4	59
3	3	1	3	2	3	1	4	2	1	4	3	2	56
4	4	2	4	3	2	2	4	4	3	3	3	3	64
2	2	2	2	2	2	2	3	2	2	3	3	3	40
4	2	2	4	1	2	1	4	3	2	4	4	4	62
3	3	3	3	3	3	2	4	3	3	3	3	3	56
5	2	1	4	1	1	1	5	2	1	4	5	5	56
3	2	1	3	1	1	1	4	1	1	4	4	4	56
3	5	1	3	3	5	1	5	5	1	5	3	3	68
3	4	2	2	3	3	1	4	3	1	4	3	4	61
4	3	2	4	2	2	2	4	2	2	4	4	4	56
4	3	1	2	2	3	2	4	2	2	2	4	4	64
4	2	1	1	1	1	1	4	2	1	4	4	4	50
3	2	1	2	3	3	1	3	2	1	3	3	2	54
3	3	1	2	2	2	1	4	1	1	4	3	3	60
4	3	2	4	2	3	3	4	2	2	4	3	3	57
4	4	1	3	3	1	1	5	3	1	5	4	4	61
5	1	1	3	1	1	1	5	1	1	5	5	5	59
5	2	1	1	1	1	1	3	1	1	5	5	5	53
3	2	1	3	1	1	1	3	1	1	4	3	3	43
3	3	3	3	3	3	3	3	3	2	3	3	2	64
4	1	1	3	1	1	1	3	1	1	4	3	4	56
4	2	3	3	2	3	2	4	3	2	3	4	4	52
4	3	1	3	1	1	1	4	1	1	4	4	4	57
3	2	1	3	2	2	1	4	3	1	4	3	3	57
4	3	2	3	2	3	2	4	4	2	4	4	4	62
3	2	2	4	3	2	1	3	2	1	4	4	4	67
3	3	3	3	3	3	2	3	2	2	3	3	3	56
3	3	1	3	3	3	2	3	4	1	3	3	3	68
3	1	1	3	1	1	1	4	1	1	5	5	4	52
3	3	3	3	3	3	3	3	3	3	3	3	3	57
5	1	1	5	1	1	1	5	1	1	5	5	5	76
4	2	1	3	2	1	1	4	2	1	4	3	3	60
3	3	2	3	3	3	3	3	3	3	3	3	3	70
5	1	1	5	1	1	1	5	1	1	5	5	5	61
3	4	2	2	3	3	2	4	3	1	3	2	3	67
3	3	1	3	1	1	1	3	1	2	3	3	3	50
4	3	2	4	2	2	2	4	2	2	4	4	3	62
5	4	1	5	2	2	1	4	2	1	5	5	4	60
5	1	1	5	1	1	1	5	1	1	5	5	5	55
4	3	1	5	1	1	1	5	2	1	5	5	5	69
3	3	2	3	2	2	2	3	2	1	3	3	3	54
3	3	2	2	2	3	2	3	4	2	4	3	2	62
3	4	2	3	4	3	1	5	2	1	4	4	4	59
3	2	2	3	2	2	3	3	2	2	3	3	3	51
3	1	1	3	1	1	1	3	1	1	3	3	3	51
4	2	2	4	2	2	2	4	2	1	4	4	5	65
4	3	2	4	2	2	2	4	2	2	4	4	4	62
4	4	1	4	1	2	1	5	2	1	5	5	5	59
3	2	1	4	2	1	1	5	2	1	4	4	4	62
5	2	1	4	1	1	1	5	1	1	5	5	5	56

5	2	1	5	1	1	1	5	1	1	5	5	5	64
3	4	2	3	3	3	3	3	3	2	3	3	2	53
2	3	1	3	4	3	1	4	3	1	4	4	2	59
5	1	1	2	1	1	1	5	1	1	5	5	5	61
3	3	2	3	2	1	1	4	2	1	3	4	4	65
5	1	1	5	1	1	1	4	1	1	1	5	5	55
4	3	1	3	1	1	1	5	1	1	4	5	5	56
4	2	1	4	3	2	1	4	1	1	4	4	4	53
3	3	4	2	4	2	3	4	2	1	4	2	4	58
4	2	1	4	2	2	2	4	2	2	4	4	4	58
5	2	1	5	1	1	1	5	2	1	5	5	4	62
3	2	1	2	2	2	2	4	2	1	4	3	4	64
4	3	2	3	2	2	2	4	2	2	4	4	4	61
5	2	1	4	2	2	1	4	1	1	5	4	4	73
3	3	3	3	3	3	1	3	3	2	3	3	3	58
3	3	2	2	2	2	2	4	2	1	3	3	3	55
5	5	5	5	5	5	5	5	5	5	5	5	5	95
4	3	2	3	3	2	1	3	3	1	3	3	3	62
5	1	1	5	1	1	1	5	1	1	5	5	5	59
5	3	1	3	1	3	1	4	3	1	5	3	2	61
4	3	2	3	2	1	1	4	2	1	4	4	4	57
3	3	3	3	3	4	3	4	3	3	2	3	3	60
4	3	1	2	1	1	1	4	1	1	4	3	3	56
4	3	2	3	2	2	2	3	2	2	4	4	4	61
3	3	2	3	2	2	2	3	2	3	3	3	3	67
4	2	1	4	1	3	1	4	1	1	4	3	4	51
4	1	1	4	1	1	1	5	5	1	5	5	5	66
3	2	2	3	2	2	1	4	2	1	4	4	4	60
5	5	5	3	5	5	1	1	5	2	5	5	1	75
4	4	1	5	2	1	1	3	5	1	3	2	2	51
3	3	2	3	3	2	3	3	2	2	3	3	3	55
3	4	2	2	3	3	2	4	3	2	3	3	3	59
4	2	2	3	2	2	2	4	3	2	3	3	3	62
3	3	4	2	5	5	5	5	5	4	5	4	4	56
4	2	2	3	2	2	2	4	2	2	4	4	4	59
4	4	1	3	2	2	2	4	3	1	4	4	4	65
2	2	2	3	2	2	2	4	3	2	4	4	4	72
4	3	1	4	3	3	2	4	3	1	4	5	4	55
5	5	1	2	3	1	1	3	2	1	5	5	5	65
3	5	5	1	5	5	5	5	5	3	5	3	1	58
3	3	2	2	4	3	3	4	3	2	3	3	3	55
3	4	1	2	2	2	1	3	4	1	3	3	3	65
3	3	3	3	3	3	3	3	3	3	3	3	3	62
3	2	2	3	2	2	1	4	2	2	4	4	4	61
3	3	3	3	3	3	3	4	2	1	3	3	3	61
4	2	1	3	1	1	1	4	1	1	4	4	4	62
5	2	2	4	2	2	2	4	2	1	5	5	3	60
3	3	2	3	2	2	2	3	2	1	3	3	3	59
5	1	1	5	1	1	1	5	1	1	5	5	5	62
5	5	5	5	5	5	5	5	5	5	5	5	5	95
5	2	1	4	1	1	1	5	3	1	5	5	5	65
4	2	1	3	1	1	1	4	3	1	4	3	4	62
3	3	2	3	3	2	3	3	3	2	3	3	3	68
4	4	2	4	2	2	2	4	3	1	4	4	4	68
5	4	4	2	4	4	4	4	2	4	4	4	4	67
4	4	4	4	4	4	4	4	4	4	4	4	4	68

4	2	2	4	1	2	2	4	2	2	4	4	4	57
5	2	1	4	3	1	3	4	1	1	5	5	4	71
3	2	2	2	3	3	2	4	2	2	3	3	3	68
4	3	1	3	2	3	2	4	1	1	3	3	3	55
5	2	2	3	3	2	2	4	2	2	4	4	4	57
2	4	1	4	3	1	1	4	2	1	3	2	2	66
4	1	1	3	1	1	1	3	1	1	5	5	4	65
4	4	4	4	4	4	4	4	4	4	4	4	4	62
3	4	2	2	3	2	2	4	4	2	4	3	3	69
5	1	1	1	1	1	1	5	1	1	5	5	5	55
5	1	1	4	1	1	1	4	1	1	4	5	5	50
4	4	2	2	2	1	1	3	2	1	3	3	4	69
1	5	1	1	4	4	1	1	5	4	2	1	1	61
5	3	1	4	3	1	1	2	4	1	4	4	3	61
4	1	1	3	3	2	1	5	3	1	4	3	4	71
4	2	2	4	3	2	1	4	1	1	4	4	4	58
5	1	1	3	1	1	1	5	1	1	5	5	5	63
3	3	3	3	3	3	3	3	3	3	3	3	3	57
4	3	1	4	2	1	1	4	2	5	4	5	5	62
3	5	1	3	4	4	1	3	4	1	4	4	4	68
4	4	2	4	4	3	3	4	4	3	3	4	3	66
3	2	2	4	2	4	1	3	3	2	4	3	3	71
4	2	2	4	2	1	1	4	2	3	4	4	4	73
3	3	3	3	3	3	3	3	3	3	3	3	3	57
4	4	4	4	4	4	4	4	4	4	4	4	4	76
4	4	3	4	4	3	3	3	4	3	4	3	3	61
5	1	1	4	1	1	1	5	1	1	5	5	5	55
4	5	2	4	3	4	2	4	5	1	4	4	5	65
5	1	1	5	1	1	1	4	1	1	5	5	5	64
3	5	5	4	4	4	2	2	4	2	4	2	2	66
4	2	1	4	3	2	1	4	2	1	4	3	3	52
5	3	1	5	2	1	1	5	2	1	5	5	5	59
5	3	1	5	1	1	1	1	1	1	5	5	5	55
5	1	1	4	1	1	1	5	1	1	5	5	5	60
3	1	1	4	2	1	1	5	3	1	4	4	4	60
3	3	2	3	3	3	2	3	3	2	4	4	4	59
4	1	1	3	5	3	1	5	3	1	4	4	4	50
3	2	1	4	2	1	1	4	1	1	4	3	4	52
3	3	2	3	3	2	2	2	3	2	3	3	3	52
4	2	2	3	2	2	2	4	2	2	4	4	4	59
3	3	3	3	3	3	3	3	3	3	3	3	3	57
5	1	1	5	1	1	1	5	1	1	5	5	5	63
3	5	3	3	4	2	1	3	4	3	4	4	4	64
4	2	1	3	4	3	1	4	1	1	4	4	3	57
3	3	2	2	2	3	3	3	3	2	3	3	3	64
4	1	1	4	4	1	1	4	1	1	4	4	4	63
4	3	2	3	2	2	2	4	3	2	3	3	3	56
3	3	2	3	2	2	2	3	2	1	3	3	3	72
4	3	1	2	1	1	1	4	1	1	3	4	4	51
4	2	2	4	2	2	2	4	2	2	4	4	4	60
5	1	1	4	2	2	2	4	2	2	4	4	4	61
5	1	1	4	1	1	1	5	1	1	5	5	5	60
4	2	2	4	2	2	2	4	2	2	4	4	4	61
3	4	2	4	4	3	2	4	3	1	4	3	3	70
4	3	1	3	2	3	1	4	2	2	4	4	3	49
4	1	1	3	1	1	1	4	1	1	4	4	4	60

3	3	2	4	2	2	2	3	2	1	4	3	3	64
3	3	3	4	4	3	2	3	3	3	3	3	3	62
5	1	1	5	1	1	1	5	1	1	5	5	5	58
2	3	2	4	3	4	2	2	3	2	2	2	2	66
5	2	1	3	3	1	1	5	2	1	5	5	5	64
5	1	1	5	1	1	1	5	1	1	5	5	5	71
4	3	1	3	2	1	1	4	2	1	4	4	3	58
5	3	1	4	2	2	1	5	2	1	5	5	4	73
4	2	2	4	3	1	1	4	1	1	4	4	4	64
4	2	2	2	2	2	2	4	2	2	4	4	4	57
4	2	1	4	1	1	1	4	1	1	4	4	3	68
4	2	1	4	1	1	1	4	1	1	4	4	4	61
4	3	1	3	2	1	1	4	1	1	3	3	4	58
4	2	1	4	1	1	1	3	1	1	4	4	4	54
3	2	1	4	1	2	1	3	1	1	3	2	2	59
5	2	1	3	1	1	1	2	1	1	4	4	4	58
5	5	1	3	1	1	1	3	1	1	5	5	5	51
3	3	1	3	3	1	1	3	3	1	3	3	4	57
4	3	1	4	3	2	2	3	2	2	4	3	4	63
3	3	1	2	1	1	1	4	1	1	5	5	4	53
5	2	1	4	1	1	1	5	1	1	5	4	4	56
4	3	1	3	3	1	1	4	2	1	4	3	3	65
5	2	1	4	2	2	1	4	1	1	5	5	4	65
4	2	2	3	2	2	2	4	2	2	4	4	4	55
3	4	1	3	1	1	1	4	1	1	4	5	5	60
4	1	1	3	1	1	1	4	1	1	4	4	4	61
5	2	1	4	1	1	1	5	1	1	5	4	4	59
3	4	2	3	2	1	1	3	1	4	3	3	4	68
4	2	2	3	2	2	2	4	2	2	4	4	4	61
4	2	1	3	3	2	1	4	2	1	4	4	4	65
5	2	1	4	2	1	1	4	1	1	4	4	4	61
4	3	1	2	4	2	1	3	1	4	4	3	3	80
4	1	1	2	1	1	1	4	1	1	5	5	5	61
3	2	2	3	2	2	1	4	2	2	4	4	4	60
4	1	1	4	1	1	1	4	2	1	4	4	3	58
4	1	1	5	1	1	1	5	1	1	1	4	5	70
3	3	1	3	1	1	1	3	3	1	3	2	3	56
4	1	1	5	1	1	1	5	1	1	5	5	5	56
4	3	1	4	1	2	1	4	3	3	3	3	3	58
4	3	1	4	1	1	1	4	1	1	4	4	4	55
4	2	2	3	2	2	1	3	2	1	4	4	4	73
4	1	1	1	3	1	1	4	1	1	4	4	4	58
5	1	1	5	1	1	1	5	1	1	5	5	5	55
3	3	2	4	3	2	2	3	3	2	4	3	3	58
4	4	1	4	4	3	1	3	4	1	3	3	3	68
4	3	3	4	1	1	1	4	1	1	2	3	3	59
3	2	1	4	3	3	1	4	2	1	4	4	4	64
2	4	2	3	3	4	1	4	3	1	3	3	3	62
2	3	2	3	3	3	2	3	3	2	3	2	2	60
2	4	2	3	2	2	2	5	2	1	3	3	2	60
3	4	1	4	3	2	1	4	3	2	4	4	4	69
4	3	2	3	3	2	2	4	2	2	4	5	4	58
3	1	1	4	1	1	1	1	1	1	4	3	3	62
4	3	1	4	2	1	1	5	1	1	4	4	4	58
4	3	2	3	2	2	2	4	2	2	4	4	4	60
3	2	1	3	2	2	1	3	2	1	3	3	3	54

5	2	1	5	1	2	1	5	5	5	5	5	5	77
4	3	1	3	1	1	1	4	3	1	4	4	3	60
5	4	1	4	1	1	1	5	3	1	5	5	5	60
5	2	1	4	1	1	1	3	3	1	5	5	4	64
5	1	1	5	1	1	1	5	1	1	5	5	5	53
4	2	2	4	2	2	2	4	2	2	4	4	4	66
4	4	4	2	4	2	4	4	4	4	4	4	4	60
2	5	2	3	4	3	2	4	4	3	2	2	2	62
3	3	2	3	4	2	2	2	3	2	2	2	2	58
5	1	1	5	1	1	1	5	1	1	5	5	5	61
4	4	2	4	2	2	1	3	3	2	4	3	3	58
4	1	1	4	1	1	1	4	1	1	4	4	4	55
2	5	2	2	4	1	2	3	5	3	2	2	2	70
4	1	1	5	1	1	1	1	1	1	4	3	3	66
4	4	5	4	5	5	4	4	5	4	4	4	4	82
5	5	5	5	5	5	5	5	5	5	5	5	5	95
5	4	1	5	1	3	1	4	5	1	4	4	4	64
3	3	3	3	3	3	3	3	3	3	3	3	3	57
3	3	3	3	3	3	3	3	3	3	3	3	3	66
4	2	1	3	1	1	1	4	1	1	1	4	4	57
4	3	1	4	3	1	1	4	2	2	4	3	4	58
3	2	5	3	3	2	4	5	2	2	4	2	4	72
3	3	1	3	1	2	1	4	1	2	4	4	3	59
4	3	1	4	1	1	1	5	4	1	5	4	4	52
4	2	1	3	2	2	1	4	1	1	4	4	4	58
5	1	1	5	1	1	1	5	1	1	5	5	5	57
3	4	2	3	2	2	2	4	2	4	4	4	4	60
3	3	3	3	3	3	3	4	2	2	4	4	4	70
4	4	1	4	1	1	1	4	3	1	4	4	4	54
4	2	1	4	1	1	1	4	2	1	4	4	4	55
4	2	2	4	1	1	1	4	1	1	4	4	4	59
4	3	2	3	2	2	2	4	2	2	4	4	4	64
5	1	1	3	1	1	1	5	1	1	5	5	5	57
3	3	3	3	3	2	3	3	3	3	3	3	3	57
4	1	1	2	1	1	1	5	1	1	5	5	4	59
4	1	3	4	1	1	1	1	1	1	4	4	4	59
5	1	1	5	1	1	1	5	1	1	5	5	5	55
3	3	1	3	2	1	1	4	1	1	4	4	3	59
5	2	1	4	1	1	1	5	1	1	4	4	4	54
5	2	1	4	1	1	1	5	1	1	4	4	4	54
3	2	1	3	1	1	1	4	1	1	4	3	3	55
5	1	1	5	1	1	1	5	1	1	5	5	5	59
3	3	1	3	2	3	1	3	1	1	4	4	2	50
4	4	1	1	1	1	1	5	2	4	5	4	5	60
4	3	1	4	3	4	1	3	4	2	4	4	4	63
5	3	1	3	1	1	1	4	1	1	5	4	4	61
5	5	5	5	5	5	1	5	1	1	5	5	5	85
5	2	1	5	1	1	1	5	1	1	5	4	5	69
5	4	2	4	3	3	1	3	3	3	3	3	3	63
4	2	1	4	3	2	1	4	2	1	5	4	4	56
3	3	1	5	5	3	1	3	2	1	3	3	3	66
4	4	1	1	1	5	3	4	3	1	4	4	4	47
3	4	2	4	2	2	1	4	3	1	3	3	3	58
3	2	1	4	1	2	1	4	1	1	4	4	4	61
3	3	3	3	3	3	3	3	3	3	3	3	3	57
4	2	1	5	1	1	1	4	2	1	4	4	4	63

5	2	1	5	1	1	1	5	1	1	5	5	5	83
4	2	1	3	1	1	1	4	1	1	4	4	4	61
2	4	1	3	1	1	2	5	1	1	5	5	4	74
4	1	1	4	1	1	1	4	1	1	4	4	4	46
4	1	1	2	1	2	2	4	1	2	5	4	4	67
3	2	2	3	2	2	2	3	2	1	4	3	3	63
5	2	2	4	2	2	2	4	2	2	4	4	4	58
3	3	3	3	3	3	3	3	3	3	3	3	3	56
3	3	1	4	2	2	1	4	2	1	4	3	3	60
3	3	3	3	3	3	3	3	3	3	3	3	3	57
3	3	2	4	3	2	1	3	2	2	4	3	3	57
5	3	1	5	1	1	1	5	1	1	5	5	5	55
5	5	5	5	5	5	5	5	5	5	5	5	5	84
4	4	4	4	4	4	4	4	4	4	4	4	4	76
5	1	1	5	1	1	1	4	1	1	5	5	5	51
4	2	1	4	2	2	1	4	1	1	4	4	4	53
5	1	1	5	1	1	1	4	1	1	5	5	5	67
4	1	1	5	1	1	1	4	1	1	5	5	4	58
3	3	1	3	2	2	1	3	3	1	4	3	3	51
5	1	1	5	3	1	1	5	1	1	5	5	5	57
4	4	1	4	3	2	1	5	2	2	4	4	4	71
4	4	2	4	2	2	2	4	2	2	4	4	3	58
4	3	2	3	2	2	1	4	2	2	4	4	4	56
4	3	2	3	3	2	1	4	4	2	4	3	3	63
5	1	1	5	1	1	1	5	1	1	5	5	5	62
4	3	1	5	4	1	1	4	2	1	4	4	4	62
4	2	1	3	1	1	1	4	1	1	4	4	4	57
4	4	1	3	3	5	1	4	1	1	5	4	5	62
5	1	1	1	1	1	1	3	1	1	5	5	5	53
4	2	2	3	2	2	2	3	2	2	4	3	3	57
4	4	2	4	3	2	2	3	3	2	4	4	4	57
3	3	2	1	3	3	1	2	3	2	2	3	2	62
4	2	1	4	2	1	1	4	2	1	4	4	4	62
4	1	1	4	1	1	1	4	1	1	4	4	4	50
3	2	1	3	2	2	2	3	3	2	3	3	3	49
5	2	1	5	2	4	1	5	4	3	3	4	5	72
4	3	3	3	3	3	2	4	3	2	4	3	4	69
4	3	2	4	2	2	2	4	2	2	4	3	4	67
3	3	2	3	2	3	1	3	2	2	3	3	3	63
4	3	1	3	3	2	1	3	3	2	4	4	3	80
4	4	1	4	3	3	2	4	3	2	4	4	3	74
5	2	1	5	1	2	1	3	4	1	4	3	4	65
5	3	1	4	1	2	1	4	3	1	5	5	5	60
4	3	2	2	2	2	1	4	2	1	4	4	3	65
5	1	1	5	1	1	1	5	1	1	5	5	5	59
5	1	1	4	1	1	1	4	1	1	5	4	4	61
3	1	1	3	1	1	1	5	1	1	4	4	4	62
3	2	1	2	3	2	1	2	2	1	2	2	2	55
4	1	1	4	1	1	1	5	3	1	5	4	5	55
4	3	2	4	2	2	2	3	2	3	4	4	4	62
4	2	1	3	1	1	1	4	1	1	4	4	3	61
4	2	1	4	1	2	1	4	1	1	4	3	3	56
5	5	5	5	5	5	5	5	5	5	5	5	5	95
2	3	2	4	4	3	2	4	4	2	3	3	2	62
5	1	1	5	1	1	1	4	1	1	5	5	5	59
3	3	3	3	3	3	3	3	3	3	3	3	3	57

3	3	1	3	3	3	1	3	3	1	3	3	3	60
5	1	1	4	1	1	1	4	2	1	5	5	4	64
5	1	1	5	1	1	1	5	1	1	5	5	5	59
4	3	1	5	3	1	1	3	2	1	4	4	4	57
4	4	4	4	2	3	2	3	2	2	3	3	3	58
3	2	1	3	1	1	1	4	1	1	4	4	3	51
3	3	3	3	3	3	3	4	2	3	3	3	3	61
4	2	1	4	1	3	1	4	1	1	4	4	4	59
5	1	1	3	1	1	1	5	1	1	5	5	5	58
4	1	1	4	1	1	1	4	1	1	4	4	4	55
4	2	1	4	1	2	1	4	2	1	4	5	5	61
4	2	1	3	1	1	1	4	1	1	4	4	4	58
2	3	2	2	2	3	2	2	3	1	2	2	2	60
4	3	1	3	3	3	1	4	4	1	4	3	4	59
3	3	2	3	3	3	2	3	3	3	3	3	3	51
3	2	2	2	2	2	2	4	2	4	3	3	3	50
4	3	1	4	3	3	1	3	4	2	4	3	3	56
5	1	1	5	1	1	1	5	1	1	5	5	5	67
4	4	1	4	4	2	1	4	3	1	4	4	4	63
4	2	2	4	2	2	2	4	2	2	4	4	4	52
3	5	1	5	5	1	1	5	5	1	4	4	4	82
4	4	1	2	2	2	2	4	4	2	3	3	3	60
3	5	1	4	4	1	1	4	3	1	5	3	3	91
5	1	1	5	1	1	1	5	1	1	5	5	5	52
3	3	2	3	2	2	1	3	3	2	4	3	3	64
4	3	1	3	1	2	1	4	2	1	4	3	3	61
5	3	3	1	3	2	2	4	2	2	5	5	4	52
4	2	3	3	3	2	3	4	2	3	4	3	3	70
2	3	3	3	3	3	3	3	3	3	3	3	3	63
2	2	2	3	5	2	1	2	4	3	3	3	3	81
3	3	3	3	3	3	3	3	3	3	3	3	3	57
4	4	2	4	4	4	1	2	2	2	4	4	4	70
5	3	2	4	1	1	1	5	2	1	5	5	5	67
4	1	1	4	3	1	1	2	2	2	4	4	4	60
3	4	1	4	2	3	1	4	3	1	3	2	2	57
3	1	1	5	1	1	1	4	2	1	5	5	5	56
3	4	2	3	2	2	1	3	2	1	3	2	2	68
5	5	2	5	2	3	2	5	2	5	5	5	5	95
4	2	1	4	1	2	1	4	3	1	4	4	4	61
5	5	2	5	5	2	1	4	4	1	5	5	5	76
4	2	2	2	2	2	2	4	2	2	4	4	4	56
5	1	1	5	1	1	1	5	1	1	5	5	5	59
5	1	1	5	1	1	1	5	1	1	5	5	5	59
4	2	1	4	2	1	1	4	2	1	4	3	3	69
4	4	4	4	4	4	4	4	4	4	4	4	4	76
4	3	1	2	3	2	1	4	2	2	4	3	3	62
3	3	1	3	1	1	1	5	1	1	5	5	3	53
3	3	2	2	2	2	2	5	2	1	2	2	2	44
5	4	4	5	4	4	3	4	4	4	4	4	5	83
5	2	1	4	1	1	1	5	1	1	5	5	5	61
4	3	2	3	2	1	1	4	2	2	4	3	3	62
5	2	1	5	3	2	1	5	1	1	5	3	3	63
5	1	1	5	1	1	1	5	1	1	5	5	5	59
4	2	1	4	1	2	1	4	1	1	4	4	4	61
5	1	1	5	1	1	1	5	1	1	5	5	4	59
4	5	1	4	2	2	1	4	2	2	5	5	5	64

3	2	1	3	2	2	2	4	2	1	4	4	3	64
4	1	1	3	1	1	1	3	1	1	5	5	5	60
4	1	1	4	1	1	1	5	1	1	4	4	4	57
4	2	2	3	2	2	2	2	2	2	2	4	4	62
4	3	2	3	2	2	2	4	2	2	4	3	4	57
3	3	1	3	4	4	1	4	3	1	4	2	3	66
4	2	2	3	2	2	2	4	2	2	4	4	4	56
4	3	1	3	2	2	2	4	2	1	3	3	3	63
4	4	3	3	3	3	3	3	3	3	3	3	3	56
3	3	1	5	1	1	1	5	1	1	5	5	3	53
5	2	1	3	1	1	1	4	1	1	5	5	4	67
4	2	1	4	2	1	1	4	1	1	4	3	4	62
5	1	1	4	2	1	1	5	1	1	5	4	4	55
3	3	3	3	3	3	3	3	3	3	3	3	3	57
5	1	1	3	1	1	1	5	1	1	5	5	5	55
4	4	1	5	2	2	1	4	3	2	5	5	5	66
5	4	1	1	1	1	1	4	1	1	5	5	4	73
4	3	1	2	2	2	1	3	1	1	5	5	5	63
3	3	3	3	3	3	3	3	3	3	3	3	3	57
5	5	1	5	1	1	1	1	1	1	5	5	5	71
4	3	1	5	3	2	1	5	1	2	3	5	4	54
4	2	2	2	2	3	2	4	2	2	3	3	3	56
4	3	1	5	4	1	1	5	2	1	4	4	4	62
4	4	1	3	3	2	2	4	2	3	4	3	3	64
2	4	4	2	3	3	3	3	3	3	3	3	3	62
3	3	2	2	2	2	2	3	2	2	4	3	3	50
3	4	1	2	1	2	1	3	2	1	4	3	2	61
3	3	3	3	3	3	3	3	3	3	3	3	3	57
3	3	1	3	1	3	1	4	1	1	4	4	3	63
4	2	2	3	3	2	2	4	2	2	3	4	3	60
4	2	1	4	2	2	2	4	2	2	4	4	4	61
2	3	2	3	3	2	1	3	2	1	3	3	2	50
4	4	1	5	3	1	1	4	1	1	5	5	5	66
4	3	2	5	2	1	1	4	2	1	4	4	4	66
4	3	2	3	1	2	2	3	3	2	4	4	4	54
3	3	1	3	2	2	1	3	3	3	3	3	3	65
4	1	1	3	1	1	1	4	1	1	4	4	4	65
4	2	2	3	2	2	2	4	2	2	4	4	4	58
3	4	2	3	3	3	3	3	3	3	3	3	3	69
4	2	2	4	1	1	1	4	1	1	4	4	4	59
3	3	1	4	3	3	1	5	2	1	4	3	3	75
4	2	1	2	1	1	1	4	1	1	4	4	4	65
5	1	1	5	1	1	1	4	1	1	5	5	5	59
3	2	1	4	3	2	1	4	2	1	4	4	4	61
4	2	1	3	2	2	1	4	1	1	4	3	3	55
3	3	1	2	3	1	1	4	4	1	4	3	3	53
5	2	1	4	2	1	1	5	1	1	5	5	5	61
5	1	1	5	1	1	1	5	1	1	5	5	5	59
3	2	2	2	1	1	1	3	1	1	3	3	3	57
5	5	1	5	2	1	1	5	1	1	5	5	5	61
4	3	1	3	3	2	1	3	3	1	3	3	3	59
4	2	1	2	2	1	1	1	1	1	4	4	4	63
4	2	2	2	2	2	1	4	1	3	4	4	4	66
4	1	1	3	1	1	1	3	1	1	4	3	3	46
4	1	1	4	1	1	1	4	4	1	4	4	4	54
2	4	3	2	4	3	3	4	4	2	3	3	3	62

4	3	2	4	4	3	2	3	2	2	4	4	4	60
3	4	1	2	3	2	2	4	2	1	4	5	3	70
3	3	3	3	3	3	3	3	3	3	3	3	3	57
3	3	2	2	2	2	2	3	2	2	3	3	3	50
4	2	1	4	1	1	1	3	1	1	4	5	4	63
4	1	1	1	1	1	1	4	1	1	4	4	4	50
4	1	2	1	2	2	1	4	1	1	4	4	4	65
3	3	3	3	3	3	3	3	3	3	3	3	3	57
3	2	2	4	1	3	1	3	1	3	3	3	3	67
5	1	1	4	1	1	1	4	1	1	4	4	3	58
4	2	1	3	1	1	1	4	1	1	4	4	4	59
5	3	1	2	2	1	1	4	3	1	4	4	4	59
5	2	2	5	3	3	1	5	2	2	5	5	5	84
4	1	2	4	2	1	1	4	1	1	4	4	4	61
5	4	2	4	3	3	2	4	4	1	4	4	3	67
3	3	1	3	1	3	1	3	1	1	3	3	3	54
3	2	1	2	3	4	2	4	2	1	3	3	3	60
4	3	2	2	2	3	2	4	4	1	3	3	3	57
4	3	1	4	2	1	1	4	2	1	4	4	4	57
5	5	5	5	5	5	5	5	5	5	5	5	5	95
3	4	4	1	4	4	1	3	4	3	3	3	2	61
3	2	3	2	3	2	4	3	3	3	4	2	4	57
5	5	1	5	1	1	1	5	1	1	5	5	5	64
4	3	2	3	2	1	1	4	2	1	4	3	3	60
5	1	1	5	1	1	1	5	1	1	5	5	5	55
4	3	1	3	2	2	1	4	2	2	4	4	4	59
4	2	1	4	1	1	1	4	1	1	4	4	4	60
4	4	4	4	4	4	4	4	4	4	4	4	4	76
4	2	2	3	2	2	2	4	2	2	5	5	5	61
4	3	1	5	2	3	1	4	3	1	4	4	4	61
3	2	1	3	3	2	1	4	4	1	4	4	4	67
3	3	3	3	3	3	3	3	3	3	3	3	3	57
3	2	2	3	2	2	1	4	2	2	4	3	3	56
5	2	1	4	1	1	1	3	1	1	5	4	3	60
5	1	1	5	1	1	1	5	1	1	5	5	5	59
4	2	1	2	1	2	1	3	2	1	4	4	3	58
4	2	1	3	2	2	1	4	2	1	4	4	4	57
5	1	1	5	1	1	1	5	1	1	5	5	5	63
4	2	1	2	4	2	1	2	2	1	4	3	4	57
4	3	2	3	1	1	1	5	2	1	4	4	4	57
4	3	3	4	2	2	2	4	2	2	4	4	4	70
5	3	1	5	2	2	1	5	2	1	5	5	5	63
5	1	1	4	1	1	1	4	1	1	5	5	5	53
3	3	2	2	3	2	2	3	3	2	4	3	3	58
4	1	1	3	1	2	2	4	2	2	4	4	4	58
3	3	2	2	3	2	2	2	3	2	3	3	3	57
5	1	1	5	1	1	1	5	1	1	5	5	5	57
4	2	1	5	3	1	1	5	1	1	5	5	5	61
3	4	1	4	3	2	2	4	1	1	3	4	4	61
5	1	5	4	1	1	1	5	1	1	5	5	4	61
5	2	1	4	1	1	1	5	1	1	5	5	4	71
5	2	1	3	1	1	1	5	1	1	5	5	5	60
4	3	1	5	1	1	1	5	1	1	5	3	3	60
3	1	1	3	1	1	1	3	1	1	4	3	3	64
3	4	1	2	1	1	1	4	3	1	4	3	3	56
4	2	1	5	1	1	1	5	2	3	5	5	5	71

3	2	2	3	2	3	2	3	2	3	2	3	3	69
5	2	1	5	1	1	1	5	1	1	5	5	5	76
5	1	1	5	1	1	1	5	1	1	5	5	5	61
5	2	1	5	1	1	1	5	1	1	5	4	4	59
3	4	4	4	3	4	3	4	4	4	4	4	3	69
3	3	3	3	3	3	3	3	3	3	3	3	3	57
4	5	4	5	4	5	1	4	4	2	4	4	4	63
5	1	1	5	1	1	1	5	1	1	5	5	5	89
3	3	3	3	3	3	3	3	3	3	3	3	3	57
4	4	2	2	2	2	2	3	4	3	4	4	4	65
4	3	1	1	1	1	1	4	3	2	4	3	3	57
1	1	1	5	1	1	1	3	3	1	5	3	3	65
4	2	2	2	2	4	4	4	4	3	4	4	4	61
4	2	2	4	2	2	2	4	2	2	4	4	4	57
4	3	1	4	2	2	1	3	2	1	4	3	3	54
4	2	1	4	3	2	1	4	2	1	4	4	4	54
4	2	1	3	1	2	1	5	2	1	4	3	4	53
3	3	2	4	3	3	3	4	3	2	4	4	3	63
5	2	2	3	3	3	3	3	3	3	3	3	3	59
3	2	2	3	4	3	2	4	3	3	3	3	3	65
4	2	1	4	1	1	1	3	1	1	4	4	3	55
3	1	1	3	1	1	1	3	1	1	3	3	3	56
4	1	1	4	1	4	1	3	2	1	4	4	4	54
5	2	1	4	1	1	1	4	1	1	5	5	4	62
5	1	1	3	1	1	1	5	1	1	5	5	4	58
4	4	2	3	3	4	2	4	2	2	4	4	3	63
3	3	2	3	3	3	3	3	3	3	3	3	3	57
4	4	2	1	3	2	2	4	2	2	4	4	3	56
4	3	2	3	4	3	2	4	3	1	4	4	4	56
5	3	1	5	2	1	1	5	1	1	5	5	5	83
3	3	2	4	3	2	1	3	3	1	3	3	3	52
5	2	1	4	1	1	1	5	2	1	5	5	5	63
5	1	1	5	1	1	1	5	1	1	5	5	5	61
3	3	3	3	3	3	3	3	3	3	3	3	3	57
5	1	1	5	1	1	1	5	1	1	5	5	5	69
4	3	2	4	2	2	2	4	2	2	4	4	4	69
5	1	1	5	1	1	1	5	1	1	5	5	5	59
3	4	1	3	2	2	1	4	2	2	3	2	2	64
4	4	1	4	3	2	1	4	3	1	4	4	4	58
3	3	3	4	3	2	2	4	2	1	3	2	3	45
4	2	2	3	2	2	2	3	2	2	4	3	3	58
5	1	1	5	1	1	1	5	1	1	5	5	5	60
3	2	1	4	2	1	1	2	3	1	4	4	4	58
4	2	1	4	1	1	1	2	2	1	5	5	4	55
5	1	1	4	1	1	1	5	1	1	5	5	5	60
4	3	2	3	1	1	1	3	1	1	3	3	3	52
4	3	2	3	3	3	3	3	3	4	3	3	3	65
3	5	1	5	4	2	1	3	3	2	3	3	4	59
4	2	1	4	2	1	1	4	2	1	4	3	3	62
5	1	1	3	1	1	1	5	1	1	5	5	5	57
4	3	1	5	3	2	1	4	3	1	4	5	4	60
4	2	2	3	2	2	2	3	2	2	4	3	3	63
5	2	1	2	2	1	1	3	1	1	5	5	5	64
3	3	3	3	3	3	3	3	3	3	3	3	3	59
3	4	1	5	3	1	1	2	1	1	5	5	5	87
4	3	1	2	2	2	2	4	2	1	4	4	4	56

5	1	1	5	1	1	1	5	1	1	5	5	5	60
4	1	1	4	1	1	1	4	1	1	4	4	4	60
3	4	1	3	3	4	1	3	3	1	4	3	3	64
5	1	1	5	1	1	1	5	1	1	5	5	5	59
5	1	1	3	2	1	1	5	1	1	5	5	5	67
5	1	1	3	1	1	1	5	1	1	5	3	3	61
3	2	3	3	3	3	2	3	3	2	3	3	3	53
4	3	3	2	4	3	3	2	4	2	3	4	2	58
4	3	1	5	3	1	1	3	3	3	5	5	5	64
5	1	1	5	1	5	1	5	5	5	5	5	5	75
5	2	1	3	1	1	1	4	1	1	5	5	5	56
3	4	2	4	3	3	2	4	3	1	4	4	4	63
4	2	1	5	1	1	1	4	1	1	5	4	4	55
3	3	3	4	3	3	2	4	3	2	4	4	4	69
3	3	3	3	3	3	3	3	3	3	3	3	3	57
4	2	1	2	1	1	1	4	1	1	4	4	1	58
4	3	1	5	2	2	1	4	1	1	5	4	4	57
2	3	3	3	3	4	2	3	2	1	3	2	2	61
5	1	1	5	1	1	1	5	1	3	5	5	5	69
5	2	1	4	1	1	1	5	1	1	5	5	4	60
5	5	4	5	5	1	1	5	1	3	5	5	5	58
5	1	1	5	1	5	1	5	5	1	5	5	5	93
3	3	3	3	2	2	1	3	2	1	4	3	3	62
3	3	1	1	2	2	2	3	3	1	4	3	3	49
3	3	2	3	3	2	1	4	2	2	4	3	3	65
3	3	1	5	1	1	1	4	1	1	5	4	4	59
4	2	1	5	1	1	1	5	1	1	4	4	4	58
1	4	2	1	2	4	1	3	4	2	4	2	2	63
3	2	2	3	1	1	1	4	1	1	4	4	4	54
3	2	1	2	2	3	1	4	4	2	4	4	4	64
5	2	1	5	1	1	1	5	1	1	5	5	5	54
4	4	4	4	4	4	2	2	4	2	4	4	4	74
5	1	1	3	1	1	1	3	1	1	5	5	5	65
4	3	2	3	4	2	1	3	2	2	3	3	3	58
4	3	1	3	2	2	1	2	2	1	4	4	4	59
4	4	4	3	3	3	3	3	3	3	3	3	3	62
3	3	2	3	3	2	1	3	3	2	3	2	3	56
2	2	4	2	2	2	4	4	2	4	2	2	2	67
5	1	1	4	1	1	1	4	1	1	4	4	4	63
5	5	1	5	5	5	5	5	5	5	5	5	5	95
4	3	1	3	2	2	1	4	2	2	4	4	3	51
3	3	1	3	3	2	1	4	2	1	4	3	3	63
3	3	3	3	3	3	3	3	3	3	3	3	3	57
5	5	2	5	4	4	1	4	2	3	4	3	3	67
3	3	3	3	3	3	3	3	3	3	3	3	3	57
3	2	2	3	3	2	2	3	3	2	4	3	3	59
4	3	4	3	3	2	1	4	3	3	4	4	4	69
3	3	3	3	3	3	3	3	3	3	3	3	3	50
3	3	2	4	5	2	2	3	4	2	4	3	3	68
5	1	1	1	1	1	1	5	1	1	5	5	5	63
3	3	2	2	3	2	1	3	3	1	3	3	3	59
4	2	2	2	2	2	1	4	2	1	4	4	4	56
5	2	1	3	1	1	1	5	2	1	5	5	5	57
5	1	1	4	1	1	1	5	1	1	5	5	5	59
5	2	1	4	1	1	1	4	3	1	5	5	3	69
5	1	1	5	1	1	1	5	1	1	5	5	5	61

4	3	2	5	2	2	1	4	1	1	4	4	4	59
5	2	1	3	2	2	2	5	1	1	5	5	5	55
5	3	3	4	2	1	1	5	1	1	5	5	5	64
3	3	1	5	2	3	1	3	5	1	4	3	4	59
3	1	1	3	1	3	1	4	1	1	4	4	3	59
3	3	1	3	1	1	1	3	2	1	3	3	3	63
3	2	1	1	1	1	1	4	1	1	5	5	4	64
3	5	2	1	2	4	2	1	3	1	2	2	2	67
4	1	1	4	2	1	1	5	3	1	4	4	4	72
3	3	3	2	3	5	2	2	3	1	3	3	3	64
3	2	2	2	2	2	2	2	2	2	4	4	3	61
5	2	1	5	1	2	2	5	2	1	5	5	5	61
3	2	1	3	1	1	1	5	1	1	5	3	4	67
5	3	1	4	2	2	1	5	1	1	4	4	5	60
4	1	1	4	1	1	1	4	1	1	4	4	4	58
4	3	1	4	1	1	1	5	2	1	5	4	4	55
5	3	1	5	3	3	1	5	2	1	5	5	5	71
3	2	2	2	2	2	2	3	2	2	3	3	2	56
5	5	5	5	5	5	5	5	5	5	5	5	5	95
4	2	1	4	1	1	1	4	1	1	4	4	4	57
3	2	2	2	2	5	2	5	4	3	2	2	4	76
4	2	2	4	2	2	2	4	2	2	4	4	4	57
5	2	1	5	1	1	1	5	1	1	5	5	5	67
4	3	1	3	1	1	1	4	1	1	4	4	3	56
4	2	1	3	1	1	1	3	1	1	4	4	4	49
5	5	1	5	4	1	1	5	1	1	5	5	5	56
5	5	5	5	5	5	5	5	5	5	5	5	5	95
5	3	3	5	3	2	1	5	1	1	5	5	5	71
4	3	2	3	4	3	1	4	4	3	4	4	4	73
4	3	1	3	3	2	2	4	2	1	4	4	4	65
3	4	1	3	2	3	1	4	2	2	4	4	4	66
4	2	2	3	2	2	1	4	1	1	5	5	3	63
4	3	2	4	2	2	2	4	3	1	4	3	4	57
2	3	2	3	3	3	1	4	3	1	4	3	3	61
5	1	1	4	1	1	1	4	1	1	5	5	4	55
3	2	2	4	3	3	3	3	3	3	3	3	3	57
4	3	1	3	2	2	1	3	1	1	4	4	3	66
5	2	1	5	1	1	1	5	1	1	5	5	5	57
5	4	2	3	3	2	1	4	4	1	3	3	3	58
4	2	2	3	2	2	2	4	2	2	4	4	4	57
4	4	1	4	1	2	1	5	3	1	5	5	5	52
3	4	2	3	2	3	3	3	3	2	3	2	3	55
4	3	2	3	3	2	2	4	3	2	4	3	4	58
4	3	2	3	3	4	2	4	3	2	3	4	4	62
4	1	1	5	1	1	1	4	1	1	1	1	5	59
2	4	1	4	4	1	1	4	5	1	4	3	3	69
3	3	2	3	3	2	2	2	3	2	4	4	4	67
3	2	1	3	2	2	1	3	2	1	4	3	3	64
5	5	5	5	5	5	5	5	5	5	5	5	5	95
4	4	1	3	3	3	1	2	4	1	4	4	4	60
3	2	1	3	2	3	1	3	3	2	3	3	3	62
4	2	2	3	2	1	1	4	2	1	4	4	4	56
3	3	3	3	3	3	3	3	3	3	3	3	3	57
3	4	3	3	3	3	2	3	3	2	3	3	3	61
5	3	1	4	3	2	1	4	3	1	4	4	4	68
3	2	2	4	3	2	2	4	2	2	4	2	4	61

3	3	3	3	3	3	3	3	3	3	3	3	3	57
3	3	2	3	2	3	2	4	3	1	4	3	3	64
4	4	1	4	3	2	1	3	2	1	4	3	3	63
5	3	1	3	1	1	1	5	1	1	5	5	5	57
4	3	1	3	2	2	2	4	4	2	4	5	5	61
5	3	1	3	1	1	1	3	2	1	4	4	3	56
4	3	1	5	1	1	1	4	2	1	5	4	4	67
3	3	1	3	3	2	1	5	1	3	5	3	3	83
4	2	1	4	3	2	1	4	2	1	4	4	4	62
5	1	1	4	1	1	1	4	1	1	4	4	4	52
4	2	2	2	1	1	1	5	1	1	5	3	4	68
5	1	1	5	1	1	1	5	1	1	5	5	5	59
4	4	4	4	4	4	4	4	4	4	4	4	4	76
3	1	1	3	1	2	1	4	1	1	3	3	3	64
5	1	1	5	1	1	1	5	1	1	5	5	5	63
3	3	1	3	3	2	1	4	3	2	3	3	3	56
4	4	3	3	3	2	1	4	4	1	5	4	4	69
4	2	1	4	2	1	1	4	1	1	4	4	4	61
4	4	4	4	4	4	4	4	4	2	4	4	4	73
3	3	2	3	2	3	2	4	3	2	4	4	4	61
5	4	2	5	2	5	2	5	2	2	5	5	5	78
3	2	2	4	2	1	1	4	3	1	5	4	2	63
5	1	1	4	4	1	1	5	3	1	5	5	5	63
4	2	1	3	2	1	1	4	2	1	5	4	3	61
4	2	1	2	2	2	1	4	2	1	4	4	4	58
2	2	2	3	3	2	2	2	2	2	2	2	2	56
5	5	5	5	5	5	5	5	5	5	5	5	5	95
3	3	1	4	1	2	1	4	2	1	3	3	3	69
5	5	1	5	2	2	2	3	2	1	5	5	5	62
4	3	2	2	4	2	4	4	4	4	4	4	2	51
4	4	4	4	3	3	2	4	2	3	3	3	4	71
3	2	2	3	3	3	2	4	3	3	3	3	3	55
4	1	1	4	1	1	1	4	2	1	4	4	4	59
4	2	1	3	1	1	1	3	3	1	4	4	4	61
5	1	1	5	1	1	1	5	1	1	5	5	5	58
3	2	1	2	1	1	1	4	1	1	4	4	4	63
5	4	1	4	4	1	1	4	4	1	4	5	5	72
3	3	2	4	2	3	1	3	3	1	3	3	3	66
5	1	1	5	1	1	1	3	1	1	5	5	5	59
4	3	1	2	3	3	1	4	3	1	4	4	4	63
5	3	1	5	3	1	1	5	3	1	5	5	4	76
4	2	2	4	1	1	1	4	1	1	5	4	4	57
5	3	1	5	3	5	5	5	5	1	5	5	5	81
4	4	4	4	4	4	4	4	4	4	4	4	4	76
4	3	1	4	2	1	1	4	2	1	4	4	4	61
4	3	1	2	2	2	1	5	2	2	4	4	4	70
4	3	1	3	2	1	1	4	2	1	4	4	4	61
3	3	2	2	4	4	3	2	3	3	3	2	2	60
5	3	2	5	2	2	2	4	2	1	4	3	4	61
4	4	4	4	4	4	4	4	4	4	4	4	4	62
2	2	3	3	3	3	3	3	3	3	3	3	3	55
3	3	3	3	3	3	3	3	3	3	4	4	4	58
5	1	1	3	1	1	1	3	1	1	3	3	3	56
3	3	2	3	2	2	2	3	2	2	3	3	3	60
5	1	1	5	1	1	1	5	1	1	5	5	5	59
2	2	1	3	2	4	1	4	1	1	4	3	4	59

5	1	1	4	1	1	1	4	2	1	4	4	4	61
4	3	2	3	3	1	1	4	2	1	3	3	4	59
3	2	1	2	2	1	1	2	2	2	2	2	2	54
5	3	5	5	2	2	2	5	2	1	5	5	5	63
4	2	1	3	1	1	1	4	2	1	4	4	4	59
4	2	2	4	2	2	2	4	2	2	4	4	4	66
4	2	1	3	2	2	1	4	2	1	4	4	4	63
4	4	1	3	3	3	1	4	3	1	4	3	3	62
5	1	1	4	1	2	1	5	1	2	5	5	5	75
4	1	1	4	1	1	1	3	2	3	3	2	3	65
4	1	1	2	1	1	1	4	1	1	4	4	4	52
4	2	2	4	2	4	2	4	2	2	4	4	4	56
3	3	2	3	2	1	1	3	1	2	4	3	4	56
5	1	1	3	1	1	1	5	1	3	5	5	5	59
4	5	4	5	4	5	1	1	2	1	5	5	5	88
5	3	1	3	2	1	1	5	1	1	4	3	3	70
3	2	1	2	1	1	1	4	1	1	4	4	4	54
3	2	1	4	3	2	1	4	1	1	4	4	4	58
4	4	1	3	3	2	2	4	2	2	4	3	4	68
4	2	2	4	2	2	2	4	2	2	4	4	4	58
5	5	5	5	5	5	5	5	5	5	5	5	5	95
5	1	1	5	1	1	1	5	1	1	5	5	5	55
5	5	5	5	5	5	5	5	5	5	5	5	5	95
5	3	1	4	1	1	1	5	1	2	5	4	5	68
3	3	3	3	3	3	3	3	3	3	3	3	3	57
5	1	1	5	1	1	1	5	1	1	5	5	5	57
3	1	1	3	1	1	1	4	1	1	3	3	3	57
3	3	2	3	2	2	2	3	2	1	4	3	3	54
5	1	1	5	1	1	1	5	1	1	5	5	5	57
3	4	1	2	2	3	1	4	3	2	4	3	3	55
4	1	1	4	1	1	1	4	1	1	5	4	5	59
3	4	2	2	3	3	2	3	2	3	3	3	3	65
4	2	1	4	2	2	1	3	2	2	4	4	4	60
2	3	3	4	3	3	2	3	3	2	3	3	3	62
2	4	2	4	4	3	1	2	4	2	2	1	1	66
3	2	1	2	2	2	2	4	2	1	3	3	3	61
3	3	2	3	3	3	3	3	3	3	3	3	2	69
5	1	1	5	1	1	1	5	1	1	5	5	5	55
3	3	1	3	2	1	1	4	2	2	4	4	4	52
4	4	4	4	4	4	4	4	4	4	4	4	4	74
5	1	1	4	1	1	1	5	5	1	5	5	5	59
4	2	4	3	2	2	2	4	2	3	4	4	4	63
4	2	2	4	2	2	1	5	1	1	4	4	4	53
4	1	1	4	1	1	1	4	1	1	4	4	4	58
2	4	2	2	4	5	4	1	4	2	2	2	2	55
3	2	2	3	3	2	2	3	3	2	4	4	4	62
5	1	1	3	1	1	1	5	1	1	5	5	5	55
3	3	1	2	4	2	1	4	3	1	3	3	3	63
5	1	1	5	1	1	1	5	1	1	5	5	5	63
1	1	1	1	1	1	1	5	1	1	5	5	5	59
3	3	1	4	2	3	1	2	3	1	3	3	3	58
3	3	1	3	2	2	1	4	2	1	3	3	3	55
3	3	3	3	3	3	3	3	3	3	3	3	3	58
3	3	2	3	2	1	1	4	1	1	4	4	4	53
3	4	3	2	4	2	2	4	4	2	4	3	3	61
3	2	2	3	3	2	2	3	2	2	3	3	3	57

5	5	5	5	5	5	5	5	5	5	5	5	5	93
5	1	1	5	1	1	1	5	1	1	5	5	5	61
3	4	1	4	2	1	1	4	1	1	5	4	4	76
5	3	1	5	3	1	1	3	2	1	5	5	5	69
4	2	2	2	2	2	2	3	2	2	4	3	3	64
4	3	2	3	2	2	2	3	3	1	4	4	4	54
3	4	2	3	2	1	1	3	4	1	4	4	4	59
3	3	2	2	3	4	3	3	3	2	4	4	3	67
3	3	3	3	3	3	3	3	3	3	3	3	3	57
4	4	1	5	3	1	1	2	4	1	4	4	2	54
5	2	1	4	1	2	1	5	1	1	5	4	4	52
3	3	2	3	2	2	2	3	2	1	4	4	4	69
3	2	1	4	3	3	1	3	3	1	3	3	3	62
3	2	1	4	2	3	1	3	2	1	3	3	3	59
4	1	1	4	1	1	1	4	1	2	5	3	4	60
4	3	3	3	3	3	3	2	3	3	3	3	3	59
3	3	1	2	3	3	2	3	2	1	3	3	3	67
5	3	3	5	2	2	2	5	2	2	5	5	5	73
4	3	2	3	3	1	1	4	3	3	5	4	4	57
3	3	1	3	3	1	1	3	1	1	3	3	3	55
4	2	2	4	2	2	2	4	2	2	4	4	4	58
3	3	1	3	1	2	2	2	2	2	3	3	3	57
5	1	1	4	1	1	1	5	1	1	5	5	5	57
5	1	1	5	1	1	1	5	1	1	5	5	5	55
4	4	4	4	4	2	2	4	4	2	4	4	4	69
3	3	2	3	3	3	2	3	3	2	3	3	4	55
3	3	3	3	2	3	3	3	3	3	3	3	3	57
3	3	1	3	1	1	1	4	2	1	4	3	3	66
4	1	1	5	1	1	1	5	1	1	5	5	5	59
4	2	2	3	2	2	2	4	2	2	4	3	3	59
4	1	2	4	2	2	2	4	1	1	4	4	3	68
3	3	1	4	1	2	1	4	1	1	4	3	4	73
3	3	4	4	4	1	1	4	3	3	3	3	3	52
4	2	1	3	1	1	1	3	3	1	4	4	4	57
5	1	1	5	1	1	1	5	1	1	5	5	5	67
3	3	2	3	2	2	1	3	1	1	4	4	4	76
4	3	2	2	3	2	2	3	2	3	3	3	3	58
3	3	2	2	2	2	2	4	1	1	3	3	3	53
5	1	1	5	1	1	1	5	1	1	5	5	5	63
4	3	2	3	3	3	2	3	2	1	4	4	3	65
3	3	3	3	3	3	3	3	3	2	3	3	3	72
3	3	1	3	3	2	1	3	3	1	3	3	3	58
4	4	1	4	1	1	1	5	1	1	4	4	3	59
4	3	1	3	4	1	1	4	1	1	4	4	4	72
5	1	1	5	1	1	1	5	1	1	5	5	5	59
3	4	3	3	3	2	1	3	3	3	3	3	3	69
4	2	1	4	3	1	1	3	1	1	4	4	4	57
3	3	2	3	3	3	3	4	2	2	4	4	4	58
5	4	4	4	4	4	4	4	4	4	5	4	4	79
3	3	1	4	2	1	1	4	3	1	5	5	5	70
5	5	1	2	1	1	1	1	1	1	5	5	3	56
5	5	5	5	1	1	1	5	1	1	5	5	5	87
2	2	2	3	2	2	2	3	3	2	3	4	4	66
5	1	1	1	1	2	1	5	1	2	5	5	2	66
4	2	2	3	2	2	2	4	2	2	4	4	4	54
5	1	1	1	2	3	5	5	5	1	5	2	3	65

5	1	1	4	1	1	1	5	5	5	5	5	5	66
5	1	1	5	1	1	1	5	1	1	5	5	5	67
5	1	1	4	1	2	1	4	1	1	4	4	4	58
3	2	1	3	1	1	1	4	1	1	3	3	3	59
3	3	2	3	3	2	2	3	2	2	3	4	3	59
4	2	2	3	2	2	2	4	2	2	4	4	4	61
4	3	3	3	3	4	3	3	3	2	3	3	3	58
4	3	2	4	3	2	2	3	1	1	3	4	4	67
3	2	2	2	2	2	2	3	2	2	3	3	3	54
4	3	1	4	1	1	1	4	4	1	5	3	4	58
4	1	1	4	2	1	1	4	1	1	4	5	4	73
3	2	2	3	2	2	2	3	3	2	3	3	3	55
5	1	1	5	1	1	1	5	1	1	5	5	5	59
5	1	1	3	1	1	1	5	1	1	5	5	5	63
4	3	2	2	2	2	2	3	2	2	4	4	4	56
4	3	2	3	2	2	2	4	3	2	4	4	3	56
5	3	3	3	3	3	3	3	3	3	3	3	3	58
4	3	1	4	1	1	1	4	2	1	4	3	3	57
5	3	1	4	1	1	1	3	1	1	5	5	5	64
5	1	1	4	1	2	1	4	1	1	4	4	3	68
5	1	1	5	1	1	1	5	5	1	5	5	5	59
3	3	1	3	2	2	1	3	3	2	3	3	3	66
3	3	3	3	3	3	3	3	3	3	3	3	3	57
3	3	3	3	3	3	3	3	3	3	3	3	3	57
4	3	2	4	2	2	2	4	2	2	4	4	4	56
3	2	2	3	2	2	2	3	2	2	3	3	3	45
4	3	3	3	3	1	1	5	2	1	5	5	5	66
5	1	1	3	1	1	1	3	1	1	5	5	5	49
5	1	1	4	1	1	1	5	1	1	5	5	5	62
2	4	5	5	2	5	5	2	5	5	2	2	2	64
5	4	1	3	1	1	1	4	1	1	5	5	5	56
5	1	1	3	1	1	1	5	1	1	5	5	5	57
4	1	1	4	1	1	1	5	1	1	5	5	5	52
4	2	2	4	1	1	1	4	1	1	4	4	4	54
5	4	1	4	3	1	1	5	2	1	5	4	5	60
3	2	1	3	1	2	1	4	1	1	3	3	3	51
3	3	3	3	3	3	3	3	3	3	3	3	3	58
2	4	1	4	4	4	3	3	4	2	2	2	2	69
3	3	2	4	2	3	3	3	3	2	4	2	3	58
4	3	4	3	4	3	3	4	3	3	4	3	4	70
3	2	1	3	1	1	1	4	1	1	3	3	3	63
4	3	1	4	2	1	1	4	2	1	4	4	4	62
5	1	1	4	1	1	1	5	1	1	5	5	5	57
4	2	2	4	2	2	2	3	1	1	4	3	3	56
5	1	1	5	1	1	1	5	1	1	5	5	5	59
3	3	2	1	3	2	1	3	2	3	4	4	3	72
4	3	1	3	1	1	1	4	2	1	4	4	4	61
5	5	5	5	5	5	5	5	5	5	5	5	5	95
5	5	5	5	5	3	3	3	3	3	3	3	3	57
4	5	3	1	3	5	3	5	3	2	4	4	3	64
2	3	4	3	4	4	1	1	4	2	2	2	2	78
5	1	1	4	1	1	1	4	1	1	4	4	4	54
4	4	2	3	2	2	2	4	2	2	4	3	3	60
4	4	2	3	3	2	2	3	2	2	4	4	4	62
3	2	2	3	2	2	3	3	2	2	3	3	3	59
4	2	2	4	2	2	2	4	2	2	4	4	4	57

3	3	1	4	1	1	1	3	1	1	4	4	4	64
4	2	1	3	1	1	1	4	1	1	4	4	4	55
3	3	2	3	2	3	1	4	4	1	4	4	4	64
5	5	5	5	5	5	5	5	5	5	5	5	5	63
3	3	2	4	3	3	2	4	2	1	4	3	3	57
5	1	1	5	1	1	1	5	1	1	5	5	5	69
3	3	2	4	4	3	3	4	4	4	4	4	4	77
3	3	2	2	2	2	1	4	2	2	3	2	3	61
5	1	1	5	1	1	1	5	1	1	5	5	5	55
2	3	1	3	2	1	1	5	1	1	2	4	4	59
5	2	1	4	1	1	1	4	1	1	4	3	3	59
4	2	1	4	1	1	1	5	1	1	5	4	4	59
3	1	1	4	2	1	1	3	1	1	4	4	3	72
1	1	1	3	1	1	1	5	5	5	5	5	5	55
5	3	1	5	1	1	1	5	2	1	4	5	5	64
2	3	2	4	4	3	2	4	4	4	4	4	3	67
3	1	1	2	2	3	1	3	3	1	2	3	3	59
3	2	2	3	2	2	2	4	2	2	4	4	3	55
5	3	1	3	1	1	1	4	1	1	5	5	5	67
4	3	2	5	2	1	1	4	1	1	4	4	4	52
4	4	2	3	3	2	2	4	3	2	4	4	3	60
5	1	1	3	1	1	1	4	1	1	4	4	4	58
5	1	1	4	1	1	1	5	1	1	4	5	4	58
3	2	2	2	2	3	1	2	2	2	3	3	3	52
3	3	2	2	3	2	2	3	2	1	3	3	3	62
5	1	1	5	1	1	1	5	1	1	5	5	5	56
3	4	5	4	5	4	5	4	5	4	5	4	4	85
4	1	2	3	2	2	1	5	1	1	5	5	5	63
4	2	1	4	2	1	1	5	4	1	5	4	4	50
5	1	1	4	1	1	1	5	2	1	5	4	4	73
5	1	1	3	2	2	1	4	1	1	3	4	3	63
3	2	1	3	1	1	1	3	1	1	4	4	4	57
5	1	1	5	1	1	1	5	1	1	5	5	5	63
4	2	1	3	1	1	1	4	3	1	4	4	4	68
4	3	2	3	2	2	2	3	2	2	3	3	3	62
5	4	2	5	2	3	2	5	2	2	3	3	3	68
3	1	1	3	1	2	1	5	1	2	5	3	5	57
4	3	2	2	3	3	2	4	3	2	4	4	3	60
5	2	2	3	1	3	1	4	3	1	5	5	5	75
5	3	1	4	1	1	1	4	1	1	4	4	4	70
4	3	1	3	3	2	2	3	2	2	4	3	3	64
4	3	3	3	3	3	3	2	2	3	4	3	3	69
3	4	2	2	2	1	2	4	4	2	4	3	3	55
3	3	1	3	3	3	1	3	3	1	3	3	3	74
5	1	1	5	1	1	1	5	1	1	5	5	5	59
3	1	1	3	3	3	2	4	2	1	3	3	3	64
3	4	2	2	3	4	1	3	3	2	4	3	3	67
4	3	2	3	2	2	2	4	2	2	3	3	3	56
4	3	1	3	2	3	1	4	4	1	4	4	4	69

dy1	dy2	dy3	dy4	dy5	dy6	dy7	dy8	dy9	dy10	dy11	dy12	dy13	dy14
1	5	1	5	1	1	5	1	1	1	5	5	1	5
3	3	3	3	5	5	3	3	5	3	4	4	2	4
1	5	1	5	2	3	5	3	3	1	5	5	1	5
4	3	2	3	3	1	2	2	1	3	2	3	3	4
4	4	4	3	4	2	2	4	3	4	4	4	2	4
4	5	5	3	3	4	3	3	3	4	4	4	3	4
1	5	3	3	1	1	3	1	1	1	5	5	1	5
1	5	4	4	2	1	4	4	2	2	4	4	4	4
3	3	3	3	3	3	3	3	3	3	3	3	3	3
4	4	4	4	2	1	5	4	4	4	4	4	4	4
2	4	1	4	3	1	5	1	1	1	4	4	1	4
3	3	2	3	3	2	3	2	4	3	4	4	3	3
3	5	3	3	5	3	4	3	4	4	3	3	3	4
3	4	3	3	4	2	3	3	3	3	3	4	4	3
1	5	1	5	5	1	4	1	1	1	4	4	1	4
2	4	2	3	5	1	3	2	4	4	4	3	4	4
3	5	3	3	5	2	3	3	4	3	4	3	3	4
3	3	3	3	3	3	3	3	2	2	5	4	3	4
1	5	3	4	5	1	3	1	1	2	3	5	1	4
4	5	3	2	1	1	2	4	4	4	4	3	3	3
3	3	2	3	4	2	4	3	3	3	4	3	2	3
1	5	5	5	5	1	5	5	5	5	5	5	5	5
3	4	4	4	4	3	4	3	4	3	4	3	4	3
1	5	2	5	4	1	4	1	1	1	4	5	1	5
3	4	3	3	4	1	3	2	3	3	3	3	2	3
1	5	2	3	4	1	5	2	2	2	5	5	2	5
2	5	1	3	4	1	4	2	1	3	4	5	2	5
4	5	2	2	5	5	3	4	4	4	5	5	3	3
2	4	3	4	4	2	3	2	2	2	5	5	2	4
3	5	1	4	1	2	4	1	3	2	4	5	2	4
2	5	1	5	1	1	4	2	1	1	4	5	2	5
1	5	1	5	3	1	5	1	2	1	5	5	1	5
4	4	4	4	4	2	2	2	2	4	4	4	2	4
3	4	3	1	2	1	2	2	2	2	3	3	3	3
2	5	1	5	5	1	5	1	1	2	5	5	2	5
3	5	2	3	5	1	5	5	5	5	5	5	2	4
2	4	2	3	4	2	4	2	2	2	4	4	2	4
3	3	3	3	3	3	3	3	3	3	3	3	3	3
2	4	3	4	4	1	4	2	4	3	3	4	2	4
3	3	3	3	3	3	3	3	3	3	3	3	3	3
3	4	3	4	4	1	3	3	4	3	4	4	3	5
3	3	3	3	3	3	3	3	3	3	3	3	3	3
2	4	1	3	2	1	4	2	2	2	4	5	2	4
1	1	1	5	5	4	1	1	1	1	1	1	1	5
2	4	2	4	4	1	3	2	3	2	4	4	2	4
4	5	3	4	5	1	4	3	3	3	2	3	1	4
1	5	1	5	5	1	5	1	1	1	5	5	2	5
5	5	5	5	5	5	5	5	5	5	5	5	5	5
3	4	3	4	3	2	4	2	3	2	4	4	2	4
1	5	1	4	5	1	4	1	2	1	5	5	1	4

1	5	1	5	5	1	5	1	1	1	5	5	5	5
4	3	2	2	2	4	3	4	3	4	2	4	3	3
2	4	2	4	1	1	4	1	2	2	4	4	2	4
2	5	3	4	3	1	2	1	3	2	3	4	2	5
1	3	2	4	4	2	4	2	2	3	3	4	2	4
2	3	2	3	4	2	2	2	4	4	4	4	2	3
2	3	2	3	1	1	4	2	2	3	4	4	2	3
4	3	3	4	4	2	4	2	3	3	4	4	3	3
2	4	2	2	3	2	2	2	2	1	2	3	2	2
3	4	3	3	3	2	4	3	3	3	3	4	3	4
2	5	3	2	5	1	2	3	3	4	2	2	3	4
2	5	2	4	5	1	2	1	1	2	4	4	1	5
1	5	2	4	4	1	4	1	1	2	4	5	2	4
4	5	1	5	1	1	5	1	5	1	5	5	3	5
2	4	3	3	5	2	4	2	3	4	3	4	2	3
2	4	1	4	4	1	4	1	1	1	5	5	1	4
3	3	2	4	4	2	3	2	3	4	5	4	2	4
1	4	1	4	3	1	3	2	2	2	4	4	1	4
2	5	2	4	2	1	2	2	2	2	4	3	2	3
3	5	2	3	4	2	3	3	3	2	4	4	3	4
1	5	3	4	3	1	3	3	3	2	4	4	2	4
2	5	1	5	5	1	5	2	2	2	4	4	1	5
1	5	1	5	5	1	5	1	1	1	5	5	1	5
1	5	1	5	5	3	5	1	1	1	5	5	1	5
1	1	1	3	3	1	3	1	1	1	4	4	1	4
2	4	4	3	3	2	3	3	3	4	3	4	4	4
1	5	1	4	4	1	4	1	1	1	5	5	1	5
1	5	1	3	2	1	4	1	1	1	5	5	2	4
1	4	1	4	4	1	4	1	2	2	4	4	1	5
2	4	2	4	4	2	3	3	2	2	4	4	2	3
3	3	3	3	3	2	4	3	4	3	3	4	3	5
3	5	2	4	5	1	4	3	4	4	3	4	2	4
3	4	2	3	2	1	3	1	3	3	4	4	2	4
4	5	2	4	4	2	4	2	2	2	3	4	3	4
1	5	1	4	1	1	3	1	1	1	5	5	1	5
3	3	3	3	3	3	3	3	3	3	3	3	3	3
5	5	4	4	5	1	2	4	5	5	2	5	5	4
2	5	2	4	4	2	3	2	3	2	4	4	2	4
3	3	3	3	4	2	4	4	4	4	4	4	4	4
1	5	1	5	5	1	5	1	1	1	3	5	1	5
4	5	2	3	4	2	3	2	3	4	4	4	3	4
1	5	1	4	1	1	3	1	1	2	3	5	3	3
3	4	2	4	4	2	4	2	2	2	4	4	3	4
1	5	1	4	2	1	5	1	1	1	5	5	1	5
1	5	1	5	1	1	5	1	1	1	5	5	1	5
2	5	1	5	5	2	5	1	2	1	5	5	4	5
1	5	3	3	4	1	3	1	2	1	4	4	3	3
4	3	4	3	3	2	3	4	4	2	4	4	3	4
3	4	4	2	4	2	2	1	4	4	2	2	1	5
2	3	2	3	3	2	3	2	2	2	4	4	2	3
1	4	3	4	3	1	3	1	3	1	4	4	1	4
3	5	2	3	5	2	4	2	1	2	4	4	4	4
3	5	2	4	4	2	4	2	2	3	4	4	2	4
1	5	1	5	5	1	3	1	1	1	5	5	1	5
2	4	2	4	4	1	3	2	2	3	4	5	3	4
1	5	1	5	3	1	5	1	1	1	5	5	1	5

1	5	4	5	5	1	5	1	1	1	5	5	2	5
2	3	3	3	3	2	3	3	3	3	3	3	3	4
3	5	1	4	4	1	1	3	2	4	5	5	3	4
1	5	1	5	2	1	5	1	2	2	5	5	2	5
3	5	3	3	4	1	3	2	2	3	5	4	2	5
1	5	1	4	5	1	3	2	3	3	3	3	3	3
1	4	1	4	4	1	3	2	2	2	4	4	2	4
1	5	1	4	3	1	1	1	2	2	5	5	1	4
3	3	2	2	4	1	4	2	4	2	2	4	3	4
2	4	2	4	4	2	4	2	2	3	4	4	2	4
1	5	1	4	5	1	5	1	1	1	5	5	4	5
2	4	3	3	4	2	4	3	3	3	4	4	3	3
3	5	3	3	4	1	4	3	2	3	3	3	3	3
4	5	2	4	5	3	3	2	3	4	4	5	3	4
3	3	3	3	3	2	3	3	3	3	2	4	3	3
4	2	2	3	3	2	2	2	1	2	4	4	2	4
5	5	5	5	5	5	5	5	5	5	5	5	5	5
3	5	4	4	4	2	3	3	3	3	3	3	3	3
1	5	1	5	5	1	5	1	1	1	5	5	1	5
1	5	2	5	3	1	4	2	1	1	5	5	2	5
2	5	2	3	4	1	3	2	3	2	4	4	1	4
3	4	4	3	2	3	3	3	3	3	2	3	3	4
1	5	1	4	3	1	4	2	3	1	4	4	3	4
2	4	2	4	4	2	3	2	2	3	5	5	2	4
4	2	4	4	5	2	2	2	4	4	2	3	4	3
2	2	2	4	4	1	4	1	1	1	4	4	2	4
2	5	2	5	5	1	5	1	2	2	5	5	2	5
2	4	2	3	4	1	4	2	3	2	4	4	3	4
5	5	5	5	5	1	5	1	1	1	5	5	5	5
1	5	1	3	1	1	1	1	3	5	3	1	2	4
3	3	3	3	3	2	3	3	3	3	3	3	2	3
3	3	2	3	3	2	3	3	2	2	4	4	2	4
2	3	1	5	3	2	3	2	2	1	5	5	2	5
3	4	5	1	1	1	1	1	3	3	4	3	2	3
3	4	3	3	3	1	4	2	2	3	3	3	3	4
2	5	2	4	5	1	4	1	2	2	5	5	2	5
5	5	5	1	5	2	1	1	5	5	4	4	1	5
2	5	1	4	4	1	4	1	1	1	4	5	2	5
3	5	3	5	5	3	5	3	3	3	3	3	3	3
1	5	3	4	1	1	3	1	1	3	5	5	3	5
3	3	3	3	3	1	3	3	3	3	3	3	3	3
2	5	4	4	4	1	5	2	2	2	5	5	2	4
3	3	3	3	3	1	3	3	3	1	4	4	4	4
2	5	1	4	5	1	3	2	2	2	4	5	2	4
1	5	2	5	2	1	5	3	1	1	5	4	1	5
2	4	4	4	4	1	4	4	3	2	4	4	1	4
4	4	1	5	5	2	5	1	1	1	5	5	1	5
2	4	3	4	4	2	3	2	2	4	4	4	2	3
4	5	1	5	1	1	4	1	1	1	5	5	2	5
5	5	5	5	5	5	5	5	5	5	5	5	5	5
1	5	1	5	3	1	5	1	3	2	5	5	3	5
2	5	1	4	3	1	5	1	1	3	5	5	2	4
3	4	2	4	4	2	4	2	3	4	4	4	4	4
4	5	2	4	4	1	4	4	4	1	5	5	2	5
3	3	3	3	4	2	3	4	4	4	4	4	4	4
4	4	3	4	4	4	3	4	3	4	3	4	3	4

2	4	1	4	4	2	4	1	1	1	4	4	2	4
4	5	2	4	4	1	5	3	2	2	5	5	2	5
4	5	4	2	5	2	3	3	4	4	4	4	3	4
2	4	1	4	3	1	4	2	2	1	4	4	3	4
2	5	1	5	3	1	1	1	1	1	5	5	2	5
5	5	3	2	5	1	3	2	4	4	2	3	3	3
4	5	3	4	5	1	3	2	3	4	4	4	2	4
4	4	4	4	4	3	3	3	3	3	3	3	3	3
4	5	5	3	4	2	4	3	4	4	2	3	3	4
1	5	1	5	1	1	5	1	1	1	5	5	1	5
1	5	1	5	3	1	3	1	1	1	4	4	1	4
5	5	3	2	5	2	3	4	5	5	2	3	2	4
5	5	4	1	1	1	1	1	5	5	2	3	4	2
1	5	1	5	4	1	3	1	1	2	4	5	3	5
4	4	4	3	5	1	3	4	4	4	3	4	4	3
4	4	1	4	4	1	4	4	2	3	3	3	3	3
1	5	3	5	5	1	5	1	1	1	5	5	1	5
3	3	3	3	3	3	3	3	3	3	3	3	3	3
3	5	1	4	4	1	4	2	3	4	4	4	1	4
1	5	3	3	5	1	5	3	5	3	3	3	4	3
3	3	3	4	4	3	4	4	3	3	4	3	4	3
4	5	3	4	5	2	2	2	4	4	4	3	5	4
5	5	4	3	5	1	3	4	5	4	2	3	3	3
3	3	3	3	3	3	3	3	3	3	3	3	3	3
4	4	4	4	4	4	4	4	4	4	4	4	4	4
2	5	2	4	4	2	4	2	2	2	4	4	2	4
1	5	1	5	1	1	5	1	1	1	5	5	1	5
4	4	2	4	4	2	4	3	2	3	4	5	2	3
4	5	5	3	5	3	1	5	5	5	1	1	5	1
5	1	4	2	4	2	2	4	5	5	2	2	5	3
1	5	1	4	2	1	5	1	1	1	1	5	2	5
4	5	1	5	1	1	3	1	3	2	5	5	1	5
1	5	1	5	5	1	1	1	1	1	5	5	1	5
1	5	1	5	4	1	5	1	3	1	4	5	1	5
1	2	4	5	4	1	2	1	1	1	5	5	1	5
2	4	2	3	4	1	4	4	3	1	4	4	2	4
1	2	3	3	1	1	3	2	3	3	3	3	3	3
1	5	1	5	2	1	4	1	1	1	4	4	3	4
1	4	2	4	3	1	3	1	1	1	4	4	2	4
2	4	3	4	4	2	4	2	2	2	3	3	3	4
3	3	3	3	3	3	3	3	3	3	3	3	3	3
1	5	1	5	5	1	5	1	2	3	5	5	3	5
1	5	2	4	4	1	4	2	3	3	4	4	2	4
1	4	1	4	5	1	5	1	1	2	1	5	2	5
4	4	4	3	4	2	2	2	4	4	2	4	4	4
3	4	3	3	4	1	4	3	3	3	4	4	2	4
2	5	2	3	3	1	3	2	2	2	5	4	3	3
4	5	4	5	5	1	3	4	5	5	3	3	2	2
2	3	2	4	2	1	4	2	1	1	4	4	1	4
2	5	2	4	4	2	4	2	2	2	4	4	2	4
2	5	1	4	4	1	4	1	2	4	5	5	3	4
3	5	4	5	3	1	4	1	1	1	4	5	1	5
2	4	2	4	4	2	4	2	2	2	4	4	2	4
4	5	3	4	5	1	4	2	4	4	3	4	3	4
3	4	3	4	4	1	4	1	1	1	1	1	1	3
2	5	2	4	5	2	4	2	2	2	4	4	2	4

4	4	3	3	4	2	2	4	4	4	3	3	4	4
3	4	2	3	4	1	4	2	4	4	4	4	2	4
1	5	1	5	5	1	4	1	1	1	1	5	1	5
4	5	4	3	5	3	3	4	4	5	1	1	3	1
2	5	2	5	5	2	5	3	3	3	3	3	3	4
1	5	1	5	5	1	5	5	5	5	5	5	1	5
3	4	4	4	2	1	2	2	3	3	4	4	2	5
1	5	2	4	5	1	4	5	4	2	5	5	5	5
4	5	2	4	2	1	4	2	3	3	4	5	4	4
3	3	3	3	3	3	3	3	3	3	3	3	3	3
4	5	2	5	5	1	3	1	3	4	5	5	1	5
2	5	1	4	4	1	5	1	1	1	5	5	2	5
4	4	2	4	4	1	4	1	1	1	4	4	2	4
1	4	1	4	2	1	4	1	1	1	5	5	2	5
2	5	3	4	5	1	2	1	2	2	4	4	2	4
3	5	1	4	4	1	3	1	3	2	3	3	1	5
1	5	1	1	1	1	5	1	1	1	5	5	1	5
3	3	3	3	3	3	3	3	3	3	3	3	3	3
3	4	2	3	3	2	3	3	4	2	4	5	2	4
1	5	1	4	3	1	3	1	1	1	5	5	2	5
1	5	1	5	3	1	4	1	1	1	5	5	1	5
3	5	3	3	5	3	2	2	4	4	2	4	1	5
4	5	1	5	3	1	3	2	4	3	4	4	2	5
2	5	2	4	4	1	4	1	1	2	4	4	1	4
1	5	3	5	5	1	2	3	1	1	4	4	3	5
3	4	1	4	4	1	3	1	4	3	4	4	2	4
1	5	1	5	5	1	5	1	1	1	5	5	1	5
4	4	4	3	4	4	4	4	4	4	3	2	4	3
3	4	3	4	4	2	4	2	3	3	3	3	3	3
2	5	1	4	3	1	4	2	3	3	4	5	4	4
2	5	1	5	4	1	4	1	1	2	4	5	2	5
5	5	4	3	3	1	3	4	5	5	2	5	5	5
4	5	1	4	5	2	5	1	1	1	5	4	2	4
2	4	4	4	4	2	4	3	2	2	3	3	2	4
1	5	3	4	3	1	5	1	1	1	5	5	1	5
3	5	5	5	5	2	5	2	2	2	4	4	3	3
1	5	2	3	3	2	3	2	2	2	4	4	1	4
1	5	1	5	2	1	5	1	1	1	5	5	1	5
1	5	2	4	5	5	3	2	1	2	5	5	1	4
1	5	1	4	4	1	4	1	1	1	5	5	1	4
4	5	4	4	4	2	4	5	4	4	2	4	3	4
1	5	1	5	1	1	5	1	1	1	5	5	1	5
1	5	1	5	1	1	5	1	1	1	5	5	1	5
2	4	2	4	3	2	3	2	2	3	4	4	4	4
1	4	3	5	3	1	4	4	2	2	4	5	3	5
2	5	2	4	4	1	2	1	1	2	5	5	1	4
4	1	1	4	5	1	3	2	5	5	2	4	3	5
4	4	4	4	4	1	2	3	4	4	1	2	4	2
3	5	4	3	3	2	3	2	3	3	2	3	3	3
5	1	5	3	3	1	3	3	5	5	2	3	3	3
4	4	2	4	5	1	2	3	4	4	2	4	2	4
2	4	2	4	4	2	4	2	2	2	4	4	2	4
1	5	1	4	3	1	5	1	4	1	5	5	1	5
2	5	1	4	5	1	4	1	1	2	5	5	2	5
2	4	2	4	4	2	4	2	2	2	4	4	2	4
1	4	1	3	3	1	3	1	1	2	5	5	1	5

3	5	3	5	5	1	5	3	3	4	3	5	5	5
2	5	2	4	3	1	5	1	1	2	5	5	2	4
1	5	2	4	5	1	4	1	1	1	5	5	1	5
1	5	1	5	5	1	5	2	2	2	5	5	2	4
1	5	1	5	1	1	5	1	1	1	5	5	1	5
2	4	2	4	2	2	4	2	4	4	4	4	4	4
4	5	3	3	3	3	3	3	3	3	3	3	3	3
4	4	4	3	3	2	2	4	4	3	4	4	3	2
3	4	3	3	2	2	3	3	3	3	3	3	3	2
1	5	1	5	3	1	5	1	1	1	5	5	1	5
3	4	2	3	4	2	4	3	3	3	3	3	3	3
1	5	1	4	2	1	4	1	1	1	5	5	2	5
3	3	5	2	5	2	5	2	5	5	2	3	5	2
5	5	5	1	5	1	2	1	5	5	5	2	1	4
4	5	4	5	4	5	5	4	4	4	4	5	4	4
5	5	5	5	5	5	5	5	5	5	5	5	5	5
1	5	1	5	5	1	5	1	1	1	5	5	3	5
3	3	3	3	3	3	3	3	3	3	3	3	3	3
4	4	4	3	4	4	3	3	4	4	3	3	4	3
2	4	1	4	2	1	3	2	2	2	4	5	2	5
2	4	3	4	2	2	4	2	2	2	4	4	2	4
4	4	5	4	4	2	4	4	3	4	4	3	5	4
3	5	2	2	2	3	4	2	2	3	5	5	2	3
1	5	1	1	1	1	5	1	1	1	5	5	1	5
2	5	2	4	5	1	3	1	3	2	4	4	2	4
3	3	3	3	3	3	3	3	3	3	3	3	3	3
2	4	2	3	2	2	4	2	3	2	4	4	2	4
2	4	2	4	4	2	4	4	4	4	4	4	4	4
1	4	1	4	4	1	4	1	1	1	4	4	2	4
1	5	1	3	5	1	4	1	1	2	4	5	1	3
2	4	2	4	3	2	3	2	2	2	4	4	3	4
3	4	3	4	4	2	4	2	2	3	4	4	3	4
1	3	1	5	5	1	3	1	1	3	5	5	1	5
3	3	3	3	3	3	3	3	3	3	3	3	3	3
1	5	1	5	5	1	5	1	1	1	5	5	1	5
3	5	1	4	4	1	4	1	2	2	3	4	4	4
1	5	1	5	1	1	5	1	1	1	5	5	1	5
2	4	1	4	3	1	3	1	4	3	4	4	3	4
3	5	1	4	2	1	4	1	1	1	5	5	1	5
3	5	1	4	2	1	4	1	1	1	5	5	1	5
2	5	1	4	3	1	3	1	1	1	5	4	2	5
1	5	1	5	5	1	5	1	1	1	5	5	1	5
1	4	1	4	2	1	4	1	1	1	4	4	1	4
1	5	1	5	1	1	5	1	1	1	5	5	4	5
3	5	2	3	4	1	4	3	2	2	5	5	2	4
3	5	4	3	4	3	2	2	3	3	4	2	1	4
3	5	2	4	5	1	5	5	5	5	5	5	5	5
2	5	2	5	5	1	5	2	2	2	5	5	2	5
2	5	1	4	5	2	4	2	2	3	5	5	1	4
1	5	1	5	5	1	4	2	1	1	4	5	1	5
4	5	1	3	3	1	3	3	3	3	3	4	3	5
1	4	1	4	1	1	3	1	1	1	4	4	1	4
3	4	2	3	4	1	4	2	3	3	4	4	2	3
3	5	3	4	4	1	4	1	2	1	4	4	4	2
3	3	3	3	3	3	3	3	3	3	3	3	3	3
2	5	1	5	3	1	5	1	1	1	5	5	2	5

5	5	5	5	5	1	5	1	5	1	5	5	5	5
2	5	2	4	4	1	3	2	2	2	4	4	4	4
1	5	5	5	5	1	5	5	4	3	3	3	3	3
1	4	1	4	1	1	4	1	1	1	4	4	1	4
3	5	3	4	4	1	4	1	4	3	3	4	1	5
3	4	3	4	4	1	3	2	2	3	3	4	3	4
3	4	2	4	4	1	4	2	2	2	3	4	2	4
3	5	3	3	3	1	3	2	3	3	3	3	3	3
3	4	3	3	4	3	3	3	3	3	3	3	3	3
3	3	3	3	3	3	3	3	3	3	3	3	3	3
3	4	2	4	4	2	3	2	3	3	3	4	3	3
1	5	1	5	5	1	5	1	1	1	5	5	1	1
5	5	5	5	5	5	5	5	4	4	4	4	4	4
4	4	4	4	4	4	4	4	4	4	4	4	4	4
1	5	1	5	1	1	3	1	1	1	5	5	1	5
2	4	2	3	2	2	4	2	2	2	4	4	2	4
1	5	1	5	5	1	5	1	1	1	5	5	5	5
2	5	2	4	2	1	4	1	2	2	4	5	2	5
2	4	2	3	3	1	4	1	2	2	4	4	2	3
1	5	3	5	5	1	1	1	1	1	5	5	1	5
3	5	2	4	5	1	5	2	2	3	4	5	3	5
2	5	1	4	4	1	4	2	2	2	4	4	2	4
1	5	3	4	2	4	2	1	1	2	4	4	2	4
2	4	3	4	4	3	3	2	4	3	3	4	4	4
1	5	5	5	1	1	5	1	1	1	5	5	1	5
3	5	1	4	4	1	5	1	2	4	4	4	2	4
2	5	2	4	4	1	4	1	1	1	4	5	1	4
3	5	3	3	4	1	5	2	4	4	2	3	2	4
1	5	1	5	5	1	5	1	1	1	1	5	1	5
2	4	2	4	4	2	3	2	2	2	4	4	2	4
3	4	1	4	3	2	3	2	2	3	4	4	2	3
4	3	3	3	3	4	3	3	3	4	2	4	2	3
1	5	3	5	2	1	5	1	1	2	5	5	2	5
1	4	1	4	4	1	4	1	1	1	4	4	1	4
2	3	2	3	3	1	3	2	3	3	2	3	3	3
4	3	4	4	4	1	4	5	4	5	4	4	4	4
4	5	3	4	4	1	4	4	4	3	4	3	2	4
3	4	4	4	4	2	3	3	3	3	4	4	4	4
2	5	3	4	4	1	4	2	2	3	4	4	3	4
2	5	3	5	5	2	4	4	4	3	4	5	4	5
2	5	3	4	5	2	5	3	3	3	4	5	3	5
4	3	2	5	5	4	4	4	2	3	5	4	5	3
1	5	1	5	3	1	5	1	1	1	4	5	1	5
4	5	2	4	4	2	3	2	4	4	4	4	4	4
1	5	1	5	5	1	5	1	1	1	5	5	1	5
1	5	1	5	5	1	5	1	1	1	5	5	2	5
3	5	2	4	4	1	3	1	4	2	2	3	4	4
2	4	3	3	3	1	2	2	4	2	3	4	3	4
1	5	2	5	3	2	5	2	1	1	1	5	1	5
2	4	3	4	4	2	4	2	3	2	4	4	3	4
1	5	2	3	5	1	4	1	3	3	5	5	1	3
1	4	1	3	4	1	4	2	2	2	4	5	1	5
5	5	5	5	5	5	5	5	5	5	5	5	5	5
4	4	2	2	5	2	2	2	4	4	3	4	2	3
1	5	5	5	3	1	5	1	1	1	5	5	3	5
3	3	3	3	3	3	3	3	3	3	3	3	3	3

4	5	3	3	3	1	3	1	3	3	3	3	3	3
3	5	1	5	4	1	5	1	1	2	5	5	3	5
1	5	1	5	5	1	5	1	1	1	5	5	1	5
1	5	1	4	1	2	4	1	1	1	5	5	2	5
3	3	3	3	3	3	3	3	3	3	3	3	3	3
1	4	1	4	4	1	4	1	1	1	4	4	1	4
3	3	3	3	3	1	3	3	3	3	5	5	2	5
2	5	1	4	3	1	5	1	2	2	5	4	2	5
3	5	1	5	3	1	5	1	1	1	5	5	1	5
1	4	1	4	2	1	3	1	2	2	4	5	5	5
2	4	2	4	4	2	4	2	3	2	4	5	2	4
2	4	2	4	4	2	4	2	2	2	4	4	2	4
5	3	4	2	3	3	2	4	3	4	3	3	4	2
2	5	1	3	5	2	5	2	2	2	4	5	1	4
2	3	3	2	3	2	3	2	3	3	2	2	3	3
2	4	2	3	3	2	2	2	2	3	3	3	2	3
2	4	2	4	4	1	4	1	1	1	5	5	1	4
3	5	3	5	5	1	5	1	3	3	3	5	1	5
2	5	1	4	5	2	3	2	2	3	3	4	3	4
2	4	2	4	2	2	2	2	2	2	2	4	2	4
5	1	5	5	5	1	5	5	5	5	3	5	5	3
2	5	2	3	3	1	5	2	3	3	3	3	3	3
3	5	3	5	5	5	5	5	5	5	5	5	5	5
1	5	1	1	2	1	5	1	1	1	5	5	1	5
3	5	3	4	3	1	3	2	3	4	3	4	3	4
3	5	1	4	3	1	3	2	3	2	5	5	1	4
1	5	1	5	2	1	1	1	1	2	1	5	1	5
3	4	4	4	4	4	3	4	4	3	5	5	3	5
3	3	3	3	3	3	3	3	3	3	3	3	3	3
5	5	5	4	5	5	3	5	5	5	3	3	5	3
3	3	3	3	3	3	3	3	3	3	3	3	3	3
3	5	4	4	4	1	4	1	2	5	5	5	5	5
1	5	3	4	5	2	4	3	4	4	1	4	3	5
2	5	1	4	4	1	5	2	1	2	5	5	1	4
1	3	2	5	2	1	3	1	2	2	4	5	2	5
1	5	1	5	4	1	3	1	1	1	5	5	2	4
3	4	4	4	5	1	3	2	4	4	4	4	4	4
5	5	5	5	5	5	5	5	5	5	5	5	5	5
3	4	3	4	3	1	4	2	3	2	4	4	2	4
3	5	2	5	5	1	4	2	4	3	5	5	5	5
3	3	4	4	3	2	3	2	2	2	3	2	3	4
1	5	1	5	5	1	5	1	1	1	5	5	1	5
1	5	1	5	5	1	5	1	1	1	5	5	1	5
3	4	3	4	5	3	4	3	3	3	4	4	2	4
4	4	4	4	4	4	4	4	4	4	4	4	4	4
2	3	2	4	3	2	3	3	4	3	4	4	2	4
3	5	2	5	1	1	1	1	1	1	3	5	1	5
4	5	2	3	2	1	3	2	2	2	2	2	2	2
4	3	4	4	4	4	3	5	5	5	5	5	5	5
3	5	1	5	5	1	3	1	1	3	5	5	1	5
3	4	3	3	5	1	3	2	3	3	3	4	2	4
2	5	2	5	5	2	3	2	2	2	5	5	3	3
1	5	1	5	5	1	5	1	1	1	5	5	1	5
2	5	1	4	5	2	4	2	2	2	4	4	2	4
1	5	1	5	5	1	3	1	1	1	5	5	1	5
2	5	3	5	2	1	4	2	2	2	4	5	2	5

2	5	4	3	4	1	4	4	4	4	4	3	4	5
2	5	1	5	5	1	5	1	1	1	5	5	1	5
1	5	2	4	4	1	4	1	1	1	5	5	1	4
2	4	2	4	4	2	4	2	3	3	4	4	3	4
2	4	2	4	4	2	4	2	2	2	4	4	2	4
4	5	1	2	5	2	2	4	4	5	3	4	2	4
2	4	2	4	3	2	3	2	2	2	3	3	2	4
3	4	2	3	4	2	4	3	3	4	3	4	3	4
3	2	3	3	3	3	3	3	3	3	3	3	3	3
1	5	1	5	1	1	3	1	1	1	5	5	1	5
2	5	1	5	5	1	5	1	5	1	5	5	2	5
2	2	1	3	4	1	4	3	4	3	4	4	4	4
1	5	1	5	1	1	4	1	1	1	5	5	2	5
3	3	3	3	3	3	3	3	3	3	3	3	3	3
1	5	1	5	1	1	5	1	1	1	5	5	1	5
4	5	2	5	4	2	4	1	1	1	4	5	4	4
3	5	4	5	5	1	4	1	4	4	3	5	1	5
3	5	4	4	4	1	4	1	1	4	3	4	4	4
3	3	3	3	3	3	3	3	3	3	3	3	3	3
1	5	1	5	5	1	5	1	1	1	5	5	5	5
2	4	2	4	4	1	3	1	2	1	4	4	2	5
2	4	2	3	3	2	3	2	2	2	4	4	2	4
2	5	3	4	4	1	5	1	2	3	5	5	1	4
3	4	3	4	4	1	3	3	4	3	4	4	2	4
3	3	3	3	4	3	3	4	3	4	3	4	3	3
2	3	3	3	3	2	3	2	2	2	3	3	2	3
2	5	1	4	4	1	2	2	4	2	5	5	2	4
3	3	3	3	3	3	3	3	3	3	3	3	3	3
2	5	3	3	5	3	3	2	1	4	3	4	3	3
3	4	2	3	4	2	3	2	3	2	4	4	3	3
2	4	2	4	4	2	4	3	2	2	4	4	2	4
2	4	1	4	2	1	2	1	1	1	2	4	2	4
3	3	2	5	3	2	5	4	4	3	5	5	1	4
3	5	3	4	4	1	4	1	2	4	3	5	1	5
2	2	2	4	1	1	5	1	1	1	5	5	3	5
4	5	3	3	5	2	2	3	4	4	2	3	4	3
3	5	2	4	2	3	3	3	4	4	4	3	3	4
2	4	2	4	4	1	4	2	2	2	4	4	3	4
4	4	4	4	4	1	4	4	4	4	3	3	4	3
3	3	2	4	4	2	4	2	3	2	3	4	2	4
4	5	5	4	4	2	4	2	5	4	4	4	4	4
2	5	1	5	5	1	5	3	3	3	3	4	2	4
1	5	1	5	5	1	5	1	3	1	4	5	1	5
3	5	1	3	4	1	3	2	3	3	4	4	2	4
2	5	3	4	1	1	3	1	1	2	3	4	2	5
1	5	1	4	2	2	4	1	1	1	4	4	2	4
1	5	1	5	5	1	5	1	2	1	5	5	2	5
1	5	1	5	5	1	5	1	1	1	5	5	1	5
3	5	3	3	3	3	3	3	1	3	3	3	3	3
1	5	1	5	5	1	5	1	1	2	5	5	1	5
2	5	1	3	3	1	4	2	4	1	5	5	3	4
2	4	1	4	5	1	5	1	2	2	4	4	3	4
2	5	3	4	4	3	4	3	4	4	3	3	3	3
1	4	1	4	1	1	4	1	1	1	3	4	1	4
1	5	1	4	2	1	4	2	2	2	4	4	2	4
3	4	3	3	3	1	4	3	2	3	4	4	2	4

4	4	2	4	4	1	4	2	2	3	4	4	2	4
4	3	1	4	4	1	5	1	1	3	5	5	3	5
3	3	3	3	3	3	3	3	3	3	3	3	3	3
3	3	3	3	3	1	3	1	3	3	3	3	2	2
3	5	1	5	4	1	4	1	1	2	5	5	2	5
1	4	1	4	2	1	4	1	1	1	4	4	1	4
2	4	2	4	4	2	4	2	2	2	4	4	2	5
3	3	3	3	3	3	3	3	3	3	3	3	3	3
5	5	4	4	5	1	3	2	4	4	2	2	1	4
1	5	1	5	4	1	4	1	1	2	5	5	1	5
3	4	2	3	4	2	3	2	3	3	3	4	2	4
2	5	2	4	4	1	4	1	3	4	4	4	2	4
2	5	5	5	5	1	5	5	5	5	5	5	3	5
1	5	1	4	4	1	4	4	3	1	5	5	1	5
3	5	3	5	3	1	4	2	3	3	4	4	2	4
1	5	1	4	4	3	4	1	2	1	3	4	2	4
4	4	4	4	4	1	4	3	4	3	3	1	3	3
1	5	2	4	1	1	4	1	1	1	5	5	2	5
1	5	1	5	2	1	5	1	1	2	5	5	1	4
5	5	5	5	5	5	5	5	5	5	5	5	5	5
4	5	1	5	5	1	1	1	3	1	5	5	4	3
4	3	3	3	3	4	3	3	3	3	3	2	3	3
3	5	1	4	5	1	4	1	2	1	5	5	1	5
2	4	2	4	3	2	3	2	3	4	4	4	2	4
1	5	1	5	1	1	5	1	1	1	5	5	1	5
2	4	3	4	3	2	3	2	4	2	3	4	2	4
1	5	2	4	5	2	4	2	3	1	5	5	2	4
4	4	4	4	4	4	4	4	4	4	4	4	4	4
2	4	2	4	4	2	4	2	2	2	4	4	2	4
2	5	4	4	5	1	3	2	2	1	4	4	2	4
2	5	1	4	4	1	4	3	3	3	5	4	4	3
3	3	3	3	3	3	3	3	3	3	3	3	3	3
2	4	2	3	4	2	3	2	2	3	3	3	3	3
2	5	2	4	4	1	2	2	3	4	4	4	2	4
1	5	1	5	5	1	5	1	1	1	5	5	1	5
3	3	2	4	4	2	4	2	2	2	4	4	2	4
2	4	2	3	2	2	4	2	2	4	4	4	2	4
1	5	1	5	5	1	5	1	1	1	5	5	5	5
3	5	2	3	4	1	3	3	3	3	3	3	3	3
1	3	2	4	4	1	4	2	1	1	4	4	2	5
4	4	4	3	4	4	4	3	4	4	3	3	4	4
2	4	4	5	5	1	1	2	2	2	4	4	2	5
1	5	1	5	3	1	5	1	1	1	5	5	1	5
2	4	2	3	4	2	3	2	3	2	4	4	3	4
2	4	3	4	3	2	3	2	2	2	4	4	2	4
3	3	3	3	3	3	3	3	3	3	3	3	3	3
1	5	1	5	3	1	5	1	1	1	5	5	1	5
3	4	3	2	5	2	3	2	2	2	2	4	3	4
3	5	1	4	3	1	2	2	4	4	4	2	2	4
1	5	1	5	5	1	3	1	1	1	5	5	1	5
2	5	4	5	5	2	3	2	2	3	5	4	3	5
1	5	2	5	5	1	5	1	1	1	5	5	1	5
2	5	2	5	3	1	3	1	2	2	4	4	2	5
4	5	3	4	5	1	1	3	3	4	4	5	1	4
1	5	2	4	4	1	4	2	2	2	4	4	2	4
3	5	3	5	5	1	4	2	5	5	4	5	2	5

4	5	4	2	5	2	2	3	5	5	1	1	5	3
3	3	4	4	5	1	5	5	5	5	2	2	4	3
3	5	1	5	5	1	5	1	1	1	5	5	1	5
1	5	1	5	5	1	5	1	1	1	5	5	1	5
4	3	4	4	4	3	4	3	4	3	4	3	4	4
3	3	3	3	3	3	3	3	3	3	3	3	3	3
5	5	5	3	3	3	3	3	3	3	3	3	3	3
3	5	5	5	5	3	5	5	5	5	5	5	3	5
3	3	3	3	3	3	3	3	3	3	3	3	3	3
4	4	2	4	4	2	4	3	4	4	2	4	3	4
3	4	2	3	3	1	5	1	1	1	4	5	2	4
2	5	1	3	3	1	4	1	1	1	5	5	4	5
3	4	3	4	4	2	3	2	3	3	3	4	2	4
2	4	2	4	2	1	4	2	2	2	4	4	2	4
2	3	1	4	3	1	4	1	1	2	4	3	2	4
1	4	1	4	2	1	5	1	1	1	5	5	1	4
1	4	2	4	3	1	4	1	1	1	4	5	1	5
3	5	2	3	5	4	2	2	4	4	2	3	4	4
3	5	3	3	3	3	3	3	3	3	3	3	3	3
4	5	3	4	3	2	3	4	4	4	2	4	3	4
1	4	3	4	3	1	4	1	1	1	4	4	3	4
1	5	2	2	4	2	4	2	3	2	4	4	2	4
1	4	1	4	3	1	4	2	2	2	4	4	2	4
2	5	1	5	4	1	5	1	1	2	5	5	3	5
1	5	1	5	4	1	5	1	1	1	5	5	1	5
3	4	4	3	4	2	4	2	2	2	4	4	3	4
3	3	3	3	3	3	3	3	3	3	3	3	3	3
2	5	1	4	4	1	4	1	1	1	5	5	1	5
2	4	1	4	2	2	4	2	2	2	4	4	2	4
2	5	4	5	5	1	5	5	5	2	4	5	5	5
3	4	1	3	3	1	2	2	3	3	3	3	2	4
1	5	2	5	4	1	5	1	1	4	5	5	1	5
1	5	1	5	5	1	5	1	1	1	5	5	1	5
3	3	3	3	3	3	3	3	3	3	3	3	3	3
1	5	1	5	5	1	5	1	1	3	5	5	1	5
4	5	5	4	4	1	3	4	4	4	2	3	4	3
1	5	1	5	5	1	5	1	1	1	5	5	1	5
4	3	2	3	4	1	3	2	4	4	4	3	2	5
3	5	1	4	4	2	2	2	3	3	4	4	1	3
2	3	2	3	3	1	3	2	1	1	2	2	3	3
3	4	3	3	3	1	4	3	3	3	3	3	3	3
1	5	1	5	5	1	5	1	1	1	5	5	1	5
2	4	2	4	4	2	5	2	2	3	4	4	1	3
1	5	1	5	2	1	4	1	1	1	4	4	1	4
1	5	1	4	5	2	5	1	1	1	5	5	1	5
1	4	1	4	4	1	4	1	2	2	4	4	1	4
4	4	3	3	4	3	3	3	3	4	4	3	3	4
3	1	2	2	5	1	3	3	3	4	4	4	4	3
2	4	2	4	4	2	4	3	2	3	4	4	2	4
1	5	1	5	3	1	5	1	1	1	5	5	1	5
3	5	2	5	1	1	5	3	5	2	4	4	1	4
4	5	2	4	4	2	4	2	3	2	4	4	2	4
2	5	1	5	5	5	5	1	1	1	5	5	1	5
3	5	3	3	3	3	3	3	3	3	3	3	3	3
5	5	5	5	5	1	5	5	5	1	5	5	5	5
2	2	2	4	4	2	4	2	2	2	4	4	2	4

4	5	1	5	1	1	5	1	1	1	5	5	3	5
1	5	3	3	4	1	3	2	2	2	4	4	2	4
3	4	2	3	5	3	3	2	4	3	3	4	3	5
1	5	1	5	5	1	5	1	1	1	5	5	1	5
2	5	1	5	5	1	5	2	3	3	4	5	2	5
1	5	5	5	3	1	5	1	1	1	5	5	1	5
3	3	3	3	2	1	2	3	3	3	3	3	3	3
3	4	2	4	2	4	2	4	2	4	2	4	2	4
1	5	2	5	5	1	5	1	3	2	5	5	1	5
5	5	5	1	5	1	5	1	5	5	1	5	5	5
1	5	1	5	1	1	5	1	1	1	5	5	1	5
3	5	2	5	5	1	3	2	2	5	2	2	3	4
1	5	1	3	1	1	3	2	2	2	4	4	2	5
3	5	2	4	4	1	4	2	4	4	4	4	4	4
3	3	3	3	3	3	3	3	3	3	3	3	3	3
1	5	2	5	2	1	4	1	1	3	4	4	1	4
1	5	1	5	3	1	4	1	1	1	5	5	1	5
2	5	2	3	4	3	3	2	3	4	2	3	4	3
3	5	5	5	1	1	5	1	1	1	5	5	1	5
1	5	1	5	5	1	4	1	1	1	4	4	1	5
1	4	1	5	1	1	5	1	1	1	5	5	5	5
5	5	5	5	5	5	5	5	5	3	5	5	5	5
2	5	2	3	4	2	3	2	2	2	5	5	4	4
1	5	1	3	3	1	3	1	1	1	4	4	1	4
3	4	2	4	3	3	3	3	3	3	4	4	4	4
1	5	1	5	4	1	5	1	1	1	4	5	3	5
1	4	1	4	4	1	5	2	1	1	5	5	1	5
4	5	4	2	2	3	3	4	4	4	2	2	4	2
2	4	1	4	4	1	4	1	1	2	4	4	2	4
3	3	2	4	4	2	4	2	3	4	4	4	2	3
1	5	1	5	5	1	5	1	1	1	5	5	1	5
4	4	4	4	4	2	4	4	4	4	4	4	4	4
1	5	1	5	5	3	5	1	3	1	3	3	3	5
2	4	3	2	3	2	4	3	3	3	4	4	2	3
2	4	2	4	4	2	4	2	2	2	4	4	2	4
4	3	4	3	4	4	3	3	4	3	3	3	3	3
3	3	2	3	3	2	3	2	2	2	3	4	3	4
4	4	4	3	4	1	4	4	4	4	2	2	4	3
2	5	1	5	3	1	5	1	1	3	5	5	3	4
5	5	5	5	5	5	5	5	5	5	5	5	5	5
2	4	1	4	3	1	4	2	1	2	4	4	1	4
3	3	4	3	4	2	3	2	4	4	3	3	3	4
3	3	3	3	3	3	3	3	3	3	3	3	3	3
3	5	2	4	4	1	2	2	2	2	5	5	3	5
3	3	3	3	3	3	3	3	3	3	3	3	3	3
2	4	2	3	4	2	3	3	2	2	4	4	3	4
4	4	3	4	3	2	4	3	4	3	4	5	4	4
3	5	3	3	2	1	2	2	2	2	4	4	2	4
3	4	3	3	4	2	3	4	4	4	3	4	3	3
1	5	1	5	5	1	5	1	1	1	5	5	1	5
2	5	2	3	3	3	4	2	2	3	3	4	3	4
1	5	1	5	1	1	4	1	1	1	5	5	1	5
1	5	1	5	3	2	5	1	1	2	4	5	1	5
1	5	1	5	5	1	5	1	1	1	5	5	1	5
3	5	1	3	5	1	5	3	3	3	4	4	2	5
1	5	1	5	5	1	5	1	1	1	5	5	1	5

3	5	1	4	4	1	4	1	3	2	4	4	2	4
1	5	1	5	1	1	5	1	1	1	5	5	1	5
1	5	3	5	5	1	5	1	3	2	4	5	1	5
4	4	4	4	4	2	2	2	2	3	3	3	3	4
2	5	3	4	2	1	3	3	2	3	3	3	2	4
4	3	4	3	5	2	3	3	3	3	2	2	4	2
3	4	2	4	4	1	4	2	3	2	4	5	2	4
5	5	5	2	5	1	1	3	5	5	2	2	5	2
2	5	5	5	5	1	4	2	3	4	5	5	2	5
3	3	2	4	2	1	5	2	2	2	5	4	5	3
3	5	2	4	3	2	3	3	2	4	4	4	2	3
3	5	1	4	4	1	5	3	2	1	5	5	1	5
1	3	2	4	5	1	5	2	3	3	5	5	4	4
2	5	1	5	5	1	5	1	1	1	5	5	1	5
1	5	2	5	4	1	4	1	1	1	5	5	1	5
2	5	2	4	5	1	4	1	1	2	4	5	1	4
2	5	2	5	5	1	5	2	2	2	5	5	3	5
3	5	2	4	2	2	2	2	3	2	2	2	2	4
5	5	5	5	5	5	5	5	5	5	5	5	5	5
2	4	1	4	4	1	4	1	1	3	4	4	4	4
3	5	3	5	5	4	4	4	4	4	2	3	5	4
2	4	2	4	4	2	4	2	2	2	4	4	2	4
5	5	5	5	5	1	5	1	1	1	5	5	1	5
1	5	1	4	4	1	5	1	1	1	5	5	2	4
2	4	1	3	1	1	2	1	1	2	3	4	2	4
1	5	1	5	5	1	1	1	1	1	5	5	2	5
5	5	5	5	5	5	5	5	5	5	5	5	5	5
1	5	1	5	5	1	5	5	5	5	5	5	1	5
3	5	2	4	5	1	3	4	3	2	5	5	4	5
4	2	4	4	4	1	4	2	4	4	2	3	2	4
2	4	2	4	4	4	4	3	3	3	3	4	3	4
3	5	3	3	5	1	3	1	2	2	4	5	3	5
2	5	3	4	3	1	4	2	2	2	4	4	2	4
3	4	3	3	4	2	3	3	3	3	3	3	3	3
1	5	2	4	4	1	4	1	1	1	4	4	1	4
3	3	3	3	3	3	3	3	3	3	3	3	3	3
2	5	3	3	5	1	5	3	3	3	3	5	1	3
1	5	1	5	1	1	5	1	1	1	5	5	2	5
2	5	3	3	3	3	3	3	3	3	3	3	3	3
3	3	1	4	3	1	4	2	4	2	4	4	2	4
1	5	1	5	1	1	2	1	1	1	5	5	1	5
3	3	3	3	3	2	2	3	4	3	3	3	3	3
2	4	3	4	3	1	3	2	2	2	4	4	3	4
3	4	3	4	3	2	3	2	3	3	3	4	4	4
1	5	1	5	5	1	5	1	1	2	4	4	2	4
1	5	4	4	4	1	3	3	4	4	4	4	2	4
4	4	4	4	4	2	4	2	3	3	4	4	2	4
2	5	2	4	3	1	3	1	4	3	5	5	2	5
5	5	5	5	5	5	5	5	5	5	5	5	5	5
3	3	3	3	5	3	3	3	3	3	3	2	3	2
3	4	3	3	3	2	3	3	3	2	4	4	3	3
2	4	1	2	5	3	4	3	2	2	4	4	2	4
3	3	3	3	3	3	3	3	3	3	3	3	3	3
2	4	3	4	4	3	4	2	2	4	4	4	2	3
3	4	3	4	3	3	4	2	4	2	4	4	4	4
3	3	3	3	3	2	4	3	2	2	4	4	2	3

3	3	3	3	3	3	3	3	3	3	3	3	3	3
2	5	2	3	4	1	3	3	4	2	4	4	4	3
4	5	4	4	4	1	3	2	2	2	3	4	2	4
3	3	3	3	3	3	3	3	3	3	3	3	3	3
3	5	2	4	4	1	4	1	1	1	5	5	1	5
1	5	1	5	1	1	5	1	1	1	5	5	1	5
2	5	1	5	3	1	5	1	2	2	5	5	1	5
3	5	5	5	5	1	5	5	5	5	5	5	3	5
1	5	1	5	5	1	4	1	1	2	5	5	2	5
1	5	1	4	3	1	3	1	1	1	4	5	1	5
2	5	2	5	2	1	5	4	3	3	4	5	3	5
1	5	1	5	5	1	5	1	1	1	5	5	1	5
4	4	4	4	4	4	4	4	4	4	4	4	4	4
3	3	4	4	4	1	4	2	2	4	4	4	3	4
1	5	1	5	5	1	5	5	1	1	5	5	1	5
2	5	3	3	5	1	3	2	1	3	3	4	2	3
4	5	3	5	5	1	5	3	3	3	3	3	3	3
3	5	1	4	5	1	4	1	2	2	4	4	1	4
4	4	4	4	4	3	4	4	4	2	4	4	4	4
2	5	2	4	4	1	4	1	5	2	5	4	2	4
1	5	2	5	5	3	5	2	5	2	5	5	5	5
3	4	4	4	4	1	4	2	2	2	5	5	1	5
2	5	1	5	5	1	5	1	1	1	5	5	4	5
1	5	3	4	5	2	4	2	1	1	5	5	1	4
1	4	2	4	4	2	4	2	2	2	3	3	1	4
3	3	2	3	3	3	3	3	3	3	3	3	3	3
5	5	5	5	5	5	5	5	5	5	5	5	5	5
3	5	1	4	4	1	2	2	3	4	5	5	2	5
1	5	1	5	3	1	5	1	1	1	5	5	4	5
2	4	2	4	2	2	1	2	2	2	4	4	4	4
4	4	4	4	4	2	4	4	4	2	3	4	4	4
2	4	2	4	4	2	3	2	2	2	4	4	2	3
2	3	1	4	5	1	3	1	3	2	4	4	3	5
3	5	1	4	3	1	4	1	3	3	4	4	3	4
1	5	1	5	4	1	5	1	1	1	5	5	1	5
3	5	3	3	2	1	4	3	4	3	3	4	3	4
4	5	2	5	5	1	5	3	1	2	5	5	5	5
3	5	1	5	5	1	3	1	2	1	5	5	3	5
1	5	1	5	5	1	5	1	1	1	5	5	1	5
4	4	1	4	4	1	4	1	3	3	4	5	3	4
3	5	1	5	5	1	5	3	4	3	5	5	4	5
2	4	2	4	4	1	4	1	1	1	5	4	1	4
1	5	5	5	5	1	5	1	5	3	5	5	5	5
4	4	4	4	4	4	4	4	4	4	4	4	4	4
1	5	2	5	5	1	3	1	3	2	4	5	2	5
1	5	4	3	5	1	5	2	5	2	4	5	4	3
3	5	3	4	4	3	4	3	2	2	3	4	2	4
3	4	3	3	3	2	4	3	3	3	3	3	4	3
4	4	2	4	4	3	4	3	3	3	3	3	3	3
4	4	4	4	4	3	3	3	3	3	3	3	3	3
3	3	3	3	3	1	3	3	3	3	3	3	3	3
2	4	2	4	4	2	4	2	2	2	4	4	2	4
1	5	2	3	3	3	3	3	3	3	3	3	3	3
4	4	3	3	3	2	4	3	4	2	3	3	3	3
1	5	1	5	5	1	5	1	1	1	5	5	1	5
1	5	1	4	5	4	4	2	1	1	4	5	2	4

2	5	1	4	4	1	4	3	2	1	5	5	2	4
3	4	3	4	4	1	3	3	3	3	4	3	2	4
2	3	4	3	1	2	3	2	2	2	3	4	3	3
2	5	2	5	3	2	5	3	2	2	5	5	1	5
1	5	2	4	3	2	4	2	2	2	4	4	3	4
2	5	3	5	5	1	5	1	1	1	5	5	3	5
3	5	2	4	5	2	2	2	3	3	3	4	2	4
3	5	2	3	5	2	5	3	3	3	5	4	2	3
3	5	3	5	5	1	5	2	3	5	3	5	2	4
1	5	3	5	5	1	5	1	1	1	1	5	5	4
1	5	1	4	4	1	4	1	1	1	4	5	1	4
2	4	3	3	3	1	3	2	3	2	3	4	2	4
3	4	2	3	2	1	4	2	3	2	4	4	2	3
1	5	1	5	5	1	5	1	1	1	5	5	1	5
5	5	3	5	5	5	5	5	5	5	4	4	5	5
3	4	3	3	5	2	4	3	3	4	5	5	2	4
2	4	3	4	3	1	2	2	3	2	4	4	2	4
2	4	2	4	4	1	4	2	2	2	4	4	3	4
4	5	2	4	5	2	3	3	4	4	3	5	2	4
2	4	3	4	4	2	4	2	3	3	3	3	3	3
5	5	5	5	5	5	5	5	5	5	5	5	5	5
1	5	1	5	1	1	5	1	1	1	5	5	1	5
5	5	5	5	5	5	5	5	5	5	5	5	5	5
4	3	5	5	5	4	1	4	4	5	2	2	3	4
3	3	3	3	3	3	3	3	3	3	3	3	3	3
1	5	1	5	5	1	3	1	1	1	5	5	1	5
2	5	1	4	3	1	5	1	2	1	2	5	2	4
2	5	1	5	3	1	3	2	2	2	3	4	2	4
1	5	1	5	3	1	5	1	1	1	5	5	1	5
3	4	3	3	3	1	2	1	3	2	3	4	2	4
1	5	1	5	5	1	5	1	1	1	5	5	1	5
4	4	4	2	4	2	2	4	4	4	2	4	3	3
3	4	3	3	3	1	3	2	3	3	3	3	3	4
3	4	1	3	4	1	3	2	3	2	5	5	3	3
4	4	4	2	4	2	3	4	4	4	2	2	4	3
2	5	2	4	4	2	4	3	3	4	3	4	2	3
3	4	3	3	4	2	3	4	4	4	3	4	3	3
1	5	1	5	5	1	5	1	1	1	5	5	1	5
1	1	2	4	4	1	4	1	2	1	4	4	2	5
4	4	4	4	4	4	4	4	4	4	4	4	4	4
1	5	1	5	1	1	5	1	1	1	5	5	1	5
2	4	3	4	4	2	4	2	3	2	4	3	4	4
1	5	2	4	4	1	4	1	1	1	1	4	4	4
1	4	1	4	4	1	4	1	4	1	4	4	1	4
2	4	2	4	3	2	4	2	2	4	2	3	4	3
4	4	2	3	4	1	4	2	4	3	4	4	2	3
1	5	1	5	1	1	5	1	1	1	5	5	1	5
3	5	2	5	2	1	5	2	2	1	4	5	2	5
1	5	1	5	5	1	5	1	1	1	5	5	1	5
1	5	1	5	5	1	5	1	1	1	5	5	1	5
2	5	3	3	4	1	4	3	2	3	3	4	2	3
2	4	1	3	5	2	3	1	1	1	5	5	2	4
3	3	3	3	3	3	3	3	3	2	4	4	3	3
1	4	1	4	3	1	4	1	1	1	4	4	2	4
3	4	4	2	5	2	3	2	3	4	2	3	2	4
3	4	2	3	4	2	3	3	2	3	3	3	3	4

5	5	5	5	5	5	5	5	5	5	5	5	3	5
1	5	1	5	5	1	5	1	1	1	5	5	1	5
2	5	4	5	5	2	5	1	4	4	4	5	1	5
1	5	1	5	3	1	5	1	5	1	5	5	3	5
2	4	4	3	3	2	3	3	3	3	4	4	4	4
1	4	1	1	2	3	3	3	3	2	4	4	2	4
2	4	2	4	4	2	4	2	2	2	4	4	3	3
3	5	3	4	4	2	4	3	3	4	3	4	4	4
3	3	3	3	3	3	3	3	3	3	3	3	3	3
1	4	2	4	1	1	4	1	1	2	2	4	4	4
1	5	1	4	3	1	2	1	1	2	4	4	2	4
3	4	2	3	3	3	3	4	4	4	4	4	4	4
3	5	3	3	5	1	3	2	3	4	2	3	3	3
3	5	2	3	5	2	3	3	2	3	4	4	3	3
2	5	1	5	1	1	3	2	3	1	5	4	1	4
3	4	3	3	4	3	3	3	3	3	3	3	2	3
4	5	4	4	5	1	4	1	3	3	4	4	3	4
2	5	5	5	5	1	5	2	2	2	5	5	2	5
2	4	2	4	4	1	3	1	2	2	4	4	3	4
3	3	3	3	3	3	3	3	3	3	3	3	1	3
2	4	2	4	4	2	4	2	2	2	4	4	2	4
3	5	1	4	3	2	3	3	3	3	2	3	3	3
1	5	1	5	5	1	4	1	1	1	5	5	1	5
1	5	5	5	1	1	5	1	1	1	1	5	1	5
5	4	4	2	4	4	2	4	4	5	2	2	4	3
2	3	4	3	4	2	3	2	2	4	2	3	2	4
3	4	3	3	4	2	3	2	3	3	2	3	3	3
4	4	2	4	5	1	4	2	3	5	4	4	2	4
1	5	1	5	3	1	5	1	1	1	5	5	1	5
3	4	3	4	4	2	3	3	3	3	3	3	3	3
2	5	5	4	2	1	5	2	2	2	5	5	2	5
4	5	5	3	5	2	4	4	5	5	5	3	1	3
2	5	2	2	2	2	1	1	1	1	5	5	1	5
3	4	1	4	3	1	3	2	2	4	4	4	2	4
1	5	5	5	5	1	5	1	1	1	5	5	1	5
4	4	3	3	5	3	4	3	4	4	3	5	4	4
2	4	2	4	3	2	3	3	2	4	3	3	3	4
2	4	2	3	2	1	4	1	3	2	3	3	2	4
1	5	1	5	5	1	5	1	1	1	5	5	1	5
3	5	2	5	5	1	4	2	2	3	4	4	2	4
4	5	2	4	4	2	4	3	5	5	5	5	3	3
3	5	2	3	2	1	3	2	2	3	4	4	3	4
2	5	1	4	5	1	3	1	2	2	5	5	1	4
4	5	4	4	5	2	5	4	5	3	2	4	3	4
1	5	1	5	5	1	5	1	1	1	5	5	1	5
3	2	4	4	4	1	4	4	4	4	2	4	4	4
3	5	2	3	3	1	2	2	2	3	4	4	2	4
2	4	2	4	3	2	4	2	2	2	4	4	2	4
4	4	4	5	5	4	5	4	5	3	4	4	4	4
2	5	4	4	4	2	4	4	2	3	4	4	3	4
1	1	1	4	3	1	4	1	1	1	5	5	5	4
5	5	1	5	5	1	5	5	5	5	5	5	5	5
4	5	4	4	4	2	3	2	4	4	3	4	2	4
2	5	1	5	5	1	5	1	1	1	5	5	5	5
2	4	2	4	4	2	4	2	2	2	2	2	2	4
3	5	5	5	1	1	1	1	1	1	5	5	5	5

2	5	3	5	5	1	5	1	3	1	4	4	3	5
5	5	5	5	5	1	5	1	1	1	5	5	1	5
1	5	1	4	4	1	4	2	3	1	5	5	1	4
3	5	3	3	3	3	3	3	3	3	3	3	3	3
3	5	3	3	3	2	4	3	3	4	2	3	3	3
2	4	4	4	3	2	4	3	2	3	3	4	2	4
2	5	2	4	4	1	3	2	2	3	3	4	3	3
3	5	4	4	5	1	4	3	3	3	4	4	3	4
2	3	3	3	3	2	3	3	3	3	3	3	2	3
1	5	1	4	5	1	4	1	1	1	4	5	2	5
3	4	2	5	5	2	5	5	4	3	4	4	3	4
2	4	2	3	3	2	3	2	2	2	4	4	3	3
1	5	1	5	5	1	5	1	1	1	5	5	1	5
1	5	1	5	5	1	5	1	1	1	5	5	1	5
2	4	2	4	2	1	4	2	2	2	5	4	2	4
2	4	2	4	4	2	3	2	3	3	3	3	2	4
4	4	4	3	3	1	2	3	4	4	2	2	4	3
1	4	4	4	4	2	3	1	1	1	5	5	1	5
1	5	5	5	5	1	4	4	1	1	5	5	2	5
3	5	1	4	5	1	5	1	4	2	2	5	4	5
1	5	1	5	5	1	5	1	1	1	5	5	1	5
4	5	5	2	5	3	3	3	4	5	1	1	3	3
3	3	3	3	3	3	3	3	3	3	3	3	3	3
3	3	3	3	3	3	3	3	3	3	3	3	3	3
2	3	2	4	2	3	4	2	2	2	4	3	2	4
3	3	3	3	3	2	2	2	2	2	2	2	2	2
3	5	1	5	5	1	5	1	4	3	3	3	3	3
1	5	3	4	3	1	3	1	1	1	1	3	1	5
1	5	2	5	5	1	5	1	1	1	5	5	1	5
5	4	5	2	5	5	2	5	5	5	1	1	5	1
1	5	1	5	3	1	5	1	1	1	5	5	1	5
1	5	1	5	3	1	5	1	1	1	5	5	1	5
1	5	1	4	4	1	4	1	1	1	4	4	2	4
1	4	1	4	4	1	4	2	2	2	4	4	2	4
1	5	1	5	5	1	4	1	1	1	5	5	1	5
1	5	2	4	2	1	4	1	2	2	4	4	1	4
3	3	3	3	3	3	3	3	3	3	3	3	3	3
4	4	1	2	5	3	2	5	5	5	2	5	3	2
4	3	2	3	3	2	4	2	3	3	4	4	3	2
4	4	3	4	4	3	4	3	4	2	4	4	3	4
3	5	3	3	3	2	3	3	3	3	3	4	2	4
5	5	2	4	4	3	3	3	3	3	3	3	3	3
1	5	1	5	3	1	5	1	1	1	5	5	1	5
1	4	1	4	3	2	3	2	2	2	4	5	3	4
1	5	1	5	5	1	5	1	1	1	5	5	1	5
3	5	3	2	5	2	3	3	5	5	4	4	3	3
3	4	2	4	4	1	4	2	2	2	4	5	2	4
5	5	5	5	5	5	5	5	5	5	5	5	5	5
3	3	3	3	3	3	3	3	3	3	3	3	3	3
4	4	4	3	1	3	3	3	4	3	3	3	3	4
4	5	5	3	5	1	4	5	5	5	2	4	5	2
1	5	1	5	1	1	4	1	1	1	5	5	1	5
2	4	2	4	4	2	4	2	2	2	4	4	2	4
2	4	2	4	4	2	4	2	2	4	4	4	2	4
2	4	3	3	3	2	3	3	3	3	3	3	2	4
2	5	2	4	4	2	4	2	2	2	4	4	2	3

1	5	2	5	5	2	4	2	2	2	5	5	2	4
3	5	2	4	3	1	3	2	2	2	2	4	2	3
3	3	1	4	4	1	4	2	4	4	5	3	4	4
1	5	5	5	5	1	5	1	1	1	5	5	1	5
1	4	1	4	4	1	4	1	1	1	5	5	2	5
3	5	2	5	5	1	5	3	3	3	2	4	4	4
4	5	4	5	4	1	5	3	5	4	2	3	4	4
2	5	2	3	4	1	4	2	2	3	4	4	2	4
1	1	1	5	5	1	5	1	1	1	5	5	1	5
1	5	1	3	4	1	5	1	1	2	5	5	2	5
2	5	3	5	2	1	4	2	2	1	5	5	1	4
1	5	1	5	4	1	5	1	1	1	5	5	1	5
4	4	4	3	5	5	3	4	4	4	3	3	4	4
1	5	1	5	5	1	5	1	1	1	1	5	1	5
2	5	2	4	5	1	5	1	2	1	5	5	2	5
3	4	2	4	4	1	4	4	4	2	4	4	3	4
3	5	3	3	4	1	3	2	2	1	5	5	1	5
2	4	2	4	2	2	2	2	2	2	4	4	2	3
3	5	3	3	3	1	3	1	3	3	5	5	3	5
1	4	2	4	4	1	4	1	1	1	1	4	1	4
2	4	2	4	3	2	4	2	4	4	4	3	2	4
1	5	2	5	3	1	4	1	1	2	5	5	1	5
1	5	1	5	4	1	5	1	1	1	5	5	1	5
1	4	3	4	3	2	2	2	2	2	3	3	2	3
2	5	3	4	5	1	4	1	1	1	4	5	3	5
1	5	1	5	2	1	5	1	1	1	5	5	1	5
4	5	4	5	4	5	4	5	4	5	4	5	4	5
4	4	2	4	3	2	4	2	2	2	4	4	2	5
1	1	1	4	4	1	2	2	1	1	5	5	1	4
3	5	2	4	5	1	5	4	4	4	3	4	2	4
1	5	4	4	4	1	4	2	2	2	5	5	2	5
2	4	2	4	3	1	3	1	3	2	4	4	3	4
1	5	1	5	5	1	5	1	1	1	5	5	1	5
4	5	3	3	4	4	2	2	4	4	3	4	3	3
3	4	2	3	4	3	3	3	3	3	3	3	3	4
3	4	3	4	4	2	4	2	2	3	5	5	2	5
1	5	1	5	1	1	5	1	1	3	5	5	1	5
2	5	1	5	4	3	3	3	3	3	3	3	3	4
5	5	5	5	5	1	5	2	2	1	5	5	2	4
3	5	4	4	5	1	4	1	4	3	5	5	1	4
3	5	2	4	5	2	4	2	3	2	4	4	2	3
3	5	3	3	3	2	4	4	3	4	4	3	4	4
1	5	1	4	3	2	3	2	2	2	4	4	2	4
5	5	5	2	5	1	5	3	3	5	1	5	5	3
1	5	1	5	5	1	5	1	1	1	5	5	1	5
2	5	5	5	1	1	4	2	4	2	4	4	4	4
4	4	3	3	4	2	4	4	4	3	4	2	3	3
2	4	2	4	2	1	4	2	2	2	4	4	2	4
4	4	2	4	4	4	4	2	4	3	4	4	3	3

dy15	dy16	dy17	dy18	dy19	attotal	xjgh	jjgh	xjdh
1	5	5	5	1	308	81	95	39
3	1	1	3	4	271	77	76	52
5	5	3	5	3	243	46	89	24
2	1	5	5	5	203	44	62	36
3	3	4	4	4	277	81	70	55
3	3	2	4	5	268	79	59	69
1	5	5	5	5	243	63	67	51
2	4	4	4	2	267	83	73	51
3	3	3	3	3	264	81	57	69
4	4	5	4	3	320	101	76	69
2	4	4	5	2	238	58	84	34
2	3	3	3	3	264	81	57	69
3	4	4	5	3	258	73	71	58
4	4	4	4	3	274	78	70	67
1	4	4	5	1	265	58	85	46
3	4	3	5	3	247	64	67	52
3	3	3	4	4	278	85	69	62
4	4	5	5	5	295	92	77	55
1	5	5	5	3	260	77	76	39
4	4	3	3	4	277	88	60	74
4	4	4	4	4	303	92	80	63
5	5	5	5	5	352	99	95	67
4	4	4	4	4	319	98	71	80
1	5	4	5	2	247	45	93	25
2	4	4	4	4	240	63	61	55
2	5	5	5	2	264	69	80	39
1	5	5	5	2	245	63	71	45
4	2	3	4	5	284	93	75	56
2	4	3	5	2	230	52	75	31
2	5	4	5	5	241	60	75	43
1	5	4	5	4	257	71	75	40
1	5	5	5	2	287	79	95	35
2	4	4	4	4	262	83	65	56
3	1	2	2	5	304	94	62	81
2	5	5	5	1	281	75	93	27
3	5	3	5	4	303	99	79	61
3	4	4	4	3	263	70	74	47
3	3	3	3	3	260	79	58	64
3	4	3	4	3	263	78	75	43
3	3	3	3	3	264	81	57	69
3	1	1	1	3	270	89	63	66
3	3	3	3	3	265	81	57	69
2	5	5	5	2	279	93	68	68
5	5	3	5	3	301	97	88	45
2	4	4	4	4	281	91	74	52
3	4	5	5	4	238	48	78	38
1	5	5	5	2	290	97	76	60
5	5	5	5	5	395	121	88	101
3	3	2	3	2	217	53	66	34
1	5	5	5	3	252	56	86	29

5	5	5	5	5	265	59	95	23
4	3	4	4	3	275	83	58	76
2	4	4	4	2	258	74	75	45
3	2	4	5	4	266	69	77	45
3	4	4	4	3	233	57	80	29
4	2	4	5	3	271	88	74	54
2	5	4	5	4	279	96	79	52
3	4	4	4	3	300	97	75	64
2	1	2	2	2	220	65	53	47
2	4	4	4	3	266	72	73	52
3	2	3	3	4	258	73	62	65
1	5	5	5	1	247	53	84	28
2	3	4	5	2	246	69	76	33
1	5	5	5	5	303	81	82	65
2	3	3	4	5	268	91	63	57
1	5	5	5	2	271	71	76	49
3	5	4	4	3	268	82	71	55
2	2	3	4	3	241	54	76	36
2	4	4	5	3	244	74	61	57
2	3	2	5	3	243	68	76	36
2	3	3	4	3	262	68	76	48
1	5	4	5	2	264	78	85	36
1	5	5	5	1	245	34	95	23
1	1	1	5	1	260	67	82	41
1	4	3	4	2	224	59	58	48
4	3	3	4	4	261	94	62	62
1	5	4	5	2	224	47	84	25
2	5	3	4	2	195	39	58	41
2	5	5	5	2	271	65	95	33
2	4	3	4	3	225	59	70	43
3	2	3	4	4	288	88	69	66
3	4	4	5	3	249	58	79	37
3	3	4	4	3	286	96	68	66
5	4	4	5	5	298	88	79	66
1	5	4	5	2	244	40	95	23
3	3	3	3	3	264	81	57	69
5	3	3	5	4	310	96	88	60
3	3	4	5	2	248	65	72	46
4	4	4	4	4	260	76	61	61
1	5	5	5	5	236	29	95	23
4	4	4	5	3	276	85	67	65
2	3	3	5	3	220	50	69	37
2	4	4	4	4	288	91	77	49
5	5	5	5	2	296	82	91	47
1	5	5	5	1	244	31	95	23
1	5	5	5	5	283	64	94	36
2	4	3	5	2	232	73	59	42
3	3	4	2	3	294	85	69	75
3	4	3	5	4	285	95	63	72
2	3	3	3	3	230	67	57	51
1	4	4	4	1	230	65	73	27
4	4	4	4	4	269	70	83	39
2	4	4	4	3	290	82	77	61
1	5	5	5	3	270	70	90	35
4	3	4	5	3	274	76	81	46
1	4	4	5	2	252	53	95	25

1	5	5	5	2	241	37	95	28
2	2	2	4	2	243	70	55	66
2	1	1	5	5	271	77	72	63
3	5	5	5	1	259	60	95	24
3	5	4	5	3	267	81	57	65
3	3	3	3	3	259	79	58	65
2	4	4	5	3	227	45	80	24
1	5	4	5	2	265	68	81	42
2	4	3	5	4	285	85	65	66
2	4	3	4	2	252	62	68	49
2	5	5	5	1	277	77	91	40
3	4	4	4	4	259	80	68	56
3	4	3	4	4	266	82	65	60
4	5	4	4	5	288	92	77	61
3	1	4	5	4	317	113	76	77
2	4	3	5	4	242	79	55	57
5	5	5	5	5	440	135	95	115
3	5	3	4	1	263	81	74	58
1	5	5	5	1	252	39	91	27
1	5	5	5	3	330	118	87	65
1	5	4	4	3	293	88	81	58
3	3	4	4	3	273	84	60	66
2	3	3	4	4	248	68	76	40
2	5	4	4	2	284	82	74	57
4	4	5	5	4	262	74	68	59
1	4	4	4	2	259	70	76	48
2	5	5	5	2	234	37	91	23
2	4	4	5	3	294	97	78	52
1	5	5	5	5	320	105	91	79
3	5	4	4	3	257	74	61	53
3	3	3	3	3	257	77	57	67
2	4	4	5	4	287	102	61	75
2	5	5	5	4	237	68	58	48
3	5	5	5	3	267	95	51	74
4	4	4	4	2	251	75	69	47
2	5	5	5	3	275	73	85	43
4	5	5	5	4	221	48	75	31
1	2	5	5	2	288	94	67	64
3	3	3	3	3	264	81	57	69
1	5	3	5	3	266	98	59	64
3	3	3	3	3	267	84	57	72
2	3	5	5	3	261	82	69	55
4	4	4	4	4	261	80	58	67
2	5	5	5	2	263	70	77	45
4	4	4	5	3	235	63	60	52
1	4	4	4	4	228	61	73	37
1	1	5	5	3	295	89	87	46
2	4	3	5	2	238	69	63	47
5	5	5	5	1	297	70	95	50
5	5	5	5	5	400	123	83	107
2	5	5	5	3	264	50	95	25
3	5	5	5	2	290	80	90	43
4	4	4	4	4	270	85	59	70
1	5	5	5	2	263	57	95	27
4	4	4	3	3	264	86	58	69
3	4	3	4	3	293	83	69	73

1	5	4	5	4	250	57	79	38
4	5	5	5	3	274	65	89	39
3	4	4	4	2	289	88	71	59
2	4	3	4	3	261	77	63	61
2	5	5	5	2	351	99	94	65
4	4	3	5	5	285	90	86	41
2	4	4	4	3	269	72	89	39
3	3	3	3	3	264	81	57	69
3	4	4	4	4	285	97	59	74
1	5	5	5	1	299	100	78	51
1	4	4	5	1	278	74	95	33
5	3	2	5	4	267	84	74	46
5	1	5	5	5	260	71	75	61
3	5	4	5	3	302	98	83	49
4	4	4	5	4	242	58	81	39
3	3	3	3	3	264	81	57	69
1	5	5	5	3	270	65	95	27
3	3	3	3	3	264	81	57	69
3	4	4	5	2	263	67	79	41
2	4	5	5	5	337	121	80	80
4	3	4	3	4	290	90	66	73
3	5	5	5	2	300	102	77	54
4	5	5	5	4	271	87	70	52
3	3	3	3	3	268	81	57	73
4	4	4	4	4	287	93	62	75
2	4	4	4	4	306	99	76	69
1	5	5	5	1	281	77	95	24
4	3	4	4	4	302	86	77	61
5	1	1	3	5	270	87	61	65
5	3	4	3	5	286	91	77	59
1	4	5	5	2	245	71	73	42
1	5	5	5	1	327	103	95	40
1	5	5	5	1	247	40	95	23
1	5	5	5	2	249	42	94	24
5	5	5	5	2	265	64	84	42
2	4	3	5	3	264	73	83	42
2	4	4	4	2	245	65	61	50
1	4	3	5	2	251	71	74	47
1	4	4	5	3	257	82	65	51
2	4	4	4	3	251	63	75	46
3	3	3	3	3	264	81	57	69
3	5	1	5	2	276	56	95	36
3	5	5	5	3	323	110	75	78
1	5	5	5	3	272	89	78	53
4	2	4	4	3	274	91	63	58
2	4	4	4	4	296	102	74	54
2	5	3	4	2	237	64	65	48
4	4	4	4	5	269	88	72	55
1	5	4	4	2	236	65	71	34
2	4	4	5	2	262	73	74	47
2	4	4	4	2	294	99	70	65
1	5	4	5	2	267	54	95	25
4	4	4	4	3	266	71	76	49
3	4	4	5	4	291	94	74	60
1	4	4	4	4	241	62	70	49
2	3	4	5	2	253	79	72	43

3	3	4	4	2	293	96	75	57
3	4	3	4	3	273	84	62	68
1	5	5	5	5	238	53	87	23
3	5	3	5	4	283	100	60	74
3	3	3	4	3	322	96	86	73
1	5	5	5	1	255	43	94	23
2	4	3	4	2	268	79	73	52
1	5	5	5	4	288	92	85	42
2	4	3	5	3	268	67	89	34
3	3	3	3	3	235	65	61	53
2	5	5	5	2	281	77	88	42
2	5	5	5	2	277	72	90	31
4	4	4	4	2	232	60	76	26
1	5	5	5	1	255	89	76	31
1	5	5	5	2	249	64	81	43
3	5	5	5	1	225	50	83	25
1	4	4	5	3	255	53	93	29
3	3	3	3	3	263	79	57	70
2	5	4	5	3	269	79	62	66
1	4	4	5	1	240	56	83	36
1	5	4	5	2	263	72	84	31
3	4	4	4	4	300	114	79	50
4	3	4	5	3	283	66	92	44
1	4	4	5	2	246	71	73	39
2	1	4	5	5	300	92	88	49
3	4	4	4	4	285	90	76	53
1	5	5	5	1	274	63	95	30
4	2	2	5	4	269	94	65	59
3	3	4	4	3	262	65	76	46
3	4	5	5	3	261	73	82	41
1	5	5	5	3	317	112	81	59
5	5	5	5	5	291	90	75	63
1	5	4	5	2	230	56	78	23
2	4	3	5	3	256	80	73	45
1	5	5	5	1	259	65	86	34
3	3	4	5	5	302	84	91	40
2	4	4	4	4	272	90	68	54
1	5	5	5	1	242	38	95	23
1	2	4	5	1	264	80	82	39
1	5	5	5	1	233	43	90	25
2	4	5	5	4	288	88	73	57
1	5	5	5	4	250	59	78	45
1	5	5	5	1	280	75	95	27
2	3	4	4	2	263	75	74	55
3	5	5	5	4	302	95	82	56
1	5	4	5	5	304	101	89	47
2	5	5	5	2	291	95	80	58
4	4	3	4	4	275	88	60	68
3	4	4	4	3	236	74	56	59
3	3	3	3	3	303	82	93	45
5	4	5	5	5	316	104	80	67
2	4	4	4	2	262	65	77	49
1	5	5	5	4	262	71	88	26
1	4	4	5	1	292	93	84	41
2	4	4	4	4	279	84	76	48
1	5	5	5	2	244	70	77	38

2	5	5	5	5	292	66	95	40
1	5	5	5	2	252	70	76	43
1	5	5	5	3	273	67	93	41
2	5	5	5	2	270	82	76	51
1	3	5	5	1	243	34	95	25
4	4	4	4	4	266	68	76	46
3	3	3	3	3	264	81	57	69
3	3	3	4	3	272	88	57	79
3	4	4	3	4	293	99	66	76
5	5	5	5	1	271	59	95	26
3	3	3	3	3	273	88	59	66
1	5	5	5	1	247	50	90	24
5	4	4	3	5	316	114	86	66
3	5	5	5	1	255	68	85	36
5	4	4	4	4	379	114	84	100
5	5	5	5	5	440	135	95	115
1	5	5	5	4	324	105	93	50
3	3	3	3	3	264	81	57	69
4	3	2	3	4	267	81	59	70
4	4	3	5	2	260	78	77	40
2	4	4	4	3	279	86	80	46
4	3	3	4	4	267	80	66	60
2	4	3	4	3	258	75	71	50
1	5	5	5	2	315	112	90	52
2	4	4	4	2	254	64	78	42
3	3	3	3	3	268	67	69	62
4	4	4	4	4	274	83	72	58
4	4	4	4	4	302	104	68	71
1	4	4	5	4	256	71	76	46
1	5	5	5	2	246	49	91	32
3	4	4	4	3	205	34	71	29
3	4	4	4	3	262	74	73	51
1	5	5	5	1	232	27	93	27
3	3	3	3	3	281	87	63	71
1	5	5	5	1	299	91	91	44
3	4	4	5	1	258	69	91	29
1	5	5	5	1	250	54	95	23
3	3	4	5	3	244	66	73	46
1	4	4	5	1	230	47	82	32
1	4	4	5	1	230	47	82	32
2	4	4	5	2	224	49	78	31
1	5	5	5	1	262	47	95	25
1	4	4	4	4	276	91	70	58
3	5	5	5	1	264	44	95	34
2	5	4	4	3	295	86	85	54
4	2	3	5	4	236	45	89	30
5	5	5	5	5	440	135	95	115
2	5	5	5	4	248	53	94	25
3	4	3	5	3	281	87	76	55
1	4	4	5	1	229	50	82	26
3	5	5	5	4	287	91	70	67
3	4	4	4	1	190	42	31	76
2	4	3	4	3	252	71	65	56
4	4	3	5	3	255	63	80	34
3	3	3	3	3	264	81	57	69
1	5	5	5	5	265	75	92	28

5	5	5	5	5	352	87	95	75
2	4	4	5	3	262	77	76	44
4	5	5	5	4	285	84	65	74
1	4	4	4	1	242	69	76	23
4	5	5	5	3	273	71	76	54
4	4	4	4	4	280	94	63	66
2	4	4	5	2	265	71	76	48
3	3	3	3	3	260	82	57	65
3	3	3	4	3	246	65	75	42
3	3	3	3	3	264	81	57	69
3	3	3	3	2	265	81	76	47
1	5	5	5	1	308	84	95	34
4	4	4	4	4	304	91	74	69
4	4	4	4	4	352	108	76	92
1	1	5	5	3	241	44	90	23
2	2	3	5	2	258	66	78	44
5	5	5	5	1	253	41	95	23
2	5	4	5	1	270	65	95	34
3	3	3	3	2	244	76	63	49
1	5	5	5	1	275	51	94	37
3	5	5	5	4	327	106	91	50
2	4	4	5	2	274	82	76	48
2	5	3	5	2	259	65	75	48
3	5	2	4	2	282	82	80	57
1	5	5	5	4	248	47	91	37
2	5	4	5	2	254	61	78	41
1	5	5	5	2	250	64	79	42
3	3	4	5	2	292	84	83	55
1	5	3	3	3	240	53	83	27
2	4	4	4	2	253	72	68	48
3	4	2	4	4	270	69	68	63
4	3	2	4	5	274	95	62	68
1	5	5	5	3	251	67	75	41
1	4	4	4	2	244	64	76	32
2	3	3	3	2	221	65	57	46
1	4	4	5	4	312	101	81	46
3	4	4	5	4	323	101	91	62
4	4	3	4	3	286	87	75	56
3	4	3	5	3	256	74	70	50
5	5	5	5	5	262	74	80	43
3	5	4	5	5	312	97	85	60
1	1	4	4	2	332	102	81	81
1	5	5	5	5	316	104	84	64
2	4	2	4	3	274	81	72	57
1	5	5	5	1	317	94	89	63
1	5	5	5	2	280	72	94	27
4	5	4	4	3	249	61	80	29
2	2	4	5	2	262	88	61	63
1	5	4	5	1	237	38	92	23
2	4	4	4	3	270	68	75	60
1	5	5	5	3	267	88	79	31
1	5	4	5	2	237	51	78	38
5	5	5	5	5	440	135	95	115
4	4	3	4	4	269	85	71	58
1	1	5	5	1	285	57	95	45
3	3	3	3	3	264	81	57	69

3	4	4	5	3	261	78	59	69
1	5	4	5	3	307	94	93	43
1	5	5	5	1	248	35	95	23
1	5	5	5	3	277	86	94	30
3	3	3	4	3	267	82	61	66
3	3	4	5	1	263	75	79	38
2	3	3	5	3	271	77	73	51
2	4	4	5	2	264	81	72	44
1	5	3	5	2	240	37	93	23
1	4	4	4	2	262	77	61	61
2	4	4	5	2	285	88	77	45
2	4	4	4	2	251	59	76	48
3	4	2	2	4	259	90	57	65
1	4	4	5	2	311	97	90	50
3	3	2	3	4	250	79	52	67
2	2	3	4	3	262	80	57	68
1	4	4	4	4	283	89	81	51
3	5	5	5	1	274	63	95	33
4	4	4	4	4	282	93	76	45
2	4	4	4	2	290	90	76	50
5	5	4	5	5	305	91	87	57
3	4	4	5	3	247	76	61	52
5	5	5	5	5	298	96	63	75
1	5	5	5	1	267	61	95	25
3	4	4	5	3	271	87	72	54
2	5	5	5	2	259	80	74	40
2	5	5	5	3	239	52	84	37
4	3	3	3	2	267	82	66	59
3	3	5	5	5	297	103	67	74
5	4	4	2	5	353	126	88	86
3	3	3	3	3	258	78	54	69
1	1	5	5	5	296	86	93	34
3	3	3	5	5	259	53	93	30
2	5	4	5	2	284	86	90	41
1	5	5	5	3	261	90	67	52
3	3	3	4	4	268	75	72	53
3	4	3	5	3	280	94	81	49
5	5	5	5	5	440	135	95	115
2	4	4	5	3	262	69	78	43
2	5	5	5	5	273	69	79	55
3	4	3	4	2	292	86	76	58
1	5	5	5	1	255	51	95	23
1	5	5	5	1	292	76	95	32
3	5	4	4	4	282	87	81	48
4	4	4	4	4	277	74	76	51
2	4	4	5	4	266	81	69	51
1	5	5	5	2	222	52	72	30
2	2	2	2	2	227	68	48	59
4	4	4	5	5	289	71	94	34
1	5	5	5	1	255	44	93	26
3	4	4	4	4	288	94	72	63
2	4	4	4	3	290	90	78	64
1	5	5	5	1	284	84	95	28
2	5	3	5	3	279	91	84	32
1	5	5	5	3	231	35	94	25
2	5	5	5	3	263	61	83	40

3	2	3	4	1	261	75	74	45
1	5	5	5	1	245	41	95	24
1	5	5	5	2	250	70	83	26
3	4	3	4	3	247	74	66	50
2	4	3	4	2	253	61	75	47
3	5	3	4	4	294	90	69	72
2	4	4	5	3	275	77	76	47
2	4	4	5	2	262	69	75	51
3	3	3	3	3	268	81	57	68
1	5	5	5	1	266	76	95	31
1	5	5	5	3	276	70	90	32
1	5	4	5	4	308	99	88	45
1	5	5	5	1	244	36	95	25
3	3	3	3	3	264	81	57	69
1	5	5	5	1	240	27	95	23
4	4	2	5	5	303	99	81	49
3	5	5	5	5	254	68	75	47
1	4	4	4	4	239	61	66	46
3	3	3	3	3	264	81	57	69
5	5	5	5	5	326	89	95	55
1	3	4	5	2	291	90	81	51
2	4	3	5	3	234	62	68	48
3	4	2	4	4	266	81	82	32
2	4	4	4	4	280	75	73	58
3	3	3	4	3	280	88	61	70
2	4	3	3	2	247	72	66	48
2	5	5	4	2	251	75	68	50
3	3	3	3	3	264	81	57	69
3	4	4	5	3	236	54	78	38
3	4	4	4	3	238	59	67	49
3	3	4	4	4	272	83	76	49
2	4	4	5	4	262	86	66	58
2	4	3	5	3	323	98	94	49
1	5	5	5	5	269	82	74	47
1	5	4	5	1	225	59	59	50
4	2	3	5	4	250	76	67	54
3	4	4	4	3	259	56	93	25
2	4	4	4	2	279	86	77	52
3	4	4	4	4	266	82	57	73
2	4	4	4	3	258	66	79	40
4	4	4	4	4	313	97	78	74
2	5	5	5	2	257	60	84	45
1	1	5	5	4	267	74	89	30
3	4	4	5	3	271	73	76	48
2	3	4	5	4	234	56	78	33
2	4	4	4	3	274	84	76	49
1	5	5	5	1	277	69	92	34
1	5	5	5	1	244	35	95	23
3	3	3	3	3	260	85	75	48
1	5	2	5	5	269	52	95	31
2	4	3	4	3	250	69	68	49
4	5	5	5	2	260	57	89	39
4	4	3	4	3	254	57	81	44
1	4	4	5	1	240	68	72	44
2	4	4	4	2	288	94	75	56
3	4	5	5	2	270	82	57	74

2	4	4	4	2	285	88	76	56
5	5	5	5	5	248	61	83	33
3	3	3	3	3	268	83	57	70
3	3	3	3	2	229	70	57	48
1	5	5	5	3	260	71	89	27
4	4	4	4	1	221	42	75	29
2	5	5	5	5	279	59	89	42
3	3	3	3	3	264	81	57	69
4	5	5	5	2	280	91	68	65
1	5	5	5	1	253	52	94	26
2	5	4	4	2	238	64	73	35
2	3	4	4	2	318	110	94	45
3	5	5	5	5	297	71	86	59
2	4	4	5	2	252	63	84	27
4	3	4	5	5	323	109	75	76
1	4	4	4	2	296	105	79	50
4	4	1	3	3	277	88	68	54
1	5	4	5	4	289	86	67	75
1	5	4	5	3	278	83	83	41
5	5	5	5	5	378	112	87	96
5	1	4	5	2	243	80	56	64
3	3	3	3	2	252	75	53	68
1	5	5	5	5	249	35	95	27
3	4	4	4	2	285	90	74	52
1	5	5	5	1	249	42	95	23
2	4	4	5	2	277	76	79	49
1	5	3	4	2	255	64	84	33
4	4	4	4	4	282	88	60	73
3	5	4	4	3	256	48	88	34
3	3	4	5	3	261	76	76	47
2	5	5	5	4	275	75	88	41
3	3	3	3	3	264	81	57	69
3	3	3	4	4	208	55	60	37
1	5	4	5	2	236	37	90	29
1	5	5	5	1	252	51	91	23
2	4	4	4	2	223	55	56	52
2	3	4	4	3	254	67	73	46
1	5	5	5	1	248	35	95	23
3	3	3	3	3	256	76	68	49
3	5	3	5	3	250	63	83	35
4	3	3	4	4	285	84	74	58
2	5	5	5	3	293	76	95	33
1	3	3	5	1	243	44	92	25
2	4	4	4	2	240	71	61	50
2	4	4	4	3	262	72	75	42
3	3	3	3	3	264	81	57	69
1	5	5	5	1	268	57	95	24
3	5	4	5	3	276	68	94	35
4	4	4	4	4	305	105	77	57
1	5	5	5	5	279	61	95	35
4	5	5	5	2	239	49	95	28
1	5	5	5	1	244	33	95	23
2	5	5	5	2	250	54	85	35
2	1	4	5	5	239	67	75	30
2	4	3	4	2	257	79	75	41
3	4	3	5	2	309	107	84	52

5	3	4	5	5	264	83	68	54
5	5	5	5	5	338	105	94	56
1	5	5	5	1	252	53	95	23
1	5	5	5	1	232	40	94	23
3	4	3	4	4	325	101	67	86
3	3	3	3	3	264	81	57	69
3	3	3	3	3	264	81	57	69
5	5	5	5	5	319	83	95	48
3	3	3	3	3	264	81	57	69
4	3	4	4	2	310	99	74	65
3	2	5	5	3	308	101	87	46
4	5	5	5	5	273	88	77	51
3	3	4	4	3	259	64	76	48
2	4	4	4	4	266	77	76	48
2	4	5	5	3	283	82	89	47
1	5	4	5	3	263	67	76	49
1	4	4	5	2	264	74	79	40
4	2	2	4	4	285	94	67	70
3	3	3	3	3	264	81	57	69
3	2	3	4	4	270	83	68	59
2	4	4	4	3	265	74	72	51
2	2	4	4	3	256	71	76	47
2	4	4	4	2	269	70	76	48
2	4	4	5	2	268	59	95	37
1	5	5	5	1	238	37	95	23
4	4	4	4	2	289	88	69	66
3	3	3	3	3	269	84	61	67
2	4	4	4	2	292	94	72	53
2	4	4	4	3	257	65	75	47
5	5	5	5	5	283	68	84	43
2	3	3	4	3	230	62	61	50
2	5	5	5	1	324	103	93	40
1	5	5	5	3	303	85	95	34
3	3	3	3	3	264	81	57	69
5	5	5	5	5	272	71	95	23
4	3	4	4	4	291	82	73	65
1	5	5	5	1	260	49	93	31
4	4	4	4	4	274	85	74	57
4	2	4	5	2	277	84	85	40
1	3	3	4	3	249	68	73	43
3	3	4	4	2	272	79	71	58
1	5	5	5	2	280	68	95	24
2	4	4	4	2	254	79	77	38
3	5	5	5	2	237	61	78	24
1	5	5	5	2	282	72	85	43
1	4	4	4	2	238	74	56	56
3	4	3	4	3	283	85	66	73
2	3	2	5	5	309	106	84	55
2	4	4	5	3	267	79	76	48
1	5	5	5	1	252	41	95	23
2	1	5	5	2	294	85	78	58
3	4	4	4	2	261	63	85	47
1	5	5	5	1	258	46	95	29
3	3	3	3	3	266	83	57	69
5	5	5	5	5	294	72	95	37
2	4	4	4	2	261	65	76	46

1	5	5	5	1	257	48	95	27
2	5	5	5	3	250	58	86	38
3	4	3	4	3	283	94	69	59
1	5	5	5	1	284	75	95	23
3	4	5	5	2	269	68	79	46
1	5	5	5	1	253	58	95	25
3	3	3	3	3	260	82	58	64
2	4	2	3	4	267	84	55	71
1	5	5	5	2	250	77	61	54
5	5	5	5	1	265	60	94	28
1	4	5	5	3	280	75	87	39
3	4	3	5	4	301	102	78	55
2	5	5	5	2	229	40	90	27
4	4	4	4	4	267	83	61	66
3	3	3	3	3	312	103	67	79
2	5	5	5	3	260	80	75	45
1	5	5	5	2	280	89	86	43
2	3	4	5	4	285	95	62	74
5	5	5	5	5	264	81	57	69
5	5	5	5	1	248	35	93	31
1	1	5	5	5	315	83	91	54
5	5	5	5	5	366	127	95	60
4	4	3	3	3	292	107	77	56
1	4	4	5	2	262	81	56	68
3	4	4	4	3	282	94	67	63
1	5	5	5	1	234	33	87	25
1	5	5	5	2	275	84	91	27
4	4	4	4	2	244	76	57	62
2	4	4	4	2	229	48	76	34
2	5	5	4	4	282	92	70	59
1	3	2	5	1	285	86	93	37
4	4	4	4	4	319	90	75	78
3	5	5	5	3	232	61	63	49
2	3	3	5	3	276	84	66	65
2	4	4	4	3	264	65	76	48
3	3	3	3	3	270	82	59	72
3	3	4	5	2	266	79	65	63
4	4	4	4	4	286	104	53	90
3	4	4	5	3	272	68	92	30
5	5	5	5	5	428	131	95	107
1	3	4	4	2	252	68	70	46
4	4	2	5	3	270	82	78	48
3	3	3	3	3	266	82	58	69
2	5	5	5	5	316	104	92	59
3	3	3	3	3	260	81	57	65
2	4	4	4	3	268	76	76	52
3	4	3	4	4	296	85	73	64
2	2	2	2	3	264	81	57	69
4	4	4	5	4	317	102	76	68
1	5	5	5	5	240	27	95	23
2	3	3	4	4	286	95	72	60
1	5	5	5	3	260	75	83	30
1	5	3	5	2	264	81	57	69
1	5	5	5	1	261	53	95	25
4	5	5	5	3	277	85	84	47
1	5	5	5	3	236	27	95	23

2	4	4	5	2	288	95	77	47
1	5	5	5	1	240	36	91	28
1	5	5	5	2	301	94	86	43
3	2	3	4	3	273	81	60	72
3	2	5	5	4	294	91	80	58
3	4	3	5	5	246	78	68	45
3	4	4	5	4	270	76	85	36
5	1	3	5	5	303	123	69	69
2	5	5	5	2	292	85	82	57
3	5	5	5	3	293	96	65	72
3	4	4	4	2	271	75	79	50
1	5	4	5	1	252	53	91	25
3	5	5	5	2	295	95	87	50
1	5	5	5	1	272	69	77	48
1	5	5	5	1	241	49	91	23
1	3	4	5	1	278	79	85	42
2	5	5	5	5	284	75	87	43
2	4	4	5	4	233	69	58	59
5	5	5	5	5	310	93	75	73
2	4	4	4	2	250	62	76	40
5	2	4	5	5	304	115	72	65
2	4	3	4	2	268	78	76	41
1	5	5	5	1	246	35	95	23
1	4	4	5	2	261	82	79	33
3	4	4	4	3	216	41	75	31
1	5	5	5	1	294	88	95	26
5	5	5	5	5	440	135	95	115
1	5	5	5	1	262	47	95	25
2	5	5	5	5	304	96	82	58
4	4	4	5	4	269	87	71	48
3	4	4	4	4	270	79	71	52
2	3	5	5	3	240	59	75	39
3	3	3	4	2	262	70	76	50
2	3	4	4	5	282	96	67	58
1	5	5	5	2	269	65	93	31
3	3	3	3	3	264	81	57	69
2	5	5	5	4	250	79	74	32
1	5	5	5	2	292	71	95	31
3	3	3	3	3	264	81	57	69
2	4	4	4	2	268	69	76	48
1	5	5	5	1	274	69	95	31
3	3	3	3	2	276	90	67	63
3	4	2	4	4	265	73	79	50
3	4	3	4	3	297	80	82	62
1	5	5	5	2	287	81	94	30
4	4	4	5	5	312	107	78	60
4	4	4	4	3	292	94	76	54
3	5	3	5	3	251	68	71	47
5	5	5	5	5	440	135	95	115
2	5	3	4	4	296	95	77	65
3	4	5	4	3	253	75	69	55
2	3	3	3	3	264	81	80	35
3	3	3	3	3	264	81	57	69
3	3	3	4	3	298	98	70	69
4	4	3	5	4	274	80	79	40
4	4	4	4	4	263	78	66	56

3	3	3	3	3	264	81	57	69
3	4	5	5	3	281	85	70	60
3	4	4	5	3	296	97	69	67
3	3	3	3	3	264	81	57	69
1	4	5	5	4	273	79	78	40
2	5	5	5	1	303	92	95	42
5	5	5	5	4	256	58	89	32
3	5	5	5	3	334	107	93	56
1	5	5	5	3	284	88	78	52
1	5	4	5	1	227	34	89	23
1	5	5	5	3	290	90	86	42
1	5	5	5	1	256	43	95	23
4	4	4	4	4	352	108	76	92
4	3	4	4	3	268	81	63	67
1	5	5	5	1	284	75	95	23
1	4	3	5	3	254	82	63	51
3	4	4	5	4	271	87	63	52
4	4	4	5	3	260	67	76	50
4	4	4	4	4	331	100	76	79
2	4	4	4	2	292	93	71	63
4	5	5	4	5	268	56	90	31
1	5	3	4	4	255	78	72	50
1	5	5	5	1	325	121	90	41
1	5	5	5	2	276	79	90	36
2	5	4	5	4	265	79	73	46
3	3	3	3	3	263	81	57	68
5	5	5	5	5	440	135	95	115
5	5	4	5	4	280	82	78	52
1	4	4	5	5	290	73	93	43
2	2	2	4	2	221	60	58	50
4	4	4	4	4	324	98	76	75
2	4	3	4	2	267	82	69	56
1	5	4	5	3	286	80	80	52
2	4	4	5	3	257	63	73	48
1	5	5	5	1	280	68	95	23
3	4	3	5	3	249	76	70	41
1	5	5	5	3	317	87	95	44
3	5	5	5	3	286	89	76	57
1	5	5	5	1	255	42	95	23
4	3	4	4	3	297	94	84	44
4	5	5	5	3	305	82	95	43
1	4	4	5	5	246	53	82	36
5	5	5	5	5	392	125	94	87
4	4	4	4	4	352	108	76	92
1	4	5	5	2	284	86	87	44
4	4	5	4	4	296	99	82	47
2	3	3	4	3	259	71	77	38
3	3	2	4	4	284	103	56	79
3	3	3	3	3	264	81	57	69
3	3	3	3	3	324	96	70	90
3	3	3	3	3	264	81	57	69
2	4	4	4	2	261	78	60	65
3	3	3	3	3	221	70	55	44
4	4	3	2	3	282	87	68	67
1	5	5	5	1	236	27	95	23
2	4	3	5	2	324	100	76	72

2	4	5	5	2	262	68	80	37
3	3	3	4	2	294	93	75	63
4	4	3	3	3	260	89	68	53
1	5	4	5	1	282	75	94	33
2	4	4	5	2	253	62	76	39
2	5	5	5	2	304	87	86	58
2	5	4	5	3	276	73	87	38
2	3	2	4	3	289	85	92	46
5	5	4	5	5	277	84	89	40
4	4	4	5	5	266	69	87	42
1	4	4	5	1	215	40	79	25
2	5	3	5	2	259	67	79	44
2	4	3	4	4	256	74	73	45
1	5	5	5	1	247	36	95	23
4	4	5	5	4	286	89	60	76
4	4	3	5	4	248	70	71	48
2	2	4	4	2	269	92	69	43
2	4	4	4	2	267	78	76	50
2	4	4	5	3	299	93	85	54
3	3	3	3	3	289	92	75	55
5	5	5	5	5	438	135	93	115
1	5	5	5	1	250	45	95	23
5	5	5	5	5	294	86	79	63
5	1	1	5	5	271	69	91	37
3	3	3	3	3	264	81	57	69
1	5	5	5	1	258	47	95	23
2	5	5	5	2	235	58	83	25
2	4	3	4	2	238	73	62	49
1	5	5	5	1	249	36	95	23
3	4	3	4	3	254	79	71	46
1	5	5	5	1	247	31	95	27
4	4	4	3	4	265	95	57	75
3	4	4	5	3	234	63	68	45
2	5	5	5	3	276	89	62	66
4	4	4	4	4	274	94	61	74
2	3	3	5	3	267	94	66	55
4	4	4	5	5	302	101	65	80
1	5	1	5	1	266	61	95	31
1	5	4	4	2	266	67	76	48
4	4	4	3	3	321	98	69	85
1	5	5	5	5	274	83	80	35
3	4	3	4	4	288	88	70	61
1	4	4	4	3	284	88	75	54
4	4	4	4	4	220	44	76	24
2	2	2	4	4	284	106	47	93
4	4	4	4	2	256	79	67	56
1	5	5	5	1	248	36	95	24
1	5	5	5	3	280	91	73	56
1	5	5	5	5	275	72	95	23
1	5	5	5	1	324	79	87	67
2	2	3	5	4	284	95	77	52
1	4	3	5	3	261	74	77	45
3	3	3	3	3	261	80	57	65
2	4	4	4	4	221	54	74	27
2	3	4	5	4	296	107	71	59
2	4	3	4	2	261	79	60	65

5	5	5	5	5	280	90	68	64
1	3	5	5	5	239	34	95	23
4	5	5	5	5	314	94	91	50
3	5	5	5	5	250	61	87	35
2	4	4	4	4	282	95	67	60
2	5	4	3	3	269	80	65	58
3	4	4	3	3	286	86	82	59
4	4	3	4	2	271	78	64	67
3	3	3	3	3	264	81	57	69
2	5	5	5	2	305	86	91	48
1	5	5	5	1	251	49	87	33
4	4	4	4	4	244	67	62	56
3	3	4	5	4	272	83	70	56
3	2	3	4	2	289	97	72	63
4	5	4	5	4	238	44	89	27
4	2	4	2	4	271	87	60	68
3	4	3	5	3	267	90	61	62
2	5	5	5	5	354	103	95	63
2	4	4	4	3	261	61	76	50
3	3	3	3	3	244	77	57	53
2	4	4	4	2	270	73	76	50
3	2	4	4	3	255	77	54	69
1	5	4	5	1	250	45	95	23
1	5	5	5	1	264	51	95	23
4	4	4	4	4	335	102	76	83
2	4	2	4	3	242	69	68	43
4	2	3	4	3	251	77	65	53
2	5	4	5	2	271	80	80	50
1	5	5	5	3	253	49	89	26
3	3	3	3	3	250	72	63	56
3	5	5	5	3	274	81	82	42
5	1	3	5	5	310	114	81	50
1	5	5	5	1	256	79	72	47
2	3	4	4	3	212	57	62	34
5	5	5	5	1	268	55	95	23
4	5	5	4	5	286	88	80	50
3	3	3	4	3	266	87	62	57
2	4	4	4	3	226	65	58	47
1	5	5	5	5	270	43	95	39
3	4	4	5	3	254	77	68	46
4	4	4	4	2	281	76	71	69
2	4	4	4	3	265	79	60	69
1	5	5	5	2	279	73	89	39
4	2	2	5	5	301	103	85	42
1	5	5	5	1	267	62	95	36
4	2	5	5	5	320	108	76	73
2	4	4	4	3	274	82	75	47
2	4	4	4	3	292	94	65	67
4	4	4	4	4	343	108	75	86
3	5	4	5	4	293	100	74	55
4	4	4	5	2	277	84	72	59
5	5	5	5	5	424	127	95	107
3	3	3	5	3	272	84	64	67
1	3	5	5	5	264	81	57	69
2	4	4	4	2	263	68	76	48
5	5	1	5	5	273	88	79	51

4	4	4	4	3	288	74	88	49
1	5	5	5	1	324	91	95	43
1	5	5	5	1	233	51	88	29
3	3	3	3	3	264	81	57	69
3	3	3	3	3	273	83	63	68
3	4	3	4	3	277	80	76	53
2	2	4	5	4	299	96	70	71
2	3	4	5	3	296	89	75	64
3	3	3	3	3	236	67	61	47
2	5	4	5	2	293	89	77	60
3	5	5	5	2	289	76	91	36
2	4	4	4	2	253	72	68	48
1	5	5	5	1	248	35	95	23
1	5	5	5	5	256	45	95	23
2	4	4	4	2	282	78	75	53
2	4	3	4	2	245	68	67	54
3	3	3	3	3	264	81	57	69
1	4	4	5	2	245	62	79	39
1	1	4	4	5	250	52	93	26
4	5	4	5	3	252	55	88	33
1	5	5	5	1	244	27	95	27
3	3	4	5	4	267	97	63	58
3	3	3	3	3	268	80	61	67
3	3	3	3	3	264	81	57	69
2	4	4	4	3	255	64	75	46
2	2	2	3	3	256	77	57	65
3	5	5	5	3	322	104	89	56
1	5	4	5	1	224	36	86	23
1	3	5	5	5	247	42	95	23
5	1	1	1	5	323	124	65	106
1	5	4	5	1	252	43	95	25
1	5	5	5	1	250	35	95	25
1	3	4	5	2	253	56	84	38
2	3	4	4	2	276	59	95	42
1	5	5	5	3	284	85	89	32
1	3	3	5	2	236	58	74	40
3	4	3	3	3	264	81	57	69
3	5	3	5	5	322	112	83	68
3	3	3	4	3	270	77	60	73
4	4	4	4	4	332	106	73	84
2	5	4	5	3	268	82	73	50
3	3	3	3	3	264	81	57	69
1	5	5	5	1	272	71	90	34
2	4	3	4	3	267	82	73	48
1	5	5	5	1	249	41	95	23
4	4	4	5	5	258	75	70	60
2	5	4	5	2	262	73	81	34
5	5	5	5	5	440	135	95	115
3	3	3	3	3	263	81	57	69
4	4	4	4	3	277	86	55	74
5	4	4	5	5	324	120	77	75
1	5	5	5	1	232	37	92	23
2	4	4	4	4	265	78	75	51
2	4	4	4	4	259	77	76	47
3	4	4	4	3	235	73	58	52
3	3	3	3	3	253	73	71	47

2	3	4	5	4	266	76	84	41
1	4	4	5	3	269	74	77	51
3	4	2	5	4	263	90	54	68
1	5	5	5	1	384	111	95	83
2	4	4	5	3	262	81	68	53
2	5	5	5	3	264	64	88	33
5	5	5	5	4	273	86	60	70
2	5	4	5	3	262	85	65	50
1	5	5	5	1	242	35	95	27
3	4	2	5	4	257	64	94	35
1	5	4	5	2	283	84	89	38
1	5	5	5	2	282	85	89	33
4	4	2	4	4	282	87	72	65
1	1	5	5	5	302	92	95	46
5	4	4	5	1	257	46	92	33
4	4	4	4	4	337	112	88	64
1	5	4	5	1	275	97	61	65
3	4	4	4	3	251	74	62	57
3	5	3	5	5	278	64	84	45
3	5	4	4	3	225	43	78	31
3	3	2	4	4	277	89	59	70
1	5	5	5	1	239	59	80	30
1	5	5	5	1	265	57	95	26
2	5	4	4	1	262	81	59	67
1	5	5	5	2	256	79	69	50
1	5	5	5	1	252	38	95	24
4	5	4	5	4	342	106	80	81
2	5	4	5	3	255	61	85	37
1	4	4	5	3	245	52	88	34
4	5	5	5	4	306	101	78	62
1	5	5	5	1	271	92	68	47
1	4	5	5	2	243	57	85	33
1	5	5	5	5	284	67	95	35
3	5	3	5	4	306	91	86	54
3	4	4	4	3	273	86	67	60
2	5	4	5	4	278	86	61	72
1	5	5	5	1	238	43	89	27
3	3	3	3	3	228	57	64	49
4	5	4	5	5	271	54	94	36
4	5	5	5	2	223	49	81	26
3	4	4	4	4	280	90	73	52
4	4	4	4	4	313	94	72	77
2	3	4	5	2	257	70	73	50
5	5	3	5	3	290	91	67	68
1	5	5	5	1	254	48	95	23
2	4	4	5	2	284	87	73	61
3	4	3	5	5	299	96	68	77
2	4	4	4	3	245	71	65	50
4	4	4	4	4	301	90	76	67

jjdh	ad1	ad2	ad3	ad4	da5	ad6
93	45	21	50	18	43	23
66	45	29	35	23	31	21
84	22	13	46	11	38	14
61	20	21	33	15	28	13
71	47	34	36	21	35	21
61	40	42	30	27	31	22
62	31	29	30	22	32	20
60	47	28	34	23	26	20
57	45	39	30	30	27	21
74	56	41	38	28	36	26
62	29	17	33	17	29	20
57	45	39	30	30	27	21
56	44	33	29	25	27	17
59	46	38	31	29	28	19
76	31	26	38	20	38	17
64	39	31	34	21	30	14
62	45	36	32	26	30	27
71	52	27	38	28	33	23
68	46	21	32	18	36	16
55	50	39	28	35	27	27
68	54	36	33	27	35	19
91	63	45	46	22	45	19
70	51	44	37	36	33	27
84	21	15	47	10	37	14
61	36	32	30	23	31	16
76	39	21	42	18	34	20
66	31	30	33	15	33	17
60	50	27	33	29	27	29
72	28	18	37	13	35	14
63	33	23	31	20	32	15
71	36	22	38	18	33	20
78	43	17	40	18	38	23
58	50	33	31	23	27	19
67	59	44	36	37	31	20
86	36	15	45	12	41	27
64	56	30	32	31	32	24
72	38	28	37	19	35	19
59	43	38	30	26	29	21
67	43	23	37	20	30	23
57	45	39	30	30	27	21
52	49	35	26	31	26	26
58	45	39	31	30	27	21
50	53	37	27	31	23	27
71	57	27	33	18	38	25
64	50	27	34	25	30	25
74	24	25	37	13	37	12
57	53	30	27	30	30	28
85	65	57	45	44	40	32
64	30	18	35	16	29	11
81	26	16	42	13	39	18

88	23	13	47	10	41	23
58	45	43	31	33	27	22
64	42	25	33	20	31	21
75	33	26	40	19	35	22
67	26	17	37	12	30	22
55	47	28	31	26	24	25
52	51	29	25	23	27	27
64	54	37	33	27	31	26
55	37	26	28	21	27	15
69	37	30	36	22	33	23
58	42	38	32	27	26	17
82	23	14	42	14	40	16
68	38	19	37	14	31	20
75	41	35	40	30	35	19
57	49	30	29	27	28	26
75	40	26	39	23	36	18
60	50	29	33	26	27	22
75	28	21	39	15	36	15
52	44	31	29	26	23	19
63	33	21	32	15	31	24
70	36	26	36	22	34	18
65	35	20	28	16	37	27
93	17	13	48	10	45	10
70	39	21	33	20	37	20
59	32	27	31	21	28	15
43	56	36	24	26	19	24
68	24	14	35	11	33	15
57	21	22	31	19	26	8
78	37	19	38	14	40	19
53	31	23	29	20	24	18
65	49	38	31	28	34	24
75	32	20	42	17	33	16
56	54	38	28	28	28	26
65	55	37	32	29	33	22
86	20	13	47	10	39	10
57	45	39	30	30	27	21
66	51	34	33	26	33	24
65	36	26	35	20	30	18
62	43	32	32	29	30	19
89	15	13	45	10	44	7
59	49	36	30	29	29	25
64	24	19	32	18	32	15
71	50	28	39	21	32	26
76	46	22	41	25	35	25
95	15	13	50	10	45	7
89	31	20	46	16	43	20
58	39	21	30	21	28	18
65	51	41	34	34	31	19
55	56	41	27	31	28	24
55	33	29	29	22	26	20
65	36	13	35	14	30	19
77	35	18	38	21	39	22
70	41	34	34	27	36	22
75	37	20	39	15	36	19
71	39	25	38	21	33	24
79	26	13	46	12	33	18

81	21	14	45	14	36	9
52	42	38	27	28	25	17
59	46	35	33	28	26	21
80	30	13	45	11	35	18
64	47	38	33	27	31	21
57	45	37	30	28	27	21
78	19	14	43	10	35	15
74	39	23	39	19	35	19
69	51	39	34	27	35	20
73	34	28	40	21	33	16
69	38	24	35	16	34	27
55	44	30	31	26	24	24
59	45	34	30	26	29	23
58	53	29	29	32	29	27
51	64	42	25	35	26	30
51	43	31	25	26	26	20
95	75	65	50	50	45	35
50	46	31	28	27	22	23
95	23	17	50	10	45	11
60	68	36	30	29	30	35
66	52	32	33	26	33	22
63	47	38	32	28	31	22
64	40	22	33	18	31	16
71	45	32	37	25	34	25
61	44	31	32	28	29	18
65	39	27	35	21	30	19
83	19	13	42	10	41	10
67	55	30	35	22	32	25
45	65	47	24	32	21	19
69	38	35	34	18	35	20
56	43	38	29	29	27	20
49	58	42	25	33	24	27
63	36	27	34	21	29	19
47	47	36	27	38	20	34
60	38	27	33	20	27	23
74	39	23	39	20	35	19
67	23	18	32	13	35	15
63	53	36	33	28	30	24
57	45	39	30	30	27	21
45	53	34	18	30	27	28
54	48	40	29	32	25	21
55	43	27	28	28	27	25
56	45	38	30	29	26	21
71	33	24	37	21	34	25
60	34	30	33	22	27	16
57	35	22	32	15	25	17
73	48	25	35	21	38	21
59	40	27	30	20	29	17
82	36	26	45	24	37	18
87	71	61	46	46	41	31
94	25	14	49	11	45	18
77	39	23	40	20	37	26
56	46	39	29	31	27	22
84	31	16	43	11	41	14
51	51	37	25	32	26	21
68	44	40	34	33	34	23

76	30	23	40	15	36	16
81	38	26	40	13	41	14
71	49	35	38	24	33	21
60	44	32	31	29	29	20
93	59	36	49	29	44	27
68	42	24	35	17	33	28
69	32	22	35	17	34	24
57	45	39	30	30	27	21
55	56	44	29	30	26	24
70	55	23	39	28	31	24
76	35	20	39	13	37	25
63	45	26	30	20	33	24
53	39	36	24	25	29	18
72	52	27	35	22	37	27
64	30	21	33	18	31	19
57	45	39	30	30	27	21
83	23	17	44	10	39	29
57	45	39	30	30	27	21
76	34	23	41	18	35	21
56	73	40	30	40	26	29
61	50	40	31	33	30	21
67	56	27	36	27	31	29
62	50	28	30	24	32	24
57	45	41	30	32	27	21
57	55	43	30	32	27	21
62	54	36	31	33	31	27
85	42	14	45	10	40	25
78	50	32	41	29	37	22
57	47	37	30	28	27	23
59	51	30	32	29	27	23
59	42	26	31	16	28	19
89	57	22	47	18	42	28
89	20	13	47	10	42	14
89	22	13	47	11	42	13
75	31	22	38	20	37	19
66	40	23	36	19	30	22
69	37	28	36	22	33	16
59	44	23	31	24	28	16
59	46	29	28	22	31	22
67	34	26	38	20	29	17
57	45	39	30	30	27	21
89	28	20	48	16	41	18
60	67	39	32	39	28	29
52	53	31	28	22	24	19
62	50	31	32	27	30	25
66	56	27	35	27	31	29
60	32	26	33	22	27	20
54	49	31	26	24	28	26
66	32	17	35	17	31	21
68	39	26	37	21	31	23
60	59	33	28	32	32	26
93	22	13	48	12	45	20
70	40	27	39	22	31	21
63	49	33	32	27	31	27
60	36	27	34	22	26	17
59	39	25	32	18	27	24

65	54	31	33	26	32	26
59	47	38	32	30	27	23
75	17	13	39	10	36	22
49	56	39	27	35	22	28
67	52	42	34	31	33	25
95	15	13	50	10	45	17
64	42	29	34	23	30	22
69	49	25	32	17	37	26
78	34	18	41	16	37	22
56	36	30	31	23	25	17
74	43	23	39	19	35	22
84	41	18	45	13	39	18
70	31	15	37	11	33	19
59	43	17	29	14	30	29
61	37	23	33	20	28	17
67	26	13	39	12	28	17
80	23	17	45	12	35	19
57	45	40	30	30	27	19
62	46	38	30	28	32	21
65	29	18	38	18	27	18
76	35	17	40	14	36	21
57	64	27	26	23	31	34
81	33	26	43	18	38	19
63	42	20	32	19	31	16
71	51	25	37	24	34	31
66	46	29	34	24	32	24
86	33	19	46	11	40	21
51	50	35	27	24	24	27
75	35	26	40	20	35	18
65	37	23	32	18	33	22
65	62	31	34	28	31	31
63	53	35	32	28	31	25
73	24	13	39	10	34	21
58	44	23	29	22	29	22
74	38	19	39	15	35	18
87	47	22	42	18	45	27
60	53	33	29	21	31	21
86	20	13	47	10	39	10
63	47	18	35	21	28	20
75	22	14	40	11	35	13
70	50	30	37	27	33	24
68	33	21	34	24	34	17
83	43	13	50	14	33	23
59	40	30	32	25	27	21
69	49	31	36	25	33	27
67	50	21	31	26	36	33
58	55	33	34	25	24	26
59	51	37	28	31	31	25
47	46	28	24	31	23	18
83	41	23	44	22	39	24
65	58	40	33	27	32	27
71	33	28	38	21	33	20
77	39	13	41	13	36	23
74	46	24	37	17	37	30
71	41	27	38	21	33	25
59	38	20	30	18	29	21

91	37	19	50	21	41	24
63	39	23	33	20	30	21
72	35	20	36	21	36	22
61	45	25	32	26	29	22
89	15	15	50	10	39	11
76	30	26	40	20	36	20
57	45	39	30	30	27	21
48	51	45	21	34	27	21
52	55	41	26	35	26	28
91	27	14	47	12	44	18
60	49	38	32	28	28	22
83	23	14	43	10	40	18
50	63	30	27	36	23	30
66	31	20	31	16	35	24
81	62	56	44	44	37	31
95	75	65	50	50	45	35
76	61	21	38	29	38	23
57	45	39	30	30	27	21
57	45	40	30	30	27	22
65	42	18	34	22	31	24
67	41	24	36	22	31	25
61	42	35	33	25	28	23
62	41	29	32	21	30	19
61	67	30	30	22	31	31
70	34	24	38	18	32	18
70	39	33	37	29	33	19
61	42	33	33	25	28	24
59	57	39	31	32	28	28
63	37	25	33	21	30	22
74	24	16	39	16	35	16
71	16	15	38	14	33	11
64	42	28	34	23	30	19
85	15	17	46	10	39	7
60	49	40	31	31	29	22
73	50	23	40	21	33	29
69	34	17	33	12	36	25
78	20	13	43	10	35	24
59	40	25	31	21	28	17
69	25	19	37	13	32	16
69	25	19	37	13	32	16
66	25	17	35	14	31	15
95	23	15	50	10	45	11
57	51	29	28	29	29	23
91	20	19	50	15	41	15
70	47	29	37	25	33	21
72	24	18	36	12	36	12
95	75	65	50	50	45	35
76	26	14	39	11	37	17
63	52	27	31	28	32	22
71	27	16	38	10	33	16
59	50	37	31	30	28	24
41	24	50	25	26	16	11
60	40	30	32	26	28	20
78	32	19	40	15	38	20
57	45	39	30	30	27	21
70	36	17	32	11	38	26

95	55	45	50	30	45	23
65	43	23	33	21	32	22
62	50	42	30	32	32	16
74	38	13	40	10	34	17
72	37	32	39	22	33	20
57	54	37	30	29	27	25
70	39	28	37	20	33	19
56	47	36	29	29	27	20
64	34	21	32	21	32	20
57	45	39	30	30	27	21
61	44	26	33	21	28	25
95	32	14	50	20	45	32
70	47	39	38	30	32	24
76	60	52	40	40	36	28
84	18	13	45	10	39	12
70	37	23	38	21	32	19
94	20	13	50	10	44	12
76	39	20	44	14	32	14
56	43	27	30	22	26	20
93	29	22	49	15	44	15
80	60	26	43	24	37	29
68	44	27	36	21	32	24
71	34	28	36	20	35	16
63	42	33	35	24	28	26
73	29	23	42	14	31	9
74	37	23	38	18	36	13
65	36	22	32	20	33	20
70	48	31	37	24	33	24
77	29	15	39	12	38	13
65	41	26	33	22	32	18
70	39	36	35	27	35	17
49	49	36	25	32	24	27
68	35	22	36	19	32	20
72	35	20	40	12	32	20
53	35	26	29	20	24	18
84	54	28	43	18	41	31
69	56	31	34	31	35	26
68	47	30	34	26	34	24
62	42	26	32	24	30	20
65	39	23	35	20	30	18
70	57	31	35	29	35	25
68	61	44	33	37	35	25
64	56	30	31	34	33	30
64	42	34	34	23	30	24
71	56	33	37	30	34	29
87	30	14	46	13	41	27
79	34	17	41	12	38	14
50	47	32	28	31	22	27
84	18	13	45	10	39	11
67	38	37	35	23	32	17
69	47	16	37	15	32	25
70	25	22	37	16	33	16
95	75	65	50	50	45	35
55	45	30	31	28	24	24
88	31	26	49	19	39	16
57	45	39	30	30	27	21

55	44	39	28	30	27	21
77	51	21	41	22	36	31
95	23	13	50	10	45	7
67	43	17	30	13	37	27
58	45	38	30	28	28	22
71	42	21	37	17	34	20
70	43	28	39	23	31	21
67	41	25	35	19	32	27
87	23	13	50	10	37	9
63	42	36	33	25	30	22
75	43	24	38	21	37	27
68	33	27	36	21	32	15
47	54	33	25	32	22	26
74	56	24	35	26	39	27
52	44	37	29	30	23	22
57	44	39	30	29	27	21
62	49	26	30	25	32	23
83	35	16	41	17	42	18
68	50	25	32	20	36	25
74	48	28	38	22	36	26
70	43	35	34	22	36	31
58	41	28	30	24	28	23
64	55	40	33	35	31	25
86	28	13	48	12	38	16
58	46	30	29	24	29	25
65	43	23	33	17	32	25
66	33	19	35	18	31	11
60	43	32	29	27	31	24
53	54	42	27	32	26	32
53	69	43	26	43	27	35
57	45	36	31	33	26	18
83	44	19	45	15	38	26
83	25	15	44	15	39	14
67	45	21	31	20	36	25
52	48	25	28	27	24	26
68	42	28	37	25	31	21
56	54	25	28	24	28	28
95	75	65	50	50	45	35
72	35	23	39	20	33	20
70	39	36	37	19	33	16
72	44	33	37	25	35	26
86	31	13	43	10	43	11
89	38	17	46	15	43	21
66	46	24	34	24	32	25
76	41	28	40	23	36	22
65	43	27	34	24	31	22
68	23	14	39	16	29	18
52	40	34	30	25	22	17
90	38	19	50	15	40	21
92	23	13	48	13	44	11
59	54	35	31	28	28	23
58	51	35	29	29	29	26
77	50	15	41	13	36	23
72	49	15	37	17	35	28
77	19	14	41	11	36	9
79	30	23	41	17	38	18

67	43	23	35	22	32	19
85	19	14	48	10	37	13
71	35	14	39	12	32	25
57	40	28	28	22	29	19
70	32	27	38	20	32	18
63	54	39	33	33	30	20
75	40	27	39	20	36	23
67	40	27	36	24	31	19
62	45	38	32	30	30	21
64	44	13	35	18	29	23
84	40	19	42	13	42	18
76	53	22	39	23	37	29
88	18	15	48	10	40	10
57	45	39	30	30	27	21
95	15	13	50	10	45	7
74	55	30	39	19	35	27
64	30	27	32	20	32	23
66	32	26	36	20	30	17
57	45	39	30	30	27	21
87	43	29	46	26	41	27
69	45	28	34	23	35	30
56	35	26	29	22	27	15
71	40	19	38	13	33	26
74	41	33	38	25	36	19
61	49	40	33	30	28	22
61	40	26	35	22	26	19
58	43	24	32	26	26	20
57	45	39	30	30	27	21
66	30	18	34	20	32	9
63	32	28	34	21	29	17
64	45	27	33	22	31	23
52	50	29	30	29	22	21
82	55	25	41	24	41	28
66	45	26	35	21	31	23
57	31	28	30	22	27	15
53	43	28	27	26	26	20
85	30	13	44	12	41	15
64	48	28	34	24	30	23
54	47	43	30	30	24	22
73	37	23	40	17	33	19
64	51	40	33	34	31	25
68	35	26	33	19	35	17
74	38	14	37	16	37	17
74	38	27	38	21	36	20
67	28	17	35	16	32	18
65	49	26	32	23	33	20
82	37	17	44	17	38	21
91	15	13	50	10	41	15
52	51	27	28	21	24	23
91	26	20	46	11	45	18
64	40	27	33	22	31	17
75	34	21	36	18	39	14
72	31	26	38	18	34	15
56	40	24	30	20	26	16
63	55	30	35	26	28	25
57	48	42	30	32	27	21

65	52	27	37	29	28	24
71	32	15	34	18	37	19
58	47	40	31	30	27	21
54	37	27	29	21	25	22
73	37	16	37	11	36	25
75	23	16	40	13	35	12
89	27	23	46	19	43	16
57	45	39	30	30	27	21
56	48	33	32	32	24	30
81	23	13	44	13	37	22
66	36	18	34	17	32	15
69	58	20	32	25	37	32
81	41	35	42	24	39	17
78	30	14	39	13	39	19
63	58	43	30	33	33	30
62	55	27	32	23	30	33
67	46	33	36	21	31	25
61	48	46	31	29	30	21
71	45	20	34	21	37	22
83	59	54	43	42	40	32
43	40	32	26	32	17	25
56	40	39	29	29	27	20
92	18	15	49	12	43	8
69	48	29	37	23	32	27
89	24	13	50	10	39	13
73	40	29	38	20	35	22
74	35	20	39	13	35	19
61	49	41	32	32	29	23
86	23	19	45	15	41	14
62	42	24	33	23	29	21
71	40	22	37	19	34	27
57	45	39	30	30	27	21
56	32	22	31	15	25	13
80	18	18	41	11	39	10
87	23	13	50	10	37	19
60	33	30	34	22	26	12
68	37	27	35	19	33	18
95	19	13	50	10	45	7
63	42	29	29	20	34	23
69	35	20	36	15	33	20
69	47	30	35	28	34	23
89	34	19	47	14	42	26
82	21	15	40	10	42	11
58	39	28	31	22	27	19
73	37	24	37	18	36	23
57	45	39	30	30	27	21
92	27	13	48	11	44	19
79	37	17	43	18	36	20
66	59	30	33	27	33	27
88	31	17	47	18	41	13
67	20	15	37	13	30	19
93	15	13	50	10	43	11
76	25	19	41	16	35	20
67	30	16	36	14	31	21
62	42	22	31	19	31	23
66	55	30	32	22	34	31

59	46	32	30	22	29	20
83	58	31	40	25	43	32
81	24	13	43	10	38	22
75	17	13	41	10	34	16
71	56	49	37	37	34	25
57	45	39	30	30	27	21
57	45	39	30	30	27	21
93	43	22	48	26	45	23
57	45	39	30	30	27	21
72	54	36	39	29	33	26
74	56	30	37	16	37	27
57	46	23	32	28	25	25
71	38	26	38	22	33	14
65	39	28	36	20	29	24
65	47	24	32	23	33	21
71	37	27	38	22	33	18
71	38	21	38	19	33	21
54	52	38	27	32	27	27
57	45	39	30	30	27	21
60	50	31	32	28	28	19
68	37	28	35	23	33	19
62	45	26	33	21	29	18
75	33	28	39	20	36	23
77	35	19	41	18	36	15
83	17	13	48	10	35	14
66	48	36	35	30	31	26
57	48	38	30	29	27	21
73	51	31	39	22	34	25
70	33	26	37	21	33	22
88	40	31	43	12	45	11
57	35	27	29	23	28	16
88	57	20	46	20	42	30
89	45	22	48	12	41	21
57	45	39	30	30	27	21
83	41	13	48	10	35	17
71	46	37	38	28	33	22
87	23	15	50	16	37	17
58	48	33	28	24	30	21
68	50	22	37	18	31	24
65	34	24	32	19	33	21
64	42	32	33	26	31	24
93	33	14	49	10	44	23
60	40	23	29	15	31	24
74	30	13	37	11	37	17
82	38	25	43	18	39	16
52	41	31	29	25	23	22
59	48	39	31	34	28	22
64	64	30	33	25	31	30
64	41	27	35	21	29	22
93	17	13	48	10	45	11
73	49	40	39	18	34	23
66	37	26	36	21	30	18
88	22	18	50	11	38	15
57	45	39	30	30	27	23
90	37	16	47	21	43	18
74	33	26	40	20	34	18

87	24	17	46	10	41	19
68	31	21	36	17	32	17
61	54	36	28	23	33	25
91	37	13	48	10	43	19
76	36	27	39	19	37	18
75	31	15	38	10	37	15
56	45	36	30	28	26	21
57	46	40	32	31	25	22
58	42	27	32	27	26	20
83	27	13	40	15	43	24
79	32	21	40	18	39	27
66	55	26	35	29	31	28
72	20	15	36	12	36	12
57	48	37	31	29	26	22
63	61	42	36	37	27	27
60	41	23	30	22	30	28
62	45	22	33	21	29	30
54	54	41	29	33	25	25
57	45	39	30	30	27	21
89	19	13	49	18	40	11
87	37	27	45	27	42	27
84	67	29	47	31	37	35
52	54	28	28	28	24	32
57	45	38	30	30	27	21
58	54	34	31	29	27	23
89	18	14	48	11	41	10
73	42	15	37	12	36	28
49	40	35	24	27	25	19
71	24	20	38	14	33	14
61	51	34	32	25	29	24
69	40	18	36	19	33	28
76	54	46	40	32	36	18
59	33	25	32	24	27	17
61	46	37	31	28	30	24
75	34	26	40	22	35	15
57	45	41	30	31	27	22
59	42	37	29	26	30	21
39	60	53	21	37	18	31
82	37	17	43	13	39	20
95	71	57	50	50	45	35
68	36	27	37	19	31	19
62	43	27	34	21	28	24
57	46	39	30	30	27	21
61	59	30	32	29	29	28
57	45	35	30	30	27	21
64	43	30	34	22	30	19
74	46	35	38	29	36	23
57	45	39	30	30	27	21
71	57	32	35	36	36	26
95	15	13	50	10	45	7
59	50	29	32	31	27	27
72	38	16	39	14	33	22
57	45	39	30	30	27	21
88	28	14	48	11	40	16
61	40	22	29	25	32	30
91	15	13	50	10	41	7

69	55	22	36	25	33	28
85	19	16	45	12	40	9
78	54	26	41	17	37	25
60	45	44	31	28	29	22
65	57	27	33	31	32	20
55	36	23	25	22	30	23
73	35	20	38	16	35	27
42	69	34	18	35	24	35
68	45	33	33	24	35	25
60	57	38	30	34	30	27
67	44	27	35	23	32	18
83	29	14	43	11	40	13
63	50	29	31	21	32	27
78	38	28	42	20	36	17
78	19	13	42	10	36	20
72	44	22	34	20	38	19
79	34	23	42	20	37	28
47	39	37	24	22	23	17
69	45	41	36	32	33	27
72	33	21	38	19	34	17
52	62	34	23	31	29	35
73	37	22	40	19	33	24
93	15	13	50	10	43	7
67	42	18	35	15	32	24
69	22	15	38	16	31	13
85	39	15	40	11	45	35
95	75	65	50	50	45	35
95	23	13	50	12	45	19
68	54	30	36	28	32	26
63	47	26	32	22	31	26
68	45	27	35	25	33	21
67	30	24	34	15	33	18
66	42	27	35	23	31	17
61	54	30	32	28	29	27
80	30	15	45	16	35	22
57	45	39	30	30	27	21
65	41	17	33	15	32	24
95	26	17	50	14	45	28
57	45	39	30	30	27	21
75	33	28	40	20	35	23
79	39	15	43	16	36	16
56	51	33	27	30	29	25
63	40	29	35	21	28	19
73	46	34	40	28	33	20
82	37	18	46	12	36	30
67	59	29	34	31	33	29
68	50	31	36	23	32	27
65	37	29	33	18	32	19
95	75	65	50	50	45	35
59	52	32	33	33	26	27
54	44	31	28	24	26	18
68	42	17	35	18	33	20
57	45	39	30	30	27	21
61	54	36	31	33	30	27
75	41	24	39	16	36	25
63	44	28	35	28	28	24

57	45	39	30	30	27	21
66	46	33	34	27	32	24
63	53	38	30	29	33	27
57	45	39	30	30	27	21
76	42	24	38	16	38	23
74	48	22	37	20	37	26
77	32	18	39	14	38	18
78	57	30	41	26	37	31
66	51	29	35	23	31	28
81	19	13	43	10	38	10
72	44	22	37	20	35	28
95	21	13	50	10	45	13
76	60	52	40	40	36	28
57	45	39	30	28	27	21
91	27	13	46	10	45	33
58	42	27	30	24	28	24
69	46	28	36	24	33	24
67	35	28	34	22	33	18
76	55	41	40	38	36	26
65	49	35	34	28	31	26
91	37	16	46	15	45	11
55	43	24	31	26	24	26
73	67	29	41	12	32	35
71	40	19	39	17	32	27
67	44	26	37	20	30	21
57	45	38	30	30	27	21
95	75	65	50	50	45	35
68	42	29	35	23	33	23
81	43	26	43	17	38	20
53	36	26	31	24	22	14
75	53	40	39	35	36	27
60	46	31	31	25	29	21
74	46	32	38	20	36	21
73	35	27	38	21	35	16
94	28	13	49	10	45	26
62	40	22	30	19	32	22
91	43	22	48	22	43	28
64	47	28	33	29	31	25
95	18	13	50	10	45	19
75	48	23	38	21	37	29
85	46	23	43	20	42	23
75	25	22	40	14	35	18
86	70	47	47	40	39	30
76	60	52	40	40	36	28
67	45	25	35	19	32	26
68	43	28	32	19	36	35
73	34	20	36	18	37	22
46	60	43	25	36	21	26
57	45	39	30	30	27	21
68	54	51	35	39	33	26
57	45	39	30	30	27	21
58	43	36	31	29	27	20
52	41	24	28	20	24	20
60	47	37	32	30	28	22
91	15	13	46	10	45	7
76	53	41	40	31	36	28

77	35	19	41	18	36	15
63	55	32	34	31	29	24
50	51	27	23	26	27	23
80	35	17	45	16	35	26
76	31	22	40	17	36	19
73	47	34	37	24	36	25
78	34	21	42	17	36	24
66	52	26	33	20	33	22
64	46	20	32	20	32	24
68	32	17	35	25	33	24
71	20	15	38	10	33	13
69	36	24	36	20	33	18
64	43	25	33	20	31	22
93	20	13	50	10	43	11
61	47	44	32	32	29	26
59	37	26	32	22	27	20
65	48	24	35	19	30	29
63	45	27	34	23	29	21
67	52	30	35	24	32	26
67	48	32	37	23	30	26
95	75	65	50	50	45	35
87	24	13	49	10	38	13
66	47	36	34	27	32	22
74	36	19	38	18	36	17
57	45	39	30	30	27	21
93	23	13	50	10	43	17
69	28	14	32	11	37	17
54	43	28	29	21	25	18
95	16	13	50	10	45	15
58	47	25	30	21	28	19
94	15	17	50	10	44	11
38	48	47	19	28	19	29
58	34	24	31	21	27	18
59	47	34	30	32	29	27
45	54	41	23	33	22	25
52	54	31	25	24	27	26
56	57	45	29	35	27	26
79	27	17	42	14	37	27
75	38	26	40	22	35	17
69	56	48	35	37	34	25
76	40	21	40	14	36	26
69	51	33	36	28	33	21
67	43	31	36	23	31	28
76	22	13	40	11	36	14
38	59	52	19	41	19	29
54	45	32	27	24	27	22
93	17	14	50	10	43	10
60	50	30	30	26	30	26
85	29	13	46	10	39	25
91	55	41	50	26	41	19
60	52	25	30	27	30	27
65	42	26	33	19	32	17
59	44	35	30	30	29	21
66	27	15	34	12	32	17
59	58	31	30	28	29	28
57	45	36	30	29	27	18

58	54	32	32	32	26	22
87	16	13	46	10	41	12
79	53	24	43	26	36	24
67	33	19	39	16	28	16
60	53	34	31	26	29	26
66	44	30	34	28	32	21
59	52	31	29	28	30	21
62	48	36	33	31	29	17
57	45	39	30	30	27	21
80	44	25	42	23	38	27
82	22	18	46	15	36	15
59	35	32	32	24	27	17
63	48	29	34	27	29	20
57	53	35	28	28	29	26
78	19	17	42	10	36	14
56	46	38	29	30	27	24
54	49	35	29	27	25	25
93	56	34	48	29	45	27
74	35	29	40	21	34	16
57	43	27	30	26	27	21
71	41	28	39	22	32	20
55	46	40	28	29	27	20
87	21	13	45	10	42	19
95	23	13	50	10	45	19
74	54	43	40	40	34	28
62	41	23	35	20	27	15
56	43	28	31	25	25	22
61	46	26	31	24	30	20
89	24	13	49	13	40	13
59	38	32	31	24	28	20
69	45	23	35	19	34	20
65	60	29	32	21	33	31
58	44	22	33	25	25	22
59	29	21	32	13	27	17
95	31	13	50	10	45	19
68	43	27	35	23	33	26
60	48	36	28	21	32	25
56	38	26	29	21	27	17
93	23	21	48	18	45	15
63	41	23	33	23	30	24
65	46	39	33	30	32	17
57	45	39	29	30	28	20
78	40	24	42	15	36	24
71	53	22	33	20	38	33
74	30	20	39	16	35	21
63	59	41	28	32	35	29
70	41	24	36	23	34	24
66	53	38	33	29	33	25
74	60	48	39	38	35	27
64	54	27	32	28	32	28
62	51	37	33	22	29	19
95	71	61	50	46	45	31
57	48	40	30	27	27	23
57	45	39	30	30	27	21
71	38	27	39	21	32	17
55	44	33	29	18	26	27

77	36	23	40	26	37	21
95	43	25	50	18	45	31
65	26	15	37	14	28	17
57	45	39	30	30	27	21
59	48	37	31	31	28	21
68	44	28	38	25	30	23
62	56	38	33	33	29	23
68	51	35	35	29	33	24
61	37	27	32	20	29	20
67	47	34	33	26	34	26
86	41	19	48	17	38	24
65	40	26	33	22	32	21
95	15	13	50	10	45	15
93	29	13	48	10	45	7
76	40	29	40	24	36	24
56	40	31	29	23	27	16
57	45	39	30	30	27	21
65	33	20	33	19	32	16
79	28	14	44	12	35	13
76	34	18	40	15	36	12
95	15	17	50	10	45	7
49	53	33	24	25	25	31
60	45	38	31	29	29	20
57	45	39	30	30	27	21
70	32	26	38	20	32	20
57	44	37	30	28	27	18
73	62	29	38	27	35	25
79	18	13	42	10	37	12
87	18	13	50	10	37	11
28	71	61	12	45	16	35
89	25	13	48	12	41	11
95	19	13	50	12	45	11
75	30	20	37	18	38	17
80	31	23	43	19	37	18
78	49	13	40	19	38	18
64	34	24	33	16	31	15
57	45	39	30	30	27	21
59	58	32	30	36	29	33
60	44	40	33	33	27	18
69	58	47	35	37	34	29
63	46	28	32	22	31	23
57	45	39	30	30	27	21
77	31	17	37	17	40	27
64	44	26	34	22	30	23
90	21	13	49	10	41	15
53	41	37	27	23	26	21
74	41	18	38	16	36	22
95	75	65	50	50	45	35
56	45	39	30	30	26	21
62	52	41	33	33	29	20
52	71	36	27	39	25	34
80	23	13	43	10	37	9
61	46	28	29	23	32	20
59	44	27	29	20	30	21
52	42	26	29	26	23	18
62	41	26	32	21	30	18

65	41	19	33	22	32	26
67	36	29	34	22	33	24
51	53	37	26	31	25	22
95	55	45	50	38	45	35
60	46	27	34	26	26	23
79	32	23	38	10	41	16
57	48	39	29	31	28	23
62	47	28	35	22	27	25
85	19	17	46	10	39	9
64	31	17	38	18	26	20
72	46	20	37	18	35	24
75	46	16	40	17	35	26
58	44	36	32	29	26	28
69	50	23	36	23	33	25
86	25	17	47	16	39	14
73	62	32	35	32	38	26
52	56	37	26	28	26	25
58	42	30	31	27	27	18
85	35	25	45	20	40	16
73	23	18	39	13	34	12
59	52	40	29	30	30	23
70	29	15	37	15	33	17
87	30	14	48	12	39	17
55	45	38	29	29	26	23
58	45	29	30	21	28	19
95	20	14	50	10	45	8
75	54	44	39	37	36	30
72	32	20	40	17	32	16
71	26	20	39	14	32	14
65	55	31	34	31	31	28
64	52	28	33	19	31	24
68	31	20	36	13	32	16
87	31	17	42	18	45	27
75	47	31	35	23	40	28
60	48	32	31	28	29	22
59	47	40	30	32	29	24
79	19	17	45	10	34	15
58	33	29	31	20	27	15
87	29	20	45	16	42	14
67	25	15	36	11	31	16
65	50	28	32	24	33	23
70	52	42	36	35	34	24
64	44	27	32	23	32	16
64	52	40	32	28	32	24
88	30	13	49	10	39	9
63	51	33	33	28	30	23
58	55	44	30	33	28	25
59	36	29	31	21	28	21
68	51	40	34	27	34	25

ad7	ad8	ad9	ad10	a1	a2	a3	a4	a5	a6
35	35	13	25	5	1	5	1	5	5
28	31	11	17	5	4	4	5	4	5
31	33	10	25	2	1	5	1	5	3
23	25	11	14	1	1	2	1	4	1
25	27	13	18	4	4	3	2	4	4
20	22	17	17	4	3	3	3	4	4
24	25	12	18	5	3	3	3	3	4
26	28	16	19	4	1	4	4	4	3
21	21	15	15	3	3	3	3	3	3
28	28	19	20	2	4	2	3	4	2
30	31	9	23	2	2	4	2	4	4
21	21	15	15	3	3	3	3	3	3
26	27	12	18	5	3	3	3	4	2
26	26	13	18	4	4	3	3	3	3
30	31	10	24	3	3	4	3	5	1
24	25	11	18	4	3	3	2	3	3
25	29	13	15	3	3	4	3	4	4
27	29	17	21	4	2	4	3	4	3
25	33	15	18	5	1	4	2	5	2
22	23	11	15	4	5	3	3	3	3
28	31	19	21	4	2	4	2	4	1
35	35	17	25	5	1	5	1	5	1
25	26	20	20	4	3	4	4	4	4
34	35	10	24	2	2	5	1	5	1
22	24	11	15	4	2	3	2	3	2
29	30	10	21	2	3	4	3	5	3
25	26	15	20	3	3	3	2	3	1
29	30	14	16	4	3	3	4	3	4
28	28	10	19	3	1	4	2	4	2
28	29	12	18	4	3	3	3	4	2
27	28	15	20	3	2	4	1	4	3
35	35	13	25	4	1	5	2	5	2
25	25	14	15	4	4	3	2	3	3
23	27	15	12	5	3	4	4	3	4
34	35	12	24	3	1	5	2	5	4
28	31	19	20	5	3	3	4	4	4
27	28	13	19	4	2	3	2	4	3
21	22	15	15	3	3	3	3	3	3
28	28	12	19	3	2	4	2	4	3
21	21	15	15	3	3	3	3	3	3
23	24	14	16	4	3	3	3	3	4
21	21	15	15	3	3	4	3	3	3
25	28	13	15	4	3	3	2	3	4
32	35	15	21	5	3	3	3	4	3
28	28	16	18	4	4	3	3	4	3
28	30	12	20	3	2	4	2	5	1
28	29	16	19	5	2	4	4	4	4
32	32	24	24	5	5	5	5	5	5
26	23	12	17	2	3	3	2	3	1
32	32	12	22	2	2	4	2	5	3

35	35	13	25	1	1	5	1	5	5
20	24	16	14	3	4	3	3	4	2
27	28	11	20	3	2	4	2	4	3
29	29	14	19	4	2	4	3	4	3
28	31	9	21	3	2	4	1	4	5
27	29	16	18	4	2	3	3	3	3
30	31	18	18	5	2	3	2	4	4
27	29	17	19	4	3	3	3	4	4
21	19	13	13	3	2	3	3	3	3
25	30	12	18	4	3	4	4	4	3
21	27	14	14	2	4	3	3	3	3
32	30	14	22	4	1	4	1	5	1
29	30	11	17	3	2	4	2	4	5
28	33	21	21	5	5	5	5	3	5
22	25	16	16	5	3	4	3	3	4
28	28	13	20	4	3	4	4	4	4
26	29	10	16	3	2	4	2	4	3
28	28	11	20	3	2	4	2	4	3
22	24	11	15	4	4	3	3	3	4
26	33	11	17	3	2	3	2	4	4
28	28	14	20	3	2	4	2	4	3
31	34	16	20	4	1	3	1	5	3
35	35	7	25	1	1	5	1	5	3
30	32	8	20	5	1	4	4	5	5
21	22	12	15	2	3	3	2	3	2
22	24	14	16	3	3	3	3	3	4
29	33	8	22	2	1	4	1	4	2
20	24	10	14	2	2	3	2	3	1
35	35	9	25	5	1	3	2	5	4
26	29	10	15	2	2	3	3	4	4
26	26	15	17	4	3	3	3	4	4
28	29	10	22	4	4	4	2	4	4
25	26	16	17	4	3	3	3	3	3
29	31	11	19	5	5	2	5	5	3
35	35	10	25	2	1	5	1	5	1
21	21	15	15	3	3	3	3	3	3
32	33	21	23	4	3	3	3	5	3
28	26	11	18	3	2	4	2	4	3
22	23	14	16	2	2	3	3	3	3
35	35	7	25	1	1	5	1	5	1
25	26	11	16	4	4	4	3	4	4
24	26	11	19	1	3	3	3	5	2
28	29	15	20	4	3	4	3	4	4
34	34	11	23	5	2	5	2	5	2
35	35	9	25	1	1	5	1	5	1
35	35	13	24	2	2	4	3	5	4
21	23	16	15	3	2	3	3	3	3
27	26	15	16	4	4	3	4	4	3
22	26	15	15	5	4	3	3	4	4
21	21	14	15	2	2	3	3	3	3
27	27	10	19	3	1	3	2	3	3
30	31	13	22	2	1	4	1	5	4
28	29	19	20	4	3	4	3	4	2
32	34	14	24	2	2	4	2	5	5
29	31	13	21	5	2	4	3	4	3
35	35	9	25	3	1	4	2	5	3

35	35	7	25	1	2	5	1	5	1
21	19	11	15	3	2	3	3	3	2
28	24	10	20	4	2	3	4	4	4
35	35	12	25	3	1	5	1	5	2
21	22	13	14	4	3	3	3	4	3
21	21	13	16	3	1	3	1	3	3
29	30	11	21	3	2	4	1	4	3
30	30	10	21	3	1	4	2	4	4
23	24	14	18	4	2	4	3	4	2
23	28	12	17	4	3	4	2	4	3
33	35	12	23	5	2	4	1	5	5
25	26	12	17	4	3	3	4	3	4
23	25	14	17	4	3	3	2	4	4
27	30	12	20	5	4	3	4	4	4
29	28	19	19	4	3	3	3	4	5
19	20	16	16	3	4	2	3	2	2
35	35	25	25	5	5	5	5	5	5
28	31	12	15	3	3	3	4	3	4
31	35	5	25	1	1	5	1	5	1
29	33	15	25	5	3	5	4	5	5
32	32	14	17	5	3	4	4	4	3
21	21	15	18	3	2	3	3	4	3
28	29	12	19	4	1	4	3	4	2
27	28	12	19	4	4	4	3	4	4
25	26	12	17	5	3	3	3	3	3
28	28	12	20	3	2	4	2	4	3
35	35	8	21	3	1	4	1	4	1
28	30	17	20	4	2	3	2	4	3
35	35	21	21	5	5	1	5	3	3
20	29	16	12	3	3	2	3	4	5
21	21	14	15	3	3	3	3	3	3
22	25	17	14	5	4	3	3	2	4
21	21	13	16	2	2	3	2	4	3
22	17	14	12	5	1	3	3	1	5
25	26	14	18	4	3	3	2	3	2
31	32	15	22	3	2	4	2	4	3
30	29	10	16	3	2	4	1	4	2
25	28	17	14	5	3	3	3	3	4
21	21	15	15	3	3	3	3	3	3
21	27	17	11	5	3	3	5	3	5
21	21	15	15	2	3	3	4	3	3
26	26	14	17	2	2	3	4	4	4
21	22	14	15	4	3	3	3	3	3
28	29	12	20	2	2	4	3	4	4
21	24	13	15	3	3	4	2	3	4
26	27	9	20	3	1	2	2	4	3
31	34	20	22	5	2	5	3	5	3
22	25	12	16	4	2	3	2	4	3
35	35	16	25	5	5	5	5	5	1
31	31	21	21	3	3	3	3	3	3
35	35	7	25	4	1	5	2	5	3
34	33	15	23	3	2	4	3	5	5
21	22	17	16	4	4	3	4	4	4
35	35	12	25	4	2	5	2	5	2
21	21	14	16	4	3	4	4	3	3
27	24	16	18	4	4	4	4	4	4

29	29	11	21	2	2	4	1	5	3
32	34	13	23	5	3	4	2	5	1
25	29	18	17	4	3	3	2	3	2
23	24	13	16	3	2	4	3	3	2
35	34	13	25	5	3	5	1	5	5
32	33	20	21	5	2	4	1	3	3
33	34	16	22	4	2	4	2	5	1
21	21	15	15	3	3	3	3	3	3
21	27	17	11	4	4	3	4	3	4
26	30	21	22	5	1	5	1	5	5
35	35	14	25	3	1	4	1	5	4
29	30	15	15	4	3	3	2	4	4
25	32	14	18	4	4	1	4	2	2
30	34	19	19	4	2	4	3	4	4
28	32	9	21	4	3	4	3	4	4
21	21	15	15	3	3	3	3	3	3
35	35	13	25	1	1	5	1	5	5
21	21	15	15	3	3	3	3	3	3
29	29	12	21	1	1	4	2	4	4
29	29	19	22	5	5	3	5	4	5
24	25	19	17	4	4	4	4	4	5
28	31	17	18	5	2	4	5	4	5
27	28	13	15	5	3	2	4	4	5
21	21	15	15	3	3	3	3	3	3
23	24	17	15	4	4	3	4	3	3
28	28	18	20	4	3	3	4	3	4
35	35	10	25	1	1	5	1	5	5
26	30	14	21	4	2	4	4	5	4
23	23	17	15	5	1	3	1	3	5
28	31	17	18	5	2	2	4	2	5
25	30	10	18	4	2	3	2	4	3
35	35	18	25	5	1	5	2	5	4
35	35	6	25	3	1	5	1	5	1
35	34	7	25	3	1	5	1	5	1
31	32	14	21	4	3	5	5	5	1
30	32	11	21	4	2	4	2	4	4
24	25	12	12	3	3	3	3	3	1
28	28	11	18	5	1	3	4	4	2
22	27	14	16	5	3	3	3	3	4
28	27	12	20	3	2	4	2	4	3
21	21	15	15	3	3	3	3	3	3
35	35	10	25	5	2	4	1	5	4
26	29	14	20	5	3	4	5	3	5
29	32	17	17	3	4	3	2	4	4
23	25	16	15	4	2	3	3	3	4
26	28	17	20	4	2	4	2	4	4
24	25	12	16	3	2	3	2	3	4
26	28	13	18	4	3	3	3	4	4
26	28	12	17	4	1	4	2	4	4
27	28	11	19	2	2	4	3	4	4
27	27	14	16	3	4	3	4	4	5
35	35	12	25	1	1	5	1	5	3
27	29	10	20	2	2	4	3	4	4
27	27	18	20	4	3	4	4	4	4
25	26	9	19	3	1	3	2	3	4
26	29	16	17	3	1	3	1	4	4

27	29	16	19	4	3	4	3	4	4
23	24	14	15	3	2	3	3	3	3
32	32	14	23	1	1	4	1	4	5
23	24	16	13	4	4	3	3	3	4
30	32	19	24	3	3	4	2	5	4
34	35	11	25	1	1	5	1	5	5
27	29	15	17	4	2	3	2	4	4
30	33	17	22	5	2	3	1	4	5
32	34	11	23	4	1	4	1	4	4
19	25	12	17	4	2	4	2	4	2
31	33	12	24	2	2	4	2	5	4
34	34	13	22	4	2	4	2	5	4
28	28	10	20	2	2	4	1	4	1
28	29	17	19	1	1	4	1	4	5
30	30	10	21	4	2	4	2	4	4
32	32	7	19	4	1	4	1	4	5
33	35	11	25	1	1	5	1	5	3
21	21	15	15	3	3	3	3	3	3
22	23	12	17	3	2	3	2	4	3
31	34	9	18	4	3	3	4	3	3
30	33	16	21	3	2	4	2	4	5
29	32	16	18	5	2	4	2	4	5
33	35	14	24	5	3	4	2	5	4
27	28	13	18	3	2	4	2	4	3
32	33	10	23	4	1	4	1	5	5
28	28	20	20	2	2	3	3	4	4
35	35	9	25	5	1	5	1	5	5
23	25	17	17	4	2	3	4	3	4
28	28	12	20	3	2	4	2	4	3
31	32	14	19	3	2	4	3	5	3
30	31	19	20	5	1	4	3	4	5
27	28	12	20	5	4	3	4	4	4
28	30	11	20	2	1	4	1	4	4
27	29	14	17	4	2	4	3	4	3
31	32	9	23	5	1	5	2	4	3
35	31	10	25	5	2	3	2	5	5
24	29	16	15	4	3	3	2	3	3
35	35	8	25	3	1	5	1	5	1
31	32	13	19	3	2	4	4	3	3
32	35	8	23	3	1	4	1	4	1
26	29	14	18	4	3	4	4	4	2
29	31	9	18	1	1	5	3	5	1
35	35	9	25	5	1	5	1	5	5
27	28	14	19	4	3	4	3	3	4
30	31	19	21	4	3	4	3	4	1
35	35	18	19	5	2	4	4	5	5
30	29	14	21	3	2	4	5	4	4
22	24	12	14	4	4	3	4	4	4
21	21	10	14	4	3	3	3	3	3
35	35	17	23	3	3	3	3	3	3
28	32	19	20	5	4	4	4	5	5
28	28	12	21	3	2	4	2	4	3
33	34	9	21	1	1	4	1	5	5
32	32	17	20	3	1	4	1	4	4
28	28	18	20	4	2	4	2	4	4
27	31	11	19	3	2	3	2	3	3

35	35	5	25	3	4	5	2	5	5
28	28	10	20	3	2	4	3	4	4
34	34	10	25	4	1	5	4	5	4
27	28	15	21	3	3	4	3	4	2
35	35	8	25	1	1	5	1	5	5
28	28	18	20	2	2	4	2	4	2
21	21	15	15	3	3	3	3	3	3
20	23	16	14	5	4	2	4	3	4
25	25	16	16	4	3	2	4	3	4
35	35	14	25	1	1	5	1	5	1
21	22	17	16	4	2	4	3	4	3
33	33	9	24	4	1	4	1	4	1
31	33	21	22	5	5	3	3	4	5
31	33	13	21	5	1	4	1	5	1
31	31	21	22	4	4	5	4	4	4
35	35	25	25	5	5	5	5	5	5
35	35	21	23	5	1	5	3	5	5
21	21	15	15	3	3	3	3	3	3
21	23	14	15	4	4	3	3	3	3
28	29	12	20	4	2	4	2	4	4
29	30	20	21	4	1	4	4	4	4
21	26	15	19	4	2	4	2	4	4
26	27	15	18	3	3	3	2	4	4
34	34	14	22	4	1	4	5	5	5
29	29	12	20	4	2	4	2	4	2
25	26	9	18	3	3	3	3	3	3
27	26	17	19	3	3	4	3	4	4
24	25	19	19	4	3	3	4	4	4
28	28	12	20	3	1	4	2	4	4
34	34	9	23	1	1	4	2	4	2
26	26	7	19	2	2	4	2	4	1
27	27	13	19	3	2	4	2	4	4
35	33	5	25	1	1	5	1	5	1
21	25	16	17	4	4	3	4	3	4
34	33	12	24	5	2	5	1	5	5
32	34	10	25	3	1	4	2	5	5
35	35	10	25	1	1	5	1	5	5
26	28	9	19	3	2	4	2	4	3
31	31	6	20	3	2	4	1	4	3
31	31	6	20	3	2	4	1	4	3
26	32	9	20	2	2	4	1	5	2
35	35	13	25	1	1	5	1	5	1
26	27	17	17	4	2	3	3	3	2
35	35	9	25	1	1	5	1	5	1
31	31	18	23	4	5	4	4	4	4
33	33	9	23	4	3	3	2	5	1
35	35	25	25	5	5	5	5	5	5
34	35	10	25	2	2	4	1	5	3
29	29	13	18	2	2	3	4	5	4
30	32	7	20	2	2	4	1	5	3
25	28	17	17	4	3	4	3	4	5
7	13	7	11	2	1	4	3	1	1
25	25	11	15	4	2	3	3	4	4
29	30	11	21	4	1	4	1	4	1
21	21	15	15	3	3	3	3	3	3
33	35	13	24	4	1	4	1	4	1

35	35	9	25	1	5	5	1	5	5
28	28	12	20	1	1	4	2	4	3
22	28	18	15	5	3	3	3	4	4
28	28	14	20	4	1	4	1	4	3
28	28	14	20	4	4	4	2	4	4
22	25	15	16	4	3	3	3	4	4
28	28	13	20	3	2	4	2	4	3
21	21	15	15	3	3	3	3	3	3
28	28	11	19	3	2	4	2	4	3
21	21	15	15	3	3	3	3	3	3
28	28	12	20	4	2	3	2	4	4
35	35	20	25	1	1	5	1	5	5
27	28	20	19	2	2	3	3	2	2
28	28	20	20	4	4	4	4	4	4
33	32	14	25	1	1	5	1	5	3
30	30	10	18	2	2	4	2	4	2
35	35	9	25	1	1	5	1	5	1
35	35	12	25	4	1	4	1	5	1
22	24	13	17	4	3	3	3	3	3
35	35	7	24	4	1	5	2	5	1
34	35	17	22	4	2	4	3	5	3
28	28	14	20	3	2	4	2	4	4
27	28	15	20	4	2	4	2	4	2
27	31	14	22	3	3	4	3	3	3
35	31	9	25	1	1	5	1	1	1
29	29	11	20	4	2	4	2	4	1
28	31	8	20	4	2	3	4	4	3
32	32	12	19	2	1	3	3	4	4
31	33	11	19	1	1	5	3	5	3
26	26	13	16	4	2	3	3	4	2
24	28	13	16	4	2	4	4	4	3
21	26	19	15	4	2	2	4	3	4
27	30	12	18	3	1	4	2	4	3
28	28	9	20	1	1	4	1	4	3
21	21	12	15	3	2	3	2	3	3
30	29	16	22	4	3	5	1	5	5
31	35	19	25	3	3	3	3	5	3
27	28	16	20	4	2	4	3	4	3
26	25	12	19	4	3	3	2	4	3
28	31	17	21	3	3	4	1	4	3
32	32	15	21	4	2	4	4	4	3
31	31	16	19	3	3	3	3	5	3
30	32	18	22	4	1	4	4	5	5
26	28	15	18	4	2	3	3	4	4
32	32	9	25	5	1	5	5	5	5
35	35	15	24	2	1	4	2	5	5
28	32	13	20	3	2	4	1	4	2
20	25	14	16	3	2	3	4	3	4
35	33	9	24	1	1	5	1	5	1
27	28	13	20	3	3	4	2	4	2
27	32	16	20	4	1	4	3	4	3
30	30	10	18	2	2	4	2	4	2
35	35	25	25	5	5	5	5	5	5
28	26	16	17	4	3	3	4	4	4
35	35	10	25	2	2	5	2	5	2
21	21	15	15	3	3	3	3	3	3

21	23	13	15	1	4	3	3	3	3
33	35	12	25	5	1	5	3	5	5
35	35	5	25	1	1	5	1	5	1
35	35	16	24	4	1	5	1	5	2
23	23	15	15	3	2	3	3	4	3
27	31	13	21	3	3	4	1	4	4
27	28	13	18	3	3	3	3	3	3
24	29	13	19	4	1	4	2	4	4
33	35	5	25	5	1	5	1	5	1
20	24	13	17	2	2	4	1	4	3
28	28	18	21	2	2	4	2	4	4
28	28	11	20	3	2	4	2	4	2
20	25	10	12	4	5	3	4	2	5
33	34	14	23	5	3	3	2	5	3
17	21	13	14	3	4	4	3	3	3
21	21	15	15	2	3	3	2	3	3
28	33	17	20	4	2	3	3	4	5
35	35	10	25	5	1	5	4	5	1
28	28	18	20	4	3	4	3	4	4
28	28	16	20	4	2	4	2	4	4
32	32	17	23	5	4	4	1	5	5
22	23	12	16	3	2	4	3	4	2
23	24	16	16	5	3	3	3	4	3
35	35	17	25	1	1	5	1	5	1
26	28	16	18	4	3	3	3	4	4
27	28	12	19	3	2	4	2	4	4
32	30	8	22	3	3	4	4	5	3
25	25	15	16	4	3	3	2	3	2
24	28	17	15	5	3	3	3	2	5
31	34	22	23	5	5	4	5	3	5
23	18	15	13	3	3	3	3	3	3
35	35	16	23	4	2	5	5	5	5
34	35	14	24	3	1	5	1	5	3
34	33	16	23	5	2	4	2	5	4
24	27	16	16	2	2	3	4	3	4
25	28	12	19	3	2	3	4	4	4
30	31	12	20	5	2	3	2	5	3
35	35	25	25	5	5	5	5	5	5
28	30	14	20	3	3	4	4	4	4
27	30	14	22	4	3	3	3	4	2
27	29	16	20	3	3	4	3	4	4
35	35	9	25	1	1	5	1	5	1
35	35	17	25	2	2	5	2	5	4
31	30	16	20	3	3	3	2	5	4
28	28	11	20	3	2	4	2	4	4
27	28	16	14	3	3	4	3	2	4
22	31	11	19	1	1	5	2	5	1
17	18	11	13	3	3	3	3	3	3
35	35	12	24	4	4	5	1	5	3
33	35	10	25	1	1	5	1	5	1
26	28	17	18	4	3	3	4	4	4
29	28	13	21	5	5	5	5	4	5
35	35	11	25	3	1	5	1	5	4
31	33	14	20	2	1	4	2	4	5
34	35	7	25	4	2	5	1	5	1
29	32	13	22	3	2	4	2	4	3

27	30	13	17	4	2	3	2	4	3
35	35	9	25	2	1	4	1	5	2
31	33	10	19	4	1	4	1	4	5
22	25	15	19	4	2	4	2	4	4
27	28	11	20	2	2	4	2	4	2
24	28	16	17	5	3	3	2	4	1
28	28	14	20	4	2	3	2	4	4
28	27	10	20	4	2	3	5	5	5
20	22	15	15	3	4	4	3	3	3
35	35	9	25	3	1	4	5	5	1
34	31	12	25	1	1	5	1	5	2
33	34	17	21	3	2	4	3	5	5
35	35	8	25	3	2	5	1	5	3
21	21	15	15	3	3	3	3	3	3
35	35	5	25	1	1	5	1	5	1
29	31	17	21	5	4	4	2	4	4
27	30	15	18	5	5	3	3	4	3
22	26	12	18	2	4	4	1	4	4
21	21	15	15	3	3	3	3	3	3
35	35	19	25	5	5	5	5	5	5
29	32	15	20	3	3	4	4	4	5
27	26	12	15	3	3	3	2	4	2
31	30	15	21	3	1	4	3	5	4
27	28	15	18	4	3	3	3	4	2
23	21	17	17	3	3	4	3	3	3
23	25	13	18	2	2	3	2	3	3
25	26	12	17	4	2	4	2	4	4
21	21	15	15	3	3	3	3	3	3
29	30	15	19	3	1	3	3	3	1
23	26	10	18	2	2	3	2	3	3
28	28	15	20	3	2	4	2	4	4
25	25	15	16	4	3	3	3	3	3
35	35	15	24	4	5	4	3	5	5
27	30	14	17	3	2	3	3	3	4
21	23	13	15	4	3	3	2	3	2
23	31	13	13	3	3	3	2	3	5
35	35	11	23	4	1	5	1	5	3
28	29	15	20	3	2	3	3	4	4
21	21	13	15	4	4	4	4	3	3
28	30	10	21	3	2	4	2	4	3
28	28	21	22	4	1	3	5	5	5
29	32	8	23	4	4	4	2	4	4
34	33	19	22	4	2	4	4	5	3
28	28	15	20	4	2	4	3	4	4
29	29	10	20	3	1	3	1	4	3
28	28	15	20	4	2	3	4	4	4
34	33	11	25	4	1	5	2	5	3
35	35	5	25	1	1	5	1	5	1
27	28	11	20	4	2	3	2	4	4
35	35	8	25	1	1	5	1	5	2
24	26	12	18	4	3	4	4	4	2
34	33	9	22	4	2	4	3	5	2
29	32	11	20	1	2	4	2	4	3
25	27	12	20	1	1	3	1	3	1
28	27	14	20	4	2	4	3	4	4
21	22	13	14	5	5	3	5	3	5

28	28	12	20	4	2	4	3	4	3
32	28	10	23	3	1	4	5	5	3
21	21	15	15	3	3	3	3	3	3
21	21	11	15	3	2	3	2	3	4
34	35	9	20	4	1	3	1	5	3
27	28	7	20	1	1	4	1	4	1
33	31	16	25	2	2	5	2	5	2
21	21	15	15	3	3	3	3	3	3
25	28	13	15	3	2	4	3	3	4
35	35	7	24	3	1	4	2	4	3
26	30	13	17	3	2	3	2	4	2
35	34	20	25	5	1	4	2	5	5
31	33	13	22	2	3	3	3	3	1
31	32	14	21	2	1	4	1	5	1
31	27	21	17	4	4	3	4	5	4
30	30	17	19	5	4	3	4	5	5
24	28	17	16	4	3	4	2	3	3
24	27	17	16	4	5	3	4	3	4
30	30	16	23	5	2	4	3	4	5
31	32	21	24	5	5	5	5	5	5
19	21	15	16	4	5	4	5	2	3
21	19	15	13	2	3	2	3	2	3
35	35	9	25	4	3	4	1	5	1
27	28	15	19	4	2	4	3	4	4
35	35	5	25	1	1	5	1	5	2
29	30	14	20	4	4	4	2	4	4
31	32	10	21	4	2	4	1	4	1
22	22	16	16	4	4	4	4	4	4
33	33	11	22	4	3	5	2	5	3
26	30	13	20	3	2	4	3	4	3
32	34	8	22	2	2	4	3	4	3
21	21	15	15	3	3	3	3	3	3
21	25	10	14	2	2	3	2	3	2
34	35	9	21	2	1	4	2	5	2
35	31	9	25	1	1	5	1	5	1
19	22	10	15	3	4	4	2	3	2
27	28	12	18	4	2	3	2	4	3
35	35	9	25	1	1	5	1	5	1
26	27	11	15	3	3	3	3	3	4
30	33	8	20	4	2	4	3	4	4
27	28	14	19	4	2	4	4	4	3
35	35	16	25	4	2	5	2	5	4
33	35	12	24	3	1	5	1	4	2
21	24	13	16	4	2	3	2	3	3
28	28	12	19	2	2	4	2	4	3
21	21	15	15	3	3	3	3	3	3
35	35	11	25	3	1	5	1	5	1
35	35	11	24	4	1	4	1	5	1
29	28	19	20	4	3	3	2	4	5
35	35	17	25	1	1	5	1	5	3
35	35	10	25	3	2	4	2	5	2
35	35	7	25	1	1	5	1	5	1
31	34	9	20	2	1	4	2	4	4
26	32	16	17	2	2	4	1	4	5
28	28	14	19	4	2	4	2	4	4
30	32	21	22	5	3	4	2	4	5

24	27	17	17	4	3	2	2	2	2
35	35	15	24	5	3	4	5	5	5
35	35	7	25	3	1	5	1	5	5
35	34	7	25	1	1	5	1	5	4
28	23	20	16	4	4	4	4	4	3
21	21	15	15	3	3	3	3	3	3
21	21	15	15	3	3	3	3	3	3
35	35	17	25	5	3	5	2	5	1
21	21	15	15	3	3	3	3	3	3
26	28	19	20	3	2	4	4	4	4
33	34	18	20	5	3	3	1	4	5
30	29	17	18	1	1	3	4	4	3
28	28	12	20	4	2	4	3	4	2
28	28	14	20	3	2	4	2	4	4
34	33	14	22	4	2	4	3	5	5
28	28	12	20	3	2	4	2	4	2
29	30	15	20	5	1	3	1	4	1
25	26	15	16	4	2	3	3	3	4
21	21	15	15	3	3	3	3	3	3
25	25	14	18	4	3	4	3	4	4
26	28	18	18	3	2	4	1	4	3
28	28	8	20	3	2	4	2	4	3
28	28	14	20	2	2	4	2	4	4
35	35	9	25	4	2	5	4	5	4
35	35	6	25	1	1	5	1	5	3
25	27	14	17	3	2	4	4	4	5
22	24	15	15	3	3	3	3	3	3
29	25	18	18	4	2	3	4	4	4
27	28	10	20	4	2	4	4	4	4
28	35	17	21	2	2	5	2	5	1
23	23	11	15	3	3	3	3	3	2
34	35	16	24	5	2	4	2	5	5
35	35	19	25	3	1	5	1	5	1
21	21	15	15	3	3	3	3	3	3
35	35	13	25	3	1	5	1	5	3
27	27	14	19	3	5	4	4	4	3
33	35	9	25	1	3	5	3	5	5
28	29	16	17	4	2	3	2	4	2
31	33	10	21	5	2	4	1	5	4
26	28	13	19	1	2	3	3	3	2
26	28	13	17	3	2	4	3	4	4
35	35	12	25	2	1	5	1	5	4
28	30	15	19	2	3	3	2	4	3
28	30	14	20	4	1	4	2	4	4
31	31	18	23	5	2	5	1	5	1
19	23	11	14	4	1	4	3	3	4
23	26	15	17	4	3	3	3	3	4
31	32	12	21	5	3	3	3	3	5
28	28	16	20	3	2	4	2	4	3
35	35	13	25	1	1	5	1	5	1
26	31	13	21	5	2	4	1	4	1
32	32	8	21	3	2	4	2	4	3
35	35	9	25	1	1	5	1	5	1
21	21	15	15	3	3	3	3	3	5
35	35	17	25	4	2	5	2	5	2
28	28	14	20	2	2	4	2	4	2

35	35	5	25	4	1	5	1	5	5
30	33	10	23	3	1	4	1	4	4
24	30	15	15	4	4	3	2	4	4
35	35	19	25	1	1	5	1	5	3
29	29	14	21	2	3	4	1	4	4
35	35	12	25	5	1	5	1	5	3
21	22	16	15	4	2	3	3	3	3
21	21	16	13	4	3	2	3	4	2
23	23	15	15	3	1	3	3	3	3
34	35	9	25	1	1	5	4	5	5
30	33	16	24	4	2	4	2	5	3
29	30	19	19	4	3	3	2	4	5
33	34	8	23	2	1	4	1	5	3
20	24	13	17	3	3	3	3	3	3
26	25	15	16	3	3	3	3	3	3
28	29	11	18	2	3	3	4	4	4
31	33	14	22	4	2	4	3	4	3
22	25	16	15	5	3	3	5	4	5
21	21	15	15	3	3	3	3	3	3
35	34	5	24	1	1	5	1	5	3
35	31	19	25	1	1	5	5	5	5
35	35	25	25	5	1	5	1	5	5
28	30	21	19	3	2	3	4	2	5
20	21	15	15	3	2	3	3	3	3
24	26	17	17	5	3	3	3	3	4
32	35	5	20	3	2	5	1	5	1
33	35	14	23	3	1	5	1	5	4
20	25	17	12	4	4	2	4	3	3
28	28	10	20	3	2	4	2	4	3
25	27	17	18	4	2	3	4	2	4
35	35	18	23	4	1	3	4	5	5
27	28	18	20	4	4	4	4	4	2
23	23	11	17	3	3	3	3	3	3
23	27	14	16	4	3	3	4	3	4
28	28	16	20	3	2	4	4	4	3
21	22	15	16	3	4	3	3	3	4
24	26	16	15	3	3	3	3	3	3
19	20	13	14	2	5	2	4	2	4
33	35	11	24	4	2	5	2	5	4
35	35	25	25	5	5	5	5	5	5
25	27	13	18	3	2	4	2	4	3
27	31	15	20	4	2	3	2	4	4
22	21	15	15	4	3	3	3	3	3
34	35	17	23	5	5	4	5	5	4
21	21	15	15	3	3	3	3	3	3
28	28	14	20	4	2	4	2	4	3
28	26	16	19	4	3	4	3	4	4
21	21	15	15	3	3	3	3	3	3
28	28	19	20	4	2	4	4	4	4
35	35	5	25	1	1	5	1	5	1
26	29	18	17	4	3	3	3	4	4
31	31	15	21	3	1	4	3	5	3
21	21	15	15	3	3	3	3	3	3
35	35	9	25	3	2	5	1	5	4
30	32	15	22	5	2	4	2	5	5
35	35	5	25	1	1	5	1	5	1

28	29	12	20	5	2	4	3	4	5
33	33	8	25	2	2	5	2	5	2
30	33	15	23	5	1	5	1	5	4
21	24	14	15	3	4	4	3	4	2
30	32	14	18	4	2	4	4	4	4
25	28	19	15	2	4	3	3	4	4
29	34	14	22	4	1	3	2	5	5
25	30	19	14	4	2	1	5	4	5
27	32	15	23	4	4	3	3	3	4
26	24	12	15	3	2	3	3	3	3
30	30	13	19	3	2	4	3	4	2
33	35	11	23	4	1	5	1	5	1
33	33	18	21	4	3	4	3	4	4
27	29	14	21	3	2	4	2	4	2
34	33	10	24	1	1	5	1	5	4
31	32	16	22	5	2	4	4	5	3
31	33	13	23	2	2	5	2	4	4
19	26	13	13	4	2	3	2	3	2
27	27	21	21	5	5	5	5	5	5
28	28	12	20	3	2	4	2	4	2
25	30	18	17	5	3	1	5	3	5
28	28	17	20	2	2	4	2	4	4
35	35	13	25	1	1	5	1	5	1
29	30	16	20	4	1	4	1	4	4
28	27	6	20	2	2	4	2	4	2
35	35	14	25	1	1	5	1	5	5
35	35	25	25	5	5	5	5	5	5
35	35	5	25	1	1	5	1	5	1
29	32	16	21	3	1	3	3	4	5
26	28	14	17	4	2	3	3	4	4
24	28	13	19	4	1	3	3	4	4
28	25	11	22	3	5	3	2	4	4
26	30	11	20	4	2	4	3	3	3
24	26	15	17	4	3	3	4	3	4
34	35	13	24	3	1	5	1	5	5
21	21	15	15	3	3	3	3	3	3
28	28	14	18	4	2	3	1	3	4
35	35	17	25	4	1	5	1	5	5
21	21	15	15	3	3	3	3	3	3
28	28	13	20	2	2	4	2	4	4
35	35	14	25	4	1	5	1	4	5
25	25	14	17	4	3	3	3	4	3
28	30	14	21	4	2	3	2	4	2
29	31	14	22	4	2	3	3	4	3
35	35	14	24	3	1	5	1	5	5
29	29	19	20	5	3	4	4	4	4
28	28	17	20	4	3	4	3	3	4
25	27	12	19	2	2	4	1	4	3
35	35	25	25	5	5	5	5	5	5
28	29	16	20	5	3	4	4	4	4
25	29	13	15	3	3	3	2	3	2
29	30	19	21	4	2	3	4	4	4
21	21	15	15	3	3	3	3	3	3
26	27	17	17	4	3	3	4	3	4
29	30	14	20	3	3	4	4	4	4
26	25	10	15	4	2	3	3	3	4

21	21	15	15	3	3	3	3	3	3
27	28	15	15	3	2	3	3	4	4
27	24	17	18	5	2	3	3	4	3
21	21	15	15	3	3	3	3	3	3
28	29	14	21	3	2	4	2	4	3
35	35	18	25	5	1	4	5	5	5
33	32	8	24	4	1	4	1	5	5
35	35	19	23	5	4	3	3	5	5
27	31	9	20	4	1	4	2	4	5
33	34	5	22	3	1	4	1	5	1
31	35	18	20	2	1	5	4	3	2
35	35	9	25	1	1	5	1	5	1
28	28	20	20	4	4	4	4	4	4
21	27	15	15	3	3	3	3	3	3
35	35	15	25	1	1	5	1	5	5
24	24	16	15	3	2	3	3	3	4
23	25	17	15	3	3	4	4	4	5
28	28	14	20	3	2	4	3	4	4
28	28	19	20	4	3	4	4	4	4
26	25	18	20	4	3	4	4	4	4
34	31	8	25	2	2	5	1	5	1
27	25	9	20	4	2	4	2	1	3
35	35	19	20	5	4	5	1	5	5
33	34	12	23	3	1	5	3	4	4
25	29	14	19	3	1	4	3	4	4
21	21	15	15	3	3	3	3	3	3
35	35	25	25	5	5	5	5	5	5
29	29	17	20	4	4	3	1	5	4
34	34	10	25	3	1	5	2	5	1
20	21	10	17	2	2	4	2	4	2
28	28	18	20	4	4	4	4	4	4
27	27	15	15	4	3	3	2	3	2
29	32	13	19	5	4	3	3	4	4
26	28	12	19	2	2	4	2	4	2
35	35	14	25	1	1	5	1	5	4
26	26	14	18	3	3	3	3	4	4
35	35	16	25	3	3	4	1	5	3
27	30	17	19	5	2	4	4	4	4
35	35	5	25	1	1	5	1	5	4
31	32	17	21	2	1	4	3	4	5
35	35	13	25	5	2	5	3	5	4
28	33	10	21	1	3	4	2	4	5
34	35	25	25	5	1	5	3	5	5
28	28	20	20	4	4	4	4	4	4
32	33	15	22	3	2	4	2	4	3
29	31	21	22	4	5	4	1	4	5
29	28	15	20	2	2	4	3	4	4
20	22	17	14	5	4	3	5	3	4
21	21	15	15	3	3	3	3	3	3
24	28	16	18	3	5	4	4	3	4
21	21	15	15	3	3	3	3	3	3
22	22	15	16	3	3	3	3	3	3
20	21	9	14	4	2	3	2	3	3
25	25	18	18	3	4	3	2	3	3
35	35	5	25	1	1	5	1	5	1
25	30	19	21	5	2	3	4	5	5

30	29	18	21	4	1	4	2	5	2
28	28	14	19	4	2	3	4	4	3
22	28	15	18	2	2	3	3	5	5
35	35	14	24	5	1	5	4	5	4
28	28	12	20	2	2	4	2	4	3
31	31	15	24	5	2	5	2	5	3
31	33	15	23	3	2	4	2	5	4
32	35	11	25	3	2	3	2	4	5
34	32	14	23	5	1	4	1	5	5
30	33	13	24	1	1	4	3	5	5
29	30	7	20	1	1	4	1	4	2
29	29	13	21	4	2	4	2	4	2
25	29	9	19	3	2	4	2	3	2
35	35	5	25	2	1	5	1	5	1
22	22	16	16	4	4	3	3	3	4
27	27	13	17	2	2	3	4	4	4
24	28	15	17	4	1	4	2	4	4
28	29	12	19	2	2	4	2	4	4
31	31	15	23	4	3	4	3	4	3
28	28	18	19	4	2	4	3	4	2
33	35	25	25	5	5	5	5	5	5
35	35	8	25	3	1	5	1	5	1
25	31	17	23	5	2	5	3	5	4
34	35	16	22	4	1	3	3	4	4
21	21	15	15	3	3	3	3	3	3
35	35	7	25	5	1	5	1	5	3
28	34	13	21	1	1	3	1	4	1
21	26	12	15	4	2	3	3	3	3
35	35	5	25	1	1	5	1	5	3
25	29	13	17	4	3	3	3	3	3
35	35	5	25	1	1	5	1	5	2
17	28	18	12	3	3	2	2	2	3
24	27	11	17	3	3	3	4	4	4
24	23	15	15	3	2	3	3	3	4
23	23	15	15	4	4	3	4	3	4
26	28	14	12	3	2	2	3	3	2
23	27	18	15	4	3	2	4	3	3
35	35	7	25	5	1	5	1	5	5
28	28	12	20	2	2	4	2	4	3
25	25	17	19	3	4	3	4	4	4
31	31	17	18	3	1	5	1	5	5
27	27	16	16	4	3	4	4	4	3
28	27	17	20	1	1	5	1	3	3
28	28	8	20	1	1	4	1	4	1
15	20	18	12	5	4	2	4	2	4
25	27	12	15	2	3	2	3	2	4
35	35	9	25	1	1	5	1	5	1
27	29	15	17	3	2	3	3	4	2
35	35	18	25	1	1	5	1	5	5
31	31	5	25	1	1	5	1	5	1
28	29	16	20	4	2	4	3	3	4
28	31	15	18	4	2	3	2	4	2
21	21	15	15	3	2	3	3	3	3
28	28	10	18	4	1	3	2	4	3
27	27	21	17	4	2	4	4	3	4
23	22	16	15	3	3	3	3	3	3

25	25	14	18	3	3	3	3	3	3
35	35	6	25	1	1	5	1	5	3
35	35	17	21	5	1	4	4	5	3
31	33	12	23	2	3	4	4	4	4
25	26	16	16	4	3	4	2	3	4
24	25	15	16	3	2	4	4	4	2
29	31	13	22	5	3	4	4	4	2
24	25	13	15	4	3	4	4	3	2
21	21	15	15	3	3	3	3	3	3
33	34	15	24	5	4	5	2	5	5
31	33	12	23	1	1	4	2	4	3
24	23	15	15	4	3	4	3	3	3
26	27	15	17	4	2	3	2	4	4
25	29	18	18	4	3	3	3	4	3
32	32	11	25	1	2	5	1	5	3
23	20	17	17	4	2	4	4	2	4
23	24	16	14	2	3	3	4	2	4
35	35	20	25	3	3	5	3	5	3
28	28	10	20	3	2	4	2	4	2
21	21	13	15	3	3	3	3	3	3
28	28	12	20	4	2	4	3	4	3
20	22	11	12	5	3	2	2	3	2
35	35	5	25	4	1	5	1	5	4
35	35	9	25	1	1	5	1	5	5
28	28	20	20	4	4	4	4	4	4
25	26	13	17	2	3	3	2	2	2
23	25	12	17	3	2	4	3	3	3
30	31	14	19	4	3	4	3	5	3
31	34	12	24	1	1	5	1	5	1
22	26	14	15	2	3	3	2	3	4
28	32	16	22	3	4	4	2	4	5
29	32	23	20	4	2	3	3	5	5
27	28	13	17	5	1	5	1	3	3
23	23	11	16	4	2	3	2	4	3
35	35	5	25	5	1	5	1	5	1
29	30	19	21	3	3	3	2	4	4
24	24	14	14	4	3	3	2	3	2
21	22	10	15	4	2	3	2	3	2
35	35	5	25	5	5	5	5	5	5
23	26	12	19	4	3	4	3	4	3
27	27	13	17	4	4	3	4	4	4
21	24	14	15	3	3	3	3	3	3
31	35	9	23	1	1	5	1	5	5
31	30	17	24	4	1	4	2	4	5
35	35	11	25	1	1	5	1	5	5
27	29	20	20	5	5	2	4	4	4
27	28	17	20	3	1	4	2	4	3
22	26	16	17	4	2	3	4	4	3
26	29	21	20	5	2	3	3	3	4
28	28	18	18	5	3	4	4	4	2
27	27	14	18	5	1	2	1	3	3
35	35	25	25	5	5	5	5	5	5
23	25	13	16	3	2	3	3	3	3
21	21	15	15	3	3	3	3	3	3
28	28	13	20	2	2	4	2	4	2
29	34	17	16	5	5	5	5	5	5

31	33	17	24	3	3	3	3	3	3
35	35	17	25	5	1	5	1	5	5
32	34	8	22	2	1	4	1	4	3
21	21	15	15	3	3	3	3	3	3
23	24	14	16	4	3	3	3	3	3
28	28	13	20	4	2	4	3	4	4
25	27	17	18	4	3	4	4	4	4
27	28	14	20	4	4	4	4	4	4
22	23	10	16	3	2	3	2	3	4
28	29	16	20	4	2	4	4	5	4
35	34	11	22	1	1	5	2	5	4
25	26	11	17	3	2	4	3	4	3
35	35	5	25	1	1	5	1	5	5
35	35	9	25	4	1	5	1	5	1
28	28	14	19	4	2	4	4	4	4
24	26	12	17	4	3	4	3	4	2
21	21	15	15	3	3	3	3	3	3
29	31	13	19	3	1	4	3	4	1
34	34	11	25	2	1	5	2	5	1
32	32	9	24	5	4	4	2	5	1
35	35	5	25	1	1	5	1	5	1
22	27	13	14	5	4	2	4	3	3
23	23	15	15	3	3	3	3	3	3
21	21	15	15	3	3	3	3	3	3
27	28	12	20	2	2	4	2	4	3
21	21	15	15	3	2	3	2	3	2
31	33	17	25	4	5	5	2	5	5
32	33	6	21	2	1	5	1	3	2
35	35	13	25	1	1	5	1	5	3
24	24	18	17	5	5	1	5	1	5
35	35	7	25	3	1	3	3	5	1
35	35	5	25	1	1	5	3	5	1
32	33	9	19	2	1	4	2	5	3
35	35	10	25	2	2	4	2	5	3
33	34	18	22	3	1	5	5	5	4
27	31	9	16	3	3	3	2	4	3
21	21	15	15	3	3	3	3	3	3
30	29	21	24	4	3	4	5	4	5
26	20	15	14	3	2	4	4	3	3
24	28	19	21	4	3	4	3	3	4
27	30	13	16	3	3	3	3	4	4
21	21	15	15	3	3	3	3	3	3
34	33	13	23	1	1	4	4	4	4
28	26	15	19	3	2	4	3	3	4
35	35	5	25	1	1	5	1	5	1
28	29	13	13	3	5	3	4	3	2
29	32	10	20	4	2	4	2	5	4
35	35	25	25	5	5	5	5	5	5
21	21	15	15	3	3	3	3	3	3
20	21	14	14	4	3	3	3	2	2
29	30	15	18	5	4	2	5	3	5
33	34	5	25	1	1	5	1	5	1
27	28	12	20	4	2	4	2	4	2
28	28	12	20	4	2	4	2	4	2
21	22	13	15	3	2	3	3	3	3
24	27	14	20	3	2	4	2	4	3

31	31	9	22	4	1	3	2	4	4
29	29	14	19	3	2	3	3	5	2
21	21	15	12	4	2	3	4	4	4
35	35	21	25	1	5	5	1	5	5
25	26	12	17	2	2	4	3	4	4
32	33	16	23	3	3	3	1	5	3
22	23	15	15	4	3	3	4	4	4
23	27	13	15	4	2	4	3	3	4
35	35	7	25	1	1	5	1	5	1
34	35	13	25	5	1	3	3	5	4
33	35	14	21	4	2	4	4	4	5
33	32	13	24	4	1	4	2	5	4
27	28	15	17	2	2	3	4	3	5
35	35	17	25	1	1	5	4	5	5
34	33	7	25	4	2	5	2	5	2
32	33	24	23	2	3	4	3	4	4
22	23	16	16	5	4	3	3	2	4
23	23	14	16	3	2	3	3	3	2
31	32	13	21	3	3	5	3	4	4
29	29	8	20	2	2	3	2	4	3
22	21	14	16	4	3	3	3	4	3
28	31	13	21	3	1	4	2	4	4
35	35	10	25	3	1	5	1	5	4
21	23	13	15	3	4	3	3	3	4
25	26	15	18	4	2	3	2	3	2
35	35	10	25	1	1	5	1	5	1
27	30	22	23	5	4	5	4	5	4
32	30	13	23	2	2	5	4	5	4
32	33	12	23	4	1	4	1	4	4
29	29	18	20	5	5	3	4	4	5
24	27	16	17	4	1	4	1	4	5
30	34	10	21	3	2	4	2	4	3
35	35	9	25	5	1	5	1	5	1
32	34	16	20	4	3	4	4	4	3
24	27	16	16	4	3	3	3	4	4
23	23	15	15	5	3	3	5	5	4
34	32	9	23	3	2	4	1	5	1
23	24	9	17	3	2	3	2	3	4
35	35	11	24	4	2	4	3	5	2
30	32	8	19	1	3	3	1	4	4
26	28	17	19	4	2	4	4	4	3
26	27	18	19	3	3	3	3	4	3
27	28	10	18	4	2	3	4	4	1
23	28	15	16	3	2	4	3	5	5
35	35	9	25	1	1	5	1	5	1
27	27	13	19	4	2	2	3	4	4
22	25	16	21	4	3	3	4	3	4
24	25	14	16	2	2	2	2	3	2
28	28	14	20	4	3	3	2	4	2

a7	a8	a9	a10	a11	a12	a13	a14	a15	a16	a17	a18	a19	a20
5	5	1	5	1	1	5	1	5	1	5	5	5	5
4	5	1	4	4	2	3	3	4	4	4	5	2	3
5	5	3	5	2	1	4	1	5	2	5	5	1	5
4	4	3	5	2	2	3	1	4	1	4	5	1	1
3	4	4	3	4	4	4	3	4	4	4	4	3	4
3	4	3	3	2	4	2	2	4	2	1	4	2	3
4	4	3	3	3	3	3	3	3	3	3	3	2	4
4	4	4	4	2	4	2	2	4	4	4	4	2	4
3	3	3	3	3	3	3	3	3	3	3	3	3	3
4	4	4	4	4	4	4	4	4	4	4	4	3	4
4	4	1	5	3	2	3	1	3	4	4	4	2	4
3	3	3	3	3	3	3	3	3	3	3	3	3	3
3	4	2	4	4	3	3	2	2	2	4	4	3	3
4	4	3	4	4	3	3	3	4	3	4	4	3	3
5	5	1	5	1	1	3	1	5	4	5	5	2	5
3	3	3	3	3	3	3	3	3	2	4	4	2	4
3	4	3	3	3	3	3	3	3	4	4	5	3	3
4	4	4	4	4	3	4	3	5	4	5	5	4	4
3	5	5	3	4	3	2	2	5	3	5	5	2	5
3	3	2	3	3	2	3	3	2	3	4	4	3	3
4	4	3	4	4	3	2	3	4	3	3	4	4	4
5	5	5	5	5	5	5	1	5	5	5	5	1	5
4	4	4	4	4	4	4	4	4	4	4	4	4	4
5	5	2	5	1	1	4	1	5	4	4	5	2	5
3	3	2	3	4	3	3	3	3	2	3	3	2	3
4	4	1	4	3	2	4	2	4	3	4	5	2	5
3	4	3	3	2	3	4	2	5	5	4	4	3	4
3	5	4	3	4	4	3	4	2	4	5	5	2	3
4	4	1	4	2	2	3	1	5	4	4	4	1	4
3	3	3	3	2	3	3	2	5	3	5	5	2	3
4	4	3	4	3	2	4	2	4	4	4	4	2	4
5	5	3	5	4	2	5	2	5	5	5	5	2	5
3	4	4	3	4	4	3	2	3	4	4	4	2	2
1	4	1	2	5	3	3	5	3	2	5	5	2	2
5	5	5	5	3	2	5	1	5	4	5	5	2	4
4	4	4	4	4	3	3	3	4	4	4	4	3	3
4	4	3	4	3	3	3	2	4	4	4	4	2	4
3	3	3	3	3	3	3	3	3	3	3	3	3	3
4	4	2	4	3	2	4	3	4	4	4	4	2	4
3	3	3	3	3	3	3	3	3	3	3	3	3	3
3	3	3	3	4	3	2	3	3	4	3	3	2	3
3	3	3	3	3	3	3	3	3	3	3	3	3	3
3	4	2	3	4	4	2	4	3	4	4	5	2	3
5	5	1	5	5	5	3	1	5	5	5	5	4	3
4	4	3	4	4	4	3	3	4	4	4	4	4	4
4	4	3	4	2	2	3	2	5	4	4	4	2	5
4	5	5	3	4	4	3	4	4	4	4	4	2	4
5	5	5	5	5	5	5	5	5	5	5	5	5	5
3	3	1	3	2	2	3	1	4	1	3	3	2	3
4	5	4	4	1	3	4	1	5	5	5	5	1	4

5	5	5	5	1	1	5	1	5	5	5	5	1	5
2	4	3	3	2	4	4	2	4	3	3	4	4	2
4	4	1	4	2	2	3	2	4	3	3	4	2	4
4	4	2	4	2	2	4	2	4	4	5	5	3	4
4	4	1	4	1	1	4	1	4	4	4	4	1	4
4	4	2	4	3	3	3	2	2	4	4	4	4	4
4	5	3	4	3	4	3	4	2	4	5	5	4	3
4	4	4	4	3	3	3	2	4	4	4	5	4	3
3	3	3	3	3	3	3	3	3	3	3	3	3	3
3	4	2	3	3	3	4	2	4	3	4	5	3	3
3	4	3	3	3	3	3	3	3	3	3	4	3	2
4	5	5	4	2	2	5	1	5	2	5	4	1	4
5	4	3	4	2	2	3	2	4	3	4	5	1	4
5	5	5	3	5	5	5	5	5	5	5	5	5	5
3	3	3	3	4	3	3	2	4	3	3	4	4	4
4	4	2	4	2	2	4	2	4	4	4	4	4	4
4	4	2	4	4	3	4	3	3	4	4	4	2	2
4	4	2	4	2	2	4	2	4	3	4	4	3	4
3	3	2	3	3	2	2	3	3	4	3	4	3	3
4	4	2	4	3	3	3	2	4	5	4	5	2	3
4	4	3	4	3	2	4	2	4	3	4	4	2	4
5	5	5	4	3	3	2	3	4	3	4	5	2	3
5	5	1	5	1	1	5	1	5	1	5	5	1	5
3	5	1	4	5	3	5	2	4	5	5	5	2	3
3	3	2	3	2	2	3	2	3	2	3	3	2	3
3	3	2	3	4	4	3	3	2	4	4	4	3	3
4	5	1	4	2	2	4	1	5	4	5	5	1	4
2	3	1	2	3	2	3	1	4	1	4	3	2	3
5	5	3	5	5	5	5	1	5	4	5	5	1	5
4	4	1	3	2	2	3	2	3	3	4	5	2	3
4	4	3	4	3	4	3	3	4	4	4	4	3	3
4	4	2	4	2	2	4	3	4	4	4	4	2	5
3	3	3	3	4	4	3	3	4	4	4	4	4	3
5	5	1	3	3	3	3	3	3	3	4	5	4	3
5	5	4	5	1	1	5	1	5	1	5	5	1	5
3	3	3	3	3	3	3	3	3	3	3	3	3	3
5	5	4	5	4	4	4	4	4	5	5	5	5	4
4	4	2	3	3	2	4	2	3	3	4	4	2	4
3	3	3	3	3	2	3	2	3	3	3	3	3	3
5	5	2	5	1	1	5	1	5	1	5	5	1	5
3	4	1	3	3	4	2	3	3	4	4	4	2	3
5	5	1	5	1	1	3	1	5	2	5	5	3	4
4	4	3	4	2	2	4	2	4	4	4	5	2	4
5	5	3	5	5	2	4	2	3	4	5	5	1	4
5	5	1	5	1	1	5	1	5	1	5	5	1	5
5	5	3	5	2	2	5	1	5	3	5	5	2	5
3	3	3	3	2	2	3	2	3	3	3	3	3	3
4	4	2	4	2	3	4	3	3	3	4	4	4	3
3	3	2	3	4	4	3	3	3	3	4	4	3	3
3	3	3	3	3	3	3	2	3	3	3	3	2	3
4	3	2	4	3	1	4	1	4	3	4	4	1	3
4	4	3	5	4	2	4	2	4	4	4	5	2	4
4	4	4	4	3	3	2	3	4	4	4	4	3	4
5	5	3	5	4	3	4	1	5	5	5	5	3	5
4	4	5	4	5	2	5	2	5	4	5	5	2	4
5	5	1	5	1	1	5	1	5	4	5	5	2	5

5	5	1	5	1	1	5	1	1	1	5	5	1	5
3	3	2	3	3	3	2	3	2	3	3	3	3	3
3	3	1	3	4	4	4	3	3	4	5	5	3	4
5	5	2	5	1	1	5	1	5	2	5	5	2	5
2	3	3	2	2	3	4	2	4	3	3	4	2	3
3	3	3	4	3	3	3	3	3	3	3	3	3	3
5	5	2	4	2	1	4	1	4	2	4	5	2	5
4	4	3	4	2	2	4	3	5	4	5	5	2	5
4	3	2	4	2	4	2	4	3	4	3	4	2	4
3	4	2	3	2	2	4	2	4	3	3	4	2	4
5	5	5	5	3	3	4	2	5	5	5	5	1	4
3	4	2	3	2	3	3	3	2	4	4	4	2	3
4	4	3	4	4	3	3	3	3	3	3	4	3	3
3	5	3	4	3	3	2	2	4	5	5	5	3	4
5	5	4	5	4	4	3	3	3	3	5	5	4	3
2	2	2	3	3	3	2	3	3	3	3	4	4	3
5	5	5	5	5	5	5	5	5	5	5	5	5	5
4	4	2	3	4	4	3	3	2	4	5	5	3	3
5	5	1	5	1	1	5	1	5	5	5	5	1	5
3	5	1	5	4	3	2	2	2	5	5	5	3	5
4	4	3	3	3	3	4	2	4	3	5	5	3	4
3	2	1	3	4	3	3	2	3	4	3	3	3	4
4	4	3	4	4	4	2	2	4	4	4	4	1	4
4	4	2	4	4	3	4	2	4	4	4	4	2	4
3	3	2	3	4	3	3	2	3	3	4	4	3	3
4	4	2	4	2	2	4	2	4	2	4	4	3	4
5	5	2	4	2	1	4	1	5	2	5	5	1	4
4	4	3	4	3	3	3	2	4	4	4	4	3	4
5	5	5	5	5	5	5	1	1	1	5	5	5	3
1	4	4	1	3	1	3	2	4	5	2	5	2	4
3	3	3	3	3	3	3	3	3	3	3	3	3	3
3	4	4	3	5	3	3	3	2	4	4	5	4	3
4	3	2	3	3	3	4	2	3	4	2	3	3	3
1	1	1	3	4	3	1	5	1	5	5	5	5	2
3	4	2	3	2	2	4	2	4	4	4	4	2	4
4	4	3	4	2	2	4	2	5	4	5	5	2	5
4	5	1	4	2	3	3	1	5	1	5	5	1	3
2	4	3	2	4	4	4	2	3	4	4	5	3	3
3	3	3	3	3	3	3	3	3	3	3	3	3	3
3	5	3	3	3	5	1	3	5	5	3	5	3	3
3	3	3	3	3	3	3	3	3	3	3	3	3	3
4	4	3	4	2	2	3	3	2	4	4	5	2	3
3	3	2	3	3	3	3	3	3	3	3	3	3	3
4	4	2	4	2	2	4	2	4	4	4	4	2	4
2	3	3	2	3	2	3	3	3	2	3	4	2	4
3	4	1	4	1	1	3	1	3	3	4	5	2	4
4	5	5	4	4	4	4	2	5	3	5	5	5	5
3	3	2	3	2	2	3	2	3	3	3	4	2	3
5	5	2	5	2	3	5	3	5	3	5	5	3	5
3	3	3	3	3	3	3	3	3	3	3	3	3	3
5	5	1	5	1	2	5	1	5	4	5	5	1	5
4	4	3	5	3	3	4	1	5	5	5	5	4	4
3	4	4	3	3	3	3	3	3	3	3	3	3	3
5	5	4	5	3	2	4	1	5	3	5	5	1	5
3	3	3	3	3	3	3	4	3	3	3	3	3	3
4	4	4	4	3	4	3	4	4	4	3	4	3	4

5	5	2	5	2	1	4	1	4	3	4	4	2	4
4	5	4	5	2	1	4	1	4	3	5	5	2	5
3	4	4	3	3	3	3	2	4	3	4	5	3	3
3	3	3	3	4	3	3	4	2	3	4	3	2	3
5	5	1	5	5	2	5	5	5	5	5	5	2	5
4	4	3	4	2	2	3	1	5	5	5	5	5	5
5	5	5	5	4	3	4	2	4	5	5	5	3	4
3	3	3	3	3	3	3	3	3	3	3	3	3	3
2	4	4	2	4	4	3	3	3	4	4	5	4	2
5	4	5	5	2	1	1	1	1	1	1	5	5	5
5	5	3	5	1	3	5	1	5	4	5	5	2	5
4	4	3	4	4	3	3	2	4	5	5	5	3	2
2	3	1	3	4	5	1	3	5	2	3	5	3	2
4	5	4	3	4	2	4	3	4	5	5	5	4	4
4	5	1	5	3	3	3	3	4	5	5	5	1	4
3	3	3	3	3	3	3	3	3	3	3	3	3	3
5	5	5	5	1	1	5	1	5	5	5	5	5	5
3	3	3	3	3	3	3	3	3	3	3	3	3	3
4	4	2	4	2	2	4	2	2	4	4	4	2	4
3	3	3	4	5	5	3	5	4	5	5	5	5	4
3	5	4	3	4	4	3	4	4	3	4	4	3	5
4	4	4	4	4	3	3	3	4	5	4	5	3	3
3	4	1	3	4	4	3	4	3	4	5	5	3	3
3	3	3	3	3	3	3	3	3	3	3	3	3	3
3	4	3	3	4	4	3	3	3	3	4	4	4	3
4	4	3	4	4	3	3	4	4	4	4	4	4	4
5	5	3	5	2	1	5	1	5	4	5	5	1	5
4	5	3	4	3	3	4	2	3	4	3	4	3	5
3	3	3	3	3	3	3	3	3	3	5	5	5	3
2	5	5	4	4	4	3	3	5	5	5	5	3	3
3	4	1	3	2	2	3	1	4	4	5	5	1	4
5	5	5	5	3	2	4	1	5	5	5	5	2	5
5	5	1	5	1	1	5	1	5	5	5	5	1	5
5	5	1	5	1	1	5	1	5	5	5	5	1	5
4	5	1	4	3	2	5	1	5	5	5	5	1	5
5	5	2	4	3	2	3	2	4	3	4	5	2	4
3	3	3	3	3	3	3	3	3	3	5	5	2	3
4	4	4	4	4	3	3	2	1	3	4	4	2	3
3	4	3	3	3	2	3	2	3	3	3	4	3	3
4	4	3	4	2	2	4	2	3	3	4	4	2	4
3	3	3	3	3	3	3	3	3	3	3	3	3	3
5	5	4	5	1	3	5	2	5	5	5	5	1	5
3	4	2	4	4	2	3	3	3	4	4	5	3	3
4	4	4	4	5	4	3	2	3	4	5	5	3	3
3	4	4	3	3	4	3	3	4	4	4	4	3	3
4	4	4	4	4	3	3	2	4	4	4	4	4	4
3	3	2	3	2	2	3	2	3	3	3	3	2	3
4	4	2	4	3	3	3	2	4	4	4	5	3	3
3	4	2	3	3	2	4	2	3	3	4	4	2	4
4	4	2	4	4	2	4	2	4	4	4	4	2	3
4	4	2	4	5	2	4	2	4	4	4	4	3	3
5	5	3	5	1	1	5	1	5	4	5	5	1	5
5	5	2	4	2	2	4	2	3	4	4	4	2	4
4	4	4	4	3	3	3	2	4	5	5	5	3	4
4	4	1	4	3	2	3	2	2	2	4	4	2	4
3	4	4	3	2	3	4	1	3	4	5	5	2	4

4	4	3	4	4	4	3	3	4	4	4	5	4	4
3	4	2	3	4	3	3	3	4	3	4	4	3	3
5	5	3	4	1	1	4	1	4	5	5	5	1	5
3	3	3	3	3	4	3	4	3	5	5	5	4	2
5	5	4	5	5	4	4	4	4	4	4	4	4	4
5	5	4	5	1	1	5	1	5	5	5	5	1	5
3	4	3	3	2	3	4	2	4	3	5	5	3	4
3	5	3	4	3	3	3	2	4	5	5	5	4	4
5	5	2	5	2	2	4	1	4	4	4	5	1	4
2	4	2	3	3	3	3	3	3	3	3	3	3	3
4	4	2	5	4	3	4	2	5	3	5	5	2	5
5	5	4	5	4	3	4	1	5	4	5	5	2	4
4	4	1	4	1	1	4	1	4	4	4	4	2	4
3	4	3	4	3	1	3	1	4	5	5	5	5	3
5	4	2	5	3	2	3	1	3	4	5	5	2	4
5	5	1	5	3	1	4	1	2	3	4	4	1	3
5	5	3	5	1	1	5	1	5	5	5	5	1	5
3	3	3	3	3	4	3	3	3	3	3	3	3	3
3	3	3	4	3	3	3	3	4	3	3	3	3	3
4	5	1	4	4	2	4	1	4	1	5	5	1	4
4	5	5	4	3	1	3	2	4	5	5	5	2	5
5	5	5	5	5	3	3	2	4	5	5	5	3	4
5	5	4	5	3	4	5	2	5	4	5	5	2	4
4	4	3	4	2	2	4	2	3	3	3	4	2	3
5	5	1	5	3	4	5	1	5	4	4	5	1	4
4	4	4	4	4	4	4	4	4	4	4	4	4	4
5	5	2	5	2	1	5	1	5	5	5	5	1	5
2	4	3	4	5	4	2	2	3	4	4	4	4	2
4	4	2	4	2	2	4	2	4	3	4	4	3	4
5	5	3	4	3	2	4	1	4	3	4	5	3	4
4	4	4	4	3	3	4	3	4	5	5	5	4	4
4	4	2	4	4	4	4	2	4	4	4	4	2	4
4	4	4	4	4	1	4	1	5	5	4	5	1	4
4	4	3	4	3	2	3	2	3	4	4	5	3	3
4	4	3	4	3	2	3	1	4	4	4	5	2	4
5	5	2	5	2	1	3	1	5	5	5	5	1	5
2	5	3	2	3	2	3	3	3	4	3	5	4	3
5	5	2	5	1	1	5	1	5	1	5	5	1	5
3	3	4	3	4	2	4	2	3	5	5	5	3	4
5	5	1	5	2	2	4	2	4	4	4	5	1	4
3	4	2	4	4	3	4	2	4	4	4	5	4	4
3	3	3	3	3	3	3	3	3	3	5	5	3	3
5	5	5	5	1	1	5	5	5	5	5	5	1	5
4	4	3	4	3	2	3	3	4	4	4	4	3	4
3	4	4	3	2	2	3	4	5	4	4	4	3	4
5	5	4	5	4	3	2	1	5	5	5	5	4	4
4	4	2	4	4	3	3	3	2	4	5	5	4	4
3	4	1	4	3	4	3	4	4	4	3	4	3	3
3	3	2	2	3	2	2	3	3	3	3	4	2	3
5	5	5	5	1	1	5	1	5	1	5	5	1	3
3	5	3	3	5	5	3	4	5	5	5	5	5	4
4	4	2	4	2	3	4	2	4	3	4	4	3	5
5	5	1	5	4	1	3	1	5	5	5	5	2	4
5	5	4	4	3	2	4	2	4	4	5	5	3	4
4	4	3	4	2	2	4	2	4	4	4	4	4	4
3	4	1	3	2	3	3	3	3	3	4	5	2	3

5	5	1	5	3	2	5	3	4	4	5	5	1	5
4	4	2	4	2	2	3	1	3	4	4	4	2	4
5	5	1	5	3	2	3	2	4	4	5	5	2	5
4	4	4	5	2	2	3	3	3	4	4	5	2	4
5	5	1	5	1	1	5	1	5	1	5	5	1	5
4	4	4	4	2	2	4	2	4	4	4	4	4	4
3	3	3	3	3	3	3	3	3	3	3	3	3	3
2	3	2	4	4	4	3	2	4	2	3	3	4	3
3	3	3	3	4	3	2	4	3	4	4	4	4	4
5	5	2	5	1	1	5	1	5	5	5	5	5	5
3	4	4	4	4	3	4	3	3	4	3	3	4	3
4	4	1	4	1	1	4	1	5	4	5	5	1	5
3	4	4	5	5	2	3	3	4	4	5	5	4	3
5	5	2	3	2	3	3	1	3	4	5	5	1	5
5	4	4	4	4	5	4	4	5	4	5	4	4	5
5	5	5	5	5	5	5	5	5	5	5	5	5	5
5	5	4	5	5	2	4	5	5	5	5	5	5	5
3	3	3	3	3	3	3	3	3	3	3	3	3	3
3	4	3	3	3	3	3	3	4	3	3	4	3	3
4	4	1	4	2	2	4	2	2	4	4	4	2	4
4	4	3	4	4	3	3	2	4	4	4	5	5	4
4	4	4	4	2	1	4	2	4	4	4	5	3	4
4	4	4	4	2	4	3	2	4	4	4	4	3	4
5	5	2	4	3	3	2	2	2	5	5	5	5	5
4	4	4	4	2	2	4	2	4	2	4	5	2	4
3	3	1	3	3	3	3	3	3	3	3	3	3	3
4	4	3	4	3	4	3	2	3	4	4	4	3	4
4	4	4	4	4	4	4	4	4	4	4	4	4	4
4	4	2	4	4	2	4	2	2	4	4	4	2	4
5	5	1	5	2	2	4	2	4	4	4	4	2	4
4	4	1	4	1	1	4	1	4	3	3	3	2	4
4	4	3	4	4	3	4	2	3	3	3	4	2	3
5	5	1	5	1	1	5	1	5	1	5	5	1	5
3	4	3	3	5	3	3	3	3	3	2	3	4	3
5	5	3	5	4	2	5	2	5	5	5	5	3	4
4	5	3	5	4	3	4	1	5	5	5	5	1	5
5	5	3	5	1	1	5	1	1	5	5	5	1	5
4	4	2	4	3	2	2	3	4	4	4	4	2	4
4	4	1	4	1	1	4	1	4	5	5	5	1	4
4	4	1	4	1	1	4	1	4	5	5	5	1	4
4	5	2	4	2	2	3	1	4	3	4	4	2	3
5	5	5	5	1	1	5	1	5	1	5	5	1	5
3	3	3	3	4	4	3	4	3	4	4	4	3	3
5	5	1	5	1	1	5	1	5	1	5	5	1	5
4	5	5	5	5	5	4	2	5	5	5	5	3	4
5	5	1	5	2	1	3	1	5	4	4	5	1	4
5	5	5	5	5	5	5	5	5	5	5	5	5	5
5	5	2	5	1	1	4	1	5	4	5	5	1	5
4	4	3	4	3	3	3	3	3	5	5	5	3	4
4	4	1	5	2	1	3	1	4	4	4	4	1	4
3	5	5	4	5	3	3	3	3	3	5	5	3	4
1	1	1	1	1	1	3	1	4	1	1	1	1	4
4	4	2	3	2	3	2	3	3	4	4	4	2	3
4	4	4	4	4	2	3	2	5	4	4	4	1	4
3	3	3	3	3	3	3	3	3	3	3	3	3	3
5	5	3	5	1	3	3	1	5	4	3	5	1	5

5	5	1	5	1	1	5	1	5	5	5	5	1	5
4	4	3	4	2	2	3	2	4	4	4	4	2	4
2	5	3	3	3	3	3	3	3	3	5	5	5	2
4	4	3	4	1	1	4	1	4	3	4	4	1	4
4	4	3	4	3	3	4	3	4	4	4	4	3	4
3	3	3	3	3	3	3	3	3	3	4	4	3	3
4	4	2	4	3	3	4	2	4	4	4	4	2	4
3	3	3	3	3	3	3	3	3	3	3	3	3	3
4	4	2	4	2	2	3	2	4	4	4	4	2	4
3	3	3	3	3	3	3	3	3	3	3	3	3	3
4	4	2	4	3	3	4	2	3	4	4	4	3	4
5	5	3	5	2	1	5	5	5	5	5	5	4	5
3	4	4	3	3	3	4	3	2	4	4	4	4	4
4	4	4	4	4	4	4	4	4	4	4	4	4	4
5	5	5	5	1	1	5	1	5	4	4	4	2	5
4	4	2	4	2	2	4	2	4	4	4	4	2	2
5	5	3	5	1	1	5	1	5	1	5	5	1	5
5	5	3	5	2	1	4	1	3	2	5	5	2	5
3	3	3	3	3	2	3	2	3	3	3	4	2	3
5	5	1	5	1	2	5	1	4	5	5	5	2	5
5	5	3	5	4	4	4	2	5	5	4	5	4	4
4	4	2	4	3	2	3	2	4	4	4	4	2	4
4	4	4	4	2	4	4	2	4	3	4	4	2	4
3	4	2	4	3	3	3	2	4	5	5	5	2	4
5	5	1	5	1	1	5	1	5	1	5	5	1	5
4	4	1	4	3	2	4	1	4	4	5	5	2	3
4	5	1	4	3	2	3	2	4	4	4	5	2	4
5	5	2	4	4	3	4	2	4	4	4	5	2	4
3	3	3	5	3	1	3	1	5	3	5	5	1	3
3	3	3	3	2	2	3	2	4	2	4	4	2	4
4	4	2	4	4	3	4	2	4	3	4	4	2	3
3	4	3	3	4	4	3	4	3	5	4	5	4	3
4	5	3	4	2	2	3	1	2	4	4	5	2	4
4	4	1	4	1	1	4	1	4	4	4	4	1	4
3	3	2	3	2	2	3	2	3	3	3	3	2	3
5	4	5	5	3	2	5	1	5	5	5	5	1	5
4	5	3	5	4	5	4	2	4	4	4	5	4	5
3	4	3	4	3	3	3	3	4	4	4	4	3	4
3	3	2	4	2	2	3	3	3	3	4	4	3	3
4	4	4	4	3	3	3	2	3	4	5	5	3	4
4	4	3	4	4	3	3	2	4	5	5	5	3	4
5	5	2	5	5	5	4	3	5	5	5	5	3	2
4	5	3	4	3	3	3	4	3	5	5	5	3	4
3	4	3	3	2	3	3	2	4	4	4	4	3	4
5	5	1	5	5	5	5	1	5	5	5	5	1	5
5	5	4	5	2	2	4	1	5	5	5	5	1	5
4	5	4	4	1	2	5	1	5	2	4	4	2	4
2	3	2	4	4	3	2	4	3	3	3	4	3	3
5	5	3	5	1	1	5	1	5	3	5	5	1	4
4	4	3	4	2	3	4	2	3	3	4	4	2	4
4	5	3	4	4	3	3	2	4	5	5	5	3	4
4	4	2	4	2	2	3	2	4	5	5	5	1	4
5	5	5	5	5	5	5	5	5	5	5	5	5	5
4	4	2	4	4	4	2	3	2	4	4	4	4	3
5	5	2	5	2	2	5	2	5	2	5	5	2	5
3	3	3	3	3	3	3	3	3	3	3	3	3	3

3	3	3	3	3	3	3	2	3	3	3	4	3	3
5	5	1	5	2	2	5	2	4	5	5	5	4	5
5	5	1	5	1	1	5	1	5	1	5	5	1	5
5	5	3	5	1	1	4	1	5	5	5	5	1	5
4	4	3	3	3	3	3	3	3	3	3	3	3	3
3	5	5	4	2	2	3	2	4	4	4	5	2	5
3	3	3	3	3	3	3	3	3	3	3	3	3	3
3	3	4	3	2	2	3	2	4	4	4	5	2	4
5	5	1	5	1	1	5	1	5	1	5	5	1	5
3	4	2	4	2	2	4	2	4	4	2	4	2	4
4	4	3	4	3	2	3	3	5	5	4	4	4	4
4	4	2	4	2	2	4	2	4	2	4	4	2	4
2	3	2	2	3	3	2	4	3	4	2	4	2	2
4	5	2	5	4	4	3	3	5	5	5	5	2	4
3	3	3	2	3	2	3	3	2	3	2	3	2	3
3	3	3	3	3	3	3	3	3	3	3	3	3	3
4	5	3	4	4	4	4	3	3	4	4	5	4	4
5	5	1	5	4	1	4	1	5	5	5	5	1	5
4	4	4	4	4	3	4	4	5	4	4	4	4	4
4	4	4	4	4	2	4	2	4	4	4	4	2	4
5	5	3	5	3	5	5	1	5	5	5	5	1	5
3	4	3	4	3	2	4	2	4	4	3	3	3	3
3	3	3	3	3	3	3	3	3	3	3	3	3	3
5	5	1	5	1	1	5	1	5	3	5	5	3	5
3	4	2	3	3	3	3	2	3	4	4	4	4	3
3	4	2	4	2	2	3	1	4	4	4	4	2	4
4	5	1	5	4	1	4	1	5	1	5	5	1	5
3	4	4	3	2	3	3	3	4	4	4	4	3	3
3	3	3	3	3	5	3	5	5	5	5	5	5	3
4	5	2	5	5	5	3	4	2	5	5	5	5	4
3	3	3	3	3	3	3	3	3	3	3	3	3	3
5	5	1	4	1	1	4	1	5	5	5	5	5	4
5	5	4	5	1	1	5	1	4	3	4	5	2	5
5	5	2	5	2	3	3	4	5	5	5	5	5	5
3	3	3	3	4	3	2	2	4	4	4	5	4	3
3	4	2	4	3	2	4	2	4	4	3	5	2	3
4	5	3	4	4	3	2	3	3	5	5	5	2	3
5	5	5	5	5	5	5	5	5	5	5	5	5	5
4	4	4	4	3	2	4	2	4	2	5	5	2	4
5	5	2	5	4	2	4	2	5	1	4	5	5	5
4	4	4	4	4	3	4	3	4	4	4	4	4	4
5	5	1	5	1	1	5	1	5	1	5	5	1	5
5	5	3	5	4	3	4	2	4	4	5	5	4	5
4	4	4	5	2	3	4	2	3	4	5	5	3	3
4	4	3	4	4	2	4	2	4	4	4	4	2	4
4	4	3	4	2	2	3	2	4	4	4	4	3	2
1	5	1	1	1	1	3	1	3	5	5	5	1	5
3	3	3	3	3	3	3	3	1	3	3	3	2	4
5	5	1	5	5	3	5	1	5	4	5	5	2	5
5	5	3	5	1	1	5	1	5	1	5	5	1	5
4	4	3	4	4	4	3	2	3	4	4	5	4	3
5	5	2	5	3	3	3	3	4	4	4	4	3	4
5	5	1	5	1	1	5	1	5	5	5	5	3	5
4	5	1	4	4	2	4	1	4	5	5	5	2	2
5	5	1	5	1	1	5	1	5	1	5	5	1	5
4	5	3	4	2	1	4	3	5	3	4	4	2	4

4	5	2	4	4	3	3	3	4	4	4	4	4	2
5	5	1	5	1	1	5	1	4	2	5	5	1	5
4	5	3	5	2	2	4	1	5	2	5	5	2	4
3	4	3	4	3	3	3	3	3	3	3	4	3	3
4	4	2	4	3	3	4	2	4	4	4	4	2	4
3	4	1	2	3	4	3	4	2	4	4	4	4	3
4	4	2	4	2	2	4	2	4	4	4	4	2	4
4	3	2	4	2	3	4	2	4	3	4	4	2	4
3	3	3	3	3	3	3	3	3	3	3	3	3	3
5	5	1	5	2	1	3	1	3	5	5	5	1	5
5	5	1	5	3	2	5	1	5	5	5	5	2	5
5	5	4	5	3	3	4	2	4	5	5	5	2	3
5	5	1	5	1	2	4	1	4	2	5	5	1	5
3	3	3	3	3	3	3	3	3	3	3	3	3	3
5	5	1	5	1	1	5	1	5	1	5	5	1	5
4	5	4	4	4	2	4	2	4	5	5	5	1	5
5	5	3	3	4	3	4	2	4	4	5	5	3	3
4	4	2	4	2	2	4	2	2	2	4	4	2	4
3	3	3	3	3	3	3	3	3	3	3	3	3	3
5	5	5	5	1	1	5	1	5	5	5	5	5	5
4	5	5	3	3	3	3	2	5	4	5	5	2	5
3	4	2	4	3	3	2	3	2	2	4	4	2	2
4	5	3	4	2	2	3	1	5	4	5	5	2	5
4	4	3	4	4	4	4	2	4	4	4	4	4	3
3	3	3	4	3	3	3	3	3	3	3	3	3	4
3	3	2	3	3	2	3	3	2	3	3	5	2	3
3	4	4	3	2	2	3	3	2	2	4	4	3	4
3	3	3	3	3	3	3	3	3	3	3	3	3	3
4	4	5	4	4	3	3	1	3	3	5	5	2	4
3	4	2	3	3	3	4	2	4	3	3	4	2	4
4	4	3	4	3	3	3	3	3	4	4	4	3	4
3	3	2	3	4	4	3	4	2	3	4	4	4	3
5	5	3	5	3	3	5	1	5	5	5	5	2	5
3	4	3	3	2	2	3	1	5	5	5	5	3	4
3	3	3	3	2	2	3	2	5	3	3	5	3	3
3	4	2	3	4	4	2	3	2	2	3	5	3	3
5	5	2	5	1	1	4	1	4	3	5	5	1	5
4	4	4	4	3	3	4	2	2	4	4	5	2	4
3	3	3	3	3	3	3	4	3	3	3	3	3	3
4	4	1	4	3	2	4	2	4	3	4	4	2	4
5	5	5	5	5	5	5	5	5	5	5	5	5	5
4	5	1	5	1	1	4	1	4	3	5	5	1	4
5	5	5	5	5	1	5	1	5	4	5	5	5	3
4	4	3	4	2	3	4	2	4	4	4	4	2	4
3	4	2	4	2	2	4	2	4	3	5	5	1	4
4	4	3	4	4	3	4	2	4	4	4	4	4	4
5	5	4	5	2	2	5	1	4	4	5	5	1	5
5	5	1	5	1	1	5	1	5	5	5	5	1	5
4	4	3	4	3	2	3	1	1	2	5	5	3	4
5	5	1	5	1	1	5	1	5	5	5	5	1	5
3	4	2	3	4	2	3	2	4	4	4	4	3	4
5	5	1	5	2	2	4	1	5	2	5	5	2	5
4	5	1	4	2	2	4	2	5	2	4	5	2	4
3	4	3	4	3	2	4	2	3	2	4	4	2	4
4	4	2	4	4	2	3	3	3	4	4	4	2	4
3	4	1	2	4	4	3	3	3	3	3	3	3	3

4	4	2	4	4	3	4	2	3	4	4	4	4	4
3	4	3	5	4	1	5	1	5	5	5	5	1	4
3	3	3	3	3	3	3	3	3	3	3	3	3	3
3	3	2	3	3	3	3	2	3	3	3	3	3	3
5	5	3	5	5	1	3	1	3	5	5	5	1	3
4	4	1	4	1	4	4	1	4	3	3	4	1	4
5	5	5	5	2	2	5	2	5	4	4	4	2	5
3	3	3	3	3	3	3	3	3	3	3	3	3	3
3	3	1	3	3	3	3	3	2	4	4	5	3	3
5	5	2	5	2	1	4	1	5	4	5	5	1	5
3	4	2	3	4	2	3	2	4	3	4	5	3	3
5	5	4	5	5	5	4	2	5	5	5	5	5	5
4	4	2	5	5	3	5	2	5	2	5	5	5	5
5	4	3	4	2	2	4	1	5	4	4	5	2	4
5	4	5	3	4	4	3	3	4	4	4	4	5	3
4	5	4	4	5	3	3	4	3	5	5	5	3	3
3	4	4	3	2	4	3	2	4	4	4	4	4	4
2	4	5	3	4	3	3	3	4	4	4	4	3	3
4	4	4	4	3	2	3	2	4	4	5	5	5	5
5	5	5	5	5	5	5	5	5	5	5	5	5	5
2	3	2	3	5	5	3	4	2	4	4	5	4	4
4	3	3	3	3	2	3	3	4	3	2	3	3	2
5	5	1	5	1	1	5	1	5	2	5	5	1	5
3	4	2	3	3	2	3	2	4	4	4	4	3	4
5	5	1	5	1	1	5	1	5	5	5	5	1	5
4	4	3	4	4	2	3	2	4	4	5	5	2	4
5	5	3	4	2	2	4	1	4	4	5	5	1	4
4	4	4	4	4	4	4	4	4	4	3	3	3	3
5	5	3	4	2	2	4	4	4	4	4	4	2	4
3	4	2	4	3	2	3	2	4	4	4	4	2	4
4	5	1	4	3	3	3	2	4	5	5	5	1	4
3	3	3	3	3	3	3	3	3	3	3	3	3	3
2	3	2	3	2	3	2	2	2	3	3	3	2	3
5	5	2	4	1	2	4	1	4	2	5	5	1	4
5	1	1	5	1	1	5	1	5	1	5	5	1	5
2	3	2	2	2	3	3	3	3	2	3	3	2	4
4	4	3	4	3	3	4	2	4	3	4	4	2	3
5	5	1	5	1	1	5	1	5	1	5	5	1	5
3	4	1	2	2	2	3	2	4	4	5	5	3	3
5	5	2	4	2	4	4	2	4	2	4	4	2	4
4	4	2	4	3	3	3	2	4	4	4	4	2	4
5	5	4	5	2	2	4	2	5	5	5	5	2	5
4	5	4	5	1	1	5	1	5	1	5	5	1	4
3	3	2	3	2	3	3	2	4	3	3	4	3	3
4	4	3	4	2	2	4	2	4	4	4	4	2	4
3	3	3	3	3	3	3	3	3	3	3	3	3	3
5	5	4	5	3	1	5	1	5	5	5	5	1	5
5	5	3	5	4	1	4	3	5	4	5	5	1	4
4	4	4	4	4	4	4	4	4	5	5	5	5	4
5	5	1	5	3	1	5	5	5	5	5	5	5	5
5	5	2	5	1	2	2	2	5	5	5	5	1	5
5	5	1	5	1	1	5	1	5	5	5	5	1	5
4	5	1	4	2	1	3	1	4	5	5	5	1	4
3	5	5	3	2	2	2	1	2	5	5	5	3	4
4	4	3	4	2	2	3	2	4	4	4	4	2	4
4	5	5	4	4	5	4	2	5	5	5	5	5	5

2	4	3	3	3	4	3	3	4	3	4	4	2	3
5	5	2	5	5	3	4	2	5	5	5	5	2	4
5	5	3	5	1	1	5	1	5	5	5	5	1	5
5	5	1	5	1	1	4	1	4	3	5	5	2	5
4	3	4	3	4	3	4	3	4	3	4	3	4	4
3	3	3	3	3	3	3	3	3	3	3	3	3	3
3	3	3	3	3	3	3	3	3	3	3	3	3	3
5	5	5	5	2	4	5	2	5	4	5	5	2	5
3	3	3	3	3	3	3	3	3	3	3	3	3	3
4	4	4	4	4	4	4	4	4	4	4	4	4	4
5	5	5	5	5	3	3	1	5	5	5	5	3	4
4	4	3	5	3	2	2	5	3	4	4	5	4	3
4	4	3	4	3	2	4	2	4	2	4	4	2	4
4	4	2	4	2	2	3	2	4	4	4	4	2	4
5	5	3	5	3	2	3	2	4	4	5	5	3	4
4	4	2	4	2	2	3	2	4	4	4	4	3	4
3	4	2	4	2	2	4	2	4	4	5	4	2	3
4	4	2	3	4	3	3	4	4	4	4	4	4	3
3	3	3	3	3	3	3	3	3	3	3	3	3	3
4	4	2	4	4	3	2	3	3	3	4	4	4	4
3	4	4	3	3	3	3	3	4	3	3	3	4	4
4	4	1	4	4	2	4	2	4	2	4	4	2	4
4	4	4	4	2	2	4	2	4	4	4	4	2	4
5	5	2	5	3	2	4	1	5	4	5	5	2	5
5	5	1	5	1	1	5	1	1	4	5	5	1	5
3	4	1	3	3	4	3	2	4	5	5	4	3	4
4	4	4	3	3	3	3	3	3	3	3	3	3	3
4	4	5	4	2	2	4	1	4	4	4	4	2	3
3	4	2	4	3	2	4	2	4	4	4	4	2	4
1	5	5	3	1	2	5	1	5	5	5	5	1	5
3	4	2	3	2	3	3	2	4	3	3	3	2	3
5	5	3	4	5	2	4	1	5	5	5	5	3	5
5	5	5	5	5	3	5	1	5	1	5	5	1	5
3	3	3	3	3	3	3	3	3	3	3	3	3	3
5	5	1	5	1	1	5	1	5	5	5	5	5	5
4	4	2	4	3	3	4	3	3	3	3	4	3	4
3	5	1	5	1	1	5	1	5	5	5	5	1	5
4	4	3	3	3	3	3	3	3	4	5	5	3	3
4	4	2	5	3	3	4	2	2	5	5	5	2	4
3	3	2	3	2	2	3	1	3	3	4	4	2	5
4	4	3	4	3	3	3	3	4	4	4	4	3	3
5	5	4	5	2	1	5	1	5	5	5	5	1	5
4	4	3	4	2	2	3	2	4	4	4	5	3	4
4	4	3	4	2	1	4	1	4	4	4	5	1	4
5	5	4	5	2	2	5	1	5	4	5	5	2	5
3	3	1	3	3	3	3	3	3	3	3	4	3	3
3	4	3	3	4	3	3	4	4	3	3	3	3	3
5	5	1	5	5	3	3	2	3	5	5	5	3	4
4	4	3	4	3	3	3	2	3	4	4	4	4	4
5	5	5	5	1	1	5	1	5	1	5	5	1	5
1	5	1	4	5	5	4	1	5	3	5	5	2	4
5	4	2	4	2	2	4	3	3	3	4	5	2	4
5	5	3	5	5	1	5	1	3	5	5	5	1	5
3	3	3	3	3	3	3	3	3	3	3	3	3	3
5	5	4	5	2	2	5	5	5	2	5	5	2	5
4	4	4	4	2	2	4	2	4	3	4	4	2	4

5	5	1	5	1	5	5	1	5	5	5	5	1	5
4	5	2	5	2	2	4	2	3	3	5	5	2	5
3	5	3	3	3	3	2	2	4	4	4	5	2	3
5	5	5	5	3	1	5	1	5	3	5	5	5	5
4	4	3	4	3	2	4	2	5	3	4	4	2	4
5	5	3	5	3	1	5	1	5	1	5	5	2	5
3	3	3	3	3	3	3	2	3	3	3	4	3	3
3	4	3	2	3	4	2	4	3	2	3	4	3	2
3	3	3	3	3	3	3	3	3	3	3	3	3	3
5	5	1	5	1	1	5	1	5	5	5	5	1	5
3	4	5	4	2	2	3	1	5	5	5	5	4	5
5	5	4	4	3	3	4	3	4	5	5	5	4	3
5	5	1	5	2	1	3	2	4	2	5	5	2	5
3	3	2	3	3	3	3	3	3	3	3	4	3	3
3	3	3	3	3	3	3	3	3	3	3	3	3	3
3	4	2	3	5	4	3	3	4	4	4	4	2	4
4	4	3	4	3	2	4	2	4	5	5	5	2	4
3	3	3	3	4	4	3	4	3	4	4	4	4	3
3	3	3	3	3	3	3	3	3	3	3	3	3	3
5	5	1	5	1	1	5	1	4	1	5	5	1	5
5	1	5	5	2	1	4	3	5	5	5	5	2	5
5	5	5	5	5	5	5	1	5	5	5	5	5	5
3	4	4	4	5	4	3	3	3	5	5	5	5	4
2	3	3	3	3	3	3	3	3	3	3	3	3	3
3	3	4	3	4	4	3	3	4	4	4	4	4	3
4	5	1	4	1	1	4	2	5	1	5	5	1	4
4	5	3	4	2	1	4	1	5	5	5	5	1	5
2	4	4	3	3	4	2	3	5	3	4	4	3	2
4	4	2	4	1	2	4	2	4	2	4	4	2	4
4	4	4	4	4	4	4	3	4	4	4	4	3	2
5	5	5	5	2	2	3	3	4	5	5	5	3	3
3	4	4	4	4	4	4	4	4	4	4	4	4	4
3	3	1	3	3	3	3	3	3	3	3	3	3	3
3	3	3	3	4	4	3	4	4	4	4	5	2	3
4	4	4	4	2	2	4	2	4	3	4	4	3	4
3	4	3	4	3	4	3	4	3	3	3	3	3	3
3	4	3	3	3	3	3	3	4	3	4	5	3	3
2	2	2	2	5	5	2	4	2	4	4	5	4	2
5	5	3	5	4	2	4	2	4	4	5	5	2	5
5	5	5	5	5	5	5	5	5	5	5	5	5	5
4	4	3	4	2	2	3	2	4	3	4	4	2	3
4	4	3	4	2	2	3	2	4	3	4	5	2	4
4	3	3	3	3	3	3	3	3	3	3	3	3	3
4	5	3	5	5	5	3	5	2	5	5	5	3	3
3	3	3	3	3	3	3	3	3	3	3	3	3	3
4	4	2	4	3	3	4	2	3	4	4	4	3	4
4	3	3	4	4	3	4	4	4	4	4	5	2	4
3	3	3	3	3	3	3	3	3	3	3	3	3	3
4	4	4	4	3	3	2	4	3	4	4	4	4	4
5	5	1	5	1	1	5	1	5	1	5	5	1	5
4	4	5	4	3	3	4	3	3	4	4	4	3	3
4	5	3	4	2	2	4	2	4	4	5	5	3	4
3	3	3	3	3	3	3	3	3	3	3	3	3	3
5	5	2	5	1	1	5	1	5	4	5	5	3	5
4	5	1	3	3	2	4	3	3	5	5	5	2	5
5	5	1	5	1	1	5	1	5	1	5	5	1	5

4	4	3	4	3	2	4	3	4	5	5	5	2	4
5	5	2	5	2	2	5	2	5	2	5	5	1	5
5	5	4	5	4	3	4	2	4	4	4	5	4	4
3	4	2	3	4	4	3	2	3	4	4	4	2	3
3	4	2	3	4	4	4	2	4	4	4	5	3	4
3	5	4	3	3	3	2	4	4	4	4	4	4	3
4	5	2	5	5	2	4	2	4	4	5	5	5	4
3	5	5	3	5	5	1	3	2	5	5	5	3	2
3	4	2	4	3	2	3	2	4	4	4	5	3	5
3	4	2	3	4	3	3	3	3	5	5	4	3	3
5	5	4	4	3	3	3	2	4	4	4	4	2	4
5	5	3	4	1	2	4	1	5	5	4	5	1	4
5	5	5	5	4	2	3	3	4	4	5	5	3	3
4	4	4	4	4	3	4	2	4	2	4	4	3	4
5	5	1	4	2	1	4	1	4	4	5	5	2	5
4	5	3	4	4	3	3	2	5	4	5	5	3	4
5	5	2	5	2	3	4	2	5	5	5	5	2	4
2	3	3	3	3	3	2	3	2	3	4	4	2	2
5	5	5	5	5	5	5	5	5	5	5	5	5	5
4	4	2	4	2	2	4	2	4	2	4	4	2	4
2	5	5	5	5	5	2	4	5	5	5	5	5	2
4	4	3	4	2	2	4	2	4	4	4	4	4	4
5	5	5	5	1	1	5	1	3	1	5	5	1	5
4	4	3	4	4	2	3	1	4	4	5	5	3	4
4	4	1	4	1	2	4	1	4	2	4	4	1	4
5	5	4	5	5	1	5	1	5	5	5	5	2	5
5	5	5	5	5	5	5	5	5	5	5	5	5	5
5	5	1	5	1	1	5	1	5	5	5	5	1	5
5	5	3	5	5	5	4	2	5	3	4	5	3	4
4	4	3	3	4	3	3	2	2	4	4	4	2	3
2	4	2	4	3	3	3	2	4	4	4	4	2	4
4	4	3	4	2	3	3	2	4	4	3	3	2	4
3	4	3	3	3	4	4	2	4	4	5	4	1	3
3	4	4	4	4	3	3	3	3	4	4	4	3	3
5	5	1	5	1	1	5	1	3	5	5	5	1	4
3	3	3	3	3	3	3	3	3	3	3	3	3	3
4	4	4	4	3	2	3	2	4	4	4	4	2	3
5	5	3	5	1	1	5	1	5	5	5	5	5	5
3	3	3	3	3	3	3	3	3	3	3	3	3	3
4	4	3	4	2	2	4	2	4	4	4	4	2	4
5	5	1	5	1	2	4	1	5	5	5	5	5	5
3	3	2	4	4	3	2	3	2	4	4	4	3	3
4	4	3	4	3	3	4	2	4	4	4	4	3	4
4	5	3	5	3	3	5	2	4	3	3	4	3	4
5	5	5	5	1	2	5	2	5	4	5	5	2	5
4	4	4	4	4	3	3	4	4	4	4	4	4	4
4	4	3	4	3	3	3	2	4	4	4	4	4	4
3	3	2	4	2	3	3	2	4	2	4	4	3	3
5	5	5	5	5	5	5	5	5	5	5	5	5	5
4	4	4	4	3	5	4	4	3	5	4	5	2	4
3	4	2	3	3	3	2	3	3	4	4	5	3	3
4	4	4	4	4	3	4	2	4	4	4	4	4	4
3	3	3	3	3	3	3	3	3	3	3	3	3	3
3	4	3	3	4	3	4	4	3	4	4	4	3	3
4	4	3	4	4	2	4	2	5	4	5	5	2	4
3	3	3	3	4	4	2	4	2	4	4	4	2	3

3	3	3	3	3	3	3	3	3	3	3	3	3	3
4	4	3	3	3	3	3	3	4	4	4	4	3	3
4	3	3	4	4	3	2	2	3	4	4	4	3	3
3	3	3	3	3	3	3	3	3	3	3	3	3	3
4	4	4	4	3	3	4	1	5	4	4	5	2	5
5	5	5	5	3	2	3	1	5	5	5	5	3	5
5	5	1	4	2	2	4	1	5	5	5	5	2	5
5	5	5	5	3	3	3	3	5	5	5	5	5	3
4	4	2	4	3	2	4	2	4	4	4	5	2	4
5	5	1	5	1	1	4	1	4	3	4	5	1	4
4	5	5	5	4	3	4	1	5	3	4	5	5	5
5	5	1	5	1	1	5	1	5	1	5	5	1	5
4	4	4	4	4	4	4	4	4	4	4	4	4	4
3	5	3	3	3	3	3	3	3	3	3	5	3	3
5	5	1	5	1	1	5	1	5	5	5	5	1	5
3	3	4	3	4	3	3	3	3	4	4	4	3	3
3	4	4	3	4	4	4	3	4	4	4	4	4	4
4	4	4	4	3	3	4	2	4	3	4	4	2	4
4	4	3	4	4	4	4	4	4	4	4	4	4	4
4	4	4	4	3	4	3	3	3	4	4	4	4	4
5	5	1	5	5	1	5	1	5	1	4	5	2	5
4	4	1	4	3	2	3	3	3	4	4	4	3	4
5	5	5	5	4	4	5	1	4	5	5	5	2	3
5	5	2	5	1	1	4	2	4	5	5	5	3	4
4	4	3	4	3	2	3	2	1	3	4	4	4	3
3	3	3	3	3	3	3	3	3	3	3	3	3	3
5	5	5	5	5	5	5	5	5	5	5	5	5	5
4	4	3	4	3	2	4	3	4	4	4	4	4	4
5	5	1	5	4	2	5	1	5	5	5	5	2	5
3	4	2	3	2	2	2	2	2	2	4	4	2	2
4	4	4	4	4	4	4	4	4	4	4	4	4	4
3	3	2	3	3	3	3	2	4	4	4	4	4	3
4	4	2	4	5	4	3	3	4	5	4	5	2	3
4	4	3	4	2	2	4	2	4	3	3	4	2	4
5	5	4	5	5	1	5	1	5	5	5	5	1	5
4	4	2	3	3	3	3	1	4	3	4	4	3	4
5	5	3	5	1	1	5	1	4	5	5	5	2	5
3	4	2	3	2	3	2	3	3	4	5	5	3	4
5	5	1	5	1	1	5	1	5	5	5	5	1	5
4	5	3	4	4	2	3	1	5	5	5	5	2	4
5	5	3	5	3	3	5	2	5	4	5	5	3	5
4	5	2	4	2	2	4	2	4	4	4	5	2	5
5	5	5	5	5	3	5	3	4	5	4	5	5	5
4	4	4	4	4	4	4	4	4	4	4	4	4	4
5	5	3	5	4	3	4	2	4	5	5	5	3	4
4	5	5	4	3	3	4	2	5	5	5	5	4	3
4	4	4	4	2	2	4	2	4	4	4	4	2	4
2	3	4	3	3	3	2	3	2	3	3	4	4	3
3	3	3	3	3	3	3	3	3	3	3	3	3	3
3	4	3	4	3	4	4	4	4	3	4	5	4	4
3	3	3	3	3	3	3	3	3	3	3	3	3	3
3	3	3	3	3	3	3	3	3	3	3	3	3	3
2	3	2	2	3	2	3	2	3	3	3	3	2	3
4	4	4	3	3	2	4	4	4	4	5	4	4	4
5	5	1	5	1	1	5	1	5	1	5	5	1	5
4	5	4	5	2	4	5	4	5	4	3	4	2	5

4	4	5	4	2	2	4	2	4	4	5	4	1	4
4	4	2	4	4	3	3	4	3	4	4	4	3	4
4	5	4	4	4	3	2	4	3	4	4	5	4	2
5	5	3	5	2	2	5	2	5	5	5	5	3	5
4	4	3	4	2	2	4	2	4	2	4	4	2	4
5	5	5	5	2	2	5	2	5	5	5	5	2	5
4	5	2	5	4	2	4	2	4	4	4	4	4	4
5	5	1	5	5	2	3	1	5	4	5	5	3	5
5	5	3	5	4	5	4	4	4	5	5	5	3	4
5	5	1	5	2	2	2	2	3	3	4	5	2	5
4	4	1	4	1	1	4	1	4	3	4	4	1	4
4	4	3	4	2	2	3	2	5	3	5	5	2	4
3	5	2	4	2	2	3	2	4	4	3	4	2	3
5	5	1	5	1	1	5	1	4	1	5	5	1	5
3	3	4	2	3	4	3	3	4	4	3	3	3	3
4	4	3	4	4	2	3	2	3	4	4	4	2	3
3	4	4	4	4	2	3	2	4	5	4	5	2	4
4	4	2	4	2	2	4	2	4	4	4	4	2	4
4	4	3	4	4	4	4	3	4	4	5	5	2	4
4	4	3	3	3	3	3	3	3	4	4	4	4	4
5	5	5	5	5	5	5	5	5	5	5	5	5	5
5	5	2	5	2	1	5	1	5	4	5	5	1	5
5	5	2	5	3	3	3	2	4	4	5	5	5	5
4	5	3	5	4	3	4	2	5	5	5	5	3	4
3	3	3	3	3	3	3	3	3	3	3	3	3	3
5	5	1	5	1	1	5	1	5	5	5	5	1	5
4	5	1	4	1	1	3	1	5	5	5	5	2	4
3	4	2	3	3	3	3	3	3	3	3	4	3	3
5	5	1	5	1	1	5	1	5	3	5	5	1	5
3	4	3	3	3	3	3	3	3	3	4	4	2	3
5	5	1	5	1	1	5	1	5	3	5	5	1	5
2	3	3	4	4	4	2	4	2	4	4	5	4	2
3	4	3	4	3	3	3	2	2	3	5	5	2	4
3	3	3	3	4	3	2	4	4	4	4	4	3	3
3	3	2	3	4	4	3	4	2	4	4	4	4	3
2	4	3	3	4	3	3	3	4	4	5	5	2	2
3	3	3	3	3	4	3	3	3	4	3	5	3	3
5	5	1	5	1	1	5	1	5	5	5	5	1	5
4	4	2	4	3	2	4	3	4	3	4	4	2	4
4	4	3	4	4	4	4	4	4	4	4	3	4	4
5	5	3	3	1	1	5	1	5	4	5	5	3	4
4	4	4	4	3	2	3	3	3	3	4	3	4	3
3	4	3	3	1	1	3	2	3	4	4	4	4	4
4	4	2	4	1	1	4	1	4	3	4	4	1	4
2	2	5	2	4	4	2	5	1	5	2	5	5	2
3	4	2	3	3	4	3	2	3	4	4	4	3	2
5	5	1	5	1	1	5	1	5	3	5	5	2	5
3	4	3	3	3	3	3	2	4	5	5	5	3	3
5	5	5	5	1	1	5	1	1	5	5	5	3	5
5	1	1	5	1	1	5	1	5	5	1	5	1	5
4	4	2	4	4	2	3	3	3	4	4	5	3	3
3	4	4	4	4	3	3	1	4	5	5	5	2	3
3	3	3	3	3	3	3	3	5	3	3	3	3	3
4	4	2	4	2	2	4	2	4	4	4	4	2	3
3	4	4	3	4	4	3	2	4	4	5	5	4	3
3	3	3	3	3	3	3	3	3	3	3	3	3	3

4	4	3	4	3	3	3	3	3	3	3	3	3	3
5	5	1	5	1	1	3	1	5	3	5	5	1	5
5	5	2	5	5	5	4	2	5	4	5	5	4	5
5	4	3	5	3	3	3	3	5	4	5	4	4	5
4	4	4	4	4	4	3	3	4	4	4	4	3	3
4	4	3	4	3	3	3	2	3	4	4	3	3	3
4	4	2	4	4	3	3	3	4	4	5	5	3	4
3	3	2	3	3	4	3	4	3	3	4	4	4	3
3	3	3	3	3	3	3	3	3	3	3	3	3	3
5	5	3	5	4	2	4	1	4	5	5	5	2	5
4	4	3	4	2	2	4	2	1	2	4	5	2	5
3	3	3	3	3	3	3	3	3	3	3	3	3	3
4	4	3	3	4	2	3	3	3	4	4	4	3	3
3	5	3	3	4	4	3	3	4	5	5	5	3	3
5	5	1	5	2	1	5	1	5	1	5	5	3	5
3	3	3	4	2	4	3	2	4	2	4	4	3	3
3	3	3	3	4	4	3	2	5	4	3	5	5	3
5	5	5	5	2	3	5	3	5	5	5	5	5	5
4	4	2	4	2	3	4	2	4	4	4	4	2	4
3	3	3	3	3	3	3	3	3	3	3	3	3	3
4	4	2	4	3	3	4	2	4	3	4	4	2	4
1	2	1	1	3	3	2	3	3	2	4	5	3	2
5	5	1	5	1	1	5	1	5	5	5	5	1	5
5	5	5	5	1	1	5	1	5	5	5	5	1	5
4	4	4	4	4	4	4	4	4	4	4	4	4	4
3	3	3	3	3	2	3	2	3	2	4	5	2	3
3	3	3	3	4	3	3	3	3	4	4	4	3	3
4	4	2	4	3	3	3	3	4	4	4	5	3	4
5	5	3	5	1	1	5	1	4	4	3	5	3	5
3	4	4	3	2	2	3	3	3	3	4	4	3	3
5	5	3	5	3	2	4	2	4	4	4	4	3	4
4	5	5	4	4	4	3	1	3	5	5	5	4	3
4	5	2	4	2	2	3	2	3	4	4	4	3	3
4	4	2	4	4	1	4	1	3	3	4	3	2	3
5	5	1	5	1	1	5	1	5	5	5	5	1	5
4	5	5	5	3	3	3	3	4	5	5	5	4	4
3	4	4	3	4	4	3	3	4	4	4	4	3	3
3	3	2	3	2	2	3	2	3	3	3	3	2	3
5	5	1	5	5	5	5	5	5	5	5	5	1	5
3	4	2	4	3	3	4	3	3	4	4	4	2	4
4	4	1	4	3	4	3	4	4	2	4	5	3	3
3	3	3	3	3	3	3	3	3	3	3	4	3	3
2	5	1	5	3	4	2	2	4	5	5	5	1	4
4	4	4	5	4	4	4	2	4	5	5	5	4	5
5	5	1	5	1	1	5	1	5	5	5	5	1	5
4	4	4	4	2	4	2	3	4	4	4	4	4	4
4	4	4	4	2	2	4	2	3	3	4	4	4	4
3	3	5	3	2	3	4	3	4	3	3	4	3	3
3	4	4	4	3	4	4	4	4	4	4	4	4	4
4	4	4	3	2	2	4	2	4	5	5	5	4	4
3	4	3	3	2	2	5	2	5	4	5	5	2	4
5	5	5	5	5	5	5	5	5	5	5	5	5	5
3	3	3	3	4	4	3	3	3	3	4	4	3	3
3	3	3	3	3	3	3	3	3	3	3	3	3	3
4	4	3	4	2	2	4	2	2	2	4	4	2	4
5	5	5	5	5	4	2	1	5	1	5	5	1	1

4	5	3	5	4	4	5	5	5	4	5	5	3	4
5	5	5	5	5	1	5	1	5	5	5	5	1	5
4	5	1	4	1	2	4	1	5	3	4	5	2	5
3	3	3	3	3	3	3	3	3	3	3	3	3	3
3	3	2	3	3	3	3	3	3	3	4	4	3	3
4	4	2	4	3	2	4	3	2	4	4	4	3	4
3	4	4	4	5	4	3	4	3	4	4	5	4	4
4	4	2	4	4	4	4	3	4	4	4	4	4	4
3	3	2	3	3	3	3	2	3	3	3	3	2	3
4	4	4	4	4	3	3	2	4	5	5	5	4	4
5	5	1	5	2	1	4	1	1	4	5	5	3	2
4	4	3	4	3	2	3	2	4	3	3	4	2	4
5	5	1	5	1	1	5	1	5	5	5	5	1	5
5	5	1	5	4	1	5	1	5	1	5	5	1	5
4	4	3	3	2	2	4	2	4	4	4	4	2	4
4	4	2	3	3	2	2	2	2	3	4	4	3	3
3	3	3	3	3	3	3	3	3	3	3	3	3	3
3	4	2	3	3	2	3	3	4	3	5	4	3	3
5	5	1	5	1	1	5	1	3	3	5	5	2	5
4	5	1	5	1	2	4	1	5	2	5	5	3	5
5	5	1	5	1	1	5	1	5	1	5	5	1	5
3	4	3	3	4	4	3	3	3	4	3	5	3	2
3	3	3	3	3	3	3	3	3	3	3	3	3	3
3	3	3	3	3	3	3	3	3	3	3	3	3	3
4	4	2	4	2	2	4	2	4	4	3	4	2	4
3	3	3	3	3	3	3	3	3	3	3	3	3	3
5	5	5	5	4	3	3	3	3	3	5	5	3	5
5	5	1	5	1	1	5	1	5	3	5	5	1	5
5	5	5	5	1	1	5	1	5	1	5	5	1	5
1	1	1	5	1	5	1	5	1	5	5	5	5	1
5	5	1	5	1	1	5	1	5	3	5	5	1	5
5	5	1	5	1	1	5	1	5	1	5	5	1	5
5	5	1	1	1	1	5	1	5	2	5	5	2	5
5	5	2	5	2	2	5	2	3	2	5	5	2	5
4	5	4	4	4	1	4	1	4	1	5	5	2	4
3	4	2	3	2	2	4	2	4	3	4	5	1	3
3	3	3	3	3	3	3	3	3	3	3	3	3	3
3	5	5	5	5	5	2	2	3	5	5	5	5	4
3	2	4	2	3	3	2	3	3	2	4	3	2	4
4	4	4	4	4	4	3	4	4	4	3	4	3	4
4	5	3	3	3	3	3	2	4	4	4	4	3	3
3	3	3	3	3	3	3	3	3	3	3	3	3	3
4	4	3	4	1	1	5	1	5	5	5	5	3	5
4	3	3	4	3	2	4	2	4	4	4	4	2	3
5	5	1	5	1	1	5	1	5	5	5	5	1	5
4	5	4	4	3	4	3	3	3	5	5	5	3	1
4	5	2	4	2	2	4	1	5	4	4	5	2	4
5	5	5	5	5	5	5	5	5	5	5	5	5	5
3	3	3	3	3	3	3	3	3	3	3	3	3	3
2	2	3	2	2	3	3	3	3	2	2	2	2	3
3	4	1	4	5	5	3	5	4	5	5	5	5	3
5	5	1	5	1	1	4	1	4	1	4	4	1	5
4	4	2	4	4	2	3	2	4	4	4	4	2	4
4	4	2	4	3	2	3	2	4	4	4	4	2	4
3	3	3	3	2	2	3	2	3	3	3	3	3	3
4	4	2	4	3	2	3	2	3	3	3	3	3	4

4	4	3	4	4	2	3	2	4	4	5	5	1	4
4	4	3	4	3	2	3	2	4	3	5	5	3	4
2	2	2	2	4	3	3	3	2	4	5	4	4	3
5	5	1	5	5	5	5	5	5	5	5	5	5	5
4	4	2	3	3	2	3	2	3	3	4	4	2	4
4	4	4	4	4	3	3	1	5	3	5	5	1	4
4	4	3	3	3	3	3	3	3	3	3	3	3	3
3	4	3	3	3	3	3	2	4	4	3	4	3	3
5	5	3	5	1	1	5	1	5	1	5	5	1	5
4	5	2	5	4	4	5	1	5	5	5	5	3	5
5	5	5	4	4	3	3	2	4	4	4	5	2	4
5	4	2	5	2	2	4	2	3	4	5	5	3	4
4	4	2	2	3	2	3	2	3	4	4	5	2	3
5	5	5	5	5	5	5	1	5	3	5	5	5	5
5	5	1	5	2	2	5	2	4	4	5	5	2	5
3	4	4	4	4	3	4	4	4	4	4	4	5	4
3	4	5	3	3	3	3	3	3	3	3	3	3	3
3	4	2	3	3	3	3	3	3	3	4	4	2	3
4	4	3	4	3	3	4	3	5	3	5	4	3	4
4	4	2	4	2	3	4	1	4	2	4	4	1	4
3	3	3	3	3	3	3	3	3	3	3	3	3	3
4	4	3	4	3	2	3	2	4	3	4	4	2	4
5	5	3	5	1	1	5	1	5	4	5	5	1	5
3	3	2	3	4	3	2	3	3	3	3	5	3	3
3	4	2	3	3	3	4	2	4	4	4	5	3	3
5	5	2	5	2	2	5	1	5	1	5	5	1	5
4	5	4	5	4	5	4	5	4	4	4	5	4	5
5	4	3	5	3	2	5	1	1	2	5	3	2	5
5	5	2	5	2	2	4	1	4	2	4	5	2	4
4	4	4	4	4	4	3	4	3	4	5	5	4	4
3	4	3	2	3	3	3	2	4	4	4	4	3	4
4	5	3	4	2	1	3	1	4	3	4	5	2	4
5	5	1	5	1	1	5	1	5	5	5	5	1	5
4	4	3	4	2	3	3	2	5	5	5	5	2	3
3	4	4	3	4	3	3	3	3	3	4	4	3	3
3	3	3	3	3	4	3	3	3	5	5	5	3	3
4	4	1	5	1	3	3	1	3	5	5	5	3	5
3	3	2	3	2	2	4	2	4	2	4	4	2	4
5	5	3	5	2	2	5	1	5	1	5	5	1	4
4	5	1	5	1	1	4	1	1	5	5	5	1	3
3	4	3	3	4	4	3	2	4	2	4	4	4	4
4	4	3	4	4	4	4	4	4	4	4	4	4	4
4	4	1	3	3	2	3	2	4	4	4	4	2	3
3	5	5	4	5	5	4	2	5	5	5	5	3	3
5	5	1	5	1	1	5	1	5	1	5	5	1	5
3	4	3	4	4	3	3	3	3	3	4	4	2	4
3	4	3	4	4	4	3	3	4	4	4	4	4	5
3	4	2	3	3	3	4	2	2	4	4	4	3	3
4	4	2	4	4	2	4	4	4	4	4	4	2	4

a21	a22	a23	a24	a25	a26	a27	a28	a29	a30	a31	a32	a33	a34
1	1	5	1	5	1	5	5	1	5	1	1	5	1
4	3	4	2	4	1	5	5	3	2	2	1	4	1
1	1	5	1	5	1	5	5	1	5	1	1	5	1
2	4	3	2	3	2	4	4	3	2	1	1	4	2
2	3	4	2	4	1	3	4	1	3	2	1	3	2
2	2	2	2	4	2	4	2	4	3	3	2	4	4
3	3	3	2	4	2	4	4	1	4	1	1	4	1
1	1	4	2	4	3	4	4	2	3	4	1	4	2
3	3	3	3	3	3	3	3	3	3	3	3	3	3
4	3	4	2	4	4	4	4	4	4	2	2	4	2
3	1	4	1	4	2	4	4	2	4	2	1	3	1
3	3	3	3	3	3	3	3	3	3	3	3	3	3
2	2	2	2	3	1	3	5	2	3	2	2	3	2
3	3	3	3	3	3	4	4	3	3	3	2	3	3
3	2	4	2	4	2	4	5	2	5	2	2	4	2
3	3	4	2	4	2	4	4	2	4	3	3	2	2
3	4	3	2	4	4	3	5	3	3	2	2	4	2
4	3	3	1	5	2	4	4	4	4	2	2	4	4
2	3	3	2	3	1	3	5	3	4	2	1	3	1
3	2	3	2	4	3	3	4	1	3	2	2	3	2
5	3	3	2	3	2	4	5	3	4	2	2	5	2
5	5	5	1	5	1	5	5	5	5	1	1	1	1
4	4	4	4	4	4	4	4	4	4	4	4	4	4
2	2	5	1	4	1	5	5	1	5	1	1	5	1
3	2	4	2	4	2	3	3	2	3	2	1	2	1
4	2	4	1	4	1	4	4	1	4	1	1	4	1
3	2	3	2	4	1	3	4	3	4	2	1	3	1
4	2	4	2	3	4	4	5	3	3	2	1	3	3
3	2	4	1	4	1	4	4	3	4	1	1	4	1
1	3	3	1	4	3	5	5	2	3	2	1	3	1
2	2	4	1	4	1	3	4	4	4	2	1	3	1
4	1	5	1	5	1	5	5	2	5	1	1	5	1
4	2	4	3	4	2	4	4	2	2	2	2	2	2
4	2	4	2	3	1	4	5	5	4	2	2	2	2
4	1	5	1	5	5	5	5	1	5	1	1	4	1
4	3	4	2	3	3	5	5	5	4	4	1	3	3
2	2	4	2	4	2	4	4	3	4	2	1	4	1
3	3	3	3	3	3	3	3	3	3	3	3	3	3
3	2	4	2	4	2	4	4	2	3	2	1	4	1
3	3	3	3	3	3	3	3	3	3	3	3	3	3
3	3	2	3	2	4	3	5	3	3	3	2	3	3
3	3	3	3	3	3	3	3	3	3	3	3	3	3
4	3	4	3	2	4	4	5	4	3	3	2	2	3
5	5	1	1	5	5	5	5	4	3	1	1	4	1
4	3	4	2	4	3	4	4	4	3	2	1	3	2
2	2	4	1	4	1	4	5	3	4	2	1	3	1
4	2	2	2	4	4	4	4	3	4	2	2	4	2
5	5	5	5	5	5	5	5	5	5	5	5	5	5
2	2	4	2	3	1	4	3	2	3	1	1	3	1
3	1	4	1	5	1	5	5	2	4	1	1	5	1

1	1	5	1	5	1	5	5	1	5	1	1	5	1
2	3	3	4	2	4	4	3	4	2	3	3	3	4
4	2	4	2	3	2	4	4	4	4	2	1	3	2
2	2	4	2	4	3	4	4	4	4	2	2	4	2
1	1	5	1	4	1	4	5	1	4	1	1	4	1
3	2	4	2	4	4	4	4	4	4	2	1	3	2
4	3	3	2	4	2	5	4	5	3	2	1	1	2
4	3	3	2	4	3	4	4	4	4	3	2	4	3
3	2	3	2	3	2	3	2	3	2	3	2	3	2
2	2	3	3	4	5	3	4	2	4	2	2	4	2
3	3	3	3	3	1	3	4	3	3	3	2	4	2
1	1	4	2	5	1	4	5	1	5	1	1	5	1
2	1	4	1	3	1	5	4	2	3	2	1	4	1
1	3	3	2	3	1	2	3	5	3	5	3	5	3
4	2	3	2	3	4	3	4	3	3	2	1	1	4
3	3	4	2	4	2	4	4	3	4	4	1	4	2
4	3	4	3	3	1	4	5	2	2	3	1	4	3
3	2	4	1	4	1	4	4	1	4	1	1	4	1
3	3	3	2	2	2	3	4	2	3	2	1	2	2
2	1	3	1	3	2	4	5	3	3	1	1	3	1
2	2	4	2	4	3	4	4	3	4	2	2	3	2
2	2	4	2	5	4	5	5	2	3	1	1	3	1
1	1	5	1	5	1	5	5	1	5	1	1	4	1
5	1	3	1	5	1	5	5	1	4	1	1	1	1
2	2	3	2	3	2	3	3	3	3	3	2	3	3
3	3	2	2	3	2	3	4	4	3	3	1	3	2
2	1	4	1	4	1	4	5	2	4	1	1	3	1
1	2	2	3	2	2	3	4	3	3	2	2	3	2
5	1	4	1	5	2	5	5	1	5	1	1	4	1
3	2	3	2	3	3	4	4	3	3	2	1	3	2
4	3	3	3	4	3	4	4	3	3	3	2	3	3
2	2	5	2	4	1	5	5	2	4	2	1	4	1
4	4	3	3	3	3	4	4	4	3	3	2	2	2
5	3	5	3	5	1	5	5	2	3	3	1	2	2
2	1	5	1	5	1	5	5	1	5	1	1	5	1
3	3	3	3	3	3	3	3	3	3	3	3	3	3
4	3	3	3	3	3	5	5	5	4	2	1	5	1
3	2	4	2	4	2	4	4	2	3	2	1	3	1
3	2	3	3	4	2	4	4	3	4	2	2	3	3
1	1	5	1	5	1	5	5	1	5	1	1	5	1
4	2	4	2	3	4	4	4	3	3	2	1	3	2
1	1	3	1	3	5	3	3	1	3	1	1	3	1
4	2	4	2	4	3	4	4	4	4	2	1	4	2
5	3	3	2	4	5	5	5	2	4	1	1	5	1
1	1	5	1	5	1	5	5	1	5	1	1	5	1
3	3	4	1	5	1	5	5	3	5	1	1	5	1
3	2	3	2	3	1	3	3	2	3	2	1	2	2
4	4	3	3	3	1	4	3	4	3	3	2	4	2
4	4	2	4	4	3	4	4	4	3	2	1	5	2
2	2	3	3	2	2	3	3	3	3	2	2	3	2
3	1	4	1	3	1	4	4	3	4	1	1	3	1
2	2	4	1	5	2	4	4	2	5	1	1	4	2
2	3	4	2	4	2	4	4	4	4	2	2	3	2
1	3	5	2	5	1	4	5	4	4	2	1	4	2
4	2	4	2	4	5	4	5	2	4	1	1	5	1
2	1	5	1	5	2	5	5	1	5	1	1	5	1

1	1	5	1	5	1	5	5	1	5	1	1	5	1
3	3	2	3	3	2	3	3	2	3	3	3	3	3
3	3	3	2	2	5	4	4	2	4	3	1	3	3
2	1	5	1	5	1	5	5	2	5	1	1	5	1
3	3	4	3	4	3	3	3	3	3	3	2	3	2
3	3	3	3	3	3	3	3	3	3	3	3	3	3
1	1	5	1	4	1	4	4	4	4	1	1	4	1
2	2	4	2	4	3	4	4	2	4	2	1	4	1
4	3	2	2	4	2	4	3	4	2	4	3	3	2
2	2	4	2	4	2	3	4	2	4	2	2	4	2
4	2	5	1	5	3	5	5	2	4	1	1	4	1
3	3	3	2	2	2	4	4	2	3	3	1	2	2
3	3	3	2	4	3	4	4	3	3	2	1	3	2
5	3	4	2	4	2	3	5	2	4	2	1	3	4
5	4	3	3	3	5	5	5	4	3	3	1	2	3
4	3	2	2	3	4	3	3	3	3	2	2	4	1
5	5	5	5	5	5	5	5	5	5	5	5	5	5
3	3	3	2	3	1	4	5	3	3	1	1	3	1
5	1	5	1	5	1	5	5	1	5	1	1	5	1
5	3	3	3	5	5	5	5	5	5	1	1	2	1
4	3	3	2	4	1	5	5	3	3	2	1	3	2
3	2	3	3	4	3	3	3	4	3	2	3	4	3
3	2	4	1	3	1	4	4	3	3	1	1	3	1
3	2	4	3	4	3	4	4	3	4	2	2	4	3
3	3	3	3	3	2	3	4	3	3	3	2	3	2
4	2	4	2	4	2	4	4	3	4	2	2	4	2
2	1	4	1	5	1	5	5	1	4	1	1	4	1
4	3	4	2	4	2	4	5	4	4	2	2	4	2
5	5	1	1	3	3	5	5	5	3	1	1	1	1
1	1	3	1	3	4	2	5	4	2	4	3	4	1
3	3	3	3	3	3	3	3	2	3	2	2	3	2
4	3	3	3	4	4	4	4	4	3	3	3	2	3
3	2	3	2	4	2	3	2	3	4	2	1	4	2
4	4	2	2	4	5	3	3	2	3	4	5	4	5
2	2	4	2	3	4	3	4	4	3	2	2	3	2
3	2	5	2	4	2	4	5	4	4	1	1	4	2
1	1	5	1	4	1	4	5	2	3	1	1	4	1
4	3	3	3	4	2	4	4	4	3	3	1	3	3
3	3	3	3	3	3	3	3	3	3	3	3	3	3
5	5	1	3	1	5	3	3	5	1	3	1	3	2
4	4	3	4	3	3	3	3	3	3	3	3	3	3
3	3	3	2	4	3	4	3	4	3	1	1	3	2
2	3	3	2	3	3	3	3	3	3	3	2	3	3
3	2	4	2	4	3	4	4	3	4	2	1	4	2
2	2	3	2	2	2	3	3	2	3	2	2	4	3
4	2	5	1	3	1	5	5	2	5	1	1	4	1
4	2	2	1	5	1	5	5	2	5	1	1	4	1
3	2	3	2	3	1	4	4	2	3	2	2	3	2
3	1	5	1	5	1	5	5	5	5	1	1	5	1
5	5	5	5	5	5	5	5	5	5	5	5	5	5
1	1	5	1	5	1	5	5	1	5	1	1	5	1
4	2	3	2	4	3	5	5	2	4	2	1	4	3
3	3	3	3	3	3	3	3	3	3	3	2	2	3
2	2	5	1	5	1	5	5	1	5	1	1	4	1
3	3	2	3	3	2	3	3	3	3	3	3	3	3
4	4	3	4	3	4	4	3	3	4	4	4	3	4

2	2	4	1	4	1	4	4	3	4	2	1	4	2
2	2	5	2	5	1	5	5	1	4	1	1	3	1
2	2	4	3	4	2	4	4	3	3	3	2	4	2
3	3	3	2	4	3	2	3	3	4	2	2	3	3
4	2	5	2	5	1	5	5	3	5	2	2	5	2
5	2	5	1	5	4	5	5	5	2	2	1	3	1
2	2	4	1	5	3	5	5	2	3	1	1	3	1
3	3	3	3	3	3	3	3	3	3	3	3	3	3
4	4	2	2	2	2	2	4	4	2	3	2	3	3
5	5	5	5	5	5	5	2	5	2	2	1	5	2
1	3	4	1	5	1	5	5	1	5	1	1	5	1
4	3	3	2	4	4	4	5	4	3	2	1	3	2
2	5	1	3	1	4	4	5	5	3	2	1	3	1
3	3	4	2	5	2	4	5	5	4	2	2	4	2
2	2	4	2	4	4	4	4	2	4	1	1	4	1
3	3	3	3	3	3	3	3	3	3	3	3	3	3
1	1	5	1	5	3	5	5	1	5	1	1	5	1
3	3	3	3	3	3	3	3	3	3	3	3	3	3
2	2	4	2	4	2	4	4	4	4	1	1	4	1
5	4	4	3	3	3	5	5	5	4	4	2	3	3
3	2	3	3	3	2	3	4	4	3	3	4	3	4
5	3	3	2	4	5	4	5	5	3	3	1	3	2
3	3	3	2	5	1	3	4	4	3	3	1	3	1
3	5	3	5	3	3	3	3	3	3	3	3	3	3
4	3	3	3	3	3	4	4	4	3	3	3	3	3
4	3	4	3	4	3	4	4	4	4	3	3	3	3
4	1	4	1	5	1	5	5	1	5	1	1	5	1
3	3	4	3	5	2	3	4	4	4	3	1	4	2
3	3	3	3	3	3	3	3	3	3	3	3	3	3
5	3	4	3	4	1	5	5	4	3	2	1	2	1
4	2	4	2	4	1	4	5	4	4	1	1	3	1
5	3	3	2	5	1	5	5	4	5	2	1	5	1
2	1	5	1	5	1	5	5	1	5	1	1	5	1
1	1	5	1	5	1	5	5	1	5	1	1	5	1
1	1	5	1	5	4	5	5	5	5	1	1	5	1
2	2	5	1	4	2	4	5	2	4	2	1	4	1
2	2	4	2	4	2	4	4	3	2	2	2	4	2
4	2	3	2	4	1	5	5	1	3	1	1	2	3
4	2	3	2	4	1	3	4	2	3	2	2	2	2
2	2	4	2	3	2	4	4	2	4	2	2	4	2
3	3	3	3	3	3	3	3	3	3	3	3	3	3
2	1	5	1	5	2	5	5	2	5	1	1	5	1
5	3	4	4	3	5	4	5	3	4	3	2	3	3
4	3	4	2	3	1	4	5	5	3	2	1	3	1
4	3	3	2	3	3	3	3	4	2	2	1	4	3
4	2	4	2	4	4	4	4	4	4	1	1	4	2
3	3	3	3	3	2	3	3	3	3	2	1	4	2
3	2	3	3	3	1	4	5	4	3	2	2	2	2
2	2	4	2	4	2	4	4	2	3	1	1	4	1
3	2	4	2	4	2	4	4	2	4	2	2	4	2
4	4	2	3	4	1	4	4	4	2	1	1	3	3
1	1	5	1	5	1	5	5	3	5	1	1	5	1
2	2	4	2	4	2	4	4	2	4	2	2	4	2
3	3	3	3	3	4	4	4	4	4	3	2	3	2
3	3	3	2	3	2	3	3	2	3	2	2	4	2
3	2	3	2	3	3	3	5	3	3	2	1	3	2

4	3	3	2	4	4	4	4	3	3	2	1	3	2
3	3	3	3	3	3	3	3	3	3	3	3	3	3
1	1	5	1	5	2	5	5	3	4	1	1	4	1
4	4	3	2	3	3	3	3	4	2	3	2	3	3
4	4	5	4	4	5	5	5	5	5	3	1	3	3
1	1	5	1	5	1	5	5	1	5	1	1	5	1
3	3	3	2	4	2	4	4	4	3	2	1	4	2
4	2	3	2	5	1	4	5	4	4	2	1	4	2
3	1	5	1	4	1	4	5	3	4	1	1	4	2
2	2	2	2	2	2	2	4	2	4	2	2	4	2
3	2	5	1	5	2	5	5	2	5	2	1	4	2
4	1	5	1	5	1	5	4	3	4	1	1	5	1
3	1	4	1	4	5	4	4	2	4	1	1	4	1
4	1	4	1	4	3	4	4	4	4	1	1	3	1
4	2	4	2	4	1	4	4	2	4	2	2	3	2
3	1	3	1	4	1	4	4	1	3	1	1	4	1
5	1	5	1	5	1	3	5	1	5	1	1	5	1
3	3	3	3	3	1	3	3	3	3	3	3	3	3
3	3	3	3	4	3	3	3	2	4	3	3	3	2
4	1	5	1	5	1	5	5	1	4	1	1	4	2
4	2	4	1	4	1	4	5	3	4	1	1	4	1
5	2	3	1	3	5	4	5	2	3	3	1	3	2
2	2	4	2	4	2	5	5	2	5	2	1	5	2
4	2	3	2	4	2	4	4	3	3	2	1	3	2
4	4	2	3	5	5	5	5	2	5	1	1	5	1
2	2	2	2	4	2	4	4	4	4	2	2	4	1
1	5	5	1	5	1	5	5	1	5	1	1	4	1
4	3	4	2	3	3	3	3	4	3	2	2	3	2
2	2	4	2	4	2	4	4	3	4	2	2	4	2
3	2	3	2	4	2	5	5	3	3	2	1	3	1
5	3	4	1	4	4	4	5	4	4	3	1	4	1
4	2	3	2	4	3	4	4	3	4	3	2	2	2
1	1	4	1	4	1	4	4	1	4	1	1	4	1
3	2	4	2	3	2	4	5	2	3	2	1	3	2
4	2	4	1	4	1	4	5	1	5	1	1	4	1
5	1	5	1	5	1	5	1	1	5	1	1	5	1
4	3	3	2	3	1	3	5	4	4	3	1	3	1
2	1	5	1	5	1	5	5	1	5	1	1	5	1
4	2	4	2	5	1	5	5	2	4	1	1	4	2
1	1	4	1	4	1	4	5	1	4	1	1	4	1
3	3	4	2	4	1	4	4	3	4	2	1	4	2
3	3	3	3	5	1	5	5	1	5	1	1	4	1
5	1	5	1	5	1	5	5	1	5	1	1	5	1
3	2	4	2	3	2	4	4	4	4	3	2	3	2
4	3	4	2	4	5	5	5	4	4	3	1	3	1
4	2	4	1	5	4	5	5	4	3	4	1	3	1
4	2	4	1	5	1	5	5	5	4	2	1	3	1
3	4	2	4	3	3	4	4	4	3	3	1	3	3
3	2	3	3	3	2	3	3	2	3	3	1	2	4
1	1	5	1	5	1	5	5	1	5	1	1	5	5
4	3	5	2	5	2	5	5	5	4	2	1	4	2
3	2	4	3	4	3	4	4	2	4	2	2	4	2
3	1	5	1	5	1	4	5	1	4	1	1	5	1
5	2	4	2	5	5	5	5	4	4	1	1	4	1
2	2	4	2	4	3	4	4	4	4	2	2	4	2
3	2	3	2	3	2	3	5	5	5	2	1	3	2

5	1	5	1	5	2	5	5	1	5	1	1	5	1
3	2	4	3	3	1	4	4	2	4	2	1	3	1
4	1	4	2	5	5	5	5	4	5	2	2	3	1
3	3	4	2	4	4	4	4	2	4	2	1	3	3
1	1	5	1	5	1	5	5	1	5	1	1	5	1
2	2	4	2	4	2	4	4	2	4	2	2	4	2
3	3	3	3	3	3	3	3	3	3	3	3	3	3
4	4	2	3	4	5	2	3	4	2	4	2	2	4
4	3	4	3	4	4	3	4	4	3	3	3	2	3
1	1	5	1	5	3	5	5	1	5	1	1	5	1
4	2	3	2	3	3	3	3	3	3	3	3	3	3
1	1	4	1	5	1	5	5	1	5	1	1	5	1
5	3	5	2	3	1	5	5	5	4	5	2	3	3
1	3	3	3	5	4	5	5	2	5	1	1	3	1
4	5	5	5	4	5	4	5	4	4	5	4	5	4
5	5	5	5	5	5	5	5	5	5	5	5	5	5
5	4	4	1	5	3	5	5	5	5	1	1	4	2
3	3	3	3	3	3	3	3	3	3	3	3	3	3
3	3	3	3	3	4	3	3	4	3	3	3	3	3
4	2	4	2	4	1	4	4	3	4	1	1	2	1
1	2	4	1	4	1	4	4	4	4	1	1	4	1
2	2	4	2	4	2	2	2	2	4	2	2	4	2
4	2	4	2	3	1	4	4	2	3	2	1	3	1
5	2	5	2	5	5	5	5	1	5	5	1	4	1
2	2	4	2	4	2	4	4	1	4	2	1	4	2
3	1	3	3	3	3	3	4	2	4	2	2	4	2
3	3	4	2	3	2	4	3	4	4	2	2	3	2
4	3	3	3	3	5	4	4	5	4	3	3	3	3
4	2	4	2	4	2	4	4	4	4	2	1	2	2
2	2	4	2	4	2	5	5	2	4	2	1	4	1
1	1	4	1	4	1	4	4	1	4	1	1	3	1
4	2	3	3	4	2	4	4	2	4	2	2	3	2
1	1	5	1	5	1	5	5	1	5	1	1	5	1
4	3	4	3	3	3	3	4	3	4	3	3	3	3
5	2	4	2	4	1	5	5	1	5	1	1	4	1
5	1	4	1	4	1	4	5	1	5	1	1	4	1
3	1	5	1	5	3	5	5	2	5	1	1	5	1
3	2	4	2	3	1	4	4	2	4	1	1	3	1
3	3	4	1	4	1	5	5	1	4	1	1	4	1
3	3	4	1	4	1	5	5	1	4	1	1	4	1
2	2	4	1	3	2	3	5	2	4	1	1	3	2
1	1	5	1	5	1	5	5	5	5	1	1	5	1
3	3	3	2	3	1	4	4	4	4	1	1	3	3
1	1	5	1	5	5	5	5	3	5	3	1	5	1
4	3	4	2	4	1	5	5	4	4	1	1	4	2
2	1	4	1	4	1	4	4	1	4	1	1	2	1
5	5	5	5	5	5	5	5	5	5	5	5	5	5
4	1	5	1	5	2	5	5	4	5	1	1	5	1
5	2	4	4	4	1	5	5	3	3	2	2	3	2
1	1	4	1	3	1	4	5	1	4	1	1	4	1
3	3	3	3	3	3	3	3	3	3	3	3	3	3
3	3	3	5	1	5	1	4	3	4	5	5	3	1
3	3	4	2	2	2	4	4	2	3	2	2	3	2
3	2	4	1	4	4	4	4	1	4	1	1	4	1
3	3	3	3	3	3	3	3	3	3	3	3	3	3
2	2	3	1	4	3	5	5	1	4	1	1	4	1

5	1	5	1	5	1	5	5	1	5	1	1	5	1
4	2	4	2	4	2	4	4	3	4	2	1	3	2
3	3	3	5	3	1	3	3	5	2	5	3	3	2
4	1	4	1	4	1	4	4	4	4	1	1	4	1
3	3	3	3	4	2	4	4	2	4	2	2	4	2
4	3	3	3	3	2	3	4	4	3	3	2	3	3
3	3	4	2	4	2	4	4	4	4	2	1	4	2
3	2	3	2	3	2	3	3	3	3	3	1	2	3
2	2	4	2	4	3	4	4	2	3	1	1	3	2
3	3	3	3	3	3	3	3	3	3	3	3	3	3
3	2	4	2	3	3	4	4	2	4	2	1	3	2
5	2	5	2	5	4	5	5	5	5	1	1	5	5
4	4	4	2	4	2	4	4	4	4	4	4	4	4
4	4	4	4	4	4	4	4	4	4	4	4	4	4
1	1	5	1	5	1	5	5	1	5	1	1	5	1
2	2	4	2	4	3	5	5	3	4	2	2	4	2
1	1	5	1	5	1	5	5	1	5	1	1	5	1
2	2	5	1	5	1	5	5	1	5	1	1	4	1
3	2	3	2	3	3	4	4	2	4	2	1	2	2
2	2	5	1	5	1	5	5	2	4	1	1	5	1
5	2	4	2	5	4	5	5	4	4	3	2	4	2
4	2	4	2	4	2	4	4	3	4	2	2	4	2
2	2	4	2	4	2	4	4	3	4	2	2	2	2
3	3	3	2	3	4	4	5	3	4	2	2	4	2
1	1	5	1	5	1	5	1	5	5	1	1	5	1
2	2	4	2	4	1	4	4	3	3	1	1	4	1
3	2	3	1	4	4	4	4	3	4	2	1	3	1
4	3	3	2	5	2	5	4	2	3	1	1	4	2
4	3	3	1	5	1	3	5	3	5	5	1	3	1
3	2	3	2	4	2	4	4	2	3	2	2	3	2
2	3	3	4	3	2	3	4	2	3	3	2	4	2
5	3	2	2	2	4	2	5	4	2	2	1	2	3
2	1	4	2	4	1	4	4	2	4	1	1	4	1
1	1	4	1	4	1	4	4	2	4	1	1	4	1
3	2	3	2	3	3	3	3	3	3	2	2	3	2
5	1	5	1	5	5	5	5	3	4	1	1	4	1
5	4	2	1	4	1	4	5	5	5	2	1	4	3
3	3	4	2	4	3	4	4	4	4	2	2	3	2
3	2	4	2	4	2	4	3	2	4	2	1	3	2
4	2	3	2	4	2	4	4	3	4	2	1	3	1
5	3	3	2	4	2	5	5	2	4	3	1	4	2
5	5	2	4	5	4	4	5	5	3	4	2	4	5
4	3	3	3	5	3	5	5	5	5	2	1	3	3
2	4	3	2	3	2	4	4	4	3	2	1	4	2
5	3	5	3	3	3	5	5	3	5	3	3	3	3
1	1	5	1	5	1	5	5	4	5	1	1	5	1
4	1	4	1	4	1	4	4	1	4	1	1	4	2
4	3	3	4	4	4	4	4	4	3	2	1	2	2
1	1	5	1	5	1	5	5	1	5	1	1	5	1
3	3	4	2	4	3	4	4	2	4	2	2	3	2
4	1	4	1	4	2	4	5	4	4	1	1	3	1
2	2	4	2	4	1	4	4	1	3	1	1	4	1
5	5	5	5	5	5	5	5	5	5	5	5	5	5
4	2	4	2	3	2	4	3	4	3	2	1	2	2
2	2	5	2	5	2	5	5	2	5	2	2	5	2
3	3	3	3	3	3	3	3	3	3	3	3	3	3

4	2	3	3	3	3	3	3	3	3	3	1	2	3
4	2	3	1	5	4	5	5	2	5	1	1	4	1
1	1	5	1	5	1	5	5	1	5	1	1	5	1
3	1	1	1	3	3	5	5	4	5	1	1	4	1
3	3	3	3	3	3	3	3	3	3	3	3	3	3
2	1	4	1	4	1	4	4	1	4	1	1	4	2
4	1	4	1	5	3	3	3	3	5	3	1	5	1
2	2	4	2	4	4	3	5	2	4	3	2	4	2
1	1	5	1	5	1	5	5	1	5	1	1	5	1
2	2	4	1	4	3	3	4	3	3	3	3	3	3
3	2	4	2	4	4	4	4	4	4	2	1	3	2
2	2	4	2	4	2	4	4	2	4	2	2	4	2
4	3	3	3	2	2	3	4	2	2	3	1	2	3
3	3	3	2	5	1	5	5	4	4	2	1	2	1
3	3	3	3	3	3	2	3	3	3	3	3	3	3
3	3	3	3	3	3	3	3	3	3	3	3	3	3
4	3	4	3	5	2	4	5	4	4	3	1	3	2
4	1	5	1	5	1	5	5	3	5	1	1	4	1
5	3	4	1	4	4	4	4	4	4	1	1	4	1
4	2	4	2	4	2	4	4	4	4	2	2	4	2
1	1	4	3	5	5	5	5	5	3	1	1	5	1
3	2	3	2	3	4	3	4	2	3	2	2	3	2
4	3	3	3	3	3	3	3	3	3	3	3	3	3
1	1	5	1	5	1	5	5	5	5	5	1	5	1
4	3	3	3	4	4	4	4	3	4	2	1	3	3
4	1	4	1	4	2	4	4	3	4	1	1	4	1
1	1	4	1	1	1	5	5	1	3	4	1	5	1
4	4	3	2	3	5	4	4	3	3	2	1	3	3
5	3	3	3	3	5	5	5	5	3	3	3	3	3
5	5	4	4	5	5	5	5	5	4	4	2	2	4
3	3	2	4	2	3	3	2	3	4	3	2	3	4
5	1	5	1	5	2	5	5	5	5	1	1	5	2
3	1	5	1	4	1	5	5	3	4	1	1	4	4
4	3	3	3	5	3	5	5	3	3	2	1	3	2
4	3	3	2	3	3	4	5	3	3	2	1	3	3
3	2	4	2	4	3	3	4	2	4	2	2	4	2
4	2	4	2	4	3	5	5	2	3	2	1	3	2
5	5	5	5	5	5	5	5	5	5	5	5	5	5
3	2	4	2	4	1	4	4	2	4	2	1	4	1
5	2	4	2	4	4	4	4	2	4	2	2	4	2
3	4	4	3	4	4	4	4	3	4	3	2	4	2
1	1	5	1	5	1	5	5	1	5	1	1	5	1
4	2	5	1	5	1	5	5	4	5	1	1	5	1
4	3	3	2	5	3	5	5	4	3	2	1	4	2
2	2	4	2	4	2	4	4	2	4	4	2	4	2
3	2	3	2	4	2	4	4	4	3	2	1	3	2
4	1	5	1	1	5	1	5	3	5	1	1	3	1
2	2	3	2	3	2	2	3	2	2	2	2	2	2
4	1	5	1	4	4	5	5	4	5	1	1	5	1
1	1	4	1	5	5	5	5	1	5	1	1	4	1
4	4	3	2	3	1	4	4	4	3	2	1	3	3
4	3	3	2	4	2	5	5	2	5	2	2	2	2
5	1	5	1	5	5	5	5	4	5	1	1	5	1
5	2	4	1	5	2	4	4	4	4	1	1	3	1
1	1	5	1	2	1	5	5	1	5	1	1	5	1
2	2	4	1	4	3	4	5	2	4	1	1	4	1

3	2	4	1	5	1	4	4	2	4	2	1	3	2
1	1	5	1	5	1	5	5	1	5	1	1	5	1
2	1	4	1	4	5	5	5	1	4	1	1	5	1
3	3	3	2	3	1	4	4	3	4	1	1	2	2
2	2	4	2	4	2	4	4	2	4	2	2	3	2
4	4	3	3	4	1	3	4	4	4	3	1	3	2
2	2	4	2	4	2	4	4	4	4	2	2	4	2
4	2	4	2	4	2	4	4	2	4	2	2	4	2
3	3	3	3	3	3	3	3	3	3	3	3	3	3
3	1	5	1	5	1	5	5	1	5	1	1	5	1
5	1	5	1	5	2	5	1	3	5	1	1	5	1
4	3	4	1	5	3	5	5	4	3	2	1	4	1
1	1	5	1	5	1	5	5	1	5	1	1	5	1
3	3	3	3	3	3	3	3	3	3	3	3	3	3
1	1	5	1	5	1	5	5	1	5	1	1	5	1
4	2	4	2	4	4	4	4	4	4	1	1	4	1
3	3	3	3	3	4	3	3	3	3	3	1	4	3
3	3	3	2	3	1	2	3	4	4	2	1	3	1
3	3	3	3	3	3	3	3	3	3	3	3	3	3
5	5	5	1	5	5	5	5	1	5	1	1	1	1
3	2	3	1	5	4	3	5	3	4	1	1	4	2
2	2	3	2	4	2	4	4	4	3	2	1	3	2
4	2	4	1	3	2	4	5	4	4	1	1	4	1
3	3	4	2	4	3	4	4	3	3	2	2	4	2
3	3	3	3	4	3	4	3	4	3	3	4	3	3
3	2	4	2	3	2	4	4	4	4	2	1	4	2
3	2	4	2	4	3	3	4	1	4	2	1	3	2
3	3	3	3	3	3	3	3	3	3	3	3	3	3
3	2	4	2	4	1	3	4	2	4	1	1	3	1
2	3	3	2	4	3	4	4	2	3	2	2	4	2
3	2	4	2	4	2	4	4	3	4	2	2	3	2
4	3	4	2	4	3	4	4	4	3	2	1	3	2
5	3	4	1	5	3	5	5	4	4	1	1	5	4
5	2	4	1	3	1	3	4	2	3	2	1	4	2
2	2	3	2	3	2	3	3	2	3	2	2	4	2
3	3	3	2	4	3	4	5	2	2	2	1	2	2
2	1	5	1	5	1	5	5	3	5	1	1	3	1
4	2	3	2	4	2	4	4	3	4	2	2	4	2
3	3	3	3	3	3	3	3	3	2	4	3	2	3
3	2	3	1	3	2	4	4	2	4	2	1	4	1
5	4	2	2	2	2	5	5	4	5	2	2	4	3
4	1	4	1	5	1	3	5	2	5	3	3	3	3
5	1	5	1	5	1	5	5	4	5	1	1	1	1
2	2	4	2	4	2	4	4	4	4	2	2	4	2
2	1	4	2	5	1	4	5	1	4	1	1	4	1
4	3	4	2	4	1	4	4	3	4	2	2	3	1
3	1	4	2	5	3	5	5	3	5	2	1	4	1
1	1	5	1	5	1	5	5	1	5	1	1	5	1
5	4	3	2	4	5	4	5	2	3	2	1	3	1
1	1	5	1	5	1	5	5	1	5	1	1	5	1
3	2	3	2	4	2	3	3	3	4	2	1	3	2
2	2	4	2	5	1	5	5	2	4	2	1	3	2
2	2	3	2	4	2	4	5	2	4	2	1	4	1
3	2	3	2	3	2	3	4	2	4	2	1	3	2
3	2	4	2	4	3	4	4	4	4	3	1	3	2
3	3	3	3	3	1	3	3	3	3	3	3	3	3

3	3	4	3	4	3	4	4	3	4	3	1	2	3
1	1	3	1	5	1	5	1	1	5	1	1	4	1
5	4	4	3	3	3	3	3	3	3	3	3	3	3
3	3	3	3	3	3	3	3	1	3	2	2	3	2
4	1	5	1	5	1	4	5	1	4	1	1	5	1
1	1	4	1	4	1	4	4	1	4	1	1	4	1
3	2	4	2	4	2	5	5	2	5	1	1	5	1
3	3	3	3	3	3	3	3	3	3	3	3	3	3
4	3	3	3	4	4	3	5	4	3	2	1	3	2
3	1	5	1	5	1	5	5	2	4	1	1	4	1
2	2	3	2	4	1	3	5	3	4	2	1	4	2
4	2	4	2	5	3	5	5	4	5	2	1	3	3
3	4	2	3	5	1	3	4	2	2	4	2	5	3
4	1	4	1	4	3	4	4	3	4	1	1	4	1
4	3	3	3	4	4	4	3	4	4	3	2	3	3
5	3	3	1	4	4	4	4	4	3	2	1	3	1
3	3	4	2	4	2	4	4	4	3	3	1	3	1
2	4	2	4	4	1	4	4	4	3	4	3	3	3
5	2	5	2	5	1	4	5	2	5	1	1	3	2
5	5	5	3	5	5	5	5	3	5	4	5	4	5
4	4	3	2	4	5	4	4	4	2	3	1	3	3
3	2	3	3	2	3	3	2	3	3	2	3	3	2
1	1	5	1	5	1	5	5	1	5	1	1	5	1
4	2	4	2	3	4	4	4	4	4	2	2	4	2
4	1	5	1	5	1	5	5	1	5	1	1	5	1
3	2	4	2	4	2	4	4	3	4	2	2	4	2
2	2	4	1	4	1	4	5	2	4	2	1	4	1
3	3	3	3	3	3	3	3	3	3	3	3	3	3
2	2	4	2	5	1	4	4	2	4	1	1	5	1
2	3	3	2	4	3	4	5	3	4	2	1	4	2
3	3	3	1	3	4	5	5	1	4	1	1	5	1
3	3	3	3	3	3	3	3	3	3	3	3	3	3
2	2	3	2	3	2	3	4	2	3	1	1	4	1
1	2	4	1	4	1	5	5	1	5	1	1	4	1
1	1	5	1	5	1	5	5	1	5	1	1	5	1
2	2	3	2	3	2	2	3	2	3	2	2	3	2
2	3	3	2	4	2	4	4	2	3	2	2	3	2
1	1	5	1	5	1	5	5	1	5	1	1	5	1
2	2	3	3	4	2	4	4	3	3	3	1	2	1
2	2	4	2	4	2	4	5	2	4	2	2	2	1
3	3	3	3	4	2	4	4	4	3	2	1	3	2
2	2	5	1	5	1	5	5	4	5	1	1	5	1
3	1	3	1	5	3	5	5	1	5	1	1	2	1
3	3	3	3	3	2	3	4	3	3	2	1	3	2
2	2	4	2	4	1	4	4	3	3	2	1	4	1
3	3	3	3	3	3	3	3	3	3	3	3	3	3
3	1	5	1	5	3	5	5	1	5	1	1	5	1
2	2	4	1	4	1	5	5	3	5	1	1	4	1
4	3	3	3	4	4	4	4	5	4	4	1	3	2
4	1	5	1	5	1	5	5	5	5	1	1	5	1
2	1	5	1	2	1	5	5	1	5	1	1	2	1
1	1	5	1	5	1	5	5	1	5	1	1	5	1
2	2	5	1	4	1	4	5	2	4	1	1	3	1
1	1	4	1	3	1	5	5	2	3	1	1	2	1
3	2	4	2	4	4	4	4	3	4	2	1	4	2
3	3	4	2	4	5	4	5	3	4	2	1	2	2

3	3	3	2	3	3	4	5	3	3	2	1	3	2
4	4	5	2	5	2	5	5	5	5	2	1	3	3
4	1	5	1	5	4	5	5	1	5	1	1	4	1
1	1	5	1	3	1	5	5	1	5	1	1	4	1
4	4	4	4	4	4	4	4	4	3	4	3	4	3
3	3	3	3	3	3	3	3	3	3	3	3	3	3
3	3	3	3	3	3	3	3	3	3	3	3	3	3
2	3	5	2	5	3	5	5	4	5	2	1	5	2
3	3	3	3	3	3	3	3	3	3	3	3	3	3
4	4	4	2	4	2	3	4	4	4	2	2	4	3
5	1	5	1	5	1	5	5	4	4	2	1	3	1
1	1	4	1	4	5	4	4	4	5	1	1	3	1
2	2	4	2	4	2	4	4	2	4	2	2	4	2
4	2	4	2	4	4	4	4	4	4	2	2	4	2
4	2	4	2	3	1	5	5	4	3	2	1	3	2
3	2	4	2	4	2	4	4	2	4	2	2	4	2
2	2	4	2	4	3	4	4	3	5	2	1	4	3
4	4	2	3	3	4	3	4	3	3	3	2	3	3
3	3	3	3	3	3	3	3	3	3	3	3	3	3
3	2	3	3	4	1	3	4	3	3	4	2	3	2
2	3	3	3	3	2	4	4	3	3	2	1	3	2
4	3	3	2	4	2	4	4	2	4	2	1	2	2
2	2	4	2	4	2	4	4	2	4	2	2	4	2
4	2	5	2	5	1	5	5	1	5	1	1	4	1
1	1	5	1	5	1	5	5	1	5	1	1	5	1
3	3	3	3	3	3	3	3	3	3	4	2	4	2
3	3	3	3	3	3	3	3	3	3	3	2	3	3
4	3	4	2	4	1	4	4	5	3	3	1	5	2
3	2	4	2	4	4	4	4	2	4	2	2	3	2
1	1	5	1	5	1	5	5	5	5	1	5	3	1
3	2	3	2	3	2	4	3	2	3	2	1	3	2
4	2	5	3	4	1	5	5	4	5	1	1	5	2
5	4	5	1	5	5	5	5	5	5	1	1	5	1
3	3	3	3	3	3	3	3	3	3	3	3	3	3
5	1	5	1	5	1	5	5	1	5	1	1	5	1
3	2	3	2	4	2	4	3	3	4	3	2	4	3
5	1	5	1	5	1	5	5	1	5	1	1	5	1
2	3	3	3	3	3	3	5	4	3	4	1	3	2
2	2	4	2	5	2	5	5	2	4	2	1	4	1
3	2	4	2	5	2	4	5	2	3	2	2	3	2
3	3	4	2	4	2	4	4	2	3	2	2	3	2
3	1	5	1	5	1	5	5	1	5	1	1	5	1
3	2	3	2	4	4	4	4	4	3	1	1	3	1
3	1	3	1	5	1	4	5	2	4	1	1	3	1
2	2	5	2	5	3	5	5	4	5	1	1	4	2
2	3	3	1	3	3	3	3	3	3	3	3	3	3
3	3	3	3	3	3	3	3	3	4	3	2	4	3
5	3	5	3	5	2	5	5	3	2	3	2	3	3
3	2	4	2	3	2	4	4	3	4	2	2	3	2
1	1	5	1	5	1	5	5	1	5	1	1	5	1
4	2	4	2	4	5	4	4	4	4	2	2	4	1
3	2	4	2	3	3	5	5	2	4	2	1	3	2
2	2	5	1	5	1	5	5	1	5	1	1	5	1
3	3	3	3	3	3	3	3	3	3	3	3	3	3
3	2	4	4	5	2	5	5	3	5	2	1	5	1
2	2	4	2	4	3	4	4	3	4	2	2	4	2

3	1	5	1	5	1	5	5	1	5	1	1	5	1
2	2	4	2	4	1	5	5	2	5	2	1	3	1
4	3	3	1	3	2	4	3	4	3	3	1	3	2
3	1	5	1	5	1	5	5	3	5	1	1	5	1
3	3	3	2	4	3	4	4	3	5	2	1	4	1
1	1	5	1	5	1	5	5	3	5	3	1	3	1
4	3	3	2	3	3	3	3	4	3	3	3	3	3
2	3	4	3	3	4	3	2	3	4	3	2	3	4
3	3	3	3	3	3	3	3	3	3	3	3	3	3
5	1	4	1	4	1	5	5	1	5	1	1	5	1
2	2	5	1	5	1	5	5	1	5	1	1	4	1
4	3	4	2	4	1	4	4	4	4	2	1	4	2
1	1	4	1	4	1	5	5	2	4	1	1	4	1
3	3	3	2	4	3	3	4	3	3	3	2	3	3
3	3	3	3	3	3	3	3	3	3	3	3	3	3
4	2	4	2	3	4	4	4	2	3	1	1	3	1
4	3	3	2	2	5	4	5	4	4	1	1	4	1
3	3	3	3	3	3	3	5	3	3	3	3	3	3
3	3	3	3	3	3	3	3	3	3	3	3	3	3
4	1	5	1	5	1	5	5	1	5	1	1	5	1
5	2	5	2	5	1	5	5	5	5	2	2	5	2
5	3	5	1	5	5	5	5	5	5	5	1	5	1
5	3	3	2	3	4	4	5	5	4	2	1	3	3
3	3	3	3	3	3	3	3	3	3	3	3	3	3
3	3	3	3	3	1	4	4	4	4	3	1	3	2
1	1	5	1	5	4	5	5	1	4	1	1	4	1
2	1	4	1	5	3	5	5	3	4	1	1	4	1
3	4	2	4	2	4	2	4	3	2	4	1	3	2
1	1	4	1	4	1	4	4	1	4	1	1	4	1
4	3	3	2	4	1	3	3	4	4	3	2	3	2
3	2	4	3	4	1	5	5	4	5	2	1	4	1
2	2	4	2	4	2	4	4	2	4	4	2	4	2
3	3	3	3	3	3	3	3	3	3	3	1	3	1
3	3	3	3	3	3	3	4	3	4	1	1	3	1
3	2	4	2	4	1	4	4	3	4	2	2	4	2
3	3	3	3	3	3	3	3	3	3	3	3	3	3
3	3	3	3	3	2	3	3	4	3	3	2	3	3
4	4	2	4	2	5	2	2	4	2	4	3	2	4
5	2	4	1	5	1	5	5	3	4	1	1	4	1
5	5	5	5	5	5	5	5	5	5	5	5	5	5
2	2	3	2	4	1	3	4	3	3	1	1	4	1
2	3	4	2	2	2	4	5	3	4	2	1	3	2
3	3	3	3	3	3	3	3	3	3	3	3	3	3
5	4	5	3	5	2	5	5	5	5	2	1	2	1
3	3	3	3	3	3	3	3	3	3	3	3	3	3
3	2	3	2	4	2	4	4	3	4	3	2	3	2
4	4	3	3	4	2	4	3	4	4	4	2	4	3
3	3	3	3	3	3	3	3	3	3	3	3	3	3
4	4	4	4	4	4	3	4	5	4	3	2	3	3
1	1	5	1	5	1	5	5	1	5	1	1	5	1
4	3	4	2	4	4	4	5	4	3	2	1	3	3
4	1	5	1	5	2	5	5	2	5	1	1	4	1
3	3	3	3	3	3	3	3	3	3	3	3	3	3
4	1	5	1	5	1	5	5	1	5	1	1	5	1
4	2	3	1	5	4	4	5	4	4	1	1	2	2
1	1	5	1	5	1	5	5	1	5	1	1	5	1

4	2	3	2	4	2	4	4	2	4	2	1	4	1
1	1	5	1	5	1	5	5	1	5	1	1	5	1
4	2	4	2	4	3	4	4	2	4	2	1	4	1
3	3	4	2	3	4	3	3	3	3	3	2	2	3
3	3	3	3	4	1	5	5	4	3	2	1	4	3
3	3	3	2	2	4	4	4	4	2	2	1	3	2
4	2	4	2	4	1	5	5	2	4	1	1	4	1
5	5	2	5	3	5	3	5	5	2	4	2	2	2
4	3	3	3	5	2	4	5	3	4	2	1	4	2
5	3	3	3	4	5	3	5	2	3	3	3	3	3
4	2	4	2	3	1	5	5	2	4	1	1	3	2
3	1	4	1	4	1	5	5	2	5	1	1	4	1
3	3	4	2	4	1	5	5	5	4	2	1	3	1
2	4	4	2	4	1	4	5	1	5	5	1	5	2
1	1	5	1	4	2	5	5	2	5	1	1	4	1
1	1	4	2	5	1	4	3	4	4	1	1	3	1
4	3	4	2	4	2	4	4	4	4	2	2	5	1
3	3	3	3	3	2	2	4	3	3	3	1	2	2
5	5	5	5	5	5	5	5	5	5	5	5	5	5
2	2	4	2	4	2	4	4	3	4	2	1	3	2
5	5	2	5	5	5	5	5	5	2	3	2	2	2
3	2	4	2	4	2	4	4	4	4	2	1	4	2
1	1	5	1	5	1	5	5	1	5	1	1	5	1
3	2	4	1	4	2	4	5	3	4	2	1	3	1
2	1	4	1	4	1	4	3	1	4	1	1	4	1
1	1	5	1	5	5	5	5	1	5	1	1	1	1
5	5	5	5	5	5	5	5	5	5	5	5	5	5
1	1	5	1	5	1	5	5	1	5	1	1	5	1
3	3	4	1	4	1	4	4	3	2	3	2	4	3
3	3	3	2	4	3	4	4	4	3	2	1	4	2
3	2	3	2	3	1	3	3	4	3	2	1	3	3
2	2	3	2	4	1	3	3	3	4	2	1	4	1
4	2	4	2	4	1	3	4	3	4	2	1	3	1
4	3	3	3	4	4	3	3	2	3	2	2	3	2
1	1	5	1	5	1	5	5	3	5	2	1	4	1
3	3	3	3	3	3	3	3	3	3	3	3	3	3
4	3	3	1	4	3	4	4	3	3	1	1	3	1
1	1	5	1	5	1	5	5	2	5	1	1	5	5
3	3	3	3	3	3	3	3	3	3	3	3	3	3
2	2	4	2	4	3	4	4	2	4	2	2	4	2
5	2	4	1	5	1	5	5	5	5	1	1	5	1
3	2	3	2	4	2	4	4	3	3	2	2	3	2
3	3	4	2	4	2	4	4	4	4	2	2	4	3
3	3	5	3	4	2	5	4	3	4	3	2	4	3
5	1	5	1	4	1	5	5	1	4	1	1	4	1
4	4	4	2	4	4	4	4	4	4	2	1	4	2
4	3	4	2	3	4	4	4	4	4	3	2	3	2
2	2	4	2	4	2	3	4	2	4	2	2	3	2
5	5	5	5	5	5	5	5	5	5	5	5	5	5
5	4	2	4	3	3	5	5	3	4	3	1	2	3
3	2	3	2	3	2	4	4	3	3	2	1	2	2
3	2	3	1	4	2	4	4	4	4	2	1	4	1
3	3	3	3	3	3	3	3	3	3	3	3	3	3
4	3	3	3	4	3	4	4	4	3	2	2	3	2
4	3	4	2	4	4	4	5	4	4	1	1	3	1
3	3	3	4	4	3	4	3	2	4	2	1	4	1

3	3	3	3	3	3	3	3	3	3	3	3	3	3
4	3	3	2	4	3	4	4	4	3	3	2	3	2
3	3	4	3	4	3	3	4	5	3	3	2	2	3
3	3	3	3	3	3	3	3	3	3	3	3	3	3
3	2	4	2	4	3	4	4	2	4	2	1	4	1
5	2	3	1	5	3	5	5	5	5	1	1	3	1
2	1	4	1	5	1	4	4	2	5	1	1	4	1
4	3	3	3	5	3	5	5	3	5	3	1	5	1
4	2	4	2	3	3	4	5	2	4	2	1	3	2
1	1	4	1	4	1	4	4	1	4	1	1	4	1
4	3	3	2	4	5	4	5	4	3	2	1	4	1
1	1	5	1	5	1	5	5	1	5	1	1	5	1
4	4	4	4	4	4	4	4	4	4	4	4	4	4
3	3	3	3	3	3	3	5	3	3	3	3	3	3
5	1	5	1	5	5	5	5	5	5	1	1	5	1
2	3	3	2	4	2	3	3	4	3	2	1	3	2
3	3	3	3	3	2	3	4	4	2	2	1	3	2
3	3	4	2	4	2	4	4	3	4	2	2	4	2
4	3	4	4	4	4	4	4	4	4	4	2	4	2
4	3	4	3	4	2	4	4	4	4	3	1	3	2
5	2	5	2	5	2	5	5	2	5	2	2	5	2
4	2	3	1	4	5	4	5	1	4	2	1	3	3
5	2	5	1	5	5	5	5	5	2	1	1	4	1
4	2	4	1	4	3	5	5	4	4	1	1	4	1
4	2	3	2	4	1	4	4	3	4	2	2	4	1
3	3	3	3	3	3	3	3	3	3	3	3	3	3
5	5	5	5	5	5	5	5	5	5	5	5	5	5
3	2	2	4	4	2	4	4	4	4	3	2	3	3
5	2	5	2	5	3	5	5	3	5	2	2	5	2
2	2	4	2	2	2	1	4	2	4	2	2	3	2
4	4	4	4	4	4	4	4	4	4	2	2	4	4
4	3	3	3	4	3	4	4	3	3	2	2	3	3
4	3	4	3	4	1	4	5	3	4	2	1	4	2
2	2	4	3	4	2	3	4	2	3	2	2	4	2
1	1	5	1	5	3	5	5	4	5	1	1	5	1
3	2	4	3	4	2	4	4	4	4	1	1	3	2
5	1	5	1	5	5	5	5	5	5	1	1	5	1
4	3	4	3	5	4	3	3	4	4	2	1	3	3
1	1	5	1	5	1	5	5	1	5	1	1	5	1
5	2	3	1	4	3	4	4	4	3	2	1	4	1
4	2	5	2	5	4	5	5	3	5	1	1	3	1
2	2	4	2	4	2	4	5	2	4	2	1	4	1
4	4	4	4	4	1	5	5	5	5	5	1	5	5
4	4	4	4	4	4	4	4	4	4	4	4	4	4
3	2	3	1	3	2	4	5	3	4	2	1	3	2
2	2	3	2	5	5	5	5	4	5	2	1	4	2
2	2	4	2	4	2	4	4	3	4	2	1	4	1
4	4	2	3	3	3	3	3	4	3	3	3	1	3
3	3	3	3	3	3	3	3	3	3	3	3	3	3
4	4	3	4	4	4	3	4	3	3	4	4	3	4
3	3	3	3	3	3	3	3	3	3	3	3	3	3
3	3	3	3	3	3	3	3	3	3	3	2	4	2
2	2	3	2	3	2	3	3	2	3	2	1	3	1
4	2	3	2	3	2	4	4	4	4	3	2	3	4
1	1	5	1	5	1	5	5	1	5	1	1	5	1
5	2	4	2	5	4	5	5	4	4	2	1	2	3

4	2	5	2	4	1	4	4	4	4	1	1	4	1
4	4	4	2	4	3	4	4	4	4	4	2	3	2
4	3	3	2	2	2	3	3	3	3	3	1	2	2
3	2	4	1	5	3	5	5	3	4	1	1	5	1
2	2	4	2	4	2	4	4	3	4	2	1	4	2
3	2	5	2	5	2	5	5	2	5	2	2	3	2
2	2	4	2	4	2	5	5	4	4	1	1	4	1
3	2	4	1	3	1	5	5	3	5	3	1	3	2
3	2	2	3	5	4	5	5	4	4	1	1	4	1
2	2	5	2	4	1	5	5	2	5	1	1	5	1
3	1	4	1	4	1	4	5	1	4	1	1	4	1
2	2	5	2	4	1	4	4	2	4	2	2	3	2
2	2	3	2	4	2	4	4	2	4	2	1	3	2
5	1	5	1	5	1	5	5	1	5	1	1	5	1
4	5	4	4	3	4	3	3	3	4	3	4	4	4
3	3	4	2	3	3	4	4	3	3	2	2	3	2
4	2	4	2	4	4	4	4	4	3	1	1	3	1
4	2	4	2	4	2	4	4	3	4	2	1	3	2
4	2	4	2	5	4	5	5	4	5	2	1	3	2
4	3	4	1	4	4	4	4	4	4	2	2	4	2
5	5	5	5	5	5	5	5	5	5	5	5	5	5
1	1	5	1	5	1	5	5	1	5	1	1	5	1
3	3	5	3	5	3	3	5	4	4	3	1	3	1
3	3	3	3	3	2	5	5	5	3	3	1	4	1
3	3	3	3	3	3	3	3	3	3	3	3	3	3
1	1	5	1	5	1	5	5	1	5	1	1	5	1
3	1	3	1	4	1	5	5	2	3	1	1	3	1
4	3	3	2	3	3	3	4	3	3	2	1	2	1
2	1	5	1	5	1	5	5	1	5	1	1	5	1
3	2	3	2	4	2	3	4	3	3	3	1	3	1
1	1	5	1	5	2	5	5	1	5	1	1	5	1
2	4	2	3	4	5	2	5	5	2	4	1	2	3
2	2	3	2	3	1	4	4	2	3	1	1	3	2
3	3	3	3	3	3	4	4	3	3	3	1	3	3
4	4	2	3	2	3	3	4	4	3	3	2	2	3
4	2	2	2	3	3	4	5	4	3	2	1	2	2
4	4	3	3	3	3	3	5	5	3	3	3	3	3
5	1	1	1	5	5	5	5	1	5	1	1	5	1
2	2	4	1	4	2	4	4	3	4	2	2	4	2
3	3	3	3	4	3	3	3	3	3	3	3	3	3
4	1	3	1	5	5	5	5	4	4	3	1	5	1
4	4	4	2	4	3	3	4	3	4	3	2	4	2
4	2	5	2	4	4	4	4	4	4	2	2	4	2
1	1	4	1	4	1	4	4	1	4	1	1	4	1
4	4	2	4	2	3	2	4	2	2	4	4	1	4
3	3	2	2	4	2	4	4	3	3	3	2	2	3
1	1	5	1	5	1	5	5	1	5	1	1	5	1
3	3	3	3	4	4	3	4	4	3	2	2	3	2
3	1	5	1	5	1	5	5	4	5	1	1	5	1
1	1	5	1	5	1	5	5	1	5	1	1	5	1
3	2	4	2	5	5	5	5	5	4	2	1	3	2
2	2	4	2	4	2	4	5	3	3	2	1	4	2
3	3	3	3	3	3	3	3	3	3	3	3	3	3
3	1	3	1	4	3	4	4	1	3	1	1	4	1
4	3	2	3	4	4	4	4	5	3	2	2	3	3
3	3	3	2	3	2	4	3	3	3	3	2	3	3

3	3	3	3	3	3	3	3	3	3	3	3	3	4
1	1	5	1	5	1	5	5	1	5	1	1	5	1
5	2	5	1	4	3	5	5	4	5	2	1	4	1
1	1	5	1	3	1	4	5	1	4	1	1	3	1
4	2	3	3	3	4	3	3	3	3	3	2	3	2
3	3	4	2	4	1	3	3	3	3	2	1	4	3
3	3	3	3	3	2	4	4	2	4	2	1	2	3
3	3	4	3	4	2	3	4	3	3	4	2	4	3
3	3	3	3	3	3	3	3	3	3	3	3	3	3
3	2	4	2	4	4	4	5	3	4	2	2	4	2
2	2	4	2	4	2	4	5	2	5	2	2	5	2
3	3	3	2	3	2	4	4	3	3	2	2	2	2
3	2	4	2	3	2	3	5	4	3	3	1	3	2
4	3	3	2	3	4	3	5	4	4	2	2	3	2
2	1	5	1	5	1	5	5	2	5	1	1	3	1
4	3	3	4	4	3	3	3	3	4	3	4	3	3
4	3	3	3	2	3	3	3	4	2	3	1	3	3
3	3	5	3	5	5	5	5	3	5	2	1	3	2
2	2	4	3	4	1	4	4	3	4	2	1	4	2
3	3	3	3	3	3	3	3	3	3	3	1	3	1
3	2	4	2	4	3	4	4	2	4	2	2	4	2
3	3	3	3	3	4	3	3	3	3	3	3	3	3
1	1	5	1	5	1	5	5	1	5	1	1	5	1
5	1	5	1	5	1	5	5	1	5	1	1	5	1
4	4	4	4	4	4	4	4	4	4	4	4	4	4
3	2	4	2	4	3	4	4	3	3	2	1	4	2
3	3	3	3	3	4	3	4	3	3	2	1	3	2
3	2	4	2	4	3	5	5	4	3	2	1	3	2
3	1	5	1	5	1	5	5	1	5	1	1	5	1
3	3	3	3	3	2	3	4	2	3	2	2	3	2
4	1	4	1	3	1	4	5	4	4	1	1	3	1
4	3	3	2	3	2	4	5	5	3	1	1	3	1
3	2	3	2	2	2	4	4	3	3	3	2	3	3
2	2	3	1	4	3	3	4	3	3	1	1	3	1
5	1	5	1	5	5	5	5	1	5	1	1	5	1
5	3	4	2	4	3	4	4	4	4	2	1	3	1
4	3	3	2	4	3	3	3	3	3	2	2	3	2
3	2	3	2	3	1	3	3	2	3	2	1	3	2
1	1	5	1	5	1	5	5	1	5	1	1	5	1
3	2	3	2	4	3	3	4	3	4	3	1	3	3
3	3	4	3	3	1	4	4	4	3	3	2	3	3
3	3	3	3	3	2	3	4	3	3	3	3	3	3
1	1	5	1	5	4	4	5	2	4	1	1	4	1
4	2	4	2	5	4	5	5	4	4	2	1	4	2
1	1	5	1	5	1	5	5	1	5	1	1	5	1
4	4	2	2	4	4	4	4	4	4	4	2	3	4
4	3	4	2	4	3	4	4	3	4	2	2	4	2
4	3	3	2	4	4	3	4	2	3	3	3	3	2
4	4	4	4	4	3	3	4	4	4	3	4	4	4
4	2	3	2	5	4	4	4	4	3	2	2	2	2
4	3	3	2	5	4	4	4	4	4	4	1	3	1
5	5	5	5	5	5	5	5	5	5	1	1	5	1
3	3	3	3	3	2	3	4	3	3	3	3	3	3
3	3	3	3	3	3	3	3	3	3	3	3	3	3
2	2	4	2	4	2	4	4	3	4	2	2	4	2
5	5	2	1	1	1	3	4	5	1	2	1	2	1

2	1	5	1	5	2	5	5	5	5	1	1	5	1
5	1	5	1	5	5	5	5	5	5	1	1	5	1
3	1	5	1	5	2	5	5	1	4	1	1	4	1
3	3	3	3	3	3	3	3	3	3	3	3	3	3
4	3	4	3	3	3	3	3	4	3	3	1	3	3
3	2	4	2	4	2	4	4	3	4	2	2	4	2
4	4	3	4	4	2	4	4	4	3	3	1	4	3
4	3	4	3	4	3	3	4	3	4	4	1	3	3
2	2	3	2	3	2	3	3	2	3	2	2	4	2
4	3	3	3	4	2	4	5	3	4	2	4	5	2
2	2	4	1	4	1	5	5	2	5	2	1	5	2
3	2	3	2	4	4	4	3	2	3	2	2	3	2
1	1	5	1	5	1	5	5	1	5	1	1	5	1
1	1	5	1	5	1	5	5	1	5	1	1	5	1
2	2	4	2	4	2	4	4	3	4	2	1	4	2
3	2	3	2	4	2	3	4	2	3	2	2	3	2
3	3	3	3	3	3	3	3	3	3	3	3	3	3
2	1	5	1	3	2	4	5	3	3	2	1	2	2
3	1	5	1	5	1	5	5	1	5	1	1	5	1
4	2	4	1	5	1	3	5	2	4	1	1	4	1
1	1	5	1	5	1	5	5	1	5	1	1	5	1
4	4	3	2	4	4	4	4	2	3	2	1	2	1
3	3	3	3	3	3	3	3	3	3	3	2	4	2
3	3	3	3	3	3	3	3	3	3	3	3	3	3
2	2	4	2	4	3	4	4	2	4	2	2	4	2
2	2	3	2	3	2	3	3	3	3	3	3	3	3
5	3	3	3	3	3	5	5	5	5	5	1	5	3
3	1	3	1	5	2	5	5	2	3	1	1	3	1
1	1	5	1	5	1	5	5	1	5	1	1	5	1
5	5	1	5	1	5	5	5	5	1	5	3	1	5
3	1	5	1	5	1	5	5	1	5	1	1	5	1
1	1	5	1	5	1	5	5	1	5	1	1	5	1
2	2	4	2	5	2	4	4	2	4	1	1	3	1
2	2	5	2	5	2	5	5	2	5	2	2	3	2
5	1	4	1	5	3	5	5	5	5	1	1	5	1
2	2	3	2	4	2	4	5	1	3	2	1	4	1
3	3	3	3	3	3	3	3	3	3	3	3	3	3
4	3	4	1	3	3	5	3	5	5	1	1	3	5
3	2	4	4	3	2	4	3	4	3	2	4	4	4
3	4	3	3	4	4	3	4	4	4	4	3	3	4
3	3	3	1	5	4	4	5	3	3	3	1	4	1
3	3	3	3	3	3	3	3	3	3	3	3	3	3
3	3	2	2	5	3	5	5	3	4	2	1	5	1
4	2	3	2	4	2	4	4	3	4	2	2	3	2
1	1	5	1	5	1	5	5	1	5	1	1	5	1
3	3	3	1	3	2	4	4	2	2	1	1	1	1
4	2	4	1	5	3	4	5	2	4	1	1	4	1
5	5	5	5	5	5	5	5	5	5	5	5	5	5
3	3	3	3	3	3	3	3	3	3	3	3	3	3
3	3	3	3	3	2	3	3	3	3	3	3	3	3
5	1	4	3	2	4	5	5	5	2	5	1	1	4
3	1	4	1	4	1	5	5	1	5	1	1	5	1
3	2	4	2	4	2	4	4	2	4	2	2	3	2
4	2	3	2	4	2	4	4	2	4	2	2	3	2
3	2	3	2	3	2	3	3	2	3	2	2	3	2
3	2	3	2	3	1	3	4	3	4	2	2	3	2

4	2	4	1	5	4	5	5	2	5	2	2	4	2
2	2	4	2	3	4	4	4	2	4	2	2	4	2
4	3	3	4	4	2	4	3	3	3	3	2	2	3
5	5	5	5	5	5	5	5	5	5	5	1	5	5
3	2	4	2	4	3	4	4	4	4	2	2	4	2
4	2	3	1	5	1	5	5	4	5	1	1	4	1
3	3	3	3	3	3	3	3	3	3	3	3	3	3
3	2	3	2	3	2	3	4	4	3	2	2	4	2
1	1	5	1	5	1	5	5	1	5	1	1	5	1
4	1	5	1	5	1	5	5	2	5	1	1	4	2
3	2	3	1	5	2	5	5	2	4	1	1	3	1
3	1	5	1	5	3	5	5	3	5	2	1	4	1
3	3	3	3	4	4	4	4	4	4	2	2	3	2
3	3	3	3	3	1	5	5	1	5	1	1	5	1
2	2	4	2	4	1	4	4	1	5	1	1	5	1
5	5	4	2	5	1	5	5	5	5	5	1	5	2
5	3	3	3	5	5	3	4	4	3	3	3	3	3
3	3	3	3	3	3	3	3	3	3	2	2	4	3
3	3	4	3	5	2	5	5	2	4	3	1	4	2
1	1	4	1	4	1	5	5	1	4	1	1	4	1
3	3	2	3	3	3	3	3	3	3	3	3	3	3
4	1	4	1	4	2	4	4	2	4	1	1	3	1
4	1	5	1	5	2	5	5	3	5	1	1	5	1
2	3	3	2	3	3	3	3	3	3	2	2	3	3
4	3	3	3	4	4	4	4	4	4	2	2	3	2
1	1	5	1	5	1	5	5	1	5	1	1	5	1
4	4	5	4	5	4	3	4	5	4	3	3	4	4
2	1	4	2	5	1	5	5	2	4	1	1	4	1
4	1	4	1	4	1	5	5	3	4	1	1	4	1
5	3	3	3	4	3	4	4	4	4	2	1	4	2
4	2	3	1	4	1	4	4	4	4	1	1	3	1
2	1	4	1	3	1	5	5	1	4	1	1	3	1
1	1	1	1	5	5	5	5	5	5	1	1	5	1
5	3	3	3	5	2	4	5	4	3	2	1	3	1
4	3	3	3	3	3	3	3	3	3	3	3	3	3
3	3	3	3	3	3	3	3	3	3	3	3	3	3
1	1	5	1	5	1	5	5	3	3	1	1	5	1
3	3	3	2	4	1	3	3	2	3	2	2	3	2
2	2	4	1	5	1	5	5	2	5	1	1	4	1
3	1	4	1	5	1	4	5	1	3	1	1	4	1
4	3	4	2	4	3	3	4	4	4	2	1	3	2
4	3	4	3	4	3	4	4	4	4	4	4	4	4
3	2	4	2	4	2	4	4	3	4	2	2	3	2
5	3	3	3	3	1	3	5	3	3	3	3	3	3
1	1	5	1	5	1	5	5	1	5	1	1	5	1
4	3	3	3	3	3	4	4	4	3	3	2	4	4
4	3	4	3	4	4	4	4	4	4	4	3	3	4
3	3	3	3	3	2	3	4	3	3	2	2	4	2
4	3	4	3	4	3	4	4	2	4	2	2	3	2

a35	a36	a37	a38	a39	a40	a41	a42	a43	a44	a45	a46	a47	a48
5	5	5	5	5	5	1	1	5	1	5	1	5	5
4	3	5	5	4	4	2	2	5	2	5	1	3	4
5	2	3	5	4	5	3	1	5	1	5	1	5	5
4	3	3	3	3	3	1	1	4	1	4	1	1	1
4	3	5	4	4	4	5	3	4	2	4	3	3	3
2	3	3	3	3	3	2	4	4	2	3	3	3	5
4	3	3	3	3	3	3	3	3	2	4	2	4	4
2	2	2	4	4	4	2	2	4	2	4	2	4	4
3	3	3	3	3	3	3	3	3	3	3	3	3	3
4	4	4	4	4	4	5	4	4	3	4	4	4	4
2	2	4	5	2	5	2	1	3	1	4	1	5	5
3	3	3	3	3	3	3	3	3	3	3	3	3	3
3	4	5	4	4	4	5	3	4	2	4	2	4	3
3	2	3	4	3	4	3	3	4	3	4	2	4	4
4	2	4	4	3	5	2	2	4	2	4	2	4	4
4	1	4	4	3	4	3	2	4	2	4	2	3	3
3	4	5	5	2	3	3	3	3	3	4	3	3	3
2	4	3	4	4	5	5	3	4	2	4	3	4	4
4	3	4	4	4	3	3	2	4	2	5	2	3	4
3	5	3	4	3	3	3	4	3	3	4	4	3	3
4	2	5	5	5	5	5	2	5	2	5	3	4	4
5	5	5	5	5	5	5	1	5	1	5	1	5	5
4	4	4	4	4	4	4	4	4	4	4	4	4	4
4	3	5	5	4	5	1	1	5	1	5	1	5	5
3	2	4	4	4	3	1	2	3	3	4	2	3	3
4	3	5	5	4	4	2	1	5	2	4	2	4	4
3	3	4	4	5	4	3	3	3	1	4	2	4	4
2	4	5	4	4	4	5	2	4	3	4	4	4	3
4	1	4	4	4	4	2	1	4	1	4	1	4	4
3	1	4	4	3	4	2	1	3	3	4	1	3	4
3	4	4	4	5	4	5	3	4	2	4	1	4	4
3	4	5	5	4	5	2	1	2	1	5	3	5	5
3	4	4	4	4	4	4	2	4	2	4	2	4	3
2	2	4	4	5	2	3	5	3	5	5	3	3	3
1	4	5	5	2	5	1	1	4	1	5	1	4	5
3	3	5	5	5	5	5	2	4	3	5	4	3	5
4	3	4	4	3	3	4	3	4	2	4	2	4	4
3	3	3	3	3	3	3	3	3	3	3	3	3	3
2	4	4	4	4	4	4	2	4	2	4	2	4	4
3	3	3	3	3	3	3	3	3	3	3	3	3	3
3	3	4	4	4	4	4	3	2	3	3	3	3	2
3	3	3	3	3	3	3	3	3	3	3	3	3	3
1	4	4	4	4	3	2	2	3	4	4	3	3	3
3	3	5	5	5	5	5	1	3	1	5	1	4	5
2	4	4	4	3	3	3	2	4	2	4	3	4	4
4	1	5	4	3	4	1	2	4	1	5	1	4	4
4	4	4	4	4	4	2	2	2	2	4	4	4	4
5	5	5	5	5	5	5	5	5	5	4	4	4	4
4	2	4	4	4	4	3	1	4	1	3	2	4	3
3	2	5	5	4	5	4	1	5	1	5	1	4	4

4	4	5	5	5	5	5	1	5	1	5	1	5	5
2	2	2	3	3	3	3	4	3	2	4	4	2	4
3	4	4	4	2	4	4	2	4	2	4	2	4	4
4	4	4	4	3	3	1	2	4	2	4	2	4	4
4	3	4	5	4	5	3	1	4	1	5	2	4	5
2	2	3	5	3	3	4	2	3	3	4	4	4	4
2	5	5	5	4	4	4	2	4	4	4	4	3	4
3	4	3	5	3	4	4	3	4	3	4	3	4	4
3	2	3	2	3	2	2	2	2	1	3	1	3	3
4	3	4	5	4	4	4	2	4	2	4	2	3	4
2	3	3	4	4	3	5	3	3	3	4	2	3	3
4	2	4	5	2	4	1	1	4	1	5	1	5	5
3	3	5	5	3	3	5	1	4	1	5	2	3	4
5	1	3	5	5	5	5	1	5	3	5	1	5	5
3	4	3	4	3	3	1	2	3	3	3	3	3	3
4	2	4	4	3	4	3	2	4	2	4	2	4	4
2	4	4	5	3	4	4	2	4	3	4	2	3	3
4	2	4	4	3	4	5	2	4	2	4	1	4	4
3	1	4	4	3	3	5	3	4	2	3	2	3	3
3	3	4	5	3	3	3	1	4	1	5	2	3	4
4	3	4	4	3	4	2	2	4	2	4	2	4	4
4	5	4	5	5	5	1	1	3	1	5	4	5	5
5	1	5	5	3	5	1	1	5	1	5	1	5	5
2	3	5	5	3	5	5	1	5	1	5	1	3	3
3	2	3	3	3	3	2	2	3	2	3	2	3	3
2	3	3	4	4	4	4	3	3	3	3	3	3	3
3	1	4	5	3	5	3	1	4	1	4	1	4	5
3	1	3	4	3	3	1	2	5	2	3	1	3	3
3	3	5	5	3	5	3	1	3	1	5	1	5	5
3	3	4	5	3	3	3	2	3	2	3	1	3	3
4	3	4	4	4	3	3	3	3	3	4	3	3	4
4	1	3	4	2	5	3	2	5	1	4	1	4	4
2	4	4	4	3	4	4	2	3	3	4	4	3	3
2	4	5	5	3	5	5	3	5	3	5	3	3	3
3	2	5	5	3	5	3	1	5	1	5	1	5	5
3	3	3	3	3	3	3	3	3	3	3	3	3	3
3	3	5	5	5	5	5	1	3	2	5	3	4	4
2	2	4	4	3	4	3	2	4	2	4	2	4	3
3	3	3	3	3	3	3	3	3	3	4	3	3	3
5	1	5	5	2	5	1	1	5	1	5	1	5	5
2	4	4	4	4	4	3	3	4	4	4	2	3	3
3	1	2	5	3	4	1	3	5	1	5	1	3	4
2	4	4	4	4	4	4	2	4	2	5	3	4	4
5	5	4	4	4	5	3	2	4	4	5	1	5	5
5	1	5	5	5	5	1	1	5	1	5	1	5	5
4	4	5	5	4	4	3	2	5	2	4	1	5	5
3	2	3	4	5	3	2	3	3	2	4	2	3	3
4	3	4	4	4	3	5	3	4	4	4	2	4	4
3	3	3	5	5	3	4	4	4	4	4	3	3	3
3	3	3	3	3	3	3	2	3	2	3	3	3	3
3	3	4	4	3	4	2	1	4	1	4	2	4	4
4	3	4	5	4	4	4	2	4	2	4	3	5	4
4	3	4	5	5	4	2	2	3	2	4	3	4	4
4	2	5	5	3	5	1	1	5	1	5	1	4	4
2	1	4	4	2	5	2	2	3	2	4	2	4	4
4	3	5	5	4	5	1	1	5	1	5	1	5	5

3	1	5	5	3	5	5	1	5	1	5	1	5	5
3	2	3	3	3	3	3	3	3	3	3	2	3	3
3	2	4	3	2	4	5	4	4	3	4	2	4	3
3	3	5	5	4	5	4	1	4	1	5	4	5	5
3	2	4	4	4	3	3	3	3	3	4	3	3	3
3	3	3	3	3	3	3	3	3	3	3	3	3	3
4	4	4	4	2	4	2	1	5	1	4	1	4	4
4	1	5	5	2	4	1	1	4	2	4	1	4	4
4	4	3	4	3	4	4	2	4	3	4	3	3	4
4	2	4	4	4	3	3	2	4	2	4	2	3	4
2	4	5	5	3	5	5	2	4	2	5	2	4	5
3	3	4	4	4	4	2	2	4	2	4	3	3	3
3	3	3	4	3	4	3	3	3	3	3	3	3	3
3	4	5	4	3	4	4	2	4	3	4	4	4	4
1	5	4	4	4	4	4	3	2	4	3	3	3	3
3	4	4	5	4	4	2	2	3	3	4	3	3	3
5	5	5	5	5	5	5	5	5	5	5	5	5	5
3	4	5	5	3	3	3	3	3	3	4	2	3	4
5	1	5	5	1	5	1	1	5	1	5	1	1	5
2	5	5	5	5	5	5	3	3	2	5	5	3	3
3	3	5	5	4	3	5	3	4	2	5	3	4	4
4	3	3	3	4	4	4	3	2	3	3	3	4	3
3	1	5	5	4	4	3	1	4	1	4	1	3	4
4	4	4	4	3	4	4	2	4	2	4	2	4	4
3	2	4	5	2	4	3	2	4	3	4	2	3	3
3	3	4	4	2	4	4	2	3	2	4	3	4	4
4	1	5	5	3	4	1	1	5	1	5	1	5	5
3	4	4	5	4	4	4	2	4	2	5	4	4	4
1	3	5	5	5	5	5	3	1	5	5	1	5	5
4	2	5	4	5	3	4	4	4	1	4	1	3	3
3	3	3	3	3	3	2	3	3	3	3	2	3	3
3	4	2	3	4	3	3	3	2	3	4	4	3	3
4	2	3	3	2	3	3	2	3	2	2	2	3	3
1	5	4	1	5	2	5	2	1	4	4	5	2	2
2	4	4	4	4	4	3	2	3	2	4	3	3	3
4	3	5	5	5	5	4	1	4	3	4	1	4	4
3	4	4	4	5	3	1	1	3	1	3	1	5	4
2	4	5	5	4	3	4	4	4	4	5	2	3	3
3	3	3	3	3	3	3	3	3	3	3	3	3	3
3	5	5	5	5	3	5	3	3	3	4	2	1	3
3	3	3	3	3	3	4	3	3	3	3	2	3	3
3	4	4	5	3	4	3	2	3	2	4	2	3	3
2	3	3	4	3	3	3	3	3	3	3	3	3	3
3	4	4	5	3	4	3	2	3	2	4	3	4	4
3	2	4	4	4	3	3	3	4	2	4	2	3	3
3	3	4	4	3	4	2	1	2	1	2	2	3	3
3	3	5	5	5	5	4	2	3	3	3	3	5	5
3	3	3	4	4	4	3	2	3	2	4	2	3	3
3	3	5	5	5	5	1	1	5	3	3	3	5	5
5	5	5	5	5	5	5	5	5	5	5	5	5	5
5	3	5	5	3	5	3	1	4	1	5	2	5	5
3	4	5	5	5	5	4	3	5	2	5	1	5	5
2	3	3	3	4	4	3	3	3	3	3	3	3	3
4	2	5	5	5	5	4	1	4	1	5	1	5	5
3	3	3	3	3	4	5	2	2	3	3	4	3	3
4	3	4	3	4	3	4	4	3	2	4	2	4	3

4	2	4	4	2	4	2	2	4	2	4	2	4	4
4	2	5	5	4	5	2	2	4	1	5	1	4	5
3	3	3	4	4	4	4	4	4	2	4	3	4	4
3	3	4	3	3	3	3	2	3	3	4	3	3	4
5	5	5	5	5	5	5	2	5	2	5	2	5	5
2	5	5	5	5	5	1	1	3	2	5	2	5	5
2	4	5	5	5	5	2	1	3	1	5	2	4	4
3	3	3	3	3	3	3	3	3	3	3	3	3	3
3	4	4	4	4	3	4	3	3	3	3	2	2	3
3	5	1	5	5	5	5	1	1	1	5	1	5	5
4	4	5	5	5	5	4	1	5	1	5	3	5	5
3	3	5	4	3	3	3	2	3	2	4	2	4	4
1	2	5	5	4	5	2	2	5	3	5	1	3	5
3	5	5	5	5	4	3	2	4	2	5	3	3	4
3	1	4	5	4	4	3	1	4	1	5	1	3	4
3	3	3	3	3	3	3	3	3	3	3	3	3	3
5	5	5	5	1	5	1	1	5	1	5	1	5	5
3	3	3	3	3	3	3	3	3	3	3	3	3	3
4	4	4	4	2	4	4	2	4	1	5	2	5	5
2	5	5	5	4	5	5	2	4	5	5	2	3	3
4	3	4	3	4	3	5	3	3	3	3	3	3	3
1	4	4	5	4	4	2	2	4	4	4	2	4	4
3	3	5	4	4	3	2	2	3	3	4	4	3	3
3	3	3	3	3	3	3	3	3	3	3	3	3	3
3	3	3	3	3	3	4	3	3	3	3	3	3	3
3	4	4	4	4	4	3	2	3	2	4	4	4	4
3	4	5	5	4	5	5	1	5	1	5	1	5	5
4	3	4	5	3	4	3	3	4	4	5	2	3	4
3	3	3	3	3	3	3	3	3	3	3	3	3	3
1	3	4	4	4	4	5	2	4	3	4	1	4	4
3	3	2	4	3	3	4	2	4	2	4	2	3	4
4	5	5	5	5	5	5	1	5	2	5	3	5	5
5	1	5	5	2	5	2	1	5	1	5	1	5	5
5	1	5	5	3	5	1	1	5	1	5	1	5	5
4	2	5	5	5	3	3	2	2	1	5	2	4	4
2	4	4	4	3	4	4	2	4	2	4	2	4	5
3	4	4	4	2	1	4	2	4	2	4	2	2	3
3	3	4	4	3	4	4	1	4	3	4	2	4	4
4	4	4	4	4	3	3	2	2	2	4	2	3	3
3	3	4	4	3	4	2	2	3	2	4	2	4	4
3	3	3	3	3	3	3	3	3	3	3	3	3	3
1	1	5	5	2	5	3	1	4	1	5	2	5	5
2	2	4	5	4	5	3	3	3	4	4	4	4	4
2	3	4	5	4	4	5	2	1	2	4	1	4	4
4	4	4	4	3	4	5	2	3	3	3	2	3	3
3	4	2	4	4	4	4	1	5	1	4	4	4	4
3	3	3	4	3	3	2	2	4	2	4	2	4	4
1	4	4	4	3	4	3	2	3	2	4	4	3	3
3	3	4	4	4	4	3	2	3	2	4	2	4	4
2	3	4	4	3	4	3	2	4	2	4	2	4	4
2	4	4	4	3	4	5	3	2	4	4	4	4	4
5	3	5	5	2	5	1	1	5	1	5	2	5	5
4	3	4	4	2	4	4	2	4	2	4	2	4	4
3	4	4	4	4	4	4	3	3	3	4	3	3	3
3	2	4	4	2	4	3	2	4	2	4	2	4	4
2	4	5	5	4	4	3	3	3	2	3	3	3	3

3	4	4	4	4	4	4	3	4	3	4	3	4	4
3	4	4	4	4	3	3	3	4	3	3	3	3	3
3	2	5	5	5	5	1	1	3	1	4	3	4	4
2	4	4	4	4	3	3	2	3	4	3	4	2	3
3	3	3	5	3	5	5	3	3	3	5	3	5	5
5	3	4	5	4	5	1	1	5	1	5	1	5	5
3	4	5	5	4	4	3	2	4	2	4	2	3	4
4	4	5	5	5	5	1	1	4	2	5	2	4	4
5	4	5	5	3	5	4	2	5	2	5	2	5	5
4	2	2	4	2	4	3	3	3	3	3	3	3	3
3	4	4	5	4	4	3	1	4	2	5	2	4	5
4	1	5	5	3	4	5	1	5	1	5	3	5	5
4	3	4	4	3	4	2	1	3	2	4	1	4	4
1	4	4	4	4	4	5	1	3	1	4	4	4	4
2	2	4	5	3	4	2	2	4	2	4	2	4	4
3	1	5	5	3	4	1	1	5	1	5	1	5	4
3	3	5	5	5	5	1	1	1	1	1	1	5	5
3	3	3	3	3	3	3	3	3	3	3	3	3	3
3	3	3	3	3	3	3	3	3	3	4	2	3	4
2	4	5	5	5	4	1	1	5	1	5	2	4	5
5	4	5	5	4	4	3	1	4	1	5	1	4	4
3	5	5	5	5	3	3	3	4	3	5	4	4	4
4	2	5	5	4	5	1	1	4	1	4	1	5	5
3	2	4	4	3	4	3	2	4	2	4	2	4	4
3	4	5	5	5	5	5	1	4	1	4	5	4	4
3	4	4	4	4	4	4	3	4	2	4	3	4	4
4	2	5	5	4	5	2	1	5	1	5	1	5	5
2	4	4	4	4	4	4	4	2	2	3	4	3	3
4	3	4	4	2	4	2	2	4	2	4	2	4	4
3	4	5	5	3	4	3	1	4	1	4	3	4	4
3	5	5	5	4	4	5	3	3	2	4	4	4	4
1	4	4	4	4	4	1	3	4	3	4	2	4	4
3	4	4	4	4	4	3	1	4	1	4	1	4	4
2	3	4	5	4	4	3	2	2	2	5	3	3	3
3	3	5	5	2	5	3	1	4	1	5	1	5	5
5	1	5	5	1	5	2	2	5	2	5	5	5	5
2	3	5	5	4	3	4	3	4	2	5	2	3	3
3	2	5	5	3	5	1	1	5	1	5	1	5	5
2	4	5	5	3	4	5	1	4	2	5	1	4	4
4	1	5	5	4	5	2	1	4	1	5	1	4	5
3	4	4	5	4	4	5	2	4	3	5	4	4	4
4	3	3	5	1	3	1	1	3	3	5	3	4	4
5	1	5	5	1	5	1	1	5	1	5	1	5	5
3	3	4	4	3	4	4	2	4	2	4	2	4	4
2	4	5	5	5	5	5	3	4	3	5	4	4	4
1	4	5	5	5	5	3	2	2	4	5	5	5	5
1	5	5	5	2	5	5	2	5	3	4	3	4	4
2	4	4	3	3	2	2	3	4	3	4	3	2	2
2	3	3	3	2	3	4	2	3	3	3	2	3	2
5	5	5	5	5	5	1	1	5	1	5	5	5	5
2	5	5	5	5	5	4	4	2	2	4	2	3	3
3	3	4	4	3	4	3	3	4	2	4	2	4	4
3	3	5	5	4	4	5	1	5	1	5	1	5	5
4	5	5	5	4	4	4	2	4	2	5	4	4	4
4	4	4	4	4	4	2	2	4	2	4	3	4	4
3	5	5	5	2	4	3	2	3	2	4	2	3	4

5	5	5	5	1	5	5	1	5	1	5	1	5	5
3	3	4	4	2	4	3	1	3	2	4	2	4	4
4	2	5	5	2	5	4	1	2	1	5	1	5	5
2	3	4	4	4	4	4	2	3	3	4	3	3	3
5	1	5	5	4	5	1	1	5	1	5	1	5	5
4	4	4	4	4	4	2	2	4	2	4	2	4	4
3	3	3	3	3	3	3	3	3	3	3	3	3	3
2	3	4	4	5	3	3	4	2	4	3	2	4	3
2	4	4	4	3	3	3	3	3	3	3	4	3	3
4	4	5	5	3	5	3	1	5	1	5	1	5	5
3	3	3	3	3	3	4	4	3	2	3	3	3	3
5	4	5	5	5	5	1	1	5	1	5	1	5	5
1	5	5	5	5	5	3	2	4	3	5	5	4	5
1	5	5	5	5	3	1	1	4	1	5	1	3	5
4	4	4	5	4	5	4	5	4	5	4	5	5	4
5	5	5	5	5	5	5	5	5	5	5	5	5	5
3	3	5	5	4	4	5	1	5	4	5	1	5	5
3	3	3	3	3	3	3	3	3	3	3	3	3	3
2	3	3	3	3	3	2	3	3	3	3	3	3	3
2	3	4	5	5	4	4	1	4	1	4	4	4	4
4	5	5	5	5	5	4	2	2	2	4	2	4	4
2	4	3	5	3	4	2	4	2	2	2	3	2	4
4	2	4	4	4	4	4	2	4	2	4	3	3	4
4	4	4	4	5	5	5	2	4	2	4	5	5	5
3	4	4	4	3	4	2	2	4	2	4	2	4	4
4	4	4	4	2	4	2	2	4	2	4	2	4	4
3	4	4	4	4	4	3	2	3	3	3	3	4	3
2	5	3	4	4	4	5	3	3	3	3	3	3	3
3	4	4	4	2	4	2	2	2	2	4	2	4	4
4	1	5	5	2	5	2	2	5	2	5	2	5	5
3	3	3	3	2	3	1	1	4	1	4	1	4	4
2	3	4	4	3	4	3	2	4	2	4	2	4	4
3	1	5	5	1	5	1	1	5	1	5	1	5	5
4	3	3	3	3	3	3	3	3	2	4	3	4	4
4	4	5	5	4	5	4	1	4	1	5	4	5	5
3	4	5	5	3	5	5	1	4	1	5	2	5	5
3	4	5	5	3	5	3	1	5	1	5	1	5	5
4	1	4	4	2	4	4	2	4	2	3	2	3	4
3	1	4	5	2	4	3	2	4	1	5	2	4	4
3	1	4	5	2	4	3	2	4	1	5	2	4	4
2	2	4	5	2	4	2	1	4	1	5	2	3	4
5	1	5	5	1	5	5	1	5	1	5	1	5	5
3	3	4	5	5	4	4	2	3	3	4	4	3	3
1	1	5	5	3	5	4	1	5	1	5	1	5	5
3	3	5	5	5	5	5	2	3	2	5	1	4	4
4	1	5	5	5	5	2	1	5	1	5	1	5	5
5	5	5	5	5	5	5	5	5	5	5	5	5	5
3	2	4	5	2	5	3	1	4	1	5	1	5	5
3	4	4	4	3	3	5	2	3	2	5	2	4	4
3	2	5	5	3	4	4	1	4	1	5	1	4	5
3	3	3	3	3	3	3	3	3	3	3	3	3	3
5	1	1	1	1	1	1	5	4	1	1	1	1	1
2	2	3	4	3	3	3	2	4	2	4	2	3	3
3	3	4	5	4	4	4	1	4	2	5	2	4	4
3	3	3	3	3	3	3	3	3	3	3	3	3	3
3	5	5	5	5	5	4	1	3	1	5	3	5	5

5	1	5	5	1	5	1	1	5	1	5	1	5	5
3	3	4	4	2	4	3	2	3	2	5	2	4	4
4	1	3	3	3	4	1	1	3	3	3	1	3	3
3	3	4	4	3	4	3	1	4	1	4	3	4	4
4	2	4	4	4	4	4	2	4	2	4	2	4	4
2	4	3	4	3	3	4	3	4	3	4	4	3	3
4	2	4	4	3	4	4	2	4	2	4	2	4	4
3	3	3	3	3	3	5	3	3	3	3	3	3	3
3	3	4	4	3	4	2	1	3	2	4	2	4	4
3	3	3	3	3	3	3	3	3	3	3	3	3	3
3	4	4	4	3	4	4	2	3	2	4	3	4	4
5	4	5	5	5	5	1	1	5	1	5	4	5	5
4	4	4	4	4	4	4	4	4	4	4	4	4	4
4	4	4	4	4	4	4	4	4	4	4	4	4	4
4	1	5	5	5	5	4	1	3	1	5	1	5	5
3	3	4	4	2	4	5	1	4	1	4	2	5	5
4	3	5	5	3	5	3	1	5	1	5	1	5	5
4	2	5	5	4	5	4	1	5	1	5	1	5	5
3	3	3	4	4	4	4	2	4	2	4	2	3	3
5	5	5	5	1	5	1	1	5	1	5	1	5	5
4	4	5	5	4	4	5	1	5	2	5	4	5	5
3	4	4	4	4	4	3	2	4	2	4	2	4	4
4	2	3	4	4	4	4	2	4	2	4	2	4	4
2	4	4	5	4	5	4	2	4	2	4	2	3	4
5	1	5	5	1	5	1	1	5	1	5	1	5	5
4	1	4	4	4	5	4	1	3	1	4	1	4	4
3	3	5	5	1	5	2	1	4	1	5	1	3	4
3	3	5	5	3	4	2	2	4	2	4	3	4	4
3	3	5	5	3	1	2	1	5	1	5	1	5	5
3	4	4	4	4	3	4	2	4	2	4	2	3	3
4	2	4	4	4	3	3	3	4	3	4	2	3	4
1	5	2	2	5	4	4	3	3	4	4	3	3	3
4	3	4	5	4	3	2	2	4	2	4	2	4	4
4	4	4	4	3	4	4	1	4	1	4	1	4	4
2	2	3	3	3	3	3	2	3	2	3	2	3	3
5	5	5	4	3	4	4	2	4	2	4	4	2	4
4	5	5	5	4	5	1	1	4	4	5	4	4	5
4	3	4	4	2	4	4	2	3	3	4	3	4	4
3	3	4	4	4	4	3	2	4	2	4	2	4	4
3	3	3	5	4	5	4	1	5	2	4	2	4	4
4	4	5	5	5	5	3	3	4	3	5	3	5	5
2	5	5	5	5	5	4	3	1	4	4	2	4	4
3	4	5	5	5	5	3	3	3	3	5	4	3	4
3	4	4	4	4	4	4	3	4	2	4	2	3	4
5	4	5	5	3	5	4	1	3	2	5	4	5	5
4	4	5	5	5	5	4	1	5	1	5	3	5	5
4	2	4	4	5	4	1	1	4	1	5	2	4	5
1	4	2	4	3	3	2	2	2	2	3	3	2	3
5	1	5	5	3	5	1	1	5	1	5	1	5	5
3	2	4	4	4	4	3	3	4	3	4	2	4	4
3	4	3	5	4	4	3	1	4	1	4	3	3	4
4	1	5	5	5	3	2	2	4	2	4	2	4	4
5	5	5	5	5	5	5	5	5	5	5	5	5	5
2	4	4	4	4	4	2	2	4	3	4	2	4	3
5	2	5	5	2	5	2	2	5	2	5	2	5	5
3	3	3	3	3	3	3	3	3	3	3	3	3	3

3	3	3	4	3	3	3	3	3	3	3	3	3	3
2	4	4	5	4	5	3	1	4	1	5	4	4	5
5	1	5	5	1	5	1	1	5	1	5	1	5	5
3	4	5	5	5	5	4	1	3	2	5	4	5	5
3	3	3	3	3	3	3	3	3	3	3	3	3	3
4	1	4	4	4	4	5	2	4	2	4	1	4	4
5	3	5	5	3	3	3	3	5	3	3	3	5	5
2	4	4	5	3	4	3	2	3	2	4	3	3	4
3	1	5	5	1	5	1	1	5	1	5	1	5	5
3	3	3	3	3	3	3	3	3	3	3	3	3	3
2	3	4	4	5	5	3	2	4	2	5	3	4	4
3	2	4	4	4	4	1	2	4	2	4	2	4	4
3	4	4	5	3	3	1	1	3	2	4	2	2	3
2	5	5	5	5	5	5	1	5	1	5	5	5	5
2	3	2	3	3	3	4	3	3	3	3	3	3	3
3	3	3	3	3	3	3	3	3	3	3	3	3	3
1	1	5	5	4	4	4	2	3	2	4	3	3	4
4	3	5	5	4	5	1	1	5	1	5	1	5	5
3	4	4	4	4	4	2	1	3	1	5	2	4	4
4	4	4	4	4	4	4	2	4	2	4	4	4	4
3	5	5	5	5	5	1	1	3	1	5	5	5	5
3	4	3	3	2	3	2	2	4	3	4	2	3	3
3	3	3	3	3	3	4	3	4	4	4	4	4	4
4	3	5	5	5	5	3	1	5	1	5	3	5	5
2	3	4	4	4	4	3	2	3	2	4	2	3	4
3	4	4	4	3	4	3	1	3	2	4	3	4	4
5	1	5	5	4	4	4	1	5	1	5	1	5	1
3	3	4	3	3	4	4	3	3	3	4	4	3	3
3	5	3	3	3	3	3	3	3	3	3	5	3	3
1	5	5	5	5	5	5	2	2	5	5	5	3	4
3	2	3	2	4	2	3	2	4	3	2	3	4	3
1	2	5	5	4	5	5	1	5	1	5	2	5	5
4	2	5	5	4	5	2	1	4	1	5	1	5	5
2	2	5	5	4	5	4	1	3	2	5	3	4	4
2	4	4	5	5	4	5	3	3	2	4	3	3	3
3	2	4	4	4	4	2	2	4	2	5	2	4	4
2	4	5	5	3	5	3	1	3	2	5	4	4	4
5	5	5	5	5	5	5	5	5	5	5	5	5	5
3	4	4	5	4	4	2	2	4	2	4	2	4	4
3	2	4	4	4	4	5	4	4	2	5	2	4	4
3	4	4	4	4	4	2	2	2	3	4	4	4	4
5	1	5	5	5	5	5	1	5	1	5	1	5	5
4	3	5	5	5	5	1	1	5	1	5	1	5	5
2	4	5	5	3	5	2	2	3	2	5	2	4	4
4	4	4	4	2	4	2	2	4	3	4	2	4	4
3	3	4	4	4	2	4	2	4	2	4	3	3	4
3	1	5	5	5	5	3	1	5	1	5	1	3	5
2	2	2	2	2	2	2	2	4	2	2	2	2	2
3	3	5	5	4	4	4	1	5	2	5	2	5	5
5	1	3	5	4	5	3	1	5	1	5	1	5	5
3	4	4	5	3	4	4	2	3	3	4	3	3	3
3	4	4	4	4	4	3	2	4	3	4	3	3	3
1	1	5	5	2	5	4	1	5	1	5	1	5	5
3	4	5	5	5	5	5	1	5	1	5	2	4	4
3	1	4	5	3	5	2	1	5	1	5	1	5	5
4	2	4	4	4	5	4	2	4	1	5	1	4	4

3	4	3	5	4	3	4	2	4	2	5	2	4	4
3	1	5	5	4	5	3	1	5	1	5	1	5	5
1	4	4	5	3	3	1	1	3	1	4	1	4	4
3	3	3	4	4	4	5	2	3	2	4	3	3	3
3	2	4	4	3	4	2	2	4	2	4	2	4	4
3	4	4	4	4	4	3	2	4	3	4	3	3	4
4	4	4	4	4	4	4	2	4	2	4	2	4	4
3	2	4	4	2	4	2	2	4	2	4	2	4	4
3	3	3	3	3	3	3	3	3	3	3	3	3	3
1	5	5	5	5	5	5	1	3	1	5	1	5	5
5	2	5	5	4	5	2	1	5	1	5	2	5	5
3	3	5	5	5	5	4	1	5	2	5	4	4	4
5	1	5	5	4	5	1	1	5	1	5	1	5	5
3	3	3	3	3	3	3	3	3	3	3	3	3	3
5	1	5	5	1	5	1	1	5	1	5	1	5	5
4	4	4	4	4	4	4	2	4	2	4	2	4	4
3	3	4	5	5	5	3	4	3	2	3	1	3	3
3	3	3	3	3	4	3	2	4	2	4	1	2	4
3	3	3	3	3	3	3	3	3	3	3	3	3	3
1	1	5	5	5	5	5	1	5	1	5	1	5	5
4	5	5	4	3	4	4	4	3	2	4	3	4	4
3	2	4	4	2	2	2	2	4	3	4	2	4	4
3	4	5	5	5	5	4	1	4	1	5	4	4	4
4	2	4	4	4	4	4	2	4	2	4	2	4	4
3	3	3	3	3	3	3	3	4	3	3	3	3	3
3	3	4	4	4	4	4	2	4	2	4	2	3	3
2	2	5	5	3	3	5	2	3	3	4	2	3	2
3	3	3	3	3	3	3	3	3	3	3	3	3	3
3	1	5	5	5	3	4	2	3	2	4	1	4	4
3	2	4	4	3	4	3	2	4	2	4	2	3	3
4	3	4	4	4	4	4	2	3	2	4	3	4	4
2	3	4	4	4	4	4	2	3	3	3	2	3	3
3	3	5	5	5	5	5	1	3	2	5	3	5	5
3	3	5	5	5	4	4	2	4	2	4	2	3	3
2	2	3	3	3	3	1	3	3	2	3	2	3	3
1	2	3	5	4	2	4	2	4	3	3	2	3	4
4	2	5	5	3	4	3	1	4	1	5	2	5	5
3	4	4	4	4	4	3	2	4	2	4	3	4	4
2	3	3	3	3	3	3	4	3	3	3	3	3	3
4	3	4	4	2	4	4	1	5	2	4	2	4	5
3	3	3	3	3	3	4	4	4	4	4	4	4	4
3	3	5	5	2	5	2	2	4	2	4	2	4	4
3	3	5	4	4	4	1	1	3	1	4	1	5	5
4	2	4	4	4	4	2	2	3	2	4	2	4	4
3	1	5	5	5	4	3	2	4	2	4	2	4	4
3	4	4	4	4	4	4	2	2	2	4	1	4	4
4	3	5	4	2	5	4	2	5	1	5	2	5	5
5	1	5	5	1	5	1	1	5	1	5	1	5	5
2	2	4	4	2	5	5	1	3	3	5	2	3	3
5	2	5	5	4	5	2	1	5	1	5	1	5	5
3	2	4	4	3	4	3	2	4	2	4	2	3	3
3	3	5	5	3	4	2	2	3	1	5	1	4	5
3	2	5	5	4	4	4	3	5	2	5	1	4	4
2	3	4	4	3	4	3	2	3	2	3	2	4	3
2	4	4	4	4	4	4	2	4	3	4	2	4	4
3	3	3	3	3	3	3	3	3	3	3	3	3	3

3	4	4	4	2	4	4	3	4	4	4	2	4	4
3	3	5	5	4	4	4	1	3	2	5	1	5	4
3	3	3	3	3	3	3	3	3	3	3	3	3	3
2	3	3	3	3	3	3	2	3	2	3	2	3	3
3	5	5	5	3	5	3	1	3	1	5	3	5	5
4	1	4	4	3	4	4	1	4	1	4	1	4	4
5	2	5	5	5	5	2	2	5	2	5	2	5	5
3	3	3	3	3	3	3	3	3	3	3	3	3	3
1	5	5	5	3	3	4	3	4	4	4	4	3	3
4	4	5	5	1	5	1	1	5	1	5	1	5	5
4	1	5	4	4	4	3	2	4	2	4	1	4	4
2	5	5	5	5	5	5	1	3	3	5	4	5	5
5	2	5	5	3	5	5	4	4	3	5	5	5	5
4	3	4	5	4	4	4	1	4	1	5	1	5	5
3	4	5	4	5	4	4	4	3	3	4	4	4	4
3	5	5	5	5	5	5	2	3	2	5	4	3	3
3	4	3	4	3	3	4	2	4	3	4	4	3	4
3	3	4	4	3	4	5	4	4	4	4	2	3	3
4	3	5	5	4	5	4	1	4	1	5	2	4	3
4	5	4	5	4	5	4	4	4	4	5	4	4	4
2	2	4	4	4	4	4	3	4	4	3	2	3	3
3	3	3	2	3	3	3	4	3	3	3	3	3	3
5	1	5	5	5	5	1	1	5	1	5	1	5	5
3	4	4	4	4	4	3	2	4	2	4	3	4	4
5	1	5	5	1	5	2	1	5	1	5	1	5	5
4	3	4	4	4	4	2	2	4	2	4	2	4	4
3	4	5	5	3	5	4	2	4	1	5	1	4	4
3	3	3	3	3	3	3	3	3	3	3	3	3	3
5	1	5	5	3	5	3	2	5	1	5	1	5	5
3	3	4	5	4	4	4	2	4	2	4	2	4	4
3	4	5	5	4	5	5	1	4	2	5	3	4	4
3	3	3	3	3	3	3	3	3	3	3	3	3	3
3	1	4	4	3	3	2	2	3	2	3	1	3	4
3	1	4	5	3	4	1	1	5	1	5	1	5	5
5	5	5	5	5	5	1	1	5	1	5	5	5	5
3	2	3	4	3	3	2	2	5	2	3	1	3	3
4	3	4	4	3	4	3	2	4	2	4	2	4	4
5	1	5	5	5	5	1	1	5	1	5	1	5	5
3	3	5	4	3	3	5	2	4	2	4	3	3	3
5	4	4	5	1	4	4	1	4	1	4	2	5	5
3	4	4	4	4	4	3	2	4	3	4	3	4	4
4	4	5	5	4	5	2	2	4	1	5	3	5	5
4	1	5	5	5	5	1	1	5	1	5	1	5	5
3	3	3	4	3	3	2	2	4	2	3	2	3	3
3	4	4	4	3	4	4	2	2	2	4	3	4	4
3	3	3	3	3	3	3	3	3	3	3	3	3	3
4	3	5	5	4	5	3	1	4	1	5	1	5	5
3	5	5	5	3	5	4	1	5	2	5	3	5	5
3	4	4	4	4	4	4	1	4	3	4	1	4	4
3	1	5	5	5	5	4	1	5	1	5	1	5	5
3	4	5	5	5	5	1	1	5	1	5	1	5	5
5	1	5	5	3	5	1	1	5	1	5	1	5	5
5	3	5	5	4	4	3	2	5	2	5	2	5	5
5	1	4	5	5	3	1	1	5	1	5	3	3	4
3	3	4	4	4	4	3	1	4	2	4	3	4	4
3	5	5	5	5	5	2	2	3	2	4	2	4	4

3	4	5	4	5	4	4	3	3	2	3	3	3	3
5	5	5	5	5	5	5	2	4	2	5	5	5	5
1	1	5	5	1	5	1	1	4	1	5	1	5	5
4	1	5	5	2	5	1	1	5	1	5	1	5	5
4	3	4	3	4	3	4	4	3	4	3	4	4	3
3	3	3	3	3	3	3	3	3	3	3	3	3	3
3	3	3	3	3	3	3	3	3	3	3	3	3	3
5	4	5	5	5	5	1	1	5	1	5	1	5	5
3	3	3	3	3	3	3	3	3	3	3	3	3	3
3	4	4	4	4	4	4	3	4	2	4	4	4	4
4	3	5	5	5	5	5	1	5	3	5	4	5	5
1	2	5	5	5	4	5	1	5	1	3	3	5	5
4	2	4	4	3	4	3	2	4	2	4	2	4	4
3	4	4	4	4	4	4	2	4	2	4	2	4	4
3	3	5	5	3	5	5	2	3	2	5	1	4	3
3	2	4	4	3	4	3	2	4	2	4	2	4	4
3	3	5	5	5	4	1	1	4	1	4	3	4	4
2	4	4	4	4	4	4	2	4	3	3	3	3	3
3	3	3	3	3	3	3	3	3	3	3	3	3	3
3	3	4	3	3	3	3	3	3	2	3	2	3	3
4	2	4	4	4	4	1	1	4	3	4	2	3	4
3	3	4	4	2	4	4	2	3	2	4	2	4	4
4	3	4	4	4	4	3	2	3	2	4	4	4	4
3	1	5	5	2	5	3	1	4	2	5	1	5	5
5	1	5	5	2	5	1	1	5	1	5	1	5	5
3	4	4	4	5	3	4	2	4	3	4	2	3	4
3	3	3	3	3	3	3	3	3	3	3	3	3	4
3	4	4	4	4	4	5	3	4	2	4	5	4	3
3	3	4	4	2	4	1	2	4	1	4	2	4	4
5	1	5	5	5	5	5	4	4	2	5	1	3	5
3	2	4	4	3	3	2	2	2	2	4	2	3	3
5	5	5	5	5	5	4	1	5	2	5	4	4	5
3	3	5	5	3	5	5	1	5	1	5	5	5	5
3	3	3	3	3	3	3	3	3	3	3	3	3	3
3	5	5	5	5	5	5	1	5	1	5	1	5	5
4	4	4	4	4	3	4	3	4	3	4	3	4	4
3	1	5	5	5	5	1	1	5	1	5	1	5	5
3	4	5	4	5	4	3	2	4	2	4	1	3	3
3	3	5	5	3	4	4	2	4	3	4	2	4	5
4	4	3	4	4	4	3	4	4	2	4	2	4	4
3	4	4	5	3	4	4	3	4	3	4	3	3	4
5	3	5	5	3	5	4	1	5	1	5	2	5	5
3	4	4	4	4	4	4	2	3	2	4	3	4	4
5	2	3	4	4	4	5	1	4	1	4	1	4	4
4	2	4	4	4	4	4	2	4	2	4	2	4	4
3	3	3	4	3	3	3	2	4	3	3	4	2	3
3	3	3	5	3	3	4	4	3	4	3	3	4	4
3	4	4	5	3	5	5	1	4	3	5	5	3	3
3	4	4	4	4	4	4	2	4	2	4	3	4	4
5	1	5	5	5	5	1	1	5	1	5	1	5	5
3	4	5	5	5	5	1	2	5	3	5	3	4	4
3	3	4	5	1	5	3	2	4	2	4	2	5	4
4	4	5	5	3	5	3	1	5	1	5	1	5	5
3	3	3	3	3	3	3	3	3	3	3	3	3	3
5	1	5	5	5	5	5	1	5	1	5	5	5	5
4	2	4	4	3	4	3	2	4	2	4	2	4	4

3	1	5	5	1	5	3	1	5	1	5	1	5	5
2	2	4	5	3	4	5	2	5	2	5	2	4	5
3	4	4	5	4	3	3	3	3	3	4	3	3	4
3	5	5	5	3	5	3	1	5	1	5	1	5	5
4	3	5	5	3	4	4	2	4	2	4	1	4	4
3	3	5	5	3	5	3	1	3	1	5	1	5	5
3	3	3	3	3	3	3	4	3	3	3	3	3	3
3	2	3	4	3	2	3	4	3	3	2	4	3	2
3	3	3	3	3	3	3	3	3	3	3	3	3	3
5	5	5	5	5	5	5	1	5	1	5	1	5	5
4	5	5	5	5	5	1	2	4	2	5	4	4	4
2	4	5	5	5	4	5	2	4	3	4	5	3	4
4	1	5	5	2	5	1	1	4	1	5	1	4	5
3	3	3	4	3	4	4	3	4	3	4	3	3	3
3	3	3	3	3	3	3	3	5	4	5	5	4	4
4	4	5	5	4	4	4	1	3	1	4	4	4	4
3	4	5	5	4	5	5	1	5	1	5	4	4	4
3	3	3	3	3	3	5	3	2	3	3	4	3	3
3	3	3	3	3	3	3	3	3	3	3	3	3	3
4	2	5	4	1	4	1	1	5	5	5	1	5	5
3	5	5	5	5	5	1	5	5	5	5	1	5	5
5	5	5	5	5	5	5	1	5	4	5	5	5	5
2	5	5	5	5	4	5	2	3	3	4	4	3	3
3	3	3	3	3	3	3	3	3	3	3	3	3	3
3	3	3	4	3	4	3	2	3	3	3	3	3	3
3	1	4	5	1	4	1	1	5	1	5	1	4	5
3	4	5	5	4	5	4	1	3	1	5	4	5	5
2	3	3	4	5	3	3	2	3	3	2	2	3	3
4	2	4	4	3	4	1	1	4	1	4	2	4	4
2	4	4	4	4	4	4	3	4	2	4	3	4	4
3	5	5	5	5	5	3	2	4	2	5	3	5	5
4	2	4	4	4	4	2	2	4	2	4	2	4	4
3	1	3	3	3	3	3	1	3	1	3	1	5	5
4	4	3	3	5	2	4	3	4	2	4	3	3	4
4	2	4	4	4	4	3	2	4	2	4	2	4	4
3	3	3	3	3	3	3	3	3	3	3	3	3	3
3	4	4	5	4	3	3	3	4	3	4	2	3	3
1	4	4	4	2	4	5	4	3	4	4	5	2	2
4	4	5	5	2	5	3	1	3	1	5	2	5	5
5	5	5	5	5	5	5	5	5	5	5	5	5	5
3	3	4	4	4	4	3	2	4	2	4	2	3	4
3	5	5	5	4	4	2	1	4	2	4	3	3	4
3	3	3	3	3	3	3	3	3	3	3	3	3	3
1	5	5	5	5	5	5	1	2	4	5	2	5	5
3	3	3	3	3	3	3	3	3	3	3	3	3	3
3	2	4	4	3	4	3	2	3	2	4	2	4	4
4	3	4	4	4	3	4	3	4	3	4	3	4	4
3	3	3	3	3	3	3	3	3	3	3	3	3	3
4	3	5	4	4	4	5	3	3	3	4	5	4	4
5	1	5	5	1	5	1	1	5	1	5	1	5	5
2	4	4	4	4	4	4	2	4	3	4	3	3	4
4	2	5	5	5	4	4	1	5	1	5	3	4	4
3	3	3	3	3	3	3	3	3	3	3	3	3	3
5	2	5	5	2	5	2	1	5	1	5	1	5	5
1	4	5	5	5	5	5	1	2	4	5	4	2	2
5	1	5	5	1	5	1	1	5	1	5	1	5	5

3	4	4	4	4	4	5	2	2	3	4	4	4	4
5	1	5	5	3	5	3	1	5	1	5	1	5	5
4	4	4	5	3	5	5	1	4	2	5	2	5	5
3	3	2	3	4	3	3	3	3	2	4	2	3	3
4	2	5	5	4	4	4	2	4	4	5	2	4	4
2	2	4	4	5	3	3	1	3	2	4	3	2	2
3	4	4	5	4	4	1	1	4	1	5	4	2	5
2	5	5	5	5	5	5	2	3	3	4	5	2	3
4	4	4	5	5	5	5	2	2	2	5	3	4	4
4	3	4	4	4	3	3	3	3	3	3	3	4	3
3	2	4	4	4	4	4	2	4	2	4	2	4	4
4	2	4	5	4	5	2	1	4	1	5	1	5	5
1	5	4	5	3	5	5	3	3	2	4	3	4	4
3	3	3	4	4	4	3	3	4	2	4	2	4	4
4	3	5	5	4	5	1	1	5	1	5	1	4	4
3	2	5	5	3	5	2	2	4	3	5	1	4	4
4	4	4	5	3	5	4	1	4	2	4	4	4	5
2	2	4	5	4	3	2	4	3	3	4	2	2	3
5	5	5	5	5	5	1	1	1	1	1	1	1	1
2	3	4	4	3	4	1	1	4	1	4	1	4	4
1	5	5	5	2	4	5	2	2	2	2	5	2	2
4	4	4	4	4	4	4	2	4	2	4	2	4	4
5	1	5	5	5	5	1	1	5	1	5	1	5	5
3	4	4	4	4	4	3	1	4	2	4	2	4	4
3	2	4	4	2	4	2	1	4	2	4	1	4	4
5	5	5	5	5	5	5	1	5	1	5	5	5	5
5	5	5	5	5	5	5	5	5	5	5	5	5	5
5	5	5	5	1	5	5	1	5	1	5	1	5	5
3	5	5	5	5	5	5	2	4	3	5	4	3	4
3	4	4	4	4	4	3	2	3	2	4	3	3	4
4	4	4	5	3	4	3	2	4	3	4	2	4	4
3	3	4	4	2	5	4	1	3	1	4	2	5	2
4	2	4	5	3	5	2	2	4	2	4	1	4	5
3	4	4	4	4	4	4	2	4	3	4	4	3	3
4	4	4	5	3	5	3	1	5	1	5	2	5	5
3	3	3	3	3	3	3	3	3	3	3	3	3	3
3	4	4	4	4	4	3	1	3	1	4	4	4	4
5	5	5	5	2	5	1	5	5	1	5	2	5	5
3	3	3	3	3	3	3	3	3	3	3	3	3	3
3	3	4	4	4	4	3	2	4	2	4	2	4	4
3	1	5	5	2	5	5	1	4	1	5	1	5	5
3	4	4	4	4	4	4	3	3	3	4	4	3	3
3	3	4	4	3	5	4	2	3	2	3	2	4	5
4	3	4	5	3	5	3	3	4	3	4	3	5	5
4	5	5	5	4	5	1	1	5	2	5	5	5	5
2	5	5	5	5	4	5	2	2	4	5	4	4	4
4	4	4	4	3	4	4	2	4	2	4	3	4	4
3	2	4	4	4	4	3	2	4	2	4	3	4	4
5	5	5	5	5	5	5	5	5	5	5	5	5	5
2	4	4	4	4	4	1	1	4	2	4	4	4	4
3	3	4	5	3	3	3	2	3	2	3	2	3	4
3	2	5	5	5	5	4	1	3	2	4	2	4	4
3	3	3	3	3	3	3	3	3	3	3	3	3	3
3	4	4	4	4	4	4	3	4	3	4	4	4	4
4	4	4	4	4	4	4	1	4	1	4	1	4	4
2	3	4	4	2	2	3	3	4	2	4	4	4	4

3	3	3	3	3	3	3	3	3	3	3	3	3	3
2	3	3	4	4	3	3	3	4	3	4	3	4	4
3	4	4	4	4	4	4	3	4	3	5	4	4	3
3	3	3	3	3	3	3	3	3	3	3	3	3	3
4	3	4	4	4	4	3	2	4	2	4	3	4	4
4	5	5	5	4	5	4	1	4	1	5	1	5	5
4	1	5	5	2	5	5	1	4	1	5	1	4	4
3	5	5	5	3	5	3	1	5	3	5	5	5	5
4	4	5	5	2	4	5	2	4	2	4	4	3	4
4	2	5	5	1	4	2	1	5	1	5	1	5	5
2	5	5	5	3	3	2	2	4	2	5	5	4	5
5	3	5	5	5	5	3	1	5	1	5	1	5	5
4	4	4	4	4	4	4	4	4	4	4	4	4	4
3	3	3	3	3	3	3	3	3	3	3	3	3	3
5	5	5	5	5	5	1	1	3	1	5	3	5	5
3	4	4	4	4	3	4	2	3	3	4	2	3	3
3	3	3	3	3	3	4	2	4	2	4	2	4	4
4	2	4	4	3	4	2	2	4	2	4	1	4	4
4	3	4	4	4	4	4	4	4	4	4	4	4	4
3	4	4	4	4	4	4	3	4	3	4	4	4	3
5	2	5	5	2	5	1	1	5	1	5	1	5	1
2	3	4	4	3	4	3	2	3	3	3	2	3	3
2	5	5	5	5	5	5	1	5	1	5	5	5	5
3	4	5	5	2	5	4	1	5	2	5	3	5	5
4	3	2	5	2	4	4	2	4	1	4	2	4	4
3	3	3	3	3	3	3	2	3	3	3	3	3	3
5	5	5	5	5	5	5	5	5	5	5	5	5	5
3	3	4	4	4	4	3	2	4	2	4	3	4	4
5	3	5	5	3	5	3	2	4	2	4	1	5	5
2	2	2	2	2	4	2	2	2	2	2	2	2	2
4	4	4	4	4	4	3	4	4	2	4	3	4	4
3	4	4	4	3	3	4	3	4	3	4	2	4	4
4	2	5	5	4	4	3	2	4	2	4	1	3	4
4	2	4	4	3	4	4	2	4	1	4	1	4	4
5	4	5	5	4	5	2	1	5	1	5	1	5	5
3	3	4	4	3	4	4	2	3	1	4	3	3	3
5	4	5	5	2	5	3	1	4	1	5	2	5	5
3	3	5	5	5	4	2	2	3	2	3	3	3	4
5	3	5	5	1	5	4	1	5	1	5	1	5	5
3	4	5	5	5	5	4	2	4	3	5	5	4	5
3	3	5	5	3	5	4	1	4	2	5	2	5	5
4	2	4	5	2	4	3	2	4	2	4	2	4	4
5	5	5	5	5	5	4	1	5	3	5	5	5	5
4	4	4	4	4	4	4	4	4	4	4	4	4	4
3	4	5	5	4	5	4	2	4	2	5	5	4	4
2	5	5	5	5	5	3	2	2	2	5	5	3	4
4	2	5	4	3	4	2	2	4	2	4	3	4	4
3	4	4	4	4	3	4	3	3	4	3	3	2	2
3	3	3	3	3	3	3	3	3	3	3	3	3	3
3	4	3	4	3	4	4	3	4	4	3	4	3	4
3	3	3	3	3	3	3	3	3	3	3	3	3	3
3	2	4	4	3	4	3	3	3	3	3	3	3	3
2	2	3	3	2	3	2	2	3	2	3	2	3	3
3	3	3	3	3	3	4	3	3	4	3	4	3	4
5	1	5	5	1	5	1	1	5	1	5	1	5	5
3	4	2	4	5	3	5	4	4	2	4	3	4	3

3	1	5	5	5	5	4	1	4	2	4	2	4	4
3	4	4	4	4	4	4	3	4	3	4	2	4	4
2	2	2	5	3	4	3	2	1	2	5	3	2	3
3	4	5	5	4	5	5	1	5	3	5	3	5	5
4	3	4	4	2	4	2	2	4	2	4	2	4	4
3	5	5	5	3	5	3	2	3	2	5	3	4	4
3	3	5	5	4	5	4	2	5	2	5	2	4	4
2	4	5	5	3	5	5	3	3	3	5	2	4	5
3	4	5	5	3	5	5	2	3	1	5	3	5	4
3	4	3	5	5	5	2	2	4	4	4	2	4	4
3	2	4	5	3	4	1	1	4	1	4	1	5	4
3	4	4	4	4	5	3	2	3	2	4	2	4	4
3	3	4	4	2	4	3	2	4	2	4	3	3	4
5	5	5	5	1	5	1	1	5	1	5	1	5	5
4	5	4	4	3	4	3	3	3	3	3	3	3	3
3	2	4	4	3	4	3	2	4	2	4	2	4	3
2	4	3	4	3	3	4	2	4	2	4	4	3	3
3	4	4	5	3	4	4	2	4	2	4	2	4	4
2	5	5	5	3	5	5	2	3	2	5	2	4	4
3	4	4	4	3	4	4	2	4	2	4	4	4	4
5	5	5	5	5	5	5	5	5	5	5	5	3	5
5	1	5	5	3	5	2	1	5	1	5	1	5	5
3	2	3	5	3	4	3	3	3	3	3	3	3	5
3	1	5	5	3	5	4	2	4	2	5	2	5	5
3	3	3	3	3	3	3	3	3	3	3	3	3	3
5	3	5	5	3	5	1	1	5	1	5	1	5	5
3	2	4	5	5	5	2	2	2	1	5	2	3	4
2	3	3	4	2	3	3	2	4	2	3	2	2	3
5	1	5	5	1	5	1	1	5	1	5	1	5	5
2	3	4	5	4	4	4	2	4	2	4	2	4	4
5	1	5	5	1	5	1	1	5	1	5	1	5	5
1	5	4	5	4	2	3	4	2	3	3	4	2	2
3	3	3	3	3	3	2	2	4	2	4	2	3	3
2	4	4	4	4	3	4	3	4	3	4	4	3	3
2	3	4	4	3	4	4	3	3	4	4	4	3	2
3	4	5	3	3	2	5	3	3	2	4	4	2	3
2	4	4	4	4	3	4	4	3	4	4	4	3	3
5	1	5	5	3	5	1	1	5	1	5	1	5	5
4	2	4	4	4	4	4	2	4	2	4	2	4	4
3	3	4	4	4	4	4	4	4	4	4	4	3	4
3	3	5	5	4	4	3	3	4	3	3	3	3	3
4	3	4	4	3	2	4	3	4	2	4	3	4	4
3	5	5	5	5	5	2	3	4	2	4	4	4	4
4	1	4	4	2	4	3	1	4	1	4	2	4	4
2	4	2	4	4	3	1	4	2	4	3	4	2	2
2	3	4	4	2	3	3	2	4	3	4	2	4	3
5	1	5	5	3	5	3	1	5	1	5	1	5	5
2	4	4	4	3	4	3	3	3	3	4	3	4	4
4	4	5	5	5	5	1	1	5	1	5	1	5	5
5	1	5	5	1	5	5	1	5	1	5	1	5	5
2	5	5	5	3	5	5	2	4	4	4	3	4	4
3	2	5	4	4	4	3	2	4	2	4	2	4	4
3	3	3	3	3	3	3	3	3	3	3	3	3	3
3	1	4	4	3	4	4	1	3	1	4	1	4	4
3	5	5	3	5	4	5	3	3	3	3	4	3	4
3	2	3	3	4	3	3	3	3	3	3	3	3	3

3	3	3	4	4	4	4	3	4	4	4	3	4	3
5	1	5	5	2	5	1	1	5	1	5	1	5	5
3	3	5	5	4	3	5	1	4	5	5	1	5	5
2	1	4	5	3	4	3	1	3	1	5	1	4	5
3	3	3	3	3	3	3	2	3	3	3	3	3	4
3	3	3	3	3	3	3	3	3	3	3	3	3	3
3	4	4	5	3	5	5	3	3	2	4	2	4	4
3	3	4	4	3	3	4	2	3	2	4	2	3	3
3	3	3	3	3	3	3	3	3	3	3	3	3	3
4	2	5	5	5	5	2	2	4	2	5	2	4	4
4	2	4	4	2	4	1	1	5	1	5	1	5	5
3	2	3	3	3	3	3	3	3	3	3	3	3	3
2	3	5	4	4	4	4	2	5	3	5	1	3	3
2	3	4	4	4	4	4	3	2	3	4	3	3	3
3	3	5	5	4	5	1	1	5	1	5	1	5	5
2	4	3	3	4	3	4	3	3	3	2	4	3	2
2	4	4	3	2	3	3	3	3	3	4	4	3	3
5	5	5	5	5	5	3	2	5	2	5	2	5	5
4	2	4	4	2	4	2	3	4	2	4	2	4	4
3	3	3	3	3	3	3	1	3	1	3	3	3	3
3	3	4	4	4	4	4	2	4	2	4	2	4	4
3	3	3	3	3	3	3	3	4	3	3	3	3	3
5	3	5	5	1	5	1	1	5	1	5	1	5	5
5	1	5	5	1	5	5	1	5	1	5	1	5	5
4	4	4	4	4	4	2	4	4	4	4	4	4	4
2	3	4	4	4	4	5	1	3	2	3	1	3	3
2	2	3	5	2	4	3	2	3	2	3	2	3	3
3	3	5	5	4	4	3	2	3	2	4	2	4	4
4	2	4	5	4	4	2	1	5	1	5	1	5	5
4	3	3	4	3	3	3	3	4	2	3	2	3	3
3	2	4	5	5	5	5	1	3	1	5	2	3	4
3	5	5	5	5	5	5	1	4	1	5	4	3	3
3	3	3	3	3	3	4	1	5	2	4	1	4	4
3	3	3	3	3	3	2	2	4	3	4	2	3	3
5	5	5	5	1	5	1	1	5	1	5	1	5	5
3	4	4	4	3	4	3	3	4	3	4	3	4	4
2	4	4	3	3	3	4	3	2	2	3	4	3	3
3	3	3	4	3	3	4	2	3	2	4	3	3	3
5	1	5	5	1	5	1	1	5	1	5	1	5	5
3	4	4	4	3	4	2	2	3	2	4	3	3	3
3	2	4	4	4	4	3	3	4	3	4	2	4	3
3	3	3	3	3	3	3	3	3	3	4	3	3	3
4	4	5	5	4	5	5	1	5	1	5	1	5	5
4	5	5	5	3	5	5	2	2	1	5	4	4	4
3	3	5	5	5	5	1	1	3	1	5	3	5	5
3	5	5	5	4	4	5	2	3	4	4	4	3	4
3	3	3	4	4	4	2	2	3	2	4	4	4	4
3	4	4	4	3	4	4	3	3	3	4	3	3	4
4	4	3	3	4	3	4	4	4	4	4	4	4	5
1	5	4	4	4	4	2	1	1	3	4	4	3	3
2	2	4	4	2	4	3	3	3	2	4	1	4	3
5	1	5	5	5	5	5	5	5	5	5	5	5	5
2	4	4	4	3	3	4	4	2	3	4	3	3	3
3	3	3	3	3	3	3	3	3	3	3	3	3	3
4	3	4	4	3	4	3	3	4	2	4	2	4	4
2	5	5	5	5	5	5	2	5	1	5	5	5	5

3	3	5	5	5	5	2	1	4	1	5	1	4	5
5	5	5	5	5	5	5	1	5	1	5	5	5	5
3	3	5	5	3	4	2	1	4	1	5	1	5	5
3	3	3	3	3	3	3	3	3	3	3	3	3	3
4	3	4	4	4	4	4	3	3	3	3	3	3	3
3	4	4	4	3	4	4	2	4	2	4	2	4	4
3	3	3	4	4	4	2	2	4	4	4	3	4	3
3	3	4	4	4	4	4	3	4	2	4	3	4	4
3	2	3	3	2	3	2	2	3	2	3	2	4	4
4	4	4	4	3	4	2	2	4	3	4	3	3	3
5	4	5	5	4	5	4	2	5	2	5	3	5	5
3	3	4	4	2	3	3	2	4	2	4	2	3	4
5	1	5	5	1	5	1	1	5	1	5	1	5	5
5	1	5	5	5	5	1	1	5	1	5	1	5	5
4	4	4	4	4	4	2	3	4	2	4	2	4	4
2	2	4	4	3	4	2	2	4	2	4	2	3	4
3	3	3	3	3	3	3	3	3	3	3	3	3	3
3	2	5	5	4	5	1	1	3	1	5	2	4	4
5	1	5	5	5	5	4	1	4	2	5	2	5	5
2	1	5	5	2	5	5	1	4	2	5	1	5	5
5	1	5	5	1	5	1	1	5	1	5	1	5	5
1	5	4	5	3	3	4	3	3	2	4	5	3	3
3	3	4	4	3	3	3	3	3	2	4	3	3	3
3	3	3	3	3	3	3	3	3	3	3	3	3	3
3	2	4	4	4	4	3	2	4	2	4	2	4	4
3	2	3	3	3	3	3	3	3	3	3	3	3	3
3	3	3	5	3	5	5	3	5	3	5	3	5	5
4	1	4	4	1	4	1	1	4	1	5	1	5	5
3	1	5	5	3	5	1	1	5	1	5	1	5	5
1	5	5	5	5	5	5	5	2	5	4	5	2	2
5	1	5	5	3	5	3	1	5	1	5	1	5	5
5	1	5	5	1	5	1	1	5	1	5	1	5	5
4	3	4	4	2	4	2	2	2	2	5	2	5	5
3	3	5	5	2	5	3	2	4	2	5	3	5	5
4	4	5	5	4	5	4	1	4	1	4	1	4	5
3	1	5	5	4	4	2	2	4	2	4	1	4	4
3	3	3	3	3	3	3	3	3	3	3	3	3	3
3	5	5	5	5	5	5	2	2	5	5	5	4	3
2	3	4	3	3	2	2	3	3	3	3	3	3	3
3	4	4	4	4	5	4	4	3	4	4	4	3	4
1	3	5	5	3	3	3	3	4	2	4	2	3	4
3	3	3	3	3	3	3	3	3	3	3	3	3	3
3	4	5	5	3	5	1	1	4	1	5	3	5	5
3	4	4	4	4	4	4	2	3	2	4	3	4	4
5	1	5	5	1	5	1	1	5	1	5	1	5	5
1	4	5	4	3	3	3	3	3	3	3	2	3	3
3	3	5	5	2	4	4	1	4	2	5	2	4	4
5	5	5	5	5	5	5	5	5	5	5	5	5	5
2	3	3	3	3	3	3	3	3	3	3	3	3	3
3	3	3	3	3	3	3	3	3	3	3	3	3	3
2	5	5	5	3	5	5	3	4	4	4	5	3	3
4	1	4	5	1	5	1	1	4	1	5	1	5	5
3	4	4	4	4	4	3	2	3	2	4	2	4	4
3	3	4	4	4	4	2	2	4	2	4	2	4	4
2	3	3	4	3	3	3	2	3	3	3	2	3	3
3	3	3	4	3	4	3	2	3	2	4	2	4	4

2	4	4	4	2	4	2	2	4	2	4	1	4	4
4	4	4	4	4	3	2	2	3	2	4	4	4	4
2	4	2	4	4	2	4	2	3	2	4	2	2	3
5	5	5	5	5	5	5	5	5	5	5	5	5	5
3	3	2	4	2	3	3	2	4	2	3	2	4	3
4	3	5	5	5	5	3	1	5	1	5	1	4	5
3	3	3	4	3	3	5	3	3	3	3	4	3	3
3	4	4	4	2	3	5	2	4	2	4	2	3	3
5	1	5	5	1	5	1	1	5	1	5	1	5	5
1	3	5	5	5	5	1	1	5	3	5	1	5	5
3	3	5	5	4	4	4	2	4	2	5	3	5	5
4	4	5	5	4	5	3	1	3	2	5	3	4	4
1	5	4	4	4	4	4	2	4	3	3	4	4	4
5	5	5	5	5	5	5	1	5	1	5	1	5	5
5	2	5	5	2	5	3	2	5	1	5	1	5	5
2	5	5	5	5	5	5	1	5	5	5	5	5	5
3	3	4	3	3	4	5	3	1	3	3	3	3	3
3	3	3	3	4	3	3	2	3	2	3	2	3	3
4	2	3	5	4	4	3	2	4	2	4	1	4	4
4	1	4	4	3	4	3	1	4	1	4	2	4	4
3	3	3	3	3	3	4	3	3	3	4	4	3	3
3	2	4	5	2	4	1	1	4	1	5	1	4	4
3	3	5	5	2	5	2	1	5	2	5	1	5	5
2	4	3	3	2	3	3	3	3	3	3	3	3	3
3	3	5	4	3	4	4	2	4	2	4	2	3	3
5	1	5	5	5	5	1	1	5	1	5	1	5	5
4	4	5	4	5	4	4	5	3	4	3	5	4	4
4	2	5	5	2	5	2	1	4	1	5	2	4	4
3	1	4	4	4	5	2	1	4	1	5	1	5	5
3	4	4	4	4	4	4	2	3	3	4	4	4	4
3	4	4	4	4	4	4	2	3	3	4	2	3	3
3	2	4	5	2	4	2	2	4	1	5	2	5	5
5	5	5	5	1	5	5	1	5	1	5	1	5	5
4	5	5	5	4	5	5	3	2	2	5	3	4	5
3	3	3	4	4	4	4	3	3	3	4	3	4	4
3	3	3	3	3	3	3	3	3	3	3	3	3	3
5	1	5	5	1	5	1	1	5	1	5	1	5	3
3	2	3	4	2	3	3	3	3	2	4	2	3	3
4	2	5	5	3	5	3	2	5	3	5	2	5	5
3	1	5	5	4	4	3	1	4	1	4	1	4	4
2	4	4	4	4	4	2	2	2	3	4	3	4	4
4	4	4	4	4	4	4	4	4	4	4	3	4	4
3	2	4	4	3	4	3	2	4	2	4	1	4	4
3	3	3	3	3	3	5	3	3	2	4	4	3	4
5	1	5	5	3	5	3	1	5	1	5	1	5	5
4	3	4	4	3	4	4	2	4	2	4	3	4	4
2	3	3	3	3	4	4	3	4	3	3	3	2	3
3	3	4	4	4	3	2	2	4	2	4	3	3	3
3	4	4	4	4	4	3	2	2	2	4	4	4	4

a49	a50	a51	a52	a53	a54	a55	a56	a57	a58	a59	a60	a61	a62
1	5	5	1	5	1	5	5	5	5	5	1	5	5
1	4	3	1	2	3	2	4	3	3	3	5	3	3
1	5	2	1	4	1	3	1	3	5	3	1	4	2
1	3	1	3	3	2	2	3	3	4	3	2	3	2
1	4	2	2	4	1	4	2	3	4	4	3	4	3
5	5	1	1	3	4	2	4	4	2	2	4	4	2
3	4	2	4	3	3	3	3	3	4	2	4	3	3
4	4	4	4	4	2	2	2	4	4	4	2	2	4
3	3	3	3	3	3	3	3	3	3	3	3	3	3
4	4	4	4	4	3	4	4	4	4	4	4	4	3
2	5	1	1	3	2	3	3	5	5	4	2	3	2
3	3	3	3	3	3	3	3	3	3	3	3	3	3
1	4	3	3	3	3	2	3	3	3	3	3	3	3
1	4	3	3	3	3	2	3	4	3	3	4	3	3
2	4	2	2	4	2	4	2	4	4	2	2	4	2
1	3	4	2	4	2	3	1	3	3	3	2	4	2
2	3	3	3	3	3	2	4	3	3	3	3	3	3
1	4	4	3	4	3	3	4	4	4	4	2	3	4
1	3	2	1	3	2	4	2	3	5	5	2	3	3
2	3	2	5	2	5	2	5	3	2	4	3	3	4
4	4	4	3	3	4	4	4	4	5	4	2	3	4
1	5	5	5	5	5	5	5	5	5	5	5	5	1
4	4	4	4	4	4	4	4	2	3	3	3	4	3
1	4	1	1	4	1	4	1	5	5	4	1	4	1
1	3	2	3	3	3	3	2	3	4	3	3	3	3
2	4	3	2	4	2	4	4	4	4	4	2	4	2
1	5	2	2	3	1	3	2	3	3	4	4	4	3
1	3	4	2	4	2	2	4	4	3	2	1	3	2
1	3	2	2	4	2	4	1	4	4	3	2	3	2
2	5	3	2	4	2	3	1	4	4	2	1	3	3
1	4	3	1	4	2	4	3	4	4	4	3	4	2
2	5	3	1	4	2	4	3	5	5	3	2	3	2
2	4	4	4	3	4	2	2	2	2	2	2	4	2
2	2	5	4	4	3	4	3	3	3	3	3	3	5
2	5	3	1	5	1	5	4	5	5	2	2	4	2
2	4	3	3	3	3	3	3	3	4	3	4	3	3
2	4	2	2	4	2	4	2	3	4	3	3	4	2
3	3	3	3	3	3	3	3	3	3	3	3	3	3
2	4	3	1	4	2	3	4	4	4	4	2	3	3
3	3	3	3	3	3	3	3	3	3	3	3	3	3
2	3	3	2	3	2	3	3	3	3	3	2	2	4
3	3	3	3	3	3	3	3	3	3	3	3	3	3
1	3	4	2	3	4	1	4	3	3	4	3	2	3
1	5	5	3	3	3	3	3	3	5	5	1	5	3
2	4	4	2	4	3	2	4	4	4	4	3	3	3
1	3	1	1	3	1	3	1	3	4	3	2	4	2
2	4	4	2	4	2	2	4	4	4	4	2	2	4
4	4	4	4	4	4	4	4	4	4	4	4	4	4
3	4	1	1	4	2	3	1	4	4	4	2	4	2
1	5	2	1	4	1	3	2	4	4	2	1	5	3

1	5	3	1	5	1	5	4	5	5	3	1	4	1
2	4	3	3	4	4	2	4	3	3	3	4	2	4
2	4	4	2	3	2	3	4	4	4	4	2	4	2
2	4	2	2	4	2	4	2	4	4	4	2	4	3
2	4	1	1	4	1	3	3	4	4	3	2	3	2
3	3	3	3	4	2	2	4	4	4	4	2	3	4
2	4	4	2	3	2	2	4	4	4	4	4	2	3
2	4	4	3	3	3	3	4	4	3	4	4	3	3
1	3	2	2	3	3	3	2	3	3	2	2	2	2
1	4	2	3	4	2	3	3	4	4	4	2	4	2
1	3	2	3	3	3	3	2	3	4	3	2	4	3
5	5	1	1	4	1	4	4	5	2	1	1	4	1
2	3	4	2	3	1	3	3	3	4	3	2	4	1
1	5	1	3	3	3	3	1	3	5	5	5	5	2
3	3	3	3	2	3	3	4	3	3	3	3	3	2
1	4	2	2	4	2	4	2	4	4	2	2	4	2
1	4	4	3	3	3	2	4	3	4	4	2	2	4
2	4	2	1	4	2	4	2	4	4	2	2	4	2
1	3	3	3	3	4	2	3	3	3	4	4	3	2
1	4	2	3	3	2	3	3	3	5	2	2	3	2
3	4	2	2	3	3	3	2	4	4	3	2	4	2
2	5	3	2	2	2	4	4	4	4	4	3	3	2
1	5	2	1	5	1	5	2	5	5	2	1	5	1
1	4	3	2	1	2	3	3	4	4	2	2	5	4
2	3	3	2	3	2	3	3	3	3	2	2	4	2
1	3	4	4	3	3	1	4	3	3	4	4	2	3
1	5	1	1	4	1	3	2	4	4	2	1	3	1
1	3	1	3	2	2	2	1	2	5	2	2	3	3
1	5	1	1	5	1	5	1	5	5	2	1	4	2
1	3	2	2	4	2	2	2	3	4	3	3	2	2
2	4	3	3	3	3	3	3	3	3	4	3	3	2
2	4	1	1	4	1	3	1	4	4	2	1	4	2
2	4	4	2	3	3	3	4	3	4	4	4	2	3
1	5	5	5	3	4	4	5	4	5	4	3	2	3
1	5	1	1	5	1	3	2	5	5	1	1	4	1
3	3	3	3	3	3	3	3	3	3	3	3	3	3
2	5	3	3	3	2	3	3	4	5	5	4	3	3
2	4	2	2	4	2	3	3	4	3	3	2	3	2
2	3	3	3	3	3	3	3	3	3	3	3	4	3
1	5	1	1	5	1	4	1	5	5	1	1	5	1
1	3	2	3	3	4	3	3	3	3	4	3	3	4
3	3	1	1	3	1	3	1	3	1	3	1	3	3
2	4	4	2	4	2	3	4	4	4	4	2	4	2
1	5	2	2	4	2	3	4	5	5	5	2	5	5
1	5	1	1	5	1	5	1	5	5	1	1	5	1
1	5	1	2	5	2	5	3	5	5	3	2	5	2
3	3	2	1	3	2	3	3	3	3	3	3	3	3
1	3	3	3	3	3	2	4	3	3	4	3	3	3
1	3	3	2	2	3	1	4	1	3	3	5	1	4
3	3	2	2	3	2	3	3	3	3	3	3	3	2
1	4	3	1	4	1	3	3	3	4	3	1	3	1
2	4	2	2	4	4	5	2	4	4	2	2	3	2
3	4	3	3	3	3	4	4	4	4	4	3	4	4
1	5	4	2	4	2	3	3	5	5	4	2	4	2
2	4	2	2	4	2	3	4	4	4	4	2	3	3
1	5	3	1	5	2	2	3	5	5	3	1	5	1

1	5	1	1	5	3	2	1	5	5	2	1	4	2
1	3	3	3	3	3	3	3	3	2	3	3	3	3
2	5	2	3	3	3	3	2	4	3	2	5	3	3
2	5	3	1	5	1	3	3	5	5	2	1	3	1
1	3	3	2	3	3	2	4	2	2	4	3	4	3
1	3	3	3	3	3	3	3	3	3	3	3	3	3
1	4	1	1	4	1	3	1	4	4	1	1	5	1
1	4	3	2	3	2	3	2	4	4	5	4	4	2
3	4	3	3	4	2	4	2	4	2	4	3	4	2
2	3	2	2	4	2	3	2	3	4	2	2	4	3
1	5	1	2	4	1	3	3	4	5	4	4	3	3
2	4	4	2	4	2	3	4	3	3	4	2	2	4
2	3	3	3	3	3	3	3	3	3	3	3	3	2
1	4	3	3	3	4	2	4	3	3	4	4	3	4
3	4	5	5	3	3	1	4	3	2	5	3	2	4
3	3	4	2	2	3	2	2	2	1	2	2	3	3
5	5	5	5	5	5	5	5	5	5	5	5	5	5
1	3	3	3	4	3	2	3	3	4	3	3	2	3
1	5	1	1	5	1	5	1	5	5	5	1	5	1
1	5	5	3	3	3	3	5	3	5	5	3	4	5
1	4	3	1	3	3	3	4	4	4	3	2	4	3
3	4	2	3	4	3	4	3	2	3	3	4	3	3
1	4	4	1	3	2	3	3	4	4	4	4	3	4
2	3	2	2	3	2	3	4	3	4	3	3	4	4
2	4	3	3	3	3	3	2	3	3	3	2	3	3
2	4	3	2	4	3	3	3	4	4	3	2	4	2
1	5	1	1	5	1	5	1	5	5	1	1	4	1
3	4	4	2	4	2	3	4	4	4	4	3	3	2
1	5	3	5	5	5	1	3	5	5	5	5	1	5
1	2	1	2	4	1	4	1	3	4	4	4	4	5
3	3	3	3	3	3	3	3	3	3	3	3	2	3
1	2	3	3	2	3	2	4	3	3	3	4	2	4
3	3	3	3	3	3	3	3	3	4	3	3	4	2
1	2	4	1	3	4	3	5	3	1	4	4	1	5
2	4	4	2	4	2	3	3	4	3	4	2	3	2
1	4	2	2	4	2	3	2	4	4	4	2	3	1
1	3	2	1	3	1	3	1	3	3	3	3	2	2
3	3	4	3	3	3	2	4	3	3	3	3	3	3
3	3	3	3	3	3	3	3	3	3	3	3	3	3
1	1	5	3	1	3	1	1	1	1	1	3	1	5
3	3	3	3	3	3	3	3	3	3	3	3	3	4
2	3	3	2	3	3	2	4	3	2	4	4	2	3
3	3	3	3	3	3	3	3	3	3	3	3	3	3
2	4	2	2	4	2	3	3	4	4	3	3	3	2
2	3	2	2	3	2	3	2	3	4	3	3	3	2
1	3	3	3	3	3	3	3	4	4	4	4	4	2
3	3	5	2	2	2	5	5	5	5	5	3	3	2
2	3	2	3	3	3	3	2	3	3	3	2	3	2
1	5	3	1	5	3	5	3	5	5	3	3	3	2
5	5	5	5	5	5	5	5	5	5	5	5	5	5
1	5	1	1	5	1	5	2	5	5	4	1	5	1
1	5	4	2	5	2	3	4	5	4	4	3	3	2
3	3	3	3	3	3	3	3	3	3	3	3	3	3
1	5	1	1	4	1	4	1	5	5	4	1	4	1
2	3	3	3	2	3	3	3	3	3	3	3	1	3
2	3	3	2	4	3	4	3	4	3	2	3	3	3

2	4	2	2	4	2	4	2	4	4	2	3	4	2
2	4	1	2	4	1	5	2	4	4	1	2	4	2
4	4	4	4	4	2	4	4	3	4	4	4	4	4
2	3	3	3	3	3	4	3	3	4	3	3	3	2
2	5	5	5	5	5	5	5	5	5	5	2	4	4
2	5	5	1	2	1	2	4	3	4	4	3	3	4
1	5	1	2	4	2	3	4	4	5	4	4	3	4
3	3	3	3	3	3	3	3	3	3	3	3	3	3
1	2	4	4	3	3	3	4	3	3	4	4	3	4
1	5	5	1	5	5	1	5	5	5	5	1	5	5
3	5	3	1	4	2	2	4	5	5	4	2	3	2
2	3	4	3	3	2	3	3	3	4	4	4	3	2
1	5	4	5	1	5	2	2	3	5	5	5	3	3
1	4	4	2	3	2	3	4	4	5	5	4	3	3
1	4	2	1	4	3	2	1	4	5	3	3	2	1
3	3	3	3	3	3	3	3	3	3	3	3	3	3
1	5	1	1	5	1	5	5	5	5	1	1	1	1
3	3	3	3	3	3	3	3	3	3	3	3	3	3
2	5	2	2	5	2	4	1	4	4	4	2	4	2
2	5	5	5	4	5	1	5	4	4	5	4	2	3
4	3	4	2	3	3	3	2	4	3	3	3	3	3
1	4	4	3	4	2	3	4	4	4	4	2	3	4
1	3	4	2	3	1	3	3	3	3	4	2	3	2
3	3	3	3	3	3	3	3	3	3	3	3	3	3
3	3	3	3	3	3	3	3	3	3	3	3	3	3
3	4	4	3	4	4	3	4	4	4	4	4	3	4
1	5	5	1	5	1	4	5	5	5	5	1	4	1
1	4	3	3	4	2	4	4	5	4	5	2	4	2
3	3	3	3	3	3	3	3	3	3	3	3	3	3
1	4	4	2	4	3	2	3	3	5	3	4	4	4
1	4	2	2	4	2	2	3	4	4	4	3	3	1
2	5	5	2	5	2	5	5	5	5	5	2	5	2
1	5	1	1	5	1	3	1	5	5	2	1	5	1
1	5	1	1	5	1	4	2	5	5	4	1	4	1
2	4	1	2	3	4	2	1	4	4	4	3	4	2
2	5	2	2	3	3	3	4	5	4	4	2	3	2
2	3	2	2	3	2	4	2	3	3	3	2	4	2
1	4	2	2	4	3	3	1	3	3	4	3	3	3
2	4	3	2	3	2	4	4	3	4	3	3	3	3
2	4	2	2	4	2	3	2	4	4	3	2	4	2
3	3	3	3	3	3	3	3	3	3	3	3	3	3
1	5	3	1	5	2	5	2	5	5	2	2	5	1
2	4	5	3	3	4	3	4	3	2	4	4	3	5
1	3	4	3	3	2	3	2	4	4	5	5	2	3
2	3	3	2	3	2	3	4	3	3	4	3	3	4
1	4	4	1	4	4	4	4	4	4	4	1	2	4
2	4	2	2	4	2	2	2	4	4	2	2	3	3
1	4	3	2	3	3	3	4	3	3	4	4	2	2
2	3	1	1	3	2	4	3	3	4	4	2	3	2
2	4	2	2	4	2	3	4	3	4	4	2	2	2
2	3	4	3	4	3	3	4	3	3	4	3	2	4
3	5	3	1	5	1	5	3	5	5	4	1	3	1
2	4	3	2	4	2	3	3	3	4	2	2	4	2
3	4	3	3	3	3	3	3	3	3	3	3	3	3
2	4	2	2	3	3	2	2	3	4	2	2	4	2
3	3	3	1	4	2	3	4	3	3	3	2	3	3

2	4	4	2	3	2	3	4	4	4	4	3	4	3
2	3	4	3	4	3	3	3	3	3	3	3	3	3
2	5	2	1	4	1	4	2	4	4	1	1	3	1
1	3	4	4	2	4	2	4	3	3	4	3	2	4
3	5	3	5	3	3	3	3	5	5	5	4	3	3
1	5	1	1	5	1	5	1	5	5	1	1	5	1
1	3	2	2	3	3	3	3	3	3	4	3	3	3
1	5	4	2	4	2	4	4	4	4	4	4	4	2
2	5	2	1	4	2	4	3	4	4	4	3	4	3
3	3	3	3	3	3	3	3	3	3	3	3	4	2
2	5	2	2	4	3	3	3	4	4	4	4	3	2
1	5	1	1	4	2	4	1	4	5	4	1	5	1
2	4	3	2	4	1	3	2	4	4	3	1	4	1
1	4	4	1	3	3	3	4	4	4	4	3	2	2
1	4	1	1	3	2	3	1	4	4	4	3	3	2
1	4	1	1	4	3	3	1	5	5	3	1	3	1
1	5	1	1	5	1	5	5	5	5	1	1	5	1
3	3	3	3	3	3	3	3	3	3	3	3	3	3
1	3	3	3	3	3	3	3	3	3	3	3	3	2
1	2	3	1	4	3	1	4	4	5	1	1	3	1
2	4	2	1	4	1	4	2	4	5	2	2	5	1
1	3	5	3	3	4	2	5	4	4	4	3	1	2
2	5	2	2	4	2	4	1	4	5	4	1	4	2
2	4	4	2	4	2	3	2	4	4	4	2	2	2
1	4	2	4	4	4	1	4	5	4	5	1	4	4
4	4	4	2	4	4	4	4	4	4	4	2	3	2
1	5	1	2	5	1	4	2	5	5	5	1	4	1
2	4	4	4	3	3	3	4	2	3	4	4	2	2
2	4	2	2	4	2	4	2	4	4	4	2	4	2
2	4	3	1	3	2	3	3	4	4	3	3	3	4
3	4	4	3	3	3	3	4	4	3	4	4	3	4
1	4	4	1	4	4	3	4	3	4	4	3	2	4
1	4	1	1	4	1	3	1	4	5	1	1	4	1
2	3	3	2	4	2	3	3	3	3	4	2	2	3
1	5	1	1	5	1	3	2	5	4	4	3	3	4
5	5	5	2	5	5	5	5	5	5	5	5	5	1
1	3	4	2	3	2	4	4	4	3	5	4	2	4
1	5	1	1	5	1	4	3	5	5	3	1	3	1
1	4	4	1	4	1	3	2	4	5	2	1	3	2
1	5	1	1	4	1	4	1	5	5	2	1	4	1
1	2	4	4	4	3	2	4	3	3	4	2	3	4
1	4	1	1	4	3	3	1	4	4	1	1	2	3
1	5	1	1	5	1	1	5	5	5	5	1	5	1
1	3	2	3	3	3	3	2	3	4	4	3	3	3
3	5	4	3	4	2	2	4	4	4	5	3	4	3
1	2	5	1	5	5	4	5	5	5	5	2	2	4
1	4	3	3	3	3	1	4	3	3	5	3	3	4
1	2	3	2	2	3	2	3	2	3	4	4	3	4
2	3	3	2	2	3	2	3	3	3	2	3	2	3
5	5	5	1	5	1	5	5	5	5	5	1	5	3
1	4	4	2	4	3	2	4	3	4	5	5	2	3
2	4	2	2	4	2	3	2	4	4	2	2	3	2
1	4	1	1	4	1	3	4	4	5	4	1	3	2
2	4	4	1	3	3	3	4	4	4	4	4	4	2
3	4	3	2	4	2	3	3	4	4	3	3	3	2
1	4	4	2	4	2	3	4	5	4	4	2	3	2

1	5	1	1	5	4	5	3	5	5	4	2	5	3
2	4	3	3	3	2	3	3	4	4	3	2	3	3
1	5	2	1	4	4	3	3	5	5	4	3	4	2
3	4	4	3	3	2	3	3	4	4	4	3	3	2
1	5	1	1	5	1	3	1	5	5	1	1	5	1
4	4	2	2	4	2	4	2	4	4	2	2	4	2
3	3	3	3	3	3	3	3	3	3	3	3	3	3
1	2	2	5	2	4	3	2	2	3	4	4	3	4
2	3	4	3	3	4	3	4	4	4	4	4	2	3
3	5	1	1	5	1	5	1	5	5	5	2	5	1
3	3	3	3	3	3	3	3	3	3	3	3	3	3
1	5	1	1	5	1	4	3	5	5	5	2	5	1
3	5	5	2	3	4	3	5	4	4	5	5	1	5
3	5	1	1	3	1	3	4	3	3	5	2	2	4
5	4	4	4	5	4	4	5	4	4	4	4	4	5
5	5	5	5	5	5	5	5	5	5	5	5	5	5
3	4	4	1	5	3	4	4	5	5	3	3	2	4
3	3	3	3	3	3	3	3	3	3	3	3	3	3
1	3	3	3	3	3	3	3	3	3	3	3	3	3
1	4	1	1	4	4	4	4	4	4	3	1	4	2
3	4	2	2	4	3	3	5	4	4	4	2	4	3
3	3	3	3	3	3	3	3	3	3	3	3	3	3
2	3	2	2	3	2	3	2	3	3	4	3	3	2
1	3	4	2	3	2	2	2	5	5	5	5	3	3
2	4	2	2	4	2	3	3	4	4	4	2	3	2
1	4	4	4	4	4	4	2	4	4	4	4	4	2
3	3	4	3	4	3	3	3	3	4	3	2	3	3
2	3	4	3	3	4	3	4	3	3	3	3	3	3
2	4	2	2	4	2	3	3	4	4	2	2	3	3
2	5	2	1	4	2	4	2	5	5	4	1	3	2
1	4	1	1	4	2	3	1	4	4	1	2	3	3
3	4	2	2	4	3	3	2	4	4	2	3	3	2
1	5	1	5	5	1	3	1	5	5	1	1	3	1
3	4	4	3	3	3	3	3	3	3	3	3	4	3
1	5	5	1	5	2	1	5	4	3	5	4	2	4
2	5	2	2	4	2	3	4	5	5	3	2	3	1
1	5	2	1	5	1	5	2	5	5	1	1	3	1
1	3	2	1	2	1	2	2	3	4	4	3	2	3
1	4	1	1	4	1	4	2	4	4	2	2	3	2
1	4	1	1	4	1	4	2	4	4	2	2	3	2
1	5	2	1	4	2	3	2	4	4	2	2	3	1
1	5	1	1	5	1	5	1	5	5	5	1	5	1
2	3	3	3	3	4	4	4	4	4	4	2	3	2
1	5	1	1	5	1	5	1	5	5	1	3	5	1
1	5	4	3	4	2	3	3	4	4	4	3	2	3
1	5	1	3	4	2	4	1	5	5	4	2	3	1
5	5	5	5	5	5	5	5	5	5	5	5	5	5
1	5	2	1	5	2	3	2	5	5	3	1	3	1
1	4	4	2	3	3	2	4	4	4	3	3	3	3
1	3	2	1	4	1	3	2	4	4	2	2	4	1
3	3	3	3	3	3	3	3	4	4	4	4	3	3
1	1	1	4	1	1	1	1	1	1	1	3	1	4
2	3	3	2	4	3	2	2	3	3	4	3	2	3
1	5	2	2	4	1	4	1	4	4	2	2	3	2
3	3	3	3	3	3	3	3	3	3	3	3	3	3
3	5	3	1	3	1	4	5	5	5	5	1	3	2

5	5	5	5	5	5	5	5	5	5	1	1	5	5
2	4	3	2	4	2	3	4	4	4	4	2	3	3
2	4	3	5	3	4	3	3	3	4	3	3	3	3
3	4	3	1	4	1	4	3	4	4	3	1	4	1
2	4	2	2	4	2	4	2	4	4	2	2	4	2
2	4	4	3	2	3	3	4	3	3	4	3	3	3
2	4	2	2	3	2	3	2	4	4	4	2	4	3
3	3	3	3	3	3	3	3	3	3	3	3	3	3
2	4	2	2	4	2	3	2	4	4	3	2	3	2
3	3	3	3	3	3	3	3	3	3	3	3	3	3
2	4	3	2	4	3	2	4	4	4	3	2	3	2
3	5	5	1	5	2	5	5	5	5	5	1	5	1
4	4	4	4	4	4	4	4	4	4	4	4	4	4
4	4	4	4	4	4	4	4	4	4	4	4	4	4
1	5	1	1	5	1	3	1	5	5	1	1	5	1
1	4	2	2	4	2	4	2	4	4	3	2	3	3
1	5	1	1	5	1	5	1	5	5	4	1	5	1
2	5	4	2	5	1	3	2	5	5	5	2	4	4
2	3	2	2	3	2	3	3	3	3	3	2	3	2
1	5	1	1	5	1	5	1	5	5	5	2	5	1
2	5	4	3	5	5	3	4	5	5	4	2	4	2
3	4	3	2	4	2	3	4	4	4	4	3	3	2
2	4	2	2	4	2	3	2	4	4	2	2	3	2
3	5	3	3	3	2	2	4	4	4	3	3	3	3
1	5	1	1	5	1	1	1	5	5	1	1	1	1
1	5	2	2	4	2	4	1	4	4	4	2	4	3
1	3	3	2	4	3	4	3	4	4	3	3	4	2
3	4	4	3	3	3	3	4	4	4	4	3	4	3
1	5	1	1	5	1	4	1	5	5	1	1	4	1
2	3	3	2	4	3	4	2	4	4	3	2	3	2
3	3	2	4	3	2	4	2	2	4	4	3	4	4
3	3	3	3	3	3	3	3	3	3	3	4	3	3
1	3	3	2	4	1	3	3	3	4	4	3	3	3
2	4	3	1	4	1	4	3	4	4	3	3	4	1
2	3	2	2	3	2	3	2	3	3	3	2	3	2
4	4	4	3	4	4	4	4	4	3	4	4	4	4
3	5	4	3	4	5	3	5	5	5	5	3	3	4
4	4	4	2	4	3	4	4	4	4	4	3	4	3
1	4	4	2	3	3	3	3	3	3	2	2	2	3
3	4	3	1	4	4	2	2	4	4	4	3	4	2
2	4	4	3	3	3	3	4	4	4	4	4	4	4
1	4	3	3	4	3	3	3	4	4	4	4	4	3
2	4	4	3	3	3	3	4	4	4	5	4	3	3
1	4	4	3	3	3	3	4	4	4	4	4	4	3
1	5	5	4	4	4	2	5	4	4	4	3	3	3
1	4	3	1	5	2	5	4	5	5	2	1	4	1
1	4	1	1	4	1	4	2	4	5	2	3	4	1
2	3	3	3	4	4	2	4	4	3	4	3	4	4
1	5	1	1	4	1	4	1	5	5	3	1	5	1
2	4	2	3	4	3	3	2	3	4	4	3	3	3
2	4	3	1	3	1	3	4	4	4	4	2	3	1
1	4	1	1	4	1	3	1	4	4	3	2	4	2
5	5	5	5	5	5	5	5	5	5	5	5	5	5
2	3	4	2	4	4	2	4	4	4	4	4	2	2
2	5	2	2	5	2	4	2	5	5	2	2	5	2
3	3	3	3	3	3	3	3	3	3	3	3	3	3

1	3	3	3	3	3	3	3	3	3	3	3	3	3
1	5	5	2	5	2	3	4	5	5	4	2	3	3
1	5	1	1	5	1	5	1	5	5	1	1	5	1
3	4	4	1	2	3	5	4	5	5	5	3	2	1
3	3	3	3	3	3	3	3	3	3	3	3	3	2
1	4	4	1	4	2	4	5	4	5	5	2	4	2
1	4	2	3	5	3	2	3	4	4	2	2	3	3
2	4	2	2	4	2	3	4	3	3	4	2	3	3
1	5	1	1	5	1	3	1	3	5	1	1	5	1
3	3	3	3	3	3	3	3	3	3	3	3	3	3
2	4	4	1	4	2	4	4	4	4	4	2	4	3
1	4	2	2	3	3	3	2	4	4	3	3	3	2
1	3	4	2	2	4	2	4	3	3	4	2	2	4
1	5	5	1	5	1	4	4	4	4	4	2	4	4
2	3	2	3	2	2	2	3	3	3	3	3	3	3
3	3	3	3	3	3	3	3	3	3	3	3	3	3
2	4	4	3	3	3	3	4	4	5	4	3	2	4
1	5	1	1	5	2	5	3	5	5	4	3	2	4
2	4	4	2	2	3	3	3	4	4	4	2	2	2
2	4	4	2	4	2	4	4	4	4	4	4	4	4
3	5	4	1	1	3	4	3	4	4	4	4	3	3
2	3	2	2	3	2	3	2	3	3	3	2	2	3
4	4	4	4	4	4	4	4	4	4	4	4	3	4
3	5	3	1	3	1	3	3	5	5	4	1	5	2
3	4	3	3	3	2	3	4	4	4	4	2	2	2
2	3	4	1	3	2	3	4	4	4	4	3	3	2
1	5	1	1	5	3	3	1	3	4	2	2	5	1
2	3	3	2	3	2	3	3	3	3	3	3	3	4
1	3	3	3	3	3	3	3	3	5	3	3	1	4
5	5	5	4	2	5	2	5	4	5	5	4	2	5
2	1	2	3	4	4	3	2	3	2	3	4	3	2
1	5	2	1	4	1	4	5	5	5	5	1	5	1
1	5	1	1	5	1	4	1	5	5	5	1	4	1
2	5	3	2	3	2	4	4	5	5	4	2	3	2
1	3	4	2	3	3	1	4	3	3	4	3	3	3
2	4	2	2	4	2	2	2	4	3	4	3	3	2
2	5	5	3	3	2	3	5	3	3	4	3	2	4
5	5	5	5	5	5	5	5	5	5	5	5	5	5
2	4	3	2	4	2	3	4	3	4	2	2	4	2
1	4	2	2	1	1	1	1	2	4	2	5	5	1
1	4	2	1	3	3	4	2	3	5	3	2	4	2
1	5	1	1	5	1	3	1	5	5	5	1	5	1
1	5	4	1	5	1	5	4	5	5	4	1	5	1
2	4	4	2	4	2	2	4	4	3	4	2	3	4
2	4	4	2	4	4	4	4	4	4	4	2	4	2
2	3	2	3	4	2	4	2	4	4	4	2	3	4
1	3	1	1	5	1	3	1	3	3	3	1	2	3
2	2	2	2	2	2	2	2	2	2	2	2	4	2
1	5	1	1	5	2	5	2	5	5	5	1	5	3
1	5	1	1	5	4	4	1	5	5	4	1	5	1
3	4	4	3	4	3	3	3	3	3	4	4	3	3
2	3	4	4	4	2	2	4	4	3	4	2	2	4
1	5	3	1	5	1	3	3	5	5	5	1	1	1
2	5	5	1	4	1	3	5	4	5	5	1	2	1
1	5	1	1	5	1	3	1	5	5	1	1	3	1
2	5	1	1	4	2	4	4	4	5	2	2	5	2

1	4	2	1	4	2	3	3	4	4	4	3	4	2
2	5	1	1	4	1	3	1	5	5	1	1	5	1
1	3	4	1	4	1	5	4	4	4	4	1	3	1
2	4	3	2	3	3	3	3	3	3	3	3	2	2
2	4	2	2	4	2	3	2	3	4	2	2	4	2
3	4	2	3	4	4	4	3	3	4	4	4	3	4
2	4	2	2	4	2	4	3	4	4	4	2	4	2
2	4	2	2	4	2	2	2	4	4	2	2	3	2
3	3	3	3	3	3	3	3	3	3	3	3	3	3
1	5	5	1	5	1	1	5	5	5	5	1	1	1
2	5	2	1	5	2	4	2	4	5	4	4	4	3
2	5	5	2	4	3	4	4	4	5	5	3	3	4
1	5	1	1	5	1	5	1	5	5	2	1	4	1
3	3	3	3	3	3	3	3	3	3	3	3	3	3
1	5	1	1	5	1	5	1	5	5	1	1	5	1
4	4	4	2	3	2	4	4	4	5	4	4	4	2
1	4	1	1	4	1	3	4	3	5	3	3	3	3
1	2	3	2	3	2	2	2	3	4	4	3	3	2
3	3	3	3	3	3	3	3	3	3	3	3	3	3
3	5	5	5	5	5	5	5	5	5	5	5	5	5
2	4	3	2	4	3	4	4	4	5	3	3	4	2
2	4	2	2	3	2	3	2	4	3	4	2	3	2
1	3	5	2	4	1	2	4	4	5	2	3	4	2
1	4	2	3	4	3	4	2	3	4	4	4	4	4
4	3	3	3	3	3	3	3	4	3	4	3	3	3
1	4	4	2	3	2	3	3	3	3	3	3	3	3
1	3	1	2	4	3	2	3	3	4	4	2	2	3
3	3	3	3	3	3	3	3	3	3	3	3	3	3
1	4	1	1	4	2	4	1	4	4	4	2	3	4
1	4	2	2	3	3	3	2	3	3	2	2	4	2
2	4	3	2	3	2	3	3	4	4	3	2	3	2
1	3	4	2	3	4	2	3	3	3	4	3	3	4
1	5	5	4	5	4	5	5	5	5	5	1	3	1
1	3	4	3	3	2	3	4	4	4	3	3	3	3
2	3	2	2	3	4	2	2	3	3	2	2	3	2
2	3	3	3	3	3	3	2	3	4	4	3	2	4
2	4	1	1	4	1	4	1	5	5	4	1	4	1
2	4	4	2	4	3	3	4	4	4	3	2	3	3
1	4	3	3	3	3	2	3	3	3	4	4	3	3
3	5	2	5	4	2	3	2	4	5	2	2	4	2
4	4	4	3	3	3	3	3	3	3	3	3	3	3
2	4	2	2	4	2	3	2	4	4	2	2	2	2
1	5	1	1	5	1	4	1	4	4	4	1	3	3
2	4	2	2	4	2	4	2	4	4	4	2	3	2
1	4	3	2	3	1	3	4	4	3	3	1	3	2
1	4	4	1	3	3	4	4	4	4	4	3	4	3
1	5	3	1	4	2	4	3	5	4	4	2	4	4
1	5	1	1	5	1	5	1	5	5	1	1	5	1
1	4	2	2	3	3	2	4	3	3	3	3	3	4
1	5	1	1	5	1	5	4	5	5	5	3	5	1
1	3	2	2	3	2	2	2	3	4	3	3	3	3
1	4	1	1	3	1	3	2	5	4	3	3	4	3
2	4	2	2	3	2	3	2	4	4	3	2	4	2
2	4	2	2	3	2	3	3	4	4	4	2	3	2
2	4	4	3	3	2	3	4	4	3	4	3	3	4
3	3	3	3	3	3	3	3	3	3	3	3	3	3

1	4	3	3	4	4	2	4	4	4	4	2	4	4
1	5	1	3	2	1	3	1	4	4	1	1	4	4
3	3	3	3	3	3	3	3	3	3	3	3	3	3
2	3	3	2	3	2	3	3	3	3	3	2	3	2
1	3	1	1	5	2	3	3	5	5	5	2	3	1
1	4	1	1	4	1	4	1	4	4	4	1	4	1
2	5	2	2	5	2	5	2	5	5	2	2	4	2
3	3	3	3	3	3	3	3	3	3	3	3	3	3
2	3	2	2	3	3	3	4	3	3	4	3	3	4
1	5	3	1	4	3	3	4	5	5	1	1	5	1
1	3	2	1	3	3	3	3	3	4	4	2	3	1
2	5	4	1	3	4	3	5	5	4	3	1	3	3
1	5	2	2	5	2	1	2	4	5	2	5	5	3
2	5	3	1	4	4	4	3	4	4	3	1	4	1
2	3	4	3	3	2	3	5	4	3	3	3	3	4
1	4	4	2	4	3	2	5	4	3	5	4	3	2
2	3	4	2	4	4	2	4	3	4	4	3	3	4
2	3	3	3	3	3	3	3	3	4	3	4	4	3
1	4	4	2	3	3	4	4	4	4	4	2	3	2
4	4	4	4	4	4	4	4	4	4	4	4	4	4
1	3	1	3	2	4	1	4	1	1	3	1	1	4
3	2	3	3	3	2	3	3	3	3	2	3	3	3
1	5	1	1	5	3	5	1	5	5	1	1	5	1
2	4	4	3	3	2	4	4	4	4	4	2	4	4
1	5	2	1	5	1	3	1	5	5	5	1	5	1
2	4	2	2	4	2	4	4	4	4	4	2	4	2
1	4	1	1	4	1	3	4	4	4	4	3	4	1
3	3	3	3	3	3	3	3	3	3	3	3	3	3
1	5	1	1	5	1	4	1	5	5	1	1	4	1
2	4	2	3	3	2	2	3	3	4	4	3	3	3
1	5	1	3	4	2	4	4	4	5	4	2	3	2
3	3	3	3	3	3	3	3	3	3	3	3	3	3
1	2	1	2	3	1	2	2	3	4	2	1	4	1
2	4	1	1	5	1	4	1	5	5	2	1	4	1
1	5	1	1	5	1	1	5	5	5	5	1	5	1
1	3	2	2	4	2	3	1	3	3	3	2	3	2
2	4	3	2	4	2	3	3	3	4	3	2	3	2
1	5	5	1	5	1	5	1	5	5	1	1	5	1
1	4	1	2	3	2	4	3	3	4	3	2	3	2
1	4	2	1	4	1	3	2	4	5	3	2	3	1
2	4	3	2	4	3	4	3	3	4	4	3	3	4
2	5	1	2	5	2	3	4	5	5	4	1	5	1
1	5	1	1	5	1	5	1	4	5	3	3	3	1
2	4	3	2	3	2	2	3	3	3	3	2	3	3
1	4	2	2	4	2	4	4	4	4	4	2	4	2
3	3	3	3	3	3	3	3	3	3	3	3	3	3
1	5	1	1	5	1	5	3	5	5	3	1	5	2
1	5	4	2	4	3	3	1	5	5	3	2	5	2
1	4	4	3	4	3	3	4	4	2	4	3	4	3
1	5	5	5	5	5	5	1	5	5	5	1	5	1
1	5	1	1	5	2	2	1	5	5	1	1	3	1
1	5	1	1	5	1	5	1	5	5	1	1	5	1
1	4	3	2	4	3	3	2	4	5	3	3	5	1
1	4	2	1	3	2	2	3	3	3	3	2	4	1
2	3	4	2	2	2	3	2	4	4	3	2	3	2
3	4	2	2	2	2	3	4	4	4	4	2	2	2

4	4	3	2	4	3	2	3	2	3	3	2	3	2
1	5	5	5	5	3	5	5	5	5	5	5	4	4
1	5	1	1	4	1	4	1	5	5	5	1	5	1
1	5	1	1	5	1	3	3	5	4	3	1	4	1
4	3	3	4	3	4	4	4	4	3	4	4	4	4
3	3	3	3	3	3	3	3	3	3	3	3	3	3
3	3	3	3	3	3	3	3	3	3	3	3	3	3
1	5	5	1	5	1	5	5	5	5	5	1	5	5
3	3	3	3	3	3	3	3	3	3	3	3	3	3
3	4	4	3	4	3	4	4	3	4	4	3	4	4
1	2	5	3	5	1	4	4	3	4	5	4	4	4
1	1	1	3	4	3	2	4	3	4	4	3	2	5
2	4	2	2	4	3	3	2	4	4	3	2	3	2
2	4	4	2	4	2	3	4	4	4	4	2	4	2
1	5	3	2	4	3	3	3	5	5	4	2	2	3
2	4	2	2	4	2	3	3	4	4	4	2	4	3
3	4	4	2	3	2	3	3	4	4	4	2	3	2
2	3	4	3	3	3	3	4	3	3	4	4	2	4
3	3	3	3	3	3	3	3	3	3	3	3	3	3
2	4	3	2	3	3	3	2	3	3	4	3	3	2
3	4	3	3	4	3	3	3	4	5	3	3	4	3
1	4	3	2	4	3	2	2	4	4	4	2	2	2
2	4	2	2	4	2	4	3	4	4	2	2	4	2
2	5	1	1	5	3	3	1	5	5	4	2	3	1
1	5	1	1	5	1	3	1	5	5	3	1	5	1
2	4	4	2	3	3	3	4	3	4	4	4	3	4
2	3	3	3	3	3	3	3	3	3	4	3	3	3
2	4	4	3	4	4	3	2	4	2	4	4	4	2
2	4	2	2	4	2	4	2	4	4	2	2	4	2
1	3	3	1	1	1	5	1	4	5	3	1	5	1
2	3	2	2	3	2	2	2	3	3	3	2	3	2
1	5	5	2	4	3	5	5	5	5	5	2	4	4
5	5	5	1	5	1	5	3	5	5	5	3	5	3
3	3	3	3	3	3	3	3	3	3	3	3	3	3
1	5	5	1	5	1	5	1	5	5	5	1	5	1
2	4	3	3	3	3	4	3	4	4	4	3	4	3
1	5	1	1	5	1	3	1	5	5	5	1	5	3
1	4	4	3	2	3	3	2	3	4	4	4	2	4
1	4	4	2	5	2	3	4	3	4	4	3	3	2
3	4	3	2	3	2	4	4	4	4	4	2	3	3
2	3	3	3	3	3	3	3	3	3	3	2	3	3
3	5	4	2	5	1	5	4	5	5	2	1	4	1
1	4	3	2	2	1	4	1	4	4	4	3	3	2
4	4	2	1	4	1	4	3	4	4	2	1	4	1
4	4	3	2	4	2	4	2	4	4	4	2	4	2
1	2	2	2	2	2	1	3	2	3	3	3	3	3
3	4	3	3	3	3	3	3	4	4	3	3	4	4
2	5	5	3	3	3	3	4	4	4	4	2	3	4
2	4	2	2	4	3	3	2	4	4	4	2	3	2
1	5	1	1	5	1	5	3	5	5	3	1	5	1
1	4	4	2	4	1	3	4	3	4	3	3	3	3
1	4	2	2	4	2	3	2	4	4	3	2	3	2
1	5	1	1	5	1	3	1	5	5	1	1	5	2
3	3	3	3	3	3	3	3	3	3	3	3	3	3
3	5	3	1	5	1	5	1	5	5	5	1	5	1
2	4	2	2	4	2	2	2	4	4	4	2	4	2

1	5	1	1	5	1	3	5	5	5	1	1	5	1
1	4	2	2	3	2	3	2	4	5	2	2	3	2
2	3	5	4	3	3	4	4	3	4	5	3	3	3
3	5	3	1	5	1	5	3	5	5	5	1	5	1
3	4	3	2	4	2	4	2	4	4	3	2	4	3
1	5	1	3	5	1	3	5	5	5	3	1	3	1
3	3	3	3	3	3	3	3	3	3	3	3	3	4
4	3	3	2	4	3	2	4	3	3	2	4	3	3
3	3	3	3	3	3	3	3	3	3	3	3	3	3
1	5	1	1	5	1	5	2	4	5	5	1	3	3
1	5	4	1	5	4	4	5	4	5	4	2	5	3
2	4	4	2	4	4	4	4	3	3	4	3	2	4
1	4	1	1	3	2	3	2	4	4	3	2	4	1
2	4	3	3	3	3	2	3	2	3	3	3	3	3
3	4	5	5	3	4	2	5	5	4	5	3	4	4
1	4	4	2	3	2	3	4	4	4	4	2	3	2
1	5	4	1	3	2	3	4	4	5	4	3	2	2
3	3	4	3	3	3	2	3	3	3	3	4	3	3
3	3	3	3	3	3	3	3	3	3	3	3	3	3
1	5	1	1	5	2	4	2	5	5	2	1	5	4
2	5	2	2	2	2	4	5	5	5	2	2	5	2
5	5	5	1	5	5	5	5	5	5	5	1	2	5
2	3	4	2	3	3	2	4	3	3	3	3	2	4
3	3	3	3	3	3	3	3	3	3	3	3	3	3
2	3	4	3	3	3	2	4	3	4	4	3	3	4
1	4	2	1	5	1	5	1	5	5	1	1	5	1
3	5	4	1	4	2	3	4	4	5	5	1	4	1
2	2	2	2	2	2	2	2	2	2	2	2	2	2
2	4	2	2	4	2	3	2	4	4	2	2	4	1
2	4	4	2	3	2	2	4	2	4	4	3	3	3
1	5	3	2	4	1	3	4	5	5	5	1	4	2
4	4	2	4	4	4	4	2	4	4	4	4	4	2
1	5	1	1	5	3	3	3	3	3	3	1	3	3
1	4	2	2	3	2	3	3	3	4	4	4	3	3
2	4	2	2	4	2	4	2	4	4	3	2	4	2
3	3	3	3	3	3	3	3	3	3	3	3	3	3
2	3	3	3	2	2	3	3	3	3	4	4	3	2
1	4	4	4	2	4	2	5	2	2	4	4	2	4
1	5	3	2	5	1	4	2	4	5	4	1	5	1
5	5	5	5	5	5	5	5	5	5	5	5	5	5
1	4	3	3	4	3	3	3	3	4	4	2	4	2
3	4	4	2	4	3	3	4	4	4	4	3	3	2
3	3	3	3	3	3	3	3	3	3	3	3	3	3
1	5	5	2	4	2	2	5	5	5	5	1	3	1
3	3	3	3	3	3	3	3	3	3	3	3	3	3
3	4	3	3	4	3	3	3	4	4	3	2	3	2
3	4	3	3	4	2	4	3	4	4	4	3	4	3
3	3	3	3	3	3	3	3	3	3	3	3	3	3
2	4	4	2	4	3	4	2	4	4	4	4	3	4
1	5	1	1	5	1	5	1	5	5	1	1	5	1
2	3	4	2	3	4	2	4	3	4	4	3	3	4
2	4	4	1	4	2	4	4	4	5	4	3	4	1
3	3	3	3	3	3	3	3	3	3	3	3	3	3
1	5	1	1	5	1	4	1	5	5	3	1	5	1
3	5	4	3	4	3	2	5	5	5	4	3	4	5
1	5	1	1	5	1	5	1	5	5	1	1	5	1

1	4	5	2	4	3	3	4	3	4	4	2	3	2
1	5	1	1	5	1	3	1	4	4	1	2	4	1
2	5	4	3	4	2	3	4	4	4	4	3	4	2
3	3	3	3	3	3	3	3	3	3	3	3	3	3
1	4	4	2	4	4	2	2	4	4	4	2	2	4
2	4	4	1	2	3	3	2	3	4	2	2	3	3
1	5	1	3	3	2	4	4	4	4	4	1	5	2
1	2	5	2	2	3	2	5	2	5	5	2	3	5
2	5	3	2	4	3	4	4	4	5	4	4	4	3
1	3	3	3	3	3	3	3	3	1	4	3	3	4
1	3	3	3	4	2	3	3	4	4	3	2	3	3
1	5	1	1	4	1	4	1	5	5	4	1	5	1
2	4	5	2	3	2	4	5	5	4	5	3	3	2
2	4	3	2	4	2	4	2	4	4	2	2	5	2
1	5	1	1	4	1	4	2	5	5	4	1	4	1
3	5	4	2	5	2	4	4	4	5	5	3	3	3
2	5	3	2	4	2	4	4	4	4	4	2	4	4
1	2	2	2	2	2	2	2	2	3	2	4	2	2
1	1	1	1	1	1	1	1	1	1	1	1	5	1
2	4	2	2	4	2	4	3	4	4	4	2	4	4
1	4	5	2	5	5	1	5	2	5	3	2	1	5
2	4	2	2	4	2	3	4	4	4	4	2	4	2
1	5	1	1	5	1	5	1	5	5	1	1	5	1
3	4	4	2	4	2	3	4	4	4	4	2	3	2
1	4	1	1	4	3	3	2	4	4	2	1	3	2
2	5	5	1	2	2	5	5	5	5	5	2	5	1
5	5	5	5	5	5	5	5	5	5	5	5	5	5
1	5	1	1	5	3	5	5	5	5	5	1	5	1
2	5	5	3	4	3	2	4	4	5	5	2	3	4
1	4	4	3	3	3	3	4	3	4	4	2	3	3
2	4	2	3	4	2	2	2	4	4	4	4	4	2
1	5	3	3	3	2	3	1	5	5	3	2	4	2
1	5	2	2	3	2	2	3	3	4	4	2	3	3
2	3	4	3	3	2	3	4	3	4	3	2	3	2
5	5	3	1	4	1	3	1	5	5	4	1	4	3
3	3	3	3	3	3	3	3	3	3	3	3	3	3
1	4	4	1	4	2	4	4	4	4	4	1	3	2
5	5	1	1	5	1	5	5	5	5	5	1	5	1
3	3	3	3	3	3	3	3	3	3	3	3	3	3
2	4	2	2	4	2	4	3	4	4	4	2	4	2
1	5	5	1	4	3	3	2	5	5	5	1	5	5
2	3	4	2	3	3	2	4	3	3	4	3	2	4
1	4	3	3	3	2	2	2	4	4	3	3	3	2
2	4	3	3	4	3	2	3	4	4	3	3	4	3
2	5	5	1	4	1	3	5	5	5	2	2	5	1
2	4	4	4	4	2	3	4	4	4	4	4	3	4
3	4	4	3	4	3	3	4	4	4	4	3	4	2
1	4	2	2	3	2	3	3	3	4	3	3	3	2
5	5	5	5	5	5	5	5	5	5	5	5	5	5
3	4	4	4	4	4	3	4	4	4	4	4	4	4
2	3	3	2	3	3	2	2	3	3	3	3	3	3
2	4	2	1	4	1	3	2	4	5	4	1	3	1
3	3	3	3	3	3	3	3	3	3	3	3	3	3
3	4	4	3	3	3	3	4	3	3	3	4	3	4
1	4	3	1	4	1	3	4	4	4	4	4	4	1
1	3	3	1	4	3	3	3	3	3	3	2	3	3

3	3	3	3	3	3	3	3	3	3	3	3	3	3
1	3	2	2	4	3	4	3	4	4	4	3	4	4
2	4	4	3	3	3	4	4	4	3	5	4	4	3
3	3	3	3	3	3	3	3	3	3	3	3	3	3
2	4	3	2	4	2	4	4	4	4	3	3	4	2
1	5	4	1	5	1	4	4	5	5	5	1	5	5
1	5	1	2	4	2	3	1	5	5	3	2	4	2
3	5	5	3	5	3	3	3	5	5	5	3	5	5
1	4	4	2	4	3	3	4	4	4	5	3	3	3
1	5	1	1	5	1	4	1	5	5	2	1	5	1
1	4	5	2	3	3	4	5	5	5	4	2	4	3
1	5	1	1	5	1	5	1	5	5	5	1	5	1
4	4	4	4	4	4	4	4	4	4	4	4	4	4
3	3	3	3	3	3	3	3	3	3	3	3	3	3
3	5	1	1	5	1	5	5	5	5	1	1	5	1
1	3	3	2	3	2	3	4	3	3	4	2	3	3
2	3	4	4	5	3	3	5	3	3	4	4	4	2
2	4	2	1	3	2	3	2	4	4	3	3	3	3
4	4	4	4	4	4	4	4	4	4	4	4	4	4
2	4	4	3	3	2	3	4	3	3	4	4	3	3
1	5	5	1	5	1	5	2	5	5	3	1	5	2
1	4	4	2	4	2	2	5	3	2	3	2	2	4
2	5	5	2	5	1	2	5	5	5	5	5	1	2
1	5	4	1	5	3	2	4	4	4	4	2	3	1
2	4	2	2	4	2	3	4	3	4	4	4	3	3
3	3	3	3	3	3	3	3	3	3	3	3	3	3
5	5	5	5	5	5	5	5	5	5	5	5	5	5
2	4	4	1	4	3	2	2	4	4	4	4	3	3
1	5	3	3	4	1	4	3	4	4	4	2	5	3
2	4	2	2	4	2	4	2	4	3	2	2	2	2
2	4	4	3	4	4	4	4	4	4	4	4	4	4
3	3	3	2	4	3	3	2	4	4	4	3	3	3
2	4	3	1	4	2	4	4	4	4	2	3	4	2
2	4	2	2	3	3	3	2	4	4	4	2	4	2
1	5	2	1	5	1	5	5	5	5	5	1	5	1
2	3	3	3	3	2	3	3	3	3	4	1	3	2
4	5	5	4	5	3	5	5	5	5	5	2	5	5
3	4	4	3	4	3	4	3	4	4	4	2	4	3
1	5	1	1	5	1	5	1	5	5	1	1	5	1
3	5	4	3	4	3	5	3	5	4	5	2	3	2
1	5	2	1	5	3	5	2	5	5	3	3	5	2
2	4	2	2	4	1	4	1	4	5	1	2	4	1
5	5	5	5	5	5	3	5	5	5	5	5	5	5
4	4	4	4	4	4	4	4	4	4	4	4	4	4
2	4	4	2	3	1	3	3	4	4	3	3	3	2
3	5	5	1	3	3	4	5	3	3	5	2	2	2
3	4	4	2	3	2	4	4	4	4	4	2	4	3
1	2	3	3	3	4	2	4	3	3	3	3	3	3
3	3	3	3	3	3	3	3	3	3	3	3	3	3
3	3	3	4	3	4	4	4	4	4	4	3	4	4
3	3	3	3	3	3	3	3	3	3	3	3	3	3
3	3	3	3	3	3	3	3	3	3	3	3	3	3
1	3	3	2	3	3	3	4	3	3	4	2	3	2
3	4	4	3	3	3	3	3	3	3	3	3	3	3
1	5	1	1	5	1	5	1	5	5	1	1	5	1
4	4	3	5	4	2	4	4	4	5	4	3	4	3

3	4	4	4	4	2	4	2	4	4	4	2	4	2
1	3	3	2	3	4	2	4	4	4	4	4	4	4
1	5	4	2	2	4	3	5	4	4	4	4	2	2
1	5	5	1	5	1	2	3	5	5	3	3	4	1
2	4	2	2	4	2	4	3	4	4	2	3	4	2
3	4	4	2	4	2	4	4	4	4	4	4	3	3
1	5	2	2	4	2	3	4	4	5	4	2	5	2
1	5	3	2	4	2	3	3	4	5	4	5	3	3
1	5	4	1	3	2	3	1	5	4	4	2	3	1
3	4	3	2	4	3	4	4	4	4	4	1	2	3
1	4	1	1	4	1	3	2	4	4	3	2	4	1
2	4	2	2	4	2	4	2	4	4	4	2	3	2
1	4	4	2	4	2	3	4	4	4	4	2	2	2
1	5	1	1	5	1	4	1	5	5	1	1	5	1
3	3	3	3	3	3	3	3	3	3	3	3	3	3
2	3	2	2	4	2	3	2	3	4	3	3	3	3
2	3	4	1	4	2	3	4	3	4	4	3	3	2
2	3	4	2	4	3	3	2	4	4	4	2	2	4
3	5	4	3	4	4	3	4	4	5	4	2	2	2
4	4	4	2	4	2	2	4	4	4	4	4	4	2
5	5	5	5	5	5	5	5	5	5	5	5	5	5
1	5	1	1	5	1	3	2	5	5	3	1	5	1
3	5	3	3	4	3	3	3	3	3	3	3	2	3
2	5	2	1	4	1	4	2	5	5	3	1	5	1
3	3	3	3	3	3	3	3	3	3	3	3	3	3
1	5	1	1	5	1	5	1	5	5	5	1	5	1
3	5	5	1	4	1	4	3	3	5	3	1	3	1
2	3	2	2	3	2	2	2	3	3	3	2	3	2
1	5	1	1	5	1	5	4	5	5	1	1	5	1
1	4	2	2	3	3	3	3	3	4	4	3	3	2
1	5	1	1	5	1	5	1	5	5	1	1	5	1
2	2	4	4	2	4	1	4	1	4	4	4	1	4
1	3	2	2	3	2	3	2	3	4	3	2	3	2
2	3	4	3	3	3	3	4	3	3	3	3	3	3
2	2	4	4	2	3	2	3	3	2	3	3	2	3
2	2	4	2	3	2	2	4	3	3	5	4	1	3
3	3	4	4	3	4	3	4	3	3	4	4	3	4
1	5	1	1	5	1	5	5	5	5	5	1	5	5
1	4	2	2	4	2	4	2	4	4	2	2	4	2
3	4	4	3	3	3	3	3	3	3	3	3	4	4
3	3	3	3	3	3	3	3	3	3	3	3	5	1
2	3	4	3	4	3	3	3	4	4	2	2	3	3
1	4	4	1	4	3	3	4	4	2	4	3	3	4
2	4	2	1	4	2	4	2	4	4	4	1	4	1
2	3	4	4	2	4	2	4	2	1	5	3	2	4
2	4	3	2	4	2	3	3	2	4	3	3	3	3
2	5	1	1	5	1	5	1	5	5	1	2	5	1
2	4	4	2	3	2	3	4	4	4	4	3	3	3
1	5	4	1	5	1	5	4	5	5	4	1	5	1
1	5	1	1	5	1	1	5	5	5	5	5	5	5
3	4	4	3	3	3	3	3	3	3	3	2	3	3
2	4	2	3	3	3	3	2	3	5	4	2	3	2
3	3	3	3	3	3	3	3	3	3	3	3	3	3
2	4	2	2	4	1	3	2	4	4	2	1	3	1
3	4	4	3	3	2	2	4	4	4	4	2	3	3
3	3	3	3	3	3	3	3	3	3	3	3	3	3

1	4	4	2	3	2	3	3	4	4	4	4	3	4
1	5	1	1	5	1	3	1	5	5	2	1	5	1
3	3	5	4	5	4	3	5	5	5	5	3	4	3
1	5	3	1	4	1	3	3	5	5	3	2	4	2
3	3	4	3	3	2	3	4	4	4	3	3	3	2
3	3	3	2	3	3	3	4	3	5	4	4	4	4
3	5	4	2	3	3	3	3	4	4	4	4	4	3
1	3	2	2	3	4	3	2	3	3	3	2	3	4
3	3	3	3	3	3	3	3	3	3	3	3	3	3
2	5	4	3	4	2	2	4	5	5	4	2	4	4
3	5	1	1	5	1	4	3	5	5	4	2	5	1
3	3	3	3	3	3	3	3	3	3	3	3	3	2
1	4	3	3	3	3	2	3	3	3	4	3	3	3
4	4	4	2	3	3	3	4	3	3	3	3	3	4
1	5	1	1	5	1	2	1	3	4	3	1	3	1
4	3	3	4	2	4	2	4	4	2	4	2	4	2
2	3	3	3	2	2	2	3	3	3	5	3	3	4
2	5	3	3	5	5	5	2	5	5	5	5	5	3
1	4	2	3	4	2	3	2	4	4	2	3	4	2
1	3	1	1	3	3	3	3	3	3	3	3	3	3
2	4	3	3	4	3	3	3	4	4	4	2	4	2
1	3	3	2	2	3	3	3	3	3	3	3	3	3
1	5	3	1	5	1	4	2	5	5	2	1	5	1
1	5	1	1	5	1	5	5	5	5	1	1	5	1
4	4	4	4	4	4	4	4	4	4	4	4	4	4
1	4	1	2	4	2	3	2	3	3	4	3	3	3
1	4	3	2	3	3	3	3	3	3	4	2	2	3
1	4	4	2	3	3	3	2	4	4	4	3	2	3
1	5	1	1	5	3	3	1	4	4	4	1	4	1
2	3	3	3	3	3	3	3	3	4	3	3	3	3
1	4	3	1	4	3	4	2	4	5	4	3	3	2
4	5	5	4	4	2	3	5	3	4	5	4	2	4
2	4	3	2	3	2	2	4	3	3	3	3	2	2
1	3	2	2	3	1	3	1	3	3	2	2	3	1
1	5	1	1	5	1	5	1	5	5	1	1	5	1
3	4	3	2	4	3	3	3	4	4	3	2	4	3
1	2	3	2	3	3	4	4	3	4	3	3	2	3
1	3	2	2	3	2	2	3	3	3	3	2	3	3
1	5	1	1	5	1	5	1	5	5	1	1	5	1
2	3	4	2	3	2	3	4	2	4	3	2	3	3
1	3	2	2	3	3	2	1	3	3	3	3	3	3
2	3	3	3	3	3	3	3	3	4	3	3	2	3
1	5	5	1	5	1	2	1	5	5	5	1	4	1
2	5	4	2	2	2	3	5	4	3	4	2	3	4
3	5	2	1	5	2	5	2	5	5	2	2	2	2
4	4	4	4	4	4	4	4	4	4	4	4	3	3
2	4	4	2	4	2	4	4	4	4	4	2	4	3
3	4	4	3	4	3	3	4	3	3	4	4	4	3
5	5	4	4	4	4	4	4	5	5	5	4	5	4
2	4	4	2	4	3	4	4	4	4	4	2	4	2
3	3	3	2	2	3	2	2	4	4	4	4	3	3
5	5	5	5	5	5	5	5	5	5	5	5	5	5
1	4	3	2	3	3	3	4	3	4	3	3	3	2
3	3	3	3	3	3	3	3	3	3	3	3	3	3
2	4	4	2	4	2	3	3	4	4	2	2	4	2
1	4	5	1	1	1	2	5	1	5	5	5	1	5

1	5	3	2	4	4	4	4	4	4	4	4	4	2
1	5	1	1	5	1	5	1	5	5	5	5	5	1
1	5	1	1	4	3	2	3	4	4	4	1	3	2
3	3	3	3	3	3	3	3	3	3	3	3	3	3
1	3	3	3	3	4	3	3	3	4	3	3	3	3
2	4	3	2	4	3	4	3	4	4	3	3	3	3
1	3	4	3	3	3	2	3	3	3	4	3	3	3
1	4	3	2	4	3	3	3	4	4	4	3	4	4
2	4	4	2	3	2	3	3	3	4	3	2	3	2
2	4	4	3	3	3	3	4	4	4	4	3	3	3
1	5	5	1	5	1	5	4	5	4	5	4	5	2
2	3	3	2	3	3	3	3	3	3	3	2	3	2
1	5	1	1	5	1	5	1	5	5	1	1	5	1
1	5	3	1	5	1	5	1	5	5	5	1	5	1
2	4	4	2	4	2	4	4	4	4	4	4	4	4
2	4	3	2	2	2	2	2	3	3	3	3	3	3
3	3	3	3	3	3	3	3	3	3	3	3	3	3
1	5	2	2	4	2	3	3	3	4	4	4	3	3
2	5	2	1	5	1	3	2	5	5	4	1	3	1
1	5	4	1	4	1	3	1	5	5	4	1	5	4
1	5	1	1	5	1	5	1	5	5	1	5	5	1
2	3	4	2	2	3	3	5	2	3	4	3	2	3
3	3	3	3	3	3	3	3	3	3	3	3	3	4
3	3	3	3	3	3	3	3	3	3	3	3	3	3
2	4	2	2	4	2	3	2	4	4	2	2	3	2
3	3	3	3	3	3	3	3	3	3	3	3	3	3
1	5	5	3	5	3	5	5	5	5	5	3	3	3
1	4	1	1	4	1	4	1	4	5	1	1	5	1
3	5	2	1	5	1	4	3	5	5	2	1	5	1
2	5	5	5	2	5	2	5	5	5	5	5	1	5
1	5	1	1	5	1	5	1	5	5	3	1	5	1
1	5	1	1	5	1	5	3	5	5	3	1	5	1
2	5	3	2	5	2	4	2	5	5	2	2	3	2
2	5	3	1	5	2	3	3	5	5	2	2	3	2
3	4	4	1	4	2	4	1	5	4	5	1	4	1
1	3	1	1	3	1	3	2	3	4	3	2	3	2
3	3	3	3	3	3	3	3	3	3	3	3	3	3
1	5	3	2	4	2	3	5	3	3	5	2	2	5
2	3	3	4	3	3	4	3	4	3	2	4	3	3
4	4	4	4	4	4	4	5	4	4	5	4	4	4
1	4	3	3	3	3	3	3	3	4	4	2	3	2
3	3	3	3	3	3	3	3	3	3	3	3	3	3
1	5	2	2	3	2	4	3	5	4	4	1	3	1
3	4	4	2	4	2	3	3	4	3	3	3	3	2
1	5	1	1	5	1	3	1	5	5	5	1	5	1
1	3	3	3	3	3	3	3	3	5	3	3	3	3
2	4	2	2	4	2	3	2	4	4	4	2	4	2
5	5	5	5	5	5	5	5	5	5	5	5	5	5
3	3	3	3	3	3	3	3	3	3	3	3	3	3
3	3	3	3	3	4	4	4	4	4	4	4	4	4
1	4	5	3	4	4	2	5	3	3	5	3	2	5
1	5	3	1	5	1	4	3	5	5	3	1	4	1
2	4	3	2	2	2	3	2	3	4	4	3	3	4
2	4	2	2	3	2	3	4	4	4	4	2	2	2
2	3	2	2	3	3	2	3	3	3	3	2	3	3
3	4	3	2	3	2	3	3	3	4	3	2	3	2

1	5	4	1	3	2	4	4	4	4	4	2	3	5
2	4	3	3	4	2	3	3	4	4	3	3	3	3
2	2	4	4	3	3	2	4	2	3	3	4	3	2
5	5	5	1	5	5	5	5	5	5	5	5	5	5
2	3	3	2	4	3	3	4	3	4	4	2	3	3
2	5	1	1	4	1	5	2	4	4	3	3	4	1
3	3	3	3	2	3	3	3	3	3	3	3	3	3
1	3	2	2	4	2	2	4	3	4	4	2	3	3
1	5	1	1	5	1	5	1	5	5	5	5	5	1
1	5	2	1	5	2	2	2	5	5	3	1	3	3
1	5	4	2	4	2	3	3	4	5	5	2	4	2
1	5	3	1	4	2	3	4	4	4	4	2	4	2
3	4	4	3	3	3	3	3	4	4	3	4	4	3
1	5	5	1	3	3	3	5	5	5	5	3	3	3
1	5	1	1	5	3	4	2	5	5	1	1	5	2
5	5	5	2	2	5	4	5	5	5	5	4	3	3
1	3	4	2	2	2	2	4	3	3	3	3	2	3
3	4	3	3	3	3	3	3	3	3	3	2	3	3
1	5	2	1	4	1	4	1	5	5	2	4	5	3
1	4	2	1	4	1	4	1	4	4	2	2	5	3
2	4	4	3	3	3	3	3	3	3	4	3	3	4
4	5	2	1	4	1	4	3	4	5	4	2	4	2
1	5	2	1	4	1	4	1	5	5	3	1	5	2
3	3	3	3	3	3	3	3	3	3	4	3	3	3
3	4	3	2	3	2	2	2	3	3	2	3	3	2
1	5	5	1	5	1	5	1	5	5	1	1	5	1
4	5	5	2	5	2	4	5	3	4	4	4	4	4
4	4	4	1	4	1	3	2	4	4	2	2	4	2
1	5	1	2	4	3	4	1	4	4	4	3	4	1
2	4	4	3	4	2	3	4	4	4	4	3	4	4
2	3	4	2	4	2	3	4	2	4	4	2	3	2
2	5	2	2	4	1	3	3	4	5	2	1	4	1
1	5	1	5	5	5	5	5	5	5	5	1	5	5
3	5	5	3	4	3	4	5	5	5	4	5	4	4
2	3	4	2	3	3	3	3	3	4	3	3	3	3
3	3	3	3	3	3	3	3	3	3	3	3	3	3
1	5	1	1	5	1	3	1	5	5	3	2	5	1
1	4	1	1	4	1	1	1	3	3	3	3	3	4
2	5	2	2	5	1	5	2	5	5	2	1	5	2
1	4	1	1	4	1	3	1	4	4	3	1	4	1
2	4	4	2	4	3	4	4	4	4	4	2	2	3
3	3	3	3	3	3	3	3	3	3	3	3	3	3
1	4	3	2	3	2	3	2	3	4	4	2	2	3
1	3	4	3	3	3	3	3	3	3	3	3	3	3
3	5	1	1	5	1	3	1	5	5	3	1	5	1
1	4	4	2	3	3	3	3	4	3	3	3	3	3
2	4	4	4	3	3	3	3	2	3	3	3	2	4
2	4	3	2	2	2	3	3	3	3	4	3	3	2
4	4	4	4	4	4	4	4	4	4	4	4	3	4

a63	a64	a65	a66	a67	a68	a69	a70	a71	a72	a73	a74	a75	a76
5	5	5	5	5	5	5	5	3	3	1	5	1	5
2	3	4	4	2	3	3	1	2	4	1	3	1	4
3	4	5	3	1	1	5	1	3	1	1	5	1	4
3	2	4	4	2	2	4	1	3	1	1	4	1	1
4	4	4	4	3	3	3	2	3	2	2	3	2	4
4	4	2	2	4	4	2	2	4	2	4	4	2	4
3	3	3	3	2	2	3	1	4	1	1	3	1	4
2	4	4	4	4	2	4	2	2	4	4	2	2	2
3	3	3	3	3	3	3	3	3	3	3	3	3	3
4	4	4	4	4	4	4	2	4	4	3	4	2	4
2	4	4	4	2	2	4	2	3	3	1	3	2	4
3	3	3	3	3	3	3	3	3	3	3	3	3	3
3	3	4	4	3	3	3	3	3	3	3	3	3	3
3	3	3	3	3	3	3	3	3	3	2	2	2	3
4	4	4	4	2	2	4	2	4	2	2	3	2	4
3	3	3	4	2	3	3	3	2	2	2	3	1	4
4	4	4	4	4	3	3	2	2	3	2	3	2	4
3	3	3	4	2	3	4	3	3	3	1	4	3	4
3	3	4	5	5	2	3	1	3	3	1	3	1	4
2	4	3	3	3	4	2	4	2	4	2	3	5	5
4	4	4	4	3	3	4	2	3	4	4	2	3	4
5	1	5	5	5	5	5	5	5	5	5	5	1	5
3	3	3	3	3	3	3	3	3	3	3	3	3	3
3	3	5	5	1	1	5	1	3	1	1	5	1	4
4	4	3	4	3	3	3	2	3	2	2	3	1	4
3	4	4	4	2	3	4	1	2	3	1	4	2	4
4	3	4	3	2	2	3	1	3	1	2	3	1	4
4	5	4	5	3	3	3	4	3	4	2	2	2	4
3	4	4	4	2	2	3	1	3	2	1	4	1	4
2	4	4	4	2	3	3	1	3	3	1	3	1	4
3	4	4	4	2	2	4	2	4	1	1	3	3	3
3	5	5	5	3	3	3	1	3	1	1	3	3	5
2	2	4	4	4	3	2	2	2	4	2	2	2	4
3	5	3	3	5	5	5	3	3	5	5	5	5	5
5	5	5	5	1	1	5	1	5	1	1	3	1	5
3	3	4	4	4	3	3	3	3	3	2	2	4	4
3	3	4	4	3	2	4	2	4	2	2	3	2	4
3	3	3	4	2	2	3	1	4	2	3	3	1	4
2	4	4	4	2	2	4	1	3	2	2	2	2	4
3	3	3	3	3	3	3	3	3	3	3	3	3	3
3	5	4	4	3	3	3	3	3	3	3	2	4	3
3	3	3	3	3	3	3	3	3	3	3	3	3	3
2	4	4	4	4	4	2	3	3	2	3	2	2	4
5	5	5	5	3	3	3	1	3	3	1	5	3	5
4	4	4	4	4	2	3	2	3	4	1	3	2	3
3	3	4	5	1	4	4	1	3	1	1	4	1	5
2	4	4	4	2	4	2	2	2	4	2	2	4	4
4	4	4	4	4	4	4	4	4	4	4	4	3	4
3	3	4	3	1	1	3	1	3	3	1	3	1	3
5	4	5	4	2	1	4	1	4	2	1	3	1	4

3	3	5	5	1	1	5	1	4	1	1	3	1	5
3	3	4	3	4	4	2	4	2	4	3	4	3	4
3	3	4	4	2	2	3	2	3	2	2	3	2	4
4	4	4	4	2	2	4	1	3	1	2	4	1	4
2	4	4	4	1	2	4	1	3	2	2	2	2	1
2	4	4	4	3	3	3	2	2	3	2	2	3	3
2	4	4	4	4	3	2	1	3	1	1	2	2	4
3	4	4	4	3	4	3	2	3	3	2	3	3	3
3	2	3	3	2	2	3	1	3	2	2	3	2	3
3	4	4	4	3	4	3	2	3	2	2	2	1	4
3	3	3	4	3	3	3	2	2	3	3	3	2	3
4	5	5	4	1	1	4	4	4	1	1	3	1	4
2	3	4	4	1	1	4	1	3	2	2	3	2	4
3	5	5	5	3	3	3	1	3	1	3	3	3	5
2	4	4	4	4	3	3	3	3	3	3	3	2	4
4	2	4	4	2	4	4	2	3	2	2	4	2	5
3	4	4	4	3	3	2	1	2	3	3	2	2	4
4	3	4	4	2	2	3	1	4	1	2	4	1	4
2	3	3	3	2	2	3	3	2	2	2	3	2	3
3	5	4	5	2	3	3	1	3	2	1	3	1	3
3	2	4	4	2	2	4	2	4	2	2	3	2	4
2	4	4	5	2	2	3	1	3	2	1	3	2	5
5	1	5	5	1	1	5	1	5	1	1	4	1	5
5	2	5	5	1	3	3	1	3	2	3	3	3	5
4	2	3	4	2	2	3	2	3	2	2	3	2	3
2	4	3	3	4	4	2	2	1	5	2	2	3	2
3	4	4	4	1	1	3	1	3	1	1	3	1	4
3	1	3	2	2	2	3	1	3	1	1	3	1	3
4	4	5	5	2	3	4	1	4	2	1	3	3	4
2	2	4	4	2	3	3	2	2	2	2	2	1	2
4	4	4	3	3	4	3	3	3	3	2	3	2	4
3	4	4	4	1	2	4	1	3	2	1	4	2	4
3	4	4	4	4	3	2	3	3	3	3	3	2	3
3	3	3	3	4	3	4	2	3	3	3	3	1	3
4	2	5	5	1	1	5	1	5	1	1	4	1	4
3	3	3	3	3	3	3	3	3	3	3	3	3	3
4	4	4	4	3	2	3	2	3	3	3	3	3	3
3	3	4	4	2	2	2	2	3	2	2	3	2	4
4	2	3	4	2	3	3	3	3	3	3	3	3	3
5	1	5	5	1	1	5	1	5	1	1	1	1	5
3	4	4	4	3	4	2	2	3	2	3	2	3	4
2	3	3	3	3	2	2	3	3	1	2	3	2	3
3	4	4	4	2	3	4	2	3	4	2	3	2	4
3	4	5	5	2	2	4	1	3	3	2	4	4	4
5	1	5	5	1	1	5	1	5	1	1	5	1	5
5	4	5	5	2	1	5	1	5	1	1	5	1	5
3	4	3	4	2	2	3	2	3	3	1	3	1	3
4	3	4	4	3	4	3	4	4	4	3	4	4	3
1	4	4	4	4	5	3	3	4	3	2	2	3	4
3	3	3	3	2	2	3	2	3	2	3	2	2	3
3	4	4	4	2	1	3	1	3	2	1	3	2	4
4	4	5	5	2	1	4	2	4	4	1	3	2	4
4	4	4	4	2	3	3	2	4	2	2	4	4	4
3	2	4	5	2	1	3	1	3	2	1	4	1	3
4	5	4	5	2	2	4	2	3	2	2	2	2	4
1	2	5	5	1	1	5	1	3	2	1	2	1	3

5	3	5	5	1	1	5	1	5	1	1	3	1	5
2	3	3	2	3	3	3	3	3	3	3	2	2	3
2	2	4	3	3	4	3	2	2	3	2	3	2	3
2	3	5	5	2	1	5	1	2	2	1	3	1	5
3	3	4	3	3	3	3	3	3	2	3	3	2	4
3	3	3	3	3	3	3	3	3	3	3	3	3	3
4	3	4	4	1	1	4	1	4	1	1	4	1	4
4	4	4	4	2	2	4	1	3	2	2	4	3	4
4	3	2	4	2	3	4	4	3	4	3	4	2	5
3	2	4	4	2	2	4	2	3	2	2	4	2	4
1	5	5	5	1	1	4	3	3	3	2	1	1	5
3	4	4	4	2	4	3	2	2	3	2	3	3	2
3	4	3	3	3	3	3	3	3	3	3	3	3	3
2	4	4	4	3	3	2	2	2	4	2	2	3	4
3	5	4	4	3	3	3	4	3	5	4	1	4	5
2	2	2	2	2	2	3	3	3	2	2	2	3	4
5	5	5	5	5	5	5	5	5	5	5	5	5	5
2	5	4	4	2	3	3	2	2	3	1	1	4	1
5	1	5	5	1	1	5	1	5	1	5	5	1	5
1	5	5	5	5	3	2	1	2	5	2	3	5	5
3	5	5	5	4	4	2	2	2	3	2	3	3	5
3	3	3	4	3	4	3	2	3	4	3	3	3	3
3	4	4	4	2	3	3	1	3	2	1	4	2	4
4	4	4	4	2	3	3	2	3	2	2	4	2	4
3	4	5	4	3	3	3	3	3	3	2	3	3	4
3	3	4	4	2	2	3	2	3	2	2	3	2	2
4	3	5	5	1	1	4	1	4	1	1	4	1	5
3	4	4	4	4	3	4	2	3	3	3	2	3	3
1	5	5	5	5	5	5	1	1	5	1	1	3	5
4	2	4	4	2	3	3	1	4	1	1	2	1	4
3	3	3	3	3	3	3	3	3	3	3	3	3	3
2	3	3	3	4	4	3	3	2	4	3	3	4	3
4	3	3	3	2	2	4	2	3	2	2	3	2	2
3	4	4	4	3	5	4	4	2	3	2	3	5	1
2	3	4	4	2	2	3	2	3	2	2	3	2	3
3	4	5	5	2	2	4	2	4	1	1	3	2	4
4	5	5	3	1	1	4	3	5	1	1	1	1	4
3	4	4	4	4	3	3	3	3	2	1	3	1	5
3	3	3	3	3	3	3	3	3	3	3	3	3	3
3	5	5	5	5	3	1	4	2	1	2	3	1	5
2	4	3	3	4	3	2	2	3	3	3	3	3	2
2	4	4	4	3	3	3	2	2	2	3	3	3	4
3	3	3	3	3	3	3	3	3	3	3	3	3	3
4	4	4	4	2	2	4	2	4	2	2	3	2	4
3	2	3	3	2	2	3	2	3	2	2	2	2	3
3	2	3	2	1	3	3	1	2	3	1	2	2	2
2	3	2	4	3	2	4	2	5	3	2	5	2	5
3	3	3	4	2	3	3	1	3	2	2	3	2	3
3	4	5	5	3	2	4	1	4	1	1	4	3	4
5	5	5	5	5	5	5	5	5	5	5	5	5	5
5	3	5	5	1	1	5	1	5	1	1	5	1	5
3	4	5	5	2	2	4	2	4	2	1	4	1	5
3	3	3	3	3	3	3	3	3	3	3	3	3	3
4	4	5	5	1	1	4	1	5	1	1	5	1	4
3	3	3	3	2	3	2	3	2	3	3	3	3	3
3	3	4	4	2	2	3	2	4	2	2	4	3	4

4	3	4	4	2	2	4	1	3	3	2	4	1	4
4	4	5	5	2	3	4	1	4	3	2	4	1	5
3	4	4	4	2	2	4	2	4	3	2	4	3	4
3	3	4	4	3	3	3	3	3	3	2	3	3	3
4	4	5	4	5	4	5	4	5	5	5	5	2	5
4	5	5	5	3	2	3	1	2	1	1	4	2	5
4	5	5	5	1	1	4	1	3	1	1	2	1	3
3	3	3	3	3	3	3	3	3	3	3	3	3	3
3	4	4	4	4	4	3	3	3	3	3	3	3	3
5	2	4	4	4	2	4	2	2	2	4	4	2	4
4	5	5	5	4	3	4	1	3	3	1	2	1	4
4	3	4	4	3	2	3	1	3	2	1	2	2	4
5	5	5	4	3	3	3	1	3	1	1	3	1	5
4	4	5	5	4	2	4	1	4	2	1	3	2	5
3	3	4	4	1	2	4	1	3	1	1	1	1	3
3	3	3	3	3	3	3	3	3	3	3	3	3	3
1	5	5	5	5	1	5	1	5	1	1	3	1	3
3	3	3	3	3	3	3	3	3	3	3	3	3	3
4	4	4	4	2	2	4	2	4	2	2	4	2	4
2	4	4	4	5	4	3	2	2	5	3	2	4	3
3	3	3	3	3	3	3	3	3	3	3	3	3	3
2	4	4	4	3	3	2	1	4	4	2	5	1	5
3	4	5	5	2	3	3	2	2	4	2	4	3	5
3	3	3	3	3	3	3	3	3	3	3	3	3	3
3	3	3	3	3	4	3	3	3	4	4	3	3	3
3	4	4	4	4	3	3	3	3	4	3	3	3	4
4	5	5	5	1	1	5	1	4	2	2	2	1	5
3	3	4	4	3	3	5	2	4	3	2	4	4	4
3	3	3	3	3	3	3	3	3	3	3	3	3	3
5	5	5	3	4	2	3	2	2	3	2	4	2	2
2	3	4	4	2	2	3	1	2	2	2	2	2	3
5	5	5	5	3	2	5	1	3	2	1	5	3	5
4	4	5	5	1	1	5	1	5	1	1	4	1	5
4	2	5	4	1	1	5	1	5	1	1	4	1	4
4	4	4	4	2	2	3	2	3	3	2	4	2	4
2	3	4	4	2	2	4	2	3	3	2	2	2	4
4	2	3	3	2	2	4	2	4	2	2	4	2	4
3	4	4	4	3	2	2	1	3	2	1	3	1	3
3	4	3	4	2	3	3	2	3	2	2	3	2	3
3	2	4	3	2	2	4	2	3	2	2	3	2	3
3	3	3	3	3	3	3	3	3	3	3	3	3	3
5	2	5	5	2	2	5	2	5	2	2	5	2	5
3	5	4	4	5	4	3	5	3	4	3	2	3	4
1	4	4	5	3	3	3	1	2	3	2	3	4	2
3	4	3	4	2	3	3	2	3	3	3	3	2	4
2	5	4	4	4	4	3	2	2	4	2	2	4	4
3	4	4	4	2	2	3	2	3	2	2	3	2	3
2	5	4	4	3	3	3	2	2	3	2	1	1	5
3	4	4	4	2	1	4	1	3	2	1	3	1	3
3	4	4	4	2	2	4	2	3	2	2	3	2	4
4	4	4	4	4	3	2	1	3	5	3	2	4	4
5	4	5	5	1	1	5	1	5	1	1	5	3	5
3	3	3	4	2	3	4	2	3	3	2	3	2	3
3	4	4	4	3	3	3	2	3	3	2	3	2	4
3	3	3	3	2	3	3	2	2	3	2	3	2	4
3	2	4	4	3	2	3	2	3	2	2	2	1	3

3	3	3	4	4	3	4	2	3	4	2	3	3	4
3	4	3	3	3	3	3	3	2	3	3	3	3	3
4	3	4	4	1	1	4	1	4	1	1	4	1	4
2	4	3	3	4	3	3	4	2	4	3	1	3	2
3	3	3	3	3	3	3	3	3	3	3	3	3	3
5	1	5	5	1	1	5	1	5	1	1	5	1	5
3	4	4	4	3	3	3	3	2	2	2	3	2	3
3	5	5	5	2	2	3	1	3	3	1	2	1	5
4	4	5	5	2	2	4	1	3	2	1	3	1	4
2	2	4	4	2	2	4	2	2	2	2	2	2	2
2	4	5	5	2	1	4	1	2	3	2	3	2	5
3	4	5	5	1	2	5	1	3	1	1	4	1	5
4	3	4	4	1	1	4	1	3	2	1	3	1	3
4	4	4	4	1	1	3	1	4	1	1	1	1	2
2	3	4	4	2	2	3	2	2	2	2	2	2	4
3	5	4	5	1	1	4	1	3	1	1	4	1	1
5	1	5	5	1	1	5	3	5	1	1	5	1	1
3	3	3	3	3	3	3	3	3	3	3	3	3	3
3	4	4	4	2	3	3	3	3	3	3	3	3	4
1	3	4	4	2	3	3	1	3	1	1	3	1	3
2	3	4	4	2	2	4	1	3	2	1	4	2	5
4	5	2	4	3	5	1	1	3	5	1	3	1	3
4	5	4	5	2	2	4	1	4	1	2	5	2	4
3	2	4	4	2	2	3	1	3	2	1	2	2	4
4	4	4	5	3	3	3	3	3	5	1	2	4	4
2	3	4	4	2	2	4	2	3	4	2	2	2	4
4	5	5	5	1	2	4	1	4	1	1	4	1	4
2	4	5	4	3	2	2	3	3	2	2	2	2	2
4	3	4	4	2	2	4	2	3	3	2	4	2	4
3	4	4	4	2	4	3	1	3	2	1	2	1	4
3	4	4	5	4	3	3	4	3	4	3	2	3	3
4	4	4	4	3	4	3	1	3	3	3	3	4	4
3	5	4	4	1	1	4	1	4	1	1	3	1	4
3	4	5	4	2	2	3	2	3	3	2	2	2	3
4	4	4	4	2	3	4	1	3	2	1	3	1	5
5	5	5	5	1	1	5	1	5	1	1	1	1	5
4	4	4	3	3	4	3	2	3	4	2	1	1	4
4	1	5	5	1	1	5	1	4	1	1	4	1	4
2	4	5	5	3	3	3	1	2	3	1	3	2	3
3	4	5	5	1	1	4	1	3	1	1	4	1	4
4	5	4	4	2	2	3	2	3	4	2	3	2	4
3	5	5	5	1	3	3	1	1	1	1	5	1	5
1	5	5	5	1	1	5	1	1	1	1	5	1	5
2	4	4	4	2	3	3	2	3	2	2	3	3	2
3	5	5	5	3	4	4	3	3	2	2	3	1	5
4	5	5	5	2	3	2	1	2	4	1	2	1	5
3	5	4	3	4	4	3	3	3	3	2	2	1	1
4	4	4	4	4	4	2	3	4	3	1	3	1	4
2	2	3	3	3	3	3	2	2	3	2	2	4	3
5	4	5	5	2	3	3	2	4	2	2	5	3	2
2	4	4	5	3	3	3	2	3	2	2	2	3	4
4	4	4	4	2	2	4	2	3	2	2	3	2	4
3	4	5	4	1	1	4	1	3	1	1	4	1	4
4	4	4	4	3	3	3	2	4	3	3	4	1	4
3	4	4	4	2	2	3	2	3	2	2	4	3	4
3	2	4	4	2	1	3	1	3	2	1	3	1	4

4	4	5	5	1	2	5	1	3	4	1	5	2	5
3	4	4	4	3	3	3	1	3	2	2	4	2	4
3	3	4	4	1	2	4	1	3	3	1	4	2	4
3	3	4	4	3	2	2	2	2	3	2	4	3	4
3	1	5	5	1	3	5	1	3	1	1	5	1	5
4	4	4	4	2	2	4	2	4	2	2	4	2	4
3	3	3	3	3	3	3	3	3	3	3	3	3	3
3	3	3	4	3	4	2	4	2	4	2	2	1	3
2	4	4	3	4	4	3	4	3	4	3	2	4	3
5	3	5	5	1	1	5	1	5	2	1	2	3	5
3	3	3	3	3	3	3	3	3	3	3	3	3	3
4	4	4	4	1	1	4	1	4	1	1	3	1	4
1	5	5	5	2	2	2	5	1	5	2	1	5	1
4	5	5	5	3	3	3	2	4	1	1	1	1	5
4	4	4	5	4	4	4	5	4	4	4	4	4	4
5	5	5	5	5	5	5	5	5	5	5	5	5	5
3	2	5	5	3	2	4	1	3	4	2	3	1	5
3	3	3	3	3	3	3	3	3	3	3	3	3	3
3	3	3	3	3	3	3	3	3	3	3	3	3	3
3	4	4	4	2	2	2	4	4	4	2	3	1	4
2	4	4	4	2	2	4	2	2	2	2	4	2	4
3	3	3	3	3	3	3	3	3	3	3	3	3	3
3	3	4	4	2	2	3	3	2	2	2	3	3	3
1	5	5	5	3	4	2	2	3	4	2	2	2	5
3	3	5	4	2	2	4	2	2	2	1	3	1	5
4	2	4	4	2	2	4	2	4	2	4	4	4	4
3	4	4	4	3	4	3	2	3	3	2	2	2	3
3	3	3	3	3	3	3	3	3	4	3	3	2	3
3	3	4	4	2	2	4	2	4	2	2	2	2	3
3	3	5	5	1	1	4	1	3	1	1	3	1	4
3	1	4	4	1	1	4	1	4	1	1	4	1	4
3	3	4	3	2	2	3	2	3	4	2	3	2	4
5	1	5	3	1	1	5	1	3	1	1	3	1	5
3	3	3	4	3	3	3	3	3	3	3	2	3	3
2	5	5	5	2	4	4	2	2	2	2	2	5	5
3	4	4	4	1	1	4	1	4	1	1	1	1	4
3	4	5	5	1	1	4	1	3	1	1	2	1	5
2	4	4	4	2	4	2	1	2	1	1	4	3	4
2	2	5	4	2	2	4	1	3	1	1	3	1	3
2	2	5	4	2	2	4	1	3	1	1	3	1	3
3	2	4	5	1	1	3	2	3	2	1	3	1	3
5	5	5	5	1	3	5	1	5	1	1	5	1	5
2	5	4	4	2	3	3	1	3	3	1	2	3	4
5	5	5	5	1	1	5	5	5	1	1	5	1	5
3	4	4	3	2	2	4	1	3	2	1	4	4	3
4	3	5	4	1	1	4	1	2	1	1	5	1	3
5	5	5	5	5	5	5	5	5	5	5	5	5	5
4	3	5	5	1	1	4	1	3	1	1	3	1	4
3	2	3	3	3	3	3	2	3	2	2	3	2	4
3	3	5	5	1	2	3	1	3	1	1	4	1	4
2	4	4	5	3	3	3	3	3	3	3	3	3	4
1	1	1	4	1	5	3	3	1	3	3	1	4	1
4	4	4	3	2	3	3	2	3	2	2	3	4	4
4	5	5	5	1	3	4	1	4	1	1	5	1	5
3	3	3	3	3	3	3	3	3	3	3	3	3	3
4	5	5	5	3	2	3	1	4	3	1	2	1	5

5	5	5	5	5	5	5	5	5	5	5	5	5	5
2	4	4	4	2	2	3	1	3	3	1	3	1	4
5	3	3	5	5	5	3	3	4	3	3	3	3	3
4	1	4	4	1	1	4	1	3	3	1	4	1	4
3	4	4	4	2	4	4	2	2	2	2	4	2	4
3	4	3	4	3	3	3	2	3	4	3	3	3	2
3	4	4	4	3	3	3	1	3	2	2	4	2	4
3	3	3	3	3	3	3	3	3	3	3	3	3	3
4	3	4	4	2	2	3	2	3	2	2	2	2	3
3	3	3	3	3	3	3	3	3	3	3	3	3	3
3	3	4	4	2	2	3	2	3	3	2	3	2	3
5	5	5	5	5	1	5	1	5	1	1	5	1	5
4	4	4	4	4	4	4	2	4	2	2	4	2	4
4	4	4	4	4	4	4	4	4	4	4	4	4	4
4	1	4	3	1	1	5	1	5	1	1	3	1	3
3	3	4	4	2	2	4	1	2	3	2	3	3	4
5	4	5	5	1	1	5	1	5	1	1	5	1	5
1	5	5	5	1	2	4	1	2	1	1	5	1	4
2	3	3	3	3	3	3	2	2	3	2	3	2	3
5	1	5	5	1	5	4	1	5	1	1	5	4	5
3	5	5	5	2	2	4	1	3	4	2	4	3	4
3	4	4	4	2	2	3	2	3	3	2	3	2	4
4	3	4	4	2	2	4	2	4	2	2	4	2	4
3	4	4	4	3	3	3	2	3	2	3	4	3	4
3	3	5	5	1	1	5	1	3	3	3	3	3	3
4	4	4	4	2	2	4	2	4	2	2	3	2	4
3	2	4	4	3	3	3	2	2	3	2	3	2	4
3	4	5	5	3	3	4	2	3	2	2	4	2	4
1	1	5	5	5	1	5	1	5	1	1	5	1	5
2	4	4	4	2	2	3	2	3	2	2	3	2	4
4	3	4	4	3	3	3	2	4	2	3	3	2	4
3	3	4	4	3	3	2	3	2	3	2	2	3	3
3	4	4	3	2	2	3	2	4	2	2	3	2	4
3	4	4	4	2	1	4	1	3	3	2	4	2	2
2	3	3	3	2	2	3	2	2	2	2	2	2	3
4	3	4	4	4	3	4	1	4	4	3	4	1	5
3	4	5	5	3	3	3	1	2	5	2	3	4	5
3	4	4	4	2	3	3	2	3	3	2	3	2	4
2	4	4	4	3	4	3	2	3	2	1	3	2	4
4	2	4	5	1	3	4	1	4	3	1	3	2	2
3	4	4	4	4	3	3	2	3	4	2	4	4	5
4	3	4	3	4	3	4	3	4	3	4	3	4	3
2	5	4	4	3	3	3	3	2	4	3	3	4	5
3	4	4	4	2	3	3	2	3	2	2	3	2	3
3	3	3	3	3	3	3	3	3	3	3	3	3	3
4	5	5	5	1	1	5	1	4	1	1	4	1	4
4	3	4	5	1	1	4	1	3	3	1	4	1	5
1	5	3	4	4	3	3	2	2	3	3	2	2	3
3	3	5	3	1	1	5	1	3	1	1	4	1	4
4	3	4	4	3	3	3	2	3	2	3	3	2	4
3	4	4	4	1	1	4	1	3	3	1	4	3	4
3	4	4	4	2	2	3	2	3	2	2	3	1	4
5	5	5	5	5	5	5	5	5	5	5	5	5	5
2	4	4	4	2	3	2	2	2	2	2	4	4	3
3	4	5	5	2	2	4	1	2	2	2	5	2	5
3	3	3	3	3	3	3	3	3	3	3	3	3	3

3	3	3	3	3	3	2	3	3	3	3	3	2	3
4	5	5	5	1	2	4	1	3	5	1	5	4	5
5	1	5	5	1	1	5	1	5	1	1	5	1	5
3	5	5	5	1	3	3	1	4	3	1	3	1	4
3	4	4	4	3	3	3	2	3	3	3	3	3	3
2	4	4	4	2	1	3	1	4	3	1	3	2	4
3	3	4	5	2	2	3	2	3	2	1	4	1	4
4	4	4	4	2	2	4	1	3	3	2	2	1	4
3	3	5	5	1	1	5	1	3	1	1	5	1	5
3	3	3	3	3	3	3	3	3	3	3	3	3	3
4	4	4	4	4	3	4	2	4	3	2	4	1	5
3	3	4	4	2	2	3	2	3	2	2	3	2	4
2	5	4	3	4	4	2	2	1	5	3	2	4	3
4	4	5	5	3	3	4	5	4	4	1	2	5	5
3	4	2	3	3	3	2	4	3	3	3	3	3	2
3	3	3	3	3	3	3	3	3	3	3	3	3	3
3	4	4	4	4	2	3	1	4	3	1	3	1	5
5	4	5	5	1	2	4	1	3	1	1	4	1	5
4	4	4	4	2	2	3	1	4	3	1	3	1	4
4	4	4	4	2	2	4	2	4	2	2	4	2	4
3	3	3	3	3	3	3	3	3	3	3	3	3	3
3	5	4	3	2	2	3	3	2	2	3	2	2	2
3	5	3	4	3	3	3	4	3	4	3	4	3	4
3	2	5	5	1	1	5	1	3	1	1	5	1	5
3	4	4	4	3	3	3	2	3	3	2	3	2	3
3	4	4	4	2	2	4	2	3	2	2	2	2	4
2	3	5	5	2	4	1	1	2	3	1	1	4	3
4	3	4	4	3	2	3	3	4	3	2	2	2	3
2	4	2	4	5	4	2	3	2	4	3	3	2	3
2	5	5	5	4	4	2	3	2	5	2	2	3	5
4	2	4	3	3	3	3	3	3	3	3	3	3	3
4	5	5	5	1	2	4	1	4	4	1	4	1	5
4	3	5	5	1	2	5	1	4	2	2	4	1	5
3	4	5	4	3	2	4	1	4	1	1	1	1	3
2	4	3	3	2	3	2	1	2	3	1	2	4	3
2	4	4	4	2	2	3	3	3	3	2	4	3	4
2	4	4	4	4	3	3	3	2	3	1	1	1	2
5	5	5	5	5	5	5	5	5	5	5	5	5	5
4	3	4	4	2	2	4	1	3	2	2	3	2	4
4	4	4	4	2	2	4	1	3	1	4	4	4	4
4	4	4	4	3	4	4	2	4	2	2	4	2	4
5	5	5	5	1	1	5	1	5	1	1	1	1	5
5	4	5	5	1	1	5	1	5	3	1	5	4	5
2	4	4	4	2	2	3	2	3	3	1	4	4	5
4	2	4	4	2	2	4	2	4	2	2	4	2	4
3	4	4	4	2	2	3	2	3	2	2	3	2	4
3	4	4	3	1	2	3	2	3	2	1	3	1	3
3	3	3	3	3	3	3	3	3	3	3	3	3	3
3	3	5	5	2	1	5	1	5	2	1	5	1	5
5	1	5	5	1	1	5	1	5	1	1	5	1	5
2	4	4	4	3	3	3	3	3	3	3	3	2	3
2	4	4	4	2	4	2	2	2	2	2	2	2	4
4	4	5	5	4	3	4	1	3	2	1	1	1	5
3	5	5	5	1	1	4	1	3	1	1	3	4	5
3	3	5	5	1	1	5	1	5	1	1	1	2	5
4	2	5	5	1	2	4	2	4	2	2	4	1	4

2	2	4	4	2	3	3	4	2	1	2	3	2	4
4	5	5	5	1	2	5	1	4	1	1	5	1	4
3	4	5	5	1	1	4	1	3	1	1	4	1	3
3	2	3	3	2	2	2	2	3	2	2	3	2	3
3	4	4	4	2	2	4	2	4	2	2	3	2	3
3	4	4	4	3	3	3	3	3	4	3	3	4	3
4	4	4	4	2	2	4	2	4	2	2	4	2	4
3	3	4	4	2	2	2	2	2	3	2	4	2	4
4	3	2	4	4	3	4	2	4	4	2	4	4	4
3	5	5	5	1	1	3	1	1	5	1	1	1	5
4	3	5	5	2	2	4	1	5	1	1	1	1	4
3	5	5	5	2	2	4	1	3	4	1	3	2	5
3	1	5	5	1	1	5	1	3	1	1	5	1	5
3	3	3	3	3	3	3	3	3	3	3	3	3	3
5	1	5	5	1	1	5	1	5	1	1	5	1	5
2	4	4	4	4	4	4	2	4	4	2	4	2	5
4	4	4	4	1	1	3	1	4	1	2	2	1	4
4	4	4	4	2	2	4	2	4	4	2	4	4	4
3	3	3	3	3	3	3	3	3	3	3	3	3	3
5	5	5	5	1	1	5	1	5	1	1	5	1	5
3	5	4	4	2	2	3	1	2	2	1	3	4	4
2	3	4	3	2	2	3	2	2	3	2	3	2	3
3	4	5	1	1	2	4	1	3	1	1	3	1	4
4	4	4	4	2	2	3	2	4	2	2	4	2	4
3	4	3	3	4	3	3	3	3	4	3	3	3	3
3	3	3	3	3	2	4	2	2	2	2	3	2	3
2	4	4	3	2	2	3	1	2	2	2	2	4	4
3	3	3	3	3	3	3	3	3	3	3	3	3	3
3	1	4	4	1	1	4	1	4	1	1	3	1	4
3	2	3	4	2	2	4	2	3	2	2	3	2	2
2	4	4	4	3	2	4	2	3	3	2	3	2	4
2	4	4	4	2	4	2	3	2	4	1	3	2	2
5	4	5	5	1	1	4	1	3	4	1	5	4	5
3	4	4	5	1	2	4	1	3	2	2	3	3	4
3	2	3	3	2	2	3	2	3	2	2	3	2	3
4	4	4	4	2	2	2	2	3	4	2	2	2	3
5	3	5	5	1	1	5	1	4	1	1	5	2	5
3	2	4	4	3	2	4	2	3	3	2	2	3	4
2	4	3	3	3	3	3	2	3	3	3	3	2	3
4	4	4	4	2	2	4	1	3	2	2	4	2	4
3	3	3	3	3	3	3	3	3	3	3	3	3	3
4	2	4	4	2	2	3	1	4	2	2	2	2	4
4	4	5	5	1	1	4	2	4	4	1	4	1	3
4	4	4	4	3	2	4	2	4	2	2	4	2	4
3	4	4	3	2	2	4	2	3	2	1	3	1	3
3	2	4	4	3	2	4	2	3	3	2	3	1	4
3	3	4	5	2	2	5	1	4	1	1	4	1	4
5	5	5	5	1	1	5	1	5	1	1	5	1	1
2	4	4	4	4	2	3	1	2	2	2	1	1	2
5	3	5	5	1	3	5	2	5	2	1	1	1	5
3	3	4	4	2	2	3	1	3	3	2	3	2	4
5	3	5	4	2	2	4	2	3	2	1	3	1	5
3	3	4	4	2	2	4	2	3	2	2	3	1	4
3	3	3	4	3	2	2	2	3	3	2	3	3	3
2	4	4	4	3	4	3	2	2	4	2	4	2	4
3	3	3	3	3	3	3	3	3	3	3	3	3	3

2	4	4	4	4	3	3	2	2	3	1	4	1	4
3	5	5	5	1	1	3	1	3	1	1	3	1	5
3	3	3	3	3	3	3	3	3	3	3	3	3	3
3	4	3	3	3	3	3	2	2	2	2	2	2	3
4	5	5	5	1	3	3	1	3	1	1	3	1	5
3	4	4	4	1	1	4	4	4	1	1	4	1	4
4	2	4	2	2	2	5	2	5	2	2	4	2	5
3	3	3	3	3	3	3	3	3	3	3	3	3	3
2	5	4	4	3	3	3	2	2	4	2	2	5	3
3	5	5	5	1	1	5	1	4	2	1	4	1	4
2	4	4	4	2	1	4	1	3	2	1	4	1	4
4	5	5	5	3	2	3	2	3	5	2	3	1	5
5	4	5	5	3	2	3	1	5	2	2	5	2	5
4	4	5	5	1	1	4	1	4	2	1	3	1	4
3	5	5	5	3	4	2	3	3	4	3	3	4	4
1	5	5	5	4	3	3	2	2	3	1	3	2	5
4	4	4	4	3	3	3	1	3	2	3	4	1	4
3	4	4	4	2	4	2	1	3	3	3	3	1	3
3	3	4	4	2	2	3	1	4	2	2	4	2	4
4	4	4	4	4	4	4	4	4	4	5	4	5	4
1	5	1	1	4	3	2	4	1	4	3	1	1	1
3	2	3	3	2	3	3	4	3	3	4	3	3	4
3	1	5	5	1	1	5	1	5	1	1	5	1	5
3	4	4	4	3	3	4	1	3	3	3	3	2	4
2	2	5	5	1	1	5	1	4	1	1	5	1	5
3	3	4	5	2	3	3	1	4	2	2	4	3	4
3	4	4	4	2	1	4	1	4	2	2	3	1	5
3	3	3	3	3	3	3	3	3	3	3	3	3	3
4	3	5	5	2	2	4	1	4	1	1	4	1	5
2	3	4	4	2	2	3	1	3	3	1	3	3	3
4	4	5	5	2	2	4	1	3	3	1	3	2	4
3	3	3	3	3	3	3	3	3	3	3	3	3	3
3	2	3	3	2	2	3	1	3	2	2	2	1	3
4	2	5	5	1	2	4	1	5	1	1	2	1	5
5	1	5	5	1	1	5	1	1	1	1	5	1	5
2	2	3	3	2	2	3	2	3	2	2	3	3	3
3	2	4	4	2	2	4	1	3	2	2	3	2	4
5	1	5	5	1	1	5	1	5	1	1	5	1	5
5	4	3	3	3	3	3	2	3	2	2	2	1	4
4	4	4	4	2	1	3	1	1	3	1	4	2	4
4	4	4	4	3	3	4	2	3	3	3	3	2	4
5	5	5	5	2	1	5	1	5	1	1	4	2	5
5	2	5	5	1	1	4	1	4	1	1	3	1	5
3	3	3	3	3	3	3	2	3	2	2	3	2	3
4	4	4	4	2	2	4	2	4	2	2	3	1	5
3	3	3	3	3	3	3	3	3	3	3	3	3	3
5	3	5	5	1	1	5	1	5	1	1	5	1	5
4	5	5	5	2	2	4	1	3	2	1	4	1	4
4	4	4	5	5	4	3	4	3	3	1	1	1	4
5	1	5	5	1	1	5	1	3	1	1	5	1	5
1	5	5	5	1	1	3	1	2	1	1	3	1	5
5	1	5	5	1	1	5	1	3	1	1	5	1	5
3	3	4	4	1	2	4	1	3	1	1	4	1	4
3	3	3	5	2	1	3	1	2	2	1	4	4	5
2	3	4	4	2	3	3	1	3	2	1	2	2	4
3	5	4	4	2	2	4	3	3	5	2	3	1	5

4	2	4	4	4	3	3	2	4	2	2	2	2	4
4	5	5	5	2	2	5	1	4	4	1	5	2	5
5	5	5	5	1	1	5	1	3	1	1	2	1	5
3	3	5	5	1	1	3	1	3	1	1	3	1	4
4	4	4	4	4	4	3	4	3	4	4	4	4	4
3	3	3	3	3	3	3	3	3	3	3	3	3	3
3	3	3	3	3	3	3	3	3	3	3	3	3	3
5	5	5	5	1	3	5	1	5	5	1	5	5	5
3	3	3	3	3	3	3	3	3	3	3	3	3	3
3	4	4	4	3	3	4	2	3	2	2	3	2	4
4	5	5	5	3	4	3	1	3	2	2	2	1	3
2	4	5	2	1	3	3	2	3	5	2	3	2	3
3	2	4	4	2	2	4	2	3	2	2	4	2	4
2	2	4	4	2	4	4	2	3	2	2	2	2	2
3	4	5	5	2	4	3	1	3	3	1	2	2	4
4	3	4	4	2	3	4	2	3	3	2	3	3	4
3	4	4	5	2	2	5	2	4	2	2	4	2	4
3	4	4	4	4	3	2	3	3	2	3	2	4	3
3	3	3	3	3	3	3	3	3	3	3	3	3	3
3	4	4	4	4	2	4	3	2	3	2	3	4	3
3	4	5	4	4	3	3	1	4	2	2	3	2	4
3	4	4	4	2	2	4	2	2	3	2	3	2	3
4	3	4	4	2	3	4	2	4	2	2	4	2	4
2	3	5	5	1	2	4	1	3	2	1	4	2	5
3	3	5	5	1	1	5	1	3	1	1	5	1	5
3	3	4	4	3	5	3	2	3	3	2	4	4	4
3	3	3	4	3	3	3	2	3	4	3	3	3	3
4	5	5	4	3	5	3	1	4	1	1	4	1	4
4	3	4	4	2	2	4	2	3	2	2	3	2	3
5	1	5	5	1	1	5	1	5	1	1	5	1	5
3	3	3	3	2	2	3	2	3	2	2	3	3	3
4	5	5	5	2	2	5	1	4	2	2	5	1	5
5	3	5	5	3	2	5	1	5	2	2	3	1	3
3	3	3	3	3	3	3	3	3	3	3	3	3	3
1	1	5	5	1	1	5	1	3	5	1	3	1	3
3	4	4	4	3	3	4	2	3	3	2	4	2	4
3	3	5	5	1	1	5	1	3	1	1	5	3	5
4	5	5	4	3	3	2	1	4	2	3	2	2	2
3	4	5	5	2	2	3	1	2	3	1	1	2	4
3	4	4	4	2	2	4	1	2	2	1	3	2	5
3	4	4	4	3	3	3	2	3	3	2	3	2	3
5	4	5	5	1	1	5	1	4	2	1	5	1	5
3	5	4	5	2	2	3	1	4	1	1	3	1	1
3	2	5	4	1	1	4	1	4	1	1	5	1	4
4	2	4	4	2	2	4	2	4	2	2	4	2	4
2	2	3	3	3	3	2	3	2	3	2	2	2	3
4	3	3	3	3	3	4	4	2	3	3	2	3	3
3	5	5	5	2	3	4	1	3	3	2	2	1	3
3	4	4	4	2	2	4	2	3	2	2	3	2	3
5	3	5	5	1	1	5	1	5	1	1	5	1	5
3	3	4	4	4	3	3	2	3	3	2	4	2	4
3	2	5	5	2	3	3	2	4	2	2	3	2	3
3	2	5	5	1	1	5	1	5	1	5	5	1	5
3	3	3	3	3	3	3	3	3	3	3	3	3	3
5	5	5	5	1	1	5	1	3	2	1	5	2	5
4	4	4	4	2	2	4	2	4	2	2	4	2	4

5	1	5	5	1	1	5	1	5	1	1	1	1	5
4	3	4	3	2	2	4	2	3	2	2	2	1	4
3	4	3	4	3	3	3	1	3	3	1	2	3	5
5	3	5	5	1	1	5	1	5	1	1	3	1	5
4	2	4	4	2	2	4	2	4	2	2	4	2	4
3	1	5	5	1	1	3	1	3	1	1	3	1	5
2	3	3	3	2	3	3	3	3	3	3	3	3	3
2	4	3	2	4	3	3	2	4	3	2	4	3	2
3	2	5	5	1	1	5	1	3	2	1	3	2	2
5	5	5	5	1	1	4	1	4	1	1	3	1	5
3	4	4	5	1	2	4	1	4	2	2	2	2	4
3	4	4	4	2	3	3	2	2	4	1	4	4	4
3	2	5	5	1	2	4	1	3	1	1	3	1	5
3	4	3	3	3	3	3	3	2	4	4	3	3	2
4	5	5	5	4	4	3	4	1	5	5	5	5	3
3	4	4	4	2	2	3	1	1	1	1	1	3	4
2	5	5	5	2	2	3	2	2	3	1	3	3	4
2	3	3	4	4	3	3	3	2	3	3	3	3	3
3	3	3	3	3	3	3	3	3	3	3	3	3	3
4	1	5	5	1	1	5	1	4	1	1	4	1	5
5	5	5	5	2	2	4	2	5	2	2	5	2	5
1	5	5	5	1	3	5	5	1	5	5	5	3	5
2	5	5	5	3	2	3	1	3	2	2	2	2	3
3	3	3	3	3	3	3	3	3	3	3	3	3	3
3	4	4	4	3	3	3	3	3	3	2	3	2	3
5	1	5	5	1	1	5	1	3	1	1	5	1	5
3	4	5	5	3	3	3	1	3	2	1	2	1	4
3	2	4	4	3	4	2	3	4	2	4	2	2	2
3	2	4	4	2	2	4	1	3	2	2	3	1	4
4	4	4	4	3	3	3	2	3	3	3	2	2	4
1	5	5	5	1	2	3	1	4	1	1	3	1	4
4	4	4	4	4	4	4	4	4	4	4	4	4	4
3	3	3	3	3	3	3	3	3	3	3	3	3	3
3	3	4	4	4	3	3	3	3	2	3	3	3	3
4	2	4	4	2	2	4	2	3	2	2	4	2	4
3	3	3	3	3	3	3	3	3	3	3	3	3	3
3	4	4	3	3	3	2	2	3	3	3	3	2	4
1	4	3	3	4	4	2	4	2	4	4	2	4	2
4	3	4	5	2	1	4	1	3	2	1	5	2	5
5	5	5	5	5	5	5	5	5	5	5	5	5	5
3	4	4	3	2	3	3	1	3	2	2	4	2	3
3	3	3	4	2	3	3	2	3	2	2	3	2	2
3	3	3	3	3	3	3	3	3	3	3	3	3	3
2	5	5	5	5	2	4	1	3	4	1	2	3	4
3	3	3	3	3	3	3	3	3	3	3	3	3	3
3	3	4	4	2	2	3	2	3	3	3	4	2	3
4	4	4	3	3	3	3	2	4	4	3	4	4	4
3	3	3	3	3	3	3	3	3	3	3	3	3	3
4	4	4	4	4	2	4	3	4	4	2	4	5	5
5	1	5	5	1	1	5	1	5	1	1	5	1	5
2	4	4	4	2	3	3	2	2	3	2	3	4	4
2	4	4	2	1	1	3	1	3	1	1	3	1	1
3	3	3	3	3	3	3	3	3	3	3	3	3	3
4	3	5	5	1	1	5	1	3	2	1	3	1	4
3	3	5	5	1	3	1	1	3	3	1	3	1	5
3	1	5	5	1	1	5	1	5	1	1	5	1	3

4	4	4	4	2	3	4	1	3	3	2	4	4	4
4	1	4	4	1	1	4	1	4	1	1	3	1	4
4	4	4	5	3	4	4	1	4	3	2	4	2	4
3	4	3	4	3	3	3	3	3	3	3	3	3	3
1	5	5	5	4	4	2	1	3	5	2	3	4	5
4	4	5	5	3	2	3	1	3	1	2	1	1	4
4	5	5	5	2	2	5	1	2	1	1	2	2	4
1	5	5	2	3	5	1	1	1	5	1	1	5	5
4	4	4	4	3	4	2	2	3	2	3	4	2	3
3	5	4	3	3	3	3	5	3	5	3	3	4	4
4	4	4	4	2	2	3	2	3	4	2	3	2	4
4	2	5	5	1	1	4	2	4	1	1	5	1	5
3	5	5	5	3	3	3	2	3	3	2	3	2	5
5	5	4	4	4	2	4	2	4	2	2	4	2	4
3	4	5	4	1	1	4	1	3	1	1	4	1	4
3	4	5	5	3	3	3	1	3	1	1	2	1	5
4	5	5	5	2	2	5	1	3	2	2	4	2	5
3	4	3	4	3	4	2	2	2	1	3	2	2	2
5	5	5	5	1	3	3	3	3	3	3	3	3	3
4	4	4	4	2	2	4	2	4	2	2	3	1	4
5	5	4	3	4	1	2	1	2	5	2	1	1	5
3	4	4	4	2	2	4	2	3	3	2	4	1	4
5	1	5	5	1	1	5	1	5	1	1	5	1	5
3	4	4	4	2	2	3	1	3	2	1	3	2	4
3	3	4	4	1	1	4	1	4	2	1	3	1	2
5	5	5	5	5	2	5	1	5	1	1	5	1	5
5	5	5	5	5	5	5	5	5	5	5	5	5	5
5	1	5	5	1	1	5	1	5	1	1	5	1	5
3	4	4	4	3	2	3	2	2	3	2	4	3	4
4	4	4	4	2	3	3	1	3	3	2	3	2	4
4	4	3	4	2	4	3	2	4	3	1	4	3	4
4	3	4	4	2	2	4	1	3	1	1	4	1	4
3	3	4	4	2	3	3	2	3	2	1	3	3	4
2	3	4	4	3	2	3	2	3	3	2	4	4	4
3	4	5	5	2	2	5	1	3	1	1	4	4	4
3	3	3	3	3	3	3	3	3	3	3	3	3	3
4	1	4	4	1	1	4	1	3	1	1	3	1	3
5	5	5	5	1	1	5	1	5	1	1	5	1	5
3	3	3	3	3	3	3	3	3	3	3	3	3	3
4	4	4	4	2	2	4	2	4	2	2	4	2	4
3	1	5	5	1	1	5	1	3	1	1	2	1	5
3	4	4	4	3	4	2	3	3	3	2	3	4	4
3	4	4	5	2	3	4	2	2	2	2	4	2	3
3	3	4	4	3	3	4	2	4	3	3	3	3	4
3	5	5	5	1	3	4	1	4	2	2	4	1	3
4	4	4	4	3	3	4	1	3	4	1	2	4	4
4	4	4	4	2	2	3	2	4	4	2	3	2	3
3	4	4	4	2	2	3	2	3	3	2	3	1	4
5	5	5	5	5	5	5	5	5	5	5	5	5	5
3	3	3	3	3	3	3	2	3	2	1	2	2	1
3	3	4	4	3	3	3	2	3	3	3	3	2	3
4	4	4	4	2	1	3	1	3	4	1	4	2	4
3	3	3	3	3	3	3	3	3	3	3	3	3	3
3	4	4	4	3	3	3	3	3	4	3	3	4	4
4	4	4	4	2	3	4	2	4	2	1	4	1	4
2	3	4	4	4	1	4	2	4	2	3	4	4	4

3	3	3	3	3	3	3	3	3	3	3	3	3	3
3	4	4	4	3	3	3	2	3	3	3	3	2	4
3	5	4	3	3	4	3	3	3	3	3	3	3	4
3	3	3	3	3	3	3	3	3	3	3	3	3	3
4	3	4	4	3	2	4	2	4	3	2	2	1	5
1	3	5	5	1	4	3	1	3	2	1	4	1	5
3	4	5	4	2	2	4	1	4	1	2	5	2	4
3	5	5	5	3	3	5	1	3	3	1	3	1	5
3	4	3	4	2	4	3	2	2	2	3	2	2	4
4	1	5	5	1	1	5	1	3	1	1	4	1	5
4	3	5	5	2	2	4	1	3	2	1	3	1	5
5	5	5	5	1	1	5	1	5	1	1	5	1	5
4	4	4	4	4	4	4	4	4	4	4	4	4	4
3	3	3	3	3	3	3	1	3	3	3	3	3	3
5	5	5	5	1	1	5	1	5	1	1	3	1	5
3	4	4	4	3	3	3	2	2	2	2	3	2	3
3	3	3	3	2	2	4	1	4	2	1	3	1	5
3	4	4	4	2	2	3	2	3	2	2	3	2	4
4	3	4	4	4	3	4	4	4	3	4	4	4	4
3	4	3	3	3	3	2	2	3	2	2	4	3	4
5	2	5	5	2	1	1	1	5	5	1	5	2	5
2	4	5	3	2	4	3	2	2	3	1	2	4	5
2	5	5	5	5	4	3	1	2	5	2	4	1	5
2	4	4	5	1	2	2	1	3	3	1	3	1	5
3	4	4	4	2	2	4	2	3	2	2	4	2	4
3	3	3	3	3	3	3	3	3	3	3	3	3	3
5	5	5	5	5	5	5	5	5	5	5	5	5	5
3	5	5	5	2	2	4	1	3	2	2	4	1	5
3	4	5	5	2	2	4	1	3	2	2	2	1	4
2	2	4	2	2	2	2	2	2	4	2	4	4	2
4	4	4	4	4	4	4	4	4	4	2	4	2	4
3	4	4	4	2	2	3	2	2	2	2	3	2	3
4	4	5	5	2	3	4	1	4	2	2	4	1	4
4	4	4	4	2	3	3	2	4	2	2	4	2	4
5	4	5	5	1	1	5	1	5	1	1	4	1	5
3	4	4	4	1	2	3	1	3	2	1	2	2	4
4	4	5	5	2	3	5	1	5	3	2	5	5	5
2	4	4	5	2	3	2	3	3	4	3	4	3	4
5	4	5	5	1	1	5	1	5	1	1	5	1	5
4	4	4	4	2	3	4	2	3	2	1	5	3	4
5	4	5	5	2	3	4	1	4	3	1	4	1	5
4	2	4	4	2	2	4	1	3	2	2	4	1	4
5	4	5	5	3	5	3	3	3	4	3	5	5	5
4	4	4	4	4	4	4	4	4	4	4	4	4	4
2	4	5	5	2	2	3	1	3	1	2	4	3	5
4	5	4	4	2	2	4	2	4	2	2	2	1	3
4	3	4	4	2	2	4	1	4	1	1	4	1	5
1	5	3	3	3	4	2	3	1	5	3	3	4	3
3	3	3	3	3	3	3	3	3	3	3	3	3	3
4	3	4	3	4	4	3	4	4	3	4	4	3	4
3	3	3	3	3	3	3	3	3	3	3	3	3	3
3	3	3	3	3	3	3	3	3	3	3	3	3	3
2	4	3	3	2	2	2	1	2	3	1	2	2	3
3	3	3	3	3	3	4	2	3	2	3	3	3	3
5	1	5	5	1	1	5	1	5	1	1	1	1	5
2	4	3	4	4	2	4	3	4	5	4	5	4	4

4	3	4	4	2	1	4	1	4	1	1	4	1	4
3	4	4	4	4	3	2	2	2	2	2	4	3	4
2	2	3	3	3	3	3	3	3	3	2	2	2	2
3	4	5	5	1	1	5	1	3	2	1	3	1	4
4	4	4	4	2	2	4	1	4	1	1	4	1	4
3	3	3	3	3	3	3	3	3	3	3	3	3	3
4	5	5	5	2	2	4	2	4	2	1	4	1	4
4	3	4	5	3	3	4	1	2	3	1	4	2	5
2	2	4	4	2	1	4	3	2	3	1	3	1	3
3	5	5	5	2	1	3	1	3	3	1	2	3	4
4	2	4	4	1	2	4	1	3	1	1	4	1	4
4	4	4	4	2	2	3	2	2	2	2	3	2	3
3	4	4	4	2	2	3	2	3	4	2	3	2	4
5	1	5	5	1	1	5	1	5	1	1	5	1	5
3	3	3	3	3	3	3	3	3	3	3	3	3	3
2	3	4	4	2	2	3	1	2	2	2	3	2	3
3	4	4	4	2	3	3	1	2	2	2	3	2	4
2	3	4	4	2	2	4	1	3	3	2	2	2	2
2	4	4	3	2	2	4	2	3	4	2	4	1	4
4	4	4	4	2	2	4	2	3	3	3	3	3	3
5	5	5	5	5	5	5	5	5	5	5	5	5	5
3	3	5	5	2	1	5	1	3	2	1	4	1	4
3	3	3	3	3	3	3	3	3	3	3	3	3	3
4	1	5	5	1	2	4	1	3	1	1	2	3	5
3	3	3	3	3	3	3	3	3	3	3	3	3	3
5	3	5	5	1	1	5	1	3	1	1	5	1	5
4	3	4	5	1	1	3	1	3	1	1	4	1	5
2	2	4	4	2	3	2	2	3	3	2	3	2	4
5	2	5	5	1	1	5	1	5	1	1	5	1	5
2	3	4	4	3	3	2	1	3	2	1	3	2	4
5	1	5	5	1	1	5	1	4	1	5	5	1	5
2	4	2	4	4	4	2	2	3	2	4	1	2	1
3	3	3	4	2	2	2	1	2	2	1	3	2	3
3	4	3	2	2	3	3	3	3	4	3	3	4	4
2	4	3	4	4	4	2	3	2	2	3	2	3	3
2	5	5	5	2	2	3	2	2	3	3	3	3	4
3	4	4	4	4	4	3	3	3	4	3	3	3	3
1	5	5	5	1	5	1	1	1	1	1	5	1	5
4	3	4	4	2	2	4	2	3	2	2	4	3	4
4	4	4	4	4	4	4	4	4	4	4	3	4	4
4	3	5	5	1	1	3	1	3	1	1	3	1	5
4	3	4	4	2	2	4	3	3	3	3	3	3	4
4	4	4	4	4	4	3	2	3	3	3	2	2	4
4	4	4	4	2	1	4	1	4	1	1	4	1	4
3	5	3	2	5	4	2	5	2	5	5	2	5	2
3	4	4	4	3	3	2	2	2	3	2	2	2	4
3	2	5	5	1	1	5	1	5	1	1	5	1	5
3	4	4	4	3	3	3	2	3	3	2	3	3	3
4	5	5	5	1	1	5	1	5	1	1	1	1	5
5	5	5	5	5	5	5	5	5	5	5	5	5	5
2	3	3	3	3	2	3	2	4	4	2	1	2	4
3	2	4	4	2	3	3	1	3	2	1	3	2	4
3	3	3	3	3	3	3	3	3	3	3	3	3	3
3	3	4	4	1	1	4	1	3	1	1	2	1	4
3	3	3	3	3	2	3	3	3	4	2	3	2	4
3	2	4	4	3	2	3	3	3	3	3	3	3	3

2	4	4	4	3	3	4	2	2	4	3	3	4	3
5	2	5	5	1	1	5	1	3	1	1	3	1	5
3	5	5	5	2	2	4	1	3	1	1	4	1	5
1	2	4	5	2	1	4	1	3	3	1	5	1	2
4	4	4	4	3	3	3	3	3	3	2	3	3	3
4	4	4	4	2	2	3	2	4	3	2	3	2	4
3	4	4	5	4	3	2	2	3	3	2	3	2	3
3	3	4	4	3	2	3	2	3	2	3	3	2	3
3	3	3	3	3	3	3	3	3	3	3	3	3	3
5	5	5	5	2	2	5	1	4	2	2	4	4	5
5	2	5	5	1	2	5	1	4	1	1	4	1	5
4	1	5	4	1	2	3	2	2	1	2	4	2	3
3	3	4	4	2	3	3	3	3	3	2	3	3	4
3	4	4	4	3	3	3	3	3	4	3	2	3	3
3	4	4	3	1	2	3	1	4	1	1	3	1	4
4	3	3	3	3	3	2	3	3	3	3	3	2	4
3	3	4	4	2	3	3	1	1	3	2	3	2	4
5	5	5	5	2	2	5	3	5	5	3	5	2	5
4	3	4	4	2	3	4	2	3	3	2	4	2	4
3	3	3	3	3	3	3	3	3	3	3	3	3	3
3	3	4	4	2	2	4	2	3	2	2	3	2	4
3	3	3	3	3	3	3	3	3	3	3	3	3	3
4	3	5	5	1	1	5	1	4	1	1	1	1	5
5	1	5	5	1	1	5	1	5	1	1	5	1	5
4	4	4	4	4	4	4	4	2	4	2	4	4	4
3	2	4	4	2	3	3	1	3	5	1	4	1	4
3	4	4	3	2	3	3	2	3	3	2	3	2	2
2	3	4	4	2	3	3	2	2	3	2	3	2	3
4	3	5	5	1	1	5	1	5	1	1	5	1	5
3	3	3	3	3	2	3	2	3	2	2	3	2	3
4	4	4	4	3	2	3	1	3	2	1	3	3	4
4	5	5	5	2	3	3	2	3	4	1	3	2	4
3	5	5	5	5	3	3	3	2	2	2	2	4	3
2	2	3	3	1	1	3	1	1	1	1	3	1	3
5	1	5	5	1	1	5	1	5	1	1	5	1	5
4	4	4	4	4	3	4	1	3	3	3	4	3	4
4	4	4	3	3	3	3	2	4	2	3	3	1	4
3	2	3	3	2	3	2	2	3	2	2	3	2	3
5	1	5	5	1	1	5	1	5	1	1	3	1	5
2	3	4	3	2	2	3	1	3	2	2	4	1	4
5	5	4	4	3	4	3	2	3	3	3	3	2	4
3	3	3	3	3	3	3	3	3	3	3	3	3	3
3	4	5	5	1	4	3	3	3	1	1	4	1	5
4	5	4	4	4	2	4	3	4	4	2	1	1	5
2	2	5	5	2	2	2	2	2	2	2	3	3	3
4	4	3	4	4	4	2	3	4	4	3	3	2	4
4	4	4	4	2	2	4	2	4	2	2	2	3	4
4	4	3	4	4	3	3	3	3	4	3	3	2	4
4	4	4	4	5	4	5	4	5	4	3	3	3	3
3	4	4	4	4	2	4	3	3	3	2	2	2	4
3	3	3	3	3	4	4	3	3	3	2	5	2	2
5	5	5	5	5	5	5	5	5	5	5	5	5	5
3	4	3	3	3	3	3	2	3	3	2	3	2	3
3	3	3	3	3	3	3	3	3	3	3	3	3	3
4	3	4	4	2	2	4	2	3	2	2	4	3	4
1	5	5	5	1	5	1	1	1	1	1	5	1	4

4	4	4	4	1	1	4	2	4	2	2	4	4	4
5	5	5	5	1	5	5	5	5	5	5	5	5	5
1	2	5	5	3	2	4	1	2	2	1	2	2	1
3	3	3	3	3	3	3	3	3	3	3	3	3	3
3	3	3	3	3	3	3	3	3	3	3	3	3	3
2	4	4	4	2	3	4	2	3	3	2	3	3	4
3	4	4	4	4	3	3	2	3	4	3	3	3	3
4	4	4	4	4	4	2	2	3	3	2	3	3	4
4	4	3	3	2	2	4	2	4	2	2	3	2	3
3	4	4	4	3	2	3	1	3	4	2	2	2	4
5	4	5	5	2	2	5	2	3	3	1	5	2	5
3	3	4	4	2	2	3	2	3	2	2	4	2	4
5	1	5	5	1	1	5	1	5	1	1	5	1	5
5	1	5	5	1	1	5	1	5	1	1	3	1	5
4	4	4	4	2	3	4	2	4	2	2	4	2	4
3	3	3	3	3	4	2	2	2	2	2	3	2	4
3	3	3	3	3	3	3	3	3	3	3	3	3	3
3	3	5	5	2	2	3	1	3	2	1	3	1	4
3	3	4	4	2	2	4	1	3	2	1	3	1	3
5	5	5	2	2	1	4	1	3	1	1	3	1	3
5	1	5	5	1	1	5	1	5	1	1	5	1	5
1	5	3	3	3	2	2	1	3	2	1	2	3	3
4	2	4	4	3	3	3	3	3	3	3	3	3	3
3	3	3	3	3	3	3	3	3	3	3	3	3	3
3	4	4	4	2	2	4	2	3	2	2	3	2	4
3	3	3	3	3	3	3	3	3	3	3	3	3	3
3	3	3	3	3	3	3	3	3	3	1	3	1	5
3	2	4	4	1	1	4	1	3	1	1	4	1	5
2	1	5	5	1	1	5	1	3	1	1	5	1	5
1	5	1	1	5	5	1	5	1	5	5	1	4	4
3	3	5	5	1	1	5	1	3	1	1	5	1	5
5	3	5	5	1	1	5	1	5	3	1	5	1	5
4	3	4	5	2	2	5	1	3	2	2	3	2	3
5	2	5	5	2	2	4	1	3	2	2	5	2	5
4	4	5	5	4	1	4	3	4	1	1	2	1	4
3	3	4	4	2	2	3	1	3	2	2	3	1	3
3	3	3	3	3	3	3	3	3	3	3	3	3	3
3	5	5	5	3	5	2	3	2	5	2	4	5	3
3	2	4	3	2	3	4	3	3	4	3	2	3	3
4	4	3	4	3	4	3	4	4	4	3	4	4	4
3	3	4	3	3	3	3	2	3	3	1	3	3	4
3	3	3	3	3	3	3	3	3	3	3	3	3	3
5	5	5	5	1	2	4	3	4	2	1	4	1	5
3	3	4	4	2	2	3	2	3	3	2	4	2	3
5	5	5	5	1	1	5	1	3	1	1	5	1	5
3	3	4	3	2	2	3	1	3	3	3	2	2	4
3	4	4	4	2	1	4	1	3	3	1	3	2	4
5	5	5	5	5	5	5	5	5	5	5	5	5	5
3	3	3	3	3	3	3	3	3	3	3	3	3	3
4	4	3	4	4	3	4	3	4	3	4	3	3	3
2	5	5	5	5	5	2	3	2	5	3	2	3	4
3	1	5	5	1	1	3	1	3	1	1	5	1	5
3	4	4	4	3	3	3	2	3	3	2	2	2	4
2	4	4	4	2	3	3	2	2	3	2	2	2	4
2	2	3	3	3	2	3	2	2	3	2	2	3	3
3	3	4	4	2	2	4	2	4	3	2	3	2	3

3	5	5	5	2	2	4	2	3	2	1	2	2	3
3	4	4	4	2	3	3	2	3	2	2	4	2	4
3	2	4	2	4	4	2	4	2	4	4	2	4	2
5	5	5	5	5	5	5	5	5	5	5	5	1	5
2	4	4	3	2	3	2	3	2	4	2	3	3	2
5	3	5	5	1	2	4	1	4	1	1	3	1	3
3	3	3	3	3	3	3	3	3	3	3	3	3	3
2	5	4	4	2	3	3	1	3	4	2	3	2	3
3	3	5	5	1	1	5	1	3	1	1	5	1	3
1	4	5	5	2	2	3	1	1	1	1	2	1	1
3	4	5	5	2	1	3	1	3	4	1	5	1	5
3	4	5	5	3	2	3	1	3	3	1	4	3	4
3	3	3	3	3	3	3	3	3	3	3	3	3	3
3	5	5	5	3	1	5	1	1	4	1	1	3	3
4	2	5	4	1	1	5	1	4	1	1	4	1	4
5	2	5	5	3	3	4	1	4	2	2	2	2	5
3	3	3	3	3	2	3	2	2	4	3	3	3	3
4	2	4	3	3	3	3	3	2	3	2	3	2	3
5	3	5	5	1	1	5	1	4	1	1	5	1	5
3	2	4	4	2	1	4	1	4	1	2	4	1	3
3	4	4	3	3	4	3	3	3	4	3	3	3	4
2	2	4	5	1	1	4	1	3	1	1	4	2	4
4	2	5	5	1	2	5	1	3	1	1	5	1	5
3	3	3	3	3	3	3	3	3	3	3	3	3	3
1	2	3	3	3	2	3	1	3	2	2	2	2	4
5	2	5	5	1	1	5	1	5	1	1	5	1	5
4	4	4	4	3	4	3	3	3	4	3	3	3	4
3	3	4	5	2	3	4	2	2	3	2	3	2	4
1	4	5	5	1	1	4	1	3	1	1	3	1	4
3	4	4	4	3	2	3	2	3	3	2	4	4	4
2	4	4	4	4	4	4	2	3	3	2	3	3	4
3	2	4	4	1	1	3	1	3	4	2	3	2	4
5	5	5	5	1	1	5	1	5	1	1	5	1	5
5	5	5	5	3	1	4	1	3	3	3	4	1	5
3	3	4	4	3	2	4	2	3	3	2	3	3	3
3	3	3	3	3	3	3	3	3	3	3	3	3	3
2	5	5	5	1	1	5	1	3	1	1	3	1	3
3	3	4	4	3	3	4	1	3	2	2	2	2	2
5	4	5	5	2	3	5	1	4	2	1	4	1	4
4	3	4	4	1	1	4	1	3	3	1	3	2	4
4	4	4	4	2	2	4	1	3	2	2	2	2	4
3	4	3	4	3	4	3	3	4	4	3	4	4	4
3	4	4	4	2	3	3	2	3	4	2	3	1	4
3	3	3	3	3	3	3	3	3	3	3	3	3	3
3	3	5	5	1	1	5	1	3	3	1	5	1	5
3	4	4	4	3	4	4	2	3	3	2	3	2	3
2	4	4	4	4	4	3	3	3	3	3	2	3	4
3	4	4	3	2	2	3	2	3	2	2	3	2	4
4	4	4	4	2	3	4	2	3	3	3	3	2	4

a77	a78	a79	a80	a81	a82	a83	a84	a85	a86	a87	a88
1	1	5	1	5	1	5	1	1	5	5	1
3	1	4	2	4	1	1	1	5	4	2	1
1	1	4	1	1	1	1	1	1	1	1	1
1	1	3	2	1	1	1	1	1	1	1	1
2	2	4	2	4	2	3	2	4	3	4	2
2	4	2	4	2	4	4	2	4	4	2	4
3	2	2	3	2	1	1	1	1	1	1	1
1	1	4	1	4	2	4	2	2	2	4	3
3	3	3	3	3	3	3	3	3	3	3	3
3	2	4	4	4	2	4	2	4	3	4	4
1	1	3	3	2	1	1	1	1	1	1	1
3	3	3	3	3	3	3	3	3	3	3	3
3	2	2	2	2	2	2	2	2	2	2	3
3	3	4	3	3	3	3	2	2	3	3	3
2	2	4	2	2	2	2	2	2	2	2	2
2	2	4	2	2	2	2	2	2	2	1	3
3	3	3	3	3	3	3	2	3	2	3	3
4	1	4	2	4	1	4	1	3	2	4	1
2	2	4	2	4	1	2	1	3	1	2	2
3	2	3	4	5	2	3	2	3	4	5	3
3	2	2	3	4	4	3	2	2	4	4	3
5	5	5	5	5	1	1	1	5	5	5	1
3	3	3	3	3	3	3	3	3	3	3	3
1	1	5	1	2	1	1	1	1	1	1	1
2	3	3	3	2	2	2	2	2	4	2	2
2	1	5	2	4	1	2	1	2	1	3	2
1	2	4	1	2	2	1	2	2	2	2	1
2	2	4	3	4	2	4	2	3	1	3	2
1	1	4	1	1	1	2	1	1	1	2	1
1	1	3	3	3	1	1	1	2	2	4	1
3	1	4	2	2	2	2	1	2	1	1	1
2	1	5	3	3	1	2	1	5	1	3	3
4	2	4	2	4	2	2	2	2	2	4	2
5	3	3	3	5	3	3	3	3	3	3	3
1	1	5	1	2	1	4	1	4	1	5	1
3	1	4	3	5	1	5	1	2	3	4	2
2	2	4	2	3	2	2	2	2	2	2	2
3	3	3	3	3	3	3	3	3	3	3	3
2	2	4	2	4	2	3	1	3	2	3	2
3	3	3	3	3	3	3	3	3	3	3	3
3	3	4	3	3	2	3	3	3	3	4	3
3	3	3	3	3	3	3	3	3	3	3	3
4	2	4	3	4	3	4	3	4	3	4	2
1	1	3	1	5	1	5	1	1	1	5	3
2	1	4	3	3	1	4	1	2	2	3	3
1	3	4	1	1	1	1	1	1	3	2	2
4	2	2	4	4	2	4	2	2	2	4	4
4	4	4	4	4	4	4	4	4	4	4	4
1	1	4	3	3	1	1	1	1	1	2	3
1	1	4	1	1	1	2	1	1	1	1	1

1	1	5	1	1	1	1	1	1	1	1	1
3	3	3	3	2	2	4	3	4	3	3	2
2	2	2	2	3	2	3	2	2	2	3	2
2	2	4	1	3	2	2	2	2	2	2	2
2	1	3	1	2	1	2	1	1	1	2	1
2	2	3	3	4	2	3	2	2	2	4	3
3	1	2	1	4	1	4	1	3	4	4	2
4	2	4	3	4	2	3	2	4	4	4	3
3	2	3	2	2	1	3	2	2	2	3	2
2	1	4	2	2	2	2	2	2	2	2	1
3	3	3	3	3	3	2	2	3	4	2	2
1	1	5	1	1	1	1	1	1	1	5	1
3	2	4	2	4	1	2	1	1	1	2	2
3	1	3	3	1	1	1	1	1	1	1	3
3	2	4	3	5	1	3	1	3	3	3	3
3	1	3	3	3	1	2	1	2	2	2	4
4	2	4	2	3	2	3	1	3	2	3	2
1	1	4	1	1	1	1	1	1	2	2	1
3	2	3	3	4	2	3	1	2	2	1	3
2	1	4	2	2	1	1	1	3	1	4	1
2	2	3	3	3	2	2	2	2	2	3	3
3	1	2	1	3	1	1	1	1	1	4	1
1	1	5	1	1	1	1	1	1	1	1	1
3	1	3	1	3	1	1	1	1	1	1	1
2	2	3	2	2	2	2	2	2	2	2	2
3	1	1	2	5	2	3	2	4	3	4	3
1	1	3	2	3	1	1	1	1	1	2	1
1	1	4	2	1	1	1	1	1	1	1	1
4	1	3	1	1	1	1	1	3	1	1	1
2	1	3	2	2	1	2	1	1	1	1	2
3	3	4	3	4	3	3	2	3	3	4	2
2	1	4	2	4	1	1	1	2	1	2	2
3	3	4	3	4	3	4	2	2	3	4	3
2	2	3	3	3	2	3	2	3	2	4	3
1	1	4	1	2	1	1	1	1	1	1	1
3	3	3	3	3	3	3	3	3	3	3	3
3	3	3	3	3	3	3	2	1	2	5	3
2	2	4	3	3	2	2	2	2	3	2	2
3	2	4	3	4	2	3	2	3	3	3	3
1	1	4	1	1	1	1	1	1	1	1	1
4	2	3	2	4	2	4	2	4	3	3	3
2	1	4	2	3	1	1	1	1	1	3	1
2	2	4	2	4	2	4	1	4	4	4	2
2	1	3	2	3	1	1	1	3	1	5	1
1	1	5	1	1	1	1	1	1	1	1	1
3	1	3	2	3	1	2	1	2	1	2	1
3	1	4	2	3	1	3	1	2	1	3	3
4	3	3	4	3	3	3	3	3	3	3	3
3	3	2	2	5	2	4	1	4	4	4	4
2	2	3	2	2	2	2	2	2	2	2	2
3	1	4	3	2	1	2	1	2	1	2	3
2	1	4	3	2	1	2	1	1	1	3	2
3	2	4	2	4	2	2	2	2	4	4	2
2	1	2	1	4	1	3	1	2	1	2	2
2	2	4	2	2	2	2	2	2	2	2	2
1	1	5	1	3	1	1	1	1	1	2	1

1	1	3	2	1	1	1	1	2	1	1	1
2	3	3	2	3	3	3	3	3	3	2	2
3	2	4	3	4	2	2	1	4	2	2	2
1	1	5	2	3	1	2	1	1	1	2	1
3	4	3	3	4	3	4	3	3	3	3	3
3	3	3	3	3	3	3	3	3	3	3	3
1	1	4	1	1	1	1	1	1	1	1	1
2	2	4	1	4	1	2	1	4	2	4	1
2	4	3	3	4	3	4	4	3	2	4	3
2	3	4	2	3	2	2	2	2	2	2	2
1	1	2	1	3	2	1	1	1	1	4	1
2	2	4	2	4	2	3	2	2	2	4	2
3	2	3	3	4	2	3	1	2	4	3	2
4	1	3	4	4	1	2	1	2	1	4	4
3	3	3	4	5	3	5	3	3	3	5	5
3	2	2	2	2	1	3	2	4	4	4	3
5	5	5	5	5	5	5	5	5	5	5	5
3	1	3	2	4	1	3	1	4	4	4	3
1	1	5	1	1	1	1	1	1	1	1	1
3	3	3	3	5	3	5	3	5	3	5	5
3	2	3	3	5	2	3	2	3	4	4	2
4	3	4	3	3	3	3	2	3	3	3	3
2	1	3	1	4	1	1	1	2	1	3	1
3	2	3	2	4	2	2	2	3	3	4	3
2	3	4	3	2	2	3	1	3	2	2	2
2	2	2	2	2	2	2	2	3	3	3	2
1	1	4	1	1	1	1	1	1	1	1	1
3	2	4	3	4	2	4	1	4	2	4	4
5	1	3	5	5	1	5	5	5	5	1	5
3	3	5	2	2	3	1	3	4	4	4	1
3	3	3	3	3	3	3	3	3	3	3	3
3	3	2	4	4	3	4	3	4	3	5	4
2	2	3	2	2	2	2	2	2	1	2	3
2	2	5	1	1	1	1	1	5	5	1	1
2	2	3	2	2	2	2	2	2	2	3	2
2	2	4	2	4	2	2	2	3	2	4	2
1	1	3	1	1	1	1	1	3	1	1	1
3	2	4	3	3	2	4	2	3	5	4	3
3	3	3	3	3	3	3	3	3	3	3	3
3	1	1	1	5	1	5	1	1	3	5	1
3	3	3	3	3	3	3	2	3	4	4	3
2	1	2	4	4	1	4	1	3	2	4	3
3	3	3	3	3	3	3	3	3	3	3	3
2	2	4	2	2	1	2	1	2	2	2	2
2	3	4	2	3	2	2	2	2	2	2	1
3	1	4	1	2	1	1	1	1	2	4	2
3	1	3	3	4	1	1	1	2	2	2	2
3	1	3	2	3	2	3	1	3	3	3	2
3	2	4	2	3	2	2	2	2	2	2	2
5	5	5	5	5	5	5	5	5	5	5	5
1	1	5	1	3	1	1	1	1	1	1	1
2	1	4	2	2	1	2	1	1	1	3	1
3	3	3	3	3	3	3	3	3	3	3	3
2	1	4	1	1	1	1	1	4	1	1	1
3	3	3	3	4	4	4	2	4	2	4	3
2	2	4	4	2	3	3	3	3	3	3	3

2	2	4	2	2	1	2	1	2	2	2	1
2	1	4	1	5	2	3	2	3	3	5	1
3	3	4	2	3	2	4	2	3	2	4	3
3	2	3	3	3	2	3	2	2	3	3	3
2	2	5	2	5	2	2	2	2	3	5	2
1	1	5	3	5	3	1	2	1	3	5	1
1	1	4	2	4	1	1	1	1	2	4	1
3	3	3	3	3	3	3	3	3	3	3	3
4	3	3	2	4	3	3	3	4	3	4	3
3	2	4	4	4	2	4	1	1	1	4	4
3	1	3	2	3	1	1	1	1	1	2	1
2	1	4	3	4	1	3	1	1	1	4	1
1	1	3	1	3	2	3	1	1	1	3	1
4	2	2	2	4	1	3	1	3	3	5	2
2	1	3	2	2	1	1	1	2	1	1	2
3	3	3	3	3	3	3	3	3	3	3	3
1	1	5	1	1	1	1	1	1	5	5	1
3	3	3	3	3	3	3	3	3	3	3	3
2	1	4	2	2	2	2	2	2	2	4	2
5	1	2	5	5	1	5	1	4	3	5	5
3	3	3	3	3	3	3	3	3	3	3	3
4	1	5	3	5	1	5	1	2	3	4	2
3	1	3	2	4	2	4	1	4	2	2	2
3	3	3	3	3	3	3	3	3	3	3	3
4	3	3	4	4	3	4	3	3	3	4	4
3	2	2	3	4	3	4	2	2	2	4	3
1	1	5	1	4	1	4	1	3	1	3	1
4	3	4	4	3	2	4	2	4	3	3	2
3	3	3	3	3	3	3	3	3	3	3	3
1	1	2	4	4	2	2	2	2	3	4	3
2	2	2	2	4	2	2	2	3	2	4	2
2	1	5	2	5	1	3	1	5	4	5	2
1	1	3	1	1	1	1	1	1	1	1	1
1	1	4	2	1	1	3	1	1	1	1	1
2	1	2	1	3	1	1	1	1	1	1	1
2	2	4	2	2	2	2	1	2	1	4	2
2	2	3	2	4	2	2	2	2	2	2	2
3	1	4	2	4	1	1	1	3	4	2	2
2	2	3	2	5	2	3	2	3	2	4	2
3	2	4	2	2	2	2	2	2	2	3	2
3	3	3	3	3	3	3	3	3	3	3	3
2	2	5	3	1	1	1	1	1	1	1	1
5	2	4	3	5	3	5	3	5	4	5	4
2	1	3	3	4	1	4	1	2	1	4	3
3	2	4	3	4	2	3	2	3	2	4	3
4	2	4	4	4	2	3	2	4	4	4	4
2	2	3	2	2	2	2	2	2	2	2	2
3	2	3	4	4	2	4	2	2	2	4	4
2	1	3	2	2	1	1	1	1	1	2	2
3	2	4	2	3	2	2	2	2	2	3	2
4	1	4	4	4	2	4	1	4	3	4	4
1	1	5	1	1	1	3	1	1	1	1	1
3	2	4	3	3	2	3	2	2	2	4	3
3	2	4	3	4	2	3	2	3	2	4	3
2	2	4	3	3	2	2	2	2	2	2	2
2	2	4	2	4	1	3	3	2	2	2	2

3	1	2	3	4	2	3	2	2	2	4	4
3	3	3	3	3	3	3	3	3	3	3	3
1	1	4	1	1	1	1	1	1	1	2	1
3	2	4	4	4	3	5	2	3	3	4	4
3	3	3	3	3	3	3	3	3	3	3	3
1	1	5	1	1	1	1	1	1	1	1	1
2	2	4	2	4	2	3	2	2	2	4	2
4	1	2	2	5	1	3	1	3	4	4	2
1	1	4	2	3	1	2	1	1	1	2	1
2	2	2	2	2	2	2	2	2	2	2	2
4	1	4	2	4	1	2	1	2	2	4	2
3	1	4	2	2	1	3	1	2	2	4	2
1	1	3	1	3	1	4	1	2	1	2	1
2	1	3	2	4	1	5	1	1	3	5	2
3	2	4	3	3	1	2	1	2	1	1	2
1	1	4	1	1	1	1	1	3	1	1	1
1	1	4	1	1	1	1	1	1	5	5	1
3	3	3	3	3	3	3	3	3	3	3	3
4	4	3	4	3	3	4	3	3	2	3	3
1	1	4	3	3	1	1	1	1	1	1	1
2	1	4	2	3	1	1	1	2	1	3	2
4	1	1	5	5	1	5	1	2	1	5	5
2	2	4	2	2	1	2	1	2	4	2	1
2	1	3	2	3	1	4	1	1	1	3	3
2	1	4	2	4	1	4	2	1	1	4	4
3	2	4	2	3	2	4	2	2	2	4	2
1	1	5	2	5	1	1	1	1	1	5	1
3	2	4	2	3	2	4	2	2	2	4	2
2	2	4	2	3	2	2	2	2	2	2	2
3	1	3	2	3	1	1	1	3	3	2	1
3	2	4	4	5	2	4	1	4	2	5	4
4	2	4	2	4	2	4	2	4	3	4	2
1	1	4	1	2	1	1	1	3	1	1	1
2	1	2	2	4	2	3	1	3	2	3	2
1	1	4	2	4	1	3	1	1	1	3	1
1	1	5	3	5	1	5	1	3	3	3	3
1	3	4	2	5	2	4	2	2	2	4	3
1	1	5	1	1	1	1	1	1	1	1	1
1	1	2	3	3	1	3	1	4	1	3	4
1	1	4	1	3	1	1	1	1	1	1	1
2	2	4	3	4	2	3	2	3	2	4	2
3	1	2	3	5	1	3	1	3	3	3	3
5	1	5	1	5	1	5	1	1	1	5	1
2	2	2	2	2	2	2	2	2	2	3	2
2	1	3	3	3	1	3	1	4	4	4	1
1	1	5	4	5	1	2	1	1	1	4	1
1	4	4	1	5	1	3	1	5	5	5	3
3	2	3	2	4	3	4	2	4	3	4	3
3	2	2	3	3	2	3	2	3	2	3	3
2	2	3	2	5	2	5	3	3	2	3	2
4	2	4	2	5	2	5	2	1	5	5	4
2	2	4	2	2	2	2	2	2	2	2	2
1	1	4	3	3	1	3	1	5	1	3	3
2	1	3	1	4	1	4	1	1	2	4	1
2	2	4	2	4	2	3	2	4	2	4	2
2	1	2	1	2	1	2	1	2	1	3	2

1	1	5	3	4	1	1	1	1	1	1	2
3	2	3	2	3	1	2	1	1	1	3	3
2	2	3	2	2	2	1	1	1	1	1	1
3	1	3	3	4	1	2	1	1	1	4	3
1	1	5	1	1	1	1	1	1	1	1	1
2	2	4	2	2	2	2	2	2	2	2	2
3	3	3	3	3	3	3	3	3	3	3	3
4	1	1	4	3	4	2	3	3	4	2	4
4	3	3	3	3	3	4	3	3	3	4	3
1	1	5	1	2	1	1	1	2	1	4	1
3	3	3	3	3	3	3	3	3	3	3	3
1	1	4	1	1	1	1	1	1	1	2	1
2	2	2	3	5	1	5	1	3	1	5	3
2	1	5	1	1	1	1	1	1	1	5	1
4	4	4	4	4	4	5	4	4	5	4	4
5	5	5	5	5	5	5	5	5	5	5	5
4	1	2	5	5	1	4	1	5	1	4	4
3	3	3	3	3	3	3	3	3	3	3	3
3	3	3	3	3	3	3	3	3	3	3	3
2	1	3	3	4	1	4	1	2	1	3	2
2	2	3	2	5	1	2	2	2	2	5	1
3	3	3	3	3	3	3	3	3	3	3	3
3	2	3	2	4	2	2	2	3	2	2	2
5	1	1	1	5	2	5	1	5	4	4	5
2	2	4	1	2	2	2	2	2	2	3	1
4	2	4	4	2	2	2	2	2	2	2	2
2	2	4	3	3	2	2	2	3	2	3	2
3	3	3	3	4	3	4	2	4	3	4	4
2	2	4	2	2	2	2	2	2	3	4	2
1	1	4	1	2	1	1	1	1	1	1	1
1	1	4	1	1	1	1	1	1	1	1	1
3	2	3	3	4	2	3	2	2	2	2	2
1	1	5	1	1	1	1	1	1	1	1	1
3	3	3	4	2	3	3	3	3	3	3	3
1	1	5	1	4	1	5	1	1	1	4	2
1	1	1	1	4	1	1	1	1	1	1	1
1	1	4	1	1	1	1	1	1	1	1	1
4	2	4	3	4	2	2	1	2	2	4	1
1	1	3	3	3	1	1	1	1	1	1	1
1	1	3	3	3	1	1	1	1	1	1	1
2	1	4	2	2	1	2	1	1	1	1	1
1	1	5	1	1	1	1	1	1	1	1	1
4	2	2	4	4	1	4	1	3	4	4	4
1	5	5	2	1	1	1	1	1	1	1	1
3	1	4	3	2	1	2	1	2	1	4	3
1	1	3	1	1	1	1	1	1	1	1	1
5	5	5	5	5	5	5	5	5	5	5	5
1	1	2	1	2	1	1	1	1	1	2	1
3	1	3	3	4	1	3	1	3	3	5	5
2	1	4	1	3	1	2	1	1	1	2	1
3	3	3	3	3	2	3	2	3	2	4	3
1	5	2	3	1	5	1	5	1	5	1	1
2	1	4	2	4	2	3	2	2	3	2	2
3	1	5	3	3	1	1	1	1	1	1	1
3	3	3	3	3	3	3	3	3	3	3	3
1	1	4	1	3	1	1	1	1	1	3	1

5	5	5	5	5	5	5	5	5	5	5	5
3	2	3	4	3	2	3	2	4	2	3	3
3	4	3	3	4	3	3	3	3	3	3	3
1	1	4	1	4	1	3	1	3	1	3	1
3	2	4	2	2	2	2	2	2	2	2	2
3	2	3	3	4	3	4	3	3	3	4	3
2	2	3	2	2	2	2	2	3	2	2	2
3	3	3	3	3	3	3	3	3	3	3	3
2	1	3	3	3	1	3	1	2	2	3	2
3	3	3	3	3	3	3	3	3	3	3	3
2	2	3	2	4	2	2	2	3	2	3	3
1	1	5	1	1	1	1	1	1	1	1	1
4	2	3	2	4	2	2	2	2	2	2	2
4	4	4	4	4	4	4	4	4	4	4	4
1	1	4	1	1	1	1	1	1	1	1	1
2	2	4	3	4	2	3	1	1	1	3	1
1	1	5	1	1	1	1	1	1	1	1	1
2	1	4	2	5	1	1	1	1	4	4	2
3	2	3	3	3	2	2	2	3	2	2	3
2	1	5	2	4	1	2	1	2	3	1	1
4	2	5	2	4	1	4	1	4	2	5	4
2	2	4	3	4	2	2	2	2	2	4	3
2	2	3	2	2	2	2	2	2	2	2	2
3	2	4	3	4	2	2	2	3	2	2	2
3	3	3	3	3	3	3	3	3	3	3	3
3	1	4	2	2	2	2	2	2	2	2	2
3	1	2	2	3	1	1	1	1	1	1	1
3	2	4	3	4	3	4	2	3	3	5	3
1	1	1	1	1	1	1	1	1	1	1	1
2	2	4	2	4	2	2	2	2	2	4	2
2	3	3	2	2	2	2	3	2	2	2	2
3	3	3	3	3	3	3	2	3	3	3	3
2	3	4	3	4	1	3	1	2	1	2	1
2	2	4	2	4	2	2	2	2	2	4	2
2	2	3	2	3	2	2	2	2	2	2	2
3	1	4	2	5	1	2	2	5	2	4	2
3	1	4	4	5	1	4	1	4	3	4	4
3	2	3	3	3	2	3	2	3	2	3	3
3	2	4	3	4	2	3	1	3	2	2	2
2	1	2	3	3	1	1	1	1	2	2	3
3	2	3	3	4	2	4	1	4	2	4	3
4	4	4	5	5	1	3	2	4	5	5	5
4	1	3	4	4	1	4	1	3	3	5	4
2	2	4	2	4	2	2	2	4	3	2	2
3	3	3	3	3	1	3	1	3	2	4	3
1	1	5	2	4	1	1	1	3	1	3	1
2	1	4	2	4	1	1	1	4	1	4	2
2	2	3	3	4	2	3	2	4	3	2	3
1	1	2	1	1	1	1	1	1	1	1	2
2	3	3	2	3	3	2	2	3	3	2	2
4	1	5	1	4	1	4	1	2	1	4	2
1	1	4	1	3	1	1	1	1	3	1	1
5	5	5	5	5	5	5	5	5	5	5	5
2	2	4	2	4	2	3	1	2	2	4	2
2	2	5	2	3	2	2	2	2	2	2	2
3	3	3	3	3	3	3	3	3	3	3	3

3	4	3	5	3	3	3	3	3	4	3	3
3	1	3	4	5	1	5	1	2	4	4	2
1	1	5	1	5	1	1	1	1	1	5	1
2	1	3	1	3	1	3	1	3	1	5	1
3	3	3	3	3	3	3	3	3	3	3	3
1	1	4	2	4	1	1	1	4	4	4	1
3	3	4	3	5	2	2	2	2	2	4	3
2	2	4	2	3	2	3	2	2	2	4	2
1	1	5	1	3	1	1	1	3	1	1	1
3	3	3	3	3	3	3	3	3	3	3	3
3	1	4	2	2	2	2	2	4	2	2	2
2	2	4	2	2	2	2	2	3	2	3	2
2	3	4	2	4	2	4	1	5	3	4	3
1	1	4	2	5	1	4	1	3	2	5	3
3	3	3	3	3	2	3	3	3	2	2	3
3	3	3	3	3	3	3	3	3	3	3	3
2	1	2	3	2	1	2	1	3	2	4	2
4	1	3	1	1	1	1	1	3	1	3	1
3	1	3	3	4	2	3	2	4	2	4	3
2	2	2	2	2	2	4	2	4	2	4	2
3	3	3	3	3	3	3	3	3	3	3	3
3	2	2	2	4	2	2	2	4	3	3	3
4	3	3	4	4	3	4	2	3	3	3	3
1	1	5	2	3	1	1	1	1	1	1	1
3	2	3	3	4	2	2	2	2	2	3	3
2	2	3	2	4	2	4	2	3	2	3	2
1	1	1	1	1	1	1	1	1	1	4	1
3	3	3	3	3	2	2	2	2	2	3	2
3	3	3	3	3	3	3	3	3	3	5	3
5	2	3	5	5	2	5	2	4	4	5	2
3	2	3	4	3	2	3	4	3	2	3	4
1	1	4	1	4	1	4	2	2	4	4	1
1	1	3	3	1	1	1	1	1	1	1	1
1	1	4	1	5	1	3	1	3	1	4	1
4	1	4	3	3	1	1	1	4	1	3	3
2	2	4	3	4	2	3	2	3	3	3	3
3	1	4	3	5	1	4	1	1	3	4	3
5	5	5	5	5	5	5	5	5	5	5	5
2	2	4	2	2	1	2	1	2	1	3	2
2	2	4	1	1	2	2	2	2	4	1	4
2	2	4	2	5	2	2	2	4	4	4	2
1	1	2	1	5	1	5	1	1	1	1	1
1	1	2	1	4	1	1	1	1	1	3	4
2	1	3	2	4	1	3	1	4	2	4	3
2	4	4	2	4	2	2	2	2	2	2	2
2	2	4	3	4	2	4	2	4	2	2	3
1	1	5	3	1	1	1	1	1	1	1	1
3	3	3	3	3	3	3	3	3	3	3	3
1	1	5	2	4	1	1	1	2	2	1	1
1	1	5	1	1	1	1	1	1	1	4	1
4	2	3	3	4	2	4	2	3	2	4	3
4	2	2	4	4	2	4	2	2	2	4	4
2	1	5	4	5	1	5	1	1	1	5	4
2	1	4	4	4	1	5	1	2	1	5	2
1	1	2	1	1	1	1	1	1	1	1	1
2	2	4	2	2	2	2	2	2	2	2	2

2	1	4	2	4	1	2	1	3	1	3	3
1	1	5	1	2	1	1	1	1	1	1	1
1	1	4	3	5	1	1	1	1	1	5	2
3	2	3	2	3	2	2	2	2	2	2	2
2	2	4	2	3	2	2	2	2	2	2	2
3	3	4	4	4	3	4	2	4	4	4	4
3	2	4	2	3	2	2	2	2	3	4	2
2	2	4	3	4	2	2	2	4	2	2	3
3	2	2	3	3	3	2	3	3	3	2	3
1	1	5	5	5	1	5	1	1	1	1	1
2	1	3	1	4	1	4	1	4	2	3	2
3	1	4	4	5	1	5	1	1	1	4	3
1	1	5	1	1	1	1	1	1	1	1	1
3	3	3	3	3	3	3	3	3	3	3	3
1	1	5	1	1	1	1	1	1	1	1	1
2	1	4	2	4	1	4	1	4	4	5	2
1	1	3	1	1	1	1	1	1	1	1	1
2	2	4	2	1	1	1	1	1	1	1	1
3	3	3	3	3	3	3	3	3	3	3	3
5	1	5	5	5	1	1	1	1	1	1	1
3	1	3	2	5	1	3	1	3	4	5	2
2	2	2	2	2	2	2	1	2	2	2	2
1	1	4	1	4	1	4	1	3	1	4	1
3	2	4	3	2	2	2	2	2	2	3	2
3	3	4	3	3	3	3	3	4	3	3	3
2	2	4	2	2	2	2	2	2	2	3	3
2	2	4	3	4	2	4	1	3	2	3	2
3	3	3	3	3	3	3	3	3	3	3	3
2	1	4	3	1	1	1	1	1	1	1	2
2	2	2	2	2	2	2	2	2	2	2	2
2	2	3	3	4	2	4	2	3	2	3	2
2	2	3	2	4	1	3	1	3	2	4	2
3	1	3	3	5	1	5	1	3	2	4	2
3	2	4	3	4	1	3	1	3	3	3	3
2	2	2	2	2	2	2	2	2	2	2	2
3	2	4	3	3	1	2	1	2	1	2	2
1	1	5	2	4	1	3	1	1	1	2	1
3	2	3	2	4	2	4	2	3	3	3	3
3	4	3	3	3	3	3	3	3	3	3	2
2	1	4	2	3	1	2	1	2	1	3	2
3	3	3	3	3	3	3	3	3	3	3	3
1	2	3	3	4	1	2	2	2	2	2	2
1	1	3	1	4	1	1	1	1	1	4	1
2	2	4	2	3	2	2	2	2	2	4	2
1	1	3	2	1	1	1	1	1	1	2	1
3	2	2	3	3	1	2	1	2	2	4	3
2	1	4	2	3	1	2	1	1	1	3	1
1	1	5	1	1	1	1	1	1	1	1	1
2	2	3	3	4	2	4	2	4	2	4	3
2	1	5	1	4	1	1	1	1	4	1	2
3	2	4	2	3	2	2	2	2	2	2	2
1	1	4	2	4	1	2	1	2	2	3	2
2	2	4	2	2	2	2	2	2	2	2	1
3	2	3	2	3	2	2	2	2	2	3	3
3	2	4	3	4	2	4	2	4	3	4	3
3	3	3	3	3	3	3	3	3	3	3	3

2	1	4	3	4	2	4	1	3	2	4	3
3	1	3	1	3	1	3	1	4	1	1	1
3	3	3	3	3	3	3	3	3	3	3	3
1	1	3	2	3	1	3	1	2	3	2	1
1	1	4	1	5	1	1	1	1	1	3	1
1	1	4	1	1	1	1	1	1	1	3	1
2	2	4	2	2	2	1	1	1	1	1	2
3	3	3	3	3	3	3	3	3	3	3	3
3	4	4	3	4	2	4	2	1	3	4	3
1	1	4	1	1	1	1	1	1	1	1	1
1	1	3	1	3	1	1	1	3	1	3	1
4	1	2	3	5	1	4	1	1	1	5	3
3	2	5	2	2	2	2	2	2	2	2	2
1	1	4	1	2	1	1	1	1	1	2	1
4	3	4	4	4	3	4	3	4	4	5	4
3	1	4	2	4	1	4	1	1	1	4	1
2	2	4	1	4	2	3	1	2	4	4	2
3	3	4	3	4	4	3	2	3	4	4	1
2	1	2	3	4	1	2	1	1	1	4	2
4	5	4	3	4	3	4	3	3	2	2	3
1	1	3	1	3	1	1	1	1	1	1	1
2	3	3	3	4	3	3	3	3	3	2	3
1	1	5	1	1	1	1	1	1	1	1	1
2	2	4	3	4	2	3	2	2	2	4	3
1	1	5	1	1	1	1	1	1	1	1	1
2	2	4	2	3	2	3	2	2	2	3	2
1	1	4	4	1	1	1	1	2	1	4	3
3	3	3	3	3	3	5	3	3	3	3	3
1	1	5	1	1	1	1	1	1	1	1	1
3	2	3	3	3	1	2	1	3	1	2	4
2	1	4	3	4	1	3	1	1	1	4	2
3	3	3	3	3	3	3	3	3	3	3	3
2	1	4	2	3	1	3	1	3	2	3	2
2	2	5	1	1	1	1	1	1	2	1	1
1	1	5	1	1	1	1	1	1	1	5	1
2	3	3	2	3	2	2	2	2	2	2	2
2	2	4	2	2	2	2	1	2	2	3	2
1	1	5	1	1	1	1	1	1	1	1	1
2	1	3	2	4	3	4	2	3	4	4	1
2	1	4	1	3	1	1	1	1	1	3	1
3	2	4	3	4	2	4	2	3	2	3	2
2	1	5	1	5	1	1	1	2	2	4	1
1	1	5	1	1	1	1	1	1	1	1	1
2	2	3	2	4	2	2	2	2	2	3	2
2	2	4	2	2	1	3	2	2	2	4	2
3	3	3	3	3	3	3	3	3	3	3	3
1	1	4	1	1	1	1	1	1	1	3	1
2	1	5	3	4	1	2	1	1	1	1	1
4	1	4	2	4	1	4	1	4	4	4	3
1	1	2	1	1	1	1	1	1	1	1	1
1	1	5	1	1	1	1	1	1	1	3	1
1	1	5	1	1	1	1	1	1	1	1	1
2	1	4	3	1	1	1	1	1	1	1	1
1	1	5	1	5	1	1	1	1	1	3	3
3	1	2	2	3	1	3	1	2	3	3	3
4	2	4	4	5	2	5	2	3	2	5	4

3	3	4	2	3	2	4	2	2	2	4	2
5	1	1	1	4	1	1	1	4	2	5	2
1	1	4	1	1	1	1	1	1	1	1	1
1	1	3	1	1	1	1	1	1	1	1	1
4	4	4	3	4	4	3	3	4	4	3	3
3	3	3	3	3	3	3	3	3	3	3	3
3	3	3	3	3	3	3	3	3	3	3	3
1	1	3	5	5	1	2	1	1	1	5	1
3	3	3	3	3	3	3	3	3	3	3	3
4	2	4	3	4	2	4	2	4	4	4	4
3	2	4	2	5	1	1	1	4	4	4	2
4	2	3	4	4	1	4	1	4	2	5	3
2	2	3	2	3	2	2	2	3	2	3	2
2	2	3	2	2	2	2	2	2	2	2	2
2	1	4	3	4	2	3	1	2	2	3	3
2	2	4	2	3	2	2	2	2	2	2	2
2	2	4	2	4	2	4	1	1	1	2	1
4	3	3	2	4	3	3	3	3	3	3	2
3	3	3	3	3	3	3	3	3	3	3	3
3	3	4	3	3	2	3	1	3	3	3	3
2	1	4	2	3	2	2	1	3	3	2	2
2	2	4	2	4	2	4	2	2	2	3	1
2	2	4	2	2	2	2	2	2	3	4	2
2	1	3	1	3	1	1	1	1	2	3	2
1	1	3	1	1	1	1	1	1	1	1	1
2	2	4	3	4	2	3	2	3	4	3	2
3	3	3	3	4	3	3	3	3	3	3	3
4	2	4	3	4	1	1	1	4	3	4	4
2	2	3	2	2	2	2	2	2	2	2	2
5	1	5	1	5	5	1	3	5	4	5	1
3	2	3	3	3	2	2	2	2	2	2	2
1	1	5	1	5	1	5	1	5	1	5	3
2	1	5	1	1	1	1	1	3	1	3	1
3	3	3	3	3	3	3	3	3	3	3	3
1	1	5	1	3	1	3	1	1	1	1	1
3	3	4	3	3	3	3	2	4	3	2	2
1	1	5	1	1	1	1	1	1	1	1	1
4	2	4	2	4	2	3	1	2	4	4	2
2	1	5	2	4	1	3	1	4	1	5	3
2	2	2	1	2	1	2	1	2	1	2	2
3	3	3	3	3	2	2	2	2	2	3	2
1	1	5	1	3	1	2	1	1	1	4	1
1	1	3	1	3	1	4	1	3	2	4	3
1	1	2	1	2	1	1	1	1	1	3	1
2	2	4	2	3	2	2	2	2	2	2	2
2	2	3	2	3	2	3	2	2	3	2	3
3	3	2	3	3	2	3	4	3	3	3	3
3	1	3	2	5	3	5	2	5	2	5	4
3	2	3	2	4	2	2	2	2	2	3	2
1	1	3	1	1	1	1	1	1	1	1	1
2	5	4	2	4	5	3	4	4	3	4	1
2	2	4	2	4	2	2	1	2	3	3	2
1	1	5	1	1	1	1	1	1	1	1	1
3	3	3	3	3	3	3	3	3	3	3	3
1	1	3	3	5	1	1	1	1	1	1	1
2	2	4	2	2	2	2	2	2	2	2	2

1	1	5	1	3	1	1	1	1	1	1	1
1	1	4	2	1	1	1	1	2	2	3	1
3	2	3	3	4	3	4	3	3	3	4	3
3	1	5	1	3	1	3	1	3	1	3	1
2	2	4	2	2	2	2	2	2	2	2	2
1	1	3	1	3	1	1	1	1	1	3	1
3	2	3	2	3	2	3	2	2	3	3	3
4	3	4	3	2	3	4	3	3	4	3	3
2	1	3	3	3	1	4	1	3	3	3	3
1	1	1	1	1	1	1	1	1	1	1	1
1	1	4	1	2	2	2	1	1	1	4	1
3	2	3	3	4	1	5	1	3	1	4	4
1	1	3	1	1	1	1	1	1	1	2	1
3	3	3	3	3	2	3	2	4	3	3	3
5	1	4	4	5	2	5	2	5	5	2	5
2	1	4	3	4	1	1	1	1	2	4	2
2	1	2	3	4	1	2	1	1	3	3	3
3	3	3	3	4	3	3	3	3	3	4	3
3	3	3	3	3	3	3	3	3	3	3	3
1	1	5	1	1	1	1	1	1	1	1	1
2	2	5	2	5	2	2	2	2	2	5	2
5	5	5	5	5	1	5	1	1	1	5	5
3	1	3	3	4	2	4	1	4	3	4	3
3	3	3	3	3	3	3	3	3	3	3	3
4	3	4	3	4	3	3	2	4	2	4	3
1	1	5	1	1	1	1	1	1	1	1	1
2	1	4	2	4	1	3	1	2	1	4	1
2	2	4	2	3	2	3	2	2	2	2	2
2	2	3	2	2	1	1	1	1	1	1	2
2	1	4	3	3	2	4	3	4	3	3	2
2	1	4	1	5	1	3	1	1	1	4	1
4	4	4	4	4	4	4	4	4	4	4	4
3	3	3	1	1	1	1	1	1	1	1	1
3	2	3	3	3	3	3	3	3	3	3	3
2	2	4	2	2	2	2	2	2	2	2	2
3	3	3	3	3	3	3	3	3	3	3	3
2	3	3	3	3	2	2	2	2	3	3	2
4	4	2	1	4	4	4	4	4	4	4	4
1	1	4	1	2	1	1	1	1	1	3	1
1	1	5	5	5	1	5	5	5	5	5	5
3	2	4	2	2	2	2	2	3	2	2	2
4	2	4	2	3	2	4	2	2	2	4	2
3	3	3	3	3	3	3	3	3	3	3	3
2	1	3	4	5	1	4	1	1	5	5	1
3	3	3	3	3	1	3	1	3	3	3	3
2	2	3	3	3	2	3	2	3	3	3	2
2	2	4	2	3	2	2	2	2	2	2	1
3	3	3	3	3	3	3	3	3	3	3	3
4	2	4	3	4	2	4	2	3	2	4	3
1	1	5	1	1	1	1	1	1	1	1	1
3	2	2	3	4	2	3	1	3	2	4	3
1	1	3	1	4	1	3	1	1	1	4	1
3	3	3	3	3	3	3	3	3	3	3	3
3	1	5	2	1	1	1	1	2	1	2	1
3	1	2	3	3	1	1	1	1	1	1	1
1	1	5	1	1	1	1	1	1	1	1	1

4	1	4	3	4	1	3	1	4	1	4	3
1	1	4	1	1	1	1	1	1	1	1	1
2	2	4	2	4	1	4	1	3	2	4	3
3	4	3	4	3	4	3	4	3	4	3	2
3	1	3	2	5	2	5	1	4	1	3	3
2	1	2	1	1	1	2	1	2	1	4	2
2	1	4	1	2	1	1	1	1	3	3	3
5	1	2	3	5	1	5	1	5	5	5	3
2	2	4	2	4	2	2	2	2	2	3	2
3	3	3	3	5	3	5	3	4	3	4	3
3	2	4	3	4	2	4	2	2	2	2	2
1	1	4	1	1	1	1	1	1	1	3	4
2	1	2	2	3	2	3	2	2	2	4	2
2	2	4	2	2	2	2	2	2	1	1	1
1	1	3	1	1	1	1	1	1	1	1	1
4	1	3	1	4	1	1	1	3	1	5	1
2	1	3	2	3	1	1	1	1	1	1	1
2	3	3	1	4	2	3	2	3	4	3	1
3	3	3	3	3	3	3	3	3	3	3	3
1	1	4	1	2	1	1	1	3	2	4	2
1	1	5	1	5	3	5	1	5	5	5	1
2	1	4	2	2	2	2	1	2	1	3	2
1	1	5	1	1	1	1	1	1	1	1	1
2	1	4	2	2	1	2	1	4	1	3	1
2	1	4	2	2	1	1	1	1	1	1	1
1	1	2	1	1	1	1	1	1	1	5	1
5	5	5	5	5	5	5	5	5	5	5	5
1	1	5	1	1	1	1	1	1	1	1	1
3	2	3	4	3	2	3	2	3	2	4	3
3	1	4	2	3	1	3	1	3	2	4	2
2	2	4	3	4	2	4	1	3	1	4	2
1	1	3	1	2	1	1	1	1	1	2	1
3	2	4	3	4	2	4	1	1	3	3	2
4	2	3	3	4	2	4	2	4	2	4	3
1	1	4	2	1	1	1	1	2	2	3	2
3	3	3	3	3	3	3	3	3	3	3	3
1	1	4	3	4	1	4	1	1	1	3	3
1	1	5	1	5	1	1	1	1	1	1	1
3	3	3	3	3	3	3	3	3	3	3	3
2	2	4	2	2	4	2	2	2	2	2	2
1	1	5	1	5	1	1	1	1	1	2	1
3	2	3	3	3	2	3	2	4	3	4	3
2	2	3	2	4	1	2	1	2	2	2	2
3	2	4	3	3	2	3	2	3	3	3	3
2	1	5	1	3	1	3	1	2	1	5	1
4	1	4	4	4	1	4	1	4	1	4	4
2	2	4	3	4	2	2	2	4	2	4	2
3	2	3	2	3	2	3	2	2	3	3	2
5	5	5	5	5	5	5	5	5	5	5	5
4	2	4	4	4	2	4	1	4	1	4	2
3	2	3	3	3	2	3	2	3	3	3	3
1	1	4	3	3	1	1	1	3	1	4	1
3	3	3	3	3	3	3	3	3	3	3	3
3	2	2	3	4	2	4	2	4	3	4	3
1	1	4	1	4	1	1	1	3	2	4	1
4	2	4	2	2	2	4	2	2	2	2	2

3	3	3	3	3	3	3	3	3	3	3	3
3	2	4	3	3	2	3	2	3	3	4	2
3	3	2	3	4	3	3	2	3	3	3	3
3	3	3	3	3	3	3	3	3	3	3	3
2	1	4	1	3	1	3	1	3	2	4	1
2	1	3	3	4	1	5	1	1	5	4	2
2	1	2	2	3	1	1	1	1	1	3	1
4	3	4	3	5	1	5	1	3	3	3	3
3	2	4	3	4	2	4	1	3	4	4	2
1	1	3	1	1	1	1	1	1	1	1	1
2	1	3	2	4	1	1	1	2	2	5	3
1	1	5	1	1	1	1	1	1	1	1	1
4	4	4	4	4	4	4	4	4	4	4	4
3	3	3	3	3	3	3	3	3	3	3	3
1	1	5	1	5	1	1	1	1	1	5	1
3	2	3	2	3	2	2	1	2	2	3	2
5	1	2	3	3	1	2	1	1	1	4	3
2	2	2	2	3	2	2	2	2	2	2	2
3	4	4	4	4	2	4	2	2	2	3	4
4	3	4	3	3	2	3	2	2	2	4	2
2	1	5	2	1	1	1	1	1	1	1	1
2	2	4	2	4	2	3	1	3	1	1	2
2	1	4	2	5	1	5	1	5	1	5	5
1	1	4	2	3	1	2	1	3	4	4	2
2	2	4	2	4	2	4	2	2	1	4	2
3	3	3	3	3	3	3	3	3	3	3	3
5	5	5	5	5	5	5	5	5	5	5	5
3	2	4	2	3	2	2	2	2	2	2	2
2	2	4	2	3	2	3	2	2	2	3	2
2	2	4	4	2	2	2	2	2	2	4	4
3	2	3	3	4	2	3	2	2	3	4	4
2	2	2	2	4	2	2	2	4	2	3	3
2	2	4	1	3	1	3	1	4	5	4	2
2	2	4	2	3	2	2	2	2	2	2	2
1	1	5	1	1	1	1	1	1	1	4	1
2	1	3	2	3	1	2	1	2	1	4	3
3	1	5	3	3	1	4	1	1	1	2	2
3	1	3	2	3	1	2	1	4	3	4	2
1	1	5	1	1	1	1	1	1	1	1	1
2	2	4	2	4	2	5	1	1	1	5	1
2	1	3	3	3	1	5	1	3	3	3	3
1	1	4	1	2	1	1	1	2	1	1	1
5	5	5	4	5	5	5	5	5	4	5	5
4	4	4	4	4	4	4	4	4	4	4	4
4	1	4	3	4	1	2	1	3	3	4	2
1	1	4	2	5	3	2	2	1	2	5	1
3	1	1	1	3	1	1	1	1	1	4	1
4	3	3	4	5	3	5	3	5	4	3	5
3	3	3	3	3	3	3	3	3	3	3	3
3	4	3	4	4	4	4	4	4	4	3	4
3	3	3	3	3	3	3	3	3	3	3	3
3	3	3	3	3	2	2	2	3	3	3	2
2	3	3	3	4	1	2	1	1	3	4	3
3	3	3	3	3	3	3	3	3	3	3	3
1	1	5	1	1	1	1	1	1	1	1	1
5	4	5	4	3	4	3	2	1	4	4	2

1	1	4	3	3	1	1	1	1	1	1	2
3	2	4	3	4	2	4	1	4	2	4	3
3	1	3	2	5	1	3	1	3	2	5	2
1	1	4	1	1	1	3	1	1	1	1	1
2	1	4	1	2	2	2	1	3	1	3	2
3	3	3	3	3	3	3	3	3	3	3	3
1	1	4	1	2	1	1	1	3	2	2	1
4	1	2	3	3	1	2	1	4	2	4	3
2	1	2	3	2	1	4	1	2	1	3	2
2	1	4	3	3	1	1	1	1	1	2	3
1	1	2	1	1	1	2	1	1	1	1	1
2	2	5	2	3	2	2	1	2	1	3	1
2	2	4	2	4	2	2	2	3	2	4	2
1	1	5	1	1	1	1	1	1	1	1	1
3	3	3	3	3	3	3	3	3	3	3	3
3	2	2	2	3	2	2	1	1	1	3	2
2	2	4	3	4	2	3	1	4	2	4	2
3	4	3	3	4	2	2	2	3	2	3	3
2	2	3	3	4	2	4	2	3	3	4	2
3	3	3	3	3	1	3	2	3	3	3	3
5	5	5	5	5	5	5	5	5	5	5	5
2	1	5	1	1	1	1	1	1	1	1	1
3	3	3	3	3	3	3	3	3	3	3	3
1	1	5	1	3	1	2	1	1	1	3	1
3	3	3	3	3	3	3	3	3	3	3	3
1	1	5	1	1	1	1	1	1	1	1	1
1	1	4	2	3	1	2	1	1	1	2	1
3	2	3	2	4	2	3	2	2	2	3	2
1	1	5	1	1	1	1	1	1	1	1	1
3	1	3	2	4	2	3	1	4	1	4	1
1	1	5	1	1	1	1	1	1	1	1	1
4	3	3	1	2	4	3	4	4	4	3	2
2	1	4	2	3	1	3	1	2	3	2	2
3	2	3	3	3	3	3	2	3	3	2	3
4	2	2	3	4	3	4	3	3	2	4	3
3	2	3	2	4	2	3	2	5	3	4	3
3	3	3	4	4	3	4	3	4	3	4	4
1	1	5	1	1	1	1	1	1	1	1	1
3	2	4	3	4	2	3	2	2	2	3	2
4	4	4	4	4	4	4	4	4	4	4	4
3	1	4	1	3	1	3	1	3	3	3	3
3	3	3	3	4	2	4	2	3	2	4	4
3	3	3	3	3	2	3	3	2	3	4	3
1	1	4	1	1	1	1	1	1	1	1	1
4	4	2	2	4	4	4	4	4	4	4	2
2	2	3	2	4	2	4	2	4	2	3	2
1	1	5	1	1	1	1	1	1	1	1	1
3	2	3	3	4	1	4	1	4	3	4	3
1	1	5	1	1	1	1	1	3	1	5	1
5	5	5	5	5	5	5	5	5	5	5	5
3	1	2	3	3	2	5	1	2	3	5	2
3	2	3	2	4	2	3	1	3	2	2	2
3	3	3	3	3	3	3	1	3	2	2	3
2	1	4	1	1	1	1	1	1	1	1	1
4	2	3	3	5	2	4	2	4	2	4	3
3	3	3	3	3	2	3	3	3	3	3	3

3	1	3	3	4	1	4	1	4	2	5	3
1	1	5	1	1	1	1	1	1	1	1	1
1	1	5	4	5	1	2	1	1	1	5	4
2	1	4	1	1	1	3	1	2	2	1	3
3	3	3	3	4	2	4	2	4	3	4	3
4	2	3	3	3	2	3	2	2	2	3	3
3	1	2	3	4	2	3	1	1	3	4	3
3	4	3	3	4	3	4	4	4	2	3	2
3	3	3	3	3	3	3	3	3	3	3	3
2	1	4	3	5	1	4	1	2	1	2	1
1	1	5	2	1	1	1	1	1	1	2	1
2	2	4	2	2	2	2	2	2	2	2	2
3	2	4	3	3	2	3	2	3	3	3	3
3	2	3	2	4	2	4	2	3	3	4	3
1	1	5	1	1	1	1	1	1	3	1	1
3	2	2	3	2	3	2	3	3	2	3	3
3	3	3	3	4	3	3	1	4	3	4	2
5	2	5	3	5	2	5	2	3	3	5	5
3	2	4	2	4	2	2	1	2	2	2	2
3	3	3	3	3	1	3	1	3	1	3	3
2	2	4	2	2	2	2	2	2	2	3	3
3	3	3	3	3	3	3	3	3	5	3	2
1	1	4	1	1	1	1	1	1	1	1	1
1	1	5	1	1	1	1	1	1	1	1	1
2	2	4	4	4	3	4	2	2	2	4	4
2	2	4	3	4	1	1	1	1	1	3	3
2	2	4	2	3	1	2	2	3	3	3	3
3	2	3	2	4	1	2	1	3	1	4	2
2	1	5	2	2	1	2	1	1	1	1	1
2	2	3	2	3	2	2	2	2	3	3	3
3	1	4	3	5	1	2	1	1	4	3	3
2	1	4	3	5	1	5	1	5	3	5	4
2	1	4	4	4	1	1	1	1	1	3	3
1	3	3	1	1	1	2	1	2	2	2	2
5	1	5	1	5	1	1	1	1	1	1	1
2	1	2	2	4	1	3	1	2	1	2	1
2	3	3	1	4	2	3	2	3	3	4	3
2	2	3	2	2	2	2	2	3	2	3	2
1	1	5	1	1	1	1	1	1	1	1	1
1	1	3	3	3	1	3	1	4	1	3	1
3	2	4	3	3	3	3	3	4	3	3	3
3	3	3	3	3	3	3	3	3	3	3	3
1	3	5	3	5	1	1	1	5	4	4	1
2	1	5	1	5	1	4	1	1	1	5	1
2	2	4	2	3	1	2	1	2	4	4	4
3	2	4	3	4	2	4	2	4	3	4	4
2	1	3	3	3	3	3	1	3	1	3	2
3	2	3	4	4	3	3	3	2	3	4	4
3	4	3	4	4	4	4	4	4	3	4	4
3	2	4	5	5	2	5	1	2	4	5	4
3	3	3	3	3	5	3	2	2	5	5	4
5	5	5	5	5	5	5	5	5	5	5	5
3	4	4	3	3	3	3	3	3	4	4	3
3	3	3	3	3	3	3	3	3	3	3	3
2	2	3	2	3	2	3	2	3	2	4	2
1	1	5	1	1	1	1	1	1	1	5	1

2	1	2	3	4	1	2	1	1	1	3	2
1	1	5	1	5	1	1	1	1	1	1	1
2	1	3	1	1	1	1	1	1	1	1	1
3	3	3	3	3	3	3	3	3	3	3	3
3	3	3	3	3	3	3	3	3	3	3	3
2	2	4	2	4	2	3	2	2	2	4	2
3	3	3	3	4	3	3	2	5	4	4	3
3	3	3	2	4	2	3	2	3	2	2	2
2	2	3	2	3	2	2	2	2	2	3	2
4	2	3	3	3	2	3	2	2	4	2	2
2	1	5	2	4	1	1	1	2	1	4	2
3	2	3	2	3	2	2	2	3	2	3	2
1	1	5	1	1	1	1	1	1	1	1	1
1	1	5	1	1	1	1	1	1	1	3	1
2	2	4	2	4	2	2	2	2	2	4	2
3	3	3	3	3	2	2	2	2	2	2	3
3	3	3	3	3	3	3	3	3	3	3	3
2	1	3	2	4	2	2	1	2	1	1	1
1	1	5	1	1	1	1	1	1	1	2	1
1	1	4	1	1	1	2	1	1	1	1	1
1	1	5	1	1	1	1	1	1	1	1	1
3	3	3	3	3	2	4	1	2	3	5	4
3	3	3	3	3	3	3	3	3	3	3	3
3	3	3	3	3	3	3	3	3	3	3	3
2	2	4	2	3	2	2	2	2	2	2	2
3	3	3	3	3	3	3	3	3	3	3	3
4	1	3	3	3	1	3	1	3	1	5	5
1	1	5	1	1	1	1	1	1	1	1	1
1	1	5	1	2	1	1	1	1	1	1	1
5	3	1	1	5	5	5	5	5	5	5	5
3	1	5	1	1	1	1	1	1	1	1	1
1	1	5	1	1	1	1	1	1	1	1	1
2	1	3	3	3	2	2	1	1	1	3	2
2	2	5	2	2	2	2	1	2	1	2	1
4	1	4	3	5	1	1	1	1	1	5	2
3	1	3	2	3	2	2	2	2	2	3	2
3	3	3	3	3	3	3	3	3	3	3	3
3	1	3	3	5	1	3	1	5	4	4	3
4	2	4	3	2	4	4	3	4	3	2	4
4	4	4	3	4	4	4	3	4	3	4	3
3	3	3	3	3	1	3	1	3	1	4	2
3	3	3	3	3	3	3	3	3	3	3	3
1	1	3	1	3	1	1	1	4	1	4	1
2	2	3	3	4	2	2	2	1	1	4	3
1	1	4	1	3	1	1	1	1	1	1	1
3	2	3	2	3	2	2	2	3	4	3	3
3	1	3	2	3	1	2	1	3	1	3	1
5	5	5	5	5	5	5	5	5	5	5	5
3	3	3	3	3	3	3	3	3	3	3	3
4	3	4	4	4	3	4	3	4	3	4	3
4	2	3	3	5	2	5	2	4	2	5	3
1	1	4	1	1	1	3	1	1	1	1	1
3	2	2	3	4	2	4	2	2	2	2	2
2	2	2	2	4	2	4	2	2	2	4	2
3	2	3	3	3	2	3	2	3	2	3	3
3	2	3	3	3	2	3	2	2	2	2	3

3	1	3	2	1	1	1	1	2	1	4	2
2	2	3	2	3	2	2	2	2	2	3	2
4	3	2	2	3	2	3	2	3	2	3	3
5	5	5	1	1	1	5	1	1	1	1	1
3	2	3	3	4	2	4	2	2	2	3	4
1	1	5	1	2	2	2	2	2	1	3	1
3	3	3	3	3	3	3	3	3	3	3	3
2	2	4	3	4	2	4	2	4	2	2	2
1	1	1	1	1	1	1	1	1	1	1	1
3	1	3	1	1	1	1	1	1	1	1	1
3	1	4	2	4	1	1	1	1	1	4	2
2	1	5	1	4	1	3	1	3	1	4	3
3	3	3	3	3	3	3	3	3	3	3	2
3	1	1	3	1	1	1	1	5	3	5	3
2	1	4	1	3	1	1	1	1	1	1	1
4	2	2	5	3	3	4	1	5	2	5	5
3	2	3	3	5	3	3	3	3	3	4	3
2	2	3	2	3	2	2	2	3	2	3	3
3	1	5	1	2	1	1	1	4	3	3	1
2	1	3	1	1	1	1	1	1	1	1	1
3	3	3	2	4	3	4	3	4	3	3	2
1	1	3	2	3	1	1	1	2	1	1	1
1	1	4	1	4	1	1	1	1	1	4	1
3	3	3	3	3	3	3	2	3	3	3	3
3	2	2	3	4	2	3	2	3	2	2	3
1	1	5	1	1	1	1	1	1	1	1	1
3	4	3	4	4	1	3	2	2	3	3	3
2	1	3	1	3	2	2	1	2	1	1	1
1	1	4	3	1	1	1	1	1	4	1	1
4	1	3	3	4	2	3	1	3	2	4	3
4	2	3	2	4	2	4	2	3	3	4	2
2	2	4	2	3	2	2	2	1	1	3	1
1	1	1	1	1	1	1	1	1	1	5	1
3	2	4	2	2	1	2	1	2	2	3	2
3	2	3	2	3	2	2	2	2	2	3	3
3	3	3	3	3	3	3	3	3	3	3	3
1	1	5	1	1	1	1	1	1	1	1	1
2	2	2	2	2	2	2	2	2	2	2	1
1	1	4	2	2	1	2	1	1	1	2	1
1	1	2	1	3	1	1	1	1	1	1	1
3	2	4	2	4	2	4	2	4	2	4	3
4	5	4	4	4	2	4	1	2	3	3	3
3	2	4	3	3	2	3	2	3	2	2	2
3	3	3	3	3	3	3	3	3	3	3	3
3	1	4	1	3	1	4	1	3	1	1	1
2	3	4	3	4	2	3	2	4	3	3	3
3	3	3	3	4	4	4	3	4	4	3	3
2	2	3	2	2	2	2	2	2	2	3	2
3	4	4	2	3	4	4	2	4	4	4	3

总分(total)

595

570
539
469
569
570
536
539
514
642
524
549
582
557
567
533
581
594
545
561
622
641
581
537
522
561
496
594
508
514
508
581
546
588
553
620
537
517
556
552
560
524
540
601
559
543
594
724
492
550

561
539
561
563
520
559
567
578
524
546
558
528
541
593
549
531
582
511
495
507
532
523
530
562
468
534
498
463
552
499
581
548
571
615
533
519
637
523
550
513
562
528
578
585
517
568
487
590
608
474
499
582
573
564
555
543

520
509
547
549
540
577
539
560
567
539
576
541
543
600
584
531
769
539
509
621
556
534
513
555
587
527
533
559
636
513
510
560
478
531
569
566
499
576
581
508
535
525
556
545
462
499
542
508
595
729
554
583
559
572
567
579

553
580
611
525
644
599
577
556
569
591
576
568
529
577
571
568
565
519
558
641
601
597
576
523
576
580
568
597
600
612
515
624
538
545
542
542
482
530
521
536
521
581
614
541
568
576
514
568
500
540
607
568
581
562
521
553

592
562
522
582
639
555
550
589
565
507
572
540
505
499
553
520
538
575
539
533
538
577
590
545
614
555
566
545
570
535
586
629
530
546
551
598
569
529
557
521
604
547
565
548
571
600
603
563
505
587
625
530
547
574
563
463

613
532
574
561
533
544
584
569
559
564
555
517
600
544
682
769
615
524
552
557
546
578
546
592
533
587
559
614
517
551
494
543
516
540
595
568
536
538
493
493
495
555
546
525
592
562
759
542
590
503
568
393
521
527
519
560

665
539
603
501
575
565
566
520
538
525
548
589
634
644
518
565
553
563
541
559
622
543
531
584
536
528
506
609
535
519
552
540
545
500
473
603
636
573
535
585
619
605
602
564
610
570
533
512
517
546
545
510
769
569
579
530

508
600
537
558
555
536
573
547
533
546
566
524
539
631
530
523
540
578
594
566
636
512
629
553
529
518
539
552
589
630
510
599
570
558
495
553
570
770
544
582
551
540
577
562
572
542
523
481
613
549
579
597
573
583
526
558

540
539
522
527
527
608
567
564
557
543
563
601
539
522
531
602
568
511
534
599
568
501
556
566
546
498
527
519
543
521
581
517
631
555
442
543
561
553
564
547
646
555
562
578
518
502
567
537
518
571
513
563
560
492
577
536

560
544
585
496
551
497
500
572
560
537
515
609
609
558
635
588
547
549
566
707
518
511
541
558
528
574
546
564
569
562
557
527
485
528
545
499
526
543
562
542
587
593
537
520
538
557
555
593
602
579
553
533
549
561
537
620

533
658
535
528
600
517
592
625
547
598
585
534
551
538
579
511
532
616
585
562
533
553
539
562
527
579
525
561
540
600
503
623
568
550
569
593
549
550
576
523
561
573
546
516
575
506
561
639
539
543
594
547
549
570
609
536

532
537
569
568
566
546
499
528
533
598
555
602
528
544
643
527
566
564
533
533
599
687
542
488
577
520
553
496
495
545
579
602
537
535
527
597
533
579
575
749
519
543
535
632
558
547
586
558
616
537
585
513
556
550
583
531

577
532
604
572
604
540
575
602
595
553
590
539
601
572
532
563
576
528
639
524
623
537
540
549
481
597
769
570
621
560
557
528
538
535
567
595
511
580
587
546
545
549
548
597
583
617
587
551
769
574
532
538
527
585
591
531

532
542
564
528
534
555
553
657
582
517
581
542
644
574
581
550
573
513
645
566
566
511
622
565
521
521
769
592
574
508
613
546
588
525
567
537
625
555
545
572
611
541
713
647
576
595
532
551
580
623
523
522
533
550
517
611

556
582
511
582
526
601
573
592
584
571
488
575
522
536
590
570
548
545
597
570
763
534
623
615
588
551
541
507
538
554
535
551
505
537
571
550
566
559
530
613
572
574
560
493
550
534
535
529
560
589
564
532
514
492
561
511

607
536
624
542
564
553
549
547
519
571
545
522
569
554
535
525
558
653
546
503
540
559
544
553
639
501
524
561
542
533
561
638
513
483
565
582
548
500
567
565
601
537
566
591
558
616
546
568
651
601
555
741
563
573
539
585

578
601
524
587
546
562
569
583
495
589
600
522
541
554
543
546
590
514
533
550
533
555
532
574
528
502
646
526
526
592
542
539
536
567
560
504
528
645
545
622
562
580
559
535
538
525
545
769
595
555
614
521
545
534
500
533

560
552
540
681
518
572
549
537
523
561
583
565
567
607
555
611
532
521
552
499
562
518
550
527
521
538
648
543
541
621
549
529
581
620
548
592
513
509
575
532
559
636
533
620
543
568
592
505
597
